# Supplementary material for: Cationic indium catalysis as a powerful tool for generating α-alkyl propargyl cations for SN1 reactions
Source: Commun Chem. 2023 Dec 16;6:279. doi: 10.1038/s42004-023-01048-4 (PMC10725475; doi:10.1038/s42004-023-01048-4)
Supplement: Supplementary file 2 — Supplementary Material [file 42004_2023_1048_MOESM2_ESM.pdf]

Supporting Information

Cationic indium catalysis as a powerful tool for generating α-alkyl propargyl cations for S_N1 reactions

Mitsuhiro Yoshimatsu,[1]* Hiroki Goto,[1] Rintaro Saito,[1] Kodai Iguchi,[1] Manoka Kikuchi,[1] Hiroaki Wasada,[2] Yoshiharu Sawada[3]

[1]Department of Chemistry, Faculty of Education, Gifu University, Yanagido 1-1, Gifu 501-1193, Japan

[2]Department of Chemistry, Faculty of Regional Study, Gifu University, Yanagido 1-1, 501-1193 Gifu, Japan, [3]Technical center, Nagoya University, 464-8601 Nagoya, Japan. email: yoshimatsu.mituhiro.j3@f.gifu-u.ac.jp

Table of Contents

1. General Information. p.2
2. Preparations of propargyl alcohols. p.3
3. Screening for reaction conditions for the synthesis of 1,2,3-trimethoxy-4-(1-phenylpent-1-yn-3-yl)benzene (2a) (Table 1). p.15
4. Product derivatization (Scheme 2). p.49

Supplementary Methods

1. General Information.

Unless otherwise noted, all reagents were purchased from commercial suppliers and used without further purification. Analytical thin layer chromatography (TLC) was performed using silica gel precorted glass plates and visualized by ultraviolet radiation (254 nm). Flash column chromatography on silica gel was performed using silica gel (particle size 0.063-0.200 mm) under air pressure. Melting points were determined on a J-Science Lab. Micro melting point apparatus and uncorrected. ^{1}H NMR and ^{13}C NMR were recorded at 25 ^{o}C on a JEOL ECA 400 (400 MHz), ECA500 (500 MHz), and ECA600 (600 MHz) spectrometers ($CDCl_3$ as solvent) at Gifu University. Chemical shifts for ^{1}H NMR spectra are reported as δ in units of parts per million (ppm) downfield from tetramethylsilane (δ 0.00) . Multiplicities were given as: s (singlet); d (doublet); t (triplet); q (quartet); dd (double of doublets); dt (double of triplets); m (multiplets), etc. Coupling constants are reported as a *J* value in Hz. ^{13}C NMR spectra are reported as δ in units of parts per million (ppm) downfield from tetramethylsilane and relative to the signal of chloroform-d (δ 77.16, triplet). IR spectra were determined on a FT-IR infrared spectrometer and are expressed in reciprocal centimeters. EI mass spectra (MS) were obtained using JEOL MS-700 spectrometer with direct-insertion probe at 70 eV. High resolution mass determinations were obtained on the JMSD300 JMS 2000 on line system. ESI measurements and their high reolution mass were performed using DART system. The elemental analyses were performed using a JM-10 CHN-corder on the J-Science Lab. HPLC analysis was performed on the agilent 100 with DAICEL chiral analytical column (4.6 mmΦ* 250 mm length). The propargyl alcohols were prepared by two methods as shown in Supplementary Figure 1. *(S)*- and *(R)* **-1a** were prepared by the Stecko's method as shown in Supplementary Figure 2. HPLC analyses were shown in Supplementary Figure 3.

1. Preparations of propargyl alcohols.

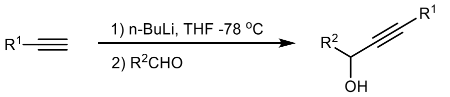

1a (R^1=Ph/R^2=Et)(51%)
1b (R^1=Ph/R^2=iBu)(84%)
1c (R^1=Ph/R^2=tBu)(76%)
1d (R^1=Ph/R^2=cyclohexyl)(96%)
1e (R^1=Ph/R^2=cyclopentyl)(68%)
1f (R^1=Ph/R^2=cyclooctyl)(59%)
1g (R^1=Ph/R^2=cyclododecyl)(54%)
1i (R^1=*m*-MeC_6H_4/R^2=Me)(72%)
1j (R^1=*o*-MeC_6H_4/R^2=Me)(55%)
1k (R^1=*p*-$MeOC_6H_4$/R^2=Et)(44%)
1l (R^1=*m*-$MeOC_6H_4$/R^2=Et)(66%)
1m (R^1=*o*-$MeOC_6H_4$/R^2=Et)(55%)
1n (R^1=2,4,6-$Me_3C_6H_2$/R_2=Et)(44%)
1q (R_1=2,4-$Cl_2C_6H_3$/R_2=$(CH_2)_4Me$)(66%)
1r (R_1=p-ClC_6H_5/R_2=$(CH_2)_4Me$)(69%)
1s (R_1=m-ClC_6H_4/R_2=$(CH_2)_4Me$)(74%)
1δ (R_1=$(CH_2)_5Me$/R_2=$(CH_2)_2Me$)(72%)
1ε (R_1=$(CH_2)_9Me$/R_2=$(CH_2)_2Me$)(81%)

1κ (R^1=Ph/R^2=*o*-$O_2NC_6H_4$)(49%)
1λ (R^1=Ph/R^2=*p*-$O_2NC_6H_4$)(63%)
1μ (R^1=Ph/R^2=*m*-$O_2NC_6H_4$)(56%)
1ν (R^1=Ph/R^2=*p*-$CF_3C_6H_4$)(-)
1ξ (R^1=Ph/R^2=*p*-$AcNHC_6H_4$)(63%)
1ο (R^1=$(CH_2)_4Me$/R^2=*p*-$O_2NC_6H_4$)(63%)
1π (R^1=$(CH_2)_4Me$/R^2=*o*-ClC_6H_4)(?%)
1ρ (R^1=$(CH_2)_4Me$/R^2=*p*-$MeO_2CC_6H_4$)(79%)

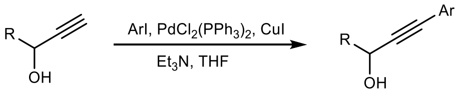

1h (Ar=4-MeC_6H_4/R=$(CH_2)_4Me$)(quant)
1o (Ar=*p*-FC_6H_4/R=$(CH_2)Me$)(85%)
1p (Ar=2,4-$F_2C_6H_3$/R=$(CH_2)_4Me$)(58%)
1t (Ar=*o*-FC_6H_4/R=Me)(quant)
1u (Ar=*p*-BrC_6H_5/R=$(CH_2)_4Me$)(92%)
1v (Ar=*o*-$CF_3C_6H_5$/R=Me)(57%)
1w (Ar=*m*-$CF_3C_6H_4$/R=Me)(50%)
1x (Ar=4-$MeO_2CC_6H_4$/R=$(CH_2)_4Me$)(46%)
1y (Ar=*o*-NCC_6H_4/R=$(CH_2)_4Me$)(quant)
1z (Ar=m-NCC_6H_4/R=$(CH_2)_4Me$)(quant)
1α (Ar=*p*-NCC_6H_4/R=Me)(78%)

1β (Ar=4-$O_2NC_6H_4$/R=Me)(78%)
1γ (Ar=2-Me-5-$O_2NC_6H_4$/R=$(CH_2)_4Me$)(87%)

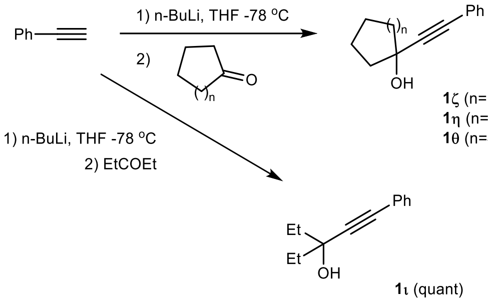

1ζ (n=1) (31%)
1η (n=2) (78%)
1θ (n=4) (38%)

1ι (quant)

Supplementary Figure 1. Preparations of propargyl alcohols 1

1-phenylpent-1-yn-3-ol (1a).

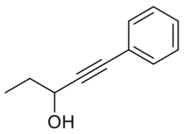

To a THF (50.0 mL) solution of ethynylbenzene (5.00 g, 49.0 mmol) was added 2.6 M *n*-BuLi (17.2 mL, 46.5 mmol) at -78 °C under an Ar atmosphere. After 10 min stirring of the mixture, a THF (10.0 mL) solution of propionaldehyde (2.70 g, 46.5 mmol) was added dropwise to the mixture. The whole was stirred for 10 min and poured into water (100 mL). The organic layer was separated and the aqueous layer was extracted with AcOEt. The organic layer was washed with NH_4Claq (50.0 mL×2) and dried

over $MgSO_4$. The solvent was removed under reduced pressure. The residue was purified by column chromatography on silica gel eluting with AcOEt-*n*-hexane (1:20 to1:10) to give 1-phenylpent-1-yn-3-ol (**1a**) (5.45 g, 73%) as a yellow oil.

IR (KBr) ν 3338, 2968, 2934, 2876, 2233, 1599, 1490, 1443, 1339, 1098, 1070, 1049, 1013, 963, 915, 866, 755, 691, 543, 524, 454; ^{1}H NMR (600 MHz, $CDCl_3$) δ 1.06 (3H, t, *J* = 7.6 Hz, CH_3), 1.78-1.85 (2H, m, CH_2), 2.31 (1H, brs, OH), 4.53-4.56 (1H, m, CH), 7.28-7.30 (3H, m, ArH), 7.42-7.43 (2H, m, ArH); ^{13}C NMR (150 MHz, $CDCl_3$) δ 9.5 (q), 30.9 (t), 64.1 (d), 84.8 (s), 90.0 (s), 122.6 (s), 128.2 (d×2), 128.3 (d), 131.6 (d×2); EIMS m/z 160 (M^+); high resolution mass calcd for $C_{11}H_{12}O$: 160.08882. Found: 160.0917. Anal. Calcd for $C_{11}H_{12}O$: C, 82.46; H, 7.55. Found: C, 82.38; H, 7.48.

(X. Chang, J. Zhang, L. Peng, C. Guo Nature Commun. 2021, 12, 299.)

5-methyl-1-phenylhex-1-yn-3-ol (1b).

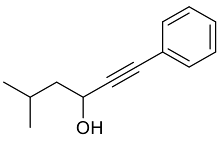

To a THF (10.0 mL) solution of ethynylbenzene (1.00 g, 9.79 mmol) was added 2.6 M n-BuLi (3.14 mL, 8.16 mmol) at -76 °C under an Ar atmosphere. After 10 min stirring the mixture, a THF (5.00 mL) solution of isovaleraldehyde (0.703 g, 8.16 mmol) was added dropwise to the mixture. After 10 min stirring the mixture, a NH_4Claq (10.0 mL) was added to the mixture and poured into water (100 mL). The organic layer was separated and the aqueous layer was extracted with AcOEt. The organic layer was washed with NH_4Claq (50.0 mL×2) and dried over $MgSO_4$. The solvent was removed under reduced pressure. The residue was purified by column chromatography on silica gel eluting with AcOEt-*n*-hexane (1:20 to1:10) to give 5-methyl-1-phenylhex-1-yn-3-ol (**1b**) (1.28 g, 84%) as pale yellow solids (mp below 30 °C).

IR (KBr) ν 3433, 2959, 2926, 2869, 2854, 2218, 1600, 1541, 1489, 1468, 1456, 1443, 1368, 1331, 1216, 1134, 1056, 1028, 1010, 995, 937, 756, 691; ^{1}H NMR (600 MHz, $CDCl_3$) δ 0.97 (3H, d, *J* = 6.9 Hz, CH_3), 0.98 (3H, d, *J* = 6.9 Hz, CH_3), 1.66 (1H, quin, *J* = 6.9 Hz, CH_2), 1.73 (1H, quin, *J* = 6.9 Hz, CH_2), 1.92 (1H, quin, *J* = 6.9 Hz, CH), 2.09 (1H, brs, OH), 4.64 (1H, t, *J* = 6.9 Hz, CH), 7.28-7.31 (3H, m, ArH), 7.41-7.43 (2H, m, ArH); ^{13}C NMR (150 MHz, $CDCl_3$) δ 22.5 (q), 22.6 (q), 24.8 (d), 46.9 (t), 61.5 (d), 84.7 (s), 90.4 (s), 122.7 (s), 128.2 (d×2), 128.3 (d), 131.6 (d×2); EIMS m/z 188 (M^+). Anal. Calcd for $C_{13}H_{16}O$(+ 2 / 3 H_2O): C; 77.96, H; 8.72. Found: C; 77.98, H; 9.00.

(Q. Wang, J. A. May Org Lett 2020, 22, 9579-9584)

4,4-dimethyl-1-phenylpent-1-yn-3-ol (1c).

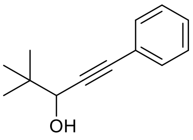

To a THF (10.0 mL) solution of ethynylbenzene (1.00 g, 9.79 mmol) was added 2.6 M n-BuLi (3.14 mL, 8.16 mmol) at -76 °C under an Ar atmosphere. After 10 min stirring the mixture, a THF (5.00 mL) solution of pivalaldehyde (0.703 g, 8.16 mmol) was added dropwise to the mixture. After 10 min stirring the mixture, a NH_4Claq (10.0 mL) was added to the mixture and poured into water (100 mL). The organic layer was separated and the aqueous layer was extracted with AcOEt. The organic layer was washed with NH_4Claq (50.0 mL×2) and dried over $MgSO_4$. The solvent was removed under reduced pressure. The residue was purified by column chromatography on silica gel eluting with AcOEt-n-hexane (1:20 to1:10) to give 4,4-dimethyl-1-phenylpent-1-yn-3-ol (**1c**) (1.17 g, 76%) as pale yellow prisms (mp below 30 °C).

IR (KBr) ν 3399, 3082, 3060, 2965, 2869, 2223, 1950, 1879, 1599, 1573, 1489, 1479, 1465, 1443, 1393, 1364, 1322, 1240, 1221, 1187, 1056, 1008, 979, 935, 914, 876, 756, 691, 604, 555, 525, 511; ^{1}H NMR (600 MHz, $CDCl_3$) δ 1.06 (9H, s, CH_3×3), 1.89 (1H, d, *J* = 5.5 Hz, OH), 4.24 (1H, d, *J* = 5.5 Hz, CH), 7.29-7.31 (3H, m, ArH), 7.42-7.44 (2H, m, ArH); ^{13}C NMR (150 MHz, $CDCl_3$) δ 25.4 (q×3), 36.1 (s), 71.8 (d), 85.7 (s), 88.9 (s), 122.7 (s), 128.25 (d×2), 128.29 (d), 131.7 (d×2); EIMS m/z 188 (M^+). Anal. Calcd for $C_{13}H_{16}O$: C; 82.94, H; 8.57. Found: C; 82.91, H; 8.82.

(Q. Wang, J. A. May Org Lett 2020, 22, 9579-9584.)

1-cyclohexyl-3-phenylprop-2-yn-1-ol (1d).

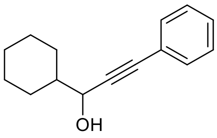

To a THF (10.0 mL) solution of ethynylbenzene (1.00 g, 9.79 mmol) was added 2.6 M n-BuLi (3.14 mL, 8.16 mmol) at -76 °C under an Ar atmosphere. After 10 min stirring the mixture, a THF (5.00 mL) solution of cyclohexanecarbaldehyde (0.915 g, 3.14 mmol) was added dropwise to the mixture. After 10 min stirring the mixture, a NH_4Claq (10.0 mL) was added to the mixture and poured into water (100 mL). The organic layer was separated and the aqueous layer was extracted with AcOEt. The organic layer was washed with NH_4Claq (50.0 mL×2) and dried over $MgSO_4$. The solvent was removed under reduced pressure. The residue was purified by column chromatography on silica gel eluting with AcOEt-*n*-hexane (1:20 to1:10) to give the titled compound (**1d**) (1.65 g, 95%) as a yellow oil.

IR (KBr) ν 3389, 2926, 2852, 1599, 1490, 1449, 1096, 1083, 1071, 1029, 983, 893, 756, 691, 578, 527; ^{1}H NMR (600 MHz, $CDCl_3$) δ 1.10-1.22 (2H, m, CH_2), 1.24-1.32 (2H, m, CH_2), 1.62-1.70 (2H, m, CH_2), 1.78 (1H, brs, CH), 1.80 (1H, brs, CH), 1.91 (1H, brs, CH), 1.93 (1H, brs, CH), 2.04 (1H, brs, OH), 4.24 (1H, d, *J* = 5.8 Hz, CH), 7.29-7.30 (3H, m, ArH), 7.43-7.44 (2H, m, ArH); ^{13}C NMR (150 MHz, $CDCl_3$) δ 25.9 (t×2), 26.4 (t), 28.2 (t), 28.6 (t), 44.3 (d), 67.6 (d), 85.6 (s), 89.2 (s), 122.7 (s), 128.2 (d×2), 128.3 (d), 131.7 (d×2); EIMS m/z 214 (M^+). Anal. Calcd for $C_{15}H_{18}O$(+ 1 / 12 H_2O): C; 83.48, H; 8.49. Found: C; 83.45, H; 8.64.

(F. D. Lu, D. Liu, L. Zhu, L.-Q. LuQ. Yang, Q.-Q. Zhou, Y. Wei, Y. Lan, W.-J. Xiao J. Am. Chem. Soc. 2019, 141, 6167-6172.)

Preparation of 1-cyclopentyl-3-phenylprop-2-yn-1-ol (**1e**).

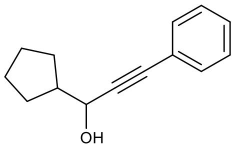

To a THF (10.0 mL) solution of ethynylbenzene (1.00 g, 10.0 mmol) was added *n*-butyllithium (2.90 mL, 7.50 mmol) at -78 ºC under an Ar atmosphere. After 5 min stirring, cyclopentanecarboxaldehyde (500 mg, 5.10 mmol) in THF (10.0 mL) was added to the mixture. The whole was stirred for 5 min and then poured into water (50 mL). The organic layer was separated and the aqueous layer was extracted with AcOEt. The combined organic layer was dried over $MgSO_4$. The solvent was removed under reduced pressure. The residue was purified by column chromatography on silica gel eluting with AcOEt-*n*-hexane (1:20) to give 1-cyclopentyl-3-phenylprop-2-yn-1-ol (**1e**) (689 mg, 68%) as a colorless oil.

IR (KBr, cm^{-1}) ν 3432, 3389, 3367, 3348, 2952, 2867, 1704, 1599, 1490, 1443, 1323, 1030, 915, 756, 691; ^{1}H NMR (600 MHz, $CDCl_3$) δ 1.47-1.53 (1H, m, CH_2), 1.54-1.60 (3H, m, CH_2), 1.67-1.68 (2H, m, CH_2), 1.82-1.85 (2H, m, CH_2), 1.90 (1H, brs, OH), 2.29 (1H, sex, *J* = 7.6 Hz, CH), 4.47 (1H, brs, CH), 7.29-7.31 (3H, m, ArH), 7.42-7.43 (2H, m, ArH); ^{13}C NMR (150 MHz, $CDCl_3$) δ 25.6 (t), 25.7 (t), 28.3 (t), 28.9 (t), 46.2 (d), 66.6 (d), 84.7 (s), 89.7 (s), 122.7 (s), 128.1 (d×3), 131.6 (d×2); EIMS m/z 200 (M^+), 131 (M^+ -C_5H_{11}); high resolution mass calcd for $C_{14}H_{16}O$: 200.1201, found m/z 200.1210. Anal. Calcd for $C_{14}H_{16}O$(+ 3 / 20 H_2O): C, 82.84; H, 8.09. Found: C, 82.80; H, 7.94.

Preparation of 1-cyclooctyl-3-phenylprop-2-yn-1-ol (**1f**).

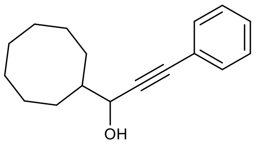

To a THF (10.0 mL) solution of ethynylbenzene (664 mg, 6.50 mmol) was added *n*-butyllithium (2.10 mL, 5.50 mmol) at -78 ºC under an Ar atmosphere. After 5 min stirring, cyclooctanecarboxaldehyde (700 mg, 5.00 mmol) in THF (3.00 mL) was added to the mixture. The whole was stirred for 5 min and then poured into water (50 mL). The organic layer was separated and the aqueous layer was extracted with AcOEt. The combined organic layer was dried over $MgSO_4$. The solvent was removed under reduced pressure. The residue was purified by column chromatography on silica gel eluting with AcOEt-*n*-hexane (1:20) to give 1-cyclooctyl-3-phenylprop-2-yn-1-ol (**1f**) (746 mg, 59%) as a yellow oil.

IR (KBr, cm^{-1}) ν 3367, 2922, 2850, 1706, 1599, 1490, 1444, 1069, 1038, 1005, 756, 691, 526; ^{1}H NMR (600 MHz, $CDCl_3$) δ 1.45-1.52 (7H, m, CH_2), 1.62 (3H, brd, *J* = 8.3 Hz, CH_2), 1.75-1.81 (3H, m, CH_2), 1.87-1.89 (2H, m, CH_2), 1.97 (1H, brs, CH), 4.40 (1H, s, OH), 7.30-7.31 (3H, m, ArH), 7.43 (2H, dd, *J* = 2.1 and 5.5 Hz, ArH); ^{13}C NMR (150 MHz, $CDCl_3$) δ 25.8 (t), 25.9 (t), 26.6 (t×3), 28.6 (t), 29.0 (t), 43.9 (d), 68.5 (d), 85.2 (s), 89.5 (s), 122.8 (s), 128.2 (d×2), 128.3 (d), 131.7 (d×2); EIMS m/z 225 (M^+ -OH), 199 (M^+ -Pr), 185 (M^+-Bu), 171 (M^+ -C_5H_{11}), 131 (M^+ -C_8H_{15}); high resolution mass calcd for $C_{17}H_{22}O$: 242.1671, found m/z 242.1652. Anal. Calcd for $C_{17}H_{22}O$ (+1/10 H_2O): C,83.63; H, 9.17. Found: C, 83.78; H, 9.35.

Preparation of 1-cyclododecyl-3-phenylprop-2-yn-1-ol (**1g**).

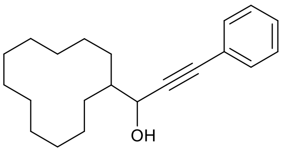

To a THF (7.00 mL) solution of ethynylbenzene (706 mg, 6.91 mmol) was added *n*-butyllithium (2.30 mL, 6.91 mmol) at -78 ºC under an Ar atmosphere. After 5 min stirring, cyclododecanecarboxaldehyde (1.13 g, 5.76 mmol) in THF (5.00 mL) was added to the mixture. The whole was stirred for 5 min and then poured into water (50 mL). The organic layer was separated and the aqueous layer was extracted with AcOEt. The combined organic layer was dried over $MgSO_4$. The solvent was removed under reduced pressure. The residue was purified by column chromatography on silica gel eluting with AcOEt-*n*-hexane (1:20) to give 1-cyclododecyl-3-phenylprop-2-yn-1-ol (**1g**) (801 mg, 47%) as white crystals (mp 75-75.5 ºC, from *n*-hexane).

IR (KBr, cm^{-1}) ν 3405, 2929, 2862, 1709, 1490, 1470, 1443, 1028, 755, 690; ^{1}H NMR (600 MHz, $CDCl_3$) δ 1.31-1.58 (22H, m, CH_2), 1.84-1.85 (1H, m, CH_2), 1.88-1.90 (1H, m, OH), 4.52-4.54 (1H, m, CH), 7.30-7.31 (3H, m, ArH), 7.42-7.43 (2H, m, ArH); ^{13}C NMR (150 MHz, $CDCl_3$) δ 22.4 (t), 22.5 (t), 23.4 (t), 23.6 (t×2), 23.8 (t×2), 24.2 (t), 24.3 (t), 25.5 (t), 25.7 (t), 40.4 (d), 66.2 (d), 85.4 (s), 89.7 (s), 122.8 (s), 128.2 (d×3), 131.7 (d×2); EIMS m/z 298 (M^+), 267 (M^+ -Et), 131 (M^+ -$C_{12}H_{23}$). Anal. Calcd for $C_{21}H_{30}O$: C, 84.51; H, 10.13. Found: C, 84.69; H, 10.40.

Preparation of 1-(*p*-tolyl)oct-1-yn-3-ol (**1h**)

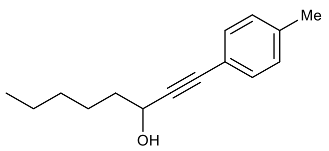

To a THF (12.5 mL) and triethylamine (12.5 mL) 1-iodo-4methylbenzene (1.09 g, 5.00 mmol) was added oct-1-yn-3-ol (947 mg, 7.50 mmol), copper(I) iodide (47.6 mg, 0.250 mmol), and $PdCl_2(PPh_3)_2$ (70.2 mg, 0.100 mmol) at room temperature for 24 h. The reaction mixture was filtered through a pad of celite with chloroform and the solvent was removed under reduced pressure The residue was purified by column chromatography on silica gel eluting with AcOEt-*n*-hexane (1:20 to 1:10) to give 1-(*p*-tolyl)oct-1-yn-3-ol (**1h**) (1.11 g, quant) as a brown oil.

IR (KBr, cm^{-1}) ν 3419, 2956, 2926, 2854, 1509, 1464, 1362, 1262, 1218, 1023, 817, 761; ^{1}H NMR (600 MHz, $CDCl_3$) δ 0.91 (3H, t, *J* = 7.6

Hz, CH_3), 1.34 (4H, q, J = 3.4 Hz, CH_2×2), 1.50-1.52 (2H, m, CH_2), 1.75-1.81 (2H, m, CH_2), 1.99 (1H, brs, OH), 2.34 (3H, s, CH_3), 4.58 (1H, t, J = 6.9 Hz, CH), 7.10 (2H, d, J = 7.5 Hz, ArH), 7.32 (2H, d, J = 8.2 Hz, ArH); ^{13}C NMR (150 MHz, $CDCl_3$) δ14.0 (q), 21.4 (q), 22.6 (t), 24.9 (t), 31.5 (t), 37.9 (t), 63.0 (d), 84.9 (s), 89.5 (s), 119.6 (s), 129.0 (d×2), 131.5 (d×2), 138.4 (s); EIMS m/z 216 (M^+).

Anal. Calcd for $C_{15}H_{20}O$ (+ 3 / 20 H_2O): C; 82.26, H; 9.34. Found: C; 82.11, H; 9.50.

(A.S. K. Hashmi, T. Wang, S. Shi, M. Rudolph *J. Org. Chem.* **2012**, *77*, 7761-7767.)

Preparation of 4-(*m*-tolyl)but-3-yn-2-ol (**1i**).

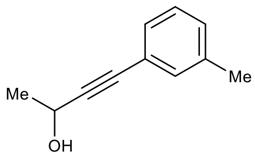

To a DMF (7.00 mL)-triethylamine (10.4 g, 0.103 mol) solution of 3-butyn-2-ol (1.00 g, 14.3 mmol), and *m*-iodotoluene (3.11 g, 14.3 mmol) was added copper iodide (545 mg, 2.86 mmol) and bis(triphenylphosphine)palladium dichloride (1.01 g, 1.43 mmol) at room temperature. The reaction mixture was stirred for 1 h and then poured into water (50 mL). The organic layer was separated and the aqueous layer was extracted with AcOEt. The combined organic layer was dried over $MgSO_4$. The solvent was removed under reduced pressure. The residue was purified by column chromatography on silica gel eluting with AcOEt-*n*-hexane (1:20) to give 4-(*m*-tolyl)but-3-yn-2-ol (**1i**) (855 mg, 66%) as a pale yellow oil.

IR (KBr, cm^{-1}) ν 3360 (OH), 2981, 2925, 2857, 2219, 1602, 1486, 1451, 1370, 1330, 1281, 1106, 1076, 1040, 953, 889, 784, 761; ^{1}H NMR (600 MHz, $CDCl_3$) δ 1.54 (3H, d, J = 6.4 Hz, Me), 2.31 (3H, s, Me), 4.71-4.78 (1H, m, CH), 7.11 (1H, d, J = 7.3 Hz, ArH), 7.16-7.22 (2H, m, ArH), 7.25 (1H, brs, ArH); ^{13}C NMR (150 MHz, $CDCl_3$) δ 21.1 (q), 24.4 (q), 58.8 (d), 84.1 (s), 90.6 (s), 122.3 (s), 128.1 (d), 128.7 (d), 129.2 (d), 132.2 (d), 137.9 (s); EIMS m/z 160 (M^+); high resolution mass calcd for $C_{11}H_{12}O$: 160.0888, found m/z 160.0904. Anal. Calcd for $C_{12}H_{14}O_2$(+1/4H_2O): C, 80.21; H, 7.65. Found: C, 80.24; H, 7.38.

Preparation of 4-(*o*-tolyl)but-3-yn-2-ol (**1j**).

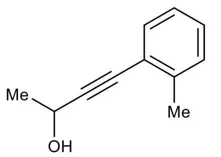

To a DMF (2.00 mL)-triethylamine (3.01 g, 29.8 mmol) solution of 3-butyn-2-ol (289 mg, 4.13 mmol) and o-iodotoluene (0.900 g, 4.13 mmol) was added copper iodide (0.157 g, 0.826 mmol) and bis(triphenylphosphine)palladium dichloride (0.58 g, 0.826 mmol) at room temperature. The reaction mixture was stirred for 1 h and then poured into water (50 mL). The organic layer was separated and the aqueous layer was extracted with AcOEt. The combined organic layer was dried over $MgSO_4$. The solvent was removed under reduced pressure. The residue was purified by column chromatography on silica gel eluting with AcOEt-*n*-hexane (1:20) to give 4-(*o*-tolyl)but-3-yn-2-ol (**1j**)(855 mg, 66%) as a pale yellow oil.

IR (KBr, cm^{-1}) ν 3379 (OH), 2982, 2926, 2854, 1705, 1456, 1370, 1330, 1251, 1120, 1100, 1076, 1033, 934, 857, 757, 857; ^{1}H NMR (600 MHz, $CDCl_3$) δ 1.55 (3H, d, J = 6.8 Hz, Me), 2.40 (3H, s, Me), 2.71 (1H, d, J = 5.2 Hz, OH), 4.78 (1H, fix, J = 5.8 Hz, CH), 7.09 (1H, t, J = 6.9 Hz, ArH), 7.15-7.20 (2H, m, ArH), 7.37 (1H, d, J = 7.5 Hz, ArH); ^{13}C NMR (150 MHz, $CDCl_3$) δ 20.5 (q), 24.5 (dx2), 58.7 (d), 82.7 (s), 95.0 (s), 122.2 (s), 125.2 (d), 128.3 (d), 129.3 (d), 131.8 (d), 140.1 (s); EIMS m/z 160 (M^+); high resolution mass calcd for $C_{11}H_{12}O$: 160.0888, found m/z 160.0904.

Preparation of 1-(4-methoxyphenyl)pent-1-yn-3-ol (**1k**).

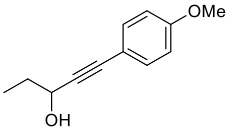

To a THF (7.00 mL) solution of 4-ethynyl-1-methoxybenzene (0.500 g, 3.78 mmol) was added 2.6 M *n*-BuLi (1.21 mL, 3.15 mmol) at -76 °C under an Ar atmosphere. After 10 min stirring the mixture, a THF (3.00 mL) solution of propionaldehyde (0.183 g, 3.15 mmol) was added dropwise to the mixture. After 10 min stirring the mixture, a NH_4Claq (5.00 mL) was added to the mixture and poured into water (50.0 mL). The organic layer was separated and the aqueous layer was extracted with AcOEt. The organic layer was washed with NH_4Claq (30.0 mL×2) and dried over $MgSO_4$. The solvent was removed under reduced pressure. The residue was purified by column chromatography on silica gel eluting with AcOEt-*n*-hexane (1:10 to1:3) to give Preparation of 1-(4-methoxyphenyl)pent-1-yn-3-ol (**1k**) (0.317 g, 44%) as a yellow oil.

IR (KBr) ν 3390, 2967, 2935, 2876, 2839, 2230, 1607, 1569, 1509, 1464, 1442, 1290, 1249, 1173, 1107, 1032, 961, 867, 832, 813, 571, 536; ^{1}H NMR (600 MHz, $CDCl_3$) δ 1.07 (3H, t, J = 7.6 Hz, CH_3), 1.80-1.83 (2H, m, CH_2), 1.96 (1H, brs, OH), 3.80 (3H, s, OCH_3), 4.53 (1H, brd, J = 5.5 Hz, CH), 6.83 (2H, brd, J = 9.0 Hz, ArH), 7.36 (2H, brd, J = 9.0 Hz, ArH); ^{13}C NMR (150 MHz, $CDCl_3$) δ 9.5 (q), 31.0 (t), 55.2 (q), 64.2 (d), 84.8 (s), 88.5 (s), 113.9 (d×2), 114.7 (s), 133.1 (d×2), 159.6 (s); EIMS m/z 190 (M^+). Anal. Calcd for $C_{12}H_{14}O_2$(+ 4 / 5 H_2O): C; 70.43, H; 7.68. Found: C; 70.43, H; 7.02.

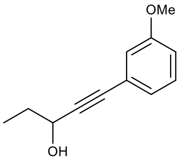

1-(3-methoxyphenyl)pent-1-yn-3-ol (**1l**).

To a THF (15.0 mL) solution of 3-methoxy-1-ethynylbenzene (910 mg, 6.81 mmol) was added *n*-butyllithium (2.30 mL, 6.19 mmol) at -78 °C under an Ar atmosphere. After 5 min stirring of mixture, propanal (0.400 g, 6.81 mmol) in THF (3.00 mL) was added to the mixture. The whole was stirred for 5 min and then poured into water (50 mL). The organic layer was separated and the aqueous layer was extracted with AcOEt. The combined organic layer was dried over $MgSO_4$. The solvent was removed under reduced pressure. The residue was purified by column chromatography on silica gel eluting with AcOEt-*n*-hexane (1:20) to give 1-

(3-methoxyphenyl)pent-1-yn-3-ol (**1l**) (855 mg, 66%) as a pale yellow oil.

IR (KBr, cm^{-1}) ν 3368 (OH), 2967, 2937, 2876, 2835, 2227, 1600, 1576, 1482, 1465, 1421, 1317, 1287, 1204, 1165, 1097, 1046, 1014, 854, 780, 687; ^{1}H NMR (600 MHz, $CDCl_3$) δ 1.08 (3H, t, *J* = 7.4 Hz, Me), 1.78-1.87 (2H, m, CH_2), 2.10 (1H, brs, OH), 3.79 (3H, s, OMe), 4.55 (1H, q, *J* = 5.7 Hz, CH), 6.87 (1H, dd, *J* = 2.3 and 8.0 Hz, ArH), 6.96 (1H, t, *J* = 1.1 Hz, ArH), 7.02 (1H, d, *J* = 8.0 Hz, ArH), 7.21 (1H, dd, *J* = 7.4 and 8.1 Hz, ArH); ^{13}C NMR (150 MHz, $CDCl_3$) δ 9.6 (q), 31.1 (t), 55.4 (q), 64.3 (d), 84.9 (s), 89.9 (s), 115.1 (d), 116.6 (d), 123.8 (s), 124.3 (d), 129.4 (d), 159.4 (s); EIMS m/z 190 (M^+); high resolution mass calcd for $C_{12}H_{14}O_2$: 190.0994, found m/z 190.0944. Anal. Calcd for $C_{12}H_{14}O_2$(+1/10H_2O): C, 75.05; H, 7.45. Found: C, 75.00; H, 7.54.

Preparation of 1-(2-methoxyphenyl)pent-1-yn-3-ol (**1m**).

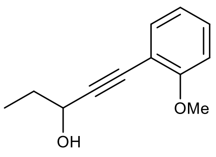

To a THF (15.0 mL) solution of 2-methoxy-1-ethynylbenzene (800 mg, 6.05 mmol) was added *n*-butyllithium (2.00 mL, 5.45 mmol) at -78 °C under an Ar atmosphere. After 5 min stirring of mixture, propanal (0.320 g, 6.05 mmol) in THF (1.50 mL) was added to the mixture. The whole was stirred for 5 min and then poured into water (50 mL). The organic layer was separated and the aqueous layer was extracted with AcOEt. The combined organic layer was dried over $MgSO_4$. The solvent was removed under reduced pressure. The residue was purified by column chromatography on silica gel eluting with AcOEt-*n*-hexane (1:20) to give 1-(2-methoxyphenyl)pent-1-yn-3-ol (**1m**) (631 mg, 55%) as a pale yellow oil.

IR (KBr, cm^{-1}) ν 3390 (OH), 2968, 2876, 2836, 2228, 1597, 1576, 1493, 1464, 1435, 1261, 1235, 1181, 1163, 1121, 1048, 1023, 961, 752; ^{1}H NMR (600 MHz, $CDCl_3$) δ 1.09 (3H, t, *J* = 7.6 Hz, Me), 1.82-1.87 (2H, m, CH_2), 2.42 (1H, brs, OH), 3.86 (3H, s, OMe), 4.60 (1H, t, *J* = 6.2 Hz, CH), 6.85 (1H, brd, *J* = 8.3 Hz, ArH), 6.89 (1H, brt, *J* = 7.5 Hz, ArH), 7.28 (1H, brt, *J* = 6.9 Hz, ArH), 7.38 (1H, d, *J* = 7.6 Hz, ArH); ^{13}C NMR (150 MHz, $CDCl_3$) δ 9.4 (q), 30.9 (t), 55.7 (q), 64.2 (d), 81.0 (s), 94.2 (s), 110.6 (d), 111.8 (s), 120.4 (d), 129.7 (d), 133.6 (d), 159.1 (s); EIMS m/z 190 (M^+); high resolution mass calcd for $C_{12}H_{14}O_2$: 190.0994, found m/z 190.0998. Anal. Calcd for $C_{12}H_{14}O_2$(+1/5H_2O): C, 74.35; H, 7.49. Found: C, 74.21; H, 7.46.

Preparation of 1-(2,4,6-trimethylphenyl)pent-1-yn-3-ol (**1n**).

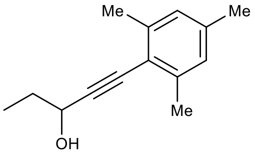

To a THF (10.0 mL) solution of 2,4,6-trimethyl-1-ethynylbenzene (433 mg, 3.00 mmol) was added *n*-butyllithium (0.96 mL, 2.50 mmol) at -78 °C under an Ar atmosphere. After 5 min stirring of mixture, propanal (145 mg, 2.50 mmol) in THF (1.00 mL) was added to the mixture. The whole was stirred for 5 min and then poured into water (50 mL). The organic layer was separated and the aqueous layer was extracted with AcOEt. The combined organic layer was dried over $MgSO_4$. The solvent was removed under reduced pressure. The residue was purified by column chromatography on silica gel eluting with AcOEt-*n*-hexane (1:10) to give 1-(2,4,6-trimethylphenyl)pent-1-yn-3-ol (**1n**) (221 mg, 44%) as a pale yellow oil.

Pale yellow prisms, mp ~30 °C, IR (KBr, cm^{-1}) ν 3340, 2969, 2921, 2876, 2224, 1707, 1610, 1479, 1458, 1377, 1097, 1049, 1015, 961, 852; ^{1}H NMR (600 MHz, $CDCl_3$) δ 1.09 (3H, t, *J* = 6.9 Hz, Me), 1.79-1.89 (2H, m, CH_2), 2.03 (1H, brs, OH), 2.26 (3H, s, Me), 2.38 (6H, s, Mex2), 4.62 (1H, t, *J* = 6.2 Hz, CH), 6.84 (2H, s, ArH); ^{13}C NMR (150 MHz, $CDCl_3$) δ 9.47 (q), 20.9 (qx2), 21.2 (q), 31.2 (t), 64.5 (d), 82.5 (s), 97.7 (s), 119.3 (s), 127.5 (dx2), 137.7 (s), 140.1 (sx2); EIMS m/z 202 (M^+), 173 (M^+ -Et); high resolution mass calcd for $C_{14}H_{18}$: 202.1358, found m/z 202.1357. Anal. Calcd for $C_{14}H_{18}O$(+1/5H_2O): C, 81.67; H, 9.01. Found: C, 81.79; H, 9.13.

Preparation of 1-(4-fluorophenyl)oct-1-yn-3-ol (**1o**).

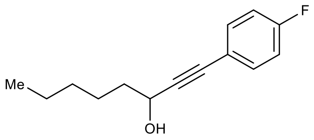

To a DMF (5.00 mL) and triethylamine (7.29 g, 721 mmol) solution of 4-fluoro-1-iodobenzene (2.22 g, 10.0 mmol) was added oct-1-yn-3-ol (1.26 g, 10.0 mmol), copper(I) iodide (0.38 g, 2.00 mmol), and $PdCl_2(PPh_3)_2$ (0.702 g, 1.00 mmol) at room temperature for 0.5 h. The reaction mixture was poured into water (50.0 mL). The organic layer was separated and the aqueous layer was extracted with AcOEt. The combined organic layer was dried over $MgSO_4$. The solvent was removed under reduced pressure. The residue was purified by column chromatography on silica gel eluting with AcOEt-*n*-hexane (1:20) to give 1-(4-fluorophenyl)oct-1-yn-3-ol (**1o**) (1.88 g, 85%) as a brown oil.

IR (KBr, cm^{-1}) ν 3419, 2931, 2860, 2366, 2231, 1602, 1507, 1482, 1231, 1024, 1156, 838, 763; ^{1}H NMR (600 MHz, $CDCl_3$) δ 0.90 (3H, t, *J* = 6.8 Hz, Me), 1.32-1.36 (4H, m, CH_2), 1.47-1.55 (2H, m, CH_2), 1.73-1.82 (2H, m, CH_2), 2.16 (1H, brs, OH), 4.58 (1H, t, *J* = 6.8 Hz, CH), 6.99 (2H, t, *J* = 8.6 Hz, ArH), 7.40 (2H, dd, *J* = 5.8 and 8.5 Hz, ArH); ^{13}C NMR (150 MHz, $CDCl_3$) δ 14.0 (q), 22.5 (t), 24.9 (t), 31.4 (t), 37.8 (t), 62.9 (d), 83.7 (s), 89.9 (s), 115.6 (d, *J* = 21.6 Hz), 118.7 (d, *J* = 3.6 Hz), 133.5 (d, *J* = 8.4 Hz), 162.4 (d, *J* = 49.5 Hz), 164.2 (s); EIMS m/z 220 (M+), high resolution mass calcd for $C_{14}H_{17}FO$: 220.1263, found m/z 220.1249.

Preparation of 1-(2,4-difluorophenyl)oct-1-yn-3-ol (**1p**).

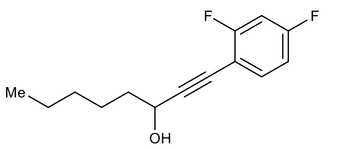

To a THF (12.5 mL) and triethylamine (12.5 mL) solution of 2,4-difluoro-1-iodobenzene (1.20 g, 5.00 mmol) was added oct-1-yn-3-ol (947 mg, 7.50 mmol), copper(I) iodide (47.6 mg, 0.250 mmol), and $PdCl_2(PPh_3)_2$ (70.2 mg, 0.100 mmol) at room temperature for 24 h. The reaction mixture was filtered through a pad of celite with chloroform and the solvent was removed under reduced pressure. The residue was purified by column chromatography on silica gel eluting with AcOEt-*n*-hexane (1:20) to give 1-(2,4-difluorophenyl)oct-1-yn-3-ol (**1p**) (697 mg, 58%) as an orange oil.

IR (KBr, cm^{-1}) ν 3419, 2957, 2930, 2859, 1709, 1618, 1589, 1505, 1467, 1426, 1363, 1299, 1266, 1219, 1145, 1098, 1029, 968, 851, 815, 762, 736, 613; ^{1}H NMR (600 MHz, $CDCl_3$) δ 0.91 (3H, t, *J* = 6.9 Hz, CH_3), 1.35 (4H, t, *J* = 3.4 Hz, CH_2×2), 1.52-1.53 (2H, m, CH_2), 1.76-1.85 (2H, m, CH_2), 2.10 (1H, brd, *J* = 38.5 Hz, OH), 4.61 (1H, q, *J* = 6.2 Hz, CH), 6.81-6.84 (2H, m, ArH), 7.37-7.41 (2H, m, ArH); ^{13}C NMR (150 MHz, $CDCl_3$) δ 13.9 (q), 22.5 (t), 24.8 (t), 31.4 (t), 37.7 (t), 63.0 (d), 77.2 (s), 95.2 (s), 104.2 (t, *J* = 26.0 Hz), 107.6 (dd, *J* = 4.4 Hz and 15.9 Hz), 111.5 (dd, *J* = 4.4 Hz and 21.7 Hz), 134.3 (d, *J* = 10.1 Hz), 162.1 (dd, *J* = 11.6 Hz and 63.6 Hz), 163.7 (dd, *J* = 11.6 Hz and 66.4 Hz); EIMS m/z 238 (M^+). Anal. Calcd for $C_{14}H_{16}F_2O$ (+ 1 / 10 H_2O): C; 70.04, H; 6.80. Found: C; 69.94, H; 6.72.

Preparation of 1-(2,4-dichlorophenyl)oct-1-yn-3-ol (**1q**).

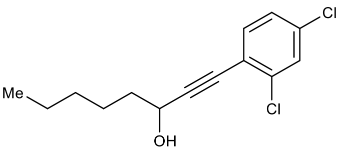

To a THF (12.0 mL) solution of 2,4-dichloro-1-ethynylbenzene (400 mg, 2.34 mmol) was added *n*-butyllithium (0.83 mL, 2.22 mmol) at -78 ºC under an Ar atmosphere. After 5 min stirring of mixture, hexanal (0.22 g, 2.22 mmol) in THF (2.00 mL) was added to the mixture. The whole was stirred for 5 min and then poured into water (50 mL). The organic layer was separated and the aqueous layer was extracted with AcOEt. The combined organic layer was dried over $MgSO_4$. The solvent was removed under reduced pressure. The residue was purified by column chromatography on silica gel eluting with AcOEt-*n*-hexane (1:20) to give 1-(2,4-dichlorophenyl)oct-1-yn-3-ol (**1q**) (421 mg, 66%) as a pale yellow oil.

IR (KBr, cm^{-1}) ν 3320 (OH), 2929, 2859, 1706, 1584, 1547, 1475, 1379, 1101, 1062, 1032, 868, 820; ^{1}H NMR (600 MHz, $CDCl_3$) δ 0.91 (3H, t, *J* = 6.9 Hz, Me), 1.35-1.36 (4H, m, CH_2), 1.52-1.57 (2H, m, CH_2), 1.77-1.84 (2H, m, CH_2), 2.06 (1H, brs, OH), 4.64 (2H, t, *J* = 6.9 Hz, CH), 7.18 (1H, dd, *J* = 2.7 and 8.0 Hz, ArH), 7.38 (1H, d, *J* = 8.0 Hz, ArH), 7.41 (1H, brs, ArH); ^{13}C NMR (150 MHz, $CDCl_3$) δ 14.0 (q), 22.5 (t), 24.8 (t), 31.4 (t), 37.6 (t), 63.0 (d), 80.6 (s), 96.5 (s), 121.2 (s), 126.9 (d), 129.2 (d), 133.9 (d), 134.7 (s), 136.7 (s); EIMS m/z 270 (M^+); high resolution mass calcd for $C_{14}H_{16}Cl_2O$: 270.0578, found m/z 270.0550. Anal. Calcd for $C_{14}H_{16}Cl_2O$: C, 62.01; H, 5.95. Found: C, 61.87; H, 6.16.

Preparation of 1-(4-chlorophenyl)oct-1-yn-3-ol (**1r**).

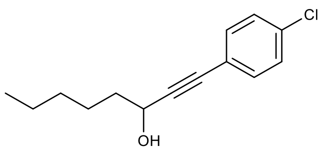

To a THF (10.0 mL) solution of 4-chloro-1-ethynylbenzene (410 mg, 3.00 mmol) was added *n*-butyllithium (1.10 mL, 2.85 mmol) at -78 ºC under an Ar atmosphere. After 5 min stirring of mixture, hexanal (0.29 g, 2.85 mmol) in THF (1.50 mL) was added to the mixture. The whole was stirred for 5 min and then poured into water (50 mL). The organic layer was separated and the aqueous layer was extracted with AcOEt. The combined organic layer was dried over $MgSO_4$. The solvent was removed under reduced pressure. The residue was purified by column chromatography on silica gel eluting with AcOEt-*n*-hexane (1:20) to give 1-(4-chlorophenyl)oct-1-yn-3-ol (**1r**) (487 mg, 69%) as a pale yellow oil.

IR (KBr, cm^{-1}) ν 3359 (OH), 2930, 2860, 2229, 1707, 1467, 1398, 1339, 1255, 092, 1015, 828, 760; ^{1}H NMR (600 MHz, $CDCl_3$) δ 0.91 (3H, t, *J* = 7.3 Hz, Me), 1.32-1.35 (4H, m, CH_2), 1.46-1.53 (2H, m, CH_2), 1.73-1.81 (2H, m, CH_2), 2.02 (1H, brs, OH), 4.58 (2H, t, *J* = 6.9 Hz, CH), 7.27 (2H, dd, *J* = 1.7 and 8.6 Hz, ArH), 7.34 (2H, dd, *J* = 1.7 and 8.5 Hz, ArH); ^{13}C NMR (150 MHz, $CDCl_3$) δ 14.0 (q), 22.5 (t), 24.9 (t), 31.4 (t), 37.8 (t), 63.0 (d), 83.7 (s), 91.2 (s), 121.2 (s), 128.6 (dx2), 132.9 (dx2), 134.4 (s); EIMS m/z 236 (M^+), 201 (M^+-Cl); high resolution mass calcd for $C_{14}H_{17}ClO$: 236.0968, found m/z 236.0964. Anal. Calcd for $C_{14}H_{17}ClO$(+1/9H_2O): C, 70.43; H, 7.27. Found: C, 70.07; H, 7.20.

Preparation of 1-(3-chlorophenyl)pent-1-yn-3-ol (**1s**).

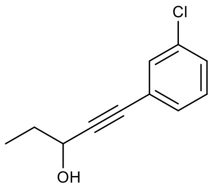

To a THF (7.00 mL) solution of 1-chrolo-3-ethynylbenzene (0.500 g, 3.66 mmol) was added 2.6 M *n*-BuLi (0.704 mL, 1.83 mmol) at -76 ºC under an Ar atmosphere. After 10 min stirring the mixture, a THF (3.00 mL) solution of propionaldehyde (0.106 g, 1.83 mmol) was added dropwise to the mixture. After 10 min stirring the mixture, a NH_4Claq (5.00 mL) was added to the mixture and poured into water (50.0 mL). The organic layer was separated and the aqueous layer was extracted with AcOEt. The organic layer was washed with NH_4Claq (30.0 mL×2) and dried over $MgSO_4$. The solvent was removed under reduced pressure. The residue was purified by column chromatography on silica gel eluting with AcOEt-*n*-hexane (1:20 to1:10) to give 1-(3-chlorophenyl)pent-1-yn-3-ol (**1s**) (0.204 g, 74%) as a yellow oil.

IR (KBr) ν 3348, 2969, 2934, 2877, 1593, 1561, 1488, 1474, 1464, 1407, 1337, 1279, 1242, 1214, 1096, 1079, 1049, 1015, 999, 965, 880, 784, 682; ^{1}H NMR (600 MHz, $CDCl_3$) δ 1.08 (3H, t, *J* = 7.6 Hz, CH_3), 1.78-1.86 (2H, m, CH_2), 2.23 (1H, brs, OH), 4.54 (1H, brt, *J* = 7.6 Hz,

CH), 7.22 (1H, t, J = 7.6 Hz, ArH), 7.28-7.30 (2H, m, ArH), 7.41 (1H, s, ArH); ^{13}C NMR (150 MHz, $CDCl_3$) δ 9.4 (q), 30.8 (t), 64.1 (d), 83.5 (s), 91.2 (s), 124.3 (s), 128.6 (d), 129.5 (d), 129.7 (d), 131.5 (d), 134.0 (s); EIMS m/z 194 (M^+), 196 (M^+). Anal. Calcd for $C_{11}H_{11}OCl$(+ 3 / 4 H_2O): C; 63.47, H; 6.05. Found: C; 63.53, H; 5.41.

Preparation of 1-(2-fluorophenyl)but-1-yn-3-ol (**1t**).

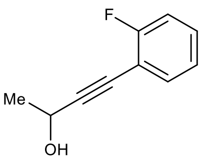

To a DMF (5.00 mL) and triethylamine (7.29 g, 721 mmol) solution of 2-fluoro-1-iodobenzene (2.22 g, 10.0 mmol), and but-1-yn-3-ol (0.650 g, 9.27 mmol) were added copper(I) iodide (0.35 g, 1.85 mmol) and $Pd(PPh_3)_4$ (1.07 g, 0.927 mmol) at room temperature for 0.5 h. The reaction mixture was poured into water (50.0 mL). The organic layer was separated and the aqueous layer was extracted with AcOEt. The combined organic layer was dried over $MgSO_4$. The solvent was removed under reduced pressure. The residue was purified by column chromatography on silica gel eluting with AcOEt-*n*-hexane (1:20) to give 1-(2-fluorophenyl)but-1-yn-3-ol (**1t**) (1.54 g, quant) as a brown oil.

IR (KBr, cm^{-1}) ν 3345, 2984, 2933, 2236, 1575, 1493, 1453, 1371, 1330, 1255, 1215, 1191, 1113, 1099, 1077, 1038, 936, 862, 808, 757; ^{1}H NMR (600 MHz, $CDCl_3$) δ 1.56 (3H, d, J = 6.9 Hz, Me), 2.94 (1H, d, J = 4.5 Hz, OH), 4.77-4.82 (1H, m, CH), 7.03 (1H, d, J = 9.8 Hz, ArH), 7.06 (1H, d, J = 7.4 Hz, ArH), 7.24-7.29 (1H, m, ArH), 7.41 (1H, dt, J = 1.8 and 8.1 Hz, ArH); ^{13}C NMR (150 MHz, $CDCl_3$) δ 24.1 (q), 58.6 (d), 76.7 (s), 96.2 (d, J = 2.4 Hz), 111.1 (d, J = 15.6 Hz), 115.3 (d, J = 21.6 Hz), 123.8 (d, J = 3.6 Hz), 129.9 (d, J = 8.4 Hz), 162.5 (d, J = 250.7 Hz); EIMS m/z 164 (M^+); high resolution mass calcd for $C_{10}H_9FO$: 164.0637, found m/z 164.0614.

Preparation of 1-(4-bromophenyl)oct-1-yn-3-ol (**1u**).

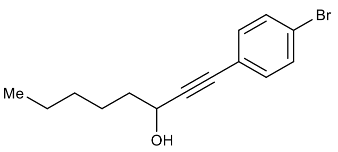

To a THF (6.60 mL) and triethylamine (7.29 g, 63.7 mmol) solution of 4-bromo-1-iodobenzene (2.50 g, 8.84 mmol), oct-1-yn-3-ol (1.12 g, 8.84 mmol) was added copper(I) iodide (0.337 g, 1.77 mmol), $PdCl_2(PPh_3)_2$ (1.02 g, 0.884 mmol) at room temperature for 0.5 h. The reaction mixture was poured into water (50.0 mL). The organic layer was separated and the aqueous layer was extracted with AcOEt. The combined organic layer was dried over $MgSO_4$. The solvent was removed under reduced pressure. The residue was purified by column chromatography on silica gel eluting with AcOEt-*n*-hexane (1:10 to 1:5) to give 1-(4-bromophenyl)oct-1-yn-3-ol (**1u**) (2.58 g, 92%) as a brown oil.

IR (KBr, cm^{-1}) ν 3366, 2928, 2859, 2229, 1487, 1466, 1394, 1070, 1011, 823, 748, 520; ^{1}H NMR (600 MHz, $CDCl_3$) δ 0.90 (3H, t, J = 6.9 Hz, Me), 1.31-1.33 (4H, m, CH_2), 1.46-1.79 (2H, m, CH_2), 2.43 (1H, d, J = 5.1 Hz, OH), 4.57 (1H, q, J = 6.3 Hz, CH), 7.26 (2H, d, J = 8.6 Hz, ArH), 7.41 (2H, d, J = 8.6 Hz, ArH); ^{13}C NMR (150 MHz, $CDCl_3$) δ 13.9 (q), 22.5 (t), 24.8 (t), 31.4 (t), 37.7 (t), 62.8 (d), 83.6 (s), 91.4 (s), 121.6 (s), 122.5 (s), 131.4 (dx2), 133.0 (dx2); EIMS m/z 280 (M^+); high resolution mass calcd for $C_{14}H_{17}BrO$: 280.0463, found m/z 280.0451. Anal. Calcd for $C_{14}H_{17}BrO$(+1/10H_2O): C, 59.80; H, 6.13. Found: C, 59.56; H, 5.86.

Preparation of 4-(3-trifluoromethyl)phenylbut-3-yn-2-ol (**1v**).

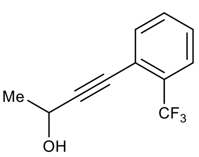

To a DMF (5.00 mL) and triethylamine (7.29 g, 72.1 mmol) solution of 1-butyn-3-ol (700 mg, 10.0 mmol) and 2-iodobenzotrifluride (2.72 g, 10.0 mmol) was added copper(I) iodide (0.38 g, 2.00 mmol), $PdCl_2(PPh_3)_2$ (0.702 g, 1.00 mmol) at room temperature for 0.5 h. The reaction mixture was poured into water (50.0 mL). The organic layer was separated and the aqueous layer was extracted with AcOEt. The combined organic layer was dried over MgSO4. The solvent was removed under reduced pressure The residue was purified by column chromatography on silica gel eluting with AcOEt-*n*-hexane (1:20 to 1:10) to give 4-(3-trifluoromethyl)phenylbut-3-yn-2-ol (**1v**) (1.23 g, 77%) as a brown oil.

IR (KBr, cm^{-1}) ν 3389 (OH), 2986, 2935, 2235, 1708, 1604, 1574, 1492, 1451, 1371, 1319, 1267, 1134, 1114, 1061, 1034, 936, 858, 767, 651; ^{13}C NMR (150 MHz, $CDCl_3$) δ 1.57 (3H, t, J = 6.9 Hz, Me), 1.81 (1H, brs, OH), 4.78 (1H, q, J = 6.3 Hz, CH), 7.41 (1H, t, J = 7.4 Hz, ArH), 7.48 (1H, t, J = 7.4 Hz, ArH), 7.56 (1H, d, J = 7.4 Hz, ArH), 7.64 (1H, d, J = 7.4 Hz, ArH); ^{13}C NMR (150 MHz, $CDCl_3$) δ 24.0 (q), 58.8 (d), 80.0 (s), 96.5 (s), 120.8 (s), 123.4 (q, J = 273.5 Hz, CF_3), 125.7 (q, J = 4.8 Hz, ArH), 128.1 (d), 131.3 (d), 131.6 (q, J = 30.0 Hz), 133.9 (d); EIMS m/z 214 (M^+); high resolution mass calcd for $C_{11}H_9F_3O$: 214.0606, found m/z 214.0608. Anal. Calcd for $C_{11}H_9F_3O$(+1/7H_2O): C, 60.95; H, 4.32. Found: C, 60.73; H, 4.10.

Preparation of 4-(3-trifluoromethyl)phenylbut-3-yn-2-ol (**1w**).

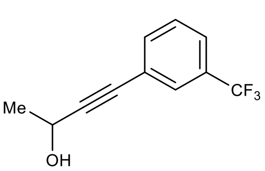

To a DMF (5.00 mL) and triethylamine (7.29 g, 72.1 mmol) solution of 1-butyn-3-ol (700 mg, 10.0 mmol) and 3-iodobenzotrifluride (2.72 g, 10.0 mmol) was added copper(I) iodide (0.38 g, 2.00 mmol), $PdCl_2(PPh_3)_2$ (0.702 g, 1.00 mmol) at room temperature for 0.5 h. The reaction mixture was poured into water (50.0 mL). The organic layer was separated and the aqueous layer was extracted with AcOEt. The combined organic layer was dried over $MgSO_4$. The solvent was removed under reduced pressure The residue was purified by column chromatography on silica gel eluting with AcOEt-*n*-hexane (1:20 to 1:10) to give 4-(3-trifluoromethyl)phenylbut-3-yn-2-ol (**1w**) (1.08 g, 50.4%)

as a brown oil.

IR (KBr, cm^{-1}) ν 3409 (OH), 2985, 2236, 1709, 1433, 1335, 1236, 1169, 1130, 1073, 1038, 948, 903, 803, 696; ^{1}H NMR (600 MHz, $CDCl_3$) δ 1.56 (3H, d, *J* = 6.9 Hz, Me), 1.91 (1H, d, *J* = 4.8 Hz, OH), 4.75-4.79 (1H, m, CH), 7.43 (1H, t, *J* = 7.5 Hz, ArH), 7.56 (1H, d, *J* = 7.5 Hz, ArH), 7.59 (1H, d, *J* = 7.6 Hz, ArH), 7.69 (1H, brs, ArH); ^{13}C NMR (150 MHz, $CDCl_3$) δ 24.1 (q), 58.6 (d), 82.4 (s), 92.5 (s), 123/5 (d), 123.6 (q, *J* = 272.3 Hz), 124.8 (d, *J* = 3.6 Hz), 128.4 (d, *J* = 3.6 Hz), 128.8 (d), 130.8 (q, *J* = 32.4 Hz), 134.7 (d); EIMS m/z 214 (M$^+$); high resolution mass calcd for $C_{11}H_9F_3O$: 214.0606, found m/z 214.0590. Anal. Calcd for $C_{11}H_9F_3O$(+1/2H_2O): C, 59.20; H, 4.52. Found: C, 59.43; H, 4.28.

Preparation of ethyl 4-(3-hydroxyoct-1-yn-1-yl)benzoate (**1x**).

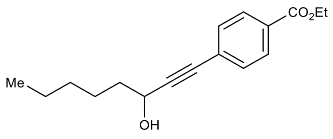

To a THF (6.60 mL) and triethylamine (6.60 mL) solution of ethyl 4-iodobenzoate (728 mg, 2.64 mmol) was added copper(I) iodide (25.0 mg, 0.198 mmol), $PdCl_2(PPh_3)_2$ (37.0 mg, 0.0528 mmol), oct-1-yn-3-ol (500 mg, 3.96 mmol) at room temperature for 24 h. The reaction mixture was filtered through a pad of celite with chloroform and the solvent was removed under reduced pressure. The residue was purified by column chromatography on silica gel eluting with AcOEt-*n*-hexane (1:20 to 1:10) to give ethyl 4-(3-hydroxyoct-1-yn-1-yl)benzoate (**1x**) (232 mg, 32%) as a brown oil.

IR (KBr, cm^{-1}) ν 3423, 2956, 2931, 2859, 1718, 1606, 1466, 1406, 1367, 1308, 1275, 1175, 1107, 1021, 859, 770, 697; ^{1}H NMR (600 MHz, $CDCl_3$) δ 0.91 (3H, t, *J* = 6.9 Hz, CH_3), 1.34-1.36 (4H, m, CH_2), 1.39 (3H, t, *J* = 6.9 Hz, CH_3), 1.50-1.54 (2H, m , CH_2), 1.79-1.83 (2H, m, CH_2), 1.97 (1H, brs, OH), 4.38 (2H, q, *J* = 6.9 Hz, OCH_2), 4.61 (1H, q, *J* = 6.2 Hz, CH_2), 7.48 (2H, d, *J* = 8.2 Hz, ArH), 7.98 (2H, d, *J* = 8.9 Hz, ArH); ^{13}C NMR (150 MHz, $CDCl_3$) δ 14.0 (q), 14.3 (q), 22.5 (t), 24.9 (t), 31.4 (t), 37.7 (t), 61.2 (t), 63.0 (d), 84.1 (s), 93.1 (s), 127.2 (s), 129.4 (d×2), 130.0 (s), 131.5 (d×2), 166.0 (s); EIMS m/z 274 (M$^+$), 245 (M$^+$ -Et), 229 (M$^+$ -OEt), 203 (M$^+$ -C_5H_{11}); high resolution mass calcd for $C_{17}H_{22}O_3$: 274.1569, found m/z 274.1576.

2-(3-hydroxybut-1-yn-1-yl)benzonitrile (**1y**).

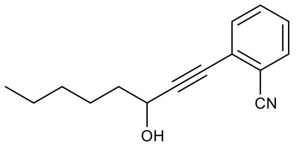

To a DMF (5.00 mL) and triethylamine (7.29 g, 72.1 mmol) solution of 1-octyn-3-ol (1.26 g, 10.0 mmol) and 2-iodobenzonitrile (2.29 g, 10.0 mmol) was added copper(I) iodide (0.38 g, 2.00 mmol), Pd $(PPh_3)_4$ (1.16 g, 1.00 mmol) at room temperature for 0.5 h. The reaction mixture was poured into water (50.0 mL). The organic layer was separated and the aqueous layer was extracted with AcOEt. The combined organic layer was dried over $MgSO_4$. The solvent was removed under reduced pressure The residue was purified by column chromatography on silica gel eluting with AcOEt-*n*-hexane (1:20 to 1:10) to give 4-(3-hydroxybut-1-yn-1-yl)benzonitrile (**1y**) (2.48 g, quant) as an orange oil.

IR (KBr, cm^{-1}) ν 3420, 2931, 113, 1594, 1482, 1445, 1380, 1338, 1276, 1123, 1025, 956, 909, 764; ^{1}H NMR (600 MHz, $CDCl_3$) δ 0.90 (3H, t, *J* = 7.4 Hz, Me), 1.34-1.36 (4H, m, CH_2), 1.54-1.57 (2H, m, CH_2), 1.83-1.87 (2H, m, CH_2), 2.49 (1H, brs, OH), 4.67 (1H, t, *J* = 6.9 Hz, CH), 7.38-7.42 (1H, m, ArH), 7.53 (2H, d, *J* = 4.0 Hz, ArH), 7.63 (1H, d, *J* = 8.0 Hz, ArH); ^{13}C NMR (150 MHz, $CDCl_3$) δ 14.0 (q), 22.5 (d), 24.7 (t), 31.4 (t), 37.5 (t), 62.8 (d), 80.8 (s), 97.1 (s), 115.3 (s), 117.5 (s), 126.6 (s), 128.3 (d), 132,3 (dx2), 132.5 (d); EIMS m/z 227 (M$^+$); high resolution mass calcd for $C_{15}H_{17}NO$: 227.1311, found m/z 171.0696.

Preparation of 4-(3-hydroxybut-1-yn-1-yl)benzonitrile (**1z**).

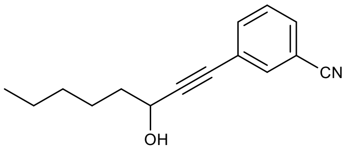

To a DMF (5.00 mL) and triethylamine (7.29 g, 72.1 mmol) solution of 1-octyn-3-ol (1.26 g, 10.0 mmol) and 3-iodobenzonitrile (2.72 g, 10.0 mmol) was added copper(I) iodide (0.38 g, 2.00 mmol), $PdCl_2(PPh_3)_2$ (0.702 g, 1.00 mmol) at room temperature for 0.5 h. The reaction mixture was poured into water (50.0 mL). The organic layer was separated and the aqueous layer was extracted with AcOEt. The combined organic layer was dried over $MgSO_4$. The solvent was removed under reduced pressure The residue was purified by column chromatography on silica gel eluting with AcOEt-*n*-hexane (1:20 to 1:10) to give 4-(3-hydroxybut-1-yn-1-yl)benzonitrile (**1z**) (2.52 g, quant) as a pale yellow oil.

IR (KBr, cm^{-1}) ν 3408. 2955, 2931, 2860, 2233, 1710, 1479, 1030, 800, 684; ^{1}H NMR (600 MHz, $CDCl_3$) δ 0.83 (3H, t, *J* = 6.9 Hz, Me), 1.30-1.33 (4H, m, CH_2), 1.45-1.53 (2H, m, CH_2), 1.74-1.81 (2H, m, CH_2), 2.42 (1H, brs, OH), 4.58 (1H, brs, CH), 7.39 (1H, t, *J* = 7.5 Hz, ArH), 7.55 (1H, dt, *J* = 1.1 and 7.4 Hz, ArH), 7.60 (1H, dt, *J* = 1.1 and 7.4 Hz, ArH), 7.65 (1H, brs, ArH); ^{13}C NMR (150 MHz, $CDCl_3$) δ 13.9 (q), 22.5 (t), 24.8 (t), 31.3 (t), 37.5 (t), 62.7 (d), 82.2 (s), 92.9 (s), 112.6 (s), 117.9 (s), 124.3 (s), 129.1 (d), 131.4 (d), 134.9 (d), 135.7 (d); EIMS m/z 227 (M$^+$), 209 (M$^+$-H_2O); high resolution mass calcd for $C_{15}H_{17}NO$: 227.1310, found m/z 227.1303. Anal. Calcd for $C_{15}H_{17}NO$ (+1/4H_2O): C, 77.72; H, 7.61; N, 6.04. Found: C, 77.78; H, 7.50; N, 6.02.

Preparation of 4-(3-hydroxybut-1-yn-1-yl)benzonitrile (**1α**).

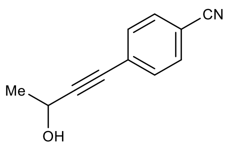

To a DMF (5.00 mL) and triethylamine (7.29 g, 72.1 mmol) solution of 1-butyn-3-ol (700 mg, 10.0 mmol) and 4-iodobenzonitrile (2.72 g, 10.0 mmol) was added copper(I) iodide (0.38 g, 2.00 mmol), $PdCl_2(PPh_3)_2$ (0.702 g, 1.00 mmol) at room temperature for 0.5 h. The reaction mixture was poured into water (50.0 mL). The organic layer was separated and the aqueous layer was extracted with AcOEt. The combined organic layer was dried over MgSO4. The solvent was removed under reduced pressure The residue was purified by column chromatography on silica gel eluting with AcOEt-*n*-hexane (1:20 to 1:10) to give 4-(3-hydroxybut-1-yn-1-yl)benzonitrile (**1α**) (1.33 g, 78%) as pale yellow powders.

mp 38-39 ºC, IR (KBr, cm^{-1}) ν 3421 (OH), 2983, 2930, 2229, 1604, 1501, 1406, 1330, 1259, 1178, 1105, 1035, 934, 841, 558; ^1H NMR (600 MHz, $CDCl_3$) δ 1.57 (3H, d, *J* = 6.3 Hz, Me), 2.44 (1H, d, *J* = 4.6 Hz, OH), 4.76-4.81 (1H, m, CH), 7.49 (2H, d, *J* = 8.6 Hz, ArH), 7.58 (2H, d, *J* = 8.6 Hz, ArH); ^{13}C NMR (150 MHz, $CDCl_3$) δ 24.0 (q), 58.6 (d), 82.2 (s), 95.4 (s), 111.55 (s), 118.3 (s), 127.5 (s), 131.9 (dx2), 132.1 (dx2); EIMS m/z 171 (M^+); high resolution mass calcd for $C_{11}H_9NO$: 171.0684, found m/z 171.0705. Anal. Calcd for $C_{11}H_9NO(+1/6H_2O)$: C, 75.84; H, 5.40; N, 8.04. Found: C, 75.96; H, 5.16; N, 7.77.

Preparation of 4-(4-nitrophenyl)but-3-yn-2-ol (**1β**).

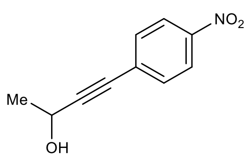

To a DMF (5.00 mL) and triethylamine (7.29 g, 72.1 mmol) solution of 1-butyn-3-ol (0.700 g, 10.0 mmol) and 4-nitroiodobenzene (2.49 g, 10.0 mmol) was added copper(I) iodide (0.38 g, 2.00 mmol), $PdCl_2(PPh_3)_2$ (0.702 g, 1.00 mmol) at room temperature for 0.5 h. The reaction mixture was poured into water (50.0 mL). The organic layer was separated and the aqueous layer was extracted with AcOEt. The combined organic layer was dried over $MgSO_4$. The solvent was removed under reduced pressure The residue was purified by column chromatography on silica gel eluting with AcOEt-*n*-hexane (1:10 to 1:5) to give 4-(4-nitrophenyl)but-3-yn-2-ol (**1β**) (1.49 g, 78%) as pale yellow needles.

mp 91-92 ºC, IR (KBr, cm^{-1}) ν 3415, 2984, 2854, 2231, 1710, 1596, 1521, 1346, 1109, 1038, 934, 858, 843, 751; ^1H NMR (600 MHz, $CDCl_3$) δ 1.58 (3H, d, *J* = 6.9 Hz, Me), 2.34 (1H, d, *J* = 4.5 Hz, OH), 4.79-4.83 (1H, m, CH), 7.55 (2H, d, *J* = 9.2 Hz, ArH), 8.17 (2H, d, *J* = 9.2 Hz, ArH); ^{13}C NMR (150 MHz, $CDCl_3$) δ 24.0 (q), 58.7 (d), 82.1 (s), 96.2 (s), 123.5 (dx2), 129.5 (s), 132.3 (dx2), 147.1 (s); EIMS m/z 191 (M+); high resolution mass calcd for $C_{10}H_9NO_3$: 191.0584, found m/z 191.0577. Anal. Calcd for $C_{10}H_9NO_3$: C, 62.82; H, 4.75; N, 7.33. Found: C, 62.65; H, 4.57; N, 7.29.

Preparation of 1-(2-methyl-5-nitrophenyl)oct-1-yn-3-ol (**1γ**).

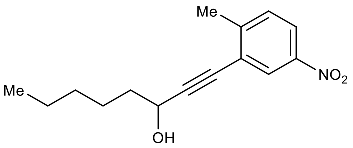

To a DMF (5.00 mL) and triethylamine (7.29 g, 72.1 mmol) solution of 1-octyn-3-ol (1.26 g, 10.0 mmol) and 2-iodo-1-methylnitrobenzene (2.63 g, 10.0 mmol) was added copper(I) iodide (0.38 g, 2.00 mmol), $PdCl_2(PPh_3)_2$ (0.702 g, 1.00 mmol) at room temperature for 0.5 h. The reaction mixture was poured into water (50.0 mL). The organic layer was separated and the aqueous layer was extracted with AcOEt. The combined organic layer was dried over $MgSO_4$. The solvent was removed under reduced pressure The residue was purified by column chromatography on silica gel eluting with AcOEt-*n*-hexane (1:20 to 1:10) to give 1-(2-methyl-5-nitrophenyl)oct-1-yn-3-ol (**1γ**) (2.28 g, 87%) as a pale orange oil.

IR (KBr, cm^{-1}) ν 3389, 2930, 2860, 1577, 1523, 1467, 1349, 1029, 903, 832, 798; ^1H NMR (600 MHz, $CDCl_3$) δ 0.91 (3H, t, *J* = 7.5 Hz, Me), 1.34-1.37 (4H, m, CH_2), 1.53-1.57 (2H, m, CH_2), 1.79-1.89 (2H, m, CH_2), 2.50 (3H, s, Me), 2.63 (1H, brs, OH), 4.68 (1H, t, *J* = 6.9 Hz, CH), 7.33 (1H, d, *J* = 8.6 Hz, ArH), 8.02 (1H, d, *J* = 8.0 Hz, ArH), 8.19 (1H, brs, ArH); ^{13}C NMR (150 MHz, $CDCl_3$) δ 13.9 (q), 20.8 (q), 22.5 (t), 24.8 (t), 31.3 (t), 37.6 (t), 62.8 (d), 81.2 (s), 96.8 (s), 122.8 (d), 123.9 (s), 126.6 (d), 130.1 (d), 145.8 (s), 147.6 (s); EIMS m/z 261 (M^+), 244 (M^+-Me); high resolutions mass calcd for $C_{15}H_{19}NO_3$: 261.1365, found m/z 261.1351. Anal. Calcd for $C_{15}H_{19}NO_3(+1/13H_2O)$: C, 68.60; H, 7.35. Found: C, 68.42: H, 7.06.

Preparation of dodec-5-yn-4-ol (**1δ**).

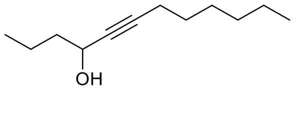

To a THF (10.0 mL) solution of 1-octyne (1.00 g, 9.07 mmol) was added 2.6 M n-BuLi (2.91 mL, 7.56 mmol) at -76 ºC under an Ar atmosphere. After 10 min stirring the mixture, a THF (5.00 mL) solution of n-butyraldehyde (0.545 g, 7.56 mmol) was added dropwise to the mixture. After 10 min stirring the mixture, a NH_4Claq (10.0 mL) was added to the mixture and poured into water (100 mL). The organic layer was separated and the aqueous layer was extracted with AcOEt. The organic layer was washed with NH_4Claq (50.0 mL×2) and dried over $MgSO_4$. The solvent was removed under reduced pressure. The residue was purified by column chromatography on silica gel eluting with *n*-hexane to AcOEt-*n*-hexane (1:20) to give dodec-5-yn-4-ol (**1δ**) (0.997 g, 72%) as a colorless oil.

IR (KBr) ν 3389, 2958, 2923, 2872, 2860, 1467, 1379, 1331, 1149, 1103, 1065, 1025, 758; ^1H NMR (600 MHz, $CDCl_3$) δ 0.89 (3H, t, *J* = 7.5 Hz, CH_3), 0.94 (3H, t, *J* = 7.6 Hz, CH_3), 1.24-1.33 (4H, m, CH_2), 1.35-1.40 (2H, m, CH_2), 1.44-1.52 (4H, m, CH_2), 1.60-1.70 (2H, m, CH_2), 1.96 (1H, brs, OH), 2.20 (2H, t, *J* = 6.9 Hz, CH_2), 4.36 (1H, s, CH); ^{13}C NMR (150 MHz, $CDCl_3$) δ 13.7 (q), 14.0 (q), 18.4 (t), 18.6 (t), 22.5 (t), 28.5 (t), 28.6 (t), 31.3 (t), 40.3 (t), 62.4 (d), 81.3 (s), 85.4 (s); EIMS m/z 183 (M^+).

Anal. Calcd for $C_{12}H_{22}O$: C; 76.02, H; 11.69. Found: C; 76.04, H; 11.69.

Liao, R. An, H. Li, Y. Xu, J.-J. Wu, X. Zhao, Angew. Chem., Int. Ed. 2020, 59, 11010-11019.

tetracos-13-yn-12-ol (**1ε**).

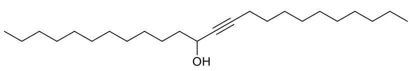

To a THF (10.0 mL) solution of 1-dodecyne (1.00 g, 6.01 mmol) was added 2.6 M *n*-BuLi (1.93 mL, 5.01 mmol) at -76 °C under an Ar atmosphere. After 10 min stirring the mixture, a THF (5.00 mL) solution of dodecanal (0.924 g, 5.01 mmol) was added dropwise to the mixture. After 10 min stirring the mixture, a NH_4Claq (10.0 mL) was added to the mixture and poured into water (100 mL). The organic layer was separated and the aqueous layer was extracted with AcOEt. The organic layer was washed with NH_4Claq (50.0 mL×2) and dried over $MgSO_4$. The solvent was removed under reduced pressure. The residue was purified by column chromatography on silica gel eluting with *n*-hexane to AcOEt-*n*-hexane (1:20) to give tetracos-13-yn-12-ol (**1ε**) (1.42 g, 81%) as a white solid (mp below 30 °C).

IR (KBr) ν 3389, 2955, 2925, 2853, 1467, 1378, 1145, 1033, 722;

^{1}H NMR (600 MHz, $CDCl_3$) δ 0.88 (3H, t, *J* = 7.6 Hz, CH_3), 1.26 (30H, brs, CH_2), 1.37-1.38 (1H, m, CH_2), 1.42-1.43 (1H, m, CH_2), 1.50 (2H, quin, *J* = 7.6 Hz, CH_2), 1.61-1.70 (2H, m, CH_2), 1.77 (1H, brs, OH), 2.20 (2H, td, *J* = 2.1 and 6.9 Hz, CH_2), 4.34 (1H, s, CH); ^{13}C NMR (150 MHz, $CDCl_3$) δ 14.1 (q×2), 18.7 (t), 22.7 (t×2), 25.2 (t), 28.7 (t), 28.8 (t), 29.1 (t), 29.30 (t), 29.32 (t), 29.35 (t), 29.5 (t), 29.58 (t×3), 29.63 (t), 29.7 (t), 31.9 (t×2), 38.2 (t), 62.8 (d), 81.3 (s), 85.5 (s); EIMS m/z 350 (M^+); high resolution mass calcd for $C_{24}H_{46}O$: 350.35487. Found: 350.3528.

(R.Toubiana, J. Asselineau Ann. Chim. 1962, 7, 593-642.)

Preparation of 1-(phenylethynyl)cyclopentane-1-ol (**1ζ**).

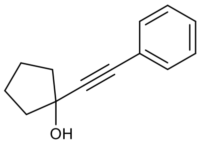

To a THF (18.0 mL) solution of ethynylbenzene (2.00 g, 20.0 mmol) was added *n*-butyllithium (6.7 mL, 18.0 mmol) at -78 °C under an Ar atmosphere. After 5 min stirring, cyclopentanone (1.51 g, 18.0 mmol) in THF (20.00 mL) was added to the mixture. The whole was stirred for 5 min and then poured into water (50 mL). The organic layer was separated and the aqueous layer was extracted with AcOEt. The combined organic layer was dried over $MgSO_4$. The solvent was removed under reduced pressure. The titled compound was precipitated from *n*-hexane to give 1-(phenylethynyl)cyclopentane-1-ol (**1ζ**) (1.05 g, 31%) as white powders (mp 34-35 °C, chloroform-*n*-hexane).

IR (KBr, cm^{-1}) ν 3399, 2969, 2824, 2853, 1707, 1489, 1443, 1364, 1195, 1070, 995, 757, 692; ^{1}H NMR (600 MHz, $CDCl_3$) δ 1.76 (2H, m, CH_2), 1.86-1.88 (2H, m, CH_2), 2.03-2.07 (4H, m, CH_2), 7.28-7.29 (3H, m, ArH), 7.41-7.43 (2H, m, ArH); ^{13}C NMR (150 MHz, $CDCl_3$) δ 23.5 (d×2), 42.5 (d×2), 74.9 (s), 83.0 (s), 92.9 (s), 122.8 (s), 128.1 (d), 128.2 (d×2), 131.6 (d×2); EIMS m/z 185 (M^+), 157 (M^+-C_2H_4), 129 (M^+-C_4H_8). Anal. Calcd for $C_{13}H_{14}O$: C, 83.83; H, 7.78. Found: C, 83.65; H, 7.64.

(H. Qiu, H. D. Srinivas, P. Y. Zavalij, M. P. Doyle J. Am. Chem. Soc. 2016, 138, 1808-1811.)

Preparation of 1-(phenylethynyl)cyclohexan-1-ol (**1η**).

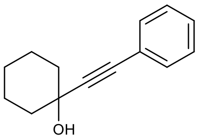

To a THF (18.0 mL) solution of ethynylbenzene (2.00 g, 20.0 mmol) was added *n*-butyllithium (6.7 mL, 18.0 mmol) at -78 °C under an Ar atmosphere. After 5 min stirring, cyclohexanone (1.77 g, 18.0 mmol) in THF (10.00 mL) was added to the mixture. The whole was stirred for 5 min and then poured into water (50 mL). The organic layer was separated and the aqueous layer was extracted with AcOEt. The combined organic layer was dried over $MgSO_4$. The solvent was removed under reduced pressure. The titled compound was precipitated from *n*-hexane to give 1-(phenylethynyl)cyclopentane-1-ol (**1η**) (3.35 g, 84%) as white powders (mp 53-55 °C, chloroform-*n*-hexane).

IR (KBr, cm^{-1}) ν 3388, 2966, 2872, 2223, 1705, 1599, 1489, 1443, 1363, 1195, 1070, 995, 914, 757, 691; ^{1}H NMR (600 MHz, $CDCl_3$) δ 1.24-1.30 (1H, m, CH), 1.55-1.67 (5H, m, CH), 1.71-1.75 (2H, m, CH_2), 2.00-2.02 (2H, m, CH_2), 2.31 (1H, s, OH), 7.28-7.31 (3H, m, ArH), 7.41-7.44 (2H, m, ArH); ^{13}C NMR (150 MHz, $CDCl_3$) δ 23.4 (t), 25.2 (tx2), 40.0 (tx2), 69.1 (s), 84.3 (s), 92.8 (s), 122.9 (s), 128.1 (d), 128.2 (dx2), 131.6 (dx2); EIMS m/z 200 (M^+). Anal. Calcd for $C_{14}H_{16}O$: C, 83.96; H, 8.05. Found: C, 84.03; H, 7.97.

(K.-W. Feng, Y.-L. Ban, P.-F. Yuan, W.-L. Lei, G. Liu, R. Fang Org. Lett. 3029, 21, 3131-3135.)

Preparation of 1-(phenylethynyl)cyclooctane-1-ol (**1θ**).

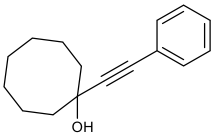

To a THF (18.0 mL) solution of ethynylbenzene (2.27 g, 18.0 mmol) was added *n*-butyllithium (6.7 mL, 18.0 mmol) at -78 °C under an Ar atmosphere. After 5 min stirring, cyclooctanone (2.04 mg, 20.0 mmol) in THF (20.0 mL) was added to the mixture. The whole was stirred for 5 min and then poured into water (50 mL). The organic layer was separated and the aqueous layer was extracted with AcOEt. The combined organic layer was dried over $MgSO_4$. The solvent was removed under reduced pressure. The residue was purified by column chromatography on silica gel eluting with AcOEt-*n*-hexane (1:20) to give 1-(phenylethynyl)cyclooctane-1-ol (**1θ**)(1.26 g, 38%) as a white crystal (mp 71-72 °C, from chloroform-*n*-hexane).

IR (KBr, cm^{-1}) ν 3274, 2924, 2852, 1490, 1443, 1066, 756, 692; ^{1}H NMR (600 MHz, $CDCl_3$) δ 1.51 (3H, brs, CH_2), 1.63 (3H, brs, CH_2), 1.70

(4H, brs, CH_2), 1.98-2.08 (4H, m, CH_2), 2.17-2.17 (1H, m, OH), 7.27-7.28 (3H, m, ArH), 7.41-7.42 (2H, m, ArH); ^{13}C NMR (150 MHz, $CDCl_3$) δ 22.1 (t×2), 24.4 (t), 27.9 (t×2), 38.3 (t×2), 71.7 (s), 83.2 (s), 93.8 (s), 122.9 (s), 128.0 (d), 128.1 (d×2), 131.6 (d×2); EIMS m/z 227 (M^+), 210 (M^+-OH), 199 (M^+-C_2H_4), 185 (M^+-C_3H_6), 171 (M^+-C_4H_8), 157 (M^+-C_5H_{10}), 129 (M^+-C_7H_{14}); high resolution mass calcd for $C_{16}H_{20}O$: 228.1514, found m/z 228.1531.

Preparation of 3-ethyl-1-phenylpent-1-yn-3-ol (**1ι**).

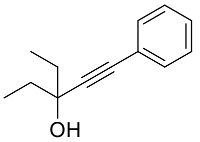

To a THF (18.0 mL) solution of ethynylbenzene (2.27 g, 18.0 mmol) was added *n*-butyllithium (6.7 mL, 18.0 mmol) at -78 °C under an Ar atmosphere. After 5 min stirring, 3-pentanone (1.72 g, 20.0 mmol) in THF (20.0 mL) was added to the mixture. The whole was stirred for 5 min and then poured into water (50 mL). The organic layer was separated and the aqueous layer was extracted with AcOEt. The combined organic layer was dried over $MgSO_4$. The solvent was removed under reduced pressure. The residue was purified by column chromatography on silica gel eluting with AcOEt-*n*-hexane (1:20) to give 3-ethyl-1-phenylpent-1-yn-3-ol (**1ι**)(2.13 g, 63%) as a colorless oil.

IR (KBr, cm^{-1}) ν 3407, 2970, 2938, 2879, 2227, 1599, 1490, 1460, 444, 1327, 1143, 961, 756, 691 ^{1}H NMR (600 MHz, $CDCl_3$) δ 1.10 (6H, t, J = 7.6 Hz, Mex2), 1.72-1.79 (4H, m, CH_2x2), 7.25-7.33 (3H, m, ArH), 7.40-7.45 (2H, m, ArH); ^{13}C NMR (150 MHz, $CDCl_3$) δ 8.6 (qx2), 34.4 (tx2), 72.5 (s), 84.4 (s), 91.7 (s), 122.9 (s), 128.1 (d), 128.2 (dx2), 131.6 (dx2); EIMS m/z 188 (M^+).

(D. A. Engel, G. B. Dudley Org Lett. 2006, 8, 4027-4029.)

Preparation of 1-(2-nitrophenyl)hept-2-yn-1-ol (**1κ**).

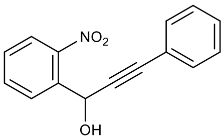

To a THF (15.0 mL) solution of ethynylbenzene (1.33 g, 13.0 mmol) was added *n*-butyllithium (4.50 mL, 12.0 mmol) at -78 °C under an Ar atmosphere. After 5 min stirring, 2-nitrobenzaldehyde (1.51 g, 10.0 mmol) in THF (8.00 mL) was added to the mixture. The whole was stirred for 5 min and then poured into water (50 mL). The organic layer was separated and the aqueous layer was extracted with AcOEt. The combined organic layer was dried over $MgSO_4$. The solvent was removed under reduced pressure. The residue was purified by column chromatography on silica gel eluting with AcOEt-*n*-hexane (1:10) to give 1-(2-nitrophenyl)hept-2-yn-1-ol (**1κ**) (1.23 g, 49%) as an orange oil.

IR (KBr, cm^{-1}) ν 3389, 3064, 2924, 2860, 2234, 1703, 1526, 1490, 1444, 1349, 1182, 962, 857, 787, 758, 723, 691; ^{1}H NMR (600 MHz, $CDCl_3$) δ 3.55 (1H, brs, OH), 6.21 (1H, s, CH), 7.26-7.31 (3H, m, ArH), 7.42-7.43 (2H, m, ArH), 7.45-7.49 (1H, m, ArH), 7.64-7.67 (1H, m, ArH), 7.94 (1H, d, J = 8.0 Hz, ArH), 7.99 (1H, dd, J = 1.2 and 8.0 Hz, ArH); ^{13}C NMR (150 MHz, $CDCl_3$) δ 61.7 (d), 86.6 (s), 86.7 (s), 121.8 (s), 124.9 (d), 128.2 (dx2), 128.7 (d), 129.2 (d), 129.4 (d), 131.7 (dx2), 133.7 (d), 135.4 (s), 148.0 (s); EIMS m/z 253 (M^+), 236 (M^+-OH). Anal. Calcd for $C_{15}H_{11}NO_3$: C, 71.14; H, 4.38; N, 5.53. Found: C, 70.95; H, 4.45; N, 5.51.

Preparation of 1-(4-nitrophenyl)-3-phenylprop-2-yn-1-ol (**1λ**).

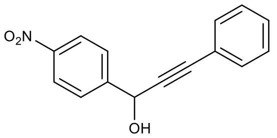

To a THF (12.0 mL) solution of ethynylbenzene (800 mg, 7.90 mmol) was added *n*-butyllithium (2.50 mL, 6.60 mmol) at 0 °C under an Ar atmosphere. After 5 min stirring, 4-nitrobenzaldehyde (1.00 g, 6.60 mmol) in THF (7.00 mL) was added to the mixture. The whole was stirred for 5 min and then poured into water (50 mL). The organic layer was separated and the aqueous layer was extracted with AcOEt. The combined organic layer was dried over $MgSO_4$. The solvent was removed under reduced pressure. The residue was purified by column chromatography on silica gel eluting with AcOEt-*n*-hexane (1:20) to give 1-(4-nitrophenyl)-3-phenylprop-2-yn-1-ol (**1λ**) (1.05 g, 63%) as an orange crystal (mp 105-107 °C from chloroform-*n*-hexane).

IR (KBr, cm^{-1}) ν 3491, 1709, 1604, 1598, 1518, 1488, 1345, 1184, 1108, 1058, 858, 804, 754, 729, 689; ^{1}H NMR (600 MHz, $CDCl_3$) δ 2.49 (1H, d, J = 6.2 Hz, OH), 5.80 (1H, d, J = 5.5 Hz, CH), 7.33-7.37 (3H, m, ArH), 7.46-7.47 (2H, m, ArH), 7.80 (2H, d, J = 9.0 Hz, ArH), 8.27 (2H, d, J = 9.0 Hz, ArH); ^{13}C NMR (150 MHz, $CDCl_3$) δ 64.1 (d), 87.4 (s), 87.7 (s), 121.7 (s), 123.8 (d×2), 127.4 (d×2), 128.4 (d×2), 129.1 (d), 131.7 (d×2), 147.4 (s), 147.8 (s); EIMS m/z 252 (M^+), 236 (M^+ -OH), 207 (M^+ -NO_2); high resolution mass calcd for $C_{15}H_{11}NO_3$: 253.0739, found m/z 253.0792. Anal. Calcd for $C_{15}H_{11}NO_3$(+ 1 / 10 H_2O): C, 70.64; H, 4.43; N, 5.49. Found: C, 70.35; H, 4.36; N, 5.44.

(N. Sakai, R. Kanada, M. Hirasawa, T. Konakahara Tetrahedron 2005, 61, 9298-9304.)

Preparation of 1-(3-nitrophenyl)-3-phenylprop-2-yn-1-ol (**1μ**).

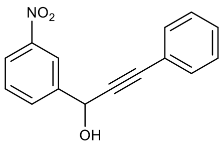

To a THF (12.0 mL) solution of ethynylbenzene (800 mg, 7.90 mmol) was added *n*-butyllithium (2.50 mL, 6.60 mmol) at 0 °C under an Ar atmosphere. After 5 min stirring, 3-nitrobenzaldehyde (1.00 g, 6.60 mmol) in THF (7.00 mL) was added to the mixture. The whole was stirred for 5 min and then poured into water (50 mL). The organic layer was separated and the aqueous layer was extracted with AcOEt. The combined organic layer was dried over $MgSO_4$. The solvent was removed under reduced pressure. The residue was purified by column chromatography on silica gel eluting with AcOEt-*n*-hexane (1:20) to give 1-(4-nitrophenyl)-3-phenylprop-2-yn-1-ol (**1μ**) (0.94 g, 56%) as an orange crystal (mp 105-107 °C from chloroform-*n*-hexane).

IR (KBr, cm^{-1}) ν 3367, 3077, 2925, 2230, 1702, 1530, 1490, 1442, 1350, 1037, 974, 917, 803, 758, 726, 692; ^{1}H NMR (600 MHz, $CDCl_3$) δ 2.68 (1H, brs, OH), 5.80 (1H, J = 4.8 Hz, CH), 7.32-7.37 (3H, m, ArH), 7.47 (2H, d, J = 6.2 Hz, ArH), 7.56-7.58 (1H, m, ArH), 7.95 (1H, d, J

= 7.6 Hz, ArH), 8.18 (1H, d, *J* = 7.5 Hz, ArH), 8.49 (1H, brs, ArH); ^{13}C NMR (150 MHz, $CDCl_3$) δ 63.9 (d), 87.4 (s), 87.6 (s), 121.7 (d), 123.2 (d), 128.4 (dx2), 129.0 (d), 129.6 (d), 131.8 (dx2), 132.7 (d), 142.6 (s), 148.4 (s) one singlet is missing; EIMS m/z 253 (M^+), 236 (M^+-OH); high resolution mass calcd for $C_{15}H_{11}NO_3$: 253.0739, found m/z 253.0720. Anal. Calcd for $C_{15}H_{11}NO_3$: C, 71.14; H, 4.38; N, 5.53. Found: C, 70.91; H, 4.57; N, 5.66.

3-phenyl-1-(4-(trifluoromethyl)phenyl)prop-2-yn-1-ol (**1**ν).

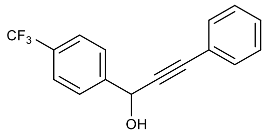

IR (KBr, cm^{-1}) ν 3395, 2925, 2852, 2200, 1705, 1620, 1491, 1415, 1327, 1166, 1126, 1067, 1018, 849, 758, 691; ^{1}H NMR (600 MHz, $CDCl_3$) δ 2.36 (1H, d, *J* = 5.7 Hz, OH), 5.76 (1H, d, *J* = 5.7 Hz, CH), 7.32-7.37 (3H, m, ArH), 7.47 (2H, dd, *J* = 1.7 and 7.5 Hz, ArH), 7.67 (2H, d, *J* = 8.0 Hz, ArH), 7.74 (2H, d, *J* = 7.5 Hz, ArH); ^{13}C NMR (150 MHz, $CDCl_3$) δ 64.3 (d), 87.2 (s), 87.9 (s), 121.9 (s), 124.0 (q, *J* = 272.3 Hz), 125.5 (qx2, *J* = 3.6 Hz), 126.9 (dx2), 128.4 (dx2), 128.9 (d), 130.4 (q, *J* = 32.4 Hz), 131.7 (dx2), 144.3 (s); EIMS m/z 276 (M^+), 259 (M^+-OH); high resolution mass calcd for $C_{16}H_{11}F_3O$: 276.0762, found m/z 276.0684. Anal. Calcd for $C_{16}H_{11}F_3O$: C, 69.56; H, 4.01. Found: C, 69.47; H, 3.79.

(Pennell, M. N.; Turner, P. G.; Sheppard, T. D. *Chem. Eur. J.* **2012**, *18*, 4748-4758.)

N-(4-(1-hydroxy-3-phenylprop-2-yn-1-yl)phenyl)acetamide (**1**ξ).

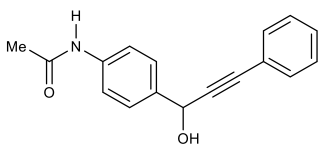

To a THF (18.0 mL) solution of ethynylbenzene (1.79 g, 17.5 mmol) was added *n*-butyllithium (5.5 mL, 15.4 mmol) at 0 °C under an Ar atmosphere. After 5 min stirring, *N*-(4-formylphenyl)acetamide (1.14 g, 7.00 mmol) in THF (7.00 mL) was added to the mixture. The whole was stirred for 5 min and then poured into water (50 mL). The organic layer was separated and the aqueous layer was extracted with AcOEt. The combined organic layer was dried over $MgSO_4$. The solvent was removed under reduced pressure. The residue was purified by column chromatography on silica gel eluting with AcOEt-*n*-hexane (1:2) to give *N*-(4-(1-hydroxy-3-phenylprop-2-yn-1-yl)phenyl)acetamide (**1**ξ) (1.10 g, 59%) as a peach crystal (mp 135-137 °C from chloroform-*n*-hexane).
IR (KBr, cm^{-1}) ν 3311, 1705, 1671, 1604, 1540, 1515, 1490, 1412, 1370, 1318, 1264, 1033, 1018, 965, 843, 759, 692; ^{1}H NMR (600 MHz, DMSO) δ 2.03 (3H, s, CH_3), 5.54 (1H, d, *J* = 6.1 Hz, CH), 6.07 (1H, d, *J* = 5.5 Hz, OH), 7.34-7.36 (3H, m, ArH), 7.42-7.44 (1H, m, ArH), 7.44 (2H, d, *J* = 7.2 Hz, ArH), 7.58 (2H, d, *J* = 8.3 Hz, ArH), 9.96 (1H, s, NH); ^{13}C NMR (150 MHz, DMSO) δ 24.0 (q), 62.6 (d), 84.3 (s), 91.5 (s), 118.8 (d×2), 122.3 (s), 126.9 (d×2), 128.6 (d), 128.7 (d×2), 131.3 (d×2), 136.6 (s), 138.8 (s), 168.3 (s); EIMS m/z not found; high resolution mass calcd for $C_{17}H_{15}NO_2$: 265.1103, found m/z 265.1099. Anal. Calcd for $C_{17}H_{15}NO_2$(+ 1 / 4 H_2O): C, 75.68; H, 5.79; N, 5.19. Found: C, 75.55; H, 5.68; N, 5.27.

(J.-F. Bai, K. Yasumoto, T. Kano, K. Maruoka Angew. Chem. Int. Ed. 2019, 58, 8898-8901.)

Preparation of 1-(4-nitrophenyl)hept-2-yn-1-ol (**1**ο).

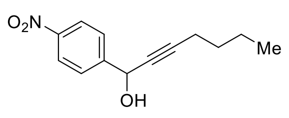

IR (KBr, cm^{-1}) ν 3313, 2958, 2933, 2225, 1703, 1607, 1523, 1348, 1136, 1108, 1012, 857, 810, 701; ^{1}H NMR (600 MHz, $CDCl_3$) δ 0.91 (3H, t, *J* = 7.5 Hz, ArH), 1.37-1.44 (2H, m, CH_2), 1.48-1.54 (2H, m, CH_2), 2.27 (1H, t, *J* = 7.4 Hz, CH), 2.84 (1H, brs, OH), 5.54 (1H, s, CH), 7.70 (2H, d, *J* = 7.4 Hz, ArH), 8.20 (2H, d, *J* = 7.4 Hz, ArH); ^{13}C NMR (150 MHz, $CDCl_3$) δ 13.4 (q), 18.3 (t), 21.9 (t), 30.4 (t), 63.6 (d), 78.8 (s), 88.7 (s), 123.6 (dx2), 127.2 (dx2), 147.5 (s), 148.1 (s); EIMS m/z 233 (M^+), 216 (M^+-OH), high resolution mass calcd for $C_{13}H_{15}NO_3$: 233.1052, found m/z 233.1064. Anal. Calcd for $C_{13}H_{15}NO_3$(+1/10H_2O): C, 66.42; H, 6.52; N, 6.00. Found: C, 66.45; H, 6.53; N, 5.94.

(S. Banerjee, S. B. Ambegave, R. D. Mule, B. Senthikumar, N. Patil Org. Lett. 2020, 22, 4792-4796.)

Preparation of methyl 4(1-hydroxyhept-2-yn-1-yl)benzene (**1**ρ).

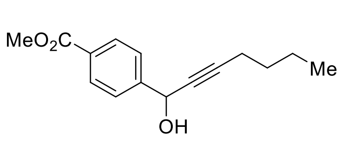

To a THF (18.0 mL) solution of ethynylbenzene (1.79 g, 17.5 mmol) was added *n*-butyllithium (5.5 mL, 15.4 mmol) at 0 °C under an Ar atmosphere. After 5 min stirring, methyl 4-formylbenzoate (1.64 g, 10.0 mmol) in THF (10.0 mL) was added to the mixture. The whole was stirred for 5 min and then poured into water (50 mL). The organic layer was separated and the aqueous layer was extracted with AcOEt. The combined organic layer was dried over $MgSO_4$. The solvent was removed under reduced pressure. The residue was purified by column chromatography on silica gel eluting with AcOEt-*n*-hexane (1:20 to 1:10) to give methyl 4(1-hydroxyhept-2-yn-1-yl)benzene (**1**ρ) (1.97 g, 80%) as a yellow oil.

IR (KBr, cm^{-1}) ν 3438, 2956, 2933, 1724, 1649, 1437, 1281, 1192, 1114, 1018, 870, 752; ^{1}H NMR (600 MHz, $CDCl_3$) δ 0.90(3H, t, *J* = 7.6 Hz, CH_3), 1.40-1.43 (2H, m, CH_2), 1.49-1.54 (2H, m, CH_2), 2.26 (2H, dt, *J* = 2.1 and 6.8 Hz, CH_2), 2.65 (1H, d, *J* = 5.5 Hz, OH), 3.91 (3H, s, OCH_3), 5.49 (1H, brd, *J* = 5.5 Hz, CH), 7.60 (2H, d, *J* = 8.2 Hz, ArH), 8.02 (2H, d, *J* = 8.2 Hz, ArH); ^{13}C NMR (150 MHz, $CDCl_3$) δ 13.5 (q), 18.4 (t), 21.9 (t), 30.5 (t), 52.1 (d), 64.2 (q), 79.4 (s), 88.1 (s), 126.4 (d×2), 129.7 (s), 129.8 (d×2), 146.1 (s), 166.9 (s); EIMS m/z 246 (M^+), 231 (M^+ -Me), 187 (M^+ -CO_2Me); high resolution mass calcd for $C_{15}H_{18}O_3$: 246.1256, found m/z 246.1264.

Anal. Calcd for $C_{15}H_{18}O_3$(+ 1 / 10 H_2O): C, 72.26; H, 7.37. Found: C, 72.66; H, 6.81.

(J.-C. Tseng, S.-L. Huang, C.-L. Lin, H.-C. Lin, B.-Y. Jin, C.-Y. Chen, J.-K. Yu, P.-T. Chou, T.-Y. Luh Org Lett. 2003, 5, 4381-4383.)

Screening for reaction conditions for the synthesis of 1,2,3-trimethoxy-4-(1-phenylpent-1-yn-3-yl)benzene (2a) (Table 1).

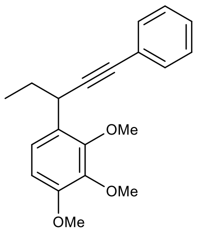

Entry 1: To a nitromethane (0.500 mL) solution of 1-phenylpent-1-yn-3-ol (**1a**) (25.0 mg, 0.156 mmol) and 1,2,3-trimethoxybenzene (78.7 mg, 0.468 mmol) were added ytterbium trifluoromethanesulfonate hydrate (19.4 mg, 0.0312 mmol). The reaction mixture was stirred at 100 °C for 10 min and poured into a saturated sodium hydrogencarbonate. The organic layer was separated and the aqueous layer was extracted with AcOEt. The combined organic layer was dried over $MgSO_4$ and the solvent was removed under reduced pressure. The residue was purified by preparative TLC on silica gel eluting with AcOEt-*n*-hexane (1:40) to give 1,2,3-trimethoxy-4-(1-phenylpent-1-yn-3-yl)benzene (**2a**) (15.5 mg, 37%) as a yellow oil.

Entry 2: To a nitromethane (0.500 mL) solution of 1-phenylpent-1-yn-3-ol (**1a**) (25.0 mg, 0.156 mmol), 1,2,3-trimethoxybenzene (78.7 mg, 0.468 mmol), and tetrabutylammonium hexafluorophosphate (12.1 mg, 0.0312 mmol) were added ytterbium trifluoromethanesulfonate hydrate (19.4 mg, 0.0312 mmol). The reaction mixture was stirred at 50 °C for 1 h and then the almost same procedure as entry 1 was performed. The residue was purified by preparative TLC on silica gel eluting with AcOEt-*n*-hexane (1:40) to give 1,2,3-trimethoxy-4-(1-phenylpent-1-yn-3-yl)benzene (**2a**) (27.2 mg, 56%) as a yellow oil.

Entry 3: To a nitromethane (0.500 mL) solution of 1-phenylpent-1-yn-3-ol (**1a**) (25.0 mg, 0.156 mmol), 1,2,3-trimethoxybenzene (78.7 mg, 0.468 mmol), and tetrabutylammonium hexafluorophosphate (12.1 mg, 0.0312 mmol) were added ytterbium(III) tris(trifluoromethanesulfonimide) (31.6 mg, 0.0312 mmol). The reaction mixture was stirred at 50 °C for 1 h and then the almost same procedure as entry 1 was performed. The residue was purified by preparative TLC on silica gel eluting with AcOEt-*n*-hexane (1:40) to give 1,2,3-trimethoxy-4-(1-phenylpent-1-yn-3-yl)benzene (**2a**) (16.3 mg, 34%) as a yellow oil and recover 1-phenylpent-1-yn-3-ol (**1a**) (7.5 mg, 30%).

Entry 4: To a nitromethane (0.500 mL) solution of 1-phenylpent-1-yn-3-ol (**1a**) (25.0 mg, 0.156 mmol), 1,2,3-trimethoxybenzene (78.7 mg, 0.468 mmol), and tetrabutylammonium hexafluorophosphate (12.1 mg, 0.0312 mmol) were added scandium trifluoromethanesulfonate (15.4 mg, 0.0312 mmol). The reaction mixture was stirred at 50 °C for 30 min and then the almost same procedure as entry 1 was performed. The residue was purified by preparative TLC on silica gel eluting with AcOEt-*n*-hexane (1:40) to give 1,2,3-trimethoxy-4-(1-phenylpent-1-yn-3-yl)benzene (**2a**) (27.2 mg, 56%) as a yellow oil.

Entry 5: To a nitromethane (0.500 mL) solution of 1-phenylpent-1-yn-3-ol (**1a**) (25.0 mg, 0.156 mmol) and 1,2,3-trimethoxybenzene (78.7 mg, 0.468 mmol) were added boron trifluoride diethyl ether complex (4.4 mg, 0.0312 mmol). The reaction mixture was stirred at 50 °C for 30 min and then the almost same procedure as entry 1 was performed. The residue was purified by preparative TLC on silica gel eluting with AcOEt-*n*-hexane (1:40) to give 1,2,3-trimethoxy-4-(1-phenylpent-1-yn-3-yl)benzene (**2a**) (28.4 mg, 58%) as a yellow oil and (oxybis(pent-1-yne-3,1-diyl))benzene (5.0 mg, 10%) as a yellow oil and (oxybis(pent-1-yne-3,1-diyl))benzene (**3a**) (9.40 mg, 10%) as a yellow oil.

Entry 6: To a nitromethane (0.500 mL) solution of 1-phenylpent-1-yn-3-ol (**1a**) (25.0 mg, 0.156 mmol), 1,2,3-trimethoxybenzene (78.7 mg, 0.468 mmol), and tetrabutylammonium hexafluorophosphate (12.1 mg, 0.0312 mmol) were added titanium tetrachloride (5.9 mg, 0.0312 mmol). The reaction mixture was stirred at 100 °C for 1.5 h and then poured into water (50 mL). The organic layer was separated and the aqueous layer was extracted with AcOEt. The combined organic layer was dried over $MgSO_4$ and the solvent was removed under reduced pressure. The residue was purified by preparative TLC on silica gel eluting with AcOEt-*n*-hexane (1:40) to give 1,2,3-trimethoxy-4-(1-phenylpent-1-yn-3-yl)benzene (**2a**) (10.0 mg, 21%) as a yellow oil and (oxybis(pent-1-yne-3,1-diyl))benzene (**3a**) (5.3 mg, 11%) as a yellow oil and recover 1-phenylpent-1-yn-3-ol (16.7 mg, 67%).

Entry 7: To a nitromethane (0.500 mL) solution of 1-phenylpent-1-yn-3-ol (**1a**) (25.0 mg, 0.156 mmol), 1,2,3-trimethoxybenzene (78.7 mg, 0.468 mmol), and tetrabutylammonium hexafluorophosphate (12.1 mg, 0.0312 mmol) were added aluminium trichloride (11.3 mg, 0.0312 mmol). The reaction mixture was stirred at 100 °C for 10 min and then the almost same procedure as entry 1 was performed. The residue was purified by preparative TLC on silica gel eluting with AcOEt-*n*-hexane (1:40) to give 1,2,3-trimethoxy-4-(1-phenylpent-1-yn-3-yl)benzene (**2a**) (3.2 mg, 7%) as a yellow oil and recover 1-phenylpent-1-yn-3-ol (**1a**) (15.2 mg, 61%).

Entry 8: To a nitromethane (0.500 mL) solution of 1-phenylpent-1-yn-3-ol (**1a**) (25.0 mg, 0.156 mmol), 1,2,3-trimethoxybenzene (78.7 mg, 0.468 mmol), and tetrabutylammonium hexafluorophosphate (12.1 mg, 0.0312 mmol) were added copper(II) triflate (4.2 mg, 0.0312 mmol). The reaction mixture was stirred at 100 °C for 1.5 h and then the almost same procedure as entry 1 was performed. The residue was purified by preparative TLC on silica gel eluting with AcOEt-*n*-hexane (1:40) to give 1,2,3-trimethoxy-4-(1-phenylpent-1-yn-3-yl)benzene (**2a**) (17.4 mg, 36%) as a yellow oil.

Entry 9: To a nitromethane (0.500 mL) solution of 1-phenylpent-1-yn-3-ol (**1a**) (25.0 mg, 0.156 mmol) and 1,2,3-trimethoxybenzene (78.7 mg, 0.468 mmol) were added indium trichloride (15.4 mg, 0.0312 mmol). The reaction mixture was stirred at 100 °C for 1 h and then the almost same procedure as entry 1 was performed. The residue was purified by preparative TLC on silica gel eluting with AcOEt-*n*-hexane (1:40) to give 1,2,3-trimethoxy-4-(1-phenylpent-1-yn-3-yl)benzene (**2a**) (28.4 mg, 59%) as a yellow oil.

Entry 10: To a nitromethane (1.00 mL) solution of 1-phenylpent-1-yn-3-ol (**1a**) (50.0 mg, 0.312 mmol), 1,2,3-trimethoxybenzene (157 mg, 0.468 mmol), and tetrabutylammonium hexafluorophosphate (24.2 mg, 0.0624 mmol) were added indium trichloride (13.8 mg, 0.0624

mmol). The reaction mixture was stirred at room temperature for 3.5 h and then the almost same procedure as entry 1 was performed. The residue was purified by preparative TLC on silica gel eluting with AcOEt-*n*-hexane (1:40) to give 1,2,3-trimethoxy-4-(1-phenylpent-1-yn-3-yl)benzene (41.8 mg, 43%) as a yellow oil, (oxybis(pent-1-yne-3,1-diyl))benzene (**3a**) (4.5 mg, 9.5%) as a yellow oil, and **1a** (18.0 mg, 36%).

Entry 11: To a nitromethane (1.00 mL) solution of 1-phenylpent-1-yn-3-ol (**1a**) (50.0 mg, 0.312 mmol), 1,2,3-trimethoxybenzene (157 mg, 0.468 mmol), and tetrabutylammonium hexafluorophosphate (24.2 mg, 0.0624 mmol) were added silver perchlorate (12.9 mg, 0.0624 mmol) and indium trichloride (13.8 mg, 0.0624 mmol). The reaction mixture was stirred at room temperature for 1.0 h and then the almost same procedure as entry 1 was performed. The residue was purified by preparative TLC on silica gel eluting with AcOEt-*n*-hexane (1:40) to give 1,2,3-trimethoxy-4-(1-phenylpent-1-yn-3-yl)benzene (54.1 mg, 55.9%) as a yellow oil, 1,2,3-trimethoxy-5-(1-phenylpent-1-yn-3-yl)benzene (**2a**) (3.00 mg, 3.1%) as a yellow oil, (oxybis(pent-1-yne-3,1-diyl))benzene (**3a**) (3.20 mg, 6.8%) as a yellow oil, and **1a** (7.20 mg, 14.4%).

Entry 12: To a dichloromethane (1.00 mL) solution of 1-phenylpent-1-yn-3-ol (**1a**) (50.0 mg, 0.312 mmol), 1,2,3-trimethoxybenzene (157 mg, 0.468 mmol) were added silver hexafluoroantimonate (21.4 mg, 0.0624 mmol) and indium trichloride (13.8 mg, 0.0624 mmol). The reaction mixture was stirred at room temperature for 1.0 h and then the almost same procedure as entry 1 was performed. The residue was purified by preparative TLC on silica gel eluting with AcOEt-*n*-hexane (1:40) to give 1,2,3-trimethoxy-4-(1-phenylpent-1-yn-3-yl)benzene (25.8 mg, 27%) as a yellow oil, (oxybis(pent-1-yne-3,1-diyl))benzene (**3a**) (5.20 mg, 11%) as a yellow oil, and **1a** (26.8 mg, 53.6%).

Entry 13: To a nitromethane (1.00 mL) solution of 1-phenylpent-1-yn-3-ol (**1a**) (50.0 mg, 0.312 mmol), 1,2,3-trimethoxybenzene (157 mg, 0.468 mmol) were added silver hexafluoroantimonate (21.4 mg, 0.0624 mmol) and indium trichloride (13.8 mg, 0.0624 mmol). The reaction mixture was stirred at room temperature overnight and then the almost same procedure as entry 1 was performed. The residue was purified by preparative TLC on silica gel eluting with AcOEt-*n*-hexane (1:40) to give 1,2,3-trimethoxy-4-(1-phenylpent-1-yn-3-yl)benzene (43.5 mg, 45%) as a yellow oil, 1,2,3-trimethoxy-5-(1-phenylpent-1-yn-3-yl)benzene (**2a**) (8.00 mg, 8.3%) as a yellow oil, (oxybis(pent-1-yne-3,1-diyl))benzene (**3a**) (4.9 mg, 10%) as a yellow oil, and **1a** (6.00 mg, 12%).

Entry 14: To a nitromethane (1.00 mL) solution of 1-phenylpent-1-yn-3-ol (**1a**) (50.0 mg, 0.312 mmol), 1,2,3-trimethoxybenzene (157 mg, 0.468 mmol) were added silver hexafluorophosphate (42.9 mg, 0.125 mmol) and indium trichloride (13.8 mg, 0.0624 mmol). The reaction mixture was stirred at room temperature for 1h and then the almost same procedure as entry 1 was performed. The residue was purified by preparative TLC on silica gel eluting with AcOEt-*n*-hexane (1:40) to give 1,2,3-trimethoxy-4-(1-phenylpent-1-yn-3-yl)benzene (56.2 mg, 58%) as a yellow oil and **1a** (6.00 mg, 6%).

Entry 15: To a nitromethane (0.500 mL) solution of 1-phenylpent-1-yn-3-ol (**1a**) (25.0 mg, 0.156 mmol) and 1,2,3-trimethoxybenzene (78.7 mg, 0.468 mmol), and tetrabutylammonium hexafluorophosphate (12.1 mg, 0.0312 mmol) were added silver perchlorate (16.2 mg, 0.0780 mmol) and indium trichloride (6.9 mg, 0.0312 mmol). The reaction mixture was stirred at room temperature for 30 min and then the almost same procedure as entry 10 was performed. The residue was purified by preparative TLC on silica gel eluting with AcOEt-*n*-hexane (1:40) to give 1,2,3-trimethoxy-4-(1-phenylpent-1-yn-3-yl)benzene (**2a**) (37.2 mg, 77%) as a yellow oil, 1,2,3-trimethoxy-5-(1-phenylpent-1-yn-3-yl)benzene (**2a**) (4.8 mg, 10%) as a yellow oil, and (oxybis(pent-1-yne-3,1-diyl))benzene (**3a**) (3.1 mg, 7%) as a yellow oil.

Entry 16: To a nitromethane (1.500 mL) solution of 1-phenylpent-1-yn-3-ol (**1a**) (50.0 mg, 0.312 mmol) and 1,2,3-trimethoxybenzene (157 mg, 0.936 mmol), and tetrabutylammonium hexafluorophosphate (24.0 mg, 0.0624 mmol), 1,1'-binaphthol (17.6 mg, 0.0624 mmol) were added silver perchlorate (16.2 mg, 0.156 mmol) and indium trichloride (13.8 mg, 0.0624 mmol). The reaction mixture was stirred at room temperature for 30 min and then the almost same procedure as entry 1 was performed. The residue was purified by preparative TLC on silica gel eluting with AcOEt-*n*-hexane (1:40) to give 1,2,3-trimethoxy-4-(1-phenylpent-1-yn-3-yl)benzene (**2a**) (66.1 mg, 68%) as a yellow oil, 1,2,3-trimethoxy-5-(1-phenylpent-1-yn-3-yl)benzene (**2a**) (7.7 mg, 8%) as a yellow oil.

Entry 17: To a nitromethane (0.500 mL) solution of 1-phenylpent-1-yn-3-ol (**1a**) (25.0 mg, 0.156 mmol) and 1,2,3-trimethoxybenzene (79.0 mg, 0.486 mmol), and tetrabutylammonium hexafluorophosphate (12.1 mg, 0.0312 mmol) was added indium tribromide (10.8 mg, 0.0312 mmol). The reaction mixture was refluxed for 10 min and then the almost same procedure as entry 1 was performed. The residue was purified by preparative TLC on silica gel eluting with AcOEt-*n*-hexane (1:40) to give 1,2,3-trimethoxy-4-(1-phenylpent-1-yn-3-yl)benzene (31.0 mg, 64%) as a yellow oil, 1,2,3-trimethoxy-5-(1-phenylpent-1-yn-3-yl)benzene (**2a**) (3.2 mg, 7%) as a yellow oil, and (oxybis(pent-1-yne-3,1-diyl))benzene (**3a**) (1.0 mg, 2%) as a yellow oil.

Entry 18: To a nitromethane (0.500 mL) solution of 1-phenylpent-1-yn-3-ol (**1a**) (25.0 mg, 0.156 mmol) and 1,2,3-trimethoxybenzene (79.0 mg, 0.486 mmol), and tetrabutylammonium hexafluorophosphate (12.1 mg, 0.0312 mmol) were added silver perchlorate (16.2 mg, 0.0780 mmol) and indium tribromide (10.8 mg, 0.0312 mmol). The reaction mixture was stirred at room temperature for 30 min and then the almost same procedure as entry 13 was performed. The residue was purified by preparative TLC on silica gel eluting with AcOEt-*n*-hexane (1:40) to give 1,2,3-trimethoxy-4-(1-phenylpent-1-yn-3-yl)benzene (**2a**) (32.1 mg, 66%) as a yellow oil, 1,2,3-trimethoxy-5-(1-phenylpent-1-yn-3-yl)benzene (5.1 mg, 11%) as a yellow oil, and (oxybis(pent-1-yne-3,1-diyl))benzene (**3a**) (2.4 mg, 5%) as a yellow oil.

Entry 19: To a nitromethane (0.500 mL) solution of 1-phenylpent-1-yn-3-ol (**1a**) (25.0 mg, 0.156 mmol) and 1,2,3-trimethoxybenzene (39.4 mg, 0.234 mmol), and tetrabutylammonium hexafluorophosphate (12.1 mg, 0.0312 mmol) were added silver perchlorate (16.2 mg, 0.0780 mmol) and indium trichloride (6.9 mg, 0.0312 mmol). The reaction mixture was stirred at room temperature for 40 min and then the almost

same procedure as entry 1 was performed. The residue was purified by preparative TLC on silica gel eluting with AcOEt-*n*-hexane (1:40) to give 1,2,3-trimethoxy-4-(1-phenylpent-1-yn-3-yl)benzene (**2a**) (66.1 mg, 68.3%) as a yellow oil, 1,2,3-trimethoxy-5-(1-phenylpent-1-yn-3-yl)benzene (3.4 mg, 7%) as a yellow oil, and (oxybis(pent-1-yne-3,1-diyl))benzene (**3a**) (1.3 mg, 3%) as a yellow oil.

Entry 20: To a nitromethane (0.500 mL) solution of 1-phenylpent-1-yn-3-ol (**1a**) (25.0 mg, 0.156 mmol) and 1,2,3-trimethoxybenzene (39.4 mg, 0.234 mmol), and tetrabutylammonium hexafluorophosphate (12.1 mg, 0.0312 mmol) were added indium tribromide (11.1 mg, 0.0312 mmol). The reaction mixture was stirred at rt for 10 min and then the almost same procedure as entry 1 was performed. The residue was purified by preparative TLC on silica gel eluting with AcOEt-*n*-hexane (1:40) to give 1,2,3-trimethoxy-4-(1-phenylpent-1-yn-3-yl)benzene (**2a**) (26.9 mg, 56%) as a yellow oil.

Entry 21: To a nitromethane (0.500 mL) and HMPA (28.0 mg, 0.156mmol) solution of 1-phenylpent-1-yn-3-ol (**1a**) (25.0 mg, 0.156 mmol) and 1,2,3-trimethoxybenzene (39.4 mg, 0.234 mmol), and tetrabutylammonium hexafluorophosphate (12.1 mg, 0.0312 mmol) were added indium tribromide (11.1 mg, 0.0312 mmol). The reaction mixture was stirred at 100 ºC for 10 min and then the almost same procedure as entry 1 was performed. The residue was purified by preparative TLC on silica gel eluting with AcOEt-*n*-hexane (1:40) to recover 1-phenylpent-1-yn-3-ol (**2a**) (19.5 mg, 78%).

Entry 22: To a nitromethane (0.500 mL) and DMF (11.4 mg, 0.156mmol) solution of 1-phenylpent-1-yn-3-ol (**1a**) (25.0 mg, 0.156 mmol) and 1,2,3-trimethoxybenzene (39.4 mg, 0.234 mmol), and tetrabutylammonium hexafluorophosphate (12.1 mg, 0.0312 mmol) were added indium tribromide (11.1 mg, 0.0312 mmol). The reaction mixture was stirred at 100 ºC for 1 h and then the almost same procedure as entry 1 was performed. The residue was purified by preparative TLC on silica gel eluting with AcOEt-*n*-hexane (1:40) to give 1,2,3-trimethoxy-4-(1-phenylpent-1-yn-3-yl)benzene (**2a**) (6.5 mg, 13%) as a yellow oil and recover 1-phenylpent-1-yn-3-ol (**1a**) (12.7 mg, 51%).

Entry 23: To a nitromethane (0.500 mL) and water (0.0500 mL) solution of 1-phenylpent-1-yn-3-ol (**1a**) (25.0 mg, 0.156 mmol) and 1,2,3-trimethoxybenzene (39.4 mg, 0.234 mmol), and tetrabutylammonium hexafluorophosphate (12.1 mg, 0.0312 mmol) were added silver perchlorate (16.2 mg, 0.0780 mmol) and indium trichloride-4H_2O (11.1 mg, 0.0312 mmol). The reaction mixture was stirred at 100 ºC for 1 h and then the almost same procedure as entry 1 was performed. The residue was purified by preparative TLC on silica gel eluting with AcOEt-*n*-hexane (1:40) to give 1,2,3-trimethoxy-4-(1-phenylpent-1-yn-3-yl)benzene (**2a**) (13.5 mg, 28%) as a yellow oil.

Entry 24: To a nitromethane (0.500 mL) and solution of 1-phenylpent-1-yn-3-ol (**1a**) (25.0 mg, 0.156 mmol) and 1,2,3-trimethoxybenzene (39.4 mg, 0.234 mmol), and tetrabutylammonium hexafluorophosphate (12.1 mg, 0.0312 mmol) were added silver trifluoroacetate (17.2 mg, 0.0780 mmol) and indium tribromide (11.1 mg, 0.0312 mmol). The reaction mixture was stirred at 100 ºC for 30 min and then the almost same procedure as entry 1 was performed. The residue was purified by preparative TLC on silica gel eluting with AcOEt-*n*-hexane (1:40) to give 1,2,3-trimethoxy-4-(1-phenylpent-1-yn-3-yl)benzene (**2a**) (8.0 mg, 17%) as a yellow oil and (oxybis(pent-1-yne-3,1-diyl))benzene (**3a**) (1.3 mg, 3%) as a yellow oil and recover 1-phenylpent-1-yn-3-ol (**1a**) (13.0 mg, 52%).

Entry 25: To a nitromethane (0.500 mL) solution of 1-phenylpent-1-yn-3-ol (**1a**) (25.0 mg, 0.156 mmol) and 1,2,3-trimethoxybenzene (39.4 mg, 0.234 mmol), and tetrabutylammonium hexafluorophosphate (12.1 mg, 0.0312 mmol) were added indium tribromide (10.8 mg, 0.0312 mmol), Salen complex (8.4 mg, 0.312 mmol), and silver perchlorate (16.2 mg, 0.0780 mmol). The reaction mixture was stirred at 100 for 30 min and then the almost same procedure as entry 1 was performed. The residue was purified by preparative TLC on silica gel eluting with AcOEt-*n*-hexane (1:40) to give 1,2,3-trimethoxy-4-(1-phenylpent-1-yn-3-yl)benzene (**2a**) (13.3 mg, 28%) as a yellow oil and (oxybis(pent-1-yne-3,1-diyl))benzene (**3a**) (0.6 mg, 1%) as a yellow oil and recover 1-phenylpent-1-yn-3-ol (**1a**)(9.0 mg, 36%).

Entry 26: To a nitromethane (0.500 mL) solution of 1-phenylpent-1-yn-3-ol (**1a**) (25.0 mg, 0.156 mmol) and 1,2,3-trimethoxybenzene (39.4 mg, 0.234 mmol), and tetrabutylammonium hexafluorophosphate (12.1 mg, 0.0312 mmol) were added silver perchlorate (16.2 mg, 0.0780 mmol) and indium triflate (1.7 mg, 7.8×10^{-3} mmol). The reaction mixture was stirred at 50 ºC for 1 h and then the almost same procedure as entry 1 was performed. The residue was purified by preparative TLC on silica gel eluting with AcOEt-*n*-hexane (1:40) to give 1,2,3-trimethoxy-4-(1-phenylpent-1-yn-3-yl)benzene (**2a**) (24.6 mg, 51%) as a yellow oil and 1,2,3-trimethoxy-5-(1-phenylpent-1-yn-3-yl)benzene (**2a**) (4.0 mg, 8%) as a yellow oil.

Entry 27: To a nitromethane (0.500 mL) solution of 1-phenylpent-1-yn-3-ol (**1a**) (25.0 mg, 0.156 mmol) and 1,2,3-trimethoxybenzene (39.4 mg, 0.234 mmol), and tetrabutylammonium hexafluorophosphate (12.1 mg, 0.0312 mmol) were added silver perchlorate (16.2 mg, 0.0780 mmol) and indium trichloride (1.7 mg, 7.8×10^{-3} mmol). The reaction mixture was stirred at 50 ºC for 1 h and then the almost same procedure as entry 1 was performed. The residue was purified by preparative TLC on silica gel eluting with AcOEt-*n*-hexane (1:40) to give 1,2,3-trimethoxy-4-(1-phenylpent-1-yn-3-yl)benzene (**2a**) (24.6 mg, 51%) as a yellow oil and 1,2,3-trimethoxy-5-(1-phenylpent-1-yn-3-yl)benzene (**2a**) (4.0 mg, 8%) as a yellow oil.

Entry 28: To a nitromethane (0.500 mL) solution of 1-phenylpent-1-yn-3-ol (25.0 mg, 0.156 mmol) and 1,2,3-trimethoxybenzene (39.4 mg, 0.234 mmol), and tetrabutylammonium hexafluorophosphate (12.1 mg, 0.0312 mmol) were added silver perchlorate (16.2 mg, 0.0780 mmol), trifluoromethanesulfonimide (4.4 mg, 0.0156 mmol), and indium trichloride (1.7 mg, 7.8×10^{-3} mmol). The reaction mixture was stirred at 50 ºC for 40 min and then the almost same procedure as entry 1 was performed. The residue was purified by preparative TLC on silica gel eluting with AcOEt-*n*-hexane (1:40) to give 1,2,3-trimethoxy-4-(1-phenylpent-1-yn-3-yl)benzene (**2a**) (26.6 mg, 55%) as a yellow oil, 1,2,3-trimethoxy-5-(1-phenylpent-1-yn-3-yl)benzene (**2a**) (3.6 mg, 7%) as a yellow oil, and (oxybis(pent-1-yne-3,1-diyl))benzene (2.3 mg, 5%) as a yellow oil.

Entry 29: To a nitromethane (1.500 mL) solution of 1-phenylpent-1-yn-3-ol (**1a**) (50.0 mg, 0.312 mmol) and 1,2,3-trimethoxybenzene (78.7 mg, 0.468 mmol), and tetrabutylammonium hexafluorophosphate (24.0 mg, 0.0624 mmol), 1,1'-binaphthol (17.6 mg, 0.0624 mmol) were added silver perchlorate (6.47 mg, 0.0312 mmol) and indium trichloride (3.45 mg, 0.0156 mmol). The reaction mixture was stirred at 40 °C for 3 h and then the almost same procedure as entry 1 was performed. The residue was purified by preparative TLC on silica gel eluting with AcOEt-*n*-hexane (1:40) to give 1,2,3-trimethoxy-4-(1-phenylpent-1-yn-3-yl)benzene (**2a**) (57.0 mg, 59%) as a yellow oil, 1,2,3-trimethoxy-5-(1-phenylpent-1-yn-3-yl)benzene (**2a**) (8.1 mg, 8.4%) as a yellow oil.

Experiment for Scalability (Scheme 10).

To a nitromethane (30.0 mL) solution of 1-phenylpent-1-yn-3-ol (3.16 g, 19.0 mmol) and 1,2,3-trimethoxybenzene (2.66 g, 15.8 mmol), and tetrabutylammonium hexafluorophosphate (612 mg, 1.58 mmol) were added silver perchlorate (450 mg, 1.58 mmol), 1,1'-binaphthol (0.45 g, 1.58 mmol) and indium trichloride (140 mg, 0.633 mmol). The reaction mixture was stirred at room temperature for 2 h and performed the same processing way as **Entry** 10 of **Table 1**. The residue was purified by column chromatography on silica gel eluting with AcOEt-*n*-hexane (1:20) to give 1,2,3-trimethoxy-4-(1-phenylpent-1-yn-3-yl)benzene (**2a**) (4.50 g, 92%) as a yellow oil, 1,2,3-trimethoxy-5-(1-phenylpent-1-yn-3-yl)benzene (**2a**) (98 mg, 2%) as a yellow oil.

IR (KBr, cm^{-1}) δ 3447, 2967, 2930, 1715, 1491, 1461, 1412, 1312, 1219, 1167, 1081, 1050, 1011, 757, 692; ^{1}H NMR (600 MHz, $CDCl_3$) δ 1.07 (3H, t, J = 7.6Hz, CH_3), 1.77-1.82 (2H, m, CH_2), 3.85 (3H, s, OCH_3), 3.88 (3H, s, OCH_3), 3.94 (3H, s, OCH_3), 4.10 (1H, dd, J = 5.5 and 8.2 Hz, CH), 6.68 (1H, d, J = 8.9 Hz, ArH), 7.23 (1H, d, J = 8.9 Hz, ArH), 7.27-7.28 (3H, m, ArH), 7.43-7.44 (2H, m, ArH); ^{13}C NMR (150 MHz, $CDCl_3$) δ 12.0 (q), 30.6 (t), 33.2 (d), 55.9 (q), 60.7 (q), 61.1 (q), 82.3 (s), 92.2 (s), 107.1 (d), 122.7 (d), 123.9 (s), 127.6 (d), 128.0 (s), 128.1 (d×2), 131.6 (d×2), 141.9 (s), 150.9 (s), 152.4 (s); MS m/z 310 (M^+), 295 (M^+-Me), 281 (M^+-OMe); high resolution mass calcd for $C_{20}H_{22}O_3$: 310.1569, found m/z 310.1569. Anal. Calcd for $C_{20}H_{22}O_3$ (+1/10H_2O): C, 76.95; H, 7.17. Found: C, 76.85; H, 7.24.

Time course experiments for 2a.

Typical procedure for time-course experiments.

(1) Bu_4NPF_6 (0.2 eq), 1,1'-binaphthol (0.2 eq), $AgClO_4$ (0.4 eq), $InCl_3$ (0.2 eq): To a nitromethane (1.00 mL) solution of 1-phenylpent-1-yn-3-ol (**1a**) (20.0 mg, 0.125 mmol) and 1,2,3-trimethoxybenzene (62.9 mg, 0.374 mmol), and tetrabutylammonium hexafluorophosphate (9.70 mg, 0.0250 mmol), 1,1'-binaphthol (7.20 mg, 0.0250 mmol) were added silver perchlorate (10.4 mg, 0.0499 mmol) and indium trichloride (5.50 mg, 0.0624 mmol). The reaction mixture was stirred at 20 °C for 5 min and then poured into water (50.0 mL). The workup procedure as described above gave 1,2,3-trimethoxy-4-(1-phenylpent-1-yn-3-yl)benzene (**2a**) (10.0 mg, 26%), 1,2,3-trimethoxy-5-(1-phenylpent-1-yn-3-yl)benzene (**2a**) (1.5 mg, 4%), and (oxybis(pent-1-yne-3,1-diyl))benzene (**3a**) (1.80 mg, 5%), and 1-phenylpent-1-yn-3-ol (**1a**) (11.4 mg, 57%). The same procedure for 10 min stirring gave 1,2,3-trimethoxy-4-(1-phenylpent-1-yn-3-yl)benzene (**2a**) (15.2 mg, 39%), 1,2,3-trimethoxy-5-(1-phenylpent-1-yn-3-yl)benzene (**2a**) (1.50 mg, 4%), and (oxybis(pent-1-yne-3,1-diyl))benzene (**3a**) (1.20 mg, 3%) 1-phenylpent-1-yn-3-ol (**1a**) (6.80 mg, 34%). 30 Min: 1,2,3-trimethoxy-5-(1-phenylpent-1-yn-3-yl)benzene (**2a**) (1.10 mg, 3%), (oxybis(pent-1-yne-3,1-diyl))benzene (**3a**) (0.80 mg, 2%), 1-phenylpent-1-yn-3-ol (**1a**) (1.90 mg, 10%).

(2) Bu_4NPF_6 (0.2 eq), $AgClO_4$ (0.4 eq), $InCl_3$ (0.2 eq):

To a nitromethane (1.00 mL) solution of 1-phenylpent-1-yn-3-ol (**1a**) (20.0 mg, 0.125 mmol) and 1,2,3-trimethoxybenzene (62.9 mg, 0.374 mmol), and tetrabutylammonium hexafluorophosphate (9.70 mg, 0.0250 mmol) were added silver perchlorate (10.4 mg, 0.0499 mmol) and indium trichloride (5.50 mg, 0.0624 mmol). The reaction mixture was stirred at 20ºC for 10 min and then the workup procedure gave 1,2,3-trimethoxy-4-(1-phenylpent-1-yn-3-yl)benzene (**2a**) (7.30 mg, 19%), 1,2,3-trimethoxy-5-(1-phenylpent-1-yn-3-yl)benzene (**2a**) (0.60 mg, 1%), (oxybis(pent-1-yne-3,1-diyl))benzene (**3a**) (1.20 mg, 3%) and 1-phenylpent-1-yn-3-ol (**1a**) (11.4 mg, 57%). 20 Min: 1,2,3-trimethoxy-4-(1-phenylpent-1-yn-3-yl)benzene (**2a**) (8.60 mg, 22%), and (oxybis(pent-1-yne-3,1-diyl))benzene (**3a**) (1.00 mg, 3%), 1-phenylpent-1-yn-3-ol (**1a**) (13.2 mg, 66%). 120 Min:1,2,3-trimethoxy-4-(1-phenylpent-1-yn-3-yl)benzene (**2a**) (23.0 mg, 59%), 1,2,3-trimethoxy-5-(1-phenylpent-1-yn-3-yl)benzene (**2a**) (3.40 mg, 9%), (oxybis(pent-1-yne-3,1-diyl))benzene (**3a**) (2.50 mg, 7%), 1-phenylpent-1-yn-3-ol (**1a**) (1.70 mg, 9%).

(3) $AgClO_4$ (0.4 eq), $InCl_3$ (0.2 eq):

To a nitromethane (1.00 mL) solution of 1-phenylpent-1-yn-3-ol (**1a**) (20.0 mg, 0.125 mmol) and 1,2,3-trimethoxybenzene (62.9 mg, 0.374 mmol) were added silver perchlorate (10.4 mg, 0.0499 mmol) and indium trichloride (5.50 mg, 0.0624 mmol). The reaction mixture was stirred at 20ºC for 60 min and then poured into water (50.0 mL). 1,2,3-trimethoxy-4-(1-phenylpent-1-yn-3-yl)benzene (**2a**) (4.40 mg, 11%), 1,2,3-trimethoxy-5-(1-phenylpent-1-yn-3-yl)benzene (**2a**) (0.50 mg, 1%), (oxybis(pent-1-yne-3,1-diyl))benzene (**3a**) (1.20 mg, 3%), 1-phenylpent-1-yn-3-ol (**1a**) (12.3 mg, 62%). 120 Min: 1,2,3-trimethoxy-4-(1-phenylpent-1-yn-3-yl)benzene (**2a**) (15.5 mg, 40%), 1,2,3-trimethoxy-5-(1-phenylpent-1-yn-3-yl)benzene (**2a**) (1.50 mg, 4%), (oxybis(pent-1-yne-3,1-diyl))benzene (**3a**) (1.70 mg, 5%), and recover 1-phenylpent-1-yn-3-ol (**1a**) (4.10 mg, 21%).

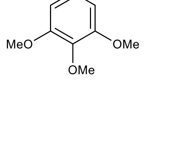

1,2,3-trimethoxy-5-(1-phenylpent-1-yn-3-yl)benzene (5-**2a**)

IR (KBr, cm^{-1}) ν 3434, 2925, 2851, 1715, 1463, 1333, 1129, 1093, 757; ^{1}H NMR (600 MHz, $CDCl_3$) δ 1.00 (3H, t, J = 7.6 Hz, CH_3), 1.71-1.84 (2H, m, CH_2), 3.65 (1H, dd, J = 6.2 and 2.0 Hz, CH), 3.77 (3H, s, OCH_3), 3.80

(6H, s, OCH_3), 6.58 (2H, s, ArH), 7.22-7.24 (3H, m, ArH), 7.35-7.38 (2H, m, ArH); ^{13}C NMR (150 MHz, $CDCl_3$) δ 11.9 (q), 31.7 (t), 40.3 (d), 56.1 (q×2), 60.8 (q), 83.5 (s), 91.3 (s), 104.5 (d×2), 123.7 (s), 127.8 (d), 128.2 (d×2), 131.6 (d×2), 136.6 (s), 137.8 (s), 153.1 (s); EIMS m/z 310 (M^+), 295 (M^+-Me); high resolution mass calcd for $C_{20}H_{22}O_3$: 310.1569, found m/z 310.1594.

(oxybis(pent-1-yne-3,1-diyl))dibenzene (**3a**).

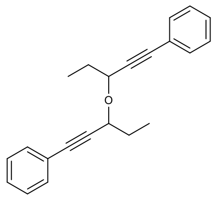

First fraction: IR (KBr, cm^{-1}) ν 3466, 2926, 2851, 1597, 1496, 1478, 1255, 1114, 1011, 756, 693; ^1H NMR (600 MHz, $CDCl_3$) δ 1.10 (3H, t, J = 7.6 Hz, CH_3), 1.84-1.90 (2H, m, CH_2), 4.64-4.66 (1H, m, CH), 7.31-7.32 (3H, m, ArH), 7.45-7.46 (2H, m, ArH); ^{13}C NMR (150 MHz, $CDCl_3$) δ 9.8 (q×2), 29.1 (t×2), 69.1 (d×2), 85.6 (s×2), 88.3 (s×2), 122.9 (s×2), 128.2 (d×6), 131.8 (d×4); EIMS m/z 301 (M^+), 244 (M^+ -$OCHCH_2CH_3$), 202 (M^+ -$O(CHCH_2CH_3)_2$). Anal. Calcd for $C_{22}H_{22}O$: C, 87.38; H, 7.33. Found: C, 64.20; H, 7.02.

Second fraction: IR (KBr, cm^{-1}) ν 3442, 2967, 2926, 2852, 1715, 1490, 1463, 1443, 1362, 1099, 1061, 756, 691; ^1H NMR (600 MHz, $CDCl_3$) δ 1.10 (3H, t, J = 7.5 Hz, CH_3), 1.86-1.92 (2H, m, CH_2), 4.49-4.52 (1H, m, CH), 7.25-7.28 (3H, m, ArH), 7.40-7.42 (2H, m, ArH); ^{13}C NMR (150 MHz, $CDCl_3$) δ 9.8 (q×2), 28.7 (t×2), 70.1 (d×2), 85.6 (s×2), 88.7 (s×2), 123.0 (s×2), 128.1 (d×6), 131.8 (d×4); EIMS m/z 302 (M^+), 273 (M^+ -Et), 202 (M^+ -$O(CHCH_2CH_3)_2$); high resolution mass calcd for $C_{22}H_{22}O$: 301.1671, found m/z 302.1690.

Preparation of 1,2,3-trimethoxy-4-(5-methyl-1-phenylhex-1-yn-3-yl)benzene (**2b**).

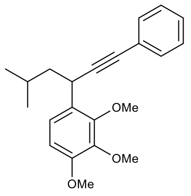

To a nitromethane (1.00 mL) solution of 5-methyl-1-phenylhex-1-yn-3-ol (**1a**)(60.0 mg, 0.319 mmol), 1,2,3-trimethoxybenzene (0.134 g, 0.797 mmol), (R)-(+)-1,1'-bi-2-naphthol (15.2 mg, 0.0536 mmol) and tetrabutylammonium hexafluorophosphate (20.8 mg, 0.0536 mmol) were added silver perchlorate (27.8 mg, 0.134 mmol) and indium trichloride (11.9 mg, 0.0536 mmol) at room temperature. The reaction mixture was stirred for 0.5 h and then filtered through a pad of celite with chloroform and then poured into water (50.0 mL). The organic layer was separated and the aqueous layer was extracted with AcOEt. The combined organic layer was dried over $MgSO_4$. The solvent was removed under reduced pressure. The residue was purified by preparative TLC on silica gel eluting with AcOEt-*n*-hexane (1:30) to give 1,2,3-trimethoxy-4-(5-methyl-1-phenylhex-1-yn-3-yl)benzene (**2b**) (80.0 mg, 74%) as a yellow oil, 1,2,3-trimethoxy-5-(5-methyl-1-phenylhex-1-yn-3-yl)benzene (**2b**) (28.9 mg, 26%) as a yellow oil.

(4)-**2b**: IR (KBr, cm^{-1}) ν 2955, 1715, 1599, 1493, 1466, 1417, 1366, 1294, 1276, 1095, 1018, 799, 767, 692, 532; ^1H NMR (600 MHz, $CDCl_3$) δ 0.97 (3H, d, J = 6.9 Hz, CH_3), 1.02 (3H, d, J = 6.2 Hz, CH_3), 1.50-1.54 (1H, m, CH_2), 1.71-1.76 (1H, m, CH_2), 1.90-1.93 (1H, m, CH), 3.85 (3H, s, OCH_3), 3.88 (3H, s, OCH_3), 3.94 (3H, s, OCH_3), 4.20-4.23 (1H, m, CH), 6.68 (1H, d, J = 8.9 Hz, ArH), 7.23 (1H, d, J = 8.9 Hz, ArH), 7.26-7.28 (3H, m, ArH), 7.42 (2H, dd, J = 2.0 and 7.5 Hz, ArH); ^{13}C NMR (150 MHz, $CDCl_3$) δ 21.6 (q), 23.3 (q), 26.4 (d), 29.7 (d), 47.1 (t), 56.0 (q), 60.7 (q), 61.1 (q), 82.0 (s), 92.4 (s), 107.4 (d), 122.5 (d), 124.0 (s), 127.5 (d), 128.1 (d×2), 128.7 (s), 131.6 (d×2), 142.0 (s), 150.8 (s), 152.4 (s); MS m/z 338 (M^+), 323 (M^+-Me), 307 (M^+-OMe), 295 (M^+-^{i}Pr), 281 (M^+-^{i}Bu); high resolution mass calcd for $C_{22}H_{26}O_3$: 338.1882, found m/z 338.1863. Anal. Calcd for $C_{22}H_{26}O_3$: C, 78.07; H, 7.74; N. Found: C, 78.13; H, 7.75.

(5)-**2b**: IR (KBr, cm^{-1}) ν 2955, 1715, 1599, 1493, 1466, 1417, 1366, 1294, 1276, 1095, 1018, 799, 767, 692, 532; ^1H NMR (600 MHz, $CDCl_3$)

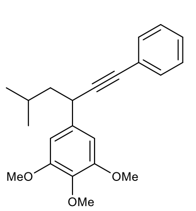

δ 0.97 (3H, d, J = 6.9 Hz, CH_3), 1.02 (3H, d, J = 6.2 Hz, CH_3), 1.50-1.54 (1H, m, CH_2), 1.71-1.76 (1H, m, CH_2), 1.90-1.93 (1H, m, CH), 3.85 (3H, s, OCH_3), 3.88 (3H, s, OCH_3), 3.94 (3H, s, OCH_3), 4.20-4.23 (1H, m, CH), 6.68 (1H, d, J = 8.9 Hz, ArH), 7.23 (1H, d, J = 8.9 Hz, ArH), 7.26-7.28 (3H, m, ArH), 7.42 (2H, dd, J = 2.0 and 7.5 Hz, ArH); ^{13}C NMR (150 MHz, $CDCl_3$) δ 21.6 (q), 23.3 (q), 26.4 (d), 29.7 (d), 47.1 (t), 56.0 (q), 60.7 (q), 61.1 (q), 82.0 (s), 92.4 (s), 107.4 (d), 122.5 (d), 124.0 (s), 127.5 (d), 128.1 (d×2), 128.7 (s), 131.6 (d×2), 142.0 (s), 150.8 (s), 152.4 (s); MS m/z 338 (M^+), 323 (M^+ -Me), 307 (M^+ -OMe), 295 (M^+ -^{i}Pr), 281 (M^+ -^{i}Bu); high resolution mass calcd for $C_{22}H_{26}O_3$: 338.1882, found m/z 338.1863. Anal. Calcd for $C_{22}H_{26}O_3$: C, 78.07; H, 7.74; N. Found: C, 78.13; H, 7.75.

Synthesis of 1-(4,4-dimethyl-1-phenylpent-1-yn-3-yl)-2,3,4-trumethoxybenzene (**2c**).

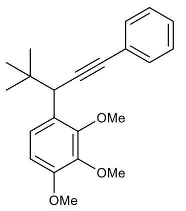

To a nitromethane (1.00 mL) solution of 4,4-dimethyl-1-phenylpent-1-yn-3-ol (**1c**) (50.0 mg, 0.266 mmol), 1,2,3-trimethoxybenzene (67.1 mg, 0.399 mmol), and tetrabutylammonium hexafluorophosphate (20.6 mg, 0.0532 mmol) were added silver perchlorate (27.6 mg, 0.133 mmol) and indium tribromide (18.9 mg, 0.0532 mmol) at room temperature. The reaction mixture was stirred for 0.5 h. The reaction mixture was filtered through a pad of celite with chloroform and then poured into a saturated sodium hydrogencarbonate. The organic layer was separated and the aqueous layer was extracted with chloroform. The combined organic layer was dried over $MgSO_4$. The solvent was removed under reduced pressure. The residue was purified by preparative TLC on silica gel eluting with AcOEt-*n*-hexane (1:40) to give a yellow oil. The titled compound was precipitated from *n*-hexane to give 1-(4,4-dimethyl-1-phenylpent-1-yn-3-yl)-2,3,4-trumethoxybenzene (**2c**) (76.8 mg, 73%) (from chloroform-*n*-hexane, white prisms mp 73-75 ºC) and (oxybis(4,4-dimethylpent-1-yne-3,1-diyl))dibenzene (**3c**) (12 mg, 11%) as white powders.

IR (KBr, cm^{-1}) δ 3451, 2964, 2932, 2854, 1715, 1599, 1492, 1463, 1416, 1363, 1284, 1256, 1220, 1097, 1030, 911, 806, 757, 691; ^{1}H NMR (600 MHz, $CDCl_3$) δ 1.03 (9H, s, CH_3×3), 3.85 (3H, s, OCH_3), 3.86 (3H, s, OCH_3), 3.91 (3H, s, OCH_3), 4.11 (1H, s, CH), 6.66-6.67 (1H, d, *J* = 8.9 Hz, ArH), 7.21-7.23 (1H, d, *J* = 8.3 Hz, ArH), 7.26-7.28 (3H, m, ArH), 7.43 (2H, brd, *J* = 7.6 Hz, ArH); ^{13}C NMR (150 MHz, $CDCl_3$) δ 27.5 (q×3), 36.0 (s), 55.9 (q), 60.6 (q), 60.9 (q), 82.2 (s), 92.4 (s), 106.5 (d×2), 124.1 (s), 124.8 (d), 125.5 (s), 127.4 (d), 128.1 (d×2), 131.5 (d×2), 141.5 (s), 151.7 (s), 152.4 (s); MS m/z 338 (M^+), 323 (M^+-Me), 308 (M^+-Me×2), 281 (M^+-t-Bu); high resolution mass calcd for $C_{22}H_{26}O_3$: 338.1882, found m/z 338.1906. Anal. Calcd for $C_{22}H_{26}O_3$: C, 78.07; H, 7.74. Found: C, 77.98; H, 7.98.

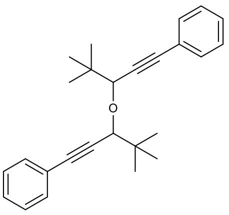

(oxybis(4,4-dimethylpent-1-yne-3,1-diyl))dibenzene (**3c**).

White powders, mp 58-59 °C, IR (KBr, cm^{-1}) ν 2956, 2866, 1735, 1696, 1490, 1314, 1243, 1193, 1061, 982, 757, 691; ^{1}H NMR (600 MHz, $CDCl_3$) δ 1.08(3H, s, CH_3), 4.28 (1H, s, CH), 7.30-7.31 (3H, m, ArH), 7.43-7.45 (2H, m, ArH); EIMS m/z 358 (small M^+), 343 (M^+-Me).

Preparation of 1-(1-cyclohexyl-3-phenylprop-2-yn-1-yl)-2,3,4-trimethoxybenzene (**2d**).

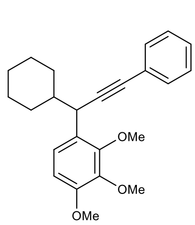

To a nitromethane (1.50 mL) solution of 1-cyclohexyl-3-phenylprop-2-yn-1-ol (**1d**) (50.0 mg, 0.250 mmol), 1,2,3-trimethoxybenzene (126 mg, 0.750 mmol), and tetrabutylammonium hexafluorophosphate (19.0 mg, 0.0500 mmol), 1,1'-binaphthol (14.3 mg, 0.0500 mmol) were added silver perchlorate (28.1 mg, 0.100 mmol) and indium trichloride (11.0 mg, 0.0500 mmol) at room temperature. The reaction mixture was stirred for 0.5 h. The reaction mixture was filtered through a pad of celite with chloroform and then poured into a saturated sodium hydrogencarbonate. The organic layer was separated and the aqueous layer was extracted with chloroform. The combined organic layer was dried over $MgSO_4$. The solvent was removed under reduced pressure. The residue was purified by preparative TLC on silica gel eluting with AcOEt-*n*-hexane (1:40) to give 1-(1-cyclohexyl-3-phenylprop-2-yn-1-yl)-2,3,4-trimethoxybenzene (**2d**) (79.3 mg, 87%) as a yellow oil and 5-(1-cyclohexyl-3-phenylprop-2-yn-1-yl)-1,2,3-trimethoxybenzene (**2d**) (3.2 mg, 4%) as a yellow oil.

IR (KBr, cm^{-1}) δ 3446, 2928, 2851, 1715, 1599, 1493, 1465, 1416, 1362, 1281, 1254, 1220, 1096, 1038, 1017, 757, 692, 529; ^{1}H NMR (600 MHz, $CDCl_3$) δ 1.18-1.21 (6H, m, CH_2), 1.63-1.65 (4H, brd, *J* = 9.6 Hz , CH_2), 1.71-1.74 (2H, m, CH_2), 1.91 (1H, brs, CH), 3.85 (3H, s, OCH_3), 3.88 (3H, s, OCH_3), 3.93 (3H, s, OCH_3), 4.00-4.02 (1H, d, *J* = 6.9 Hz, CH), 6.66-6.67 (1H, d, *J* = 8.2 Hz, ArH), 7.18-7.19 (1H, d, *J* = 9.0 Hz, ArH), 7.26-7.29 (3H, m, ArH), 7.43 (2H, dd, *J* = 2.0 and 7.5 Hz, ArH); ^{13}C NMR (150 MHz, $CDCl_3$) δ 26.3 (t), 26.4 (t×2), 29.6 (t), 31.7 (t), 38.0 (d), 43.5 (d), 55.9 (q), 60.7 (q), 61.0 (q), 82.9 (s), 91.6 (s), 106.9 (d), 123.5 (d), 124.1 (s), 126.9 (s), 127.5 (d), 128.1 (d×2), 131.6 (d×2), 141.9 (s), 151.2 (s), 152.3 (s); MS m/z 364 (M^+), 349 (M^+-Me), 333 (M^+-OMe), 281 (M^+-C_6H_{11}), 271 (M^+-OMe×3); high resolution mass calcd for $C_{24}H_{28}O_3$: 364.2038, found m/z 364.2048. Anal. Calcd for $C_{24}H_{28}O_3$: C, 79.09; H, 7.74. Found: C, 79.06; H, 7.83.

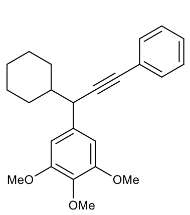

IR (KBr, cm^{-1}) δ 2926, 2852, 2359, 1593, 1507, 1457, 1419, 1332, 1236, 1129, 756, 691; ^{1}H NMR (600 MHz, $CDCl_3$) δ 1.16-1.25 (4H, m, CH_2), 1.64-1.68 (3H, m, CH_2), 1.75 (2H, brs, CH_2), 1.91 (1H, brs, CH), 3.61 (1H, d, *J* = 6.1 Hz, CH), 3.84 (3H, s, OMe), 3.88 (3H, s, OMe), 6.61 (2H, s, ArH), 7.29-7.32 (3H, m, ArH), 7.43-7.45 (2H, m, ArH); EIMS m/z 364 (M^+), 281 (M^+-cyclohexyl); high resolution mass calcd for C24H28O3: 364.2038, found m/z 364.2025.

Synthesis of 1-(1-cyclopentyl-3-phenylprop-2-yn-1-yl)-2,3,4-trimethoxybenzene (**2e**).

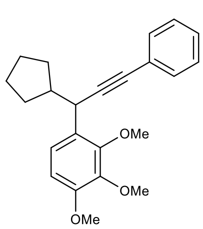

To a nitromethane (1.50 mL) solution of 1-cyclopentyl-3-phenylprop-2-yn-1-ol (**1e**) (50.0 mg, 0.250 mmol), 1,2,3-trimethoxybenzene (126 mg, 0.750 mmol), and tetrabutylammonium hexafluorophosphate (19.4 mg, 0.0500 mmol), 1,1'-binaphthol (14.3 mg, 0.0500 mmol) were added silver perchlorate (28.1 mg, 0.100 mmol) and indium tribromide (11.0 mg, 0.0500 mmol) at room temperature. The reaction mixture was stirred for 30 min. The reaction mixture was filtered through a pad of celite with chloroform and then poured into a saturated sodium hydrogencarbonate. The organic layer was separated and the aqueous layer was extracted with chloroform. The combined organic layer was dried over $MgSO_4$. The solvent was removed under reduced pressure. The residue was purified by preparative TLC on silica gel eluting with AcOEt-*n*-hexane (1:40) to give 1-(1-cyclopentyl-3-phenylprop-2-yn-1-yl)-2,3,4-trimethoxybenzene (**2e**) (60.9 mg, 70%) as a yellow oil, 5-(1-cyclopentyl-3-phenylprop-2-yn-1-yl)-1,2,3-trimethoxybenzene (**2e**) (4.7 mg, 5%) as a yellow oil.IR (KBr, cm^{-1}) ν 3459, 2953, 2866, 1714, 1599, 1493, 1465, 1284, 1096, 757, 692; ^{1}H NMR (600 MHz, $CDCl_3$) δ 1.41-1.43 (1H, m, CH_2), 1.52-1.55 (2H, m, CH_2), 1.62-1.69 (2H, m, CH_2), 1.76 (1H, m, CH_2), 2.23 (1H, sex, *J* = 7.6 Hz, CH), 3.85 (3H, s, OCH_3), 3.88 (3H, s, OCH_3), 3.94 (3H, s, OCH_3), 4.13 (1H, d, *J* = 6.8 Hz, CH), 6.66 (1H, d, *J* = 8.9 Hz, ArH), 7.21 (1H, d, *J* = 8.9 Hz, ArH), 7.25-7.29 (3H, m, ArH), 7.42 (2H, dd, *J* = 2.1 and 7.6 Hz, ArH); ^{13}C NMR (150 MHz, $CDCl_3$) δ 25.3 (t×2), 29.9 (t), 31.0 (t), 36.0 (d), 46.0 (d), 55.9 (q), 60.7 (q), 61.1 (q), 82.1 (s), 92.0 (s), 107.1 (d), 123.0 (d), 124.1 (s), 127.5 (d), 128.0 (s), 128.1 (d×2), 131.6 (d×2), 141.9 (s), 151.0 (s), 152.3 (s); MS m/z 350 (M^+), 335 (M^+-Me), 319 (M^+-OMe), 281 (M^+-C_5H_9);

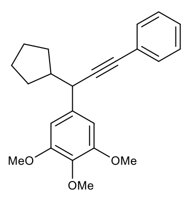

high resolution mass calcd for $C_{23}H_{26}O_3$: 350.1882, found m/z 350.1863. Anal. Calcd for $C_{23}H_{26}O_3$(+3/5H_2O): C, 76.47; H, 7.59. Found: C, 76.42; H, 7.59.

5-(1-cyclopentyl-3-phenylprop-2-yn-1-yl)-1,2,3-trimethoxybenzene (**2e**).

IR (KBr, cm^{-1}) ν 2954, 2931, 1857, 1360, 1593, 1507, 1458, 1420, 1331, 1237, 1128, 757, 692; ^{1}H NMR (600 MHz, $CDCl_3$) δ 1.41-1.47 (1H, m, CH), 1.54-1.70 (6H, m, CH), 1.78-1.84 (1H, m, CH), 2.24 (1H, sex, *J* = 7.6 Hz, CH), 3.70 (1H, d, *J* = 7.6 Hz, CH), 3.84 (3H, s, OMe), 3,87 (3H, s, OMe), 6.64 (2H, s, ArH), 7.29-7.32 (3H, m, ArH), 7.42-7.44 (2H, m, ArH); EIMS m/z 350 (M^+), 281 (M^+-cyclopentyl); high resolution mass calcd for $C_{23}H_{26}O_3$: 350.1882, found m/z 350.1878.

Synthesis of (3-phenyl-1-(2,3,4-trimethoxyphenyl)prop-2-yn-1-yl)cyclooctane (**2f**).

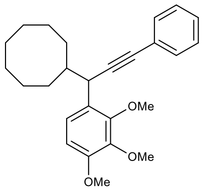

To a nitromethane (1.50 mL) solution of 1-cyclooctyl-3-phenylprop-2-yn-1-ol (**1f**) (75.6 mg, 0.312 mmol), 1,2,3-trimethoxybenzene (157 mg, 0.936 mmol), and tetrabutylammonium hexafluorophosphate (24.2 mg, 0.0624 mmol), 1,1-binaphthol (18.0 mg, 0.0624 mmol) were added silver perchlorate (35.0 mg, 0.0125 mmol) and indium trichloride (13.8 mg, 0.0125 mmol) at room temperature. The reaction mixture was stirred for 30 min. The reaction mixture was filtered through a pad of celite with chloroform and then poured into a saturated sodium hydrogencarbonate. The organic layer was separated and the aqueous layer was extracted with chloroform. The combined organic layer was dried over $MgSO_4$. The solvent was removed under reduced pressure. The residue was purified by preparative TLC on silica gel eluting with AcOEt-*n*-hexane (1:40) to give (3-phenyl-1-(2,3,4-trimethoxyphenyl)prop-2-yn-1-yl)cyclooctane (**2f**) (61.7 mg, 50%) as a yellow oil, (3-phenylprop-2-yn-1-ylidene)cyclooctane (**3f**) (17.3 mg, 25%) as a yellow oil.

IR (KBr, cm^{-1}) δ 3422, 2924, 2848, 1715, 1599, 1492, 1465, 1416, 1278, 1097, 1018, 799, 692; ^{1}H NMR (600 MHz, $CDCl_3$) δ 1.35-1.37 (1H, m, CH_2), 1.48-1.59 (9H, m, CH_2), 1.62-1.66 (1H, m, CH_2), 1.70-1.71 (2H, m, CH_2), 1.80-1.83 (1H, m, CH_2), 1.95-1.98 (1H, m, CH), 3.85 (3H, s, OCH_3), 3.87 (3H, s, OCH_3), 3.94 (3H, s, OCH_3), 4.05 (1H, d, *J* = 6.9 Hz, CH), 6.66 (1H, d, *J* = 8.9 Hz, ArH), 7.21 (1H, d, *J* = 8.3 Hz, ArH), 7.26-7.30 (3H, m, ArH), 7.42 (2H, dd, *J* = 2.0 and 5.5 Hz, ArH); ^{13}C NMR (150 MHz, $CDCl_3$) δ 25.8 (t), 26.0 (t), 26.6 (t), 26.8 (t), 27.0 (t), 28.8 (t), 32.2 (t), 39.1 (d), 42.6 (d), 55.9 (q), 60.7 (q), 61.0 (q), 82.8 (s), 91.8 (s), 106.9 (d), 123.6 (d), 124.1 (s), 127.4 (s), 127.5 (d), 128.1 (d×2), 131.6 (d×2), 141.9 (s), 151.2 (s), 152.4 (s); MS m/z 392 (M^+), 281 (M^+-C_8H_{15}); high resolution mass calcd for $C_{26}H_{32}O_3$: 392.2351, found m/z 392.2326; Anal. Calcd for $C_{26}H_{32}O_3$+(1/4H_2O): C, 78.65; H, 8.25. Found: C, 78.19; H, 7.97.

(3-phenylprop-2-yn-1-ylidene)cyclooctane (**4f**).

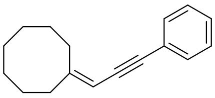

IR (KBr, cm^{-1}) δ 3438, 2851, 1715, 1598, 1490, 1468, 1443, 1360, 1219, 756, 691, 527; ^{1}H NMR (600 MHz, $CDCl_3$) δ 1.50 (6H, brs, CH_2), 1.61 (2H, brs, CH_2), 2.13-2.14 (2H, m, CH_2), 2.27 (2H, t, *J* = 6.2 Hz, CH_2), 3.13 (2H, s, CH_2), 5.71 (1H, t, *J* = 8.2 Hz, CH), 7.26-7.28 (3H, m, ArH), 7.41-7.42 (2H, m, ArH); MS m/z 225 (M^+), 209(M^+-Me); high resolution mass calcd for $C_{17}H_{20}$: 224.1565, found m/z 224.1586.

Synthesis of (3-phenyl-1-(2,3,4--trimethoxyphenyl)prop-2-yn-1-yl)cyclododecane (**2g**).

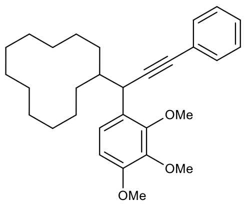

To a nitromethane (1.00 mL) solution of 1-cyclododecyl-3-phenylprop-2-yn-1-ol (**1g**) (74.6 mg, 0.250 mmol), 1,2,3-trimethoxybenzene (126 mg, 0.750 mmol), tetrabutylammonium hexafluorophosphate (19.0 mg, 0.0500 mmol), 1,1'-binaphthol (14.3 mg, 0.0500 mmol) were added silver perchlorate (28.1 mg, 0.100 mmol) and indium trichloride (11.0 mg, 0.0500 mmol) at room temperature. The reaction mixture was stirred for 0.5 h. The reaction mixture was filtered through a pad of celite with chloroform and then poured into a saturated sodium hydrogencarbonate. The organic layer was separated and the aqueous layer was extracted with chloroform. The combined organic layer was dried over $MgSO_4$. The solvent was removed under reduced pressure. The residue was purified by preparative TLC on silica gel eluting with AcOEt-n-hexane (1:50) to give (3-phenyl-1-(2,3,4-trimethoxyphenyl)prop-2-yn-1-yl)cyclododecane (**2g**) (109 mg, 97%) as a yellow oil, (3-phenyl-1-(3,4,5-trimethoxyphenyl)prop-2-yn-1-yl)cyclododecane (**2g**) (2 mg, 2%) as a yellow oil.

IR (KBr, cm^{-1}) δ 3448, 2932, 2850, 1715, 1599, 1493, 1467, 1416, 1278, 1255, 1255, 1098, 1019, 757, 692; ^{1}H NMR (600 MHz, $CDCl_3$) δ 1.11 (1H, brs, CH_2), 1.26-1.44 (18H, m, CH_2), 1.49-1.54 (3H, m, CH_2), 1.95 (1H, brs, CH), 3.85 (3H, s, OCH_3), 3.86 (3H, s, OCH_3), 3.94 (3H, s, OCH_3), 4.21 (1H, d, *J* = 5.5 Hz, CH), 6.65 (1H, d, *J* = 9.0 Hz, ArH), 7.25 (1H, d, *J* = 8.9 Hz, ArH), 7.27-7.30 (3H, m, ArH), 7.43 (2H, dd, *J* = 7.5 and 1.4 Hz, ArH); ^{13}C NMR (150 MHz, $CDCl_3$) δ 21.9 (t), 23.0 (t×3), 23.2 (t), 24.0 (t), 24.1 (t), 24.6 (t), 24.7 (t), 26.7 (t), 27.6 (t), 35.9 (d), 38.5 (d), 55.9 (q), 60.7 (q), 60.9 (q), 83.3 (s), 91.3 (s), 106.7 (d), 123.8 (d), 124.1 (s), 127.0 (s), 127.5 (d), 128.1 (d×2), 131.6 (d×2), 141.9 (s), 151.1 (s), 152.4 (s); MS m/z 448 (M^+), 281 (M^+-$C_{12}H_{23}$); high resolution mass calcd for $C_{30}H_{40}O_3$: 448.2977, found m/z 448.2982. Anal. Calcd for $C_{30}H_{40}O_3$: C, 80.31; H, 8.99. Found: C, 80.46; H, 9.02.

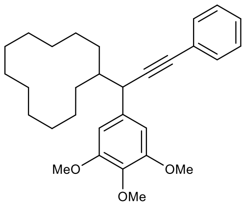

IR (KBr, cm^{-1}) δ 3459, 2933, 2850, 1592, 1507, 1491, 1468, 1419, 1333, 1235, 1129, 1011, 757, 691;

^{1}H NMR (400 MHz, $CDCl_3$) δ 1.22-1.54 (21H, m, CH_2), 1.80-1.91 (2H, m, CH_2), 3.77 (1H, d, J = 6.4 Hz, CH), 3.85 (3H, s, OCH_3), 3.88 (6H, s, OCH_3×2), 6.64 (2H, s, ArH), 7.29-7.32 (3H, m, ArH), 7.42-7.45 (2H, m, ArH); MS m/z 448 (M^+); high resolution mass calcd for $C_{30}H_{40}O_3$: 448.2977, found m/z 448.2982.

Synthesis of 1,2,3-trimethoxy-4-(1-(*p*-tolyl)oct-1-yn-3-yl)benzene (**2h**).

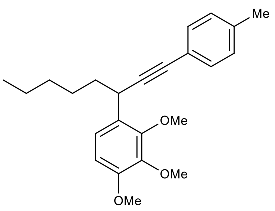

To a nitromethane (1.00 mL) solution of 1-(*p*-tolyl)oct-1-yn-3-ol (**1h**) (50.0 mg, 0.231 mmol), 1,2,3-trimethoxybenzene (77.7 mg, 0.462 mmol), tetrabutylammonium hexafluorophosphate (17.9 mg, 0.0462 mmol), and (R)-1,1'-binaphthol (13.2 mg, 0.0462 mmol) were added silver perchlorate (19.2 mg, $9.24x10^{-5}$ mmol) and indium trichloride (10.2 mg, 0.0462 mmol) at room temperature. The reaction mixture was stirred for 30 min. The reaction mixture was poured into water (50.0 mL). The organic layer was separated and the aqueous layer was extracted with chloroform. The combined organic layer was dried over $MgSO_4$. The solvent was removed under reduced pressure. The residue was purified by preparative TLC on silica gel eluting with AcOEt-*n*-hexane (1:50) to give 1,2,3-trimethoxy-4-(1-(*p*-tolyl)oct-1-yn-3-yl)benzene (**2h**) (55.5 mg, 66%) as a yellow oil and 1,2,3-trimethoxy-5-(1-(p-tolyl)oct-1-yn-3-yl)benzene (**2h**) (4.80 mg, 6%) as a yellow oil.

IR (KBr, cm^{-1}) δ 3444, 2930, 2856, 1715, 1601, 1509, 1494, 1465, 1416, 1362, 1278, 1220, 1095, 1043, 1018, 818, 529; ^{1}H NMR (600 MHz, $CDCl_3$) δ 0.89 (3H, t, J = 6.9Hz, CH_3), 1.32-1.33 (4H, m, CH_2×2), 1.46-1.55 (2H, m, CH_2), 1.72-1.76 (2H, m, CH_2), 2.33 (3H, s, CH_3), 3.85 (3H, s, OCH_3), 3.87 (3H, s, OCH_3), 3.93 (3H, s, OCH_3), 4.13 (1H, dd, J = 6.9 and 7.6 Hz, CH), 6.67-6.68 (1H, d, J = 8.2 Hz, ArH), 7.08-7.09 (2H, d, J = 7.6 Hz, ArH), 7.22-7.23 (1H, d, J = 8.3 Hz, ArH), 7.31-7.32 (2H, d, J = 7.6 Hz, ArH); ^{13}C NMR (150 MHz, $CDCl_3$) δ 14.0 (q), 21.4 (q), 22.5 (t), 27.3 (t), 31.5 (t), 31.6 (d), 37.6 (t), 56.0 (q), 60.7 (q), 61.1 (q), 82.1 (s), 91.7 (s), 107.3 (d), 120.9 (s), 122.6 (d), 128.6 (s), 128.9 (d×2), 131.4 (d×2), 137.5 (s), 142.0 (s), 150.9 (s), 152.4 (s); MS m/z 366 (M^+), 351 (M^+-Me), 295 (M^+-C_5H_9); high resolution mass calcd for $C_{24}H_{30}O_3$: 366.2195, found m/z 366.2194. Anal. Calcd for $C_{24}H_{30}O_3$: C, 78.68; H, 8.25. Found: C, 78.74; H, 8.43.

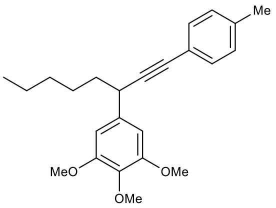

IR (KBr, cm^{-1}) δ 2930, 2855, 2257, 1715, 1592, 1509, 1460, 1420, 1335, 1235, 1129, 818, 756; ^{1}H NMR (600 MHz, $CDCl_3$) δ 0.90 (3H, t, J = 6.9 Hz, Me), 1.32 (4H, brs, CH_2x2), 1.43-1.52 (2H, m, CH_2), 1.77-1.80 (2H, m, CH_2), 2.34 (3H, s, Me), 3.75 (1H, brt, J = 6.3 Hz, CH), 3.84 (3H, s, OMe), 3.88 (6H, s, OMex2), 6.64 (2H, s, ArH), 7.10 (2H, d, J = 8.0 Hz, ArH), 7.32 (2H, d, J = 8.0 Hz, ArH); EIMS m/z 366 (M^+).

1,2,3-trimethoxy-4-(1-(*m*-tolyl)but-1-yn-3-yl)benzene (**2i**).

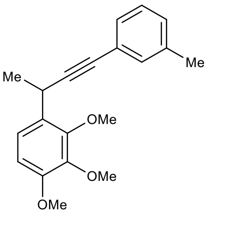

To a nitromethane (1.50 mL) solution of 1-(*m*-tolyl)but-1-yn-3-ol (**1i**) (50.0 mg, 0.312 mmol), 1,2,3-trimethoxybenzene (158 mg, 0.936 mmol), tetrabutylammonium hexafluorophosphate (24.2 mg, 0.0624 mmol), and (R)-1,1'-binaphthol (17.9 mg, 0.0624 mmol) were added silver perchlorate (25.9 mg, 0.125 mmol) and indium trichloride (13.8 mg, 0.0624 mmol) at room temperature. The reaction mixture was stirred for 0.5 h. The reaction mixture was poured into water (50.0 mL). The organic layer was separated and the aqueous layer was extracted with chloroform. The combined organic layer was dried over $MgSO_4$. The solvent was removed under reduced pressure. The residue was purified by preparative TLC on silica gel eluting with AcOEt-*n*-hexane (1:50) to give 1,2,3-trimethoxy-4-(1-(*m*-tolyl)but-1-yn-3-yl)benzene (**2i**) (53.7 mg, 55%) as a yellow oil and 1,2,3-trimethoxy-5-(1-(*m*-tolyl)but-1-yn-3-yl)benzene (**2i**) (9.0 mg, 9%) as a yellow oil.

IR (KBr, cm^{-1}) 2968, 2933, 2833, 2230, 1601, 14994, 1281, 1258, 1017, 801, 692; δ 1.51 (3H, d, J = 6.3 Hz, Me), 2.31 (3H, s, Me), 3.85 (3H, s, OMe), 3.87 (3H, d, J = 1.2 Hz, OMe), 3.91 (3H, s, OMe), 3.95 (3H, s, OMe), 4.26 (1H, dd, J = 6.9 and 13.8 Hz, CH), 6.68 (1H, d, J = 8.6 Hz, ArH), 7.08 (1H, d, J = 7.5 Hz, ArH), 7.17 (1H, t, J = 7.5 Hz, ArH), 7.23 (1H, d, J = 8.0 Hz, ArH), 7.26 (1H, brs, ArH), 7.27 (1H, d, J = 8.6 Hz, ArH); ^{13}C NMR (150 MHz, $CDCl_3$) δ 21.2 (q), 23.7 (q), 26.1 (d), 56.0 (q), 60.1 (q), 61.1 (q), 81.5 (s), 93.0 (s), 107.3 (d), 121.9 (d), 123.6 (s), 128.0 (d), 128.5 (d), 128.6 (d), 129.4 (s), 132.2 (d), 137.8 (s), 1342.0 (s), 150.8 (s), 152.5 (s); EIMS m/z 310 (M^+); high resolution mass calcd for $C_{20}H_{22}O_3$: 310.1569, found m/z 310.1569.

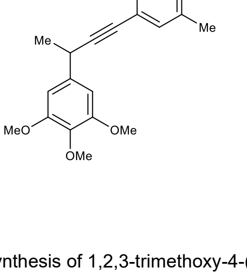

1,2,3-trimethoxy-5-(1-(*m*-tolyl)but-1-yn-3-yl)benzene (**2i**)

IR (KBr, cm^{-1}) δ 2971, 2937, 2838, 2362, 1714, 1683, 1591, 1507, 1459, 1417, 1331, 1235, 1128, 1008, 782, 756; ^{1}H NMR (500 MHz, $CDCl_3$) δ 1.57 (3H, t, J = 7.4 Hz, CH_3), 2.32 (3H, s, Me), 3.84 (3H, s, OMe), 3.89 (6H, s, OMex2), 3.93 (1H, dd, J = 6.9 and 5.2 Hz, CH), 7.11 (1H, d, J = 7.4 Hz, ArH), 7.19 (1H, t, J = 7.5 Hz, ArH), 7.24 (2H, t, J = 7.5 Hz, ArH); EIMS m/z 310 (M^+), 295 (M^+ -Me); high resolution mass calcd for $C_{20}H_{22}O_3$: 310.1569, found m/z 310.1569.

Synthesis of 1,2,3-trimethoxy-4-(1-(*o*-tolyl)but-1-yn-3-yl)benzene (**2j**).

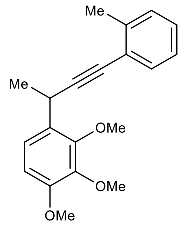

To a nitromethane (1.50 mL) solution of 1-(*o*-tolyl)but-1-yn-3-ol (**1j**) (50.0 mg, 0.312 mmol), 1,2,3-trimethoxybenzene (158 mg, 0.936 mmol), tetrabutylammonium hexafluorophosphate (24.2 mg, 0.0624 mmol), and (R)-1,1'-binaphthol (17.9 mg, 0.0624 mmol) were added silver perchlorate (25.9 mg, 0.125 mmol) and indium trichloride (13.8 mg, 0.0624 mmol) at room temperature. The reaction mixture was stirred for 2.5 h. The reaction mixture was poured into water (50.0 mL). The organic layer was separated and the aqueous layer was extracted with chloroform. The combined organic layer was dried over $MgSO_4$. The solvent was removed under reduced pressure. The residue was purified by preparative TLC on silica gel eluting with AcOEt-*n*-hexane (1:50) to give 1,2,3-trimethoxy-4-(1-(*o*-tolyl)but-1-yn-3-yl)benzene (**2j**) (59.3 mg, 61%) as a yellow oil and 1,2,3-trimethoxy-5-(1-(*o*-tolyl)but-1-yn-3-yl)benzene (**2j**) (3.2 mg, 3%) as a yellow oil.

IR (KBr, cm^{-1}) δ 2972, 2934, 2833, 2227, 1600, 1494, 1466, 1416, 1283, 1255, 1099, 1030, 912, 801, 759; ^1H NMR (600 MHz, $CDCl_3$) δ 1.45 (3H, d, *J* = 6.9 Hz, Me), 2.36 (3H, s, Me), 3.76 (3H, s, OMe), 3.80 (3H, s, OMe), 3.87 (3H, s, OMe), 4.22-4.26 (1H, m, CH), 6.59 (1H, d, *J* = 8.6 Hz, ArH), 7.02 (1H, brs. ArH), 7.09 (2H, brs, ArH), 7.21 (1H, d, *J* = 8.6 Hz, ArH), 7.31 (1H, d, *J* = 7.4 Hz, ArH); ^{13}C NMR (150 MHz, $CDCl_3$) δ 20.8 (q), 23.9 (q), 26.2 (d), 55.9 (q), 60.7 (q), 61.1 (q), 80.4 (s), 97.4 (s), 107.3 (d), 121.9 (d), 123.6 (s), 125.4 (d), 127.6 (d), 129.2 (d), 129.4 (s), 131.7 (d), 140.0 (s), 142.0 (s), 150.7 (s), 152.5 (s); EIMS m/z 310 (M^+); high resolution mass calcd for $C_{20}H_{22}O_3$: 310.1569, found m/z 310.1569. Anal. Calcd for $C_{20}H_{22}O_3(+1/4H_2O)$: C, 76.29; H, 7.20. Found: C, 76.13; H, 7.00.

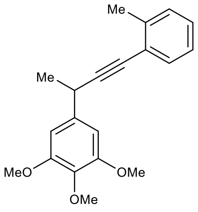

IR (KBr, cm^{-1}) δ 2934, 2837, 2359, 2232, 1592, 1508, 1458, 1419, 1333, 1234, 1181, 1129, 759; ^1H NMR (600 MHz, $CDCl_3$) δ 1.53 (3H, d, *J* = 6.9 Hz, Me), 2.39 (3H, s, Me), 3.76 (3H, s, OMe), 3.81 (3H, s, OMe), 3.90 (1H, ABq, *J* = 6.8 Hz, CH), 6.63 (2H, s, ArH), 7.04-7.08 (1H, m, ArH), 7.13 (2H, brd, *J* = 4.0 Hz, ArH), 7.33 (1H, d, *J* = 7.4 Hz, ArH); EIMS m/z 310 (M^+); high resolution mass calcd for $C_{20}H_{22}O_3$: 310.1569, found m/z 310.1569.

Synthesis of 1,2,3-trimethoxy-4-(1-(*p*-methoxyphenyl)but-1-yn-3-yl)benzene (**2k**).

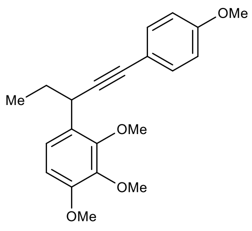

To a nitromethane (1.30 mL) solution of 1-(*p*-methoxyphenyl)pent-1-yn-3-ol (**1k**) (50.0 mg, 0.263 mmol), 1,2,3-trimethoxybenzene (133 mg, 0.788 mmol), tetrabutylammonium hexafluorophosphate (20.4 mg, 0.0526 mmol), and (R)-1,1'-binaphthol (15.1 mg, 0.0526 mmol) were added silver perchlorate (21.8 mg, 0.105 mmol) and indium trichloride (11.6 mg, 0.0526 mmol) at room temperature. The reaction mixture was stirred for 1 h. The reaction mixture was poured into water (50.0 mL). The organic layer was separated and the aqueous layer was extracted with chloroform. The combined organic layer was dried over $MgSO_4$. The solvent was removed under reduced pressure. The residue was purified by preparative TLC on silica gel eluting with AcOEt-*n*-hexane (1:50) to give 1,2,3-trimethoxy-4-(1-(p-methoxyphenyl)but-1-yn-3-yl)benzene (**2k**) (38.4 mg, 45%) as a yellow oil and 1,2,3-trimethoxy-5-(1-(*p*-methoxyphenyl)but-1-yn-3-yl)benzene (**2k**) (2.8 mg, 3%) as a yellow oil.

IR (KBr, cm^{-1}) δ 2962, 2934, 2871, 2232, 1606, 1509, 1494, 1465, 1416, 1288, 1248, 1173, 1095, 1032, 833, 801; ^1H NMR (600 MHz, $CDCl_3$) δ 0.98 (3H, t, *J* = 7.4 Hz, Me), 1.65-1.76 (2H, m, CH_2), 3.72 (3H, brs, OMe), 3.78 (3H, s, OMe), 3.80 (3H, s, OMe), 3.86 (3H, d, *J* = 1.1 Hz, OMe), 4.01 (1H, dd, *J* = 4.8 and 8.0 Hz, CH), 6.60 (1H, d, *J* = 8.6 Hz, ArH), 6.74 (2H, d, *J* = 8.5 Hz, ArH), 7.15 (1H, d, *J* = 8.6 Hz, ArH), 7.29 (2H, d, *J* = 8.0 Hz, ArH); ^{13}C NMR (150 MHz, $CDCl_3$) δ 12.4 (q), 30.7 (t), 33.2 (d), 55.2 (q), 55.9 (q), 60.7 (q), 61.1 (q), 82.0 (s), 90.6 (s), 107.1 (d), 113.7 (dx2), 116.1 (s), 122.7 (d), 128.2 (s), 132.9 (dx2), 141.9 (s), 150.9 (s), 152.4 (s), 159.0 (s); EIMS m/z 340 (M^+), 325 (M^+-Me), 311 (M^+-MeO); high resolution mass calcd for $C_{21}H_{24}O_4$: 340.1675, found m/z 340.1663. Anal. Calcd for $C_{21}H_{24}O_4(+1/3H_2O)$: C, 72.81; 7.18. Found: C, 72.58; H, 7.04.

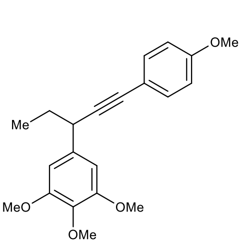

Synthesis of 1,2,3-trimethoxy-5-(1-(*p*-methoxyphenyl)but-1-yn-3-yl)benzene (**2k**)

IR (KBr, cm^{-1}) δ 2963, 2936, 2836, 2227, 13, 1507, 1462, 1420, 1339, 1286, 1237, 1129, 1045, 1009, 788, 687; ^1H NMR (600 MHz, $CDCl_3$) δ 1.00 (3H, t, *J* = 7.4 Hz, Me), 1.73-1.81 (2H, m, CH_2), 1.82 (1H, dd, *J* = 6.3 and 8.1 Hz, CH), 3.74 (3H, s, OMe), 3.77 (3H, s, Ome), 3.81 (6H, s, Omex2), 6.58 (2H, s, ArH), 6.76 (2H, d, *J* = 8.6 Hz, ArH), 7.30 (2H, d, *J* = 8.6 Hz, ArH); EIMS m/z 340 (M^+); high resolution mass calcd for $C_{21}H_{24}O_3$: 340.1675, found m/z 340.1663.

Synthesis of 1,2,3-trimethoxy-4-(1-(3-methoxyphenyl)but-1-yn-3-yl)benzene (**2l**).

To a nitromethane (1.30 mL) solution of 1-(m-methoxyphenyl)pent-1-yn-3-ol (**1l**) (50.0 mg, 0.263 mmol), 1,2,3-trimethoxybenzene (133 mg, 0.788 mmol), tetrabutylammonium hexafluorophosphate (20.4 mg, 0.0526 mmol), and (R)-1,1'-binaphthol (15.1 mg, 0.0526 mmol) were added silver perchlorate (21.8 mg, 0.105 mmol) and indium trichloride (11.6 mg, 0.0526 mmol) at room temperature. The reaction mixture was stirred for 2 h. The reaction mixture was poured into water (50.0 mL). The organic layer was separated and the aqueous layer was extracted with chloroform. The combined organic layer was

dried over $MgSO_4$. The solvent was removed under reduced pressure. The residue was purified by preparative TLC on silica gel eluting with AcOEt-*n*-hexane (1:50) to give 1,2,3-trimethoxy-4-(1-(*m*-methoxyphenyl)but-1-yn-3-yl)benzene (**2l**) (48.7 mg, 54%) as a yellow oil and 1,2,3-trimethoxy-5-(1-(*m*-methoxyphenyl)but-1-yn-3-yl)benzene (**2l**) (3.6 mg, 4%) as a yellow oil.

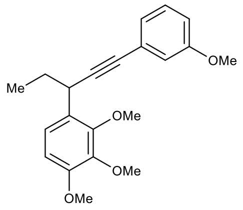

IR (KBr, cm^{-1}) δ 2965, 2936, 2226, 1599, 1465, 1417, 1284, 1202, 1096, 1039, 787, 688; ^{1}H NMR (600 MHz, $CDCl_3$) δ 1.07 (3H, t, *J* = 7.5 Hz, Me), 1.60-1.85 (2H, m, CH_2), 3.79 (3H, s, OMe), 3.85 (3H, s, OMe), 3.88 (3H, s, OMe), 3.94 (3H, s, OMe), 4.09 (1H, dd, *J* = 5.7 and 8.0 Hz, CH), 6.68 (1H, s, ArH), 6.83 (1H, dd, *J* = 2.9 and 8.6 Hz, ArH), 6.96 (1H, s, ArH), 7.03 (1H, d, *J* = 7.5 Hz, ArH), 7.19 (1H, t, *J* = 8.6 Hz, ArH), 7.22 (1H, d, *J* = 8.6 Hz, ArH); ^{13}C NMR (150 MHz, $CDCl_3$) δ 12.0 (q), 30.7 (t), 33.2 (d), 55.2 (q), 56.0 (q), 60.7 (q), 61.1 (q), 82.0 (s), 90.6 (s), 107.2 (d), 113.8 (dx2), 116.1 (s), 122.7 (d), 128.3 (s), 132.9 (dx2), 141.9 (s), 150.9 (s), 152.4 (s), 159.0 (s); EIMS m/z 340 (M^+); high resolution mass calcd for $C_{21}H_{24}O_4$: 340.1675, found m/z 340.1663. Anal. Calcd for $C_{21}H_{24}O_4$: C, 74.09; H, 4.24. Found: C, 73.87; H, 7.09.

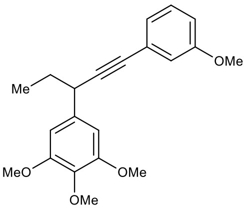

1,2,3-trimethoxy-5-(1-(*m*-methoxyphenyl)but-1-yn-3-yl)benzene (**2l**).

IR (KBr, cm^{-1}) δ 2963, 2936, 2836, 2227, 1593, 1507, 1462, 1420, 1339, 1286, 1237, 1129, 1045, 1010, 788, 687; ^{1}H NMR (600 MHz, $CDCl_3$) δ 1.07 (3H, t, *J* = 7.4 Hz, Me), 1.82-1.89 (2H, m, CH_2), 3.71 (1H, t, *J* = 6.9 Hz, CH), 3.80 (3H, s, OMe), 3.87 (3H, s, OMe), 3.88 (6H, s, OMex2), 6.65 (2H, s, ArH), 6.86 (1H, dd, *J* = 2.9 and 8.6 Hz, ArH), 6.97 (1H, brs, ArH), 7.04 (1H, d, *J* = 8.0 Hz, ArH), 7.21 (1H, t, *J* = 8.0 Hz, ArH); high resolution mass calcd for $C_{21}H_{24}O_4$: 340.1675, found m/z 340.1663.

Synthesis of 1,2,3-trimethoxy-4-(1-(*o*-methoxyphenyl)but-1-yn-3-yl)benzene (**2m**)

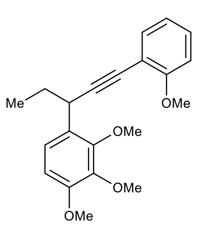

To a nitromethane (1.30 mL) solution of 1-(*o*-methoxyphenyl)pent-1-yn-3-ol (**1m**) (50.0 mg, 0.263 mmol), 1,2,3-trimethoxybenzene (133 mg, 0.788 mmol), tetrabutylammonium hexafluorophosphate (20.4 mg, 0.0526 mmol), and (R)-1,1'-binaphthol (15.1 mg, 0.0526 mmol) were added silver perchlorate (21.8 mg, 0.105 mmol) and indium trichloride (11.6 mg, 0.0526 mmol) at room temperature. The reaction mixture was stirred for 5 h. The reaction mixture was poured into water (50.0 mL). The organic layer was separated and the aqueous layer was extracted with chloroform. The combined organic layer was dried over $MgSO_4$. The solvent was removed under reduced pressure. The residue was purified by preparative TLC on silica gel eluting with AcOEt-*n*-hexane (1:50) to give 1,2,3-trimethoxy-4-(1-(*o*-methoxyphenyl)but-1-yn-3-yl)benzene (**2m**) (43.1 mg, 48%) as a yellow oil and 1,2,3-trimethoxy-5-(1-(*o*-methoxyphenyl)but-1-yn-3-yl)benzene (**2m**) (4.0 mg, 4%) as a yellow oil.

IR (KBr, cm^{-1}) δ 2964, 2935, 2835, 2229, 1597, 1494, 1465, 1416, 1257, 1096, 1025, 908, 801, 754; ^{1}H NMR (600 MHz, $CDCl_3$) δ 1.09 (3H, t, *J* = 7.5 Hz, Me), 1.76-1.86 (2H, m, CH_2), 3.85 (3H, s, OMe), 3.87 (3H, s, OMe), 3.88 (3H, s, OMe), 3.93 (3H, d, *J* = 1.1 Hz, MeO), 4.16 (1H, dd, *J* = 5.8 and 8.0 Hz, CH), 6.67 (1H, d, *J* = 8.6 Hz, ArH), 6.85-6.90 (2H, m, ArH), 7.23 (1H, d, *J* = 8.0 Hz, ArH), 7.31 (1H, d, *J* = 8.6 Hz, ArH), 7.41 (1H, d, *J* = 7.4 Hz, ArH); ^{13}C NMR (150 MHz, $CDCl_3$) δ 11.9 (q), 30.7 (t), 33.6 (d), 55.7 (q), 55.9 (q), 60.7 (q), 61.1 (q), 78.6 (s), 96.4 (s), 107.1 (d), 110.6 (d), 113.1 (s), 120.3 (d), 122.9 (d), 128.2 (s), 128.9 (d), 133.4 (d), 141.9 (s), 150.9 (s), 152.3 (s), 160.0 (s); EIMS m/z 340 (M^+), 311 (M^+-OMe); high resolution mass calcd for $C_{21}H_{24}O_4$: 340.1675, found m/z 340.1663. Anal. Calcd for $C_{21}H_{24}O_4$(+1/2H_2O): C, 72.18; H, 7.21. Found: C, 72.42; H, 7.08.

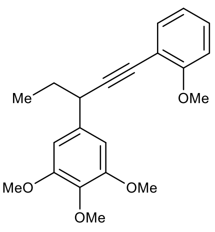

Synthesis of 1,2,3-trimethoxy-5-(1-(*o*-methoxyphenyl)but-1-yn-3-yl)benzene (**2m**)

IR (KBr, cm^{-1}) ν 2963, 2935, 2230, 1593, 1494, 1463, 1420, 1338, 1238, 1128, 1009, 754; ^{1}H NMR (600 MHz, $CDCl_3$) δ 1.10 (3H, t, *J* = 7.4 Hz, Me), 1.81-1.92 (2H, m, CH_2), 3.79-3.82 (1H, m, CH), 3.84 (3H, s, OMe), 3.87 (3H, s, OMe), 3.89 (6H, s, OMex2), 6.71 (2H, s, ArH), 6.87-6.92 (2H, m, ArH), 7.25-7.29 (1H, m, ArH), 7.41 (1H, dd, *J* = 1.8 and 7.8 Hz, ArH); EIMS m/z 340 (M^+); high resolution mass calcd for $C_{21}H_{24}O_4$: 340.1675, found m/z 340.1663.

Synthesis of 1,2,3-trimethoxy-4-(1-(2,4,6-trimethylphenyl)but-1-yn-3-yl)benzene (**2n**).

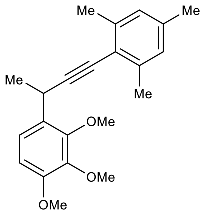

To a nitromethane (1.20 mL) solution of 1-(2,4,6-trimethylphenyl)pent-1-yn-3-ol (**1n**) (50.0 mg, 0.247 mmol), 1,2,3-trimethoxybenzene (125 mg, 0.741 mmol), tetrabutylammonium hexafluorophosphate (19.1 mg, 0.0494 mmol), and (R)-1,1'-binaphthol (14.1 mg, 0.0494 mmol) were added silver perchlorate (20.5 mg, 0.0988 mmol) and indium trichloride (10.9 mg, 0.0494 mmol) at room temperature. The reaction mixture was stirred for 0.5 h. The reaction mixture was poured into water (50.0 mL). The organic layer was separated and the aqueous layer was extracted with chloroform. The combined organic layer was dried over $MgSO_4$. The solvent was removed under reduced pressure. The residue was purified by preparative TLC on silica gel eluting with AcOEt-*n*-hexane (1:50) to give 1,2,3-trimethoxy-4-(1-(2,4,6-trimethylphenyl)but-1-yn-3-yl)benzene (**2n**) (55.0 mg, 63%) as a yellow oil.

IR (KBr, cm^{-1}) ν 2963, 2932, 2872, 1600, 1509, 1494. 1465, 1434, 1416, 1297, 1278, 1259, 1097, 1035, 1020, 855, 801. 757, 693; ^{1}H NMR (600 MHz, $CDCl_3$) δ 1.11 (3H, t, J=6.9 Hz, Me), 1.73-1.85 (2H, m, CH2), 2.26 (3H, s, Me), 2.40 (6H, s, Mex2), 3.85 (3H, s, OMe), 3.88 (3H, s, OMe), 3.94 (3H, s, OMe), 4.20 (1H, dd, *J* = 5.2 and 8.6 Hz, CH), 6.66 (1H, d, *J* = 8.6 Hz, ArH), 6.85 (2H, s, ArH), 7.29 (1H, d, *J* = 8.6 Hz, ArH); ^{13}C NMR (150 MHz, $CDCl_3$) δ 12.1 (q), 21.1 (q), 21.2 (q), 31.0 (t), 33.6 (d), 55.9 (q), 60.7 (q), 61.1 (q), 80.1 (s), 99.8 (s), 107.1 (d), 120.6 (s), 122.7 (d), 127.4 (dx2), 128.5 (s), 136.8 (s), 139.9 (sx2), 141.9 (s), 150.8 (s), 152.3 (s); EIMS m/z 352 (M^+), 337 (M+-Me), 323 (M+-MeO); high resolution mass calcd for $C_{23}H_{28}O_3$: 352.2038, found: m/z 352.2034. Anal. Calcd for $C_{23}H_{28}O_3$(+1/3H_2O): C, 77.06; H, 8.07. Found: C, 76.91; H, 8.07.

Synthesis of 1-(1-(4-fluorophenyl)oct-1-yn-3-yl)-2,3,4-trimethoxybenzene (**2o**).

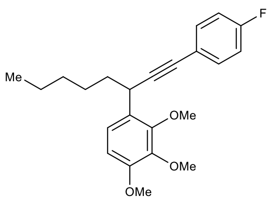

To a nitromethane (1.10 mL) solution of 1-(4-fluorophenyl)oct-1-yn-3-ol (**1o**) (50.0 mg, 0.227 mmol), 1,2,3-trimethoxybenzene (115 mg, 0.681 mmol), tetrabutylammonium hexafluorophosphate (17.6 mg, 0.0454 mmol), and 1,1'-binaphthol (13.0 mg, 0.0454 mmol) were added silver perchlorate (18.8 mg, 0.0908 mmol) and indium trichloride (10.0 mg, 0.0454 mmol) at room temperature. The reaction mixture was stirred for 12 h. The reaction mixture was poured into water (50.0 mL). The organic layer was separated and the aqueous layer was extracted with AcOEt. The combined organic layer was dried over $MgSO_4$. The solvent was removed under reduced pressure. The residue was purified by preparative TLC on silica gel eluting with AcOEt-*n*-hexane (1:40) to give a yellow oil. The titled compound was precipitated from *n*-hexane to give 1-(1-(4-fluorophenyl)oct-1-yn-3-yl)-2,3,4-trimethoxybenzene (**2o**) (41.1 mg, 49%) as a yellow oil and 5-(1-(2,4-difluorophenyl)oct-1-yn-3-yl)-1,2,3-trimethoxybenzene (**2o**) (10.7 mg, 13%) as a yellow oil.

IR (KBr, cm^{-1}) ν 22932, 2856, 2230, 1600, 1507, 1494, 1466, 1417, 11278, 1231, 1156, 1095, 1042, 1017, 837, 801; ^{1}H NMR (600 MHz, $CDCl_3$) δ 0.89 (3H, t, *J* = 7.5 Hz, Me), 1.30-1.37 (4H, m, CH_2), 1.42-1.59 (2H, m, CH_2), 1.74 (2H, q, *J* = 7.5 Hz, CH_2), 3.85 (3H, s, Me), 3.88 (3H, s, OMe), 3.94 (3H, s, OMe), 4.13 (1H, t, *J* = 7.5 Hz, CH), 6.69 (1H, d, *J* = 8.5 Hz, ArH), 6.97 (2H, t, *J* = 8.6 Hz, ArH), 7.20 (1H, d, *J* = 8.6 Hz, ArH), 7.39 (2H, dd, *J* = 5.6 and 8.6 Hz, ArH); ^{13}C NMR (150 MHz, $CDCl_3$) δ 14.0 (q), 22.5 (t), 27.3 (t), 31.5 (t), 31.5 (d), 37.5 (t), 56.0 (q), 60.7 (q), 61.1 (q), 80.9 (s), 92.2 (q), 107.3 (d), 115.2 (d), 115.4 (d), 120.0 (s), 122.5 (d), 128.3 (s), 133. 3 (d), 133.4 (d), 142.0 (s), 150.9 (s), 152.5 (s), 162.1 (d, *J* = 248.3 Hz); EIMS m/z 370 (M^+), 355 (M^+-Me), 299 (M^+-$(CH_2)_4$Me); high resolution mass calcd for $C_{23}H_{27}FO_3$: 370.1944, found m/z 370.1952.

Synthesis of 1-(1-(2,4-difluorophenyl)oct-1-yn-3-yl)-2,3,4-trimethoxybenzene (**2p**).

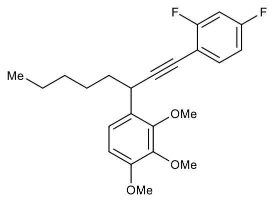

To a nitromethane (1.00 mL) solution of 1-(2,4-difluorophenyl)oct-1-yn-3-ol (**1p**) (50.0 mg, 0.210 mmol), 1,2,3-trimethoxybenzene (53.0 mg, 0.315 mmol), tetrabutylammonium hexafluorophosphate (16.3 mg, 0.0420 mmol), and 1,1'-binaphthol (16.2 mg, 0.0418 mmol) were added silver perchlorate (21.8 mg, 0.105 mmol) and indium trichloride (9.25 mg, 0.0420 mmol) at room temperature. The reaction mixture was stirred for 1 h. The reaction mixture was poured into water (50.0 mL). The organic layer was separated and the aqueous layer was extracted with AcOEt. The combined organic layer was dried over $MgSO_4$. The solvent was removed under reduced pressure. The residue was purified by preparative TLC on silica gel eluting with AcOEt-*n*-hexane (1:40) to give a yellow oil. The titled compound was precipitated from *n*-hexane to give 1-(1-(2,4-difluorophenyl)oct-1-yn-3-yl)-2,3,4-trimethoxybenzene (**2p**) (35.5 mg, 44%) as a yellow oil and 5-(1-(2,4-difluorophenyl)oct-1-yn-3-yl)-1,2,3-trimethoxybenzene (**2p**) (10.7 mg, 13%) as a yellow oil.

IR (KBr, cm^{-1}) ν 2961,2930, 2857, 1615, 1589, 1505, 1495, 1466, 1417, 1277, 1143, 1097, 1018, 967, 850; ^{1}H NMR (600 MHz, $CDCl_3$) δ 0.80 (3H, t, *J* =7.6 Hz, CH_3), 1.29-1.36 (4H, m, CH_2), 1.45-1.58 (2H, m, CH_2), 1.74-1.78 (2, m, CH_2), 3.85 (3H, s, OCH_3), 3.88 (3H, s, OCH_3), 3.94 (3H, s, OCH_3), 4.18 (1H, t, *J* = 6.9 Hz, CH), 6.68 (1H, d, *J* = 8.9 Hz, ArH), 6.79-6.82 (2H, m, ArH), 7.22 (1H, d, *J* = 8.2 Hz, ArH), 7.36-7.40 (1H, m, ArH); ^{13}C NMR (150 MHz, $CDCl_3$) δ 14.0 (q), 22.5 (t), 27.1 (t), 31.4 (t), 31.8 (d), 37.4 (t), 55.9 (q), 60.7 (q), 61.1 (q), 74.4 (s), 97.7 (s), 104.0 (t, *J* = 26.0 Hz), 107.3 (d), 108.8 (dd, *J* = 4.3 and 15.9 Hz), 111.2 (dd, *J* = 21.7 and 2.9 Hz), 122.5 (d), 127.9 (s), 134.2 (d, *J*

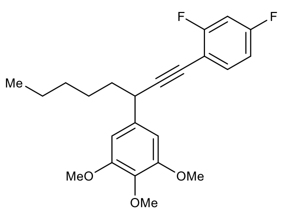

= 10.1 and 2.9 Hz), 142.0 (s), 150.1 (s), 152.5 (s), 162.1 (dd, *J* = 11.5 and 249.9 Hz), 163.1 (dd, *J* = 11.6 and 252.9 Hz); EIMS m/z 388 (M^+), 373 (M^+ -Me), 357 (M^+ -OMe). Anal. Calcd for $C_{23}H_{26}O_3F_2$ (+1/10H_2O): C, 70.78; H, 6.80. Found: C, 70.72; H, 6.86.

5-(1-(2,4-difluorophenyl)oct-1-yn-3-yl)-1,2,3-trimethoxybenzene (**2p**)

IR (KBr, cm^{-1}) ν 2929, 2855, 2243, 1715, 1592, 1505, 1463, 1421, 1237, 1129, 1098, 1011, 967, 850; ^{1}H NMR (600 MHz, $CDCl_3$) δ 0.90 (3H, t, *J* = 7.4 Hz, Me), 1.33 (4H, brs, CH_2x2), 1.46-1.52 (2H, m, CH_2), 1.79-1.82 (2H, m, CH2), 3.79 (1H, t, *J* = 7.4 Hz, CH), 3.84 (3H, s, OMe), 3.88 (6H, s, OMex2), 6.66 (2H, s, ArH), 6.83 (2H, t, *J* = 7.6 Hz, ArH), 7.37-7.41 (1H, m, ArH); EIMS m/z 388 (M^+); high resolution mass calcd for $C_{23}H_{26}F_2O_3$: 388.1850, found m/z 388.1857.

Synthesis of 1,2,3-trimethoxy-4-(1-(2,4-dichlorophenyl)oct-1-yn-3-yl)benzene (**2q**)

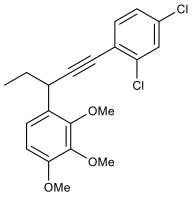

To a nitromethane (1.00 mL) solution of 1-(2,4-dichlorophenyl)oct-1-yn-3-ol (**1q**) (50.0 mg, 0.184 mmol), 1,2,3-trimethoxybenzene (93.0 mg, 0.553 mmol), tetrabutylammonium hexafluorophosphate (14.3 mg, 0.0369 mmol), and (R)-1,1'-binaphthol (10.6 mg, 0.0369 mmol) were added silver perchlorate (15.3 mg, 0.0736 mmol) and indium trichloride (8.16 mg, 0.0369 mmol) at room temperature. The reaction mixture was stirred for 23 h. The reaction mixture was poured into water (50.0 mL). The organic layer was separated and the aqueous layer was extracted with AcOEt. The combined organic layer was dried over $MgSO_4$. The solvent was removed under reduced pressure. The residue was purified by preparative TLC on silica gel eluting with AcOEt-*n*-hexane (1:40) to give 1-(1-(2,4-dichlorophenyl)hept-1-yn-3-yl)-2,3,4-trimethoxybenzene (**2q**) (38.4 mg, 50%) as a yellow oil.

IR (KBr, cm^{-1}) ν 2957, 2933, 2857, 2230, 1598, 1494, 1466, 1435, 1417, 1292, 1277, 1258, 1097, 1042, 1019, 819, 801, 754; ^{1}H NMR (500 MHz, $CDCl_3$) δ 0.82 (3H, t, *J* = 6.9 Hz, CH_3), 1.21-1.30 (4H, m, CH_2), 1.40-1.55 (2H, m, CH_2), 1.67-1.72 (2H, m, CH_2), 3.79 (3H, s, OCH_3), 3.81 (3H, s, OCH_3), 3.87 (3H, s, OCH_3), 4.13 (1H, t, *J* = 7.5 Hz, CH), 6.61 (1H, d, *J* = 8.6 Hz, ArH), 7.09 (1H, d, *J* = 8.6 and 2.3 Hz, ArH), 7.19 (1H, d, *J* = 3.5 Hz ArH), 7.30 (1H, d, *J* = 8.5 Hz, ArH), 7.33 (1H, d, *J* = 1.7 Hz, ArH); ^{13}C NMR (150 MHz, $CDCl_3$) δ 14.0 (q), 22.5 (t), 27.2 (t), 31.4 (t), 31.8 (d), 37.5 (t), 56.0 (q), 60.7 (q), 61.1 (q), 78.2 (s), 99.2 (s), 107.2 (d), 122.4 (s), 122.6 (d), 126.8 (d), 127.8 (s), 129.1 (d), 133.7 (s), 133.8 (d), 136.7 (s), 141,9 (s), 150.8 (s), 152.5 (s); MS m/z 420 (M^+), 349 (M^+-$(CH_2)_4$Me); high resolution mass calcd for $C_{23}H_{26}Cl_2O_3$:420.1259, found m/z 420.1253. Anal. Calcd for $C_{23}H_{26}Cl_2O_3$(+9/10H_2O): C, 63.13; H, 6.40. Found: C, 63.16.; H, 6.30.

Synthesis of 1,2,3-trimethoxy-4-(1-(*p*-chlorophenyl)pent-1-yn-3-yl)benzene (**2r**).

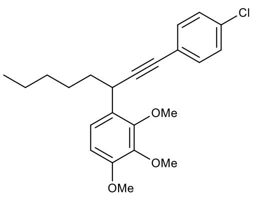

To a nitromethane (1.00 mL) solution of 1-(*p*-chlorophenyl)pent-1-yn-3-ol (**1r**) (50.0 mg, 0.211 mmol), 1,2,3-trimethoxybenzene (107 mg, 0.634 mmol), tetrabutylammonium hexafluorophosphate (16.4 mg, 0.0422 mmol), and (R)-1,1'-binaphthol (12.1 mg, 0.0422 mmol) were added silver perchlorate (17.5 mg, 0.0846 mmol) and indium trichloride (9.30 mg, 0.0422 mmol) at room temperature. The reaction mixture was stirred for 30 min. The reaction mixture was poured into water (50.0 mL). The organic layer was separated and the aqueous layer was extracted with AcOEt. The combined organic layer was dried over $MgSO_4$. The solvent was removed under reduced pressure. The residue was purified by preparative TLC on silica gel eluting with AcOEt-*n*-hexane (1:40) to give 1,2,3-trimethoxy-4-(1-(*p*-chlorophenyl)pent-1-yn-3-yl)benzene (**2r**) (49.8 mg, 61%) as a yellow oil.

IR (KBr, cm^{-1}) ν 2931, 2856, 2228, 1599, 1491, 1465, 1417, 1255, 1095, 1042, 1015, 902, 818; ^{1}H NMR (500 MHz, $CDCl_3$) δ 0.89 (3H, t, J = 6.9 Hz, Me), 1.32-1.36 (4H, m, CH_2), 1.42-1.58 (4H, m, CH_2), 3.85 (3H, s, OMe), 3.88 (3H, s, OMe), 3.93 (3H, s, OMe), 4.13 (1H, t, *J* = 6.8 Hz, CH), 6.67 (1H, d, *J* = 6.8 Hz, ArH), 7.19 (1H, d, *J* = 6.8 Hz, ArH), 7.25 (2H, d, *J* = 8.6 Hz, ArH), 7.34 (2H, d, *J* = 8.6 Hz, ArH); ^{13}C NMR (150 MHz, $CDCl_3$) δ 14.0 (q), 22.5 (t), 27.2 (t), 27.2 (t), 31.4 (t), 31.6 (d), 37.4 (t), 55.9 (q), 60.7 (q), 61.1 (q), 80.9 (s), 93.6 (s), 107.3 (d), 122.5 (d), 128.1 (s), 128.4 (dx2), 132.8 (dx2), 133.4 (s), 142.0 (s), 150.9 (s), 152.5 (s); High resolution mass calcd for $C_{20}H_{21}ClO_3$: 386.1649, found m/z 386.1666. Anal. Calcd for $C_{23}H_{27}ClO_3$(+1/4H_2O): C, 70.57; H, 7.08. Found: C, 70.39; H, 7.10.

Synthesis of 1,2,3-trimethoxy-4-(1-(3-chlorophenyl)pent-1-yn-3-yl)benzene (**2s**).

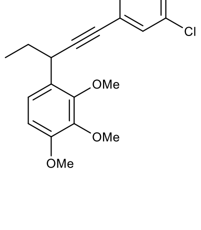

To a nitromethane (1.50 mL) solution of 1-(3-chlorophenyl)pent-1-yn-3-ol (**1s**) (62.0 mg, 0.319 mmol), 1,2,3-trimethoxybenzene (161 mg, 0.956 mmol), tetrabutylammonium hexafluorophosphate (24.7 mg, 0.0638 mmol), and (R)-1,1'-binaphthol (18.3 mg, 0.0638 mmol) were added silver perchlorate (26.5 mg, 0.128 mmol) and indium trichloride (10.2 mg, 0.0638 mmol) at room temperature. The reaction mixture was stirred for 30 min. The reaction mixture was poured into water (50.0 mL). The organic layer was separated and the aqueous layer was extracted with AcOEt. The combined organic layer was dried over $MgSO_4$. The solvent was removed under reduced pressure. The residue was purified by preparative TLC on silica gel eluting with AcOEt-*n*-hexane (1:50) to give 1,2,3-trimethoxy-4-(1-(3-chlorophenyl)pent-1-yn-3-yl)benzene (**2s**) (40.5 mg, 37%) as a yellow oil.

IR (KBr, cm^{-1}) ν 2965, 2873, 2230, 1592, 1494, 1465, 1417, 1304, 1278, 1255, 1097, 1035, 1018, 873, 784, 683; ^{1}H NMR (600 MHz, $CDCl_3$) δ 1.06 (3H, t, J = 7.5 Hz, Me), 1.74-1.83 (2H, m, CH_2), 3.86 (3H, s, OMe), 3.88 (3H, s, OMe), 3.94 (3H, s, OMe), 4.09 (1H, dd, *J* = 5.7 and 8.0 Hz, CH), 6.68 (1H, d, *J* = 8.6 Hz, ArH), 7.18 (1H, d, *J* = 8.6 Hz, ArH), 7.21 (1H, d, *J* = 7.5 Hz, ArH), 7.25 (1H, d, *J* = 7.5 Hz, ArH), 7.30 (1H, d, *J* = 7.5 Hz, ArH), 7.42 (1H, brs, ArH); ^{13}C NMR (150 MHz, $CDCl_3$) δ 12.2 (q), 30.6 (t), 33.3 (d), 56.1 (q), 60.8 (q), 61.2 (q), 81.1 (s), 93.9 (s), 107.3 (d), 122.7 (d), 125.8 (s), 127.8 (s), 128.0 (d), 129.5 (d), 129.8 (d), 131.6 (d), 134.1 (s), 142.1 (s), 151.1 (s), 152.7 (s); EIMS m/z 344 (M^+); high resolution mass calcd for $C_{20}H_{21}ClO_3$: 344.1179, found m/z 344.1197. Anal. Calcd for $C_{20}H_{21}ClO_3$(+1/12H_2O): C, 69.36; H, 6.16. Found: C, 69.35; H, 6.23.

Synthesis of 1-(4-(2-fluorophenyl)but-3-yn-2-yl)-2,3,4-trimethoxybenzene (**2t**)

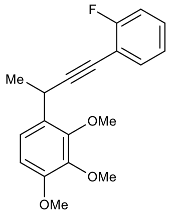

To a nitromethane (1.50 mL) solution of 1-(2-fluorophenyl)but-1-yn-3-ol (**1t**) (50.0 mg, 0.305 mmol), 1,2,3-trimethoxybenzene (154 mg, 0.914 mmol), tetrabutylammonium hexafluorophosphate (23.6 mg, 0.0609 mmol), and 1,1'-binaphthol (17.4 mg, 0.0609 mmol) were added silver perchlorate (25.3 mg, 0.122 mmol) and indium trichloride (13.5 mg, 0.0609 mmol) at room temperature. The reaction mixture was stirred for 1 h. The reaction mixture was poured into water (50.0 mL). The organic layer was separated and the aqueous layer was extracted with AcOEt. The combined organic layer was dried over $MgSO_4$. The solvent was removed under reduced pressure. The residue was purified by preparative TLC on silica gel eluting with AcOEt-*n*-hexane (1:40) to give 1-(4-(2-fluorophenyl)but-3-yn-2-yl)-2,3,4-trimethoxybenzene (**2t**) (38.9 mg, 41%) as a yellow oil and 5-(1-(2-fluorophenyl)but-1-yn-3-yl)-1,2,3-trimethoxybenzene (**2t**) (4.90 mg, 10%) as a yellow oil.

IR (KBr, cm^{-1}) ν 2933, 2233, 1600, 1494, 1466, 1417, 1301, 1281, 1255, 1098, 1030, 814, 802, 758; ^{1}H NMR (600 MHz, $CDCl_3$) δ 1.53 (3H, d, *J* = 7.1 Hz, Me), 3.85 (3H, s, OMe), 3.88 (3H, s, OMe), 4.31 (1H, q, *J* = 7.1 Hz, CH), 6.69 (1H, d, *J* = 8.7 Hz, ArH), 7.03 (1H, d, *J* = 8.4 Hz, ArH), 7.05 (1H, d, *J* = 9.3 Hz, ArH), 7.22-7.26 (1H, m, ArH), 7.29 (1H, d, *J* = 8.6 Hz, ArH), 7.41 (1H, dt, *J* = 1.8 and 7.5 Hz, ArH); ^{13}C NMR (150 MHz, $CDCl_3$) δ 23.5 (q), 26.3 (d), 56.0 (q), 60.7 (q), 61.1 (q), 74.8 (s), 98.8 (d, *J* = 3.3 Hz), 107.3 (d), 112.3 (d, *J* = 15.5 Hz), 115.3 (d, *J* = 11.3 Hz), 122.0 (d), 123.7 (d, *J* = 3.6 Hz), 128.9 (d), 129.2 (d, *J* = 7.9 Hz), 133.4 (d, *J* = 1.5 Hz), 142.0 (s), 150.8 (d), 162.0 (d), 163.6 (d, *J* = 250.7 Hz); EIMS m/z 314 (M^+); high resolution mass calcd for $C_{19}H_{19}FO_3$: 314.1318, found m/z 314.1322. Anal. Calcd for $C_{19}H_{19}FO_3$(+1/5H_2O): C, 71.77; H, 6.15. Found: C, 71.51; H, 5.82.

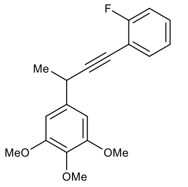

5-(1-(2-fluorophenyl)but-1-yn-3-yl)-1,2,3-trimethoxybenzene (**2t**)

IR (KBr, cm^{-1}) ν 2935, 2837, 2364, 2344, 1508, 1492, 1457, 1419, 1343, 1240, 1129, 1009, 757; ^{1}H NMR (600 MHz, $CDCl_3$) δ 1.59 (3H, d, *J* = 7.3 Hz, Me), 3.84 (3H, s, OMe), 3.90 (6H, s, Mex2), 3.93-3.97 (1H, m, CH), 6.73 (2H, s, ArH), 7.05-7.10 (2H, m, ArH), 7.25-7.30 (1H, m, ArH), 7.43 (1H, dt, *J* = 1.8 and 8.0 Hz, ArH); EIMS m/z 314 (M^+), high resolution mass calcd for $C_{19}H_{19}FO_3$: 314.1381, found m/z 314.1367.

Synthesis of 1-(1-(4-bromophenyl)oct-1-yn-3-yl)-2,3,4-trimethoxybenzene (**2u**)

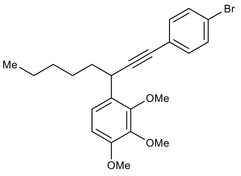

To a nitromethane (1.50 mL) solution of 1-(4-bromophenyl)oct-1-yn-3-ol (**1u**) (50.0 mg, 0.178 mmol), 1,2,3-trimethoxybenzene (89.7 mg, 0.533 mmol), tetrabutylammonium hexafluorophosphate (13.8 mg, 0.0356 mmol), and 1,1'-binaphthol (10.2 mg, 0.0356 mmol) were added silver perchlorate (14.7 mg, 0.0711 mmol) and indium trichloride (7.9 mg, 0.0356 mmol) at room temperature. The reaction mixture was stirred for 12 h. The reaction mixture was poured into water (50.0 mL). The organic layer was separated and the aqueous layer was extracted with AcOEt. The combined organic layer was dried over $MgSO_4$. The solvent was removed under reduced pressure. The residue was purified by preparative TLC on silica gel eluting with AcOEt-*n*-hexane (1:30) to give 1-(1-(4-bromophenyl)oct-1-yn-3-yl)-2,3,4-trimethoxybenzene (**2u**) (44.8 mg, 58%) as a yellow oil.

IR (KBr, cm^{-1}) ν 2930, 2856, 2228, 1600, 1493, 1465, 1416, 1278, 1095, 1041, 1011, 824, 801; ^{1}H NMR (600 MHz, $CDCl_3$) δ 0.89 (3H, t, *J* = 6.9 Hz, Me), 1.29-1.35 (4H, m, CH_2), 1.44-1.56 (2H, m, CH_2), 1.74 (2H, q, *J* = 6.8 Hz, ArH), 3.85 (3H, s, OMe), 3.86 (3H, s, OMe), 3.93 (3H, s, OMe), 4.13 (1H, t, *J* = 7.2 Hz, CH), 6.68 (1H, d, *J* = 8.7 Hz, ArH), 7.19 (1H, d, *J* = 8.7 Hz, ArH), 7.28 (2H, d, *J* = 8.3 Hz, ArH), 7.41 (2H, d, *J* = 8.4 Hz, ArH); ^{13}C NMR (150 MHz, $CDCl_3$) δ 14.0 (q), 22.5 (t), 27.2 (t), 31.4 (d), 31.6 (t), 37.4 (t), 56.0 (q), 60.7 (q), 61.1 (q), 81.0 (s), 93.9 (s), 107.3 (d), 121.6 (s), 122.5 (d), 122.9 (s), 128.1 (s), 131.3 (dx2), 133.0 (dx2), 142.0 (s), 150.9 (s), 152.5 (s); EIMS m/z 430 (M^+); high resolution mass calcd for $C_{23}H_{27}BrO_3$: 430.1144; found m/z 430.1153. Anal. Calcd for $C_{23}H_{27}BrO_3$: C, 64.04; H, 6.31. Found: C, 64.22; H, 6.20.

Synthesis of 1,2,3-trimethoxy-4-(4-(2-(trifluoromethyl)phenyl)but-3-yn-2-yl)benzene (**2v**).

To a nitromethane (1.10 mL) solution of 1-(3-trifluoromethylphenyl)but-1-yn-3-ol (**1v**) (50.0 mg, 0.233 mmol), 1,2,3-trimethoxybenzene (118 mg, 0.700 mmol), tetrabutylammonium hexafluorophosphate (18.1 mg, 0.0467 mmol), and 1,1'-binaphthol (13.4 mg, 0.0467 mmol) were added silver perchlorate (19.4 mg, 0.0934 mmol) and indium trichloride (10.3 mg, 0.0467 mmol) at room temperature. The reaction mixture was refluxed for 10 min and then poured into water (50.0 mL). The organic layer was separated and the aqueous layer was extracted with AcOEt. The combined organic layer was dried over $MgSO_4$. The solvent was removed under reduced pressure. The residue

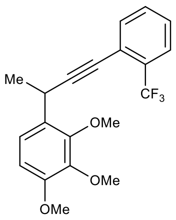

was purified by preparative TLC on silica gel eluting with AcOEt-*n*-hexane (1:40) to give 1,2,3-trimethoxy-4-(4-(2-(trifluoromethyl)phenyl)but-3-yn-2-yl)benzene (**2v**) (41.5 mg, 49%) as a yellow oil and 1,2,3-trimethoxy-5-(4-(2-(trifluoromethyl)phenyl)but-3-yn-2-yl)benzene (**2v**) (10.3 mg, 12%) as a yellow oil.

IR (KBr, cm^{-1}) ν 2974, 2936, 2837, 2232, 1603, 1494, 1468, 1417, 1319, 1283, 1259, 1171, 1134, 1101, 1058, 1033, 913, 802, 767; ^{1}H NMR (600 MHz, $CDCl_3$) δ 1.45 (3H, d, *J* = 6.9 Hz, Me), 3.78 (3H, s, OMe), 3.80 (3H, s, OMe), 3.87 (3H, s, OMe), 4.23 (1H, q, *J* = 6.9 Hz, CH), 6.61 (1H, d, *J* = 8.5 Hz, ArH), 7.19 (1H, d, *J* = 8.5 Hz, ArH), 7.27 (1H, t, *J* = 7.4 Hz, ArH), 7.37 (1H, t, *J* = 7.5 Hz, ArH), 7.46 (1H, d, *J* = 8.0 Hz, ArH), 7.55 (1H, d, *J* = 8.0 Hz, ArH); ^{13}C NMR (150 MHz, $CDCl_3$) δ 23.4 (q), 26.2 (d), 56.0 (q), 60.7 (q), 61.1 (q), 77.2 (s), 99.4 (s), 107.4 (d), 122.0 (d), 122.2 (s), 123.4 (q, *J* = 275.4 Hz), 125.6 (q, *J* = 4.8 Hz), 127.3 (d), 128.8 (s), 131.2 (d), 131.5 (q, *J* = 30.0 Hz), 133.8 (d), 142.0 (s), 150.7 (s), 152.6 (s); EIMS m/z 364 (M^+), 349 (M^+-Me); high resolution mass calcd for $C_{20}H_{19}F_3O_3$: 364.1286, found m/z 364.1304. Anal. Calcd for $C_{24}H_{27}NO_3$(+1/5H_2O): C, 71.77; H, 6.15. Found: C, 71.51; H, 5.82.

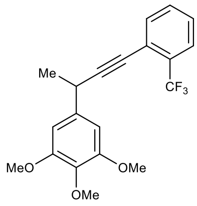

1,2,3-trimethoxy-5-(4-(2-(trifluoromethyl)phenyl)but-3-yn-2-yl)benzene (**2v**)

IR (KBr, cm^{-1}) ν 3003, 2938, 2838, 2233, 1593, 1509, 1491, 1463, 1420, 1319, 1236, 1171, 1130, 1058, 1034, 1008, 836, 766; ^{1}H NMR (500 MHz, $CDCl_3$) δ 1.52 (3H, d, *J* = 6.9 Hz, Me), 3.77 (3H, s, OMe), 3.81 (6H, s, OMex2), 3.88 (1H, q, *J* = 7.3 Hz, CH), 6.61 (2H, s, ArH), 7.31 (1H, t, *J* = 7.8 Hz, ArH), 7.40 (1H, t, *J* = 7.8 Hz, ArH), 7.49 (1H, d, *J* = 7.8 Hz, ArH), 7.57 (1H, d, *J* = 7.8 Hz, ArH); EIMS m/z 364 (M^+), 349 (M^+-Me); high resolution mass calcd for $C_{20}H_{19}F_3O_3$: 364.1286, found m/z 364.1304.

Synthesis of 1,2,3-trimethoxy-4-(4-(3-(trifluoromethyl)phenyl)but-1-yn-3-yl)benzene (**2w**).

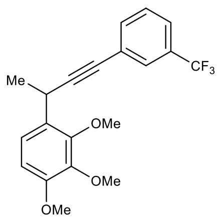

To a nitromethane (1.00 mL) solution of 1-(3-trifluoromethylphenyl)but-1-yn-3-ol (**1w**) (50.0 mg, 0.233 mmol), 1,2,3-trimethoxybenzene (118 mg, 0.700 mmol), tetrabutylammonium hexafluorophosphate (18.1 mg, 0.0467 mmol), and 1,1'-binaphthol (13.4 mg, 0.0467 mmol) were added silver perchlorate (19.4 mg, 0.0934 mmol) and indium trichloride (10.3 mg, 0.0467 mmol) at room temperature. The reaction mixture was stirred for 12 h. The reaction mixture was poured into water (50.0 mL). The organic layer was separated and the aqueous layer was extracted with AcOEt. The combined organic layer was dried over $MgSO_4$. The solvent was removed under reduced pressure. The residue was purified by preparative TLC on silica gel eluting with AcOEt-*n*-hexane (1:40) to give 1,2,3-trimethoxy-4-(4-(3-(trifluoromethyl)phenyl)but-1-yn-3-yl)benzene (**2w**) (41.5 mg, 49%) as a yellow oil and 1,2,3-trimethoxy-5-(4-(2-(trifluoromethyl)phenyl)but-3-yn-2-yl)benzene (**2w**) (8.3 mg, 10%) as a yellow oil.

IR (KBr, cm^{-1}) ν 2975, 2937, 2835, 2229, 600, 1495, 1467, 1432, 1236, 1168, 1130, 1256, 1071, 1031, 883, 801, 697; ^{1}H NMR (600 MHz, $CDCl_3$) δ 1.52 (3H, d, *J* = 6.9 Hz, Me), 3.86 (3H, s, OMe), 3.88 (3H, d, *J* = 1.1 Hz, OMe), 3.95 (3H, d, *J* = 1.1 Hz, OMe), 4.28 (1H, q, *J* = 7.4 Hz, CH), 6.70 (1H, d, *J* = 9.2 Hz, ArH), 7.23 (1H, d, *J* = 8.6 Hz, ArH), 7.40 (1H, t, *J* = 8.0 Hz, ArH), 7.51 (1H, d, *J* = 8.0 Hz, ArH), 7.58 (1H, d, *J* = 8.0 Hz, ArH), 7.68 (1H, brs, ArH); ^{13}C NMR (150 MHz, $CDCl_3$) δ 23.4 (q), 26.0 (t), 56.0 (q), 60.7 (q), 61.2 (q), 79.9 (s), 95.3 (s), 107.4 (d), 122.7 (d), 123.8 (q, *J* = 272.4 Hz, CF_3), 124.1 (q, *J* = 4.0 Hz), 124.8 (s), 128.3 (q, *J* = 3.6 Hz). 128.7 (d), 128.8 (s), 130.7 (q, *J* = 32.6 Hz), 134.7 (d), 142.1 (s), 150.8 (s), 152.7 (s); EIMS m/z 364 (M^+), high resolution mass calcd for $C_{20}H_{19}F_3O_3$: 364.1286, found m/z 364.1265. Anal. Calcd for $C_{20}H_{19}F_3O_3$(1/5H_2O): C, 65.28; H, 5.31. Found: C, 65.23; H, 5.22.

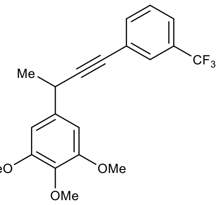

1,2,3-trimethoxy-5-(4-(2-(trifluoromethyl)phenyl)but-3-yn-2-yl)benzene (**2w**)

IR (KBr, cm^{-1}) ν 2975, 2938, 2837, 2228, 1952, 1508, 1463, 1336, 1234, 1168, 1129, 1071, 1009, 897, 832, 803, 697; ^{1}H NMR (600 MHz, $CDCl_3$) δ 1.59 (3H, t, *J* = 6.9 Hz, Me), 3.85 (3H, s, OMe), 3.89 (6H, s, OMex2), 3.93 (1H, q, *J* = 6.9 Hz, CH), 6.66 (2H, s, ArH), 7.43 (1H, t, *J* = 8.0 Hz, ArH), 7.54 (1H, d, *J* = 8.0 Hz, ArH), 7.59 (1H, d, *J* = 7.5 Hz, ArH), 7.69 (1H, brs, ArH); EIMS m/z 364 (M^+); high resolution mass calcd for $C_{20}H_{19}F_3O_3$: 364.1286, found m/z 364.1304.

Synthesis of ethyl 4-(3-(2,3,4-trimethoxyphenyl)oct-1-yn-1-yl)benzoate (**2x**).

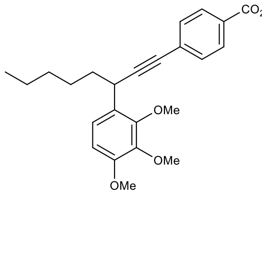

To a nitromethane (1.00 mL) solution of ethyl 4-(3-hydroxyoct-1-yn-3-yl)benzoate (**1x**) (40.0 mg, 0.146 mmol), 1,2,3-trimethoxybenzene (82.0 mg, 0.487 mmol), and tetrabutylammonium hexafluorophosphate (12.6 mg, 0.0324 mmol), 1,1'-binaphthol (9.30 mg, 0.0324 mmol) were added silver perchlorate (16.8 mg, 0.081 mmol) and indium trichloride (7.20 mg, 0.0324 mmol) at room temperature. The reaction mixture was stirred for 12 h. The reaction mixture was filtered through a pad of celite with chloroform and then poured into water (50 mL). The organic layer was separated and the aqueous layer was extracted with ethyl acetate. The combined organic layer was dried over $MgSO_4$. The solvent was removed under reduced pressure. The residue was

purified by preparative TLC on silica gel eluting with AcOEt-*n*-hexane (1:20) to give ethyl 4-(3-(2,3,4-trimethoxyphenyl)oct-1-yn-1-yl)benzoate (**2x**) (25.0 mg, 40%) as a yellow oil and ethyl 4-(3-(3,4,5-trimethoxyphenyl)oct-1-yn-1-yl)benzoate (**2x**) (8.70 mg, 14%) as a yellow oil.

IR (KBr, cm^{-1}) ν 2932, 2856, 2354, 2225, 1719, 1605, 1494, 1466, 1417, 1274, 1174, 1096, 1019, 858, 801, 770, 697; ^{1}H NMR (600 MHz, $CDCl_3$) δ 0.89 (3H, t, *J* = 6.9 Hz, Me), 1.31-1.36 (4H, m, CH_2), 1.39 (3H, t, *J* = 6.9 Hz, Me), 1.43-1.59 (2H, m, CH_2), 1.73-1.80 (2H, m, CH_2), 3.86 (3H, s, OMe), 3.88 (3H, s, OMe), 3.94 (3H, s, OMe), 4.17 (2H, dd, *J* = 6.2 and 8.2 Hz, CH), 4.37 (2H, q, *J* = 6.8 Hz. CH_2), 6.69 (2H, d, *J* = 8.9 Hz, ArH), 7.19 (2H, d, *J* = 8.9 Hz, ArH), 7.47 (2H, d, *J* = 8.2 Hz, ArH), 7.96 (2H, d, *J* = 8.3 Hz, ArH); ^{13}C NMR (150 MHz, $CDCl_3$) δ 14.0 (q), 14.3 (q), 22.5 (t), 27.3 (t), 31.4 (t), 31.7 (d), 37.4 (t), 56.0 (q), 60.7 (q), 61.0 (t), 61.1 (q), 81.5 (s), 96.0 (s), 107.3 (d), 122.5 (d), 127.9 (s), 128.7 (s), 129.2 (s), 129.3 (dx2), 131.5 (dx2), 142.0 (s), 150.9 (s), 152.5 (s), 166.2 (s); EIMS m/z 424 (M^+). High resolution mass calcd for $C_{26}H_{32}O_5$: 424.2250, found m/z 424.2269. Anal. Calcd for $C_{26}H_{32}O_5(+1/5H_2O)$: C, 72.94; H, 7.63. Found C, 72.80; H, 7.84.

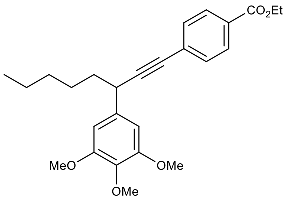

ethyl 4-(3-(3,4,5-trimethoxyphenyl)oct-1-yn-1-yl)benzoate (**2x**)

IR (KBr, cm^{-1}) ν 2933, 2857, 2360, 2228, 1716, 1593, 1507, 1463, 1420, 1365, 1274, 1220, 1175, 1129, 1107, 1018, 858, 770, 697, 644; ^{1}H NMR (600 MHz, $CDCl_3$) δ 0.90 (3H, t, *J* = 6.8 Hz, Me), 1.32-1.36 (4H, m, CH_2), 1.39 (3H, t, *J* = 6.8 Hz, Me), 1.44-1.58 (2H, m, CH_2), 1.78-1.88 (2H, m, CH_2), 3.77 (1H, dd, *J* = 6.2 and 8.2 Hz, CH), 3.85 (3H, s, OMe), 3.88 (6H, s, OMex2), 4.37 (2H, q, *J* = 6.8 Hz, OCH_2), 6.63 (2H, s, ArH), 7.47 (2H, d, *J* = 8.3 Hz, ArH), 7.98 (2H, d, *J* = 8.3 Hz, ArH); EIMS m/z 424 (M^+). High resolution mass calcd for $C_{26}H_{32}O_5$: 424.2250, found m/z 424.2243.

Synthesis of 2-(3-(2,3,4-trimethoxyphenyl)oct-1-yn-1-yl)benzonitrile (**2y**).

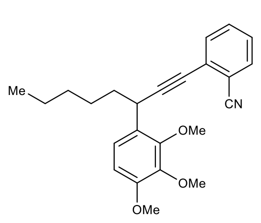

To a nitromethane (1.10 mL) solution of 2-(3-hydroxyoct-1-yn-3-yl)benzonitrile (**1y**) (50.0 mg, 0.220 mmol), 1,2,3-trimethoxybenzene (111 mg, 0.660 mmol), and tetrabutylammonium hexafluorophosphate (17.0 mg, 0.0440 mmol), 1,1'-binaphthol (12.6 mg, 0.0440 mmol) were added silver perchlorate (18.2 mg, 0.0880 mmol) and indium trichloride (9.70 mg, 0.0440 mmol) at room temperature. The reaction mixture was refluxed for 2h. The reaction mixture was poured into water (50 mL). The organic layer was separated and the aqueous layer was extracted with ethyl acetate. The combined organic layer was dried over $MgSO_4$. The solvent was removed under reduced pressure. The residue was purified by preparative TLC on silica gel eluting with AcOEt-*n*-hexane (1:20) to give 2-(3-(2,3,4-trimethoxyphenyl)oct-1-yn-1-yl)benzonitrile (**2y**) (27.4 mg, 33%) as a yellow oil and 4-(3-(3,4,5-trimethoxyphenyl)but-1-yn-1-yl)benzonitrile (**2y**) (15.3 mg, 18%) as a yellow oil.

IR (KBr, cm^{-1}) ν 2956, 2931, 2857, 2230, 1597, 1510, 1494, 1483, 1466, 1445, 1417, 1279, 1254, 1095, 1038, 761; ^{1}H NMR (600 MHz, $CDCl_3$) δ 0.89 (3H, t, *J* = 6.9 Hz, Me), 1.31-1.37 (4H, m, CH_2), 1.46-1.63 (2H, m, CH_2), 1.77-1.88 (2H, m, CH_2), 3.86 (3H, s, OMe), 3.88 (3H, s, OMe), 3.95 (3H, s, OMe), 4.24 (1H, dd, *J* = 5.9 and 8.7 Hz, CH), 6.71 (1H, d, *J* = 8.7 Hz, ArH), 7.32 (1H, d, *J* = 8.7 Hz, ArH), 7.35-7.37 (1H, m, ArH), 7.50-7.51 (2H, m, ArH), 7.62 (1H, d, *J* = 7.8 Hz, ArH); ^{13}C NMR (150 MHz, $CDCl_3$) δ 14.0 (q), 22.5 (t), 27.2 (t), 31.4 (t), 31.6 (d), 37.4 (t), 55.9 (q), 60.7 (q), 61.2 (q), 77.3 (s), 99.9 (s), 107.4 (d), 115.3 (s), 117.8 (s), 122.8 (d), 127.6 (s), 127.4 (d), 127.9 (s), 132.2 (d), 132.3 (d), 132.5 (d), 141.9 (s), 150.8 (s), 152.6 (s); EIMS m/z 377 (M^+), 306 (M^+-$(CH_2)_4Me$); high resolution mass calcd for $C_{20}H_{21}ClO_3$: 377.1991, found m/z 377.2024.

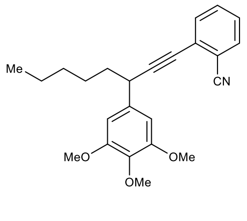

4-(3-(3,4,5-trimethoxyphenyl)but-1-yn-1-yl)benzonitrile (**2y**)

IR (KBr, cm^{-1}) ν 2932, 2857, 2229, 1508, 1482, 1463, 1421, 1336, 1237, 1128, 1102, 1010, 765; ^{1}H NMR (600 MHz, $CDCl_3$) d 0.89 (3H, t, *J* = 6.9 Hz, Me), 1.32-1.36 (4H, m, CH2), 1.44-1.62 (2H, m, CH_2), 1.81-1.95 (2H, m, CH_2), 3.82 (1H, dd, *J* = 6.3 and 8.6 Hz, CH), 3.84 (3H, s, OMe), 3.90 (6H, s, OMex2), 6.67 (2H, s, ArH), 7.35-7.39 (1H, m, ArH), 7.51 (2H, d, *J* = 4.0 Hz, ArH), 7.63 (1H, d, *J* = 8.0 Hz, ArH); EIMS m/z 377 (M^+), 306 (M^+ -$(CH_2)_4Me$); high resolution mass calcd for $C_{20}H_{21}ClO_3$: 377.1991, found m/z 377.1969.

Synthesis of 3-(3-(2,3,4-trimethoxyphenyl)oct-1-yn-1-yl)benzonitrile (**2z**)

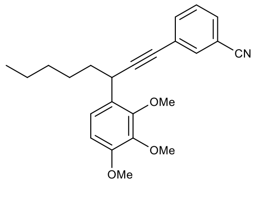

To a nitromethane (1.1 mL) solution of 3-(3-hydroxyoct-1-yn-1-yl)benzonitrile (**1z**) (50.0 mg, 0.220 mmol), 1,2,3-trimethoxybenzene (111 mg, 0.660 mmol), tetrabutylammonium hexafluorophosphate (17.0 mg, 0.0440 mmol), and (R)-1,1'-binaphthol (12.6 mg, 0.0440 mmol) were added silver perchlorate (18.2 mg, 0.0880 mmol) and indium trichloride (9.7 mg, 0.0440 mmol) at room temperature. The reaction mixture was refluxed for 5 min. The reaction mixture was poured into water (50.0 mL). The organic layer was separated and the aqueous layer was extracted with AcOEt. The combined organic layer was dried over $MgSO_4$. The solvent was removed under reduced pressure. The residue was purified by preparative TLC on silica gel eluting with AcOEt-*n*-hexane (1:20) to give 3-(3-(2,3,4-

trimethoxyphenyl)oct-1-yn-1-yl)benzonitrile (**2z**) (21.2 mg, 26%) as a yellow oil and 3-(3-(3,4,5-trimethoxyphenyl)oct-1-yn-1-yl)benzonitrile (**2z**) (15.6 mg, 19%) as a yellow oil.

IR (KBr, cm^{-1}) ν 2955, 2929, 2856, 2232, 1599, 1494, 1478, 1466, 1417, 1278, 1256, 1095, 1043, 1018, 799, 756, 684; ^1H NMR (400 MHz, $CDCl_3$) δ 0.90 (3H, t, *J* = 6.8 Hz, CH_3), 1.30-1.35 (4H, m, CH_2x2), 1.43-1.54 (2H, m, CH_2), 1.71-1.79 (2H, m, CH_2), 3.86 (3H, s, OMe), 3.88 (3H, s, OMe), 3.94 (3H, s, OMe), 4.15 (1H, t, *J* = 7.8 Hz, CH), 6.69 (1H, d, *J* = 8.7 Hz, ArH), 7.16 (1H, d, *J* = 8.3 Hz, ArH), 7.40 (1H, d, *J* = 7.8 Hz, ArH), 7.46 (1H, dt, *J* = 8.2 and 1.8 Hz, ArH), 7.62 (1H, dt, *J* = 7.8 and 1.4 Hz, ArH), 7.70 (1H, d, *J* = 1.4 Hz, ArH); ^{13}C NMR (150 MHz, $CDCl_3$) δ 14.0 (q), 22.5 (t), 27.3 (t), 31.4 (t), 31.5 (d), 37.2 (t), 56.0 (q), 60.7 (q), 61.1 (q), 79.8 (s), 95.5 (s), 107.3 (d), 112.6 (s), 118.2 (s), 122.4 (s), 125.5 (s), 127.6 (s), 129.0 (d), 130.8 (d), 135.0 (d), 135.7 (d), 142.0 (s), 150.9 (s), 152.6 (s); EIMS m/z 377 (M^+), 362 (M^+ - Me); high resolution mass calcd for $C_{24}H_{27}NO_3$: 377.1991, found m/z 377.1989. Anal. Calcd for $C_{24}H_{27}NO_3$ (+1/4H_2O): C, 75.46; H, 7.26; N, 3.67 Found: C, 75.38; H, 7.24; N, 3.66.

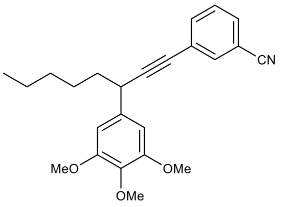

3-(3-(3,4,5-trimethoxyphenyl)oct-1-yn-1-yl)benzonitrile (**2z**)

IR (KBr, cm^{-1}) ν 2956, 2933, 2857, 2232, 1593, 1508, 1477, 1459, 1420, 1336, 1129, 1010, 685; ^1H NMR (400 MHz, $CDCl_3$) δ 0.90 (3H, t, *J* = 6.9 Hz, CH_3), 1.31-1.38 (4H, m, CH_2x2), 1.42-1.59 (2H, m, CH_2), 1.76-1.88 (2H, m, CH_2), 3.75 (1H, dd, *J* = 6.3 and 2.3 Hz, CH), 3.85 (3H, s, OMe), 3.89 (6H, s, OMex2), 6.60 (2H, s, ArH), 7.42 (1H, t, *J* = 8.0 Hz, ArH), 7.57 (1H, d, *J* = 8.1 Hz, ArH), 7.63 (1H, d, *J* = 7.4 Hz, ArH), 7.70 (1H, s, ArH); EIMS m/z 377 (M^+), 306 (M^+ -Me(CH_2)$_4$); high resolution mass calcd for $C_{24}H_{27}NO_3$: 377.1991, found m/z 377.1989.

Synthesis of 4-(3-(2,3,4-trimethoxyphenyl)but-1-yn-1-yl)benzonitrile (**2α**)

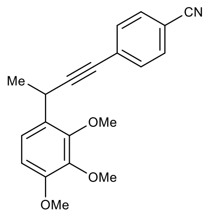

To a nitromethane (1.00 mL) solution of 4-(3-hydroxybut-1-yn-3-yl)benzonitrile (**1α**) (50.0 mg, 0.292 mmol), 1,2,3-trimethoxybenzene (148 mg, 0.811 mmol), and tetrabutylammonium hexafluorophosphate (22.6 mg, 0.0584 mmol), 1,1'-binaphthol (16.7 mg, 0.0584 mmol) were added silver perchlorate (24.2 mg, 0.117 mmol) and indium trichloride (12.4 mg, 0.0584 mmol) at room temperature. The reaction mixture was refluxed for 5 min. The reaction mixture was poured into water (50 mL). The organic layer was separated and the aqueous layer was extracted with ethyl acetate. The combined organic layer was dried over $MgSO_4$. The solvent was removed under reduced pressure. The residue was purified by preparative TLC on silica gel eluting with AcOEt-*n*-hexane (1:20) to give 4-(3-(2,3,4-trimethoxyphenyl)butt-1-yn-1-yl)benzonitrile (**2α**) (32.6 mg, 35%) as a yellow oil and 4-(3-(3,4,5-trimethoxyphenyl)but-1-yn-1-yl)benzonitrile (**2α**) (16.9 mg, 18%) as a yellow oil.

IR (KBr, cm^{-1}) ν 2974, 2936, 2835, 2226, 1603, 1493, 1466, 1417, 1282, 1257, 1224, 1100, 1029, 911, 840, 803, 557; ^1H NMR (600 MHz, $CDCl_3$) δ 1.51 (3H, d, *J* = 7.5 Hz, Me), 3.86 (3H, s, OMe), 3.88 (3H, s, OMe), 3.95 (3H, s, OMe), 4.29 (1H, q, *J* = 7.5 Hz, CH), 6.69 (1H, d, *J* = 8.5 Hz, ArH), 7.19 (1H, d, *J* = 8.6 Hz, ArH), 7.49 (2H, d, *J* = 8.6 Hz, ArH), 7.57 (2H, d, *J* = 8.6 Hz, ArH); ^{13}C NMR (150 MHz, $CDCl_3$) δ 23.2 (q), 26.2 (d), 56.0 (q), 60.7 (q), 61.1 (q), 80.0 (s), 98.5 (s), 107.3 (d), 110.9 (s), 118.6 (s), 121.7 (d), 128.5 (s), 128.9 (s), 131.9 (dx2), 132.1 (dx2), 142.1 (s), 150.8 (s), 152.7 (s); EIMS m/z 321 (M^+); high resolution mass calcd for $C_{20}H_{19}NO_3$: 321.1365, found m/z 321.1364. Anal. Calcd for $C_{20}H_{19}NO_3$(+3/7H_2O): C, 72.99; H, 6.08; N, 4.26. Found: C, 73.05; H, 5.78; N, 4.27.

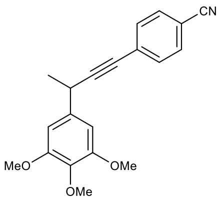

4-(3-(3,4,5-trimethoxyphenyl)but-1-yn-1-yl)benzonitrile (**2α**)

IR (KBr, cm^{-1}) ν 2932, 2852, 2227, 1593, 1463, 1128, 1332, 1290, 1236, 1128, 1010, 840, 761; ^1H NMR (600 MHz, $CDCl_3$) δ 1.59 (3H, t, *J* = 7.3 Hz, CH_3), 3.85 (3H, s, OMe), 3.89 (6H, s, OMex2), 6.64 (2H, s, ArH), 7.55 (2H, d, *J* = 7.1 Hz, ArH), 7.59 (2H, d, *J* = 6.9 Hz, ArH); EIMS m/z 321 (M^+); high resolution mass calcd for $C_{20}H_{19}NO_3$: 321.1365, found m/z 321.1351.

Synthesis of 1,2,3-trimethoxy-4-(4-(4-nitrophenyl)but-3-yn-2-yl)benzene (**2β**).

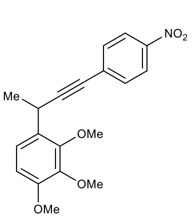

To a nitromethane (1.25 mL) solution of 4-(4-nitrophenyl)but-3-yn-2-ol (**1β**) (50.0 mg, 0.220 mmol), 1,2,3-trimethoxybenzene (132 mg, 0.785 mmol), and tetrabutylammonium hexafluorophosphate (20.3 mg, 0.0523 mmol), 1,1'-binaphthol (15.0 mg, 0.0523 mmol) were added silver perchlorate (21.7 mg, 0.105 mmol) and indium trichloride (11.6 mg, 0.0523 mmol) at room temperature. The reaction mixture was stirred at 40 °C for 12h. The reaction mixture was poured into water (50 mL). The organic layer was separated and the aqueous layer was extracted with ethyl acetate. The combined organic layer was dried over $MgSO_4$. The solvent was removed under reduced pressure. The residue was purified by preparative TLC on silica gel eluting with AcOEt-*n*-hexane (1:20) to give 1,2,3-trimethoxy-4-(4-(4-nitrophenyl)but-3-yn-2-yl)benzene (**1β**) (8.1 mg, 9%) as a pale-yellow prism (mp = 47~49 °C) and 1,2,3-trimethoxy-5-(4-(4-nitrophenyl)but-3-yn-2-yl)benzene (**1β**) (14.6 mg, 16%) as a yellow oil.

IR (KBr, cm^{-1}) ν 2974, 2935, 2228, 1595, 1520, 1494, 1467, 1417, 1344, 1297, 1283, 1100, 1029, 853, 750; ^{1}H NMR (600 MHz, $CDCl_3$) δ 1.54 (3H, d, *J* = 6.9 Hz, CH_3), 3.87 (3H, s, OMe), 3.89 (3H, s, OMe), 3.96 (3H, s, OMe), 4.31 (1H, q, *J* = 6.9 Hz, CH), 6.70 (1H, d, *J* = 8.6 Hz, ArH), 7.20 (1H, d, *J* = 8.6 Hz, ArH), 7.55 (2H, d, *J* = 9.1 Hz, ArH), 8.16 (2H, d, *J* = 9.2 Hz, ArH); ^{13}C NMR (150 MHz, $CDCl_3$) δ 23.2 (q), 26.2 (d), 56.0 (q), 60.7 (q), 61.2 (q), 79.8 (s), 99.6 (s), 107.3 (d), 121.8 (d), 123.5 (dx2), 128.3 (s), 130.9 (s), 132.3 (dx2), 142.0 (s), 146.6 (s), 150.8 (s), 152.8 (s); EIMS m/z 341 (M^+), 326 (M^+-Me); high resolution mass calcd for $C_{20}H_{21}ClO_3$: 341.1263, found m/z 341.1241. Anal. Calcd for $C_{19}H_{19}NO_5$ (+1/3H_2O): C, 65.70; H, 5.71; N, 4.03 Found: C, 65.53; H, 5.61; N, 4.05.

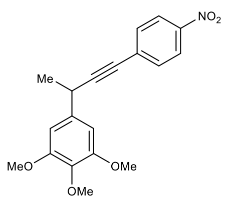

Synthesis of 1,2,3-trimethoxy-5-(4-(4-nitrophenyl)but-3-yn-2-yl)benzene (**1β**)

IR (KBr, cm^{-1}) ν 2935, 2364, 2227, 1718, 1594, 1519, 1458, 1420, 1344, 1236, 1129, 852, 751; ^{1}H NMR (400 MHz, $CDCl_3$) δ 1.61 (3H, d, *J* = 7.3 Hz, CH_3), 3.85 (3H, s, OMe), 3.89 (6H, s, OMex2), 3.96 (1H, q, *J* = 7.3 Hz, CH), 6.64 (2H, s, ArH), 7.56 (2H, d, *J* = 8.2 Hz, ArH), 8.17 (2H, d, *J* = 8.7 Hz, ArH); EIMS m/z 341 (M^+), 326 (M^+-Me); high resolution mass calcd for $C_{20}H_{21}ClO_3$: 341.1263, found m/z 341.1241.

Synthesis of 1,2,3-trimethoxy-4-(1-(2-methyl-5-nitrophenyl)oct-1-yn-3-yl)benzene (**2γ**)

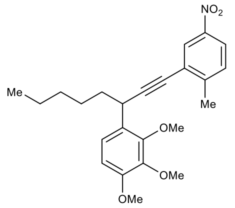

To a nitromethane (0.90 mL) solution of 1-(2-methyl-5-nitrophenyl)oct-1-yn-3-ol (**1γ**) (50.0 mg, 0.191 mmol), 1,2,3-trimethoxybenzene (96.5 mg, 0.574 mmol), and tetrabutylammonium hexafluorophosphate (14.8 mg, 0.0383 mmol), 1,1'-binaphthol (11.0 mg, 0.0383 mmol) were added silver perchlorate (15.9 mg, 0.0765 mmol) and indium trichloride (8.50 mg, 0.0383 mmol) at room temperature. The reaction mixture was stirred at 40 ºC for 6h. The reaction mixture was poured into water (50 mL). The organic layer was separated and the aqueous layer was extracted with ethyl acetate. The combined organic layer was dried over $MgSO_4$. The solvent was removed under reduced pressure. The residue was purified by preparative TLC on silica gel eluting with AcOEt-*n*-hexane (1:20) to give 1,2,3-trimethoxy-4-(1-(2-methyl-5-nitrophenyl)oct-1-yn-3-yl)benzene (**2γ**) (42.6 mg, 54%) as a yellow oil and 1,2,3-trimethoxy-5-(4-(2-methyl-5-nitrophenyl)oct-1-yn-3-yl)benzene (**2γ**) (18.2 mg, 23%) as a yellow oil.

IR (KBr, cm^{-1}) ν 2931, 2857, 2227, 1600, 1523, 1494, 1465, 1417, 1349, 1278, 1095, 1041, 1018, 903, 797, 740; ^{1}H NMR (600 MHz, $CDCl_3$) δ 0.90 (3H, t, *J* = 6.9 Hz, Me), 1.32-1.38 (4H, m, CH_2), 1.45-1.63 (2H, m, CH_2), 1.78 (2H, q, *J* = 7.5 Hz, CH_2), 2.53 (3H, s, Me), 3.87 (3H, s, OMe), 3.89 (3H, s, OMe), 3.95 (3H, s, OMe), 4.23 (1H, t, *J* = 6.9 Hz, CH), 6.70 (1H, d, *J* = 6.9 Hz, ArH), 7.20 (1H, d, *J* = 8.6 Hz, ArH), 7.33 (1H, d, *J* = 8.0 Hz, ArH), 8.01 (1H, dd, *J* = 2.9 and 8.6 Hz, ArH), 8.23 (1H, d, *J* = 2.9 Hz, ArH); ^{13}C NMR (150 MHz, $CDCl_3$) δ 14.0 (q), 21.2 (q), 22.5 (t), 27.3 (t), 31.4 (t), 31.6 (d), 37.5 (t), 56.0 (q), 60.7 (q), 61.2 (q), 79.0 (s), 99.5 (s), 107.3 (d), 122.2 (d), 122.4 (d), 125.3 (s), 126.7 (d), 127.7 (s), 130.0 (d), 142.0 (s), 146.0 (s), 147.6 (s), 150.8 (s), 152.6 (s); EIMS m/z 411 (M^+), 396 (M^+-Me), 340 (M^+-$(CH_2)_4$Me); high resolution mass calcd for $C_{24}H_{29}NO_5$: 411.2046, found m/z 411.2042. Anal. Calcd for $C_{24}H_{29}NO_5$(+1/10H_2O): C, 69.75; H, 7.12; N, 3.39. Found: C, 69.73; H, 6.90; N, 3.42.

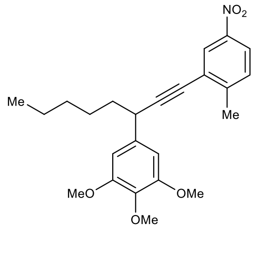

1,2,3-trimethoxy-5-(4-(2-methyl-5-nitrophenyl)oct-1-yn-3-yl)benzene (**2γ**)

IR (KBr, cm^{-1}) ν 2933, 2858, 2228, 1592, 1489, 1420, 1348, 1236, 1129, 1010, 829, 740; ^{1}H NMR (600 MHz, $CDCl_3$) δ 0.91 (3H, t, *J* = 6.9 Hz, Me), 1.32-1.38 (4H, m, CH_2), 1.44-1.63 (2H, m, CH_2), 1.82-1.88 (2H, m, CH_2), 2.54 (3H, s, Me), 3.82 (1H, q, *J* = 7.5 Hz, CH), 3.85 (3H, s, OMe), 3.89 (6H, s, OMex2), 6.63 (2H, s, ArH), 7.35 (1H, d, *J* = 8.2 Hz, ArH), 8.03 (1H. dd, *J* = 2.3 and 8.2 Hz, ArH), 8.24 (1H, d, *J* = 2.3 Hz, ArH); EIMS m/z 411 (M^+); high resolution mass calcd for $C_{24}H_{29}NO_5$: 411.2046, found m/z 411.2048.

Synthesis of 1-(dodec-5-yn-4-yl)-2,3,4-trimethoxybenzene (**2δ**).

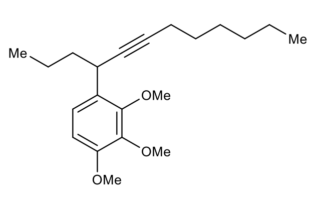

To a nitromethane (1.00 mL) solution of dodec-5-yn-4-ol (**1δ**) (50.0 mg, 0.274 mmol), 1,2,3-trimethoxybenzene (138 mg, 0.822 mmol), and tetrabutylammonium hexafluorophosphate (21.2 mg, 0.0548 mmol) were added silver perchlorate (28.4 mg, 0.137 mmol)bis(trifluoromethanesulfonyl)imide (30.9 mg, 0.110 mmol) and indium trichloride (12.1 mg, 0.0548 mmol) at room temperature. The reaction mixture was stirred for 0.5 h. The reaction mixture was poured into water (50.0 mL). The organic layer was separated and the aqueous layer was extracted with AcOEt. The combined organic layer was dried over $MgSO_4$. The solvent was removed under reduced pressure. The residue was purified by preparative TLC on silica gel eluting with AcOEt-*n*-hexane (1:20) to give a yellow oil. The titled compound was precipitated from *n*-hexane to give 1-(dodec-5-yn-4-yl)-2,3,4-trimethoxybenzene (**2δ**) (34.0 mg, 37%) as a yellow oil and 5-(dodec-5-yn-4-yl)-1,2,3-trimethoxybenzene (**2δ**) (18.9 mg, 21%) as a yellow oil.

IR (KBr, cm^{-1}) ν 2957, 2931, 2857, 1601, 1494, 1465, 1416, 1286, 1038, 1020, 797; ^{1}H NMR (600 MHz, $CDCl_3$) δ 0.89 (3H, t, *J* = 7.6 Hz, CH_3), 0.92 (3H, t, *J* = 7.6 Hz, CH_3), 1.28-1.32 (4H, m, CH_2), 1.37-1.44 (2H, m, CH_2), 1.49-1.54 (2H, m, CH_2), 1.58-1.61 (2H, m, CH_2), 2.20 (2H, dt, *J* = 2.0 and 6.8 Hz, CH_2), 3.84 (3H, s, OCH_3), 3.86 (3H, s, O CH_3), 3.89 (3H, s, O CH_3), 6.66 (1H, d, *J* = 9.0 Hz, ArH), 7.17 (1H, d, *J* = 8.9 Hz, ArH); ^{13}C NMR (150 MHz, $CDCl_3$) δ 13.8 (q), 14.0 (q), 18.8 (t), 20.7 (t), 22.6 (t), 28.5 (t), 29.1 (t), 30.7 (d), 31.3 (t), 40.2 (t), 55.9 (q), 60.7 (q), 61.0 (q), 82.0 (s), 82.3 (s), 107.2 (d), 122.5 (d), 129.2 (s), 141.9 (s), 150.8 (s), 152.2 (s); MS m/z 332 (M^+); high resolution mass calcd for $C_{21}H_{32}O_3$: 332.2351, found m/z 332.2346.

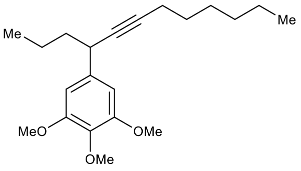

Synthesis of 5-(dodec-5-yn-4-yl)-1,2,3-trimethoxybenzene (**2δ**).

IR (KBr, cm^{-1}) ν 2932, 2858, 1953, 1508, 1464, 1419, 1335, 1233, 1129, 1013, 758; ^{1}H NMR (600 MHz, $CDCl_3$) δ (3H, t, *J* = 6.9 Hz, CH_3), 0.94 (3H, t, *J* = 7.7 Hz, CH_3), 1.26-1.42 (8H, m, CH_2), 1.44-1.53 (2H, m, CH_2), 1.66-1.69 (2H, m, CH_2), 2.20 (2H, dt, *J* = 2.3 and 7.3 Hz, CH_2), 3.86 (3H, s, OCH_3), 3.87 (6H, s, OCH_3×2), 4.35 (1H, brd, *J* = 4.0 Hz, CH), 6.58 (2H, brs, ArH); MS m/z 332 (small M^+).

Synthesis of 1,2,3-trimethoxy-4-(tetracos-13-yn-12-yl)benzene (**2ε**).

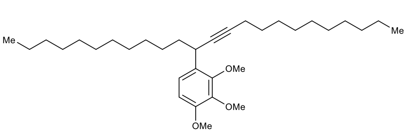

To a nitromethane (1.00 mL) solution of tetracos-13-yn-12-ol (**1ε**) (50.0 mg, 0.143 mmol), 1,2,3-trimethoxybenzene (36.2 mg, 0.215 mmol), and tetrabutylammonium hexafluorophosphate (11.0 mg, 0.0548 mmol) were added silver perchlorate (14.8 mg, 0.0715 mmol) and indium trichloride (6.30 mg, 0.0286 mmol) at room temperature. The reaction mixture was stirred for 2 h at 50 ºC. The reaction mixture was filtered through a pad of celite with chloroform and then poured into a saturated sodium hydrogencarbonate. The organic layer was separated and the aqueous layer was extracted with chloroform. The combined organic layer was dried over $MgSO_4$. The solvent was removed under reduced pressure. The residue was purified by preparative TLC on silica gel eluting with AcOEt-*n*-hexane (1:30) to give a yellow oil. The titled compound was precipitated from *n*-hexane to give 1,2,3-trimethoxy-4-(tetracos-13-yn-12-yl)benzene (**2ε**) (21.1 mg, 30%) as a yellow oil.

IR (KBr, cm^{-1}) ν 2925, 2854, 16021494, 1466, 1416, 1283, 1254, 1038, 1020, 797, 757, 721; ^{1}H NMR (600 MHz, $CDCl_3$) δ 0.81 (6H, t, *J* = 6.8 Hz, CH_2×2), 1.18 (30H, brs CH_2), 1.30-1.35 (2H, m, CH_2), 1.40-1.47 (2H, m, CH_2), 1.49-1.58 (2H, m, CH_2), 2.20 (2H, dt, *J* = 2.0 and 6.9 Hz, CH_2), 3.84 (3H, s, OCH_3), 3.86 (3H, s, OCH_3), 3.87 (1H, t, *J* = 4.0 Hz, CH), 3.89 (3H, s, OCH_3), 6.65 (2H, d, *J* = 8.2 Hz, ArH), 7.17 (2H, d, *J* = 8.2 Hz, ArH); ^{13}C NMR (150 MHz, $CDCl_3$) δ 14.1 (qx2), 18.8 (t), 22.7 (tx2), 27.6 (t), 28.9 (t), 29.1 (t), 29.2 (t), 29.3 (t), 29.4 (tx2), 29.6 (t), 29.7 (tx2), 29.8 (tx2), 29.9 (t), 30.1 (d), 31.9 (tx2), 38.0 (t), 55.9 (q), 60.7 (q), 61.0 (q), 82.0 (s), 82.4 (s), 107.2 (d), 122.5 (d), 129.3 (s), 141.9 (s), 150.8 (s), 152.2 (s); MS m/z 550 (M^+); high resolution mass calcd for $C_{33}H_{56}O_3$: 500.4223, found m/z 500.4230.
Anal. Calcd for $C_{33}H_{56}O_3$: C, 79.14; H, 11.27. Found: C, 79.31; H, 11.16.

Synthesis of 1,2,3-trimethoxy-4-(1-(phenylethynyl)cyclopentyl)benzene (**2ζ**).

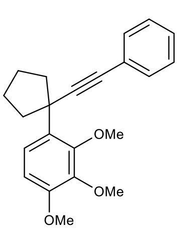

To a nitromethane (1.50 mL) solution of 1-(phenylethynyl)cyclopentan-1-ol (**1ζ**) (50.0 mg, 0.268 mmol), 1,2,3-trimethoxybenzene (135 mg, 0.805 mmol), tetrabutylammonium hexafluorophosphate (20.8 mg, 0.0536 mmol), and 1,1'-binaphthol (15.3 mg, 0.0536 mmol) were added silver perchlorate (30.0 mg, 0.145 mmol) and indium trichloride (11.9 mg, 0.0536 mmol) at room temperature. The reaction mixture was stirred for 1 h and then poured into water (50 mL). The organic layer was separated and the aqueous layer was extracted with AcOEt. The combined organic layer was dried over $MgSO_4$. The solvent was removed under reduced pressure. The residue was purified by preparative TLC on silica gel eluting with AcOEt-*n*-hexane (1:5) to give 1,2,3-trimethoxy-4-(1-(phenylethynyl)cyclopentyl)benzene (**2ζ**) (35.2 mg, 39%) as a yellow oil, (cyclopent-1-en-1-ylethynyl)benzene (**4ζ**) (27.1 mg, 60%) as a yellow oil.

IR (KBr, cm^{-1}) ν 3446, 2936, 2855, 1714, 1593, 1493, 1466, 1412, 1348, 1219, 1119, 1045, 845, 762, 700;^{1}H NMR (600 MHz, $CDCl_3$) δ 1.80 (2H, brs, CH_2), 1.77-1.82 (4H, m, CH_2), 2.43-2.47 (2H, m, CH_2), 3.85 (3H, s, OCH_3), 3.88 (3H, s, OCH_3), 4.01 (3H, s, OCH_3), 6.60 (1H, d, *J* = 8.2 Hz, ArH), 7.12 (1H, d, *J* = 9.0 Hz, ArH), 7.24-7.26 (3H, m, ArH), 7.34-7.37 (2H, m, ArH);^{13}C NMR (150 MHz, $CDCl_3$) δ 23.9 (t×2), 40.3 (t×2), 45.2 (s), 56.0 (q), 60.6 (q), 60.7 (q), 80.6 (s), 97.5 (s), 106.0 (d), 121.1 (d), 124.5 (s), 127.4 (d), 128.2 (d×2), 131.3 (s), 131.5 (d×2), 142.9 (s), 152.87 (s), 152.9 (s); MS m/z 336 (M^+), 321 (M^+-Me), 305 (M^+-OMe); high resolution mass calcd for $C_{22}H_{24}O_3$: 336.1725, found m/z 336.1725, found m/z 336.1741. Anal. Calcd for $C_{22}H_{24}O_3$ (+0.12xH_2O): C, 78.04; H, 7.22. Found: C, 77.91; H, 7.32.

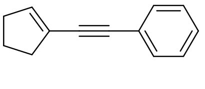

IR (KBr, cm^{-1}) ν 2955, 2926, 2847, 2202, 1714, 1672, 1594, 1491, 1442, 1361, 1219, 756, 691;^{1}H NMR (600 MHz, $CDCl_3$) δ 1.90-1.98 (2H, m, CH_2), 2.44-2.50 (2H, m, CH_2), 2.52-2.58 (2H, m, CH_2), 6.13-6.15 (1H, m, olefinic H), 7.27-7.33 (3H, m, ArH), 7.40-7.45 (2H, m, ArH); MS m/z 153 (M^+-Me).

Synthesis of 1,2,3-trimethoxy-4-(1-(phenylethynyl)cyclohexyl)benzene (**2η**).

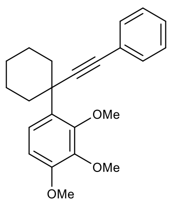

To a nitromethane (1.00 mL) solution of 1-(phenylethynyl)cyclohexan-1-ol (**1η**) (50.0 mg, 0.250 mmol), 1,2,3-trimethoxybenzene (63.1 mg, 0.375 mmol), and tetrabutylammonium hexafluorophosphate (19.4 mg, 0.0500 mmol) were added silver perchlorate (25.9 mg, 0.125 mmol) and indium trichloride (11.1 mg, 0.0500 mmol) at room temperature. The reaction mixture was stirred for 40 min. The reaction mixture was filtered through a pad of celite with chloroform and then poured into a saturated sodium hydrogencarbonate. The organic layer was separated and the aqueous layer was extracted with chloroform. The combined organic layer was dried over $MgSO_4$. The solvent was removed under reduced pressure. The residue was purified by preparative TLC on silica gel eluting with AcOEt-n-hexane (1:40) to give 1,2,3-trimethoxy-4-(1-(phenylethynyl)cyclohexyl)benzene (**2η**) (85.5 mg, 78%) as a white crystal (mp 100-103 °C from chloroform-*n*-hexane), 5',6',7'-trimethoxy-3'-phenylspiro[cyclohexane-1,1'-indene] (**5η**) (7.4 mg, 7%) as a colorless oil.

IR (KBr, cm^{-1}) ν 2932, 2855, 1715, 1598, 1493, 1460, 1411, 1107, 1068, 912, 795, 757, 692; ^1H NMR (600 MHz, $CDCl_3$) δ 1.23-1.25 (1H, m, CH), 1.70-1.81 (5H, m, CH_2), 1.94-1.98 (2H, m, CH_2), 2.23 (2H, d, *J* = 12.3 Hz, CH_2), 3.85 (3H, s, OCH_3), 3.87 (3H, s, OCH_3), 3.99 (3H, s, OCH_3), 6.62 (1H, d, *J* = 9.0 Hz, ArH), 7.19 (1H, d, *J* = 8.9 Hz, ArH), 7.25-7.29 (3H, m, ArH), 7.44 (2H, brd, *J* = 6.2 Hz, ArH); ^{13}C NMR (150 MHz, $CDCl_3$) δ 23.5 (t×2), 26.0 (t), 37.1 (t×2), 40.0 (s), 55.8 (q), 60.4 (q), 60.7 (q), 83.9 (s), 95.5 (s), 106.0 (d), 120.6 (d), 124.5 (s), 127.3 (d), 128.1 (d×2), 131.4 (d×2), 132.4 (s), 142.9 (s), 152.6 (s), 153.1 (s); EIMS m/z 350 (M^+), 307 (M^+ -Pr), 293 (M^+ -Bu), 267 (M^+ -C_6H_{11}); high resolution mass calcd for $C_{28}H_{26}O_3$: 350.1882, found m/z 350.1863. Anal. Calcd for $C_{28}H_{26}O_3$ (+ 2 / 5 H_2O): C, 77.24; H, 7.55. Found: C, 77.14; H, 7.47.

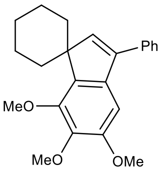

5',6',7'-trimethoxy-3'-phenylspiro[cyclohexane-1,1'-indene] (**5η**)

IR (KBr, cm^{-1}) ν 2929, 2856, 2363, 1601, 1468, 1412, 1198, 1119, 1046, 978, 841, 698; ^1H NMR (600 MHz, $CDCl_3$) δ 1.42-1.55 (3H, m, CH_2), 1.59-1.66 (2H, m, CH_2), 1.78-1.89 (5H, m, CH_2), 3.41 (3H, s, OCH_3),3.87 (3H, s, OCH_3) 3.95 (3H, s, OCH_3), 6.61 (1H, s, ArH), 6.74 (1H, s, ArH), 7.30-7.31 (1H, m, ArH), 7.35-7.38 (1H, m, ArH), 7.54 (2H, brd, *J* = 6.8 Hz, ArH); MS m/z 350 (M^+); high resolution mass calcd for $C_{23}H_{26}O_3$: 350.1882, found m/z 350.1951.

Synthesis of *(E)*-1-(phenylethynyl)cyclooct-1-ene (**4θ**).

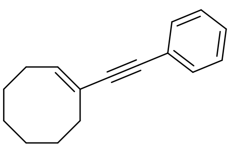

To a nitromethane (2.00 mL) solution of 1-(phenylethynyl)cyclooctan-1-ol (**1θ**) (50.0 mg, 0.219 mmol), 1,2,3-trimethoxybenzene (111 mg, 0.657 mmol), and tetrabutylammonium hexafluorophosphate (17.0 mg, 0.0438 mmol) were added silver perchlorate (22.8 mg, 0.110 mmol) and indium trichloride (9.69 mg, 0.0438 mmol). The reaction mixture was stirred at room tempreture for 30 min and then poured into water (50 mL). The organic layer was separated and the aqueous layer was extracted with AcOEt. The combined organic layer was dried over $MgSO_4$ and the solvent was removed under reduced pressure. The residue was purified by preparative TLC on silica gel eluting with AcOEt-*n*-hexane (1:10) to give (*E*)-1-(ohenykethynyl)cyclooct-1-ene (**4θ**) (32.5 mg, 71%) as a yellow oil.

IR (KBr, cm^{-1}) ν 3436, 2926, 2851, 1715, 1596, 1489, 1466, 1443, 755, 690; ^1H NMR (600 MHz, $CDCl_3$) δ 1.53-1.57 (6H, m, CH_2), 1.64-1.68 (2H, m, CH_2), 2.21-2.24 (2H, m, CH_2), 2.39 (2H, t, *J* = 6.2 and 6.8 Hz, CH_2), 6.19 (1H, t, *J* = 8.2 Hz, olefinic H), 7.25-7.30 (3H, m, ArH), 7.41-7.43 (2H, m, ArH); ^{13}C NMR (150 MHz, $CDCl_3$) δ 25.9 (t), 26.5 (t), 27.2 (t), 28.6 (t), 29.8 (t), 30.1 (t), 86.4 (s), 92.2 (s), 123.9 (s), 124.0 (s), 127.7 (d), 128.3 (d×2), 131.5 (d×2), 138.1 (d); EIMS m/z 210 (M^+), 195 (M^+ -CH_3); high resolution mass calcd for $C_{16}H_{18}$: 210.1409, found m/z 210.1389.

Synthesis of 1,2-diethyl-4,5,6-trimethoxy-3-phenyl-1-1*H*-indene (**5ι**).

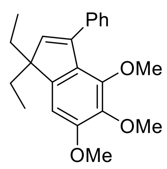

To a nitromethane (1.50 mL) solution of 3-ethyl-1-phenylpent-1-yn-3-ol (**1ai**) (58.7 mg, 0.312 mmol), 1,2,3-trimethoxybenzene (162 mg, 0.963 mmol), 1,1'-binaphthol (18.0 mg, 0.0625 mmol) and tetrabutylammonium hexafluorophosphate (24.2 mg, 0.0624 mmol) were added silver perchlorate (35.0 mg, 0.125 mmol) and indium trichloride (14.0 mg, 0.0624 mmol) at room temperature. The reaction mixture was stirred for 5 h. The reaction mixture was filtered through a pad of celite chloroform and then poured into a saturated sodium hydrogencarbonate. The organic layer was separated and the aqueous layer was extracted with chloroform. The combined organic layer was dried over $MgSO_4$. The solvent was removed under reduced pressure. The residue was purified by preparative TLC on silica gel eluting with AcOEt-*n*-hexane (1:10) to give 1,2-diethyl-4,5,6-trimethoxy-3-phenyl-1-1*H*-indene (**5ι**) (37.9 mg, 36%) as a yellow oil.

IR (KBr, cm^{-1}) ν 2594, 2386, 2315, 1375, 1300, 1199, 1002, 761, 724, 700; ^1H NMR (600 MHz, $CDCl_3$) δ 0.65 (6H, t, *J* = 7.5 Hz, CH_3×2), 169-1.75 (2H, m, CH_2), 1.85-1.91 (2H, m, CH_2), 3.39 (3H, s, OCH_3), 3.87 (3H, s, OCH_3), 3.90 (3H, s, OCH_3), 6.02 (1H, s, olefinic H), 6.61 (1H, s, ArH), 7.30-7.31 (1H, m, ArH), 7.35-7.37 (2H, m, ArH), 7.53-7.54 (2H, m, ArH); MS m/z 338 (M^+); high resolution mass calcd for $C_{22}H_{26}O_3$: 338.1882, found m/z 338.1863. Anal. Calcd for $C_{22}H_{26}O_3$ (+1/10H_2O): C, 77.66; H, 7.76. Found: C, 77.48; H, 7.77.

Synthesis of 1,2,3-trimethoxy-4-(1-(2-nitrophenyl)-3-phenylprop-2-yn-1-yl)benzene (**2κ**).

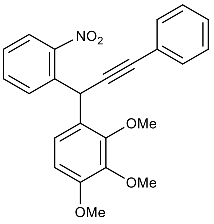

To a nitromethane (1.00 mL) solution of 1-(2-nitrophenyl)-3-phenylprop-2-yn-1-ol (**1κ**) (50.0 mg, 0.198 mmol), 1,2,3-trimethoxybenzene (66.6 mg, 0.396 mmol), tetrabutylammonium hexafluorophosphate (15.3 mg, 0.0396 mmol), 1,1'-binaphthol (15.3 mg, 3.96x10^{-5} mol) were added silver perchlorate (20.5 mg, 0.0990 mmol) and indium trichloride (8.80 mg, 3.80x10^{-5} mol) at room temperature. The reaction mixture was stirred for 1 h. The reaction mixture was poured into water (50.0 mL). The organic layer was separated and the aqueous layer was extracted with AcOEt. The combined organic layer was dried over $MgSO_4$. The solvent was removed under reduced pressure. The residue was purified by preparative TLC on silica gel eluting with AcOEt-chloroform-*n*-hexane (1:1:50) to give 1,2,3-trimethoxy-4-(1-(2-nitrophenyl)-3-phenylprop-2-yn-1-yl)benzene (**2κ**) (51.8 mg, 65%) as a yellow oil, and 1,2,3-trimethoxy-5-(1-(4-nitrophenyl)-3-phenylprop-2-yn-1-yl)benzene (**2κ**) (4.40 mg, 6%) as a yellow oil.

IR (KBr, cm^{-1}) ν 2940, 2836, 2221, 1713, 1599, 1528, 1492, 1466, 1417, 1354, 1280, 1096, 1013, 856, 759, 729, 692; ^{1}H NMR (600 MHz, $CDCl_3$) δ 3.68 (3H, s, OMe), 3.80 (3H, s, OMe), 3.85 (3H, s, OMe), 6.19 (1H, s, CH), 6.66 (1H, d, *J* = 8.6 Hz, ArH), 7.28-7.30 (4H, m, ArH), 7.35 (1H, brt, *J* = 6.9 Hz, ArH), 7.44-7.47 (2H, m, ArH), 7.51 (1H, t, *J* = 7.5 Hz, ArH), 7.60 (1H, d, *J* = 8.9 Hz, ArH), 7.86 (1H, brd, *J* = 8.0 Hz, ArH); ^{13}C NMR (150 MHz, $CDCl_3$) δ 33.3 (d), 55.9 (q), 60.3 (q), 60.6 (q), 84.8 (s), 88.7 (s), 106.6 (d), 122.6 (d), 123.1 (s), 124.4 (d), 125.8 (s), 127.5 (d), 128.1 (d), 128.2 (dx2), 130.1 (d), 131.7 (dx2), 132.7 (d), 136.5 (s), 142.0 (s), 148.6 (s), 151.0 (s), 153.5 (s); EIMS m/z 403 (M^+); high resolution mass calcd for $C_{24}H_{21}NO_5$: 403.1420, found m/z 403.1409. Anal. Calcd for $C_{24}H_{21}NO_5$(+1/5H_2O): C, 70.82; H, 5.30; N, 3/44. Found: C, 70.52; H, 5.28; N, 3.47.

Synthesis of 1,2,3-trimethoxy-4-(1-(4-nitrophenyl)-3-phenylprop-2-yn-1-yl)benzene (**2λ**).

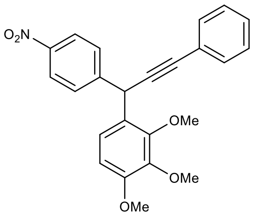

To a nitromethane (1.00 mL) solution of 1-(4-nitrophenyl)-3-phenylprop-2-yn-1-ol (**1λ**) (50.0 mg, 0.19 mmol), 1,2,3-trimethoxybenzene (50.0 mg, 0.297 mmol), and tetrabutylammonium hexafluorophosphate (15.3 mg, 0.0396 mmol) were added silver perchlorate (20.5 mg, 0.0990 mmol), 1,1'-binaphthol (14.7 mg, 3.80x10^{-5} mol) and indium trichloride (8.70 mg, 3.80x10^{-5} mol) at room temperature. The reaction mixture was stirred for 30 min. The reaction mixture was filtered through a pad of celite chloroform and then poured into a saturated sodium hydrogencarbonate. The organic layer was separated and the aqueous layer was extracted with chloroform. The combined organic layer was dried over $MgSO_4$. The solvent was removed under reduced pressure. The residue was purified by preparative TLC on silica gel eluting with AcOEt-*n*-hexane (1:10) to give 1,2,3-trimethoxy-4-(1-(4-nitrophenyl)-3-phenylprop-2-yn-1-yl)benzene (**2λ**) (58.5 mg, 74%) as a yellow oil, and 1,2,3-trimethoxy-5-(1-(4-nitrophenyl)-3-phenylprop-2-yn-1-yl)benzene (**2λ**) (9.80 mg, 12%) as a yellow oil.

IR (KBr, cm^{-1}) ν 2934, 1598, 1520, 1492, 1466, 1281, 1254, 1096, 1014, 857, 759, 693; ^{1}H NMR (600 MHz, $CDCl_3$) δ 3.82 (3H, s, OCH_3), 3.85 (6H, s, OCH_3×2), 5.60 (1H, s, CH), 6.69 (1H, d, *J* = 8.9 Hz, ArH), 7.24 (1H, d, *J* = 9.0 Hz, ArH), 7.31-7.32 (3H, m, ArH), 7.46-7.47 (2H, m, ArH), 7.61 (2H, d, *J* = 8.9 Hz, ArH), 8.16 (2H, d, *J* = 8.3 Hz, ArH); ^{13}C NMR (150 MHz, $CDCl_3$) δ 37.2 (d), 36.0 (q), 60.7 (q), 60.8 (q), 85.0 (s), 89.0 (s), 107.3 (d), 112.9 (s), 123.1 (d), 123.7 (d×2), 126.2 (s), 128.3 (d×3), 128.6 (d×2), 131.6 (d×2), 142.1 (s), 146.7 (s), 149.5 (s), 150.8 (s), 153.4 (s); MS m/z 403 (M^+), 388 (M^+-Me), 279 (M^+-$O_2NC_6H_4$); high resolution mass calcd for $C_{24}H_{21}NO_5$: 403.1419, found m/z 403.1409. Anal. Calcd for $C_{24}H_{21}NO_5$: C, 71.45; H, 5.25; N, 3.47. Found: C, 71.18; H, 5.42; N, 3.40.

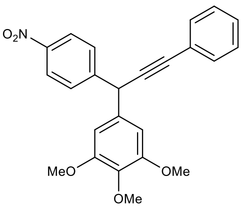

1,2,3-trimethoxy-5-(1-(4-nitrophenyl)-3-phenylprop-2-yn-1-yl)benzene (**2λ**)

IR (KBr, cm^{-1}) ν 2935, 1712, 1593, 1520, 1491, 1463, 1421, 1247, 1238, 1128, 1013, 857, 804, 759, 710, 692; ^{1}H NMR (500 MHz, $CDCl_3$) δ 3.84 (3H, s, OCH_3), 3.85 (6H, s, OCH_3×2), 5.23 (1H, s, CH), 6.63 (2H, s, ArH), 7.34 (3H, dd, *J* = 3.7 and 1.7 Hz, ArH), 7.47-7.48 (2H, m, ArH), 7.62 (2H, d, *J* = 8.5 Hz, ArH), 8.21 (2H, d, *J* = 8.6 Hz, ArH); MS m/z 403 (M^+), 388 (M^+-Me); high resolution mass calcd for $C_{24}H_{21}NO_5$: 403.1420, found m/z 403.1409.

Synthesis of 1,2,3-trimethoxy-4-(1-(3-nitrophenyl)-3-phenylprop-2-yn-1-yl)benzene (**2μ**).

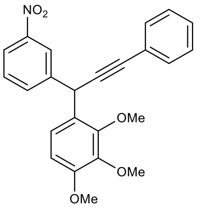

To a nitromethane (1.00 mL) solution of 1-(4-nitrophenyl)-3-phenylprop-2-yn-1-ol (**1μ**) (50.0 mg, 0.198 mmol), 1,2,3-trimethoxybenzene (66.6 mg, 0.396 mmol), and tetrabutylammonium hexafluorophosphate (15.3 mg, 0.0396 mmol), 1,1'-binaphthol (15.3 mg, 3.96x10^{-5} mol) were added silver perchlorate (20.5 mg, 0.0990 mmol) and indium trichloride (8.70 mg, 3.80x10^{-5} mol) at room temperature. The reaction mixture was stirred for 1 h. The reaction mixture was poured into water (50.0 mL). The organic layer was separated and the aqueous layer was extracted with AcOEt. The combined organic layer was dried over $MgSO_4$. The solvent was removed under reduced pressure. The residue was purified by preparative TLC on silica gel eluting with AcOEt-chloroform-*n*-hexane (1:1:50) to give 1,2,3-trimethoxy-4-(1-(3-nitrophenyl)-3-phenylprop-2-yn-1-yl)benzene (**2μ**) (70.2 mg, 88%) as a yellow oil, and 1,2,3-trimethoxy-5-(1-(4-nitrophenyl)-3-phenylprop-2-yn-1-yl)benzene (**2μ**) (4.40 mg, 6%) as a yellow oil.

IR (KBr, cm^{-1}) ν 2939, 2835, 1598, 1530, 1492, 1466, 1417, 1350, 1281, 1254, 1012, 805, 758, 729, 692; ^{1}H NMR (600 MHz, $CDCl_3$) δ 3.83 (3H, s, OMe), 3.86 (3H, s, OMe), 5.61 (1H, s, CH), 6.69 (1H, d, J = 8.9 Hz, ArH), 7.26 (1H, d, J = 8.9 Hz, ArH), 7.31-7.32 (3H, m, ArH), 7.46-7.48 (3H, m, ArH), 7.78 (1H, d, J = 7.5 Hz, ArH), 8.07 (1H, d, J = 8.3 Hz, ArH), 8.33 (1H, s, ArH); ^{13}C NMR (150 MHz, $CDCl_3$) δ 37.0 (d), 56.0 (q), 60.7 (q), 60.8 (q), 85.0 (s), 89.1 (s), 107.3 (d), 121.8 (d), 122.8 (d), 123.0 (s), 123.1 (d), 126.3 (s), 128.2 (d), 128.3 (dx2), 129.2 (d), 131.6 (dx2), 134.0 (d), 142.1 (s), 144.3 (s), 148.3 (s), 150.8 (s), 153.4 (s); EIMS m/z 403 (M^+); high resolution mass calcd for $C_{24}H_{21}NO_5$: 403.1420, found m/z 403.1432. Anal. Calcd for $C_{24}H_{21}NO_5$ (+1.7H_2O): C, 66.41; H, 5.67; N, 3.23. Found: C, 66.57; H, 5.43; N, 3.26.

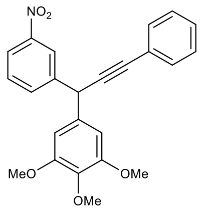

1,2,3-trimethoxy-5-(1-(4-nitrophenyl)-3-phenylprop-2-yn-1-yl)benzene (**2μ**)

IR (KBr, cm^{-1}) ν 2927, 2853, 1712, 1594, 1531, 1463, 1419, 1349, 1238, 1128, 1097, 1006, 758, 711, 691; ^{1}H NMR (600 MHz, $CDCl_3$) δ 3.84 (3H, s, OMe), 3.87 (6H, s, OMex2), 5.24 (1H, s, CH), 6.65 (2H, s, ArH), 7.33-7.35 (3H, m, ArH), 7.48-7.50 (1H, m, ArH), 7.51-7.53 (1H, m, ArH), 7.77 (1H, d, J = 7.6 Hz, ArH), 8.14 (1H, brd, J = 6.8 Hz, ArH), 8.36 (1H, brs, ArH); EIMS m/z 403 (M^+); high resolution mass calcd for $C_{24}H_{21}NO_5$: 403.1420, found m/z 403.1444.

Synthesis of 1,2,3-trimethoxy-4-(1-(4-trifluoromethylphenyl)-3-phenylprop-2-yn-1-yl)benzene (**2ν**).

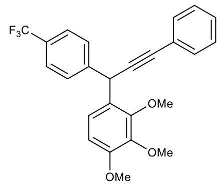

To a nitromethane (1.00 mL) solution of 1-(4-trifluoromethylphenyl)-3-phenylprop-2-yn-1-ol (**1ν**) (55.2 mg, 0.200 mmol), 1,2,3-trimethoxybenzene (67.3 mg, 0.400 mmol), tetrabutylammonium hexafluorophosphate (15.5 mg, 0.0400 mmol), 1,1'-binaphthol (11.5 mg, 4.00x10^{-5} mol) were added silver perchlorate (20.7 mg, 0.0990 mmol) and indium trichloride (8.85 mg, 4.00x10^{-5} mol) at room temperature. The reaction mixture was stirred for 30 min. The reaction mixture was poured into water (50.0 mL). The organic layer was separated and the aqueous layer was extracted with AcOEt. The combined organic layer was dried over $MgSO_4$. The solvent was removed under reduced pressure. The residue was purified by preparative TLC on silica gel eluting with AcOEt-chloroform-*n*-hexane (1:1:50) to give 1,2,3-trimethoxy-4-(1-(4-trifluoromethylphenyl)-3-phenylprop-2-yn-1-yl)benzene (**2ν**) (69.2 mg, 81%) as a yellow oil, and 1,2,3-trimethoxy-5-(1-(4-trifluoromethylphenyl)-3-phenylprop-2-yn-1-yl)benzene (**2ν**) (2.80 mg, 3%) as a yellow oil.

IR (KBr, cm^{-1}) ν 2935, 2834, 2353, 1618, 1599, 1492, 1466, 1416, 1326, 1281, 1254, 1165, 1096, 1067, 1018, 857, 799, 757, 691, 605, 537; ^{1}H NMR (600 MHz, $CDCl_3$) δ 3.81 (3H, s, OMe), 3.84 (3H, s, OMe), 3.86 (3H, s, OMe), 5.58 (1H, s, CH), 6.68 (1H, d, J = 8.9 Hz, ArH), 7.29 (1H, d, J = 8.9 Hz, ArH), 7.29-7.30 (3H, m, ArH), 7.45-7.47 (2H, m, ArH), 7.57 (4H, brs, ArH) ; ^{13}C NMR (150 MHz, $CDCl_3$) δ 37.0 (d), 55.9 (q), 60.7 (q), 60.8 (q), 84.5 (s), 89.8 (s), 107.3 (d), 123.1 (d), 123.2 (s), 124.1 (q, J = 275.4 Hz), 125.3 (d), 125.4 (d), 126.9 (s), 128.1 (d), 128.1 (dx2), 128.2 (dx2), 128.8 (q, J = 33.2 Hz), 131.6 (dx2), 142.1 (s), 146.1 (s), 150.9 (s), 153.2 (s); EIMS m/z 426 (M^+), 411 (M^+-Me), 395 (M^+-OMe); high resolution mass calcd for $C_{25}H_{21}F_3O_3$: 426.1443, found m/z 426.1434. Anal. Calcd for $C_{25}H_{21}F_3O_3$ (+1/2H_2O): C, 68.96; H, 5.09. Found: C, 68.77; H, 4.88.

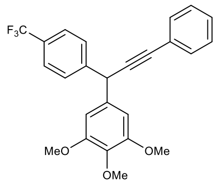

1,2,3-trimethoxy-5-(1-(4-trifluoromethylphenyl)-3-phenylprop-2-yn-1-yl)benzene (**2ν**)

IR (KBr, cm^{-1}) ν 2933, 2839, 1594, 1506, 1463, 1420, 1326, 1237, 1165, 1128, 1068, 1018, 758, 691; ^{1}H NMR (600 MHz, $CDCl_3$) δ 3.84 (3H, s, OMe), 3.85 (6H, s, OMex2), 5.19 (1H, s, CH), 6.65 (2H, s, ArH), 7.32-7.33 (3H, m, ArH), 7.46-7.48 (2H, m, ArH), 7.56 (2H, d, J = 8.6 Hz, 7.60 (2H, d, J = 8.6 Hz, ArH); EIMS m/z 426 (M^+), 411 (M^+-Me), 395 (M^+-OMe); high resolution mass calcd for $C_{25}H_{21}F_3O_3$: 426.1443, found m/z 426.1461.

Synthesis of *N*-(4-(3-phenyl-1-(2,3,4-tromethoxyphenyl)prop-2-yn-1-yl)phenyl)acetamide (**2ζ**).

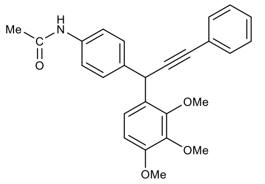

To a nitromethane (1.00 mL) solution of *N*-(4-(1-hydroxy-3-phenylprop-2-yn-1-yl)phenyl)acetamide (**1ζ**) (50.0 mg, 0.188 mmol), 1,2,3-trimethoxybenzene (47.4 mg, 0.282 mmol), and tetrabutylammonium hexafluorophosphate (14.6 mg, 0.0376 mmol), 1,1'-binaphthol (10.8 mg, 0.0377 mmol) were added silver perchlorate (19.5 mg, 0.0940 mmol) and indium trichloride (8.30 mg, 0.0377 mmol) at room temperature. The reaction mixture was stirred for 1 h. The reaction mixture was poured into water (50.0 mL). The organic layer was separated and the aqueous layer was extracted with chloroform. The combined organic layer was dried over $MgSO_4$. The solvent was removed under reduced pressure. The residue was purified by preparative TLC on silica gel eluting with AcOEt-*n*-hexane (1:1) to give methyl 4-(1-phenyl-1-(2,3,4-trimethoxyphenyl)hept-2-yn-1-yl)benzoate (**2ζ**) (92.7 mg, quant) as a yellow oil, which is filtered off as white powders (mp 141-143 ºC, from chloroform-*n*-hexane).

IR (KBr, cm^{-1}) ν 3310, 2937, 1670, 1601, 1539, 1492, 1465, 1415, 1370, 1316, 1280, 1254, 1095, 1017, 756, 692; ^{1}H NMR (600 MHz, $CDCl_3$) δ 2.13 (3H, s, CH_3), 3.80 (3H, s, OCH_3), 3.84 (3H, s, OCH_3), 3.85 (3H, s, OCH_3), 5.51 (1H, s, CH), 6.66 (1H, d, J = 8.3 Hz, ArH), 7.20 (1H, d, 9.0 Hz, ArH), 7.26-7.29 (3H, m, ArH), 7.33 (1H, brd, J = 13.8 Hz, NH), 7.38 (2H, d, J = 8.3 Hz, ArH), 7.43-7.45 (4H, m, ArH); ^{13}C NMR (150 MHz, $CDCl_3$) δ 24.5 (q), 36.5 (d), 55.9 (q), 60.7 (q), 60.9 (q), 83.8 (s), 90.7(s), 17.3 (d), 119.9 (d×2), 123.1 (d), 123.5 (s), 127.

9(d and s), 128.2 (d×2), 128.4 (d×2), 131.6 (d×2), 136.4 (s), 137.9 (s), 142.1 (s), 150.9 (s), 152.9 (s), 168.2 (s); EIMS m/z 415 (small M^+); high resolution mass calcd for $C_{26}H_{25}NO_4$: 415.1783, found m/z 415.1764. Anal. Calcd for $C_{26}H_{25}NO_4$ (+ 1 / 2 H_2O): C, 73.57; H, 6.17; N, 3.30. Found: C, 73.35; H, 6.08; N, 3.35.

Synthesis of 1,2,3-trimethoxy-4-(1-(4-nitrophenyl)hept-2-yn-1-yl)benzene (**2ο**)

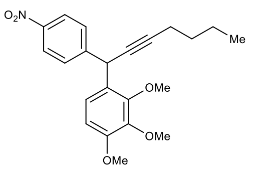

To a nitromethane (1.00 mL) solution of 1-(4-nitrophenyl)hept-2-yn-1-ol (**1ο**) (58.3 mg, 0.250 mmol), 1,2,3-trimethoxybenzene (126 mg, 0.750 mmol), and tetrabutylammonium hexafluorophosphate (19.0 mg, 0.0500 mmol) were added silver perchlorate (28.1 mg, 0.0990 mmol), 1,1'-binaphthol (14.3 mg, $5.00x10^{-5}$ mol) and indium trichloride (11.0 mg, $5.00x10^{-5}$ mol) at room temperature. The reaction mixture was stirred for 1 h. The reaction mixture was filtered through a pad of celite chloroform and then poured into water (50.0 mL). The organic layer was separated and the aqueous layer was extracted with chloroform. The combined organic layer was dried over $MgSO_4$. The solvent was removed under reduced pressure. The residue was purified by preparative TLC on silica gel eluting with AcOEt-*n*-hexane (1:10) to give 1,2,3-trimethoxy-4-(1-(4-nitrophenyl)hept-2-yn-1-yl)benzene (**2ο**) (48.7 mg, 51%) as a yellow oil.

IR (KBr, cm^{-1}) ν 2933, 2856, 2355, 1599, 1521, 1466, 1417, 1347, 1280, 1253, 1096, 1036, 1015, 857, 733; ^1H NMR (600 MHz, $CDCl_3$) δ 0.92 (3H, t, *J* = 7.5 Hz, CH_3), 1.41-1.46 (1H, m, CH), 1.52-1.55 (1H, m, CH), 2.28 (1H, dt, *J* = 2.1 and 6.9 Hz, CH_2), 3.77 (3H, s, OMe), 3.83 (3H, s, OMe), 3.85 (3H, s, OMe), 5.36 (1H, s, CH), 6.67 (1H, d, *J* = 8.3 Hz, ArH), 7.17 (1H, d, *J* = 8.3 Hz, ArH), 7.53 (2H, d, *J* = 9.0 Hz, ArH), 8.13 (2H, d, *J* = 8.9 Hz, ArH); ^{13}C NMR (150 MHz, $CDCl_3$) δ 13.5 (q), 18.5 (t), 22.0 (t), 30.9 (t), 36.6 (q), 55.9 (q), 60.6 (q), 60.7 (q), 79.5 (s), 85.5 (s), 107.2 (d), 123.0 (dx2), 126.9 (s), 128.5 (dx2), 142.0 (s), 146. 5 (s), 150.4 (s), 150.7 (s), 153.2 (s); MS m/z 383 (M^+); high resolution mass calcd for $C_{22}H_{25}NO_5$: 383.1733, found m/z 383.1750.

Synthesis of 1-(1-(2-chlorophenyl)hept-2-yn-1-yl)-2,3,4-trimethoxybenzene (**2π**)

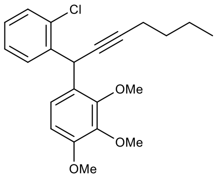

To a nitromethane (1.25 mL) solution of 1-(2-chlorophenyl)hept-2-yn-1-ol (**1π**) (50.0 mg, 0.225 mmol), 1,2,3-trimethoxybenzene (75.5 mg, 0.449 mmol), tetrabutylammonium hexafluorophosphate (17.4 mg, 0.0450 mmol), and 1,1'-binaphthol (12.9 mg, 0.0449 mmol) were added silver perchlorate (23.3 mg, 0.112 mmol) and indium trichloride (9.95 mg, 0.0449 mmol) at room temperature. The reaction mixture was stirred for 3.5 h. The reaction mixture was poured into water (50.0 mL). The organic layer was separated and the aqueous layer was extracted with AcOEt. The combined organic layer was dried over $MgSO_4$. The solvent was removed under reduced pressure. The residue was purified by preparative TLC on silica gel eluting with AcOEt-*n*-hexane (1:50) to give 1-(1-(2-chlorophenyl)hept-2-yn-1-yl)-2,3,4-trimethoxybenzene (**2π**) (66.8 mg, 80%) as a yellow oil.

IR (KBr, cm^{-1}) ν 2956, 2935, 2836, 1715, 1600, 1493, 1466, 1416, 1279, 1252, 1096, 1037, 908, 750; ^1H NMR (600 MHz, $CDCl_3$) δ 0.89 (3H, t, *J* = 6.8 Hz, Me), 1.41 (2H, fifth, *J* = 7.6 Hz, CH_2), 1.50 (2H, fifth, *J* = 7.6 Hz, CH_2), 2.23 (2H, dt, *J* = 2.1 and 6.9 Hz, CH_2), 3.68 (3H, s, OMe), 3.83 (3H, s, OMe), 3.84 (3H, s, OMe), 5.66 (1H, s, CH), 6.63 (1H, d, *J* = 8.3 Hz, ArH), 7.06 (1H, d, *J* = 8.9 Hz, ArH), 7.15 (1H, dt, *J* = 1.4 and 7.6 Hz, ArH), 7.19 (1H, dt, *J* = 1.3 and 7.8 Hz, ArH), 7.32 (1H, dd, *J* = 1.4 and 8.3 Hz, ArH), 7.42 (1H, dd, *J* = 1.4 and 7.6 Hz, ArH) ; ^{13}C NMR (150 MHz, $CDCl_3$) δ 13.6 (q), 18.6 (t), 21.9 (t), 31.0 (t), 34.0 (d), 55.8 (q), 60.4 (q), 60.6 (q), 79.7 (s), 84.5 (s), 106.6 (d), 122.8 (d), 126.6 (d), 127.2 (s), 127.8 (d), 129.4 (d), 129.8 (d), 133.3 (s), 140.0 (s), 142.1 (s), 151.2 (s), 152.8 (s); EIMS m/z 372 (M^+), 357 (M^+-Me); high resolution mass calcd for $C_{22}H_{25}ClO_3$: 372.1492, found m/z 372.1498. Anal. Calcd for $C_{22}H_{25}ClO_3$ (+1/5H_2O): C, 70.19; H, 6.80. Found: C, 70.17; H, 6.78.

Synthesis of methyl 4-(1-phenyl-1-(2,3,4-trimethoxyphenyl)hept-2-yn-1-yl)benzoate (**2ρ**).

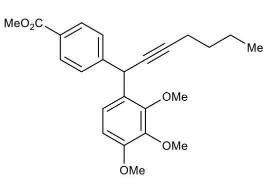

To a nitromethane (1.00 mL) solution of methyl 4(1-hydroxyhept-2-yn-1-yl)benzoate (**1ρ**)(50.0 mg, 0.203 mmol), 1,2,3-trimethoxybenzene (68.3 mg, 0.406 mmol), tetrabutylammonium hexafluorophosphate (15.7 mg, 0.0406 mmol), and 1,1'-binaphthol (11.6 mg, 0.0406 mmol) were added silver perchlorate (21.0 mg, 0.102 mmol) and indium trichloride (9.00 mg, 0.0406 mmol) at room temperature. The reaction mixture was stirred for 30 min. The reaction mixture was poured into water (50.0 mL). The organic layer was separated and the aqueous layer was extracted with chloroform. The combined organic layer was dried over $MgSO_4$. The solvent was removed under reduced pressure. The residue was purified by preparative TLC on silica gel eluting with AcOEt-*n*-hexane (1:20) to give methyl 4-(1-(2,3,4-trimethoxyphenyl)hept-2-yn-1-yl)benzoate (**2ρ**) (63.9 mg, 79%) as a yellow oil and 4-(1-(3,4,5-trimethoxyphenyl)hept-2-yn-1-yl)benzoate (**2ρ**) (2.80 mg, 4%) as a yellow oil.

IR (KBr, cm^{-1}) ν 3447, 2955, 2936, 1722, 1608, 1493, 1466, 1416, 1280, 1096, 1019, 797, 743, 705; ^1H NMR (600 MHz, $CDCl_3$) δ 0.91 (3H, t, *J* = 7.6 Hz, CH_3), 1.41-1.44 (2H, m, CH_2), 1.50-1.54 (2H, m, CH_2), 2.27 (2H, dt, *J* = 2.0 and 6.9 Hz, CH_2), 3.72 (3H, s, OCH_3), 3.83 (3H, s, OCH_3), 3.84 (3H, s, OCH_3), 3.88 (3H, s, OCH_3), 5.33 (1H, s, CH), 6.66 (1H, d, *J* = 8.9 Hz, ArH), 7.17 (1H, d, *J* = 9.0 Hz, ArH), 7.44 (2H, d, *J* = 8.4 Hz, ArH), 7.95 (2H, d, *J* = 8.2 Hz, ArH); ^{13}C NMR (150 MHz, $CDCl_3$) δ 13.6 (q), 18.6 (t), 22.0 (t), 31.0 (t), 36.6 (d), 52.0 (q), 55.9 (q), 60.6 (q), 60.7 (q), 80.2 (s), 84.8 (s), 107.2 (d), 123.1 (d), 127.8 (d×2 and s), 128.2 (s), 129.6 (d×2) 142.0 (s), 148.1 (s), 150.8 (s),

152.9 (s), 167.0 (s); MS m/z 396 (M^+), 381 (M^+-Me), 354 (M^+-Pr), 229 (M^+-$C_6H_2(OMe)_3$); high resolution mass calcd for $C_{26}H_{24}O_5$: 396.1936, found m/z 396.1936. Anal. Calcd for $C_{26}H_{24}O_5$: C, 72.71; H, 7.21. Found: C, 72.39; H, 7.19.

Synthesis of 1-methyl-4-(1-phenylpent-1-yn-3-yl)benzene (**6a**) and 1-methyl-2-(1-phenylpent-1-yn-3-yl)benzene (**6a**).

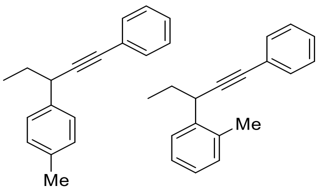

To a nitromethane (1.50 mL) solution of 1-phenylpent-1-yn-3-ol (**6a**)(50.0 mg, 0.312 mmol), toluene (287 mg, 3.12 mmol), tetrabutylammonium hexafluorophosphate (24.2 mg, 0.0624 mmol), and 1,1'-bi-2-naphthol (17.9 mg, 0.0624 mmol) were added silver perchlorate (25.9 mg, 0.125 mmol) and indium trichloride (13.8 mg, 0.0624 mmol) at room temperature. The reaction mixture was stirred for 15 min and then poured into water (50 mL). The organic layer was separated and the aqueous layer was extracted with AcOEt. The combined organic layer was dried over $MgSO_4$. The solvent was removed under reduced pressure. The residue was purified by preparative TLC on silica gel eluting with AcOEt-*n*-hexane (1:40) to give 1-methyl-4-(1phenylpent-1-yn-3-yl)benzene and 1-methyl-2-(1-phenylpent-1-yn-3-yl)benzene (**6a**)(40.0 mg, 55%) (5.3 : 1) as a colorless oil.

IR (KBr, cm^{-1}) ν 3443, 2967, 2927, 2872, 1715, 1687, 1599, 1490, 1451, 1224, 1024, 814, 757, 691; ^{1}H NMR (600 MHz, $CDCl_3$) δ 1.05 (t, *J* = 7.6 Hz, 4-CH_3), 1.11 (t, *J* = 7.6 Hz, 2-CH_3), 1.81-1.87 (m, 4- and 2-CH_2), 2.33 (s, 4-CH_3), 2.38 (s, 2-CH_3), 3.74 (t, *J* = 7.5 Hz, 4-CH), 3.96 (dd, *J* = 5.5 and 8.2 Hz, 2-CH), 7.14 (d, *J* = 8.3 Hz, ArH), 7.27 (d, *J* = 7.6 Hz, 4-ArH), 7.30 (d, *J* = 8.3 Hz, 4-ArH), 7.42-7.45 (m, 4- and 2-ArH); ^{13}C NMR (150 MHz, $CDCl_3$) δ 11.6 (4-q), 12.2 (2-q), 19.2 (2-q), 21.0 (4-q), 30.1 (2-t), 31.7 (4-t), 36.5 (2-d), 39.5 (4-d), 82.7 (2-s), 83.2 (4-s), 91.7 (4-s), 91.9 (2-s), 123.9 (4-s), 126.2 (2-d), 126.6 (2- d), 127.4 (4-d×2), 127.6 (4-d), 128.2 (4-d×2), 129.1 (4-d×2), 130.4 (2-d), 131.6 (4-d×2), 134.9 (2-s), 136.2 (4-s), 139.0 (4-s), 140.2 (2-s); EIMS m/z 233 (M^+); high resolution mass calcd for $C_{18}H_{18}$: 234.1409, found m/z 234.1420.

Synthesis of 1-methoxy-4-(1-phenylpent-1-yn-3-yl)benzene (**6b**).

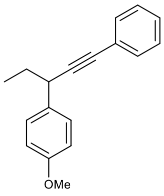

To a nitromethane (1.50 mL) solution of 1-phenylpent-1-yn-3-ol (**1a**) (50.0 mg, 0.312 mmol), anisole (337 mg, 3.12 mmol), tetrabutylammonium hexafluorophosphate (24.2 mg, 0.0624 mmol), and 1,1'-bi-2-naphthol (18.0 mg, 0.0624 mmol) were added silver perchlorate (35.0 mg, 0.125 mmol) and indium trichloride (13.8 mg, 0.0624 mmol) at room temperature. The reaction mixture was stirred for 10 min and then poured into water (50 mL). The organic layer was separated and the aqueous layer was extracted with AcOEt. The combined organic layer was dried over $MgSO_4$. The solvent was removed under reduced pressure. The residue was purified by preparative TLC on silica gel eluting with chloroform-*n*-hexane (1:10) to give 1-methoxy-4-(1-phenylpent-1-yn-3-yl)benzene (**6b**) (60.3 mg, 77%) as a yellow oil, 1-methoxy-2-(1-phenylpent-1-yn-3-yl)benzene (**6b**) (13.2 mg, 17%) as a yellow oil.

IR (KBr, cm^{-1}) ν 2966, 2932, 2873, 2836, 1714, 1681, 1609, 1601, 1510, 1490, 1462, 1442, 1301, 1250, 1176, 1036, 832, 757, 692, 552; ^{1}H NMR (600 MHz, $CDCl_3$) δ 1.04 (3H, t, *J* = 7.3 Hz, CH_3), 1.83 (2H, sex, *J* = 7.3 Hz, CH_2), 3.73 (1H, t, *J* = 6.9 Hz, CH), 3.79 (3H, s, OCH_3), 6.87 (2H, d, *J* = 8.7 Hz, ArH), 7.27-7.30 (3H, m, ArH), 7.32 (2H, d, *J* = 8.7 Hz, ArH), 7.43-7.45 (2H, m, ArH); ^{13}C NMR (150 MHz, $CDCl_3$) δ 11.8(q), 31.7 (t), 39.1 (d), 55.3 (q), 83.2 (s), 91.8 (s), 113.8 (d×2), 123.9 (s), 127.6 (d), 128.2 (d×2), 128.5 (d×2), 131.6 (d×2), 134.1 (s), 158.3 (s); EIMS m/z no found; high resolution mass calcd for $C_{18}H_{18}O$: 250.1357, found m/z 250.1312.

Anal. Calcd for $C_{18}H_{18}O$: C, 86.36; H, 7.25. Found: C, 86.35; H, 7.49.

IR (KBr, cm^{-1}) ν 2965, 2836, 1599, 1492, 1463, 1440, 1287, 1243, 1162, 1116, 1028, 754, 691; ^{1}H NMR (600 MHz, $CDCl_3$) δ 1.06 (3H, t, *J* = 6.9 Hz, CH_3), 1.71-1.76 (1H, m, CH_2), 1.83-1.88 (1H, m, CH_2), 3.84 (3H, s, OCH_3), 4.27 (1H, dd, *J* = 5.5 and 8.3 Hz, CH), 6.87 (1H, d, *J* = 8.3 Hz, ArH), 6.97 (1H, t, *J* = 7.6 Hz, ArH), 7.22 (1H, brt, *J* = 7.6 Hz, ArH), 7.27-7.30 (3H, m, ArH), 7.45 (1H, dd, *J* = 8.3 and 2.1 Hz, ArH), 7.60 (1H, dd, *J* = 5.6 and 2.0 Hz, ArH); ^{13}C NMR (150 MHz, $CDCl_3$) δ 11.9(q), 29.7 (t), 33.1 (d), 55.4 (q), 82.6 (s), 92.0 (s), 110.4 (d), 120.6 (d), 124.1 (s), 127.5 (d), 127.7 (d), 128.1 (d×2), 128.7 (d), 130.4 (s), 131.7 (d×2), 156.2(s); EIMS m/z 250 (M^+), 235 (M^+-Me), 221 (M^+-Et); high resolution mass calcd for $C_{18}H_{18}O$: 250.1357, found m/z 250.1374. Anal. Calcd for $C_{18}H_{18}O$(+ 1 / 2 H_2O): C, 83.36; H, 7.38. Found: C, 83.71; H, 7.35.

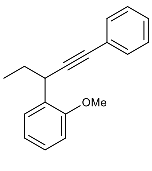

Synthesis of 1,4-dimethyl-2-(1-phenylpent-1-yn-3-yl)benzene (**6c**) .

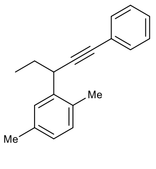

To a nitromethane (1.00 mL) solution of 1-phenylpent-1-yn-3-ol (**1a**) (50.0 mg, 0.312 mmol), *p*-xylene (331 mg, 3.12 mmol), tetrabutylammonium hexafluorophosphate (24.2 mg, 0.0624 mmol), and 1,1'-binaphthol (18.0 mg, 0.0624 mmol) were added silver perchlorate (32.4 mg, 0.156 mmol) and indium trichloride (13.8 mg, 0.0624 mmol) at room temperature. The reaction mixture was stirred for 12 h. The reaction mixture was poured into water (50.0 mL). The organic layer was separated and the aqueous layer was extracted with AcOEt. The combined organic layer was dried over $MgSO_4$. The solvent was removed under reduced pressure. The residue was purified by preparative TLC on silica gel eluting with *n*-hexane to give 1,4-dimethyl-2-(1-phenylpent-1-yn-3-yl)benzene (**6c**) (39.9 mg, 52%) as a yellow oil.

IR (KBr, cm^{-1}) ν 2967, 2928, 2871, 1599, 1502, 1491, 1460, 1442, 1378, 1335, 809, 756, 691; ^{1}H NMR (600 MHz, $CDCl_3$) δ 1.11 (3H, t, *J* =

7.6 Hz, CH_3), 1.78-1.82 (2H, m, CH_2), 2.32 (6H, d, *J* = 5.5 Hz, CH_3), 3.92 (1H, dd *J* = 8.3 Hz and 2.1 Hz, CH), 6.96 (1H, d, *J* = 8.2 Hz, ArH), 7.04 (1H, d, *J* = 7.6 Hz, ArH), 7.27-7.30 (3H, m, ArH), 7.36 (1H, s, ArH), 7.43-7.45 (2H, m, ArH); ^{13}C NMR (150 MHz, $CDCl_3$) δ 12.3 (q), 18.8 (q), 21.1 (q), 30.2 (t), 36.5 (d), 82.6 (s), 92.0 (s), 124.0 (d), 127.3 (d), 127.6 (d), 128.1 (d×2), 128.2 (d), 130.3 (d), 131.6 (d×2), 131.7 (s), 135.6 (s), 140.0 (s); EIMS m/z 246 (M^+); high resolution mass calcd for $C_{19}H_{20}$: 248.1565, found m/z 248.1597. Anal. Calcd for $C_{19}H_{20}$ (+ 1 / 2 H_2O): C, 88.67; H, 8.22. Found: C, 88.67; H, 8.16.

Synthesis of 1,4-dimethoxy-2-(1-phenylpent-1-yn-3-yl)benzene (**6d**).

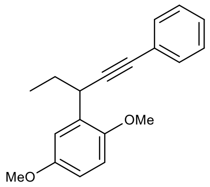

To a nitromethane (1.50 mL) solution of 1-phenylpent-1-yn-3-ol (**1a**) (50.0 mg, 0.312 mmol), 1,5-dimethoxybenzene (129 mg, 0.936 mmol), and tetrabutylammonium hexafluorophosphate (24.2 mg, 0.0624 mmol) were added silver perchlorate (35.0 mg, 0.128 mmol) and indium trichloride (13.8 mg, 0.0624 mmol) at room temperature. The reaction mixture was stirred for 2 h and then poured into water (50.0 mL). The organic layer was separated and the aqueous layer was extracted with chloroform. The combined organic layer was dried over $MgSO_4$. The solvent was removed under reduced pressure. The residue was purified by preparative TLC on silica gel eluting with AcOEt-*n*-hexane (1:40) to give 1,4-dimethoxy-2-(1-phenylpent-1-yn-3-yl)benzene (**6d**) (71.4 mg, 82%) as a yellow oil.

IR (KBr, cm^{-1}) ν 3449, 2965, 2834, 1714, 1500, 1464, 1443, 1278, 1239, 1214, 1178, 1051, 806, 758, 692; ^{1}H NMR (600 MHz, $CDCl_3$) δ 1.07(3H, t, *J* = 6.2 Hz, CH_3), 1.72-1.76 (1H, m, CH_2), 1.83-1.86 (1H, m, CH_2), 3.78 (3H, s, OCH_3), 3.80 (3H, s, OCH_3), 4.23 (1H, dd, *J* = 6.2 and 7.5 Hz, CH), 6.74 (1H, dd, *J* = 3.5 Hz, CH), 6.79 (1H, d, *J* = 8.9 Hz, ArH), 7.21 (1H, brs, ArH), 7.27-7.28 (3H, m, ArH), 7.44 (2H, brd, *J* = 8.3 Hz, ArH); ^{13}C NMR (150 MHz, $CDCl_3$) δ 11.8 (q), 29.8 (t), 33.3 (d), 55.7 (q), 56.0 (q), 82.7 (s), 91.8 (s), 111.4 (d), 111.7 (d), 115.1 (d), 124.0 (s), 127.6 (d), 128.1 (d×2), 131.6 (d×2 and s), 150.5 (s), 153.6 (s); MS m/z 280(M^+), 265 (M^+-Me), 251 (M^+-Et); high resolution mass calcd for $C_{19}H_{20}O_2$: 280.1463, found m/z 280.1426. Anal. Calcd for $C_{19}H_{20}O_2$ (+1/13H_2O): C,81.00; H, 7.21. Found: C, 80.73; H, 7.33.

Synthesis of 4-(1-phenylpent-1-yn-3-yl)benzo[d][1,3]dioxole (**6e**)

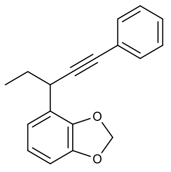

To a nitromethane (1.50 mL) solution of 1-phenylpent-1-yn-3-ol (**1a**) (50.0 mg, 0.312 mmol), benzo[d][1,3]dioxole (114 mg, 0.936 mmol), tetrabutylammonium hexafluorophosphate (24.2 mg, 0.0624 mmol), and 1,1'-binaphthol (17.9 mg, 0.0624 mmol) were added silver perchlorate (25.9 mg, 0.125 mmol) and indium trichloride (13.8 mg, 0.0624 mmol) at room temperature. The reaction mixture was stirred for 15 min and then poured into water (50.0 mL). The organic layer was separated and the aqueous layer was extracted with chloroform. The combined organic layer was dried over $MgSO_4$. The solvent was removed under reduced pressure. The residue was purified by preparative TLC on silica gel eluting with AcOEt-*n*-hexane (1:40) to give 4-(1-phenylpent-1-yn-3-yl)benzo[d][1,3]dioxole (**6e**) (53.1 mg, 64%) as a brown oil.

IR (KBr, cm^{-1}) ν 2965, 2929, 2874, 2232, 1503, 1488, 1441, 1247, 1227, 1182, 1105, 1040, 932, 810, 757, 691; ^{1}H NMR (600 MHz, $CDCl_3$) δ 1.04 (3H, t, *J* = 7.2 Hz, CH_3), 1.81-1.84 (2H, m, CH_2), 3.70 (1H, dd, *J* = 6.6 and 10.0 Hz, CH), 5.93 (2H, s, CH_2), 6.77 (1H, dd, *J* = 2.9 and 7.9 Hz, ArH), 6.84-6.86 (1H, m, ArH), 6.93-6.94 (1H, m, ArH), 7.28-7.30 (3H, m, ArH), 7.43-7.46 (2H, m, ArH); ^{13}C NMR (150 MHz, $CDCl_3$) δ 11.8 (q), 31.8 (t), 39.6 (d), 83.3 (s), 91.5 (s), 100.9 (t), 108.0 (d), 120.5 (d), 123.7 (s), 127.7 (d), 128.2 (dx2), 131.6 (dx3), 135.9 (s), 146.2 (s), 147.6 (s); MS m/z 264 (M^+), 235 (M^+-Et); high resolution mass calcd for $C_{18}H_{16}O_2$ 264.1150: , found m/z 264.1150. Anal. Calcd for $C_{18}H_{16}O_2$ (+3/7H_2O): C, 79.47; H, 6.25. Found: C, 79.37; H, 6.06.

Synthesis of 1,3,5-trimethyl-2-(1-phenylpent-1-yn-3-yl)benzene (**6f**)

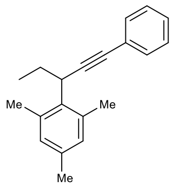

To a nitromethane (1.50 mL) solution of 1-phenylpent-1-yn-3-ol (**1a**) (50.0 mg, 0.312 mmol), mesitylene (113 mg, 0.936 mmol), tetrabutylammonium hexafluorophosphate (24.2 mg, 0.0624 mmol), and (R)-1,1'-binaphthol (17.9 mg, 0.0624 mmol) were added silver perchlorate (25.9 mg, 0.125 mmol) and indium trichloride (13.8 mg, 0.0624 mmol) at room temperature. The reaction mixture was stirred for 0.5 h. The reaction mixture was poured into water (50.0 mL). The organic layer was separated and the aqueous layer was extracted with AcOEt. The combined organic layer was dried over $MgSO_4$. The solvent was removed under reduced pressure. The residue was purified by preparative TLC on silica gel eluting with *n*-hexane to give 1,3,5-trimethyl-2-(1-phenylpent-1-yn-3-yl)benzene (**6f**) (59.0 mg, 71%) as a yellow oil.

IR (KBr, cm^{-1}) 2966, 2928, 2871, 2232, 1715, 1599, 1489, 1457, 1443, 1219, 851, 756, 691; ^{1}H NMR (400 MHz, $CDCl_3$) δ 1.09 (3H, d, *J* = 7.3 Hz, CH_3), 1.71-1.80 (1H, m, CH), 1.96-2.08 (1H, m, CH), 2.25 (3H, s, Me), 2.45 (6H, brs, Mex2), 4.15 (1H, dd, *J* = 6.4 and 9.2 Hz, CH), 6.85 (2H, s, ArH), 7.24-7.29 (3H, m, ArH), 7.38-7.40 (2H, m, ArH); ^{13}C NMR (150 MHz, $CDCl_3$) δ 12.8 (q), 20.7 (q), 20.9 (qx2), 27.7 (t), 34.4 (d), 82.4 (s), 91.8 (s), 124.1 (s), 127.4 (d), 128.1 (dx2), 130.0 (s), 131.4 (dx2), 134.9 (s), 135.8 (s), 136.2 (s); EIMS m/z 262 (M^+), 233 (M^+ -Et); high resolution mass calcd for $C_{20}H_{22}$: 262.1722, found m/z 262.1704. Anal. Calcd for $C_{20}H_{22}$(+1/8H_2O): C, 90.77; H, 8.47. Found: C, 90.71; H, 8.58.

Synthesis of 3-(4,4-dimethyl-1-phenylpent-1-yn-3-yl)-2,5-dimethylthiophene (**6g**).

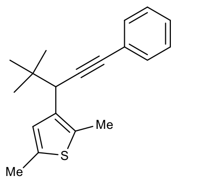

To a nitromethane (1.50 mL) solution of 4,4-dimethyl-1-phenylpent-1-yn-3-ol (**1c**) (58.7 mg, 0.312 mmol), 2,5-dimethylthiophene (175 mg, 1.56 mmol), tetrabutylammonium hexafluorophosphate (24.2 mg, 0.0624 mmol), and 1,1'-binaphthol (17.9 mg, 0.0624 mmol) were added silver perchlorate (35.0 mg, 0.125 mmol) and indium trichloride (13.8 mg, 0.0624 mmol) at 50ºC. The reaction mixture was stirred for 3 h and then poured into water (50 mL). The organic layer was separated and the aqueous layer was extracted with AcOEt. The combined organic layer was dried over $MgSO_4$. The solvent was removed under reduced pressure. The residue was purified by preparative TLC on silica gel eluting with *n*-hexane to give 3-(4,4-dimethyl-1-phenylpent-1-yn-3-yl)-2,5-dimethylthiophene (**6g**) (39.0 mg, 44%) as white powders (mp 73-75 ºC) and 3-(4,4-dimethyl-1-phenylpenta-1,2-dien-1-yl)-2,5-dimethylthiophene (**7g**) (14.6 mg, 17%) a pale yellow oil.

IR (KBr, cm^{-1}) ν 2965, 2866, 1489, 1442, 1391, 1363, 1214, 1142, 836, 691; ^{1}H NMR (600 MHz, $CDCl_3$) δ 1.05 (9H, s, *tert*-Bu), 2.33 (3H, s, Me), 2.39 (3H, s, Me), 3.62 (1H, s, CH), 6.70 (1H, s, ArH), 7.23-7.29 (3H, m, ArH), 7.41-7.43 (2H, m, ArH); ^{13}C NMR (150 MHz, $CDCl_3$) δ 13.7 (q), 15.2 (q), 27.7 (qx3), 36.8 (s), 42.8 (d), 82.9 (s), 91.4 (s), 124.1 (s), 127.5 (d), 127.6 (d), 128.1 (d×2), 131.5 (d×2), 131.9 (s), 134.2 (s), 134.7 (s); EIMS m/z 282 (M^+), 225 (M^+-tBu); high resolution mass calcd for $C_{19}H_{22}S$: 282.1442, found m/z 282.1424.Anal. Calcd for $C_{14}H_{16}S(+1/5H_2O)$: C, 79.78; H, 7.89. Found: C, 79.64; H, 7.91.

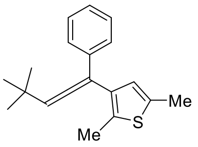

IR (KBr, cm^{-1}) ν 2961, 2918, 2865, 1715, 1457, 11363, 1220, 1140, 1070, 824, 765; ^{1}H NMR (600 MHz, $CDCl_3$) δ 1.01 (9H, s, Mex3), 2.40 (3H, s, Me), 2.46 (3H, s, Me), 3.37 (1H, s, CH), 6.40 (1H, d, *J* = 2.1 Hz, allenic H), 6.74 (1H, s, ArH), 7.17-7.19 (1H, m, ArH), 7.24-7.27 (3H, m, ArH), 7.56 (1H, d, *J* = 7.6 Hz, ArH); MS m/z 282 (M^+), 225 (M^+-tBu); high resolution mass calcd for $C_{19}H_{22}S$: 282.1442, found m/z 282.1431.

Synthesis of 4-(4,4-dimethyl-1-phenylpent-1-yn-3-yl)-2-methylthiophene (**6h**).

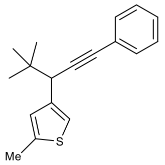

To a nitromethane (1.00 mL) solution of 4,4-dimethyl-1-phenylpent-1-yn-3-ol (**1c**) (58.7 mg, 0.312 mmol), 2-methylthiophene (153 mg, 1.56 mmol), and tetrabutylammonium hexafluorophosphate (24.2 mg, 0.0624 mmol), 1,1'-binaphthol (18.0 mg, 0.0624 mmol) were added silver perchlorate (32.4 mg, 0.156 mmol) and indium trichloride (13.8 mg, 0.0624 mmol). The reaction mixture was stirred at room temperature for 10 min. The reaction mixture was filtered through a pad of celite with chloroform and then poured into a saturated sodium hydrogencarbonate. The organic layer was separated and the aqueous layer was extracted with AcOEt. The combined organic layer was dried over $MgSO_4$ and the solvent was removed under reduced pressure. The residue was purified by preparative TLC on silica gel eluting with *n*-hexane to give 4-(4,4-dimethyl-1-phenylpent-1-yn-3-yl)-2-methylthiophene (**6h**) (69.9 mg, 83%) as a yellow oil.

IR (KBr, cm^{-1}) ν 2966, 2866, 1715, 1599, 1490, 1442, 1393, 1364, 1219, 800, 756, 691^{1}H NMR (600 MHz, $CDCl_3$) δ 1.05 (9H, s, Mex3), 2.43 (3H, s, Me), 3.79 (1H, brs, CH), 6.58 (1H, brs, ArH), 6.72 (1H, brs, ArH), 7.23-7.28 (3H, m, ArH), 7.42-7.43 (2H, m, ArH); ^{13}C NMR (150 MHz, $CDCl_3$) δ 15.3 (q), 27.7 (qx3), 35.4 (s), 45.8 (d), 83.4 (s), 90.7 (s), 123.7 (s), 124.2 (d), 126.3 (d), 127.7 (d), 128.1 (dx2), 131.5 (dx2), 138.3 (s), 139.9 (s); MS m/z 268 (M^+), 253 (M^+-Me), 238 (M^+-Mex2), high resolution mass calcd for $C_{18}H_{20}S$: 268.1286, found m/z 268.1274. Anal. Calcd for $C_{18}H_{20}S$ (+1/2H_2O): C, 77.93; H, 7.63. Found: C, 77.65; H, 7.39.

Synthesis of 3-(1-cyclohexyl-3-phenylprop-2-yn-1-yl)benzofuran (**6i**).

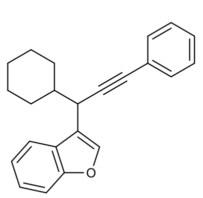

To a nitromethane (1.00 mL) solution of 1-cyclohexyl-3-phenylprop-2-yn-1-ol (**1d**) (75.0 mg, 0.350 mmol), 2,3-benzofuran (82.7 mg, 0.709 mmol), and tetrabutylammonium hexafluorophosphate (27.1 mg, 0.0700 mmol) were added silver perchlorate (36.3 mg, 0.175 mmol) and indium trichloride (15.5 mg, 0.0700 mmol) at room temperature. The reaction mixture was stirred for 30 min. The reaction mixture was filtered through a pad of celite with chloroform and then poured into water (50.0 mL). The organic layer was separated and the aqueous layer was extracted with chloroform. The combined organic layer was dried over $MgSO_4$. The solvent was removed under reduced pressure. The residue was purified by preparative TLC on silica gel eluting with chloroform-*n*-hexane (1:5) to give 3-(1-cyclohexyl-3-phenylprop-2-yn-1-yl)benzofuran (**6i**) (56.2 mg, 51%) as a yellow oil.

IR (KBr, cm^{-1}) ν 2927, 2852, 1714, 1678, 1491, 1454, 1254, 1254, 806, 755, 691, 529; ^{1}H NMR (600 MHz, $CDCl_3$) δ 1.15-1.20 (1H, m, CH_2), 1.23-1.37 (4H, m, CH_2), 1.67 (1H, brd, *J* = 13.0 Hz, CH), 1.73-1.81 (4H, m, CH_2), 2.02-2.03 (1H, m, CH), 3.97 (1H, d, *J* = 6.2 Hz, CH), 6.70 (1H, s, ArH), 7.18 (1H, dt, *J* = 1.3 nad 7.6 Hz, ArH), 7.23 (1H, dd, *J* = 1.4 and 8.3 Hz, ArH), 7.30-7.33 (3H, m, ArH), 7.45 (1H, d, *J* = 8.2 Hz, ArH), 7.47-7.49 (2H, m, ArH), 7.51 (1H, d, *J* = 7.6 Hz, ArH); ^{13}C NMR (150 MHz, $CDCl_3$) δ 26.1 (t), 26.2 (t), 26.3 (t), 29.2 (t), 31.4 (t), 39.2 (d), 41.6 (d), 83.6 (s), 87.1 (s), 103.9 (d), 111.0 (d), 120.6 (d), 122.6 (d), 123.4 (s), 123.5 (d), 128.0 (d), 128.2 (d×2), 128.5 (s), 131.7 (d×2), 154.9 (s), 157.0 (s); MS m/z 314 (M^+), 231 (M^+-C_6H_{11}); high resolution mass calcd for $C_{23}H_{22}O$: 314.1671, found m/z 314.1664. Anal. Calcd for $C_{23}H_{22}O$: C, 87.86; H, 7.05. Found: C, 87.62; H, 7.19.

Synthesis of 2- and 3-(1-cyclohenyl-3-phenylprop-2-yn-1-yl)benzo[b]thiophene (**6j**)

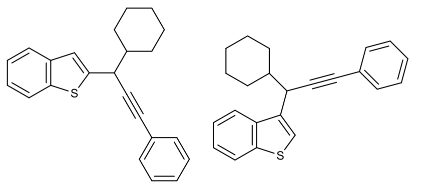

To a nitromethane (1.50 mL) solution of 1-cyclohexyl-3-phenylprop-2-yn-1-ol (**1d**) (50.0 mg, 0.233 mmol), benzothiophene (93.8 mg, 0.699 mmol), tetrabutylammonium hexafluorophosphate (18.1 mg, 0.0466 mmol), and 1,1'-bi-2-naphthol (13.3 mg, 0.0466 mmol) were added silver perchlorate (26.2 mg, 0.0932 mmol) and indium trichloride (10.3 mg, 0.0466 mmol) at room temperature. The reaction mixture was stirred for 10 min and then poured into water (50 mL). The organic layer was separated and the aqueous layer was extracted with AcOEt. The combined organic layer was dried over $MgSO_4$. The solvent was removed under reduced pressure. The residue was purified by preparative TLC on silica gel eluting with *n*-hexane to give 2- and 3-(1-cyclohenyl-3-phenylprop-2-yn-1-yl)benzo[b]thiophene (**6j**) (57.8 mg, 75%) as a yellow oil.

IR (KBr, cm^{-1}) ν 2927, 2852, 1490, 1444, 1361, 1219, 1086, 758, 691; ^{1}H NMR (600 MHz, $CDCl_3$) δ 1.15-1.35 (m, CH_2), 1.64 (brd, *J* = 7.5 Hz, CH_2), 1.78-1.90 (m, CH_2), 1.73 (brd, *J* = 9.5 Hz, CH_2), 1.78-1.90 (m, CH_2), 4.05 (d, *J* = 5.5 Hz, CH), 4.12 (d, *J* = 6.2 Hz, CH), 7.25-7.35 (m, ArH), 7.38 (t, *J* = 7.6 Hz, ArH), 7.41 (s, ArH), 7.45 (d, *J* = 6.9 Hz, ArH), 7.48 (d, *J* = 6.2 Hz, ArH), 7.69 (d, *J* = 7.6 Hz, ArH), 7.77 (d, *J* = 7.6 Hz, ArH), 7.86 (d, *J* = 8.3 Hz, ArH), 7.88 (d, *J* = 8.3 Hz, ArH); ^{13}C NMR (150 MHz, $CDCl_3$) δ 26.1 (t), 26.2 (t), 26.3 (t), 26.4 (t), 29.3 (t), 29.5 (t), 31.6 (t), 32.1 (t), 39.3 (d), 41.0 (d), 42.1 (d), 44.7 (d), 121.7 (d), 122.0 (d), 122.2 (d), 123.0 (d), 123.1 (d), 123.5 (d), 123.8 (d), 123.9 (d), 124.1 (d), 127.8 (d), 127.9 (d), 128.2 (d), 131.6 (d), 131.7 (d), 135.1 (s), 137.7 (s), 139.5 (s), 139.6 (s), 140.8 (s), 145.4 (s); EIMS m/z; high resolution mass calcd for $C_{23}H_{22}S$: 330.1442, found m/z 330.1414.

Synthesis of 4-(4,4-dimethylphenylpent-1-yn-3-yl)phenol (**6k**).

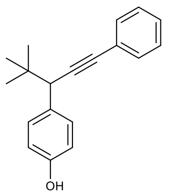

To a nitromethane (1.50 mL) solution of 4,4-dimethyl-1-phenylpent-1-yn-3-ol (**1c**) (58.7 mg, 0.312 mmol), phenol (58.7 mg, 0.624 mmol), 1,1'-binaphthol (17.9 mg, 0.0624 mmol) and tetrabutylammonium hexafluorophosphate (24.2 mg, 0.0624 mmol) were added silver perchlorate (32.4 mg, 0.156 mmol) and indium trichloride (13.8 mg, 0.0624 mmol) at room temperature. The reaction mixture was stirred for 12 h. The reaction mixture was poured into a water (50.0 mL). The organic layer was separated and the aqueous layer was extracted with AcOEt. The combined organic layer was dried over $MgSO_4$. The solvent was removed under reduced pressure. The residue was purified by preparative TLC on silica gel eluting with AcOEt-$CHCl_3$-*n*-hexane (1:1:20) to give 4-(4,4-dimethylphenylpent-1-yn-3-yl)phenol (**6k**) (63.9 mg, 77%) as white powders (mp 104-105 °C, from $CHCl_3$-*n*-hexane) and 2-(4,4-dimethylphenylpent-1-yn-3-yl)phenol (**6k**) (8.4 mg, 10%) as a yellow oil.

IR (KBr, cm^{-1}) ν 3384, 2964, 2929, 2223, 1702, 1614, 1514, 1490, 1393, 1364, 1254, 1216, 1174, 830, 757, 690; ^{1}H NMR (600 MHz, $CDCl_3$) δ 1.01 (9H, s, Mex3), 3.56 (1H, s, CH), 5.14 (1H, brs, OH), 6.77 (2H, d, *J* = 8.9 Hz, ArH), 7.23 (2H, d, *J* = 8.9 Hz, ArH), 7.26-7.29 (3H, m, ArH), 7.42-7.44 (2H, m, ArH); ^{13}C NMR (150 MHz, $CDCl_3$) δ 27.6 (qx3), 35.4 (s), 49.4 (d), 83.6 (s), 91.6 (s), 114.5 (dx2), 124.0 (s), 127.6 (d), 128.2 (dx2), 130.8 (dx2), 131.5 (dx2), 131.6 (s), 154.3 (s); EIMS m/z 264 (M^+), 207 (M^+-t-Bu). Anal. Calcd for $C_{19}H_{20}O$ (+3/10 H_2O): C, 84.59; H, 7.70. Found: C, 84.44; H, 7.53.

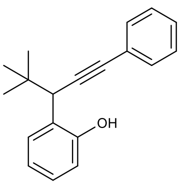

IR (KBr, cm^{-1}) ν 3428, 2965, 2929, 2867, 2359, 1705, 1598, 1455, 1364, 1093, 757, 691; ^{1}H NMR (600 MHz, $CDCl_3$) δ 1.08 (9H, s, Mex3), 3.92 (1H, s, CH), 5.96 (1H, brs, OH), 6.83 (1H, d, *J* = 9.0 Hz, ArH), 6.89 (1H, dt, *J* = 1.4 and 7.6 Hz, ArH), 7.15 (1H, dt, *J* = 1.4 and 7.6 Hz, ArH), 7.24 (1H, brd, *J* = 7.6 Hz, ArH), 7.29-7.31 (3H, m, ArH), 7.44-7.46 (2H, m, ArH); EIMS m/z 264 (M+), high resolution mass calcd for $C_{19}H_{20}O$: 264.1514, found m/z 264.1510.

Synthesis of 4-(*tert*-butyl)-2-(4,4-dimethyl-1-phenylpent-1-yn-3-yl)phenol (**6l**).

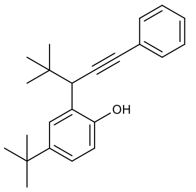

To a nitromethane (2.00 mL) solution of 4,4-dimethyl-1-phenylpent-1-yn-3-ol (**1c**) (50.0 mg, 0.266 mmol), 4-*tert*-butylphenol (120 mg, 0.797 mmol), 1,1-binaphthol (15.2 mg, 0.0531 mmol), and tetrabutylammonium hexafluorophosphate (20.6 mg, 0.0531 mmol) were added silver perchlorate (27.5 mg, 0.133 mmol), and indium trichloride (11.7 mg, 0.0531 mmol) at room temperature. The reaction mixture was stittrd for 3.5 h. The reaction mixture was poured into a distilled water. The organic layer was separated the aqueous layer was extracted with ethyl acetate. The combined organic layer was dried over $MgSO_4$. The solvent was removed under reduced pressure. The residue was purified by preparative TLC on silica gel eluting with AcOEt-n-hexane (1:40) to give 4-(*tert*-butyl)-2-(4,4-dimethyl-1-phenylpent-1-yn-3-yl)phenol (**6l**) (52.6 mg, 62%) as a yellow oil.

IR (KBr, cm^{-1}) ν 3473, 2964, 2906, 2868, 1704, 1609, 1599, 1506, 1420, 1364, 1270, 1221, 1174, 1124, 1092, 821, 756, 691; ^{1}H NMR (600 MHz, $CDCl_3$) δ 1.08 (9H, s, CH_3x3), 1.30 (9H, s, CH_3×3), 3.87 (1H, brs, CH), 5.99 (1H, brs, OH), 6.77 (1H, d, *J* = 8.9 Hz, ArH), 7.16 (1H, dd, *J* = 8.9 and 2.7 Hz, ArH) 7.23 (1H, brs, ArH), 7.29-7.32 (3H, m, ArH), 7.44 (2H, dd, *J* = 2.7 and 6.2 Hz, ArH); ^{13}C NMR (150 MHz, $CDCl_3$) δ 27.7 (q×3), 31.5 (q×3), 34.0 (s), 36.7 (s), 45.5 (d), 85.2 (s), 89.9 (s), 116.1 (d), 123.1 (s), 123.3 (s), 124.9 (d), 128.1 (d), 128.3 (d×2), 129.1 (d), 131.6 (d×2), 142.5 (s), 152.0 (s); EIMS m/s 320 (M^+), 305 (M^+ -Me), 263 (M^+ -t-Bu); high resolution mass calcd for $C_{23}H_{28}O$: 320.2140, found m/z 320.2130. Anal. Calcd for $C_{23}H_{28}O$ (+0.664xH_2O): C, 83.10; H, 8.89. Found: C, 82.92; H, 8.81.

Synthesis of 4-(*tert*-butyl)-2-(1-cyclohexyl-3-phenylprop-2-yn-1-yl)phenol (**6m**).

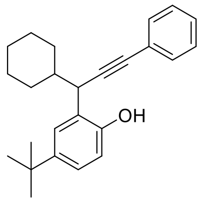

To a nitromethane (2.00 mL) solution of 1-cyclohexyl-3-phenylprop-2-yn-1-ol (**1d**) (50.0 mg, 0.233 mmol), 4-(*tert*-butyl)phenol (105 mg, 0.700 mmol), 1,1-binaphthol (13.4 mg, 0.0467 mmol), and tetrabutylammonium hexafluorophosphate (18.1 mg, 0.0467 mmol) were added silver perchlorate (24.2 mg, 0.117 mmol), and indium trichloride (10.3 mg, 0.0467 mmol) at room temperature. The reaction mixture was stirred for 30 min. The reaction mixture was poured into water (50.0 mL). The organic layer was separated the aqueous layer was extracted with ethyl acetate. The combined organic layer was dried over $MgSO_4$. The solvent was removed under reduced pressure. The residue was purified by preparative TLC on silica gel eluting with AcOEt-*n*-hexane (1:40) to give 4-(*tert*-butyl)-2-(1-cyclohexyl-3-phenylprop-2-yn-1-yl)phenol (**6m**) (61.0 mg, 75%) as white powders (mp 117-118 ºC).

IR (KBr, cm^{-1}) ν 3417, 2960, 2927, 2852, 1704, 1506, 1491, 1364, 1267, 1124, 822, 756, 691;^{1}H NMR (600 MHz, $CDCl_3$) δ 1.18-1.25 (4H, m, CH_2), 1.30 (9H, s, CH_3x3), 1.63-1.76 (6H, m, CH_2), 2.00-2.03 (1H, m, CH), 3.81 (1H, brd, *J* = 6.2 Hz, CH), 5.86 (1H, brs, OH), 6.77 (1H, d, *J* = 8.3 Hz, ArH), 7.16 (1H, d, *J* = 9.0 Hz, ArH), 7.23 (1H, d, *J* = 2.7 Hz, ArH), 7.29-7.30 (3H, m, ArH), 7.43-7.44 (2H, m, ArH); ^{13}C NMR (150 MHz, $CDCl_3$) δ 26.2 (t), 26.3 (t×2), 30.1 (t), 31.5 (q×3), 31.6 (t), 34.0 (s), 41.3 (d), 42.8 (d), 85.3 (s), 90.0 (s), 116.1 (d), 123.1 (s), 124.8 (d), 125.1 (s), 127.4 (d), 128.0 (d), 128.3 (d×2), 131.6 (d×2), 143.1 (s), 151.5 (s); EIMS/ m/z 346 (M^+), 331 (M^+-Me), 289 (M^+-t-Bu), 263 (M^+-C_6H_{11}); high resolution mass calcd for $C_{25}H_{30}O$: 346.2297, found m/z 346.2315. Anal. Calcd for $C_{25}H_{30}O$ (+1.08xH_2O): C, 82.05; H, 8.86. Found: C, 81.79; H, 8.60.

Synthesis of 2-(1-phenylpent-1-yn-3-yl)phenol (**6n**).

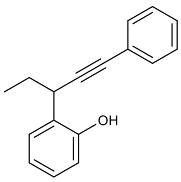

To a nitromethane (1.50 mL) solution of 1-phenylpent-1-yn-3-ol (**1a**) (50.0 mg, 0.312 mmol), phenol (58.7 mg, 0.624 mmol), 1,1'-binaphthol (24.0 mg, 0.0624 mmol) and tetrabutylammonium hexafluorophosphate (24.2 mg, 0.0624 mmol) were added silver perchlorate (32.4 mg, 0.156 mmol) and indium trichloride (13.8 mg, 0.0624 mmol) at room temperature. The reaction mixture was stirred for 12 h. The reaction mixture was poured into a water (50.0 mL). The organic layer was separated and the aqueous layer was extracted with AcOEt. The combined organic layer was dried over $MgSO_4$. The solvent was removed under reduced pressure. The residue was purified by preparative TLC on silica gel eluting with AcOEt-*n*-hexane (1:10) to give 4-(1-phenylpent-1-yl)phenol (**6n**) (31.4 mg, 43%) as a yellow oil and 2-(1-phenylpent-1-yn-3-yl)phenol (**6n**) (12.2 mg, 16.5%) as a yellow oil.

IR (KBr, cm^{-1}) ν 3455, 2965, 2927, 1702, 1598, 1455, 1234, 1105, 1088, 756, 692; ^{1}H NMR (600 MHz, $CDCl_3$) δ 1.08 (3H, t, *J* = 7.6 Hz, CH_3), 1.89 (2H, fix, *J* = 7.6 Hz, CH_2), 3.97 (1H, t, *J* = 7.6 Hz, CH), 5.80 (1H, s, OH), 6.83 (1H, dd, *J* = 8.3 and 1.4 Hz, ArH), 6.91 (1H, dt, *J* = 8.3 and 1.4 Hz, ArH), 7.15 (1H, dt, *J* = 8.3 and 1.4 Hz, ArH), 7.29-7.31 (4H, m, ArH), 7.44-7.45 (2H, m, ArH); ^{13}C NMR (150 MHz, $CDCl_3$) δ 12.1 (q), 29.4 (t), 35.5 (d), 84.5 (s), 90.1 (s), 116.5 (d), 120.9 (d), 123.0 (s), 127.2 (s), 128.1 (d×2), 128.3 (d×2), 129.3 (d), 131.7 (d×2), 153.5 (s); MS m/z 236 (M^+), 219 (M^+-OH), 207 (M^+-Et); high resolution mass calcd for $C_{17}H_{16}O$: 236.1201, found m/z 236.1204.

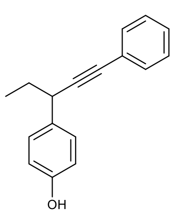

IR (KBr, cm^{-1}) ν 3373, 2930, 2235, 1614, 1598, 1513, 1490, 1444, 1376, 1245, 1173, 831, 757, 692; ^{1}H NMR (600 MHz, $CDCl_3$) δ 1.03 (3H, t, *J* = 6.8 Hz, Me), 1.78-1.86 (2H, m, CH_2), 3.72 (1H, t, *J* = 6.8 Hz, CH), 6.80 (2H, d, *J* = 8.9 Hz, ArH), 7.24-7.29 (3H, m, ArH), 7.44 (2H, dd, *J* = 2.1 and 7.6 Hz, ArH); ^{13}C NMR (150 MHz, $CDCl_3$) δ 11.8 (q), 31.7 (t), 39.1 (d), 83.2 (s), 91.8 (s), 115.2 (dx2), 123.8 (s), 127.7 (d), 128.2 (dx2), 128.7 (dx2), 131.6 (dx2), 134.3 (s), 154.2 (s); EIMS m/z 236 (M^+); high resolution mass calcd for $C_{17}H_{16}O$: 236.1201, found m/z 236.1144. Anal. Calcd for $C_{17}H_{16}O$ (+0.35xH_2O): C, 84.16; H, 6.94. Found: C, 83.94; H, 6.74.

Synthesis of 4-methyl-2-(1-phenylpent-1-yn-3-yl)phenol (**6o**).

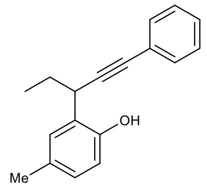

To a nitromethane (1.50 mL) solution of 1-phenylpent-1-yn-3-ol (**1a**) (50.0 mg, 0.312 mmol), *p*-cresol (169 mg, 1.56 mmol), and tetrabutylammonium hexafluorophosphate (24.2 mg, 0.0624 mmol) were added silver perchlorate (32.4 mg, 0.156 mmol) and indium trichloride (13.8 mg, 0.0624 mmol) at room temperature. The reaction mixture was stirred for 12 h. The reaction mixture was poured into a water (50.0 mL). The organic layer was separated and the aqueous layer was extracted with AcOEt. The combined organic layer was dried over $MgSO_4$. The solvent was removed under reduced pressure. The residue was purified by preparative TLC on silica gel eluting with AcOEt-$CHCl_3$-*n*-hexane (1:1:20) to give 4-methyl-2-(1-phenylpent-1-yn-3-yl)phenol (**6o**) (52.6 mg, 67%) as a yellow oil.

IR (KBr, cm^{-1}) ν 2966, 2930, 2872, 2234, 1704, 1612, 1508, 1442, 1362, 1263, 1229, 815, 758; ^{1}H NMR (600 MHz, $CDCl_3$) δ 1.08 (3H, t, *J* = 6.9 Hz, Me), 1.87 (2H, fifth, *J* = 6.9 Hz, CH_2), 2.27 (3H, s, Me), 3.92 (1H, t, *J* = 6.9 Hz, CH), 5.69 (1H, s, OH), 6.72 (1H, d, *J* = 8.2 Hz, ArH), 6.93 (1H, dd, *J* = 0.8 and 8.2 Hz, ArH), 7.09 (1H, d, *J* = 0.8 Hz, ArH), 7.28-7.29 (3H, m, ArH), 7.43-7.45 (2H, m, ArH); ^{13}C NMR (150 MHz, $CDCl_3$) δ 12.1 (q), 20.5 (q), 29.5 (t), 35.5 (d), 84.3 (s), 90.3 (s), 116.4 (d), 123.0 (s), 127.0 (s), 128.1 (d), 128.2 (dx2), 128.5 (d), 129.7 (d), 130.0 (s), 131.7 (dx2), 151.2 (s); EIMS m/z 250 (M^+), 221 (M^+-Et); high resolution mass calcd for $C_{18}H_{18}O$: 250.1358, found m/z 250.1349.

Synthesis of 4-(*tert*-butyl)-2-(1-phenylpent-1-yn-3-yl)phenol (**6p**).

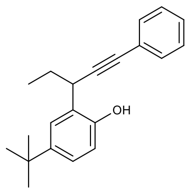

To a nitromethane (1.00 mL) solution of 1-phenylpent-1-yn-3-ol (**1a**) (50.0 mg, 0.312 mmol), 4-(*tert*-butyl)phenol (141 mg, 0.936 mmol), 1,1'-binaphthol (18.0 mg, 0.0624 mmol), and tetrabutylammonium hexafluorophosphate (24.2 mg, 0.0624 mmol) were added silver perchlorate (32.4 mg, 0.156 mmol) and indium tribromide (22.1 mg, 0.0624 mmol) at room temperature. The reaction mixture was stirred for 30 min. The reaction mixture was filtered through a pad of celite with chloroform and then poured into a saturated sodium hydrogencarbonate. The organic layer was separated and the aqueous layer was extracted with chloroform. The combined organic layer was dried over $MgSO_4$. The solvent was removed under reduced pressure. The residue was purified by preparative TLC on silica gel eluting with AcOEt-*n*-hexane (1:40) to give 4-(*tert*-butyl)-2-(1-phenylpent-1-yn-3-yl)phenol (**6p**) (77.1 mg, 85%) as a yellow oil.

IR (KBr, cm^{-1}) ν 3417, 2963, 2933, 281, 1703, 1609, 1599, 1505, 1420, 1364, 1228, 1126, 821, 756, 691; ^{1}H NMR (600 MHz, $CDCl_3$) δ 1.09 (3H, t, *J* = 6.9 Hz, CH_3), 1.30 (9H, s, CH_3×3), 1.86-1.91 (2H, m, CH_2), 3.94 (1H, t, *J* = 6.8 Hz, CH), 5.79 (1H, brs, OH), 6.77 (1H, d, *J* = 8.2 Hz, ArH), 7.16 (1H, dd, *J* = 2.7 and 8.2 Hz, ArH), 7.28-7.30 (3H, m, ArH), 7.31 (1H, d, *J* = 2.0 Hz, ArH), 7.43-7.45 (2H, m, ArH); ^{13}C NMR (150 MHz, $CDCl_3$) δ 12.1 (q), 29.5 (t), 31.5 (q×3), 34.1 (s), 36.1 (d), 84.6 (s), 90.3 (s), 116.1 (d), 123.0 (s), 124.9 (d), 126.2 (d), 126.4 (s), 128.1 (d), 128.3 (d×2), 131.6 (d×2), 143.5 (s), 151.2 (s); EIMS m/z 292 (M^+), 277 (M^+ -Me), 263 (M^+ -Et), 215(M^+ -Ph); high resolution mass calcd for $C_{21}H_{24}O$: 292.1827, found m/z 292.1782. Anal. Calcd for $C_{21}H_{24}O$ (+ 1 / 2 H_2O): C, 83.68; H, 8.36. Found: C, 83.54; H, 8.36.

Synthesis of 4-methoxy-2-(1-phenylpent-1-yn-3-yl)phenol (**6q**).

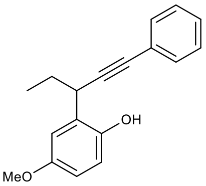

To a nitromethane (1.50 mL) solution of 1-phenylpent-1-yn-3-ol (**1a**) (50.0 mg, 0.312 mmol), 4-methoxyphenol (57.8 mg, 0.624 mmol), 1,1'-binaphthol (17.9 mg, 0.0624 mmol) and tetrabutylammonium hexafluorophosphate (24.2 mg, 0.0624 mmol) were added silver perchlorate (32.4 mg, 0.156 mmol) and indium trichloride (14.0 mg, 0.0624 mmol) at room temperature. The reaction mixture was stirred for 12 h. The reaction mixture was poured into water (50.0 mL). The organic layer was separated and the aqueous layer was extracted with AcOEt. The combined organic layer was dried over $MgSO_4$. The solvent was removed under reduced pressure. The residue was purified by preparative TLC on silica gel eluting with AcOEt-*n*-hexane (1:30) to give 4-methoxy-2-(1-phenylpent-1-yn-3-yl)phenol (**6q**) (30.0 mg, 36%) as a yellow oil and 4-methoxy-3-(1-phenylpent-1-yn-3-yl)phenol (**6q**) (14.3 mg, 17%) as a yellow oil.

IR (KBr, cm^{-1}) ν 2964, 2930, 1507, 1433, 1265, 1201, 1088, 1039, 805, 757; ^{1}H NMR (600 MHz, $CDCl_3$) δ 1.09 (3H, t, *J* = 7.6 Hz, CH_3), 1.89 (2H, sex, *J* = 7.5 Hz, CH_2), 3.77 (3H, s, OCH_3), 3.93 (1H, dd, *J* = 6.9 and 7.6 Hz, CH), 5.40 (1H, brs, OH), 6.70 (1H, dd, *J* = 2.8 and 8.3 Hz, ArH), 6.77 (1H, dd, *J* = 2.8 and 8.3 Hz, ArH), 6.90 (1H, d, *J* = 8.3 Hz, ArH), 7.30-7.31 (3H, m, ArH), 7.44-7.45 (2H, m, ArH); ^{13}C NMR (150 MHz, $CDCl_3$) δ 12.0 (q), 29.3 (t), 35.7 (d), 55.7 (q), 84.4 (s), 90.0 (s), 112.9 (d), 114.9 (d), 117.1 (d), 123.0 (s), 128.1 (d), 128.3 (dx2), 128.4 (s), 131.7 (dx2), 147.4 (s), 153.7 (s); EIMS m/z 266 (M^+), 251 (M^+ -Me), 237 (M^+ -Et); high resolution mass calcd for $C_{18}H_{18}O_2$: 266.1307, found m/z 266.1329. Anal. Calcd for $C_{18}H_{18}O_2$ (+ 3 / 4 H_2O): C, 77.25; H, 7.02. Found: C, 77.42; H, 6.96.

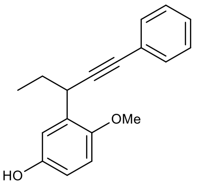

IR (KBr, cm^{-1}) ν 3360, 2964, 2929, 2358,2329, 1702, 1501, 1462, 1440, 1285, 1212, 1179, 1033, 809, 758, 692; ^{1}H NMR (600 MHz, $CDCl_3$) δ 1.56 (3H, t, *J* = 7.3 Hz, CH_3), 1.67-1.76 (1H, m, CH), 1.79-1.90 (1H, m, CH), 3.79 (3H, s, OCH_3), 4.22 (1H, dd, *J* = 5.5 and 8.3 Hz, CH), 4.59 (1H, brs, OH), 6.68 (1H, dd, *J* = 3.2 and 8.7 Hz, ArH), 6.74 (1H, d, *J* = 9.2 Hz, ArH), 7.11 (1H, d, *J* = 2.7 Hz, ArH), 7.28-7.31 (3H, m, ArH), 7.44-7.46 (2H, m, ArH); ^{13}C NMR (150 MHz, $CDCl_3$) δ 11.8 (q), 29.7 (t), 33.1 (d), 56.1 (q), 82.7 (s), 91.7 (s), 111.7 (d), 113.7 (d), 116.0 (d), 123.9 (s), 127.6 (d), 127.7 (dx2), 128.2 (dx2), 131.7 (s), 149.3 (s), 150.5 (s); MS m/z 266 (M+), 251 (M+-Me), 237 (M+-Et); high resolution mass calcd for $C_{18}H_{18}O_2$: 266.1307, found m/z 266.1290.

Synthesis of 4-chloro-2-(1-phenylpent-1-yn-3-yl)phenol (**6r**).

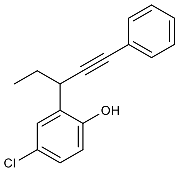

To a nitromethane (1.50 mL) solution of 1-phenylpent-1-yn-3-ol (**1a**) (50.0 mg, 0.312 mmol), 4-chlorophenol (80.2 mg, 0.624 mmol),1,1'-binaphthol (17.9 mg, 0.0624 mmol), and tetrabutylammonium hexafluorophosphate (24.2 mg, 0.0624 mmol) were added silver perchlorate (32.4 mg, 0.156 mmol) and indium trichloride (14.0 mg, 0.0624 mmol) at room temperature. The reaction mixture was stirred overnight. The reaction mixture was poured into water (50.0 mL). The organic layer was separated and the aqueous layer was extracted with AcOEt. The combined organic layer was dried over $MgSO_4$. The solvent was removed under reduced pressure. The residue was purified by preparative TLC on silica gel eluting with AcOEt-*n*-hexane (1:40) to give 4-chloro-2-(1-phenylpent-1-yn-3-yl)phenol (**6r**) (39.4 mg, 47%) as a yellow oil.

IR (KBr, cm^{-1}) ν 3441, 2927, 2854, 1702, 1491, 1417, 1335, 1269, 1220, 1167, 1116, 816, 756, 691, 651; ^{1}H NMR (600 MHz, $CDCl_3$) δ 1.08 (3H, t, *J* = 7.6 Hz, CH_3), 1.85-1.90 (2H, m, CH_2), 3.93 (1H, t, *J* = 7.5 Hz, CH), 5.76 (1H, brs, OH), 6.77 (1H, d, *J* = 8.3 Hz, ArH), 7.11 (1H, dd, *J* = 2.8 and 8.3 Hz, ArH), 7.29 (1H, d, *J* = 2.8 Hz, ArH), 7.31-7.32 (3H, m, ArH), 7.44-7.46 (2H, m, ArH); ^{13}C NMR (150 MHz, $CDCl_3$) δ 11.9 (q), 29.3 (t), 35.3 (d), 84.8 (s), 89.3 (s), 117.7 (d), 122.7 (s), 125.5 (s), 127.9 (d), 128.3 (d×2), 128.3 (d), 129.0 (d), 129.0 (s), 131.7

(d×2), 152.1 (s); MS m/z 266 (M^+), 251 (M^+-Me), 237 (M^+-Et). High resolution mass calcd for $C_{17}H_{15}ClO$: 266.1307, found m/z 266.1329. Anal. Calcd for $C_{17}H_{15}ClO$(+ 3 / 4 H_2O): C, 77.25; H, 7.02. Found: C, 77.42; H, 6.96.

Synthesis of 1-(1-phenylpent-1-yn-3-yl)naphthalen-2-ol (**6s**).

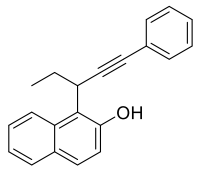

To a nitromethane (1.50 mL) solution of 1-phenylpent-1-yn-3-ol (**1a**) (50.0 mg, 0.312 mmol), 2-naphthol (90.0 mg, 0.624 mmol), and tetrabutylammonium hexafluorophosphate (24.2 mg, 0.0624 mmol) were added silver perchlorate (32.4 mg, 0.156 mmol) and indium trichloride (14.0 mg, 0.0624 mmol) at room temperature. The reaction mixture was stirred for 30 min. The reaction mixture was poured into water (50.0 mL). The organic layer was separated and the aqueous layer was extracted with AcOEt. The combined organic layer was dried over $MgSO_4$. The solvent was removed under reduced pressure. The residue was purified by preparative TLC on silica gel eluting with AcOEt-*n*-hexane (1:20) to give 1-(1-phenylpent-1-yn-3-yl)naphthalen-2-ol (**6s**) (60.8 mg, 68%) as a yellow oil.

IR (KBr, cm^{-1}) ν 3410, 3058, 2967, 2931, 2872, 1700, 1671, 1597, 1512, 1490, 1441, 1285, 1235, 1068, 957, 849, 815, 757, 691, 532; ^1H NMR (600 MHz, $CDCl_3$) δ 1.16 (3H, t, *J* = 6.9 Hz, CH_3), 1.93-1.97 (1H, m, CH_2), 2.04-2.07 (1H, m, CH_2), 4.74 (1H, t, *J* =7.6 and 6.9 HZ, olefinicH), 7.16 (1H, d, *J* =9.0 Hz, ArH), 7.30-7.31 (3H, m, ArH), 7.33 (1H, t, *J* =7.6 Hz, ArH), 7.45-7.47 (2H, m, ArH), 7.49 (1H, t, *J* = 7.6 Hz, ArH), 7.69 (1H, d, *J* = 8.9 Hz, ArH), 7.78 (1H, d, *J* = 7.5 Hz, ArH), 7.98 (1H, brs, OH); ^{13}C NMR (150 MHz, $CDCl_3$) δ 12.7 (q), 28.6 (t), 31.1 (d), 85.8 (s), 89.4 (s), 117.4 (s), 119.5 (d), 122.0 (d), 122.3 (s), 123.1 (d), 126.6 (d), 128.3 (d×2), 128.5 (d), 128.9 (d), 129.1 (d), 129.5 (s), 131.7 (d×2), 131.9 (s), 152.6 (s); EIMS m/z 286 (M^+), 257 (M^+ -Et); high resolution mass calcd for $C_{21}H_{18}O$: 286.1358, found m/z 286.1359.

Synthesis of (4,4-dimethyl-3-propoxypent-1-yn-1-yl)benzene (**8a**).

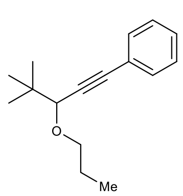

To a nitromethane (1.50 mL) solution of 4,4-dimethyl-1-phenylpent-1-yn-3-ol (**1c**) (58.7 mg, 0.312 mmol), propan-1-ol (188 mg, 3.12 mmol), 1,1-binaphthol (17.9 mg, 0.0624 mmol), and tetrabutylammonium hexafluorophosphate (24.2 mg, 0.0624 mmol) were added silver perchlorate (25.9 mg, 0.125 mmol), and indium trichloride (13.8 mg, 0.0624 mmol). The reaction mixture was refluxed for 0.5 h. The reaction mixture was poured into a distilled water. The organic layer was separated the aqueous layer was extracted with ethyl acetate. The combined organic layer was dried over $MgSO_4$. The solvent was removed under reduced pressure. The residue was purified by preparative TLC on silica gel eluting with n-hexane to give (4,4-dimethyl-3-propoxypent-1-yn-1-yl)benzene (**8a**)(54.1 mg, 75%) as a yellow oil.

IR (KBr, cm^{-1}) ν 2959, 2932, 2871, 1490, 1479, 1463, 1390, 1363, 1323, 1094, 756, 690; ^1H NMR (600 MHz, $CDCl_3$) δ 0.95 (3H, t, CH_3), 1.05 (9H, s, CH_3×3), 3.34 (1H, dt, *J* = 6.9 and 8.9 Hz CH), 3.80 (1H, dt, *J* = 6.9 and 8.9 Hz, CH), 3.82 (1H, s, CH), 7.29-7.30 (3H, m, ArH) 7.43-7.45 (2H, m, ArH); ^{13}C NMR (150 MHz, $CDCl_3$) δ 10.7 (q), 22.8 (t), 25.9 (q×3), 35.9 (s), 71.5 (t), 79.1 (d), 85.9 (s), 88.0 (s), 123.2 (s), 128.0 (d), 128.2 (d×2), 131.7 (d×2); EIMS m/s 230 (M^+), 215 (M^+ -Me), 188 (M^+ -C_3H_6) 173 (M^+ -t-Bu); high resolution mass calcd for $C_{23}H_{28}O$: 230.1671, found m/z 230.1619.

Synthesis of (3-cyclohexyl-3-(isopentyloxy)prop-1-yn-1-yl)benzene (**8b**).

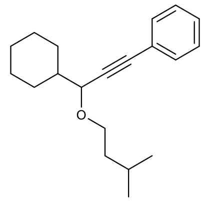

To a nitromethane (1.50 mL) solution of 1-cyclohexyl-3-phenylprop-2-yn-1-ol (**1d**) (66.9 mg, 0.312 mmol), 3-methylbutan-1-ol (138 mg, 1.56 mmol), 1,1-binaphthol (17.9 mg, 0.0624 mmol), and tetrabutylammonium hexafluorophosphate (24.2 mg, 0.0624 mmol) were added silver perchlorate (25.9 mg, 0.125 mmol), and indium trichloride (13.8 mg, 0.0624 mmol). The reaction mixture was stirred at 50 °C for 5 h. The reaction mixture was poured into a distilled water. The organic layer was separated the aqueous layer was extracted with ethyl acetate. The combined organic layer was dried over $MgSO_4$. The solvent was removed under reduced pressure. The residue was purified by preparative TLC on silica gel eluting with n-hexane to give (3-cyclohexyl-3-(isopentyloxy)prop-1-yn-1-yl)benzene (**8b**) (58.8 mg, 63%) as a yellow oil.

IR (KBr, cm^{-1}) ν 2953, 2926, 2853, 2852, 1490, 1466, 1450, 1329, 1092, 1030, 755, 690; ^1H NMR (600 MHz, $CDCl_3$) δ 0.91 (3H, t, *J* = 6.9 Hz, Me), 0.92 (3H, t, *J* = 6.2 Hz, Me) 1.12-1.28 (5H, m, 5CH_2), 1.51 (2H, q, *J* = 6.9 Hz, CH_2), 1.66-1.78 (5H, m, CH_2), 1.93 (2H, brs, CH_2), 3.43 (1H, dt, *J* = 6.8 Hz and 9.6 Hz, CH), 3.83 (1H, dt, *J* = 6.9 and 8.9 Hz, CH), 3.98 (1H, d, *J* = 6.2 Hz, CH) 7.29-7.30 (3H, m, ArH), 7.43-7.45 (2H, m, ArH); ^{13}C NMR (150 MHz, $CDCl_3$) δ 22.6 (d), 25.1 (q×2), 26.0 (t×2), 26.5 (t), 28.7 (t), 29.2 (t), 38.5 (t), 42.9 (d), 67.5 (t), 75.1 (s), 86.0 (s), 88.1 (s), 123.1 (s), 128.0 (d), 128.2 (d×2), 131.7 (d×2); EIMS/ m/z 284 (M^+), 201 (M^+ -C_6H_{11}); high resolution mass calcd for $C_{25}H_{30}O$: 284.2140, found m/z 284.2142.

Synthesis of (3-(cyclohexyloxy)pent-1-yn-1yl)benzene (**8c**).

To a nitromethane (1.50 mL) solution of 1-phenylpent-1-yn-3-ol (**1a**) (50.0 mg, 0.312 mmol), cyclohexanol (93.7 mg, 0.936 mmol), 1,1-binaphthol (17.9 mg, 0.0624 mmol), and tetrabutylammonium hexafluorophosphate (24.2 mg, 0.0624 mmol) were added silver perchlorate (25.9 mg, 0.125 mmol), and indium trichloride (13.8 mg, 0.0624 mmol). The reaction mixture was refluxed for 5 min. The reaction mixture was poured into water. The organic layer was separated the aqueous layer was extracted with ethyl acetate. The combined organic layer

was dried over $MgSO_4$. The solvent was removed under reduced pressure. The residue was purified by preparative TLC on silica gel eluting with AcOEt-*n*-hexane (1:40) to give (3-(cyclohexyloxy)pent-1-yn-1yl)benzene (**8c**) (44.6 mg, 59%) as a yellow oil.

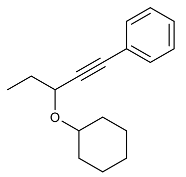

IR (KBr, cm^{-1}) ν 2931, 2856, 2229, 1716, 1489, 1450, 1336, 1101, 1074, 756, 691.34 (4H, m, CH_2), 1.38-1.44 (1H, m, CH), 1.52-1.55 (1H, m, CH), 1.75-1.84 (4H, m, CH_2), 1.94-1.98 (2H, m, CH_2), 3.62-3.65 (1H, m, CH), 4.29 (1H, t, *J* = 6.8 Hz, CH), 7.29-7.30 (3H, m, ArH), 7.42-7.44 (2H, m, ArH); ^{13}C NMR (150 MHz, $CDCl_3$) d 10.0 (q), 24.1 (t), 24.3 (t), 25.8 (t), 29.5 (t), 31.5 (t), 33.4 (t), 68.3 (d), 75.7 (d), 84.6 (s), 90.0 (s), 123.1 (s), 128.0 (d), 128.2(d×2), 131.7 (d×2); EIMS m/s 159 (M^+-C_6H_{11}), 143 (M^+-$C_6H_{11}O$). Anal. Calcd for $C_{17}H_{22}O$: C, 84.25; H, 9.15. Found: C, 84.23; H, 8.89.

Synthesis of (3-(allyloxy)pent-1-yn-1-yl)benzene (**8d**)

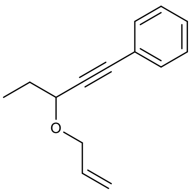

To a nitromethane (1.50 mL) solution of 1-phenylpent-1-yn-3-ol (**1a**) (50.0 mg, 0.312 mmol), prop-2-en-1-ol (90.6 mg, 1.56 mmol), 1,1-binaphthol (17.9 mg, 0.0624 mmol), and tetrabutylammonium hexafluorophosphate (24.2 mg, 0.0624 mmol) were added silver perchlorate (25.9 mg, 0.125 mmol), and indium trichloride (13.8 mg, 0.0624 mmol). The reaction mixture was stirred at 50 °C for 6 h. The reaction mixture was poured into a distilled water. The organic layer was separated the aqueous layer was extracted with ethyl acetate. The combined organic layer was dried over $MgSO_4$. The solvent was removed under reduced pressure. The residue was purified by preparative TLC on silica gel eluting with n-hexane to give (3-(allyloxy)pent-1-yn-1-yl)benzene (**8d**) (29.7 mg, 41%) as a yellow oil.

IR (KBr, cm^{-1}) ν 2969, 2935, 2854, 2230, 1489, 1463, 1335, 1100, 1071, 925, 691; ^1H NMR (600 MHz, $CDCl_3$) δ 1.07 (3H, t, *J* = 7.5 Hz, CH_3), 1.81-1.88 (2H, m, CH_2), 4.05 (1H, dd, *J* = 12.6 and 6.3 Hz, CH_2), 4.25 (1H, t, *J* = 6.8 Hz, CH), 4.33 (1H, dd, *J* = 12.6 and 7.5 Hz, CH), 5.21 (1H, brd, *J* = 10.3 Hz, CH), 5.34 (1H, dd, *J* = 17.8 and 1.7 Hz, CH), 5.94-5.99 (1H, m, CH), 7.30 (3H, t, *J* = 3.5 Hz, ArH), 7.43-7.45 (2H, m, ArH); ^{13}C NMR (150 MHz, $CDCl_3$) δ 9.8 (q), 28.9 (t), 69.7 (t), 70.6 (d), 85.7 (s), 88.2 (s), 117.3 (t), 122.9 (s), 128.2 (d×3), 131.7 (d×2), 134.6 (d); EIMS m/s 143 (M+-allylO). Anal. Calcd for $C_{14}H_{16}O$: C, 83.96; H, 8.05. Found: C, 83.95; H, 7.86.

Synthesis of (3-(2-methoxyethoxy)pent-1-yn-1-yl)benzene (**8e**)

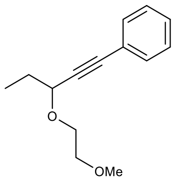

To a nitromethane (1.50 mL) solution of 1-phenylpent-1-yn-3-ol (**1a**)(50.0 mg, 0.312 mmol), 2-methoxyethan-1-ol (119 mg, 1.56 mmol), tetrabutylammonium hexafluorophosphate (24.2 mg, 0.0624 mmol), and (R)-1,1'-binaphthol (17.9 mg, 0.0624 mmol) were added silver perchlorate (25.9 mg, 0.125 mmol) and indium trichloride (13.8 mg, 0.0624 mmol) at 60°C. The reaction mixture was stirred for 2 h. The reaction mixture was poured into water (50.0 mL). The organic layer was separated and the aqueous layer was extracted with AcOEt. The combined organic layer was dried over $MgSO_4$. The solvent was removed under reduced pressure. The residue was purified by preparative TLC on silica gel eluting with AcOEt-*n*-hexane (1:30) to give (3-(2-methoxyethoxy)pent-1-yn-1-yl)benzene (**8e**) (50.9 mg, 69%) as a yellow oil.

IR (KBr, cm^{-1}) 2968, 2928, 2876, 2230, 1490, 1457, 1444, 1335, 1127, 1107, 757, 691; ^1H NMR (600 MHz, $CDCl_3$) δ 1.07 (3H, t, *J* = 7.6 Hz, CH_3), 1.81-1.90 (2H, m, CH_2), 3.40 (3H, s, OMe), 3.60-3.62 (2H, m, CH_2), 3.62-3.66 (1H, m, CH), 3.94-3.97 (1H, m, CH), 4.26 (1H, t, *J* = 6.9 Hz, CH), 7.29-7.31 (3H, m, ArH), 7.43-7.44 (2H, m, ArH); ^{13}C NMR (150 MHz, $CDCl_3$) δ 9.8 (q), 28.8 (t), 59.0 (s), 67.9 (t), 71.8 (t), 85.8 (s), 88.1 (s), 122.8 (s), 128.2 (dx2), 131.7 (dx2); EIMS m/z 218 (small M^+). Anal. Calcd for $C_{14}H_{18}O_2$(+1/15 H_2O): C, 76.61; H, 8.33. Found: C, 76.83; H, 8.30.

Synthesis of 1-bromo-2-(((1-phenylpent-1-yn-3-yl)oxy)methyl)benzene (**8f**)

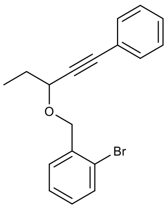

To a nitromethane (1.50 mL) solution of 1-phenylpent-1-yn-3-ol (**1a**) (50.0 mg, 0.312 mmol), (2-bromophenyl)methanol (175 mg, 0.936 mmol), tetrabutylammonium hexafluorophosphate (24.2 mg, 0.0624 mmol), and (R)-1,1'-binaphthol (17.9 mg, 0.0624 mmol) were added silver perchlorate (25.9 mg, 0.125 mmol) and indium trichloride (13.8 mg, 0.0624 mmol) at room temperature. The reaction mixture was stirred for 3 h. The reaction mixture was poured into water (50.0 mL). The organic layer was separated and the aqueous layer was extracted with AcOEt. The combined organic layer was dried over $MgSO_4$. The solvent was removed under reduced pressure. The residue was purified by preparative TLC on silica gel eluting with AcOEt-*n*-hexane (1:40) to give 1-bromo-2-(((1-phenylpent-1-yn-3-yl)oxy)methyl)benzene (**8f**) (73.7 mg, 72%) as a yellow oil.

IR (KBr, cm^{-1}) 2967, 2933, 2874, 2853, 2230, 1490, 1464, 1455, 1442, 1334, 1101, 1070, 1044, 1027, 952, 753, 690; ^1H NMR (400 MHz, $CDCl_3$) δ 1.10 (3H, t, *J* = 7.5 Hz, CH_3), 1.86-1.95 (2H, m, CH_2), 4.33 (1H, t, *J* = 6.3 Hz, CH), 4.66 (1H, d, *J* = 13.2 Hz, CH), 4.91 (1H, d, *J* = 13.1 Hz, CH), 7.13 (1H, t, *J* = 8.1 Hz, ArH), 7.29-7.32 (4H, m, ArH), 7.44-7.46 (2H, m, ArH), 7.53 (2H, d, *J* = 7.4 Hz, ArH); ^{13}C NMR (150 MHz, $CDCl_3$) δ 9.9 (q), 29.0 (t), 70.1 (t), 71.2 (d), 86.1 (s), 87.9 (s), 122.8 (s), 122.9 (s), 127.3 (d), 128.2 (dx2), 128.3 (d), 128.9 (d), 129.4 (d), 131.7 (dx2), 132.5 (d), 137.6 (s); EIMS m/z 328 (M^+); high resolution mass calcd for $C_{18}H_{17}BrO$: 328.0463, found m/z 328.0428. Anal. Calcd for $C_{18}H_{17}BrO$: C, 65.67; H, 5.20. Found: C, 65.85; H, 5.37.

Synthesis of *tert*-butyl(1-phenylpent-1-yn-3-yl)sulfane (**8g**).

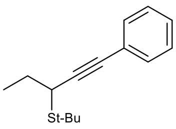

To a nitromethane (1.00 mL) solution of 1-phenylpent-1-yn-3-ol (**1a**) (50.0 mg, 0.312 mmol), tert-butyl mercaptane (282 mg, 3.12 mmol), and tetrabutylammonium hexafluorophosphate (24.2 mg, 0.0624 mmol), 1,1'-binaphthol (17.9 mg, 0.0624 mmol) were added silver perchlorate (32.4 mg, 0.156 mmol) and indium trichloride (13.8 mg, 0.0624 mmol) at room temperature. The reaction mixture was stirred for 48 h. The reaction mixture was poured into water (50.0 mL). The organic layer was separated and the aqueous layer was extracted with AcOEt. The combined organic layer was dried over $MgSO_4$. The solvent was removed under reduced pressure. The residue was purified by preparative TLC on silica gel eluting with *n*-hexane to give tert-butyl(1-phenylpent-1-yn-3-yl)sulfane (**8g**) (59.9 mg, 83%) as a yellow oil.

IR (KBr, cm^{-1}) ν 2969, 2926, 2861, 2228, 1490, 1458, 1443, 1364, 1162, 756, 691; ^{1}H NMR (500 MHz, $CDCl_3$) δ 1.15 (3H, t, *J* = 7.6 Hz, Me), 1.45 (9H, s, Mex3), 1.81-1.86 (2H, m, CH_2), 3.67 (1H, dd, *J* = 5.4 and 7.5 Hz, CH), 7.27-7.28 (3H, m, ArH), 7.39-7.41 (2H, m, ArH); ^{13}C NMR (150 MHz, $CDCl_3$) δ 11.8 (q), 29.7 (t), 31.3 (qx3), 34.2 (d), 43.9 (s), 83.2 (s), 91.5 (s), 123.5 (s), 127.8 (d), 128.1 (dx2), 131.4 (dx2); EIMS m/z 175 (M^+-St-Bu).

Synthesis of (4-methoxyphenyl)(1-1phenylpent-1-yn-3-yl)sulfane (**8h**).

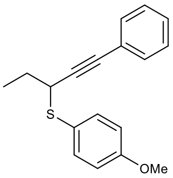

To a nitromethane (1.00 mL) solution of 1-phenylpent-1-yn-3-ol (**1a**) (50.0 mg, 0.312 mmol), 4-methoxybenzenethiol (219 mg, 1.56 mmol), and tetrabutylammonium hexafluorophosphate (24.2 mg, 0.0624 mmol) were added silver perchlorate (32.4 mg, 0.156 mmol) and indium tribromide (22.1 mg, 0.0624 mmol) at room temperature. The reaction mixture was stirred for 5 min. The reaction mixture was filtered through a pad of celite with chloroform and then poured into a saturated sodium hydrogencarbonate. The organic layer was separated and the aqueous layer was extracted with chloroform. The combined organic layer was dried over $MgSO_4$. The solvent was removed under reduced pressure. The residue was purified by preparative TLC on silica gel eluting with *n*-hexane to give (4-methoxyphenyl)(1-phenylpent-1-yn-3-yl)sulfane (**8h**) (38.6 mg, 44%) as a yellow oil.

IR (KBr, cm^{-1}) ν 3447, 2966, 2927, 1714, 1592, 1493, 1286, 1247, 1173, 1032, 828, 758, 691; ^{1}H NMR (600 MHz, $CDCl_3$) δ 1.14 (3H, t, *J* = 6.9 Hz, CH_3), 1.77-1.85 (2H, m, CH_2), 3.80 (3H, s, OCH_3), 6.86-6.87 (2H, brd, *J* = 8.9 Hz, ArH), 7.27-7.28 (3H, m, ArH), 7.34-7.35 (2H, m, ArH), 7.54 (2H, brd, *J* = 8.9 Hz, ArH); ^{13}C NMR (150 MHz, $CDCl_3$) δ 11.8 (q), 28.4 (t), 42.2 (d), 55.3 (q), 84.8 (s), 89.2 (s), 114.3 (d×2), 123.2 (s), 123.8 (s), 127.9 (d), 128.1 (d×2), 131.6 (d×2), 136.8 (d×2), 160.1 (s); EIMS m/z 282 (M^+), 267 (M^+ -Me), 143 (M^+ -SC_6H_4OMe); high resolution mass calcd for $C_{18}H_{18}O_5S$: 282.1078, found m/z 282.1098. Anal. Calcd for $C_{18}H_{18}O_5S$ (+ 1 / 10 H_2O): C, 76.07; H, 6.46. Found: C, 76.24; H, 6.61.

Synthesis of naphthalen-2-yl(1-phenylpent-1-yn-3-yl)sulfane (**8i**).

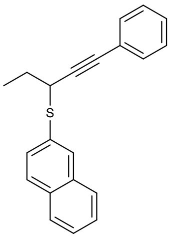

To a nitromethane (1.00 mL) solution of 1-phenylpent-1-yn-3-ol (**1a**) (50.0 mg, 0.312 mmol), naphthalene-2-thiol (65.0 mg, 0.406 mmol), and tetrabutylammonium hexafluorophosphate (24.2 mg, 0.0624 mmol) were added silver perchlorate (32.4 mg, 0.156 mmol) and indium trichloride (13.8 mg, 0.0624 mmol) at room temperature. The reaction mixture was stirred for 2 h. The reaction mixture was filtered through a pad of celite with chloroform and then poured into a saturated sodium hydrogencarbonate. The organic layer was separated and the aqueous layer was extracted with chloroform. The combined organic layer was dried over $MgSO_4$. The solvent was removed under reduced pressure. The residue was purified by preparative TLC on silica gel eluting with chloroform-*n*-hexane (1:10) to give naphthalen-2-yl(1-phenylpent-1-yn-3-yl)sulfane (**8i**) (35.6 mg, 38%) as a yellow oil.

IR (KBr, cm^{-1}) ν 3426, 3054, 2967, 2929, 1713, 1596, 1490, 1442, 857, 815, 756, 691, 531, 476; ^{1}H NMR (500 MHz, $CDCl_3$) δ 1.18 (3H, t, *J* = 7.4 Hz, CH_3), 1.83-1.96 (2H, m, CH_2), 4.07 (1H, dd, *J* = 8.0 and 5.7 Hz, CH), 7.23-7.27 (3H, m, ArH), 7.31-7.33 (2H, m, ArH), 7.45-7.49 (2H, m , ArH), 7.63 (1H, dd, *J* = 8.3 and 2.3 Hz, ArH), 7.77-7.82 (3H, m, ArH), 8.06 (1H, s, ArH); ^{13}C NMR (150 MHz, $CDCl_3$) δ 11.8 (q), 28.6 (t), 41.1 (d), 84.8 (s), 88.9 (s), 123.0 (s), 126.3 (d), 126.4 (d), 127.5 (d), 127.6 (d), 128.0 (d), 128.1 (d×2), 128.2 (d), 130.3 (d), 131.3 (s), 131.6 (d×2), 132.1 (d), 132.5 (s), 133.5 (s); MS m/z 302 (M^+), 273 (M^+ -Et), 143 (M^+ -$SC_{10}H_7$); high resolution mass calcd for $C_{17}H_{18}S$: 302.1129, found m/z 302.1150. Anal. Calcd for $C_{17}H_{18}S$ (+ 1 / 10 H_2O): C, 82.91; H, 6.03. Found: C, 82.91; H, 6.04.

Synthesis of *N*-(1-phenylpent-1-yn-3-yl)methanesulfonamide (**8j**).

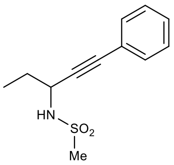

To a nitromethane (1.00 mL) solution of 1-phenylpent-1-yn-3-ol (**1a**) (50.0 mg, 0.312 mmol), methanesulfonamide (59.4 mg, 0.624 mmol), and tetrabutylammonium hexafluorophosphate (24.2 mg, 0.0624 mmol) were added silver perchlorate (32.4 mg, 0.156 mmol) and indium tribromide (22.1 mg, 0.0624 mmol) at room temperature. The reaction mixture was stirred for 30 min. The reaction mixture was filtered through a pad of celite with chloroform and then poured into a saturated sodium hydrogencarbonate. The organic layer was separated and the aqueous layer was extracted with chloroform. The combined organic layer was dried over $MgSO_4$. The solvent was removed under reduced pressure. The residue was purified

by preparative TLC on silica gel eluting with AcOEt-*n*-hexane (1:40) to give *N*-(1-phenylpent-1-yn-3-yl)methanesulfonamide (**8j**) (27.4 mg, 37%) as a yellow oil, and (oxybis(pent-1-yne-3,1-diyl))dibenzene (4.6 mg, 5%) as a yellow oil.

IR (KBr, cm^{-1}) ν 3270, 2972, 1705, 1491, 1443, 1325, 1157, 1125, 1061, 979, 760, 692, 526; ^{1}H NMR (600 MHz, $CDCl_3$) δ 1.11 (3H, t, *J* = 7.6 Hz, CH_3), 1.83 (2H, sex, *J* = 7.5 Hz, CH_2), 3.14 (3H, s, CH_3), 4.34 (1H, dt, *J* = 7.6 and 9.6 Hz, CH), 4.71 (1H, d, *J* = 9.6 Hz, NH), 7.31-7.35 (3H, m, ArH), 7.40-7.42 (2H, m, ArH); ^{13}C NMR (150 MHz, $CDCl_3$) δ 10.0 (q), 29.8 (t), 41.4 (d), 47.5 (q), 85.0 (s), 87.4 (s), 122.0 (s), 128.4 (d×2), 128.7 (d), 131.6 (d×2); EIMS m/z 237 (M^+), 222 (M^+ -Me), 208 (M^+ -Et), 157 (M^+ -SO_2Me), 141 (M^+ -$NHSO_2$Me); high resolution mass calcd for $C_{12}H_{15}NO_2S$: 237.0823, found m/z 237.0797. Anal. Calcd for $C_{12}H_{15}NO_2S$: C, 60.73; H, 6.37; N, 5.90. Found: C, 61.01; H, 6.52; N, 5.78.

Synthesis of 4-methyl-*N*-(1-phenylpent-1-yn-3-yl)benzenesulfonamide (**8k**).

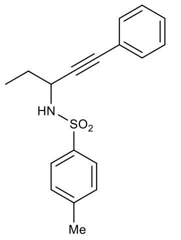

To a nitromethane (1.30 mL) solution of 1-phenylpent-1-yn-3-ol (**1a**) (50.0 mg, 0.312 mmol), 4-methylbenzenesulfonamide (80.1 mg, 0.468 mmol), tetrabutylammonium hexafluorophosphate (24.2 mg, 0.0624 mmol), and (R)-1,1'-binaphthol (24.2 mg, 0.0624 mmol) were added silver perchlorate (32.4 mg, 0.156 mmol) and indium trichloride (14.0 mg, 0.0624 mmol) at room temperature. The reaction mixture was stirred overnight. The reaction mixture was poured into water (50.0 mL). The organic layer was separated and the aqueous layer was extracted with chloroform. The combined organic layer was dried over $MgSO_4$. The solvent was removed under reduced pressure. The titled compound was precipitated from *n*-hexane and the filtrate was purified by preparative TLC on silica gel eluting with AcOEt-$CHCl_3$-*n*-hexane (1:1:20) to give 4-methyl-*N*-(1-phenylpent-1-yn-3-yl)benzenesulfonamide (**8k**) (42.4 mg, 43%) as a white crystal (mp 140-142°C from chloroform-*n*-hexane).

IR (KBr, cm^{-1}) ν 3474, 3275, 2931, 1491, 1428, 1156, 1091, 996, 819, 762, 976, 575, 543; ^{1}H NMR (600 MHz, $CDCl_3$) δ 1.06 (3H, t, *J* = 7.6 Hz, CH_3), 1.76-1.81 (2H, m, CH_2), 2.32 (3H, s, CH_3), 4.23-4.25 (1H, dt, *J* = 7.5 and 9.6 Hz, CH), 4.70 (1H, d, *J* = 9.6 Hz, NH), 7.05 (2H, d, *J* = 6.8 Hz, ArH), 7.22 (2H, d, *J* = 6.9 Hz, ArH), 7.24-7.27 (3H, m, ArH), 7.81 (2H, d, *J* = 8.2 Hz, ArH); ^{13}C NMR (150 MHz, $CDCl_3$) δ 9.9 (q), 21.4 (q), 30.0 (t), 47.7 (d), 84.5 (s), 86.9 (s), 122.2 (s), 127.5 (d×2), 128.0 (d×2), 128.3 (d), 129.5 (d×2), 131.5 (d×2), 137.4 (s), 143.5 (s); EIMS m/z 284 (M^+ -Et); high resolution mass calcd for $C_{18}H_{19}NO_2S$: 313.1136, found m/z 313.1137. Anal. Calcd for $C_{18}H_{19}NO_2S$ (+ 1 / 3 H_2O): C, 67.70; H, 6.21; N, 4.39. Found: C, 67.56; H, 6.01; N, 4.41.

Synthesis of 1-(3,4,5-trimethoxyphenyl)pent-1-yn-3-ol

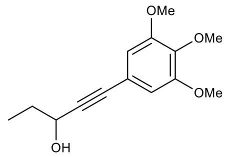

To a THF (12.0 mL) solution of 5-ethynyl-1,2,3-trimethoxybenzene (**21**)(0.700 g, 3.64 mmol) was added 2.6 M *n*-BuLi (2.69 mL, 3.30 mmol) at -78 °C under an Ar atmosphere. After 10 min stirring the mixture, a THF (3.00 mL) solution of propionaldehyde (0.190 g, 3.30 mmol) was added dropwise to the mixture. After 10 min stirring, the mixture was poured into water (100 mL). The organic layer was separated and the aqueous layer was extracted with AcOEt. The organic layer was washed with NH_4Claq (50.0 mL×2) and dried over $MgSO_4$. The solvent was removed under reduced pressure. The residue was purified by column chromatography on silica gel eluting with AcOEt-*n*-hexane (1:10) to give 1-(3,4,5-trimethoxyphenyl)pent-1-yn-3-ol (**22**)(0.65 g, 71%) as a colorless oil.

IR (KBr, cm^{-1}) ν 3436 (OH), 2967, 2939, 2876, 2839, 2229, 1710, 1578, 1505, 1464, 1411, 1341, 1237, 1129, 965, 834; ^{1}H NMR (600 MHz, $CDCl_3$) δ 1.08 (3H, t, *J* = 7.4 Hz, Me), 1.80-1.85 (2H, m, CH_2), 1.89 (1H, brs, OH), 3.85 (9H, s, OMe), 4.55 (2H, q, *J* = 6.3 Hz, CH), 6.67 (2H, s, ArH); ^{13}C NMR (150 MHz, $CDCl_3$) δ 9.5 (q), 30.9 (t), 56.1 (qx2), 60.9 (q), 64.1 (d), 84.8 (s), 89.0 (s), 108.8 (dx2), 117.6 (s), 138.8 (s), 152.9 (sx2); EIMS m/z 250 (M^+); Anal. Calcd for $C_{14}H_{18}O_4$(+1/4H_2O): C, 66.00; H, 7.32. Found: C, 66.17; H, 7.07.

(M. Kato, F. Kido, M.-D. Wu, A. Yoshikoshi *Bull. Chem. Soc. Jpn.* **1974**, *47*, 1516-1521.)

Synthesis of 1,2,3-trimethoxy-4-(1-(3,4,5-trimethoxyphenyl)pent-1-yn-3-yl)benzene (**23**).

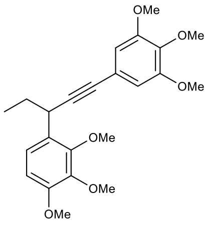

To a nitromethane (7.70 mL) solution of 1-(3,4,5-trimethoxyphenyl)pent-1-yn-3-ol (**22**)(400 mg, 1.60 mmol), 1,2,3-trimethoxybenzene (0.538 g, 3.20 mmol), tetrabutylammonium hexafluorophosphate (0.124 mg, 0.320 mmol), and 1,1'-binaphtol (90.3 mg, 0.320 mmol) were added silver perchlorate (0.166 g, 0.400 mmol) and indium trichloride (70.8 mg, 0.320 mmol) at room temperature. The reaction mixture was stirred for 2 h. The reaction mixture was filtered through a pad of celite with chloroform and then poured into a saturated sodium hydrogencarbonate. The organic layer was separated and the aqueous layer was extracted with AcOEt. The combined organic layer was dried over $MgSO_4$. The solvent was removed under reduced pressure. The residue was purified by preparative TLC on silica gel eluting with chloroform-*n*-hexane (1:10) to give 1,2,3-trimethoxy-4-(1-(3,4,5-trimethoxyphenyl)pent-1-yn-3-yl)benzene (**23**)(0.352 g, 55%) as a yellow oil.

IR (KBr, cm^{-1}) ν 2964, 2937, 2836, 2129, 1576, 1494, 1464, 1413, 1353, 1300, 1279, 1236, 1129, 1095, 1011, 832, 799; ^{1}H NMR (600 MHz, $CDCl_3$) δ 1.07 (3H, t, *J* = 7.4 Hz, Me), 1.77-1.84 (2H, m, CH_2), 3.84 (6H, s, OMe), 8.85 (3H, s, OMe), 3.86 (3H, s, OMe), 3.94 (3H, s, OMe), 4.10 (1H, dd, *J* = 4.5 and 6.3 Hz, CH), 6.67 (2H, s, ArH), 6.69 (1H, d, *J* = 8.6 Hz, ArH), 7.22 (1H, d, *J* = 8.6 Hz, ArH); ^{13}C NMR (150 MHz, $CDCl_3$) δ 12.0 (q), 30.5 (t), 33.1 (d), 55.9 (q), 56.0 (qx2), 60.6 (q), 60.8 (q), 61.0 (q), 82.1 (s), 91.2 (s), 107.1 (d), 108.7 (dx2), 119.0 (s), 122.6 (s), 127.9 (s), 138.2 (s), 141.9 (s), 150.8 (s), 152.4 (s), 152.9 (sx2); EIMS m/z 402 (M^++2). Anal. Calcd for $C_{23}H_{28}O_6$(+5/8H_2O): C, 67.10; H, 7.16. Found: C, 67.12; H, 7.10.

Hydrogenation of 1,2,3-trimethoxy-4-(1-(3,4,5-trimethoxyphenyl)pent-1-yn-3-yl)benzene (**24**).

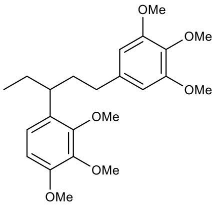

To a ethyl acetate (2.00 mL) solution of 1,2,3-trimethoxy-4-(1-(3,4,5-trimethoxyphenyl)pent-1-yn-3-yl)benzene (**23**) (0.100 g, 0.250 mmol) was added palladium-carbon (100 mg) and then stirred at room temperature under hydrogen (1.00 atm) for 12 h. The mixture was filtered through celite and washed with ethyl acetate. The combined organic layer was evaporated under reduced pressure. The residue was purified by preparative TLC on silica gel eluting with AcOEt-n-hexane (1:10) to give 1,2,3-trimethoxy-4-(1-(3,4,5-trimethoxyphenyl)pentan-3-yl)benzene (**24**)(83.0 mg, 82%) as a yellow oil.

IR (KBr, cm^{-1}) ν 2936, 1590, 1495, 1462, 1417, 1332, 1279, 1239, 1129, 1096, 1014, 827, 801; ^{1}H NMR (600 MHz, $CDCl_3$) δ 0.80 (3H, *J* = 7.5 Hz, Me), 1.52-1.59 (1H, m, CH), 1.67-1.72 (1H, m, CH), 1.78-1.95 (2H, m, CH_2), 2.37-2.48 (2H, m, CH_2), 2.91-2.97 (1H, m, CH), 3.81 (3H, s, OMe), 3.83 (6H, s, OMex2), 3.84 (3H, s, OMe), 3.86 (3H, s, OMe), 3.88 (3H, s, OMe), 6.34 (2H, s, ArH), 6.68 (1H, d, *J* = 9.2 Hz, ArH), 6.84 (1H, d, *J* = 8.6 Hz, ArH); ^{13}C NMR (150 MHz, $CDCl_3$) δ 12.2 (q), 29.2 (t), 34.5 (t), 37.9 (t), 39.2 (d), 55.9 (q), 56.0 (qx2), 60.7 (q), 60.8 (q), 60.9 (q), 105.2 (dx2), 107.5 (d), 121.4 (d), 131.2 (s), 135.9 (s), 138.7 (s), 142.1 (s), 151.5 (s), 152.4 (s), 152.9 (sx2); EIMS m/z 404 (M^+); high resolution mass calcd for $C_{23}H_{32}O_6$: 404.2199, found m/z 404.2217.

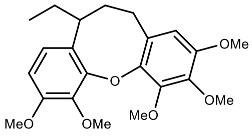

To a dichloromethane (1.90 mL) solution of 1,2,3-trimethoxy-4-(1-(3,4,5-trimethoxyphenyl)pentan-3-yl)benzene (**24**)(30.0 mg, 0.0742 mmol), Iodobenzene Diacetate (13.1 mg, 0.0408 mmol) were added Boron Trifluoride Diethyl Ether at -40ºC. The reaction mixture was stirred for 1.5 h. The reaction mixture was poured into saturated aqueous $NaHCO_3$ (50.0 mL). The organic layer was separated and the aqueous layer was extracted with chloroform. The combined organic layer was dried over $MgSO_4$. The solvent was removed under reduced pressure. The residue was purified by preparative TLC on silica gel eluting with AcOEt-*n*-hexane (1:5) to give 7-ethyl-1,2,3,8,9,10-hexamethoxy-6,7-dihydro-5*H*-dibenzo[*a*,*c*][7]annulene (**25**)(3.7 mg, 12%) as a yellow oil and 7-ethyl-1,2,3,10,11-pentamethoxy-6,7-dihydro-5*H*-dibenzo[*b*,*g*]oxocine (**26**)(17.3 mg, 60%) as a pale yellow oil.

IR (KBr, cm^{-1}) 2936, 2860, 1651, 1635, 1593, 1455, 1404, 1328, 1258, 1201, 1119, 1093, 1005, 950, 847; ^{1}H NMR (600 MHz, $CDCl_3$) δ 0.89 (3H, t, *J* = 6.9 Hz, CH_3), 0.90-0.97 (1H, m, CH), 1.41-1.56 (2H, m, CH_2), 1.89-1.95 (1H, m, CH), 2.02-2.07 (1H, m, CH), 2.82-2.96 (2H, m, CH_2), 3.64 (3H, s, OMe), 3.76 (3H, s, OMe), 3.79 (3H, s, OMe), 3.84 (3H, s, OMe), 3.98 (3H, s, OMe), 6.25 (1H, d, *J* = 9.6 Hz, ArH), 6.44 (1H, s, ArH), 6.53 (1H, d, *J* = 10.1 Hz, ArH); ^{13}C NMR (150 MHz, $CDCl_3$) δ 12.4 (q), 22.2 (t), 22.9 (t), 30.7 (t), 44.6 (d), 52.0 (s), 55.7 (q), 60.3 (qx2), 60.4 (q), 60.7 (q), 107.5 (d), 121.7 (s), 127.6 (d), 133.3 (s), 139.6 (s), 140.1 (s), 146.0 (d), 152.4 (s), 152.6 (s), 167.0 (s), 185.1 (s); EIMS m/z 388 (M^+); high resolution mass calcd for $C_{22}H_{28}O_6$: 388.1886, found m/z 388.1887.

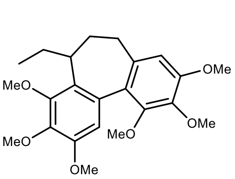

IR (KBr, cm^{-1}) 2959, 2933, 2858, 2853, 2834, 1596, 1487, 1458, 1402, 1315, 1252, 1127, 1112, 1093, 1018; ^{1}H NMR (600 MHz, $CDCl_3$) δ 0.60 (3H, t, *J* = 7.4 Hz, CH_3), 0.96-1.09 (2H, m, CH_2), 1.93 (1H, dt, *J* = 5.4 and 13.6 Hz, CH), 2.32-2.46 (3H, m, CH_3), 3.36-3.40 (1H, m, CH), 3.59 (3H, s, OMe), 3.85 (3H, s, OMe), 3.86 (3H, s, OMe), 3.90 (3H, s, OMe), 3.92 (3H, s, OMe), 3.95 (3H, s, OMe), 6.56 (1H, s, ArH), 6.89 (1H, s, ArH); ^{13}C NMR (150 MHz, $CDCl_3$) δ 13.2 (q), 28.6 (t), 32.2 (t), 34.8 (d), 38.6 (t), 55.9 (qx2), 60.5 (q), 60.8 (q), 61.2 (q), 61.4 (q), 107.2 (d), 111.1 (d), 126.7 (s), 128.9 (s), 130.8 (s), 136.6 (s), 140.8 (s), 141.0 (s), 150.5 (dx2), 151.5 (s), 152.2 (s); EIMS m/z 402 (M^+), 387 (M^+-Me), 373 (M^+-

Et); high resolution mass calcd for $C_{23}H_{30}O_6$: 402.2042, found m/z 402.2069.

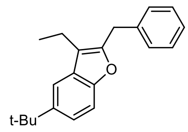

To a 1,4'-Dioxane (1.00 mL) solution of 4-(*tert*-butyl)-2-(1-phenylpent-1-yn-3-yl)phenol (**6o**) (30.0 mg, 0.103 mmol) were added t-BuOK (23.0 mg, 0.205 mmol) at room temperature. The reaction mixture was refluxed for 10 min. The reaction mixture was poured into water (50.0 mL). The organic layer was separated and the aqueous layer was extracted with AcOEt. The combined organic layer was dried over $MgSO_4$. The solvent was removed under reduced pressure. The residue was purified by preparative TLC on silica gel eluting with *n*-hexane to give 2-benzyl-5-(*tert*-butyl)-3-ethylbenzofuran(**27**)(25.6 mg, 85%) as a yellow oil.

IR (KBr, cm^{-1}) 2936, 2860, 1651, 1635, 1593, 1455, 1328, 1201, 1119; ^1H NMR (600 MHz, $CDCl_3$) δ 0.89 (3H, t, J = 6.9 Hz, CH_3), 0.90-0.97 (1H, m, CH), 1.41-1.56 (2H, m, CH_2), 1.89-1.95 (1H, m, CH), 2.02-2.07 (1H, m, CH), 2.82-2.96 (2H, m, CH_2), 3.64 (3H, s, OMe), 3.76 (3H, s, OMe), 3.79 (3H, s, OMe), 3.84 (3H, s, OMe), 3.98 (3H, s, OMe), 6.25 (1H, d, J = 9.6 Hz, ArH), 6.44 (1H, s, ArH), 6.53 (1H, d, J = 10.1 Hz, ArH); ^{13}C NMR (150 MHz, $CDCl_3$) δ 12.4 (q), 22.2 (t), 22.9 (t), 30.7 (t), 44.6 (d), 52.0 (s), 55.7 (q), 60.3 (qx2), 60.4 (q), 60.7 (q), 107.5 (d), 121.7 (s), 127.6 (d), 133.3 (s), 139.6 (s), 140.1 (s), 146.0 (d), 152.4 (s), 152.6 (s), 167.0 (s), 185.1 (s); EIMS m/z 388 (M^+); high resolution mass calcd for $C_{22}H_{28}O_6$: 388.1886, found m/z 388.1887.

Mechanistic study (Fig 4).

Preparations of *(R)*- and *(S)*-1-phenylpent-1-yn-3-ol (**1a**).

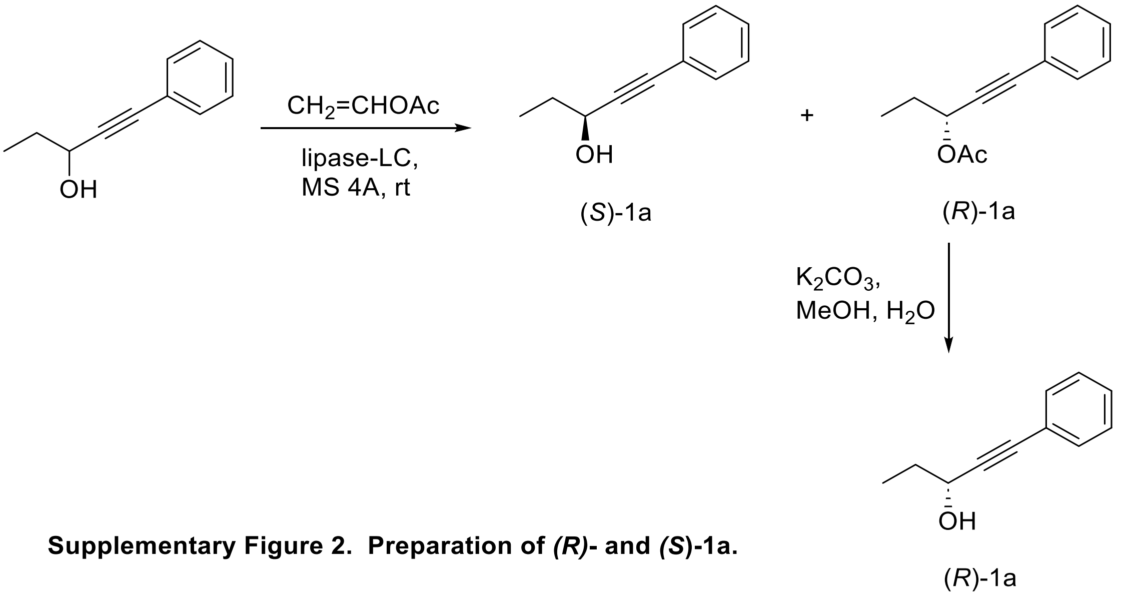

Supplementary Figure 2. Preparation of *(R)*- and *(S)*-1a.

To a pentane (25.0 mL) solution of **1a** (2.00 g, 12.5 mmol), vinyl acetate (5.38 g, 62.5 mmol), and MS 4A (0.44 g) was added lipase CL (0.13 g, Amano IM) at room temperature. The reaction mixture was stirred for 2 h and filtered through Celite, concentrated under reduced pressure and purified by column chromatography on silica gel (5-20% AcOEt in *n*-hexane) to yield *(S)*-**1a** (0.976 g, 48.8 %) as a colorless oil and *(R)*-1-phenylpent-1-yn-3-yl acetate (1.35 g, quant) as a colorless oil. The acetate was dissolved in methanol (20.0 mL)-water (7.0 mL). To the mixture was added potassium carbonate (1.46 g, 10.6 mmol). The whole was stirred for 12 h and poured into water (50 mL). The organic layer was separated and the aqueous layer was extracted with AcOEt. The combined organic layer was dried over $MgSO_4$. The solvent was removed under reduced pressure. The residue was purified by column chromatography on silica gel eluting with AcOEt-*n*-hexane (1:20) to give *(R)*-**1a** (0.902 g, quant) as a colorless oil.

HPLC analysis Chiralpak OD-3 (Hexane/IPA = 95:5, 1.0 mL/min, 254 nm, 15 °C) *(S)*-**1a**: 6.3 min; *(R)*-**1a**: 11.8 min. *(R)*-**1a** (98 % ee); *(S)*-**1a** (98% ee). The HPLC analysis data were shown in Supplementary Figure 3.

(Preparation: M. Garbacz, S. Stecko *Adv. Synth. Catal.* **2020**, *362*, 3213-3222. HPLC data: M. Lombardo, M. Chiarucci, C. Trombini *Chem. Eur. J.* **2008**, *14*, 11288-11291.)

Supplementary Figure 3. HPLC analysis data.

Data File C:\HPCHEM\1\DATA\Y-SAWADA\MYHA0011.D Sample Name: 10iPr/Hex

10%IPA/Hex MYH- 39+40
2+1 ul inject

Injection Date : 2021/10/23 10:19:35 dB'O
Sample Name : 10iPr/Hex Location : Vial 1
Acq. Operator : y-sawada
Acq. Instrument : Instrument 1
Method : C:\HPCHEM\1\METHODS\Y_SAWADA\DEF_LC1.M
Last changed : 2021/10/23 9:13:00 dB'O by y-sawada
(modified after loading)

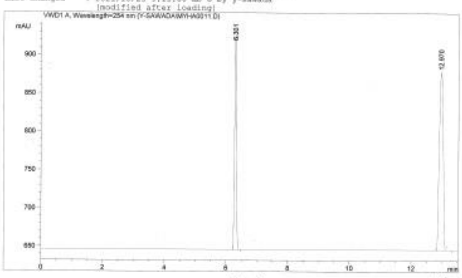

(S)-12 (R)-12

Area Percent Report

Sorted By : Signal
Multiplier : 1.0000
Dilution : 1.0000
Use Multiplier & Dilution Factor with ISTDs

(39) 2ul + (40) 1ul 2mix

Signal 1: VWD1 A, Wavelength=254 nm

Peak #	RetTime [min]	Type	Width [min]	Area mAU *s	Height [mAU]	Area %
1	6.301	BB	0.0758	1265.73486	281.05402	37.8574
2	12.970	BB	0.1562	2077.69434	233.56126	62.1426

Totals : 3343.42920 514.61528

Results obtained with enhanced integrator!

*** End of Report ***

Instrument 1 2021/10/23 10:33:16 dB'O y-sawada Page 1 of 1

Data File C:\HPCHEM\1\DATA\Y-SAWADA\MYHA0008.D Sample Name: 10iPr/Hex

10%IPA/Hex MYH-40
2ul inject

Injection Date : 2021/10/23 9:25:41 dB'O
Sample Name : 10iPr/Hex Location : Vial 1
Acq. Operator : y-sawada
Acq. Instrument : Instrument 1
Method : C:\HPCHEM\1\METHODS\Y_SAWADA\DEF_LC1.M
Last changed : 2021/10/23 9:13:00 dB'O by y-sawada
(modified after loading)

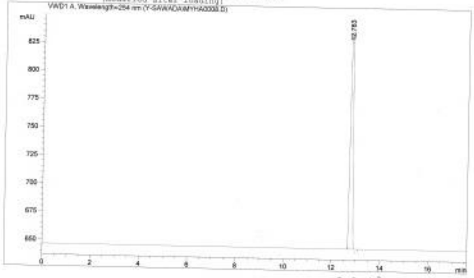

(R)-12

Area Percent Report

MYH-40

Sorted By : Signal
Multiplier : 1.0000
Dilution : 1.0000
Use Multiplier & Dilution Factor with ISTDs

Signal 1: VWD1 A, Wavelength=254 nm

Peak #	RetTime [min]	Type	Width [min]	Area mAU *s	Height [mAU]	Area %
1	12.783	BP	0.1409	1567.19385	193.77596	100.0000

Totals : 1567.19385 193.77596

Results obtained with enhanced integrator!

*** End of Report ***

Instrument 1 2021/10/23 9:43:25 dB'O y-sawada Page 1 of 1

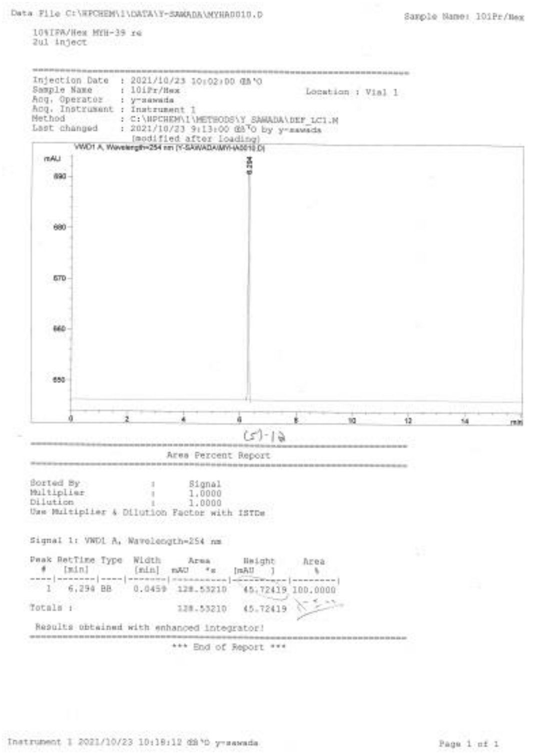

Data File C:\HPCHEM\1\DATA\Y-SAWADA\MYHA0010.D Sample Name: 10iPr/Hex

10%IPA/Hex MYH-39 re
2ul inject

Injection Date : 2021/10/23 10:02:00 [illegible]
Sample Name : 10iPr/Hex Location : Vial 1
Acq. Operator : y-sawada
Acq. Instrument : Instrument 1
Method : C:\HPCHEM\1\METHODS\Y_SAWADA\DEF_LC1.M
Last changed : 2021/10/23 9:13:00 [illegible] by y-sawada
(modified after loading)

Area Percent Report

Sorted By : Signal
Multiplier : 1.0000
Dilution : 1.0000
Use Multiplier & Dilution Factor with ISTDs

Signal 1: VWD1 A, Wavelength=254 nm

Peak #	RetTime [min]	Type	Width [min]	Area mAU *s	Height [mAU]	Area %
1	6.294	BB	0.0459	128.53210	45.72419	100.0000
Totals :				128.53210	45.72419	

Results obtained with enhanced integrator!

*** End of Report ***

Instrument 1 2021/10/23 10:18:12 [illegible] y-sawada Page 1 of 1

Attempts to the isolation of the indium catalyst (Experimental procedures for Fig 5).

To a nitromethane (2.00 mL) solution of indium chloride (22.0 mg, 0.100 mmol) was added silver perchlorate (56.2 mg, 0.200 mmol) at room temperature. The reaction mixture was stirred for 0.5 h and white powders of silver chloride formed in the flask. The nitromethane solution was decanted and evaporated under reduced pressure. To the residue was added n-hexane and the formed white powders were filtered off. The compound was highly moisture sensitive. The ^1H NMR chart for monitoring experiments by ^1H NMR was exhibited in Supplementary Figure 4.

^1H NMR (600 MHz, CD_3NO_2) δ 5.93 (brs).

Supplementary Figure 4. Monitoring experiments by ^1H NMR.

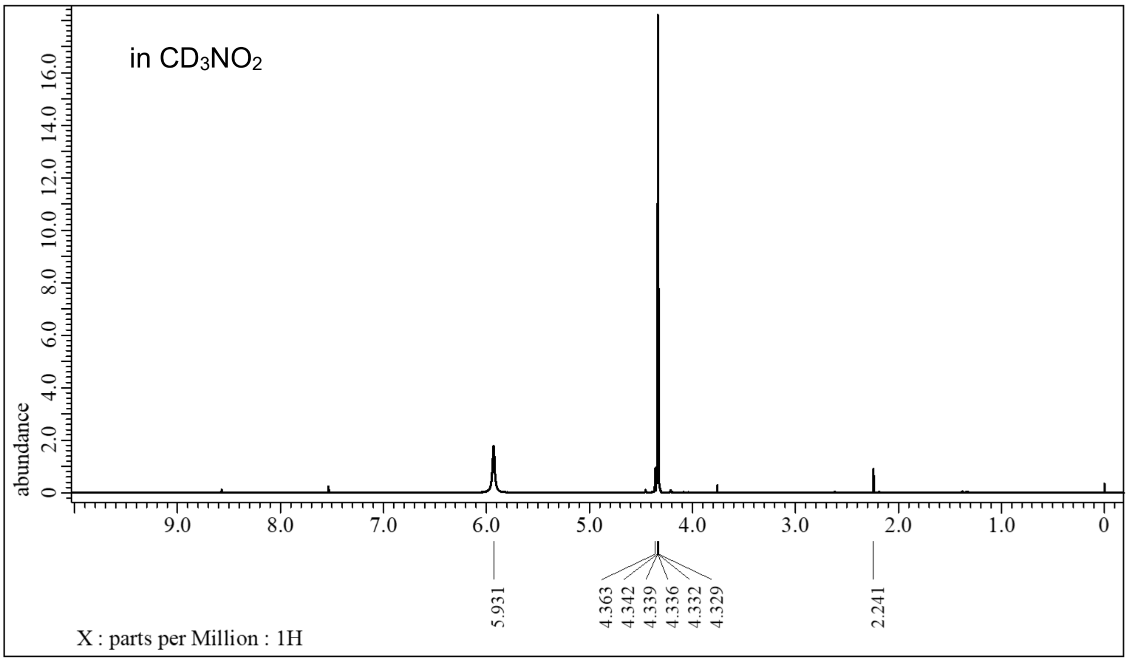

in CD_3Cl_3-CD_3OD

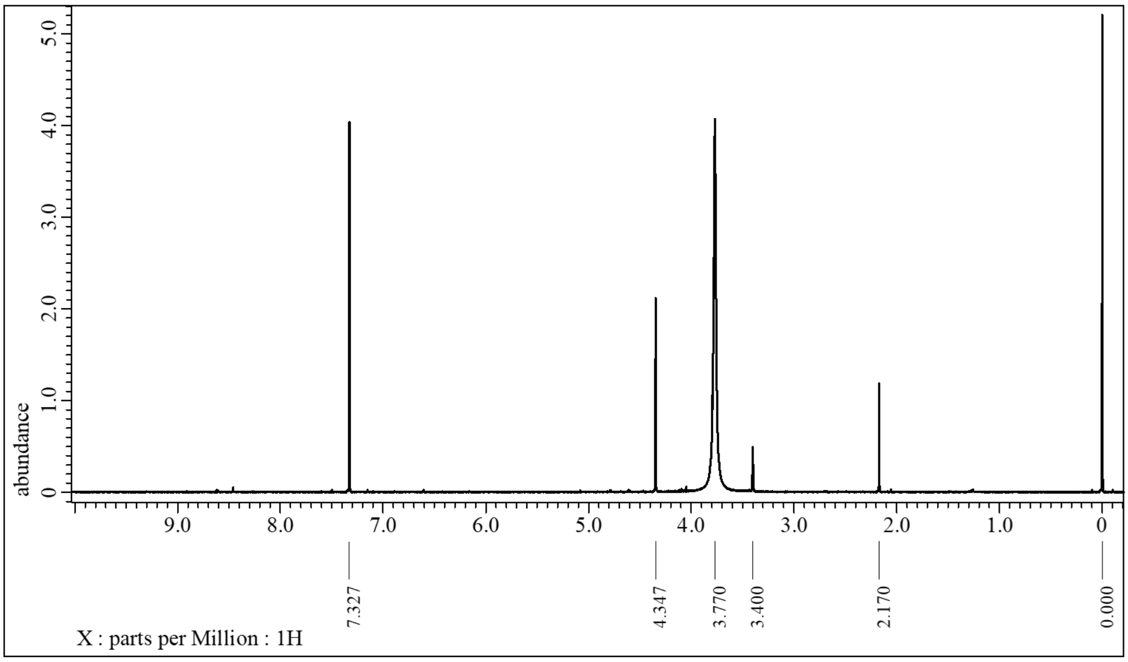

To a nitromethane (7.00 mL) solution of indium chloride (88.0 mg, 0.400 mmol) and 1,1'-binaphthol (0.225 g, 0.400 mmol) was added silver perchlorate (0.116 g, 0.800 mmol) at room temperature. The reaction mixture was stirred for 0.5 h. The nitromethane solution was decanted and evaporated under reduced pressure. The formed white powders were filtered off and washed with n-hexane. The compound was highly moisture sensitive.

^{1}H NMR (600 MHz, CD_3NO_2) δ 4.36 (s, Me), 6.51 (brs), 7.11 (d, J=7.3 Hz), 7.38-7.41 (m, ArH), 7.98 (d, J=8.3 Hz), 8.03 (d, J=8.9 Hz).

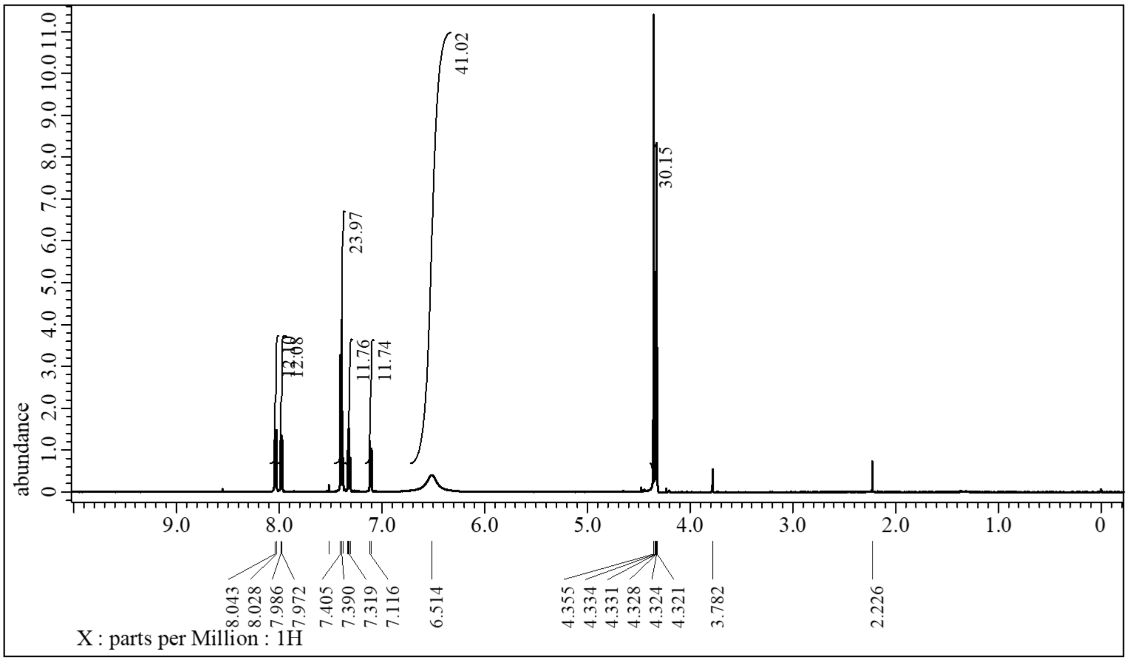

Experiments of a and b in Fig 4.

The reactions of *(S)*- and *(R)*-**1a** with 1,2,3-trimethoxybenzene were performed as shown in Supplementary Figure 5. Time course experiments by ^{19}F NMR were performed by the optimized reaction condition (Supplement Figure 6). The NMR charts of monitoring experiments of **3a** with 1,2,3-trimethoxybenzene were exhibited in Supplementary Fig 7. The molar ratio of **1o**, **2o**, and **3o** were exhibited in the graph (Supplementary Figure 8-10).

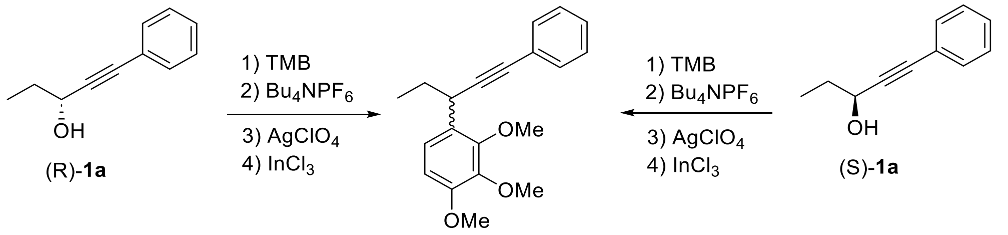

Supplementary Figure 5. Reactions of *(R)*- and *(S)*-1a with 1,2,3-trimethoxybenzene.

To a nitromethane (1.00 mL) solution of (R)-1-phenylpent-1-yn-3-ol (50.0 mg, 0.312 mmol) and 1,2,3-trimethoxybenzene (157 mg, 0.936 mmol), and tetrabutylammonium hexafluorophosphate (24.2 mg, 0.0624 mmol) were added silver perchlorate (32.3 mg, 0.156 mmol) and indium trichloride (13.8 mg, 0.0624 mmol). The reaction mixture was stirred at room temperature for 1.5 h and then the almost same procedure as described above. The residue was purified by preparative TLC on silica gel eluting with AcOEt-*n*-hexane (1:40) to give (R)- and (S)-1,2,3-trimethoxy-4-(1-phenylpent-1-yn-3-yl)benzene (65.0 mg, 67%) as a yellow oil, 1,2,3-trimethoxy-5-(1-phenylpent-1-yn-3-yl)benzene (7.90 mg, 8%) as a yellow oil, and (oxybis(pent-1-yne-3,1-diyl))benzene (15.9 mg, 17%) as a yellow oil. HPLC analysis Chiralpak AD-3 (hexane/IPA = 95:5, 1.0 mL/min, 254 nm, 15 °C) 4.52 min (48%), 4.77 min (51%).

To a nitromethane (1.00 mL) solution of (S)-1-phenylpent-1-yn-3-ol (50.0 mg, 0.312 mmol) and 1,2,3-trimethoxybenzene (157 mg, 0.936 mmol), and tetrabutylammonium hexafluorophosphate (24.2 mg, 0.0624 mmol) were added silver perchlorate (32.3 mg, 0.156 mmol) and indium trichloride (13.8 mg, 0.0624 mmol). The reaction mixture was stirred at room temperature for 1.5 h and then the almost same procedure as described above. The residue was purified by preparative TLC on silica gel eluting with AcOEt-*n*-hexane (1:40) to give (R)- and (S)-1,2,3-trimethoxy-4-(1-phenylpent-1-yn-3-yl)benzene (65.9 mg, 68%) as a yellow oil, 1,2,3-trimethoxy-5-(1-phenylpent-1-yn-3-yl)benzene (10.9 mg, 11%) as a yellow oil, and (oxybis(pent-1-yne-3,1-diyl))benzene (0.94 mg, 1%) as a yellow oil. HPLC analysis Chiralpak AD-3 (hexane/IPA = 95:5, 1.0 mL/min, 254 nm, 15 °C) 4.53 min (51%), 4.77 min (48%).

Supplementary Figure 6. Experiments of Fig 4 e.

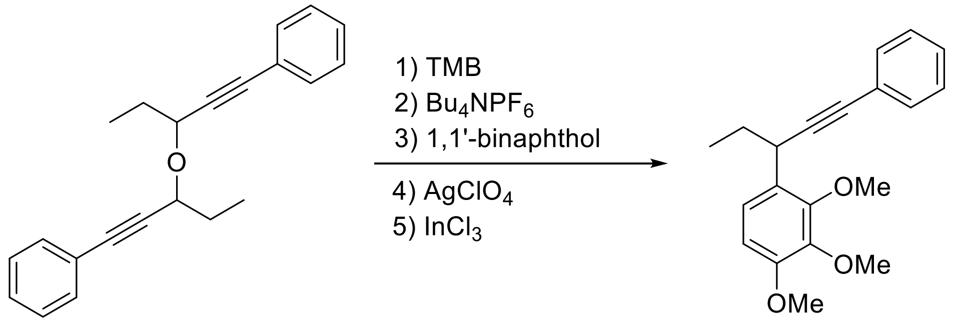

To a nitromethane (1.00 mL) solution of oxybis(pent-1-yn-3,1-diyl))dibenzene (30.0 mg, 0.100 mmol), 1,2,3-trimethoxybenzene (50.0 mg, 0.298 mmol), tetrabutylammonium hexafluorophosphate (7.70 mg, 2.00x10-5 mol), 1,1'-binaphthol (5.70 mg, 2.00x10-5) were added silver perchlorate (11.2 mg, 4.00x10-5) and indium chloride (4.40 mg, 2.00x10-5 mol) at room temperature. The reaction mixture was stirred for 1 h and then poured into water (50 mL). The organic layer was separated and the aqueous layer was extracted with AcOEt. The combined organic layer was dried over $MgSO_4$. The solvent was removed under reduced pressure. The residue was purified by preparative TLC on silica gel eluting with AcOEt-*n*-hexane to give **2a** (29.2 mg, 47%). Crystallization of **2a** gave the optically pure product as white powders. HPLC analysis Chiralpak AD-3 (hexane/IPA = 95:5, 1.0 mL/min, 254 nm, 15 °C) 4.19 min (100%).

Data File C:\HPCHEM\1\DATA\YOSHIMAE\21062301.D Sample Name: bifido adapt

```
Injection Date   : 06/23/2021 10:32:04 AM
Sample Name      : bifido adapt                    Location : Vial 1
Acq. Operator    : yoshimae
Acq. Instrument  : Instrument 1
Method           : C:\HPCHEM\1\METHODS\Y_SANADA\DEF_LC1.M
Last changed     : 06/23/2021 10:10:51 AM by yoshimae
                   (modified after loading)
```

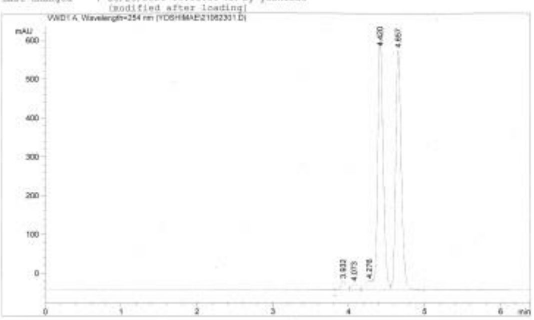

Area Percent Report

```
Sorted By           :      Signal
Multiplier          :      1.0000
Dilution            :      1.0000
Sample Amount       :      5.00000  [ng/ul]   (not used in calc.)
Use Multiplier & Dilution Factor with ISTDs
```

(rac)-2a

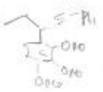

Signal 1: VWD1 A, Wavelength=254 nm

Peak #	RetTime [min]	Type	Width [min]	Area mAU *s	Height [mAU]	Area %
1	3.932	VV	0.0703	112.79228	24.22104	1.6757
2	4.073	VV	0.0778	93.75154	18.11797	1.3928
3	4.276	VV	0.0877	140.92381	24.40613	2.0936
4	4.420	VV	0.0750	3194.84253	647.80353	47.4629
5	4.657	VV	0.0791	3188.92676	618.45752	47.3750
Totals :				6731.23692	1333.00619	

MYH-1

Results obtained with enhanced integrator!

*** End of Report ***

Instrument 1 06/23/2021 10:38:37 AM yoshimae Page 1 of 1

Data File C:\HPCHEM\1\DATA\YAYOTA\20031254.D Sample Name: bifido adapt

Injection Date : 06/18/2021 7:17:46 PM
Sample Name : bifido adapt Location : Vial 1
Acq. Operator : y_sawada
Acq. Instrument : Instrument 1
Method : C:\HPCHEM\1\METHODS\Y_SAWADA\DEF_LC1.M
Last changed : 06/18/2021 6:57:05 PM by y_sawada
(modified after loading)

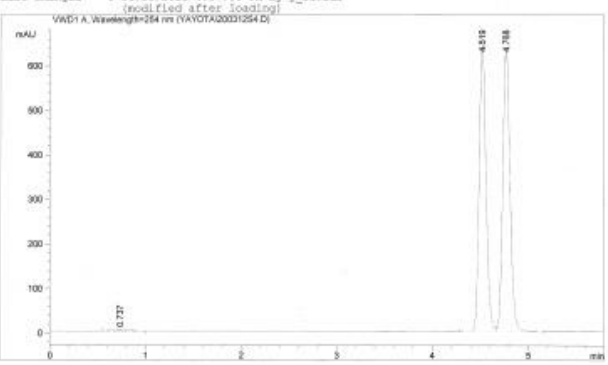

Area Percent Report

Sorted By : Signal
Multiplier : 1.0000
Dilution : 1.0000
Sample Amount : 5.00000 [ng/ul] (not used in calc.)
Use Multiplier & Dilution Factor with ISTDs

5% IPA / 15°C
2a from (R)-1

Signal 1: VWD1 A, Wavelength=254 nm

Peak #	RetTime [min]	Type	Width [min]	Area mAU *s	Height [mAU]	Area %
1	0.737	BP	0.1568	30.02207	2.98059	0.4628
2	4.519	VV	0.0761	3162.76465	645.12988	48.7549
3	4.768	VB	0.0793	3294.28076	636.38287	50.7823

Totals : 6487.06748 1284.49334

Results obtained with enhanced integrator!

*** End of Report ***

Instrument 1 06/18/2021 7:23:40 PM y_sawada Page 1 of 1

Data File C:\HPCHEM\1\DATA\YAYOTA\20031285.D Sample Name: bifido adapt

```
=====================================================================
Injection Date   : 06/18/2021 7:24:45 PM
Sample Name      : bifido adapt                 Location : Vial 1
Acq. Operator    : y_sawada
Acq. Instrument  : Instrument 1
Method           : C:\HPCHEM\1\METHODS\Y_SAWADA\DEF_LC1.M
Last changed     : 06/18/2021 6:57:05 PM by y_sawada
                   (modified after loading)
```

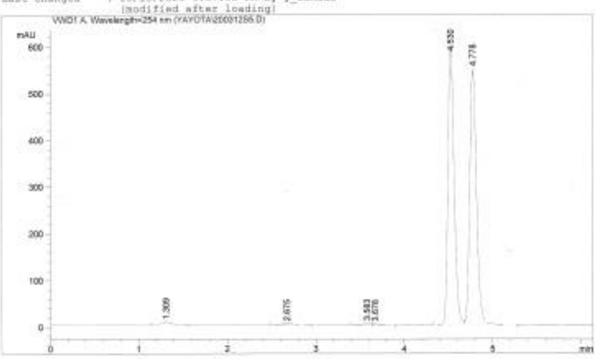


```
=====================================================================
                         Area Percent Report
=====================================================================

Sorted By             :      Signal
Multiplier            :      1.0000
Dilution              :      1.0000
Sample Amount         :      5.00000  [ng/ul]   (not used in calc.)
Use Multiplier & Dilution Factor with ISTDs
```

5% IPA/15°C

2a from (S)-1a

Signal 1: VWD1 A, Wavelength=254 nm

Peak #	RetTime [min]	Type	Width [min]	Area mAU *s	Height [mAU]	Area %
1	1.309	BB	0.1282	51.35530	6.23152	0.8712
2	2.675	BB	0.1585	25.90923	2.53665	0.4396
3	3.583	PV	0.0788	17.35536	3.38424	0.2944
4	3.676	VP	0.0709	8.61072	1.73840	0.1461
5	4.530	VV	0.0747	2957.72534	603.35583	50.1779
6	4.778	VB	0.0792	2833.51709	548.38348	48.0707
Totals :				5894.47303	1165.63013	

Results obtained with enhanced integrator!

```
=====================================================================
```

*** End of Report ***

Instrument 1 06/18/2021 7:31:00 PM y_sawada

Page 1 of 1

Procedures for the time course experiments.

The ^{19}F NMR spectral data of the selected alcohol and some products were measured and confirmed in nitromethane-d3 as the reference of trifluoroacetic acid (δ_F=-76.0 ppm).

1-(4-fluorophenyl)oct-1-yn-3-ol (**1o**): ^{19}F NMR (CD_3NO_2): 110.3 ppm; ^{19}F NMR ($CDCl_3$): -109.1 ppm

Tetrabutylammonium hexafluorophosphate: ^{19}F NMR (CD_3NO_2): -70.6 ppm; ^{19}F NMR ($CDCl_3$): -110.3 ppm.

1-(1-(4-fluorophenyl)oct-1-yn-3-yl)-2,3,4-trimethoxybenzene (**2o**): ^{19}F NMR (CD_3NO_2): -111.2 ppm; ^{19}F NMR ($CDCl_3$): -112.2 ppm.

5-(1-(4-fluorophenyl)oct-1-yn-3-yl)-1,2,3-trimethoxybenzene (**2o**): ^{19}F NMR (CD_3NO_2): -111.0 ppm.

4,4'-(oxybis(oct-1-yne-3,1-diyl))bis(p-fluorobenzene) (**3o**): ^{19}F NMR (CD_3NO_2): -111.0 ppm; ^{19}F NMR ($CDCl_3$): -108.9 ppm.

Supplementary Figure 7. NMR Chart of monitoring experiments by the ^{19}F NMR.

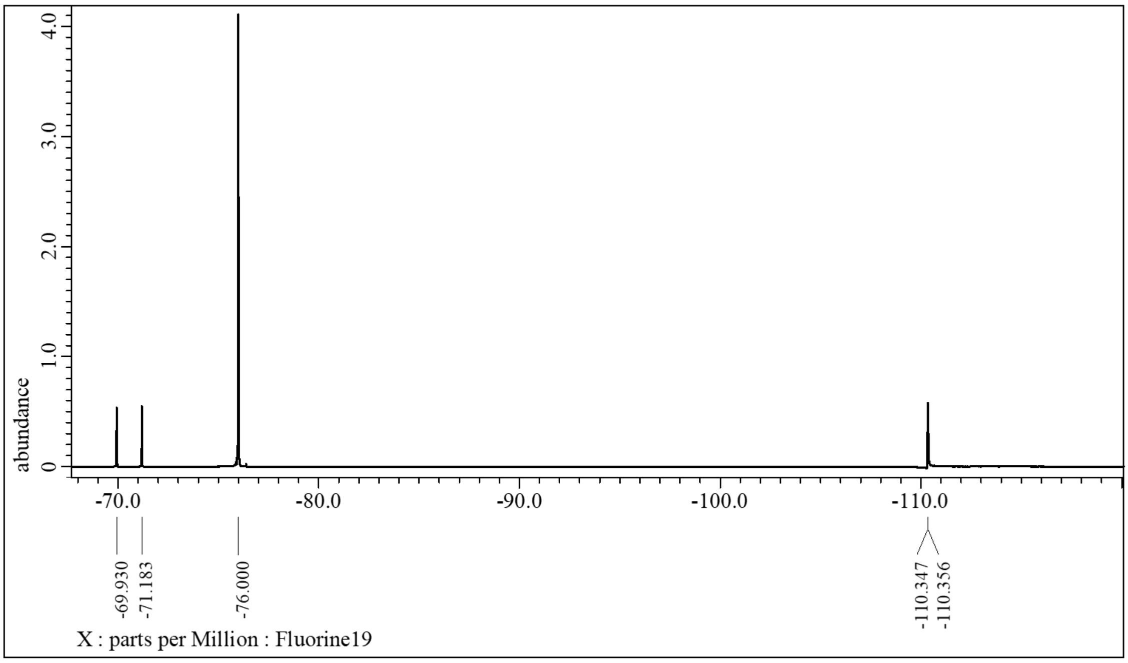

Step I: To a nitromethane-d3 (0.75 mL) solution of **1o** (30.0 mg, 0.136 mmol), 1,2,3-trimethoxybenzene (68.7 mg, 0.409 mmol), tetrabutylammonium hexafluorophosphate (10.6 mg, 2.72×10^{-2} mmol) was added into a NMR tube at room temperature. The mixture was stirred until a homogeneous solution was formed.

Step II: To the NMR tube were added indium chloride (4.52 mg, 2.04×10^{-2} mmol), and silver perchlorate (8.47 mg, 4.09×10^{-2} mmol). The mixture was stirred until the precipitates of silver chloride were formed for 10 seconds. This time is set as time 0.

Step III: ^{19}F NMR is recorded automatically at an internal of 5 min.

Step IV: Step I-III was repeated three times.

Optimization of the concentration of catalyst for the time course experiments.

1) 0.15 equiv of $InCl_3/AgClO_4$.

time [sec]		0	356	598	898	1197	1498	1799
concentration [mol/L]	**1o**	1.81.E-01	4.86.E-02	2.67.E-02	1.79.E-02	1.18.E-02	8.49.E-03	6.06.E-03
	**2o**	0.00.E+00	8.04.E-02	9.73.E-02	1.15.E-01	1.19.E-01	1.37.E-01	1.42.E-01
	**3o**	0.00.E+00	1.52.E-03	1.60.E-03	1.75.E-03	1.92.E-03	1.91.E-03	1.83.E-03

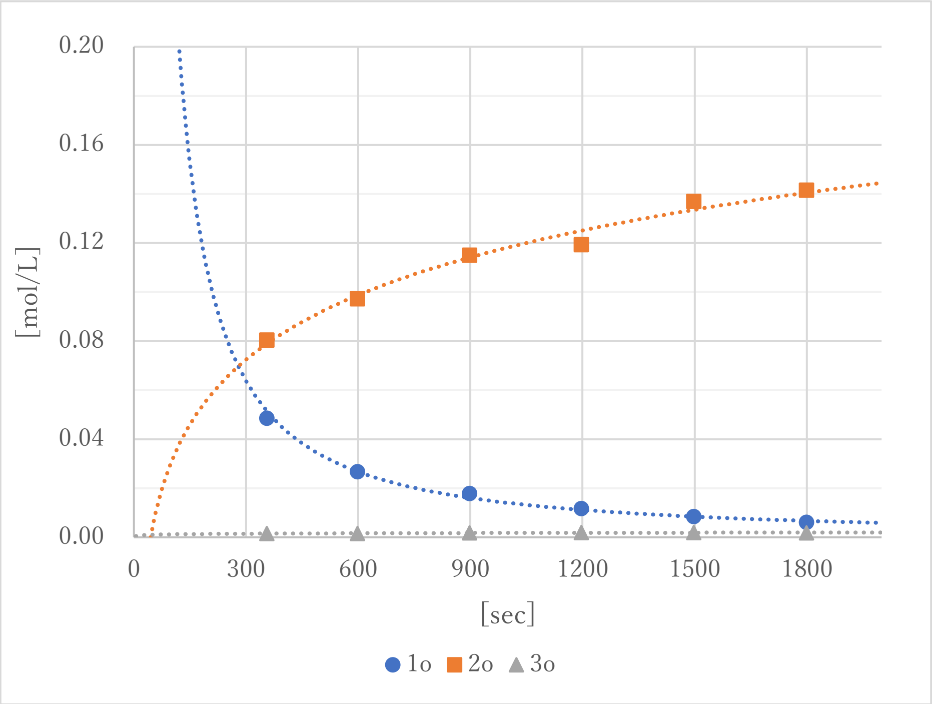

Supplementary Figure 8. Monitoring experiments of compounds 1o, 2o, and 3o by the ^{19}F NMR.

2) 0.10 equiv of $InCl_3/AgClO_4$.

time [sec]		0	350	573	893	1200	1499
concentration [mol/L]	1o	0.1813	0.1767	0.1645	0.1499	0.1465	0.1319
	2o	0	2.66.E-03	9.59.E-03	1.86.E-02	2.23.E-02	3.14.E-02

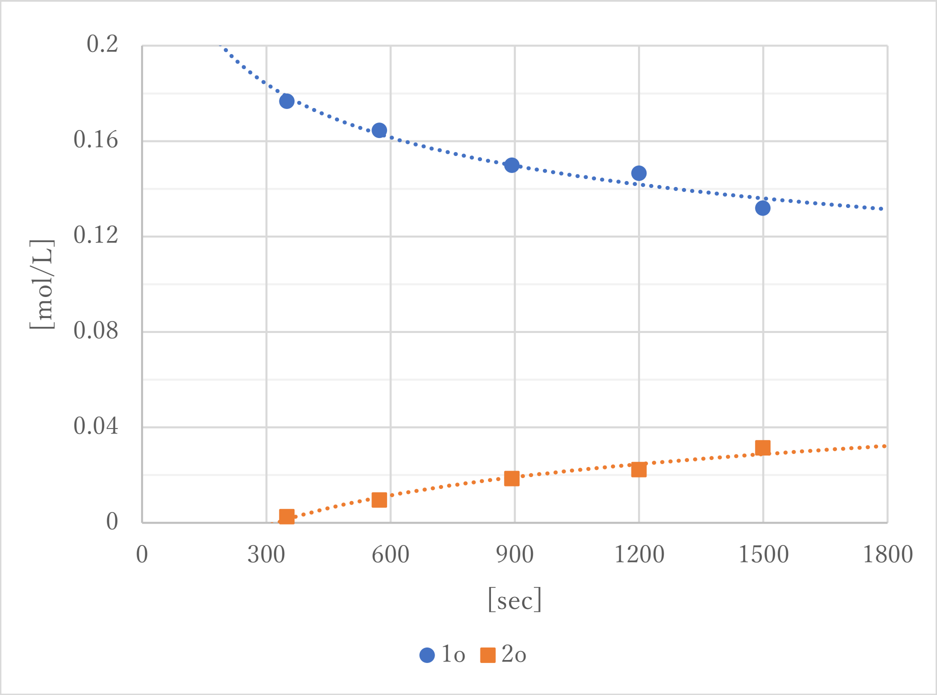

Supplementary Figure 9. Monitoring experiments of both 1o and 2o by the ^{19}F NMR.

3) 0.12 equiv of $InCl_3/AgClO_4$.

first	time [sec]		0	280	600	899	1200	1500	1799	2101	2400	2702	3001	3302	3599
	concentration [mol/L]	1o	1.81.E-01	8.06.E-02	4.70.E-02	3.29.E-02		1.95.E-02	1.61.E-02	1.34.E-02	1.21.E-02	9.40.E-03	9.07.E-03	7.72.E-03	6.92.E-03
		2o	0.00.E+00	9.74.E-02	1.12.E-01	1.26.E-01		1.37.E-01	1.39.E-01	1.50.E-01	1.46.E-01	1.51.E-01	1.52.E-01	1.52.E-01	1.60.E-01
		3o	0.00.E+00	0.00.E+00	2.40.E-03	3.20.E-03		3.10.E-03	2.90.E-03	3.20.E-03	2.90.E-03	2.80.E-03	2.80.E-03	2.70.E-03	2.70.E-03

second	time [sec]		0	280	602	899	1201	1500	1800	2100	2398	2697	3000	3302	3600
	concentration [mol/L]	1o	1.81.E-01	7.91.E-02	4.29.E-02	3.30.E-02	2.52.E-02	2.15.E-02	2.02.E-02	1.49.E-02	1.23.E-02	1.15.E-02	1.01.E-02	8.70.E-03	8.01.E-03
		2o	0.00.E+00	6.79.E-02	7.85.E-02	8.97.E-02	9.66.E-02	9.66.E-02	1.09.E-01	1.06.E-01	1.01.E-01	1.11.E-01	1.07.E-01	1.26.E-01	1.22.E-01
		3o	0.00.E+00	2.90.E-03	2.98.E-03	3.40.E-03	3.36.E-03	3.17.E-03	3.89.E-03	3.20.E-03	3.06.E-03	3.21.E-03	3.17.E-03	3.49.E-03	3.33.E-03

thrird	time [sec]		0	342	598	898	1198	1500	1799	2099	2399	2700	2999	3299	3599
	concentration [mol/L]	1o	1.81.E-01	8.40.E-02	4.86.E-02	3.61.E-02	2.99.E-02	2.27.E-02	1.67.E-02	1.54.E-02	1.15.E-02	1.14.E-02	8.40.E-03	6.94.E-03	8.14.E-03
		2o	0.00.E+00	7.46.E-02	8.96.E-02	1.10.E-01	1.15.E-01	1.40.E-01	1.29.E-01	1.30.E-01	1.30.E-01	1.53.E-01	1.21.E-01	1.25.E-01	1.53.E-01
		3o	0.00.E+00	3.36.E-03	3.49.E-03	3.97.E-03	4.00.E-03	4.37.E-03	3.91.E-03	3.99.E-03	3.79.E-03	4.21.E-03	3.54.E-03	3.52.E-03	3.68.E-03

avarage	time [sec]		0	300	600	900	1200	1500	1800	2100	2400	2700	3000	3300	3600
	concentration [mol/L]	**1o**	1.81.E-01	8.12.E-02	4.62.E-02	3.40.E-02	2.75.E-02	2.13.E-02	1.77.E-02	1.46.E-02	1.20.E-02	1.08.E-02	9.17.E-03	7.79.E-03	7.69.E-03
		**2o**	0.00.E+00	8.00.E-02	9.34.E-02	1.09.E-01	1.06.E-01	1.25.E-01	1.26.E-01	1.29.E-01	1.25.E-01	1.39.E-01	1.27.E-01	1.34.E-01	1.45.E-01
		**3o**	0.00.E+00	2.09.E-03	2.96.E-03	3.52.E-03	3.68.E-03	3.55.E-03	3.57.E-03	3.46.E-03	3.25.E-03	3.41.E-03	3.17.E-03	3.24.E-03	3.24.E-03

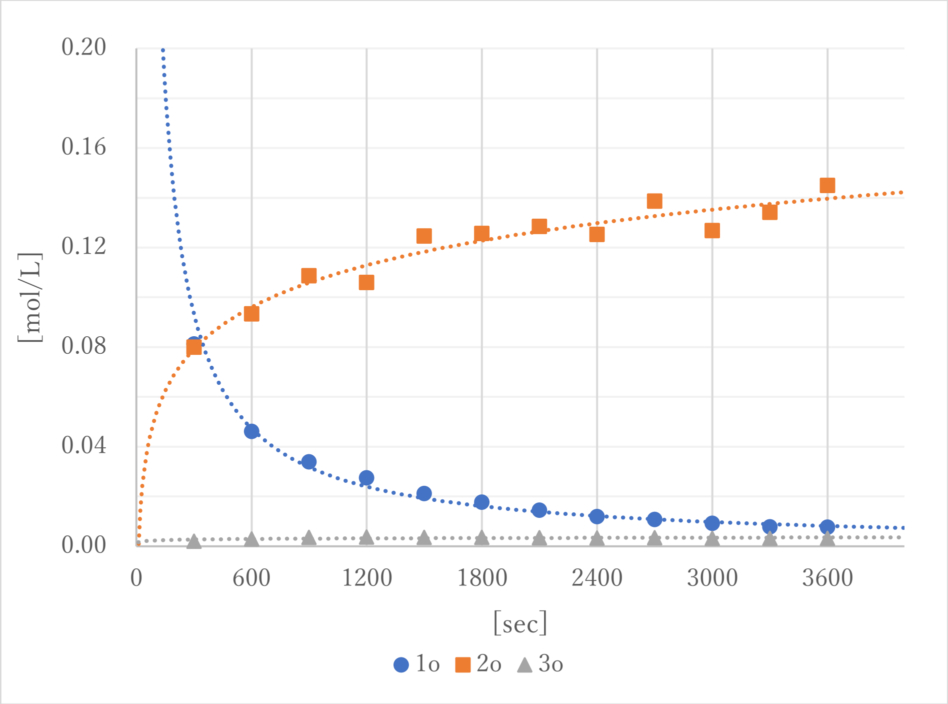

Supplementary Figure 10. Monitoring experiments (avarage) by the ^{19}F NMR.
