# Supplementary material for: Cationic indium catalysis as a powerful tool for generating α-alkyl propargyl cations for SN1 reactions
Source: Commun Chem. 2023 Dec 16;6:279. doi: 10.1038/s42004-023-01048-4 (PMC10725475; doi:10.1038/s42004-023-01048-4)
Supplement: Supplementary file 3 — Description of Additional Supplementary Files [file 42004_2023_1048_MOESM3_ESM.pdf]

Description of Additional Supplementary Files

File name: Supplementary Data 1

Description: NMR data
