# Supplementary figures and images for: Cationic indium catalysis as a powerful tool for generating α-alkyl propargyl cations for SN1 reactions
Source: Commun Chem. 2023 Dec 16;6:279. doi: 10.1038/s42004-023-01048-4 (PMC10725475; doi:10.1038/s42004-023-01048-4)

$^1\text{H}$  and  $^{13}\text{C}$  NMR Charts.

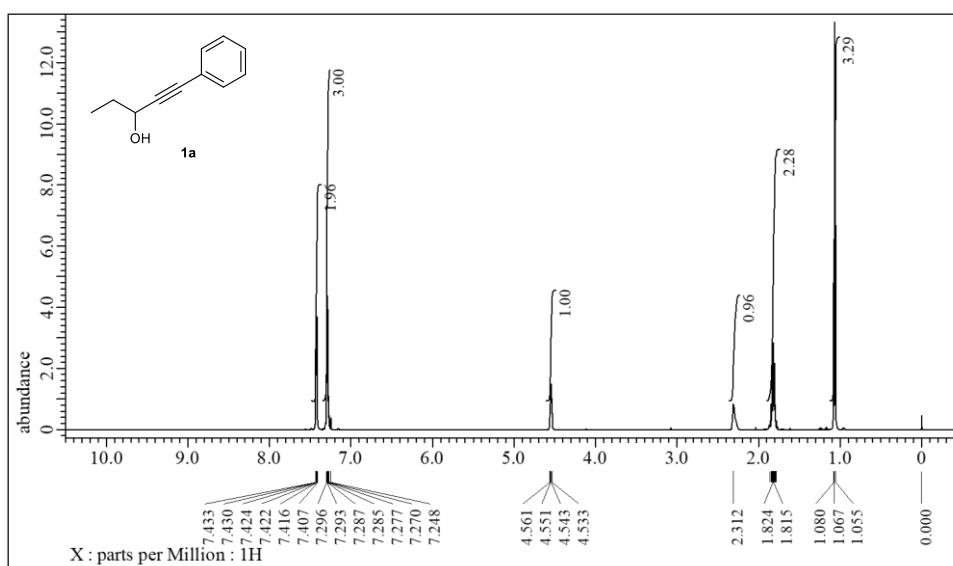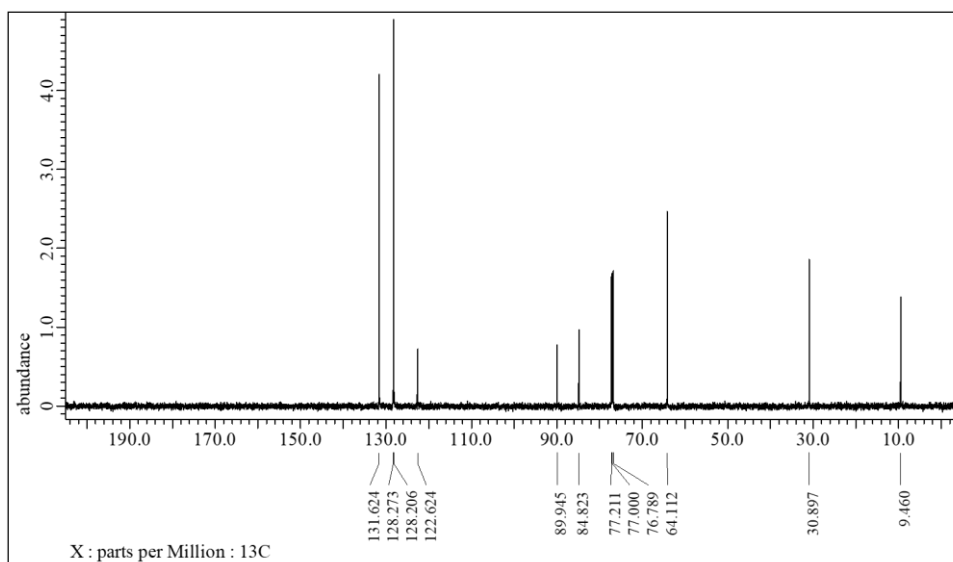

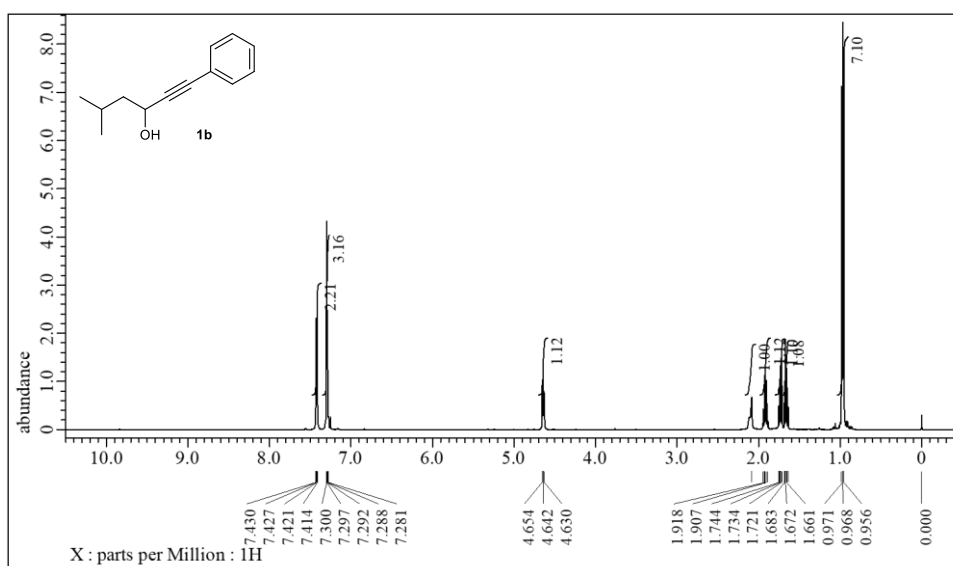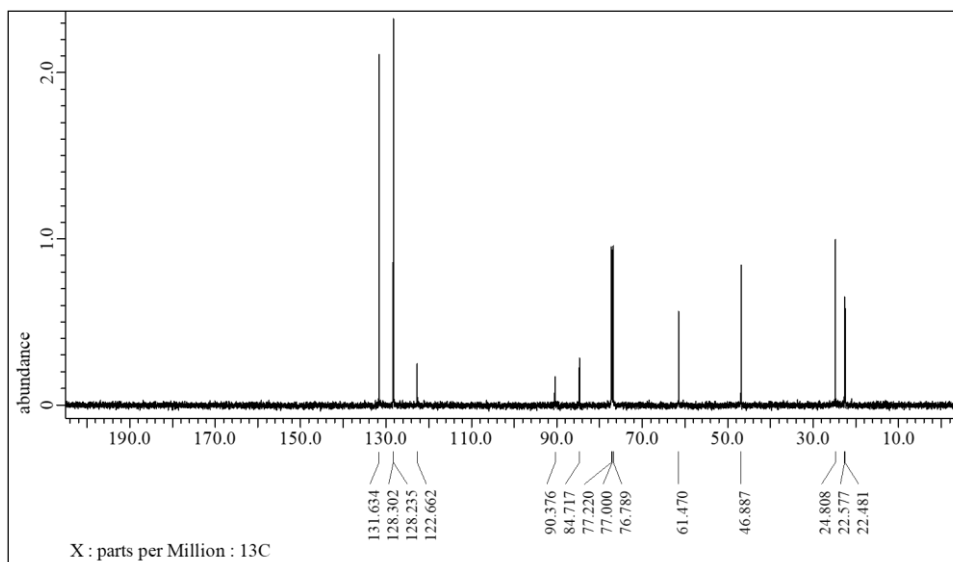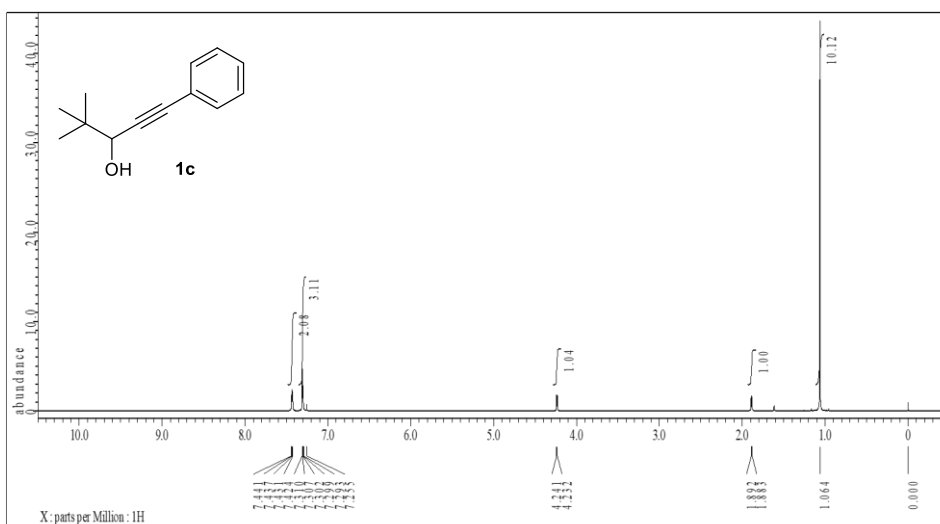

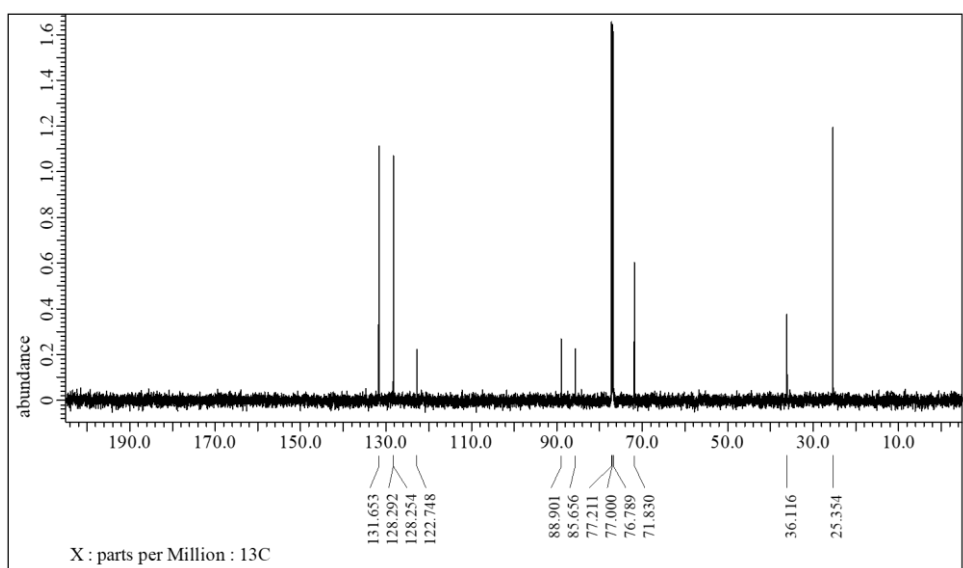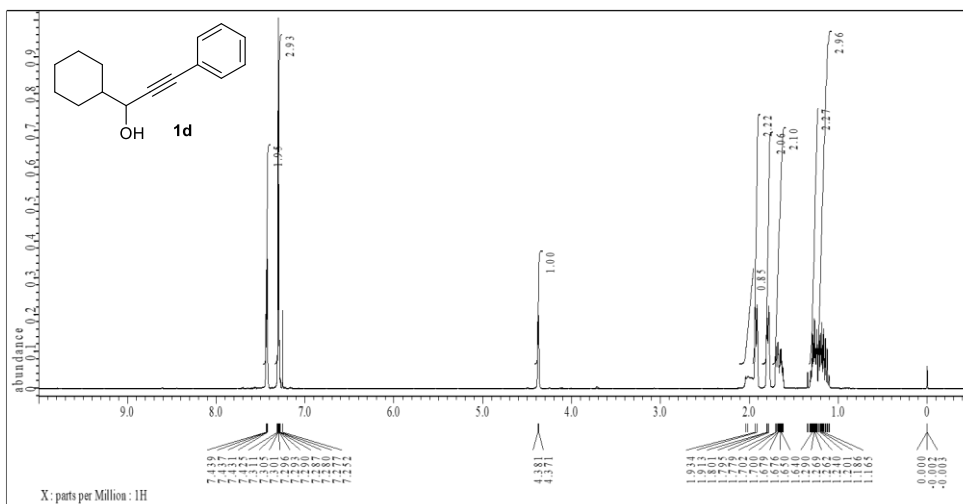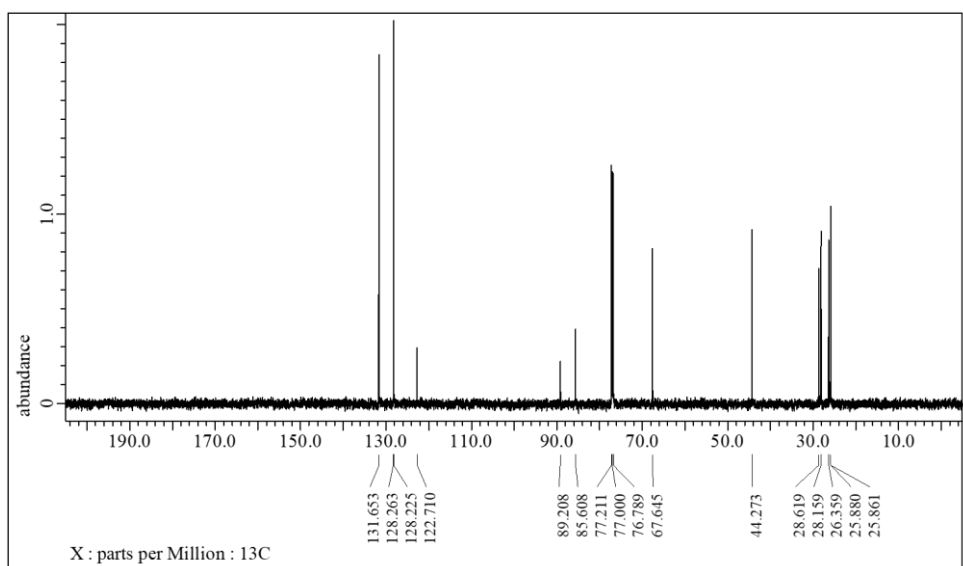

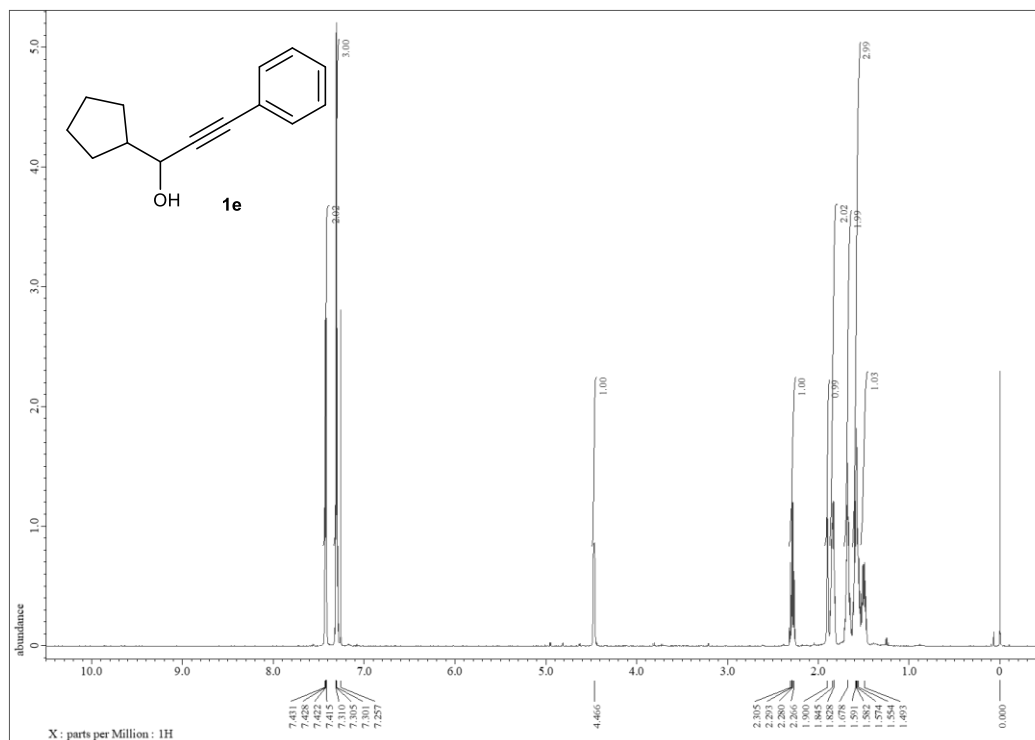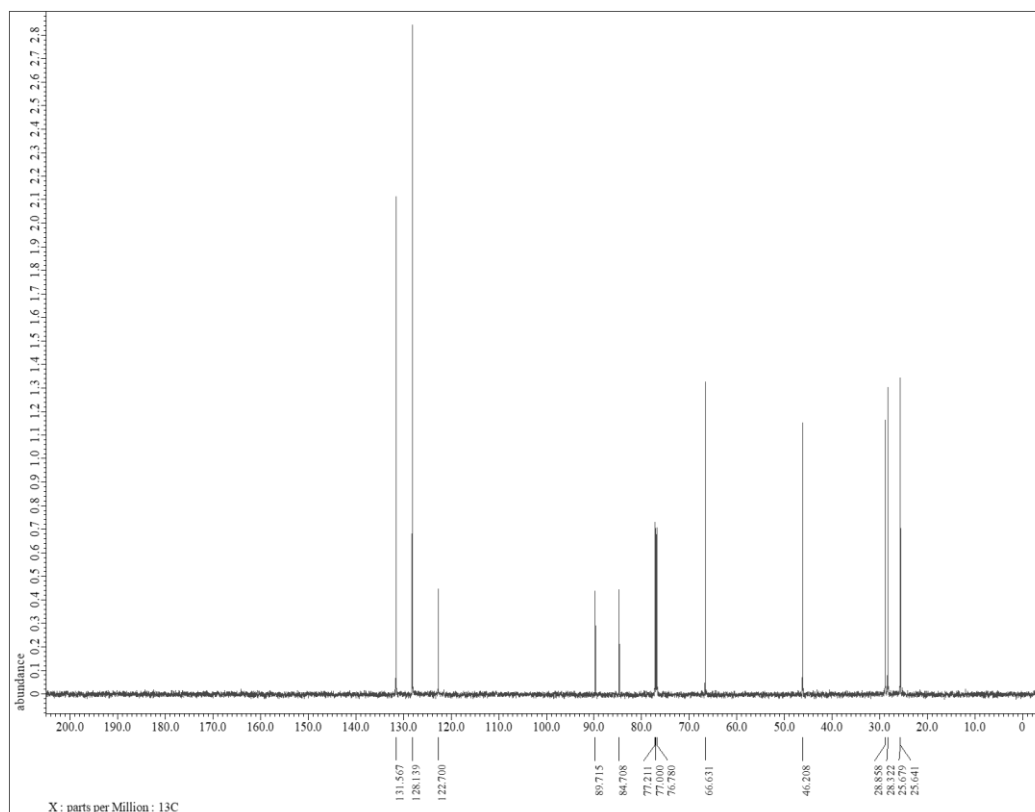

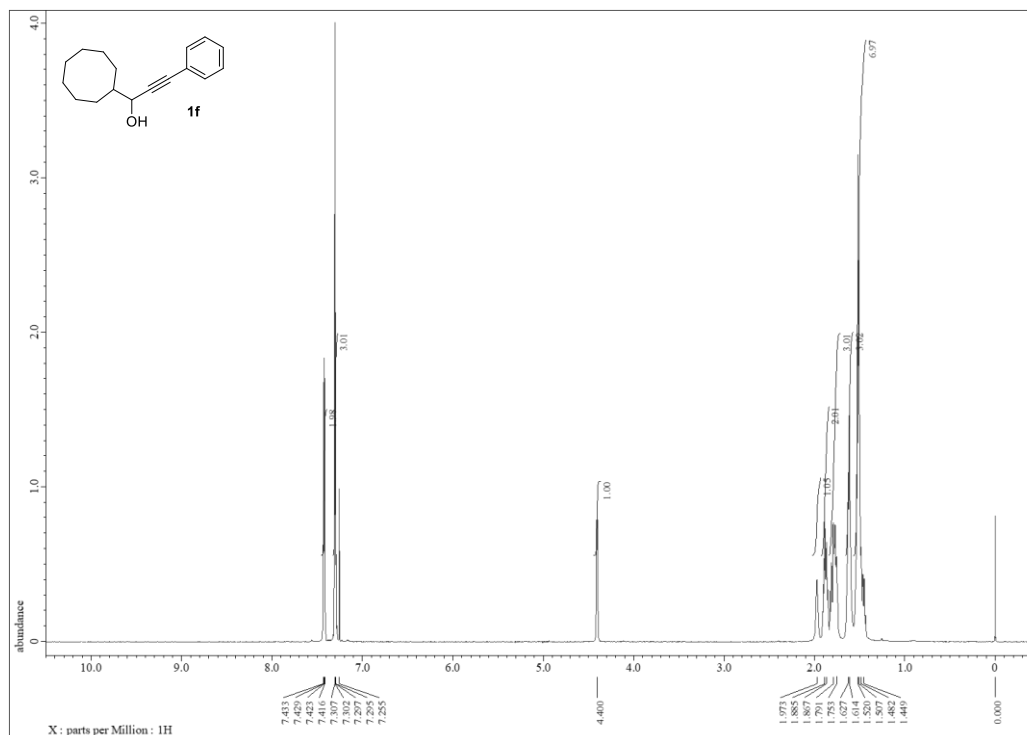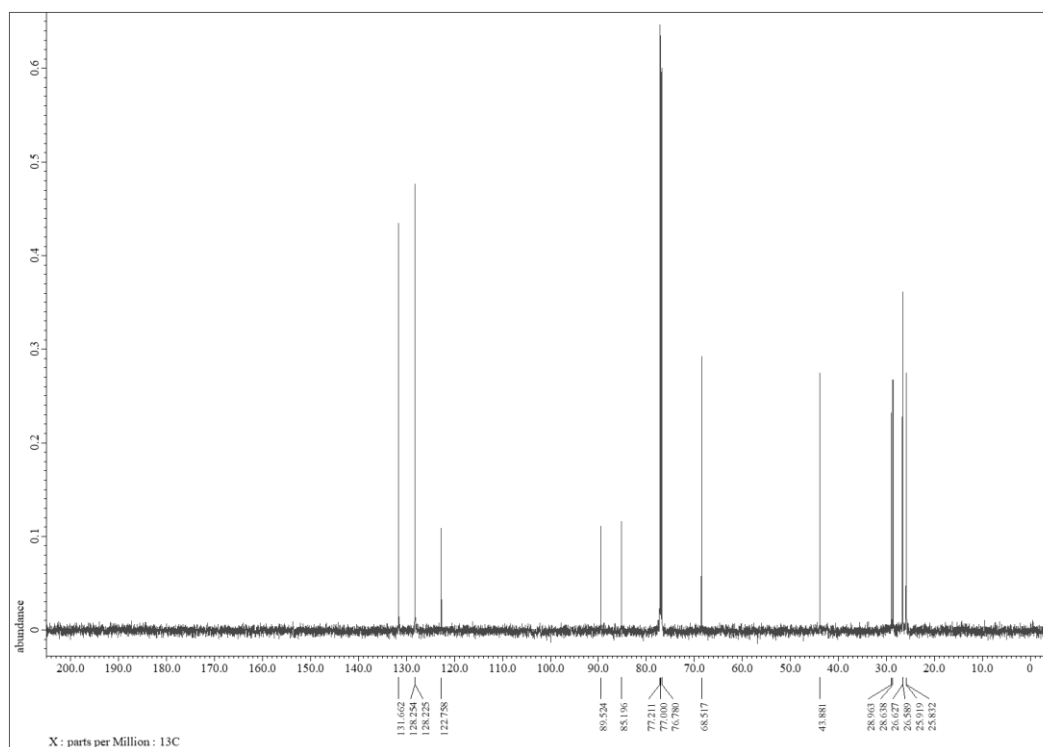



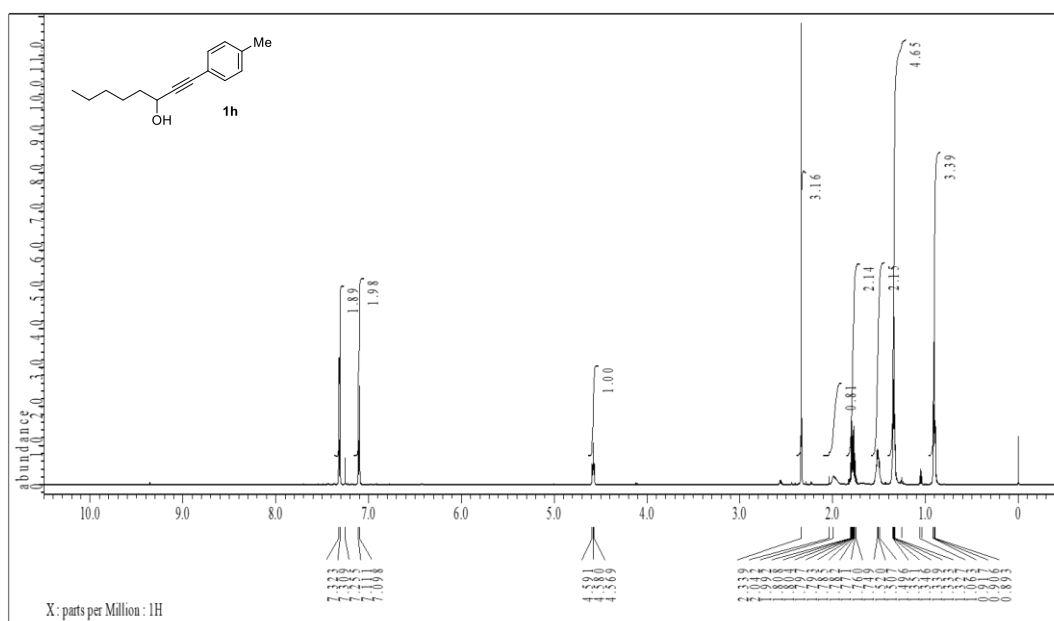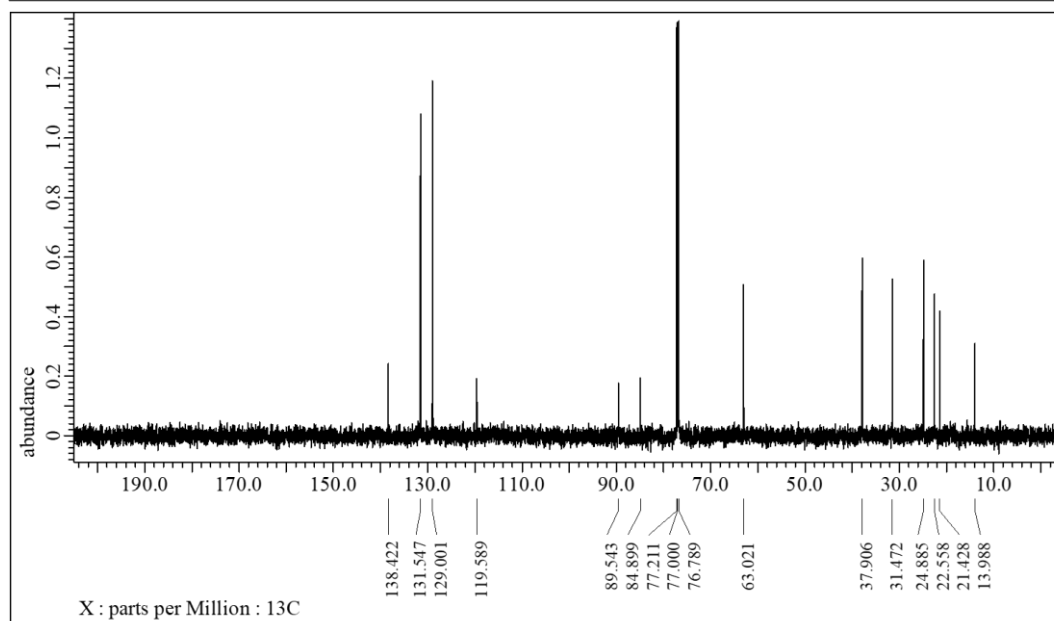

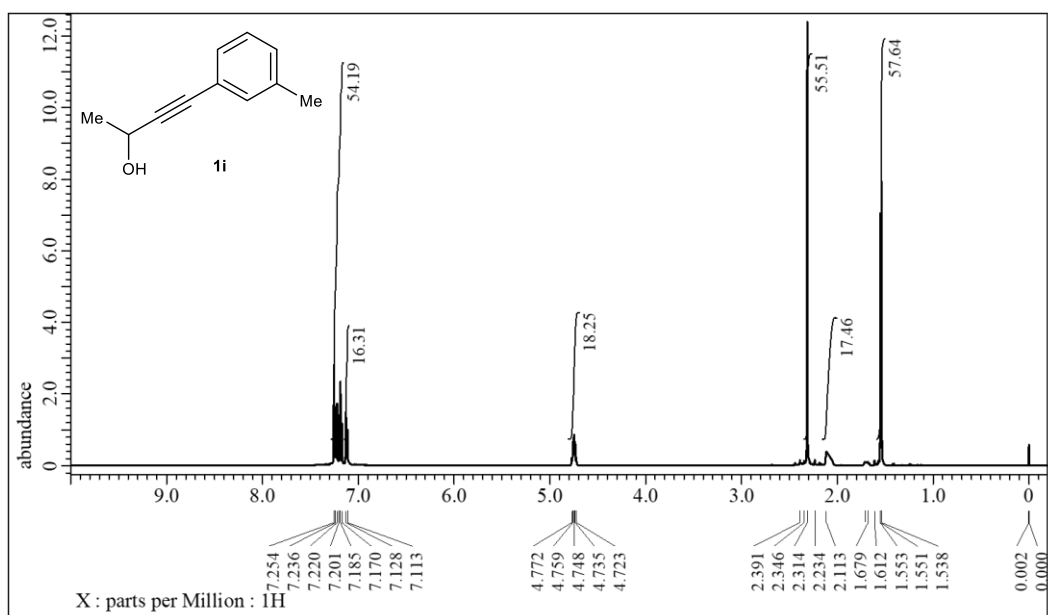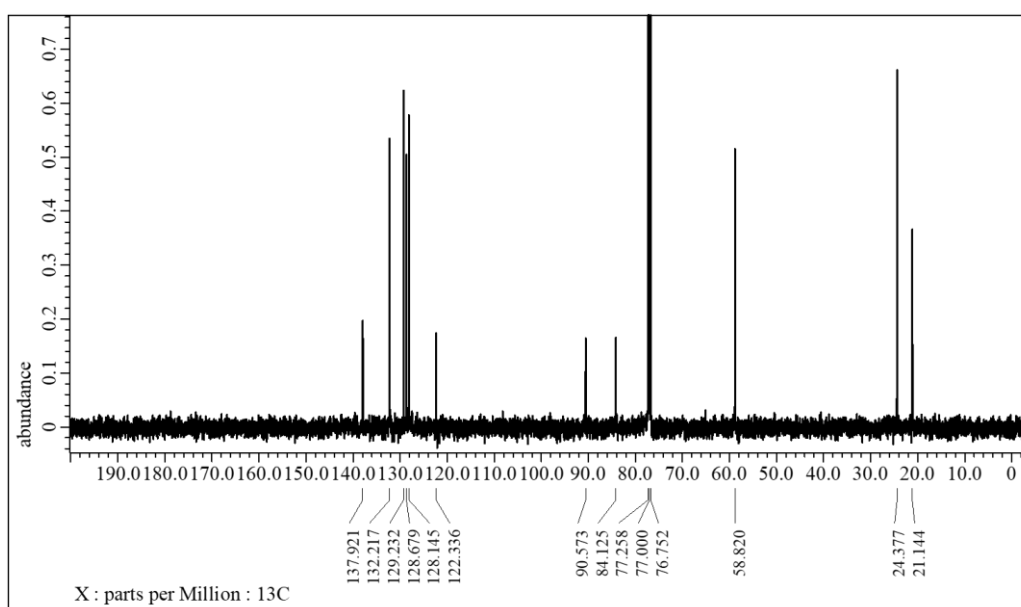

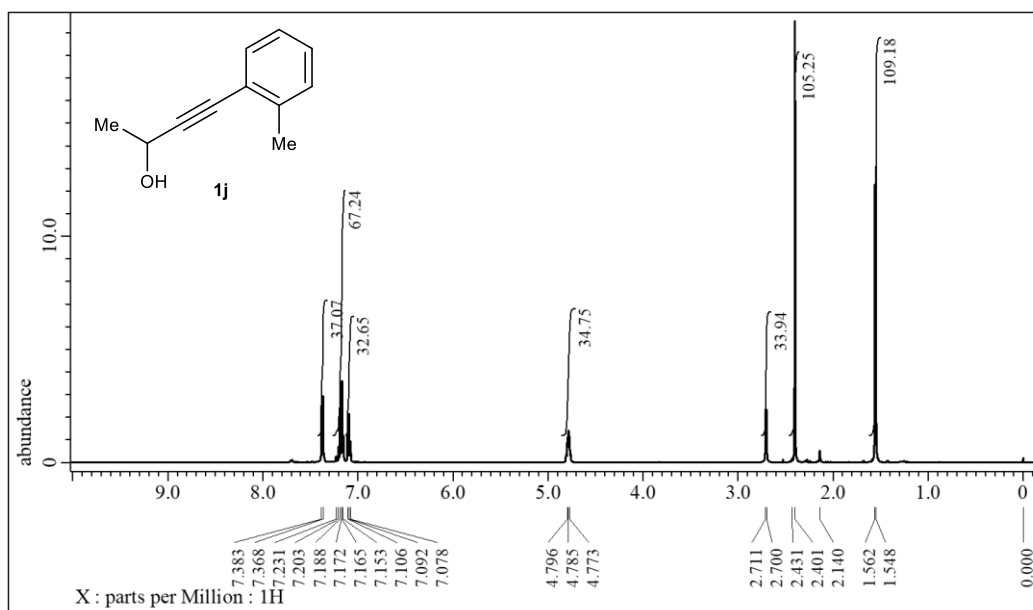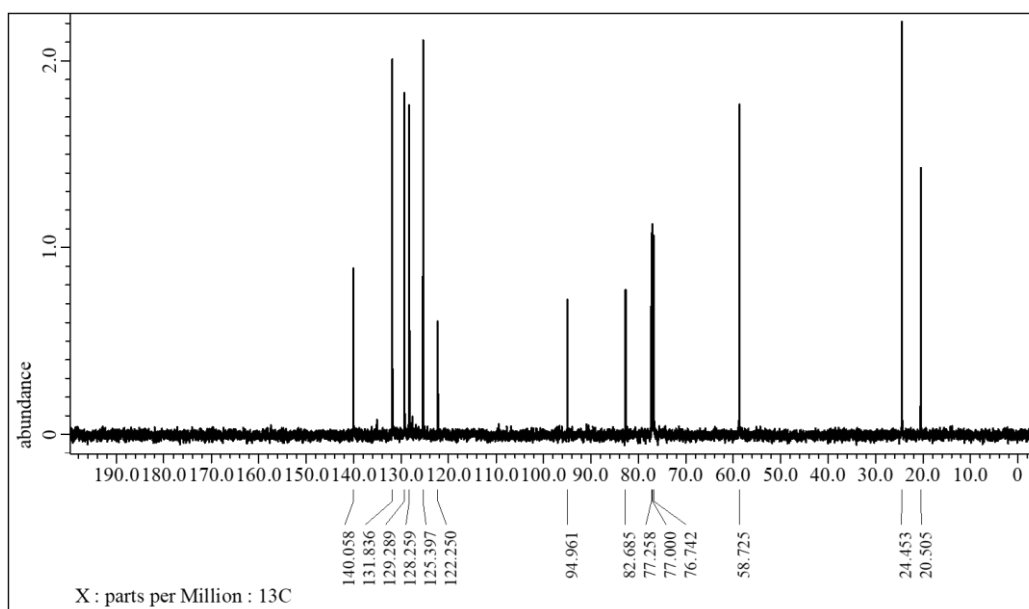

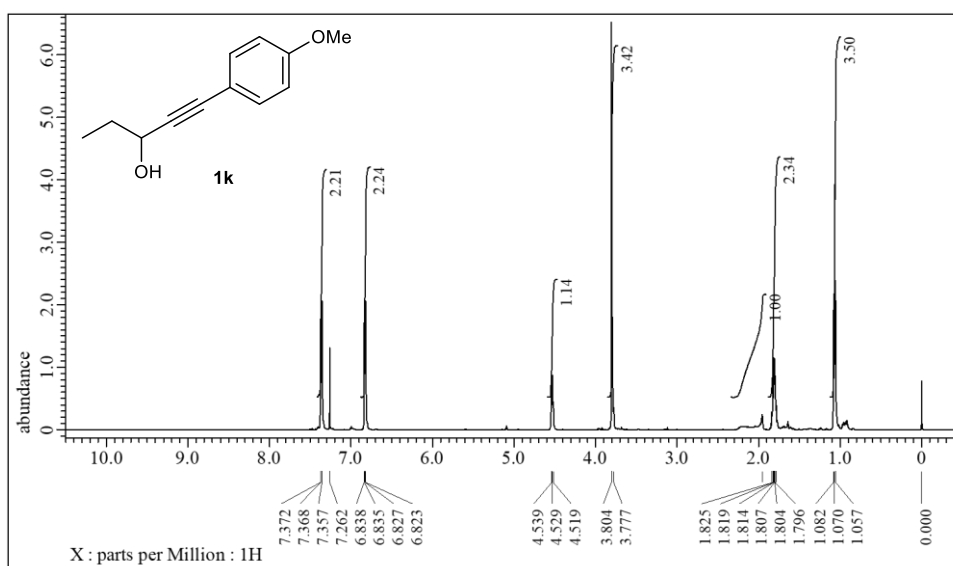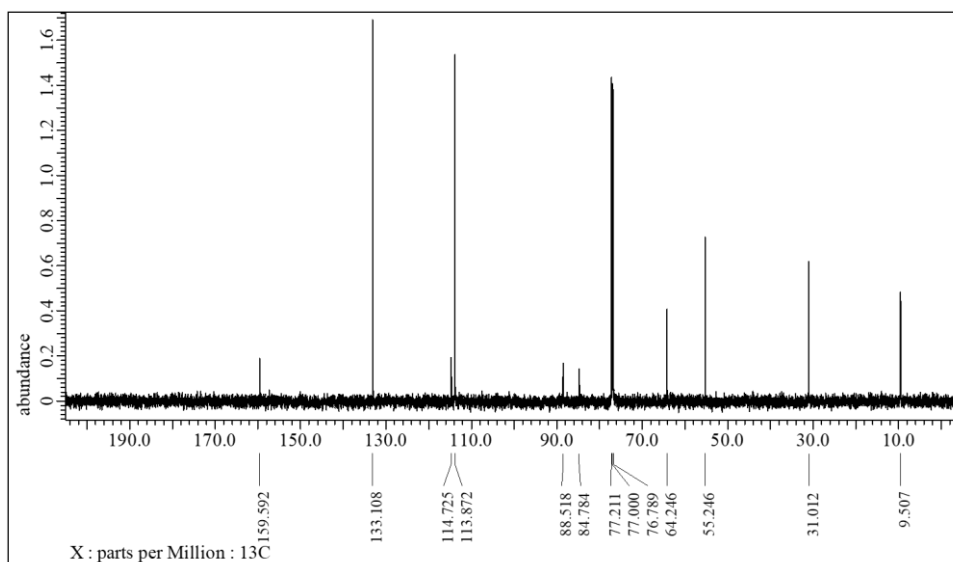

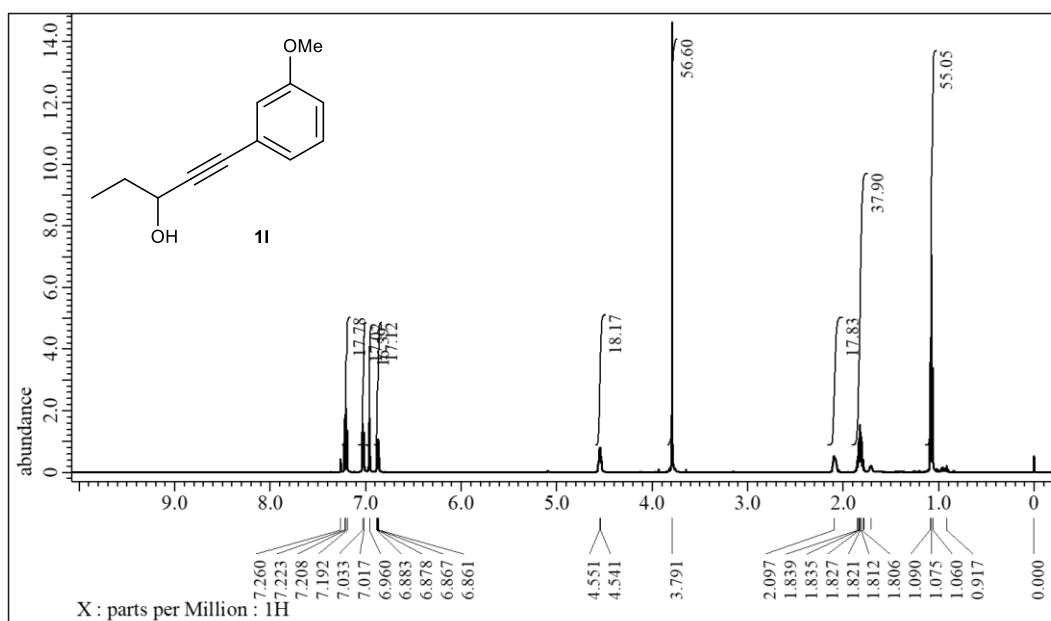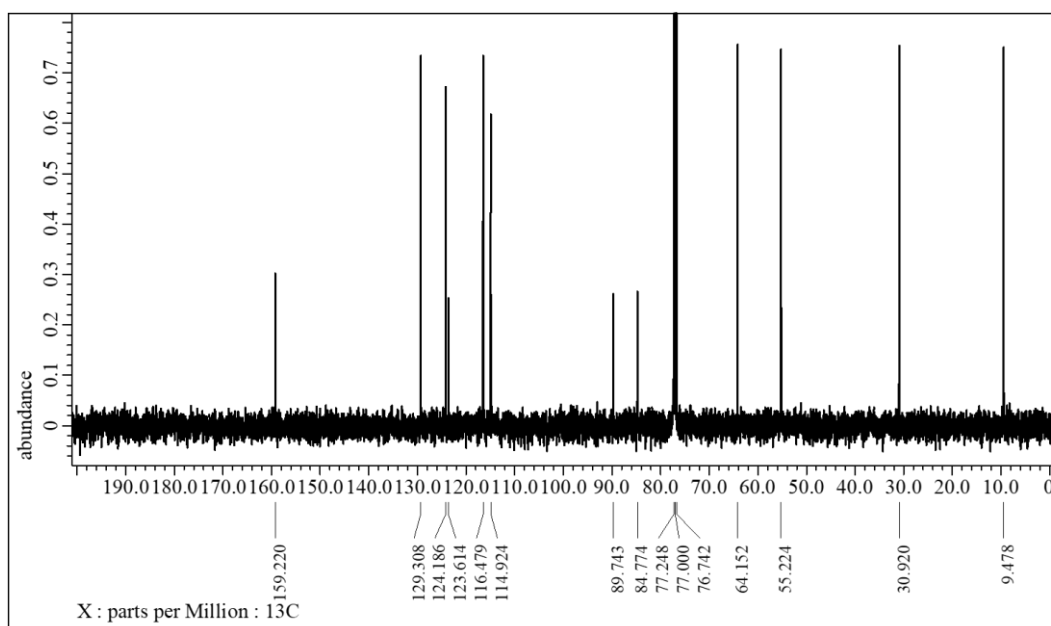

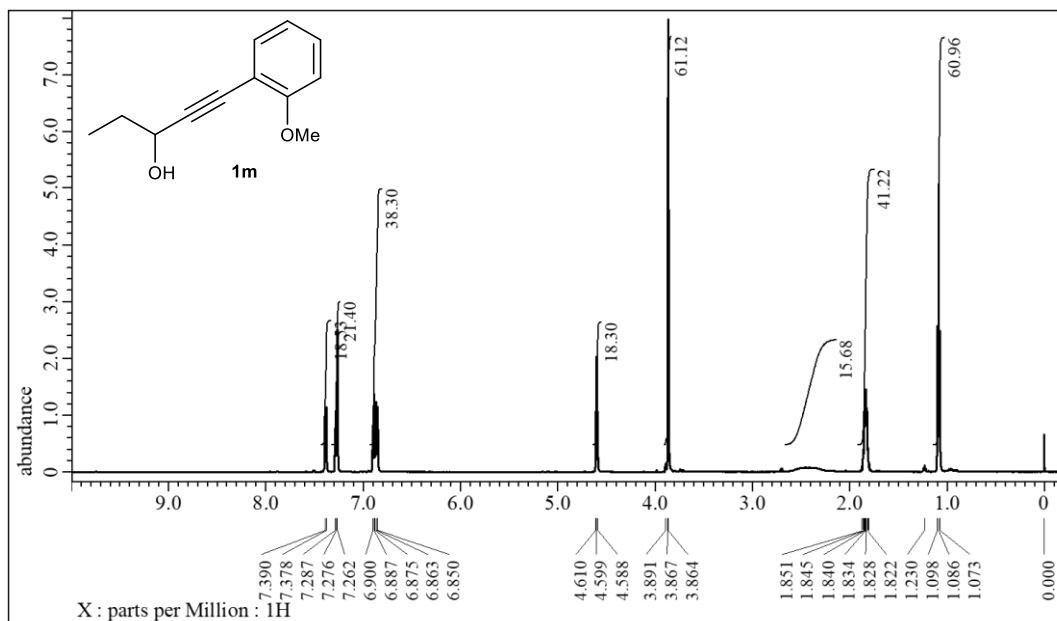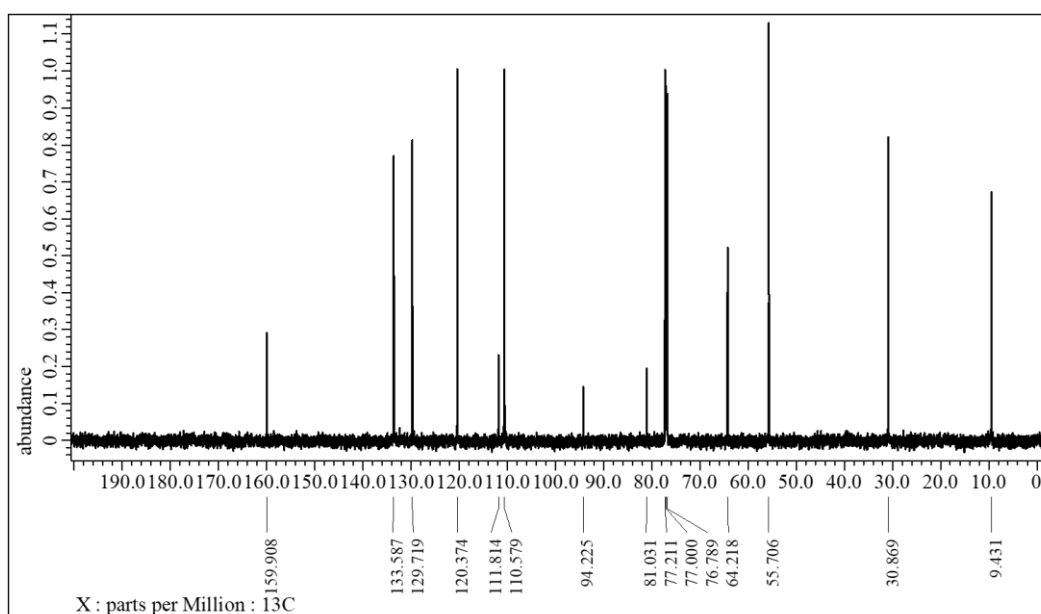

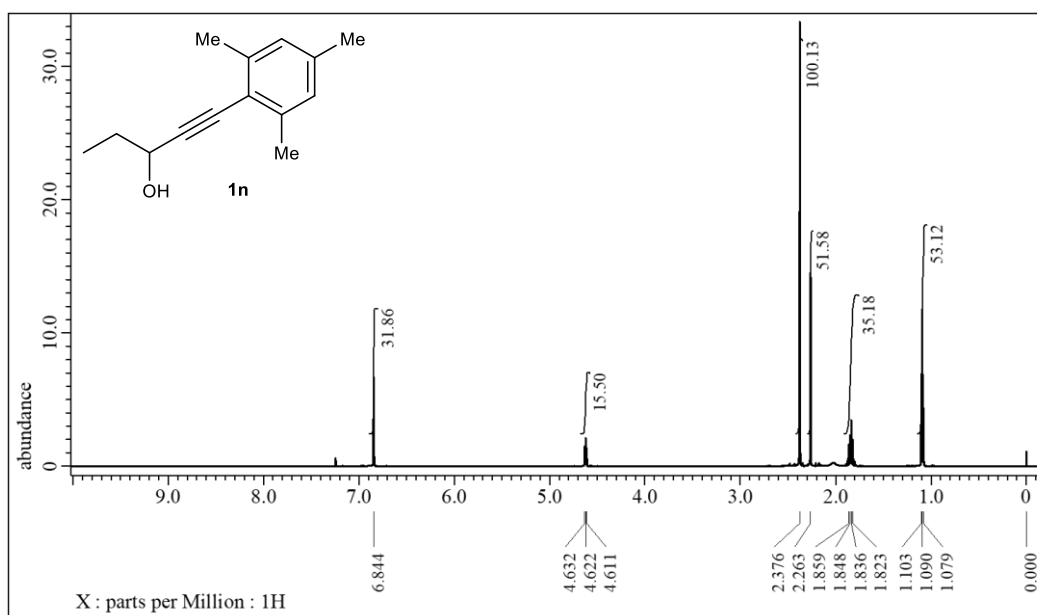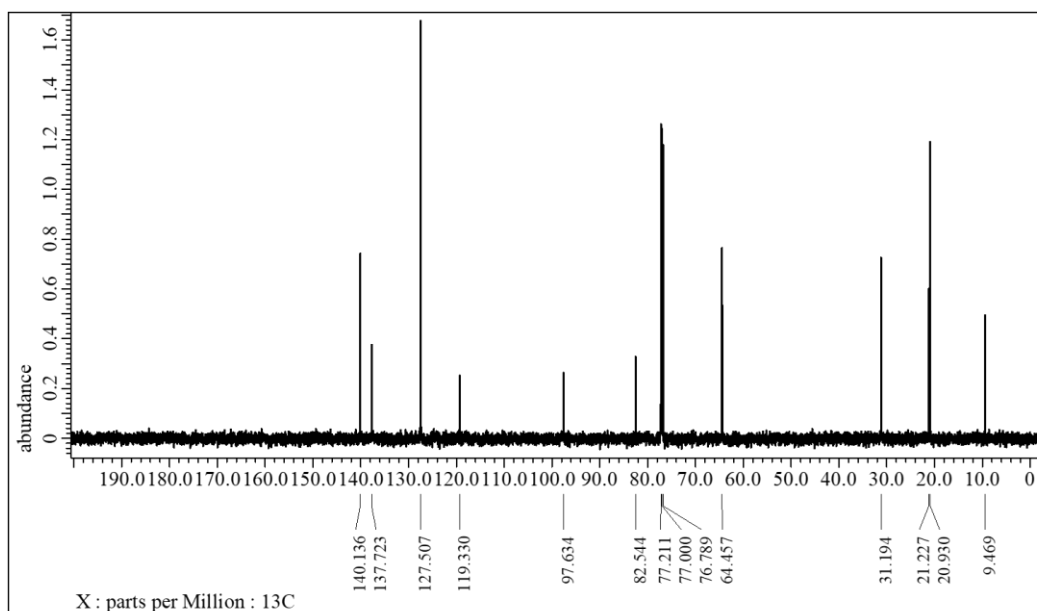

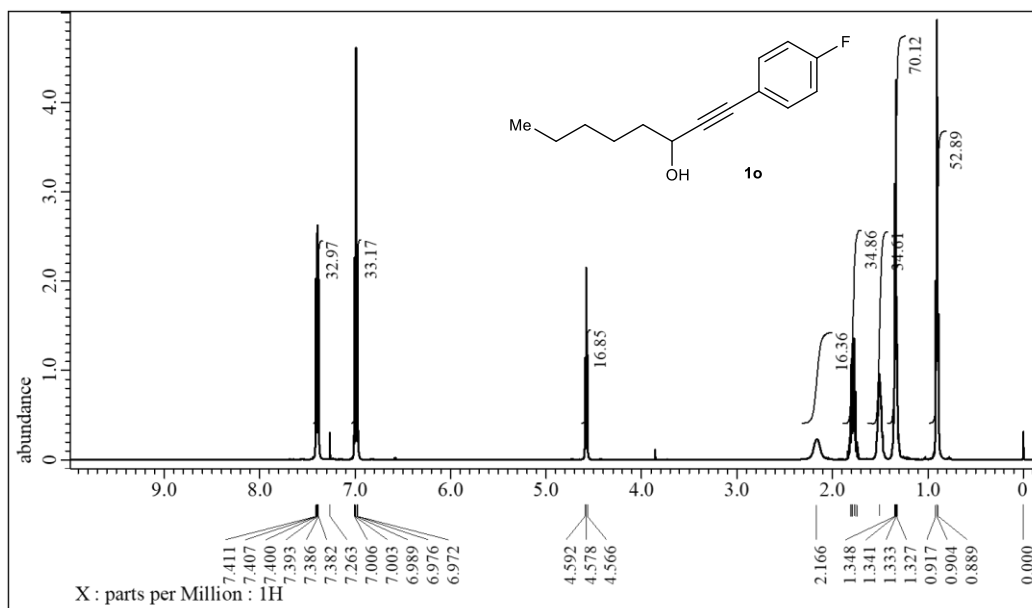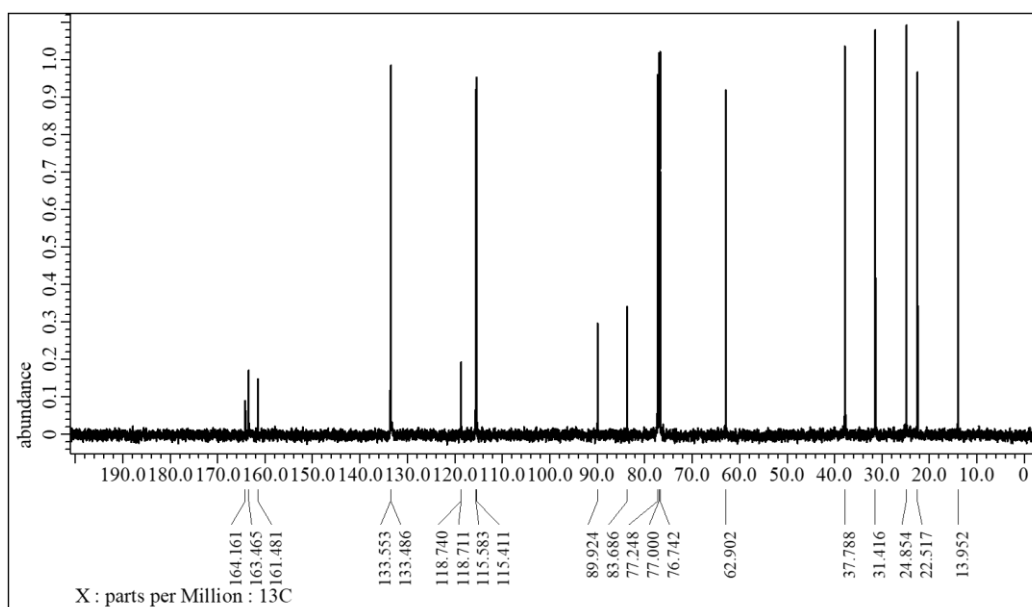

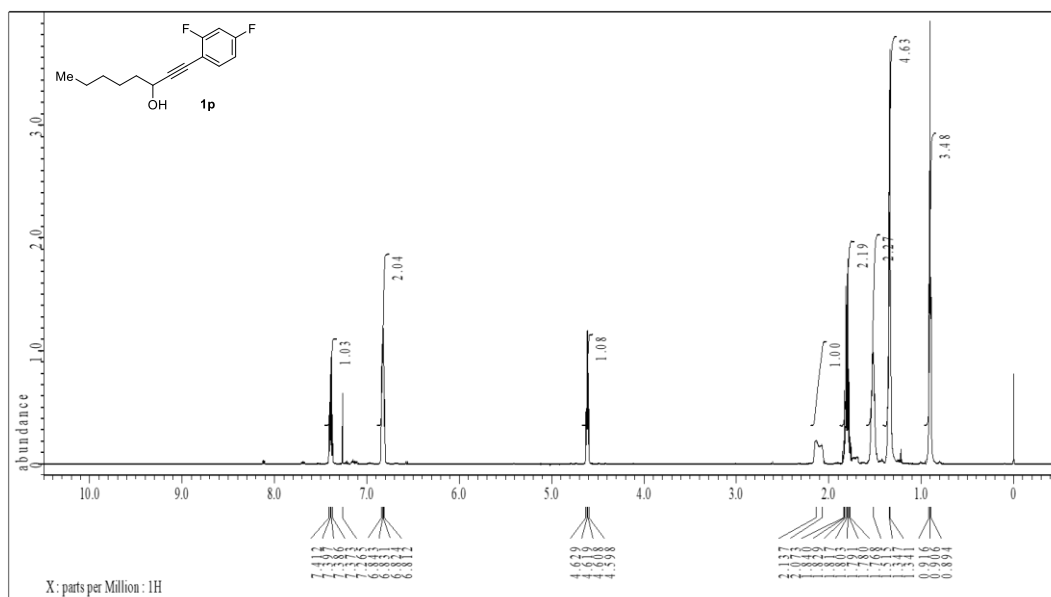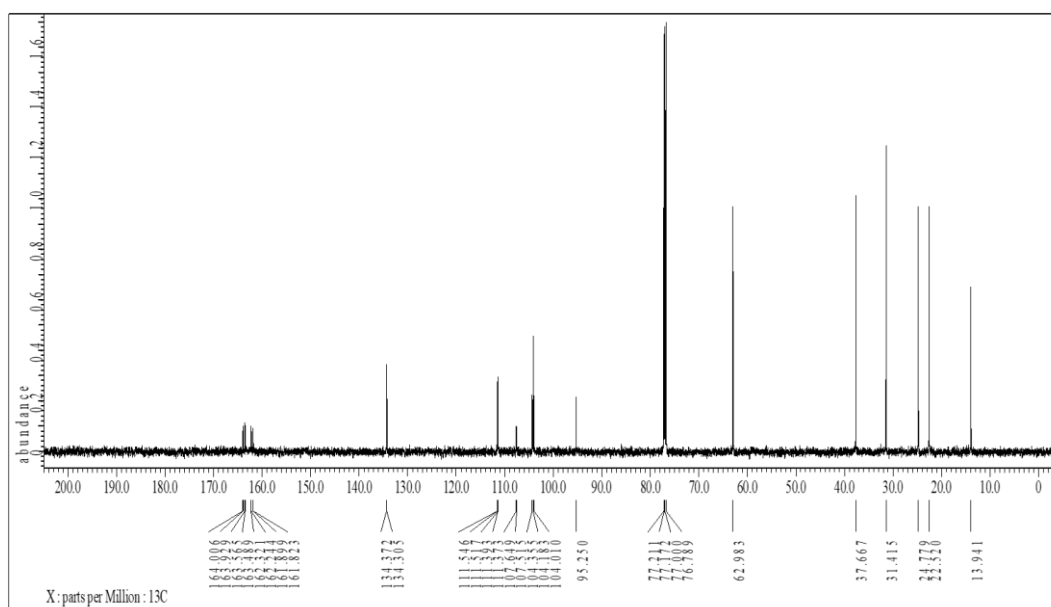

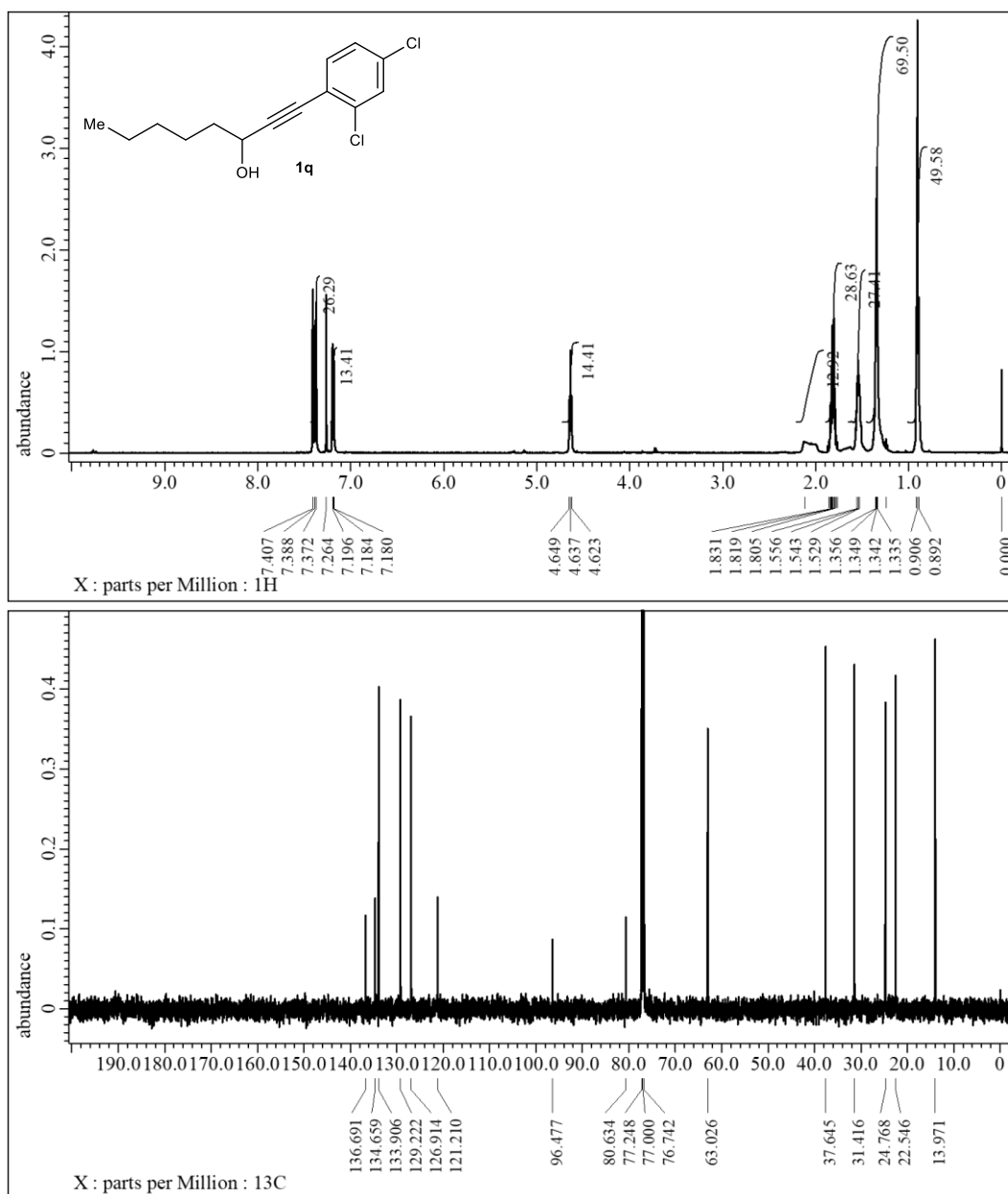

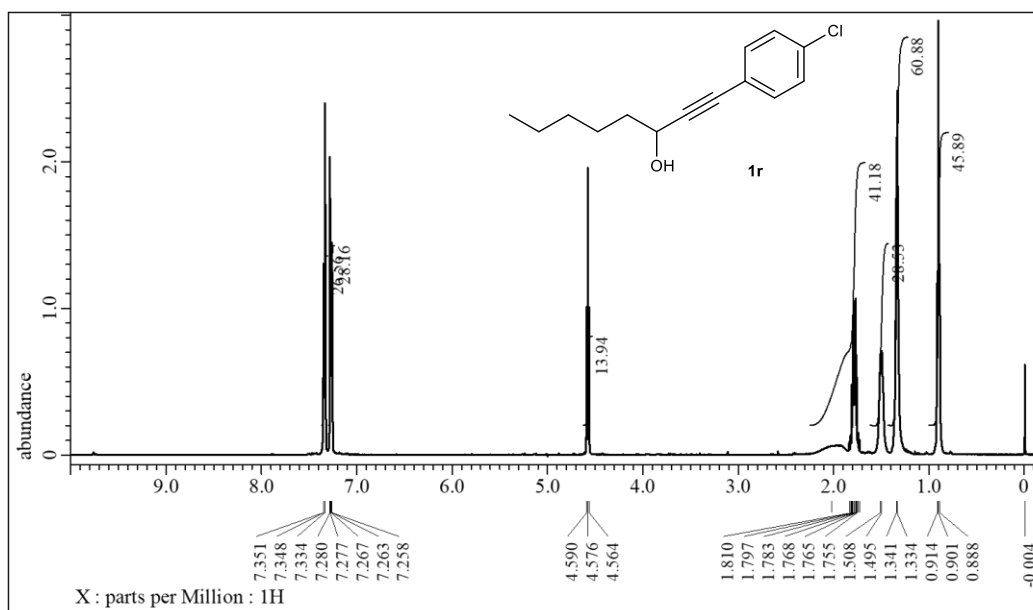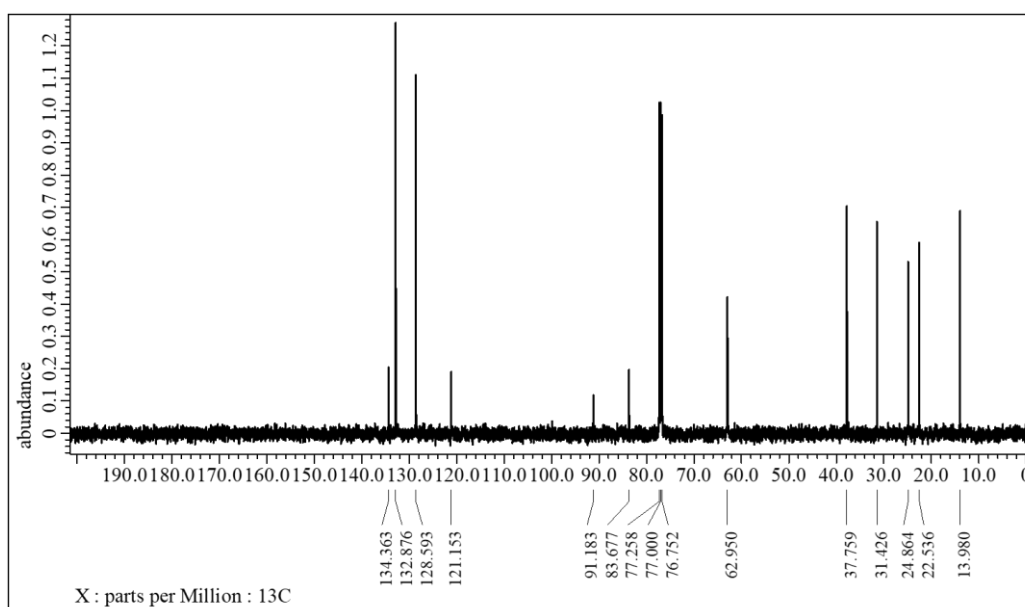

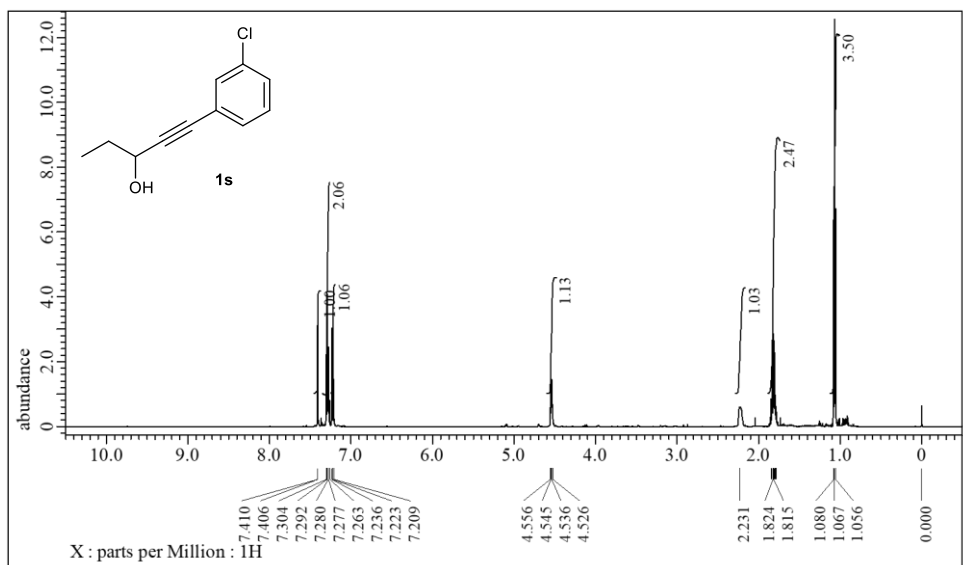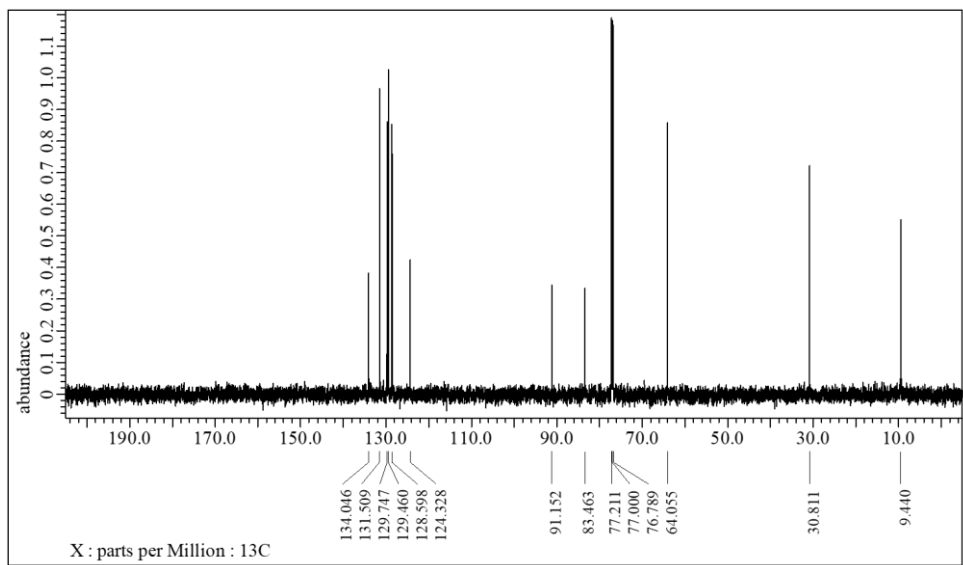

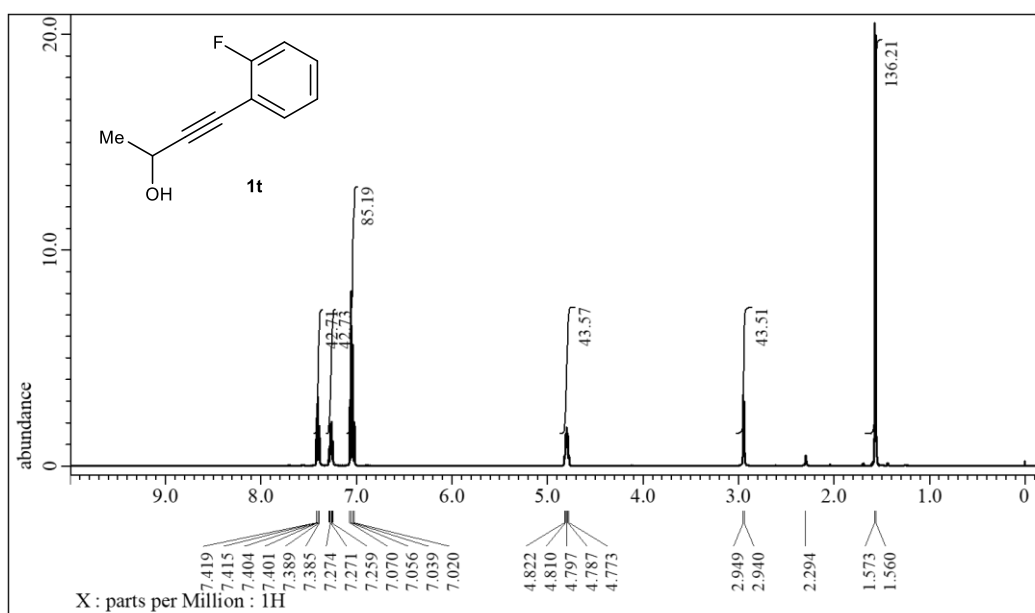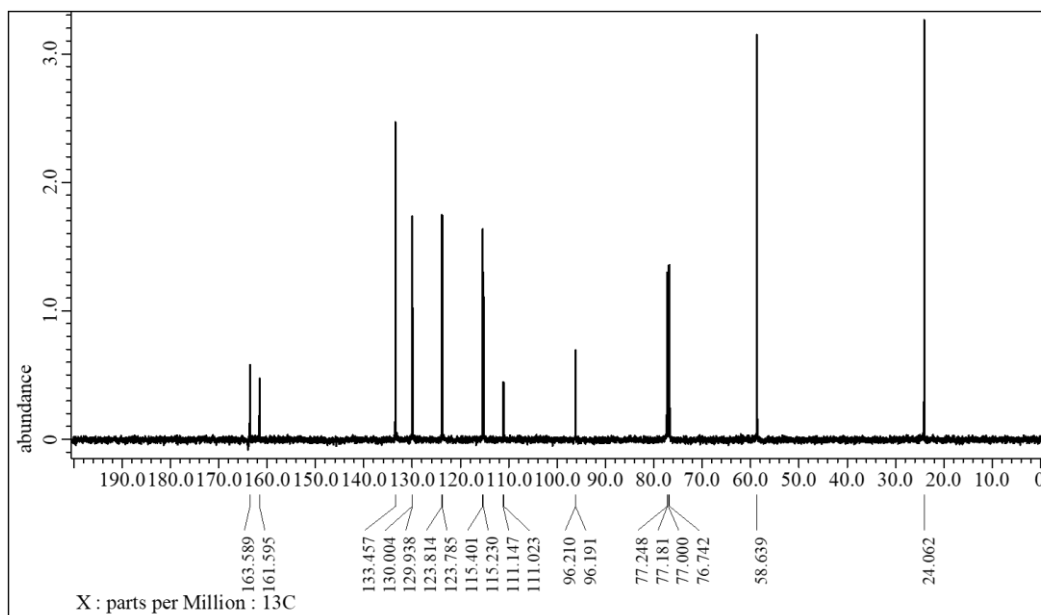

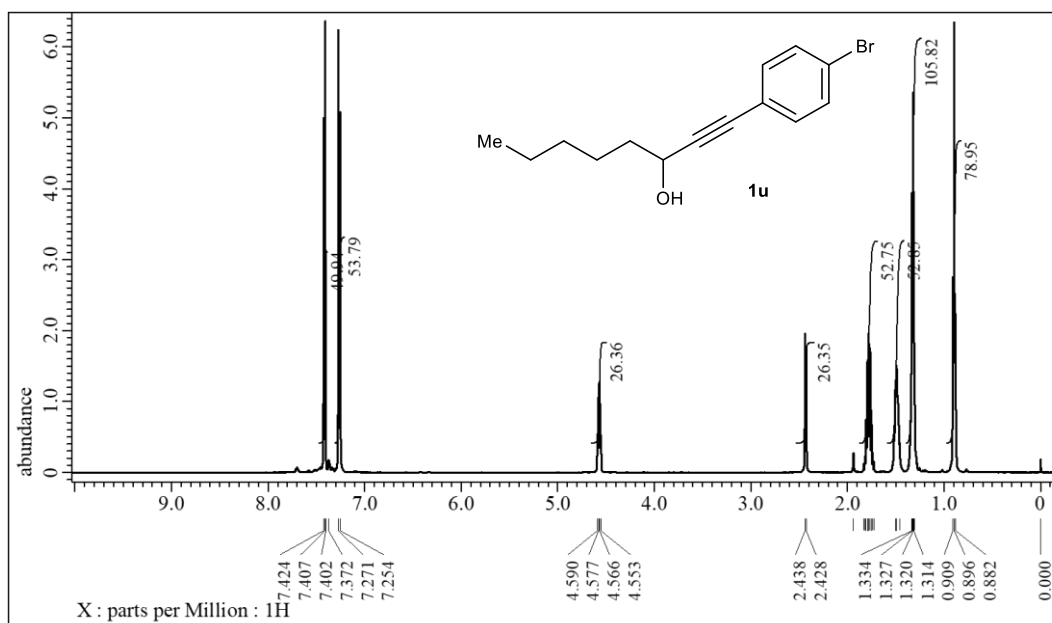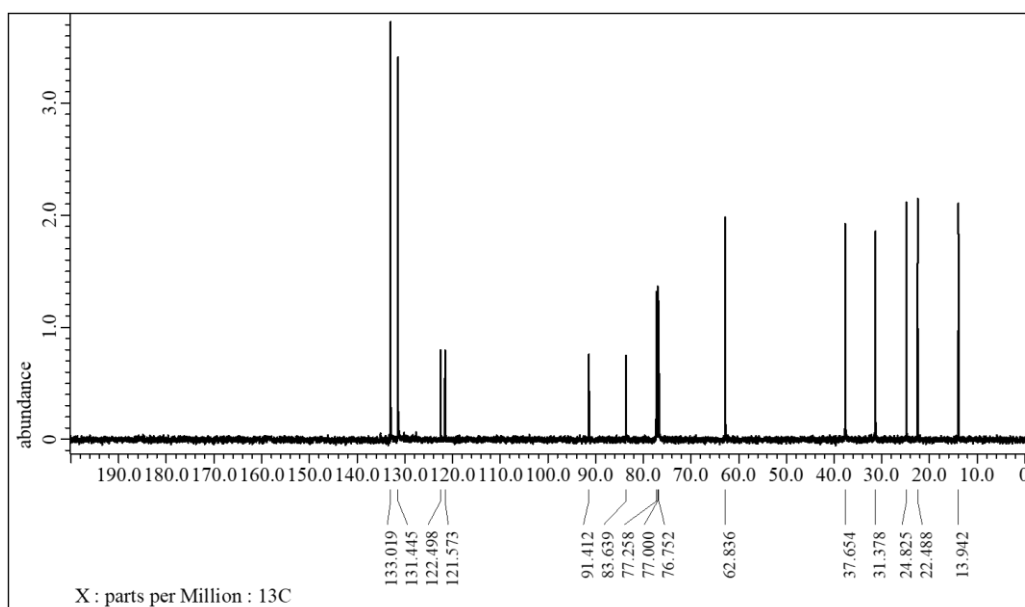

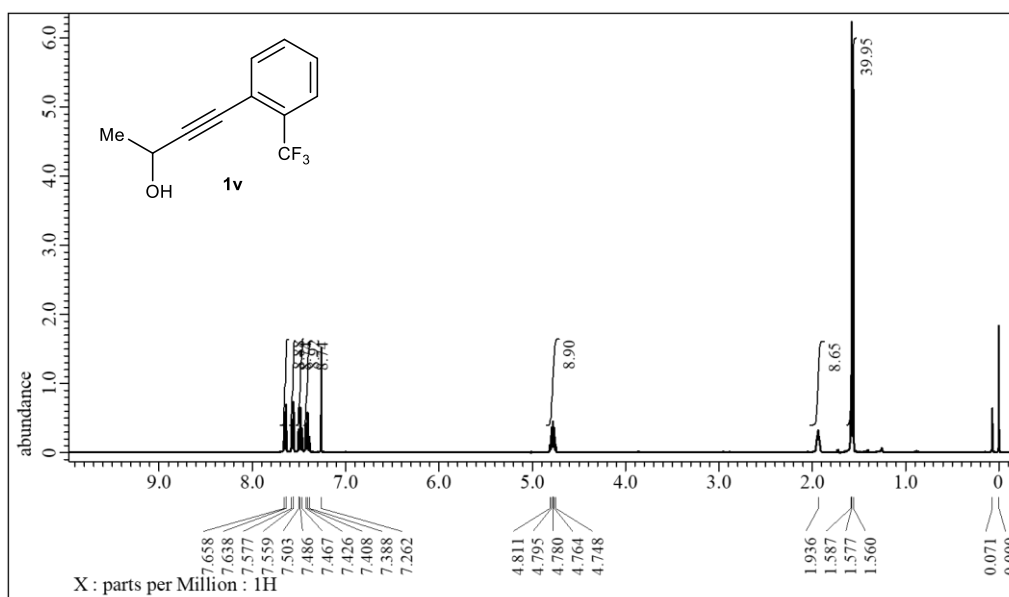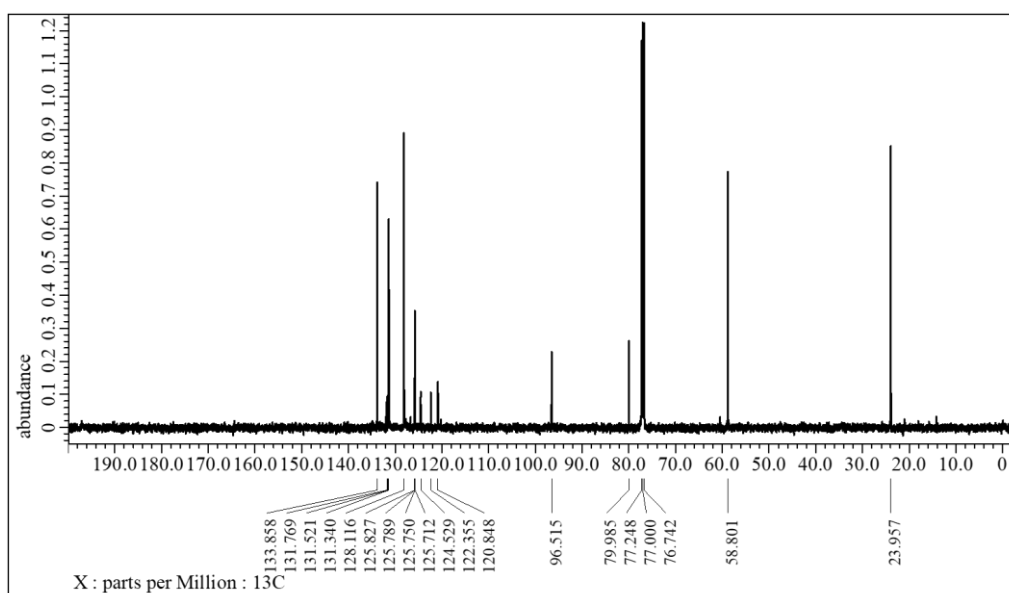

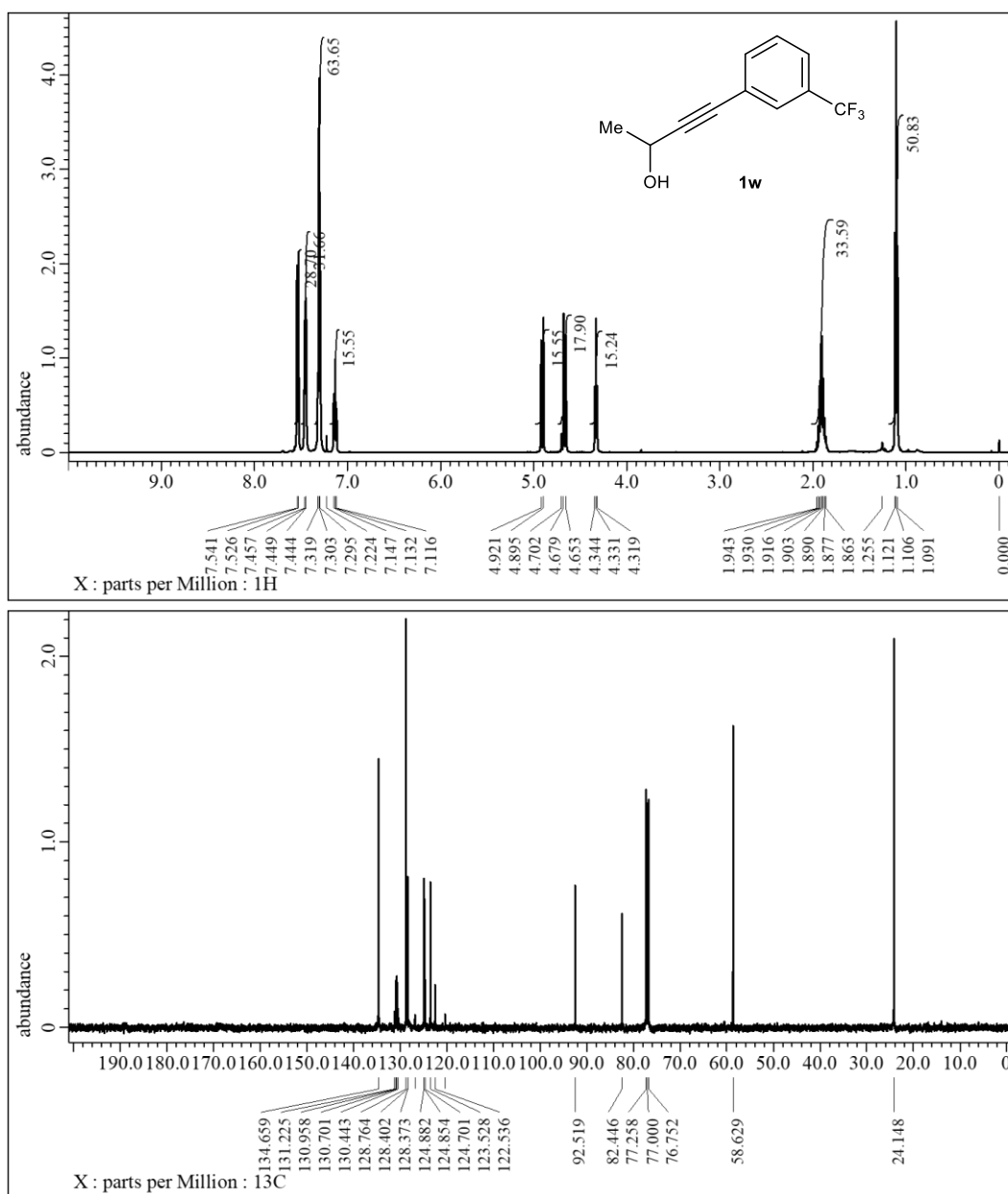

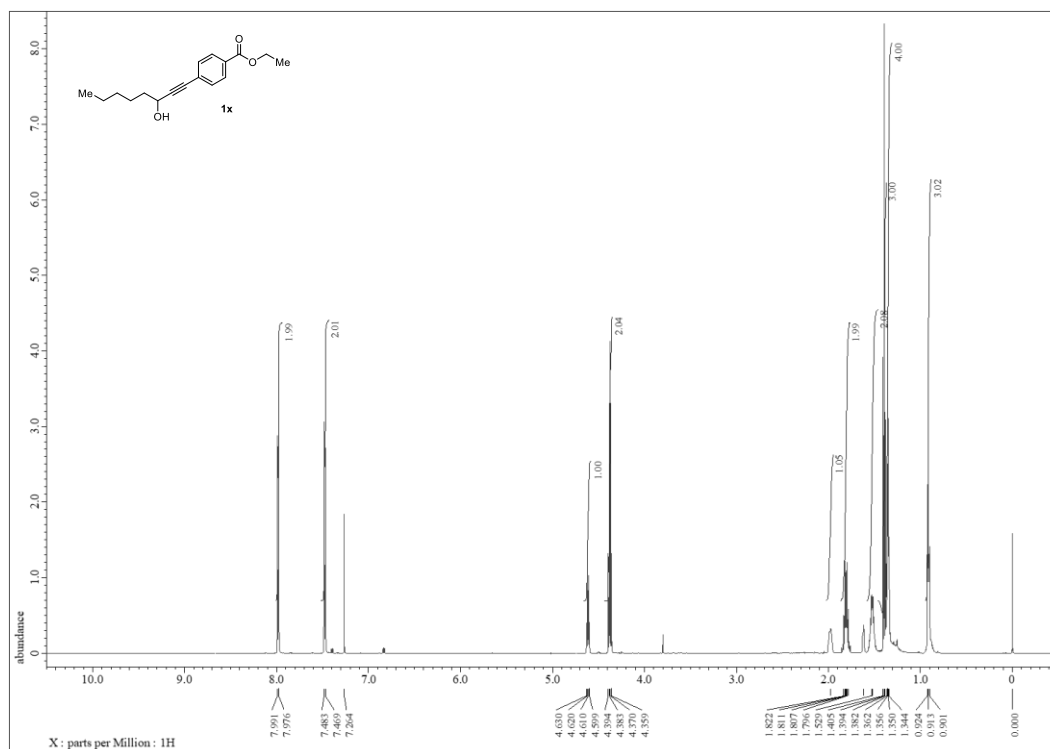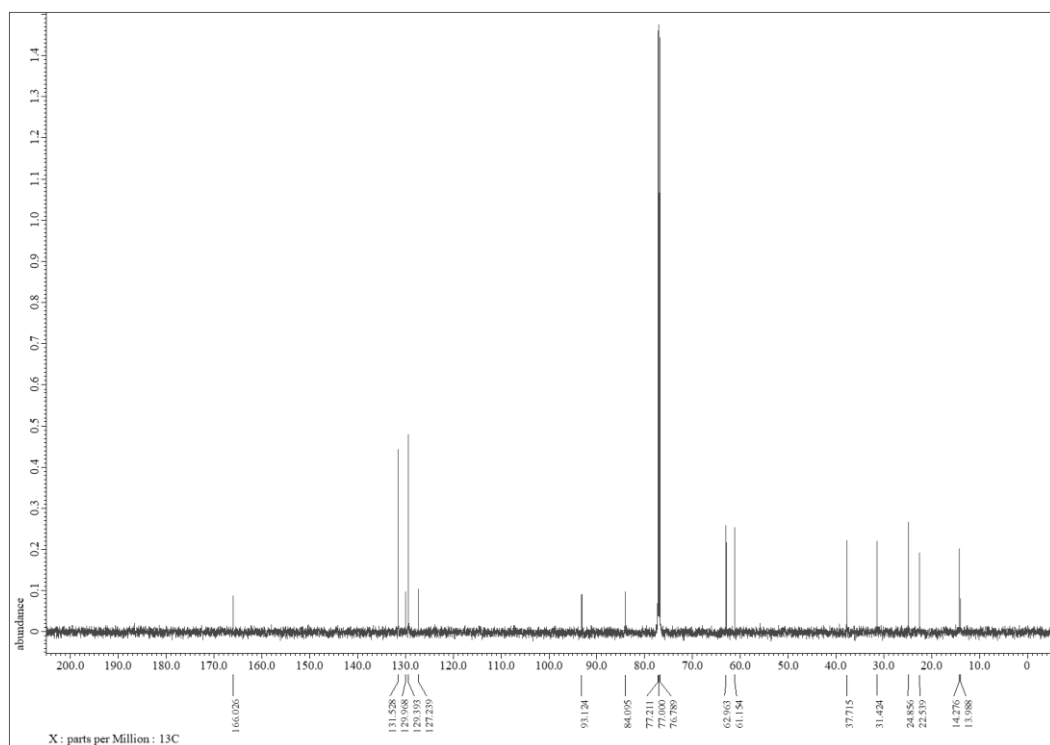

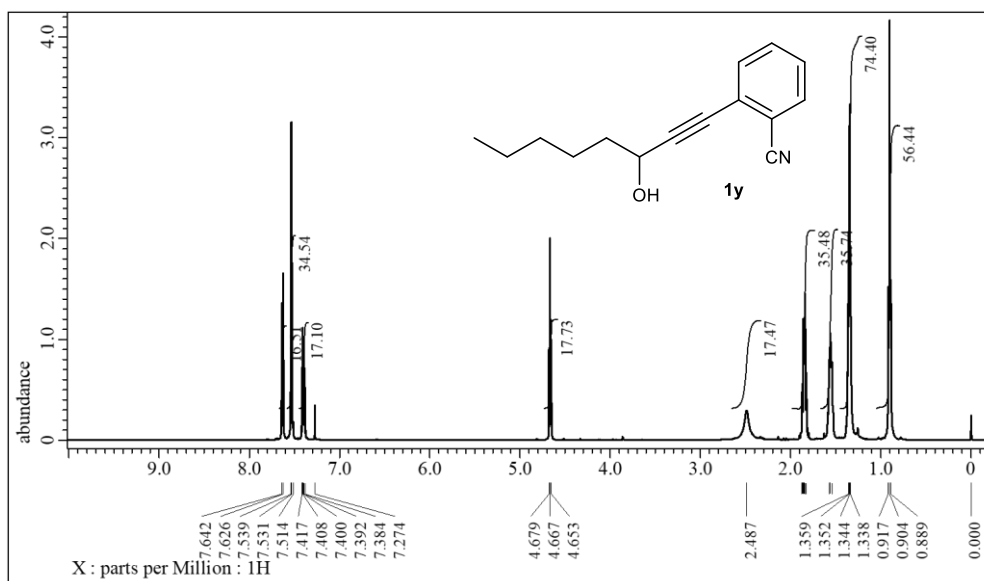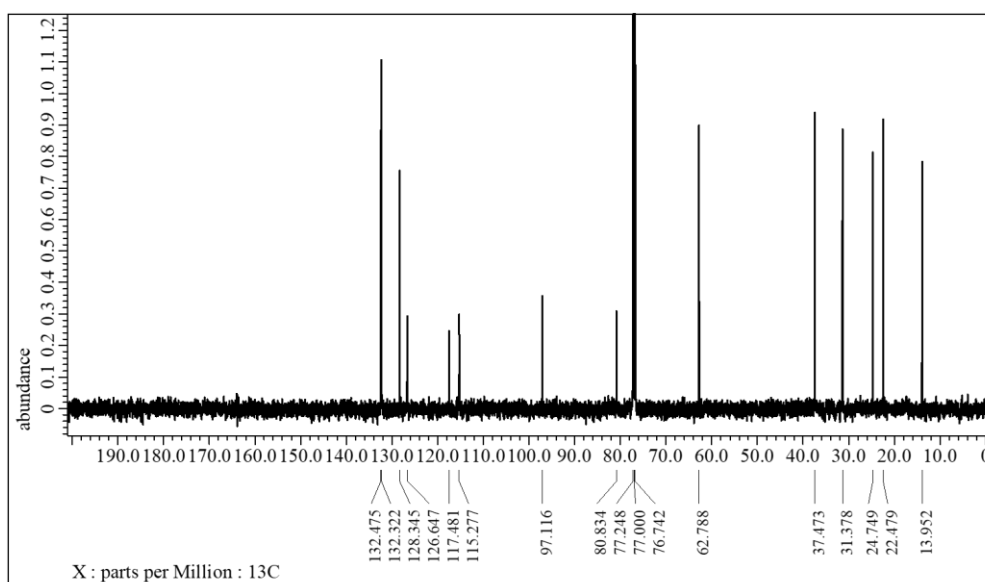

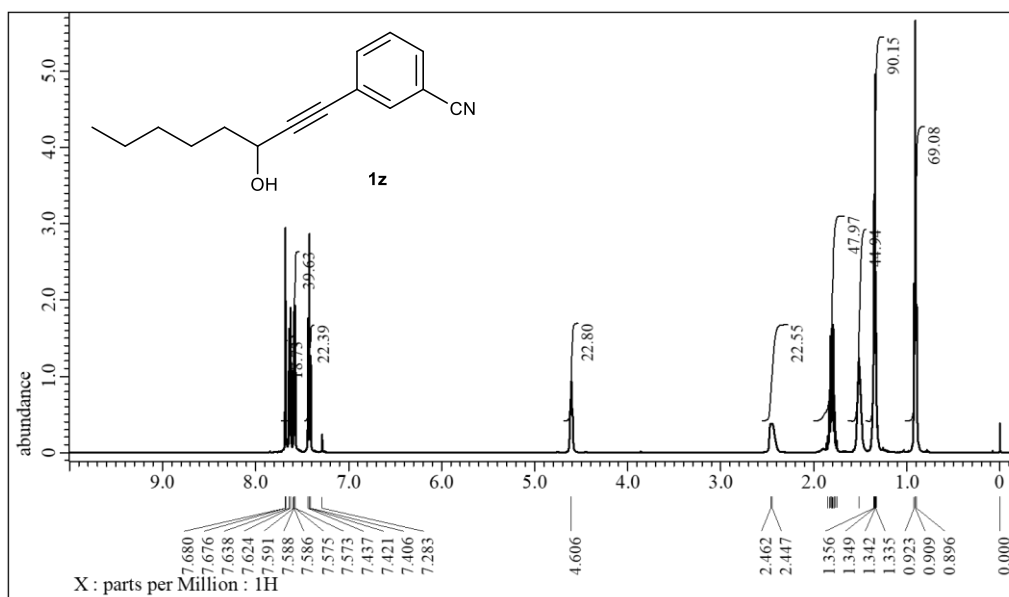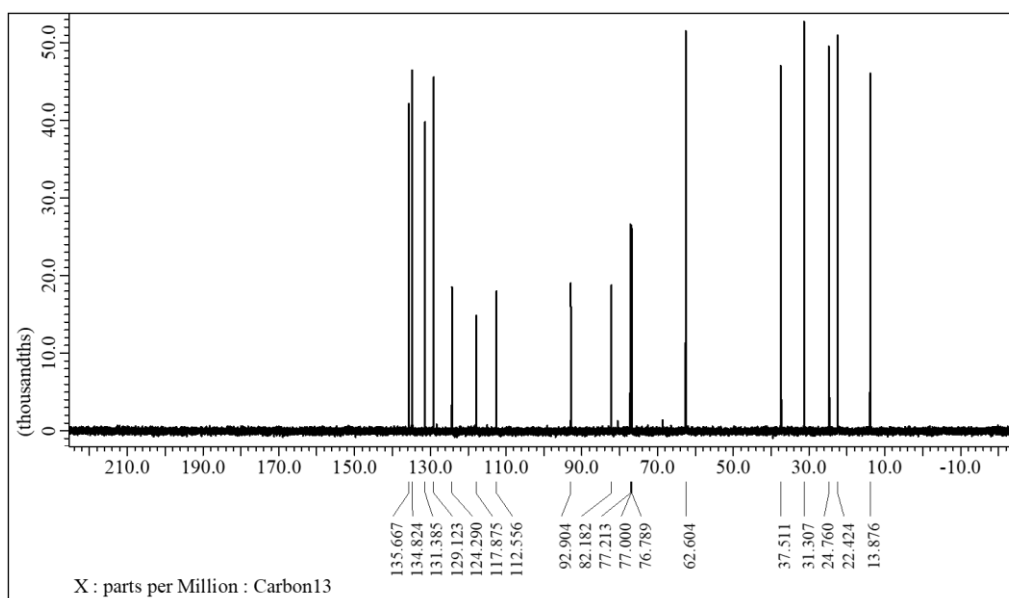

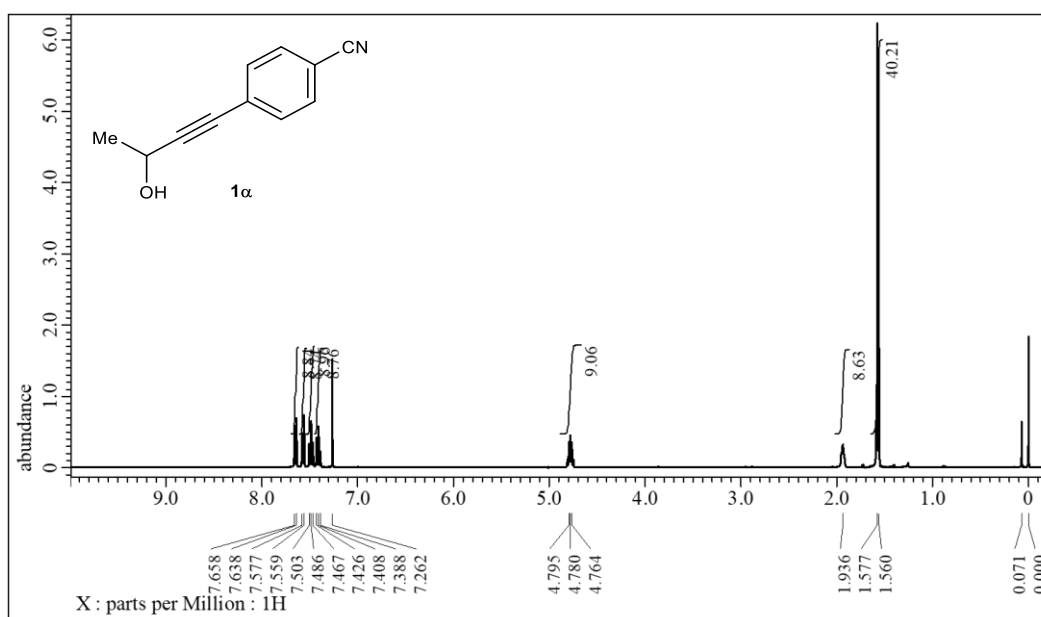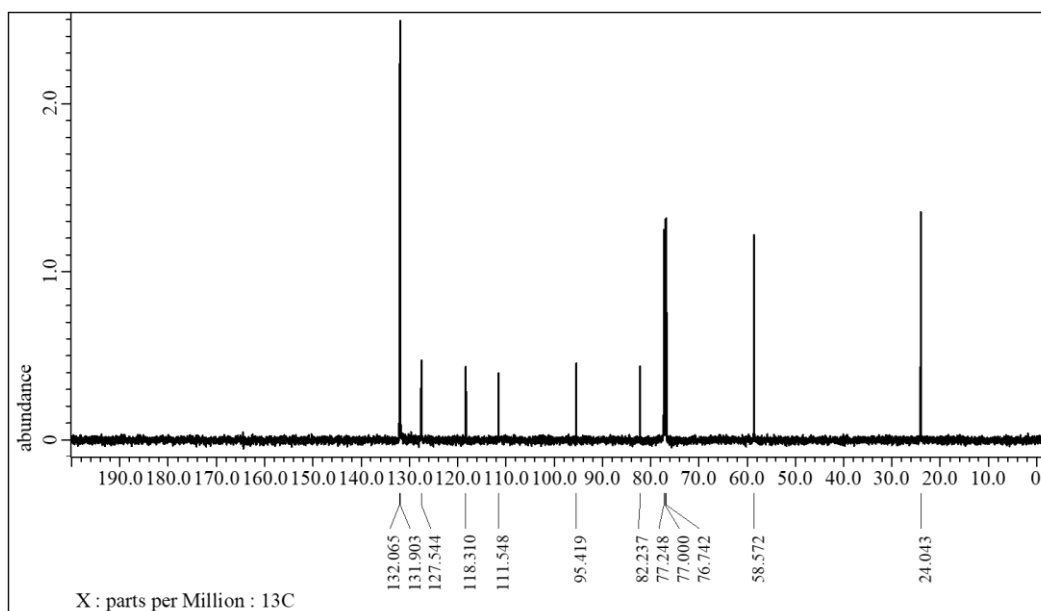

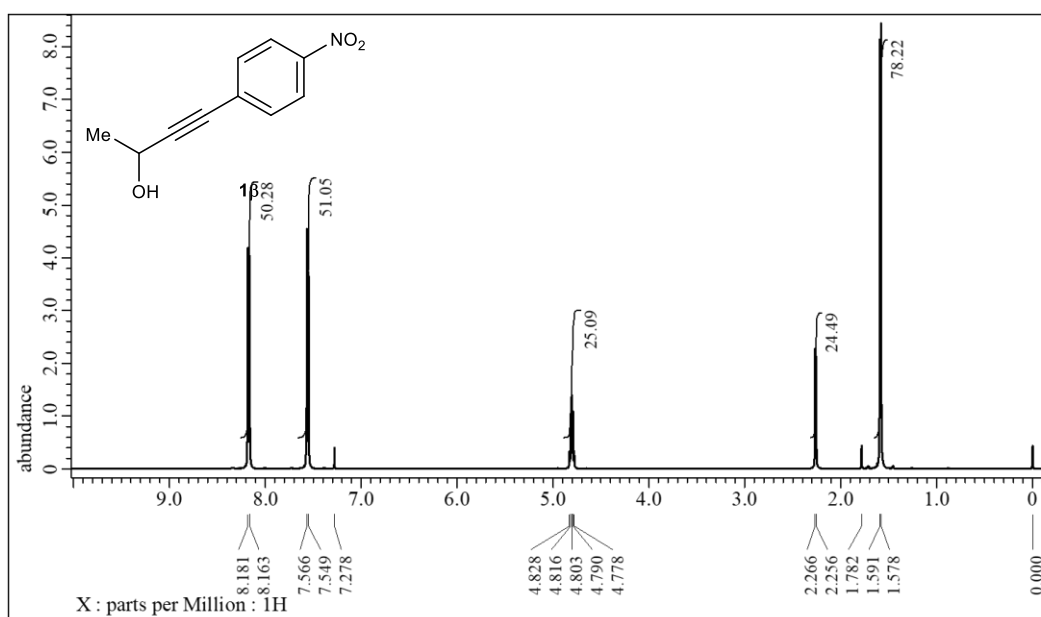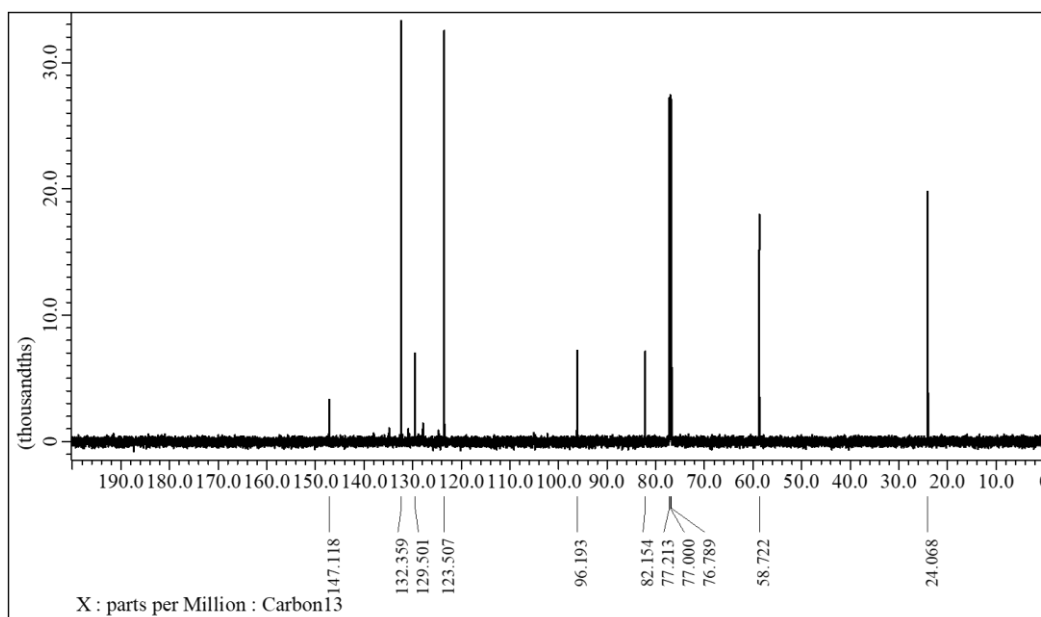

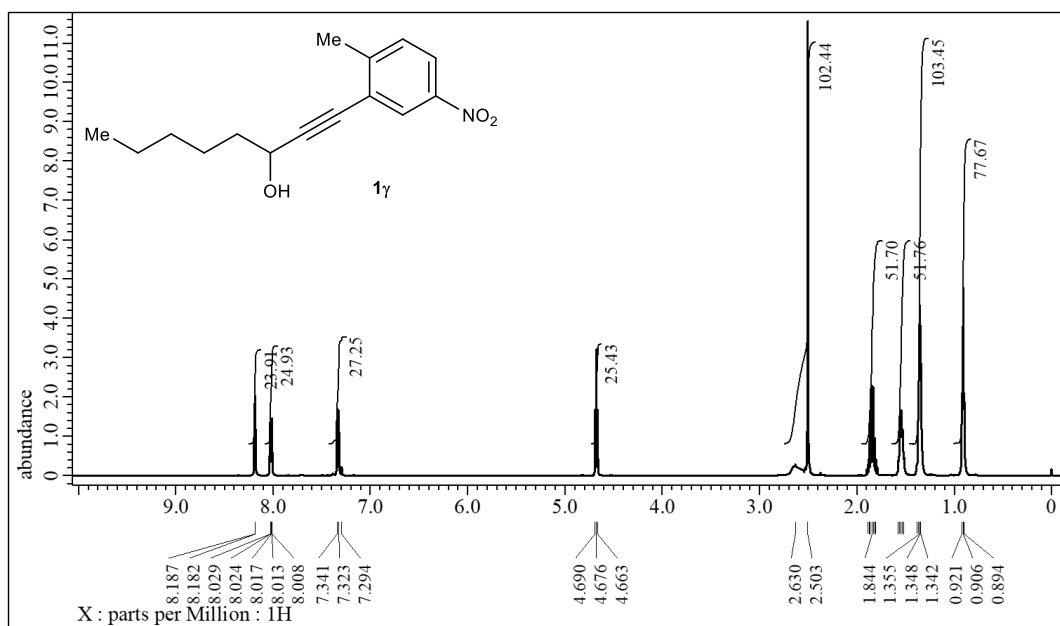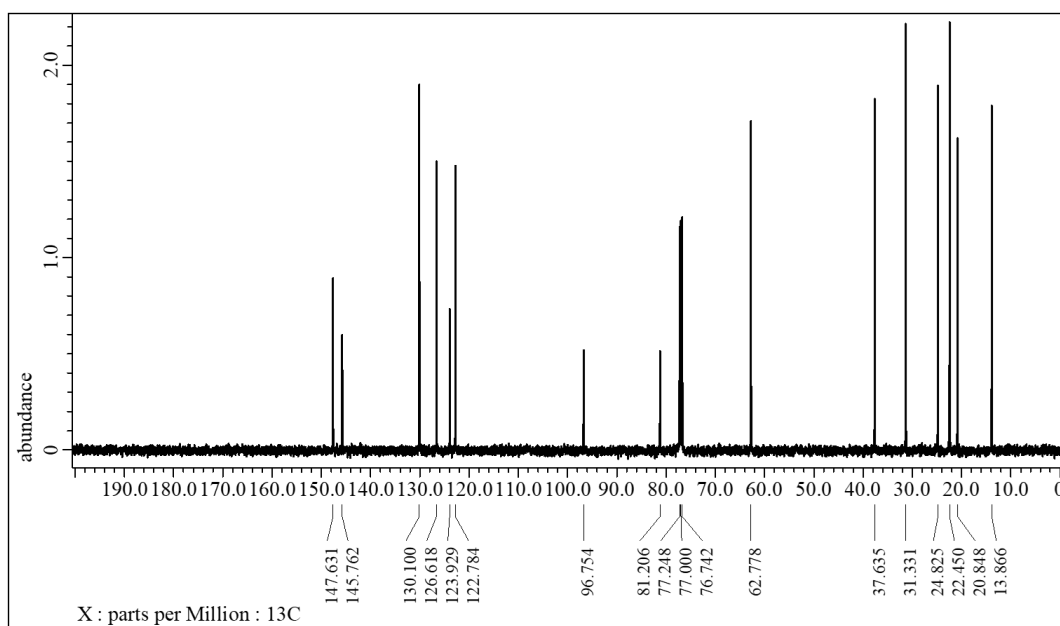

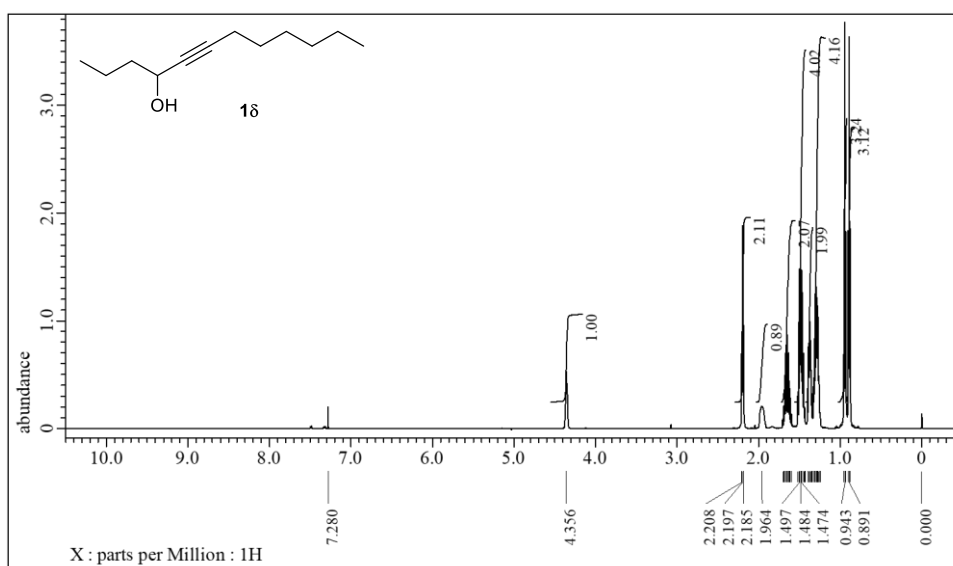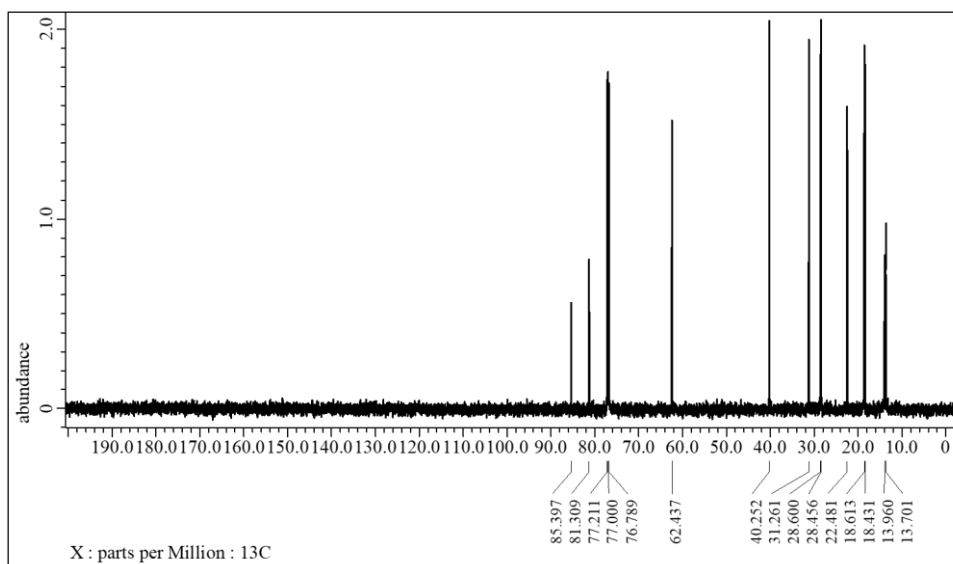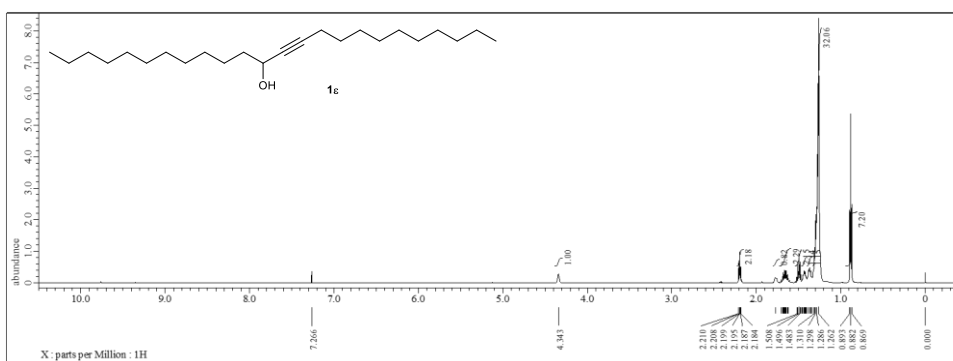

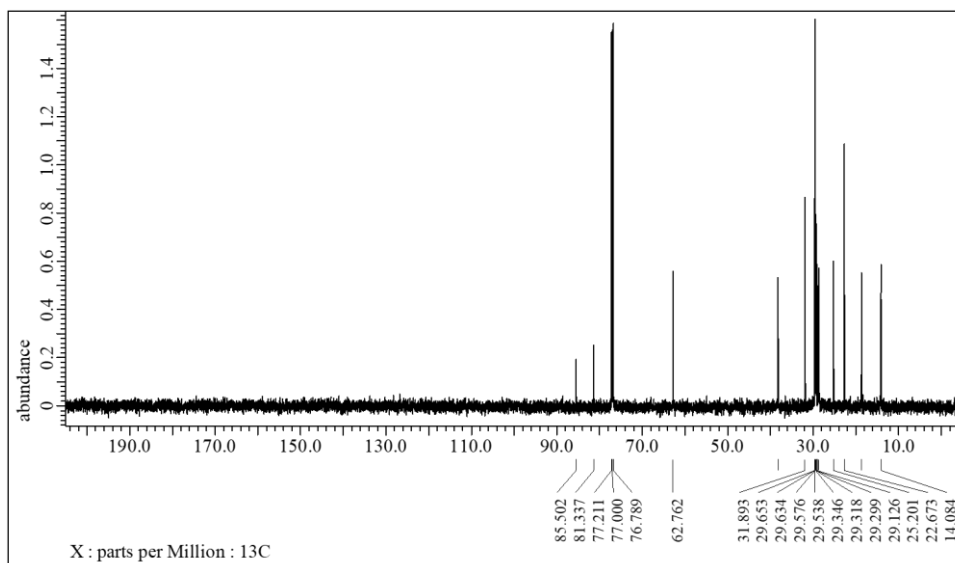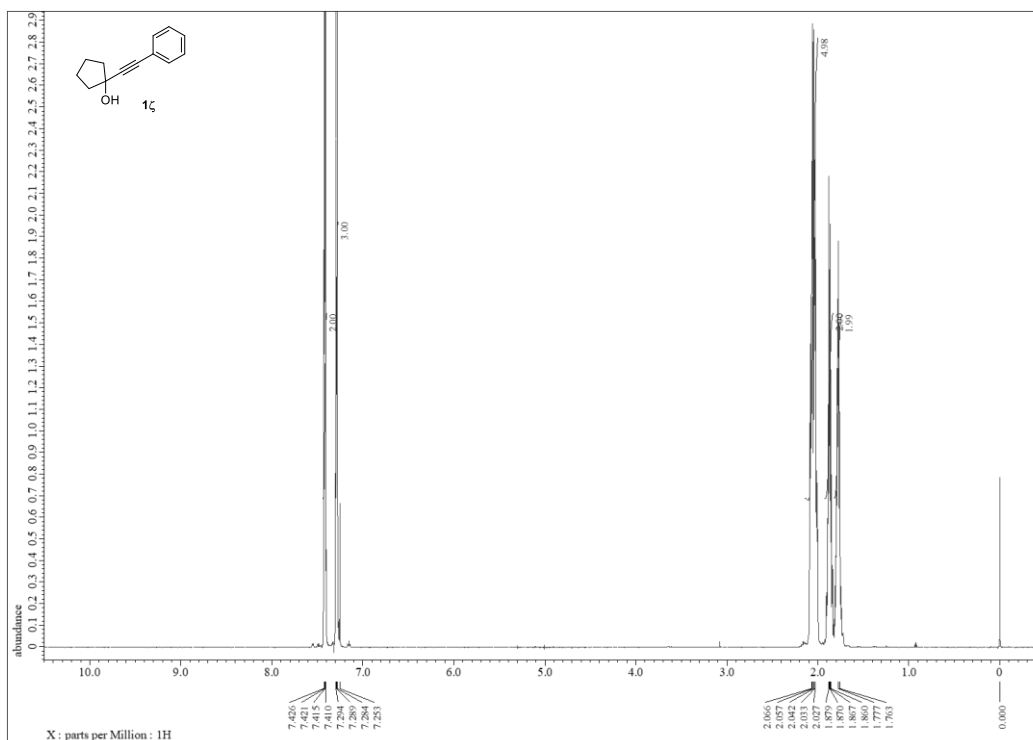



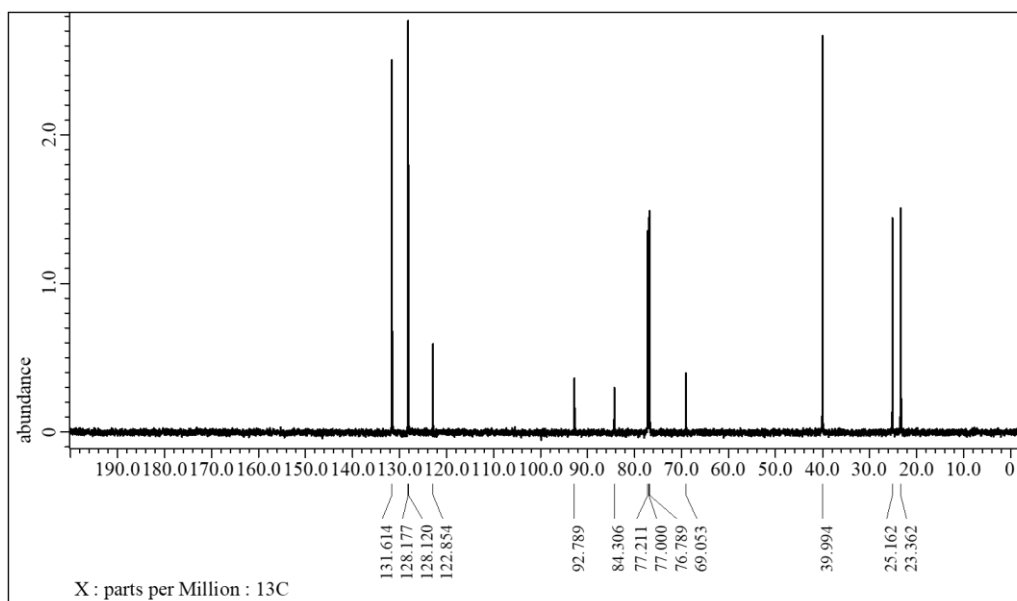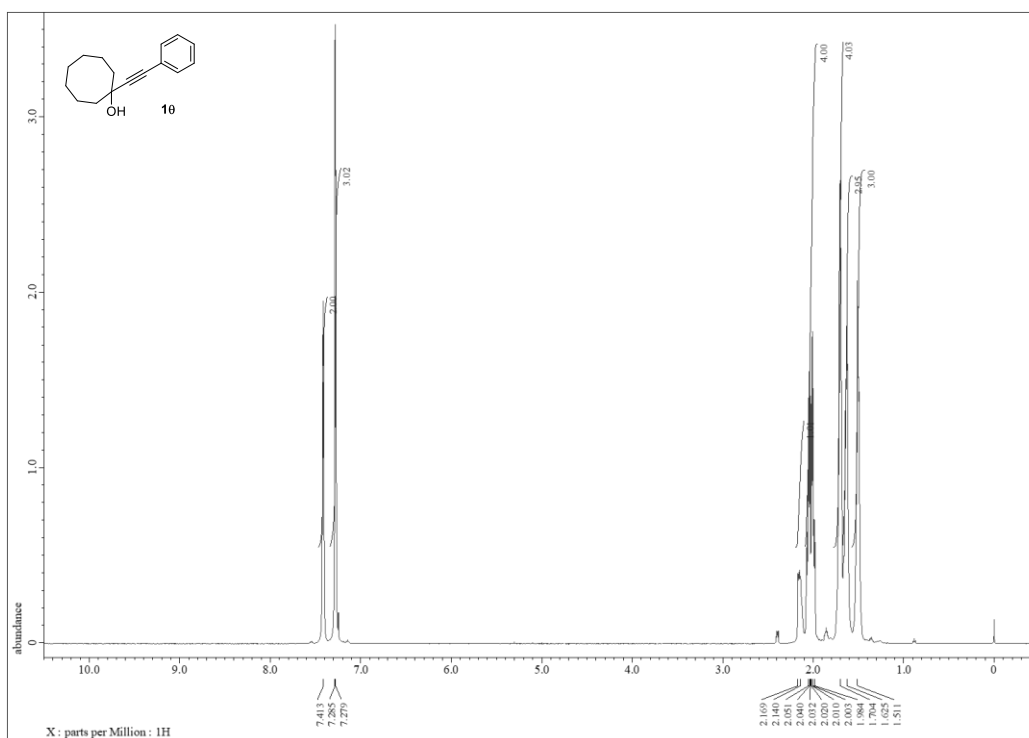

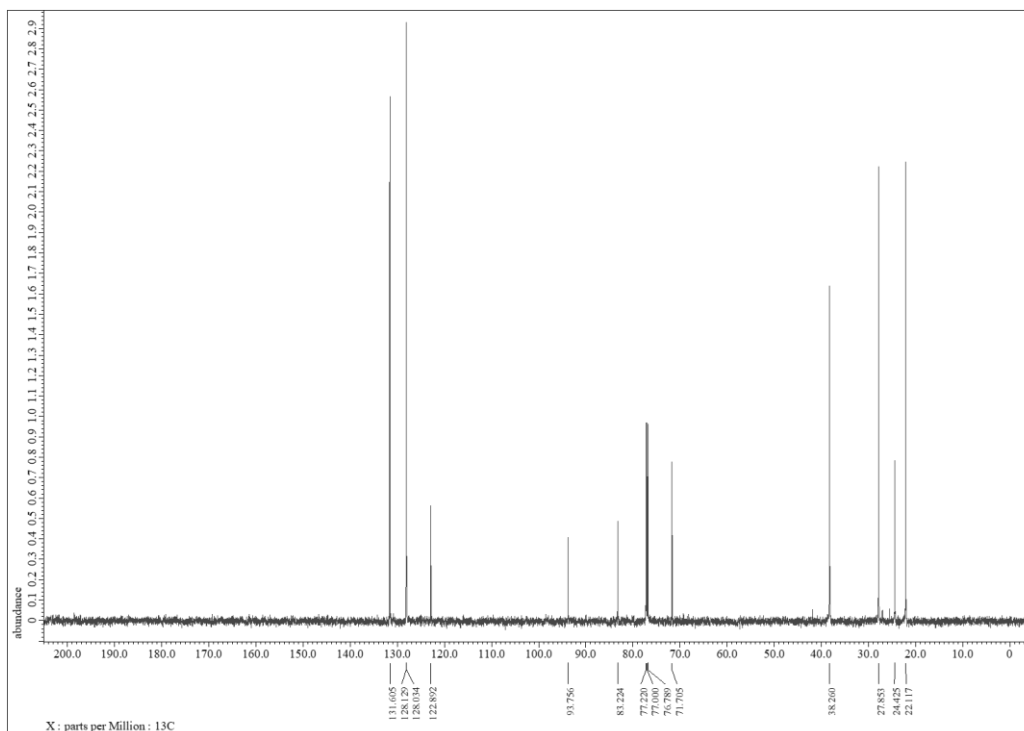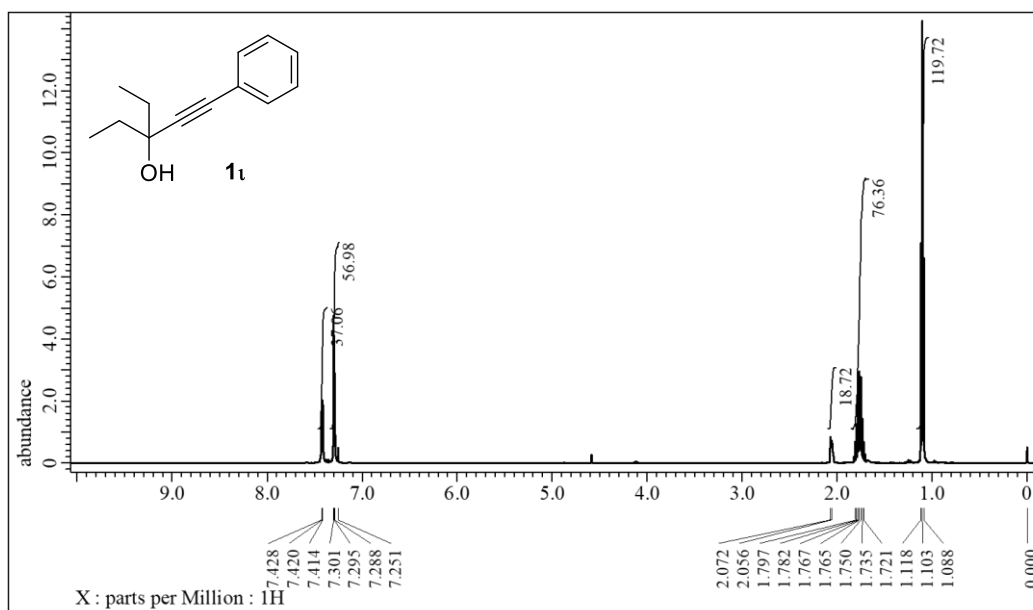

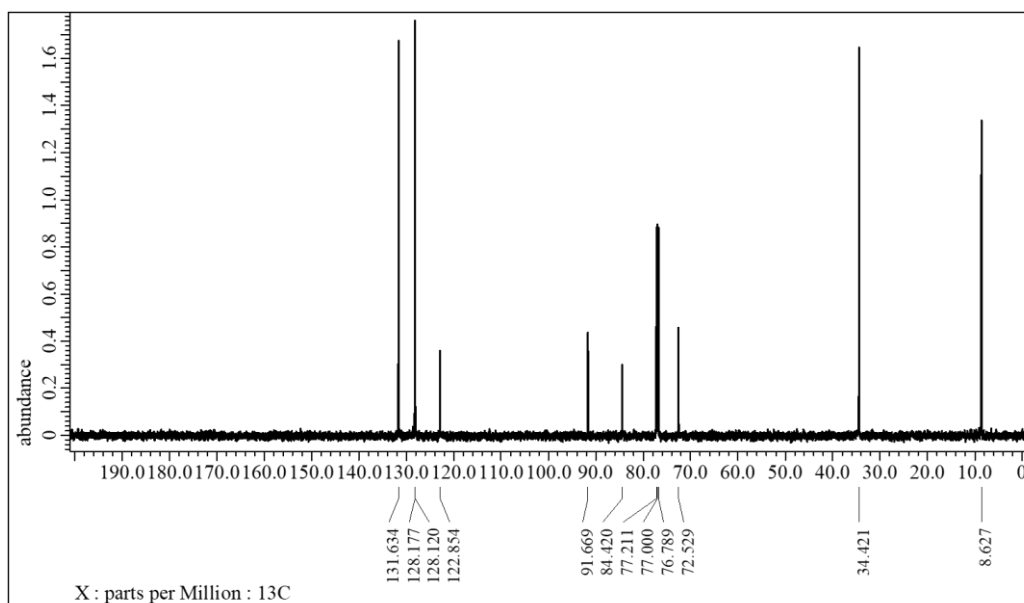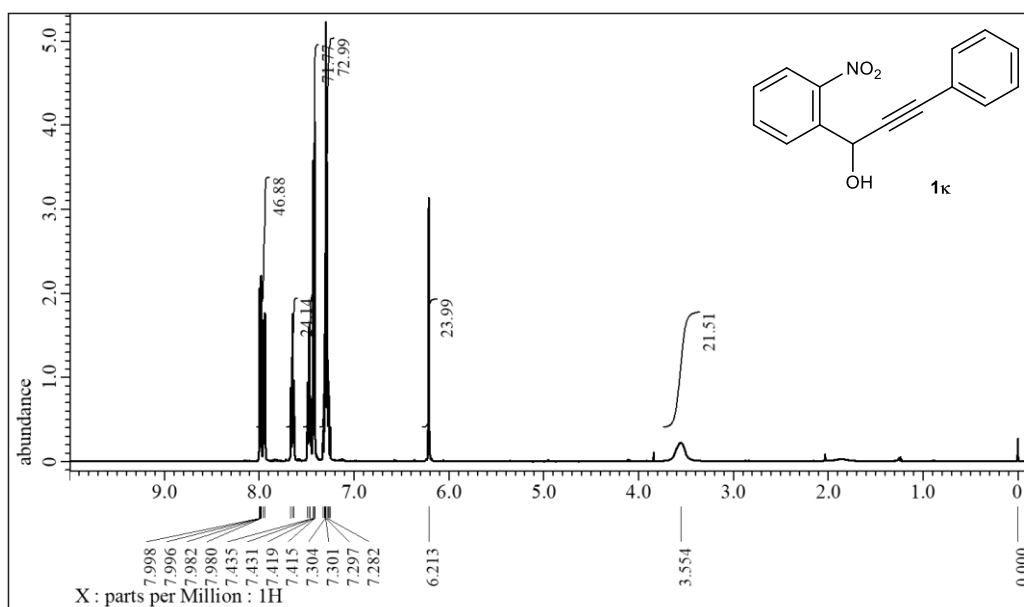

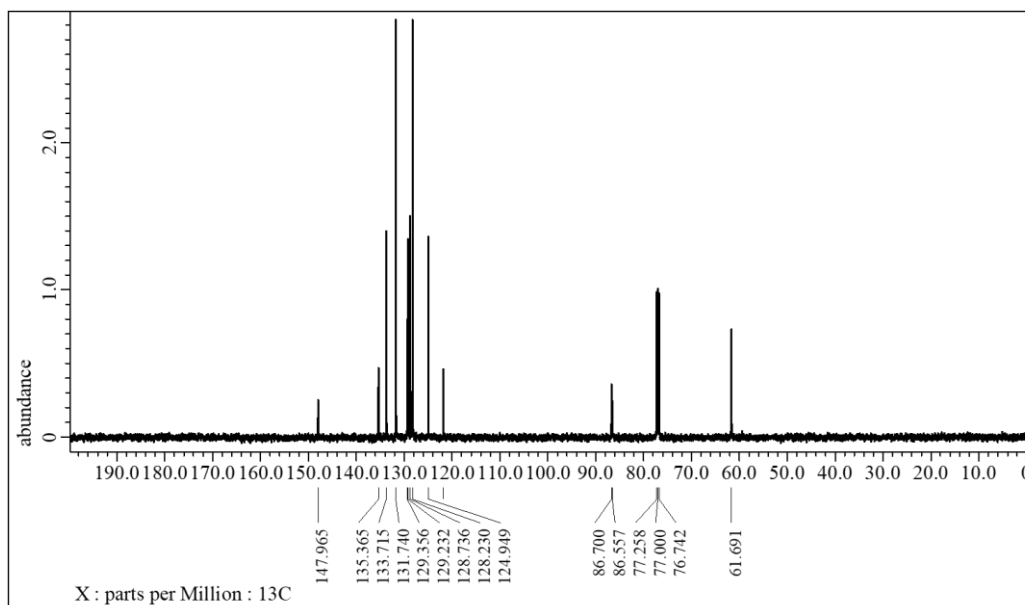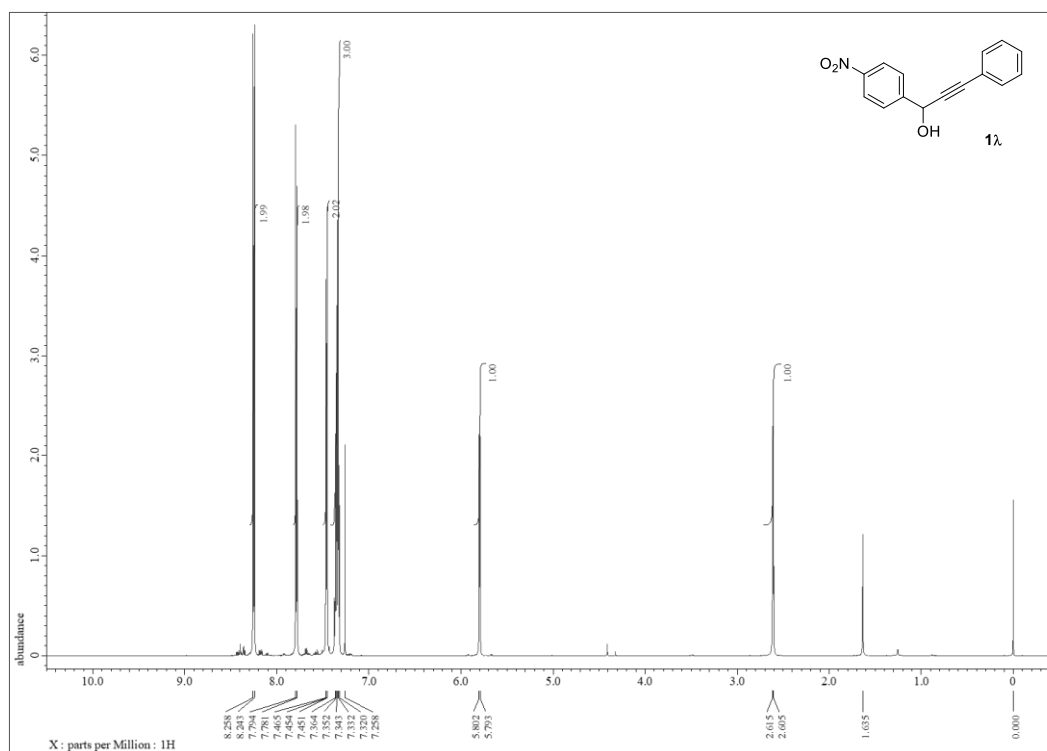

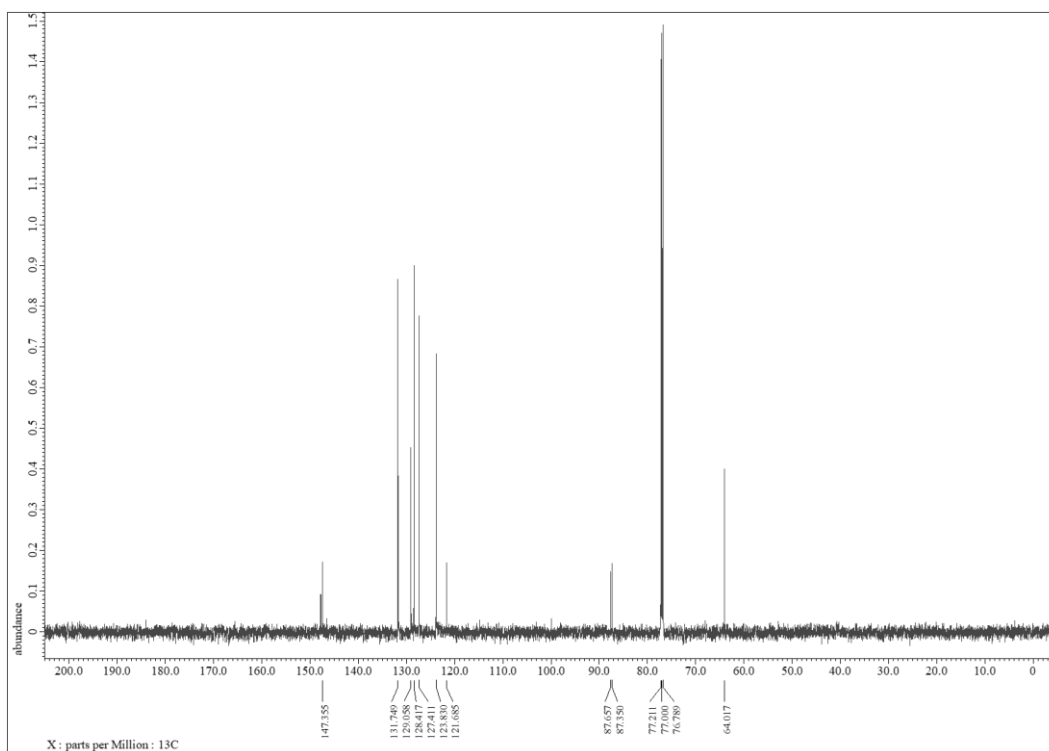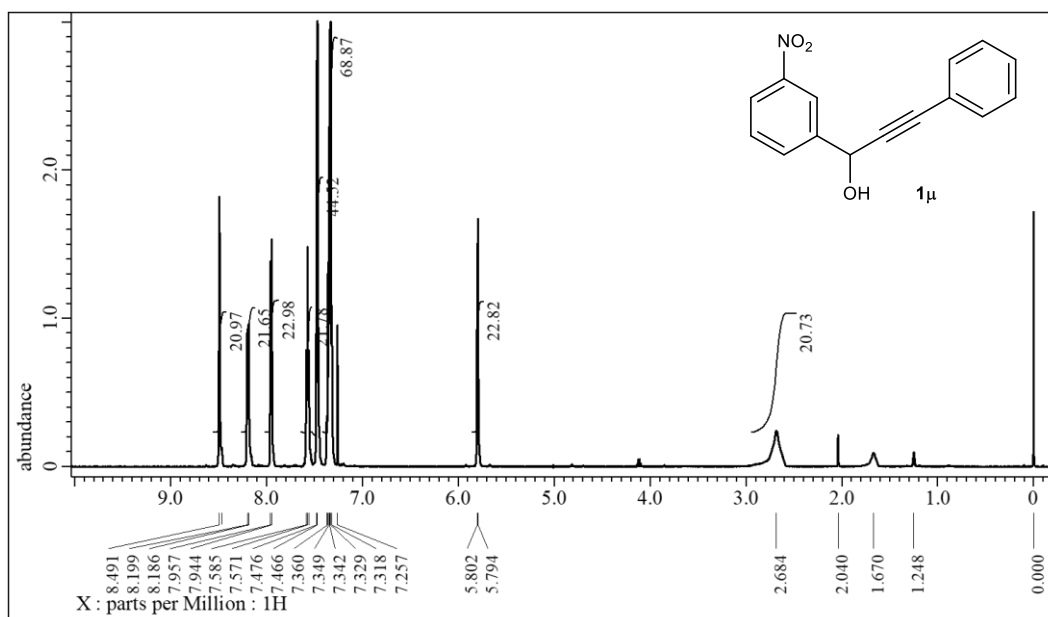

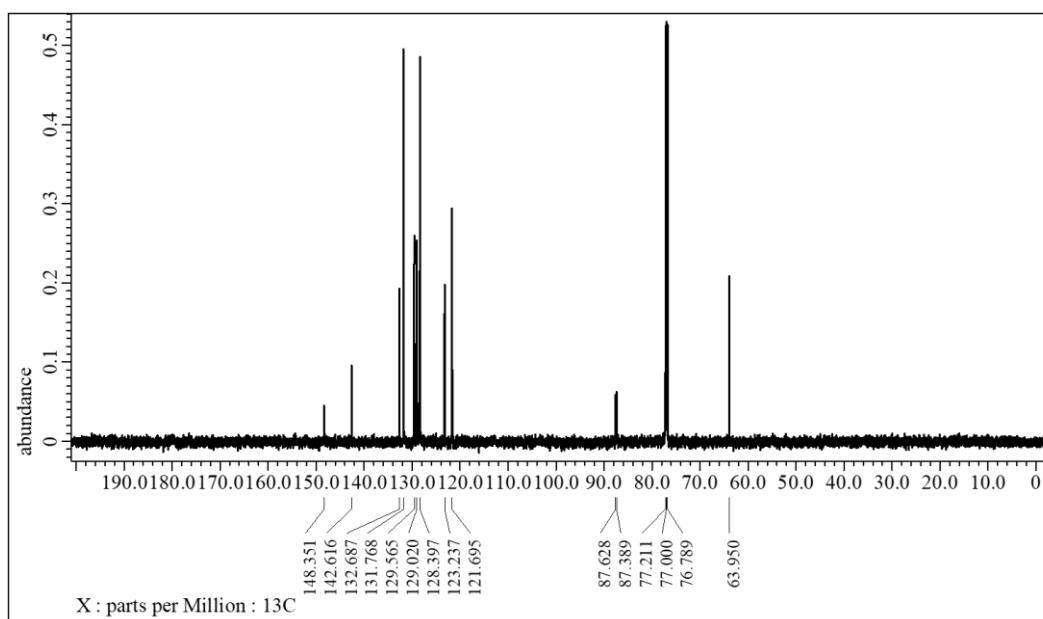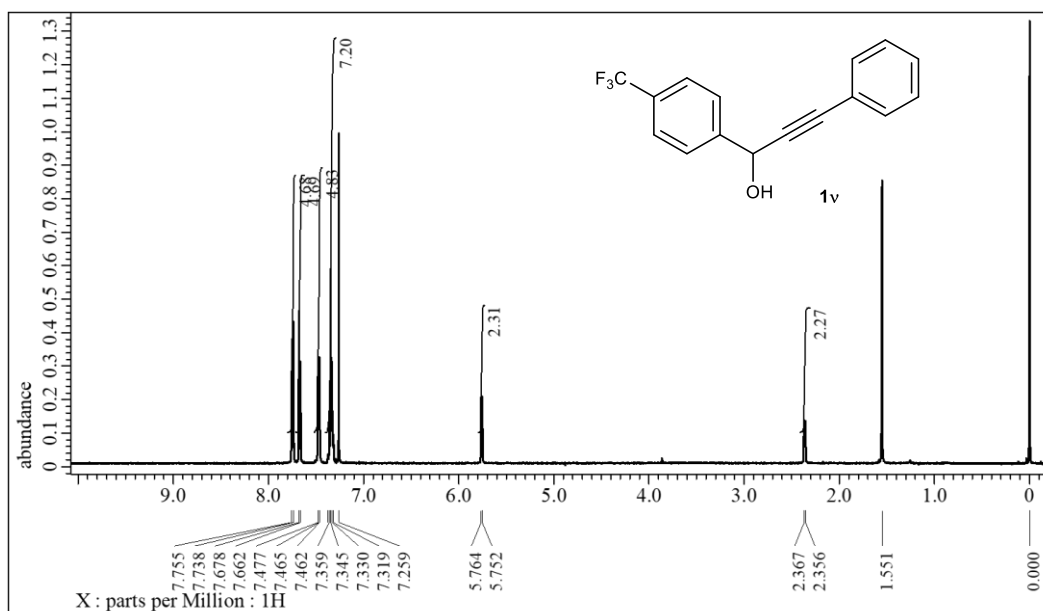

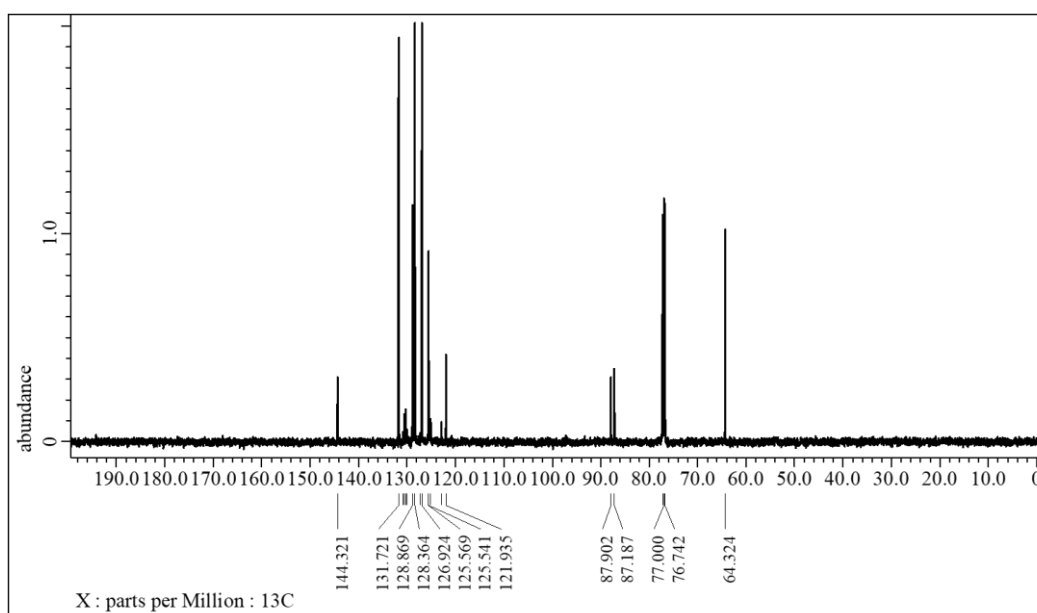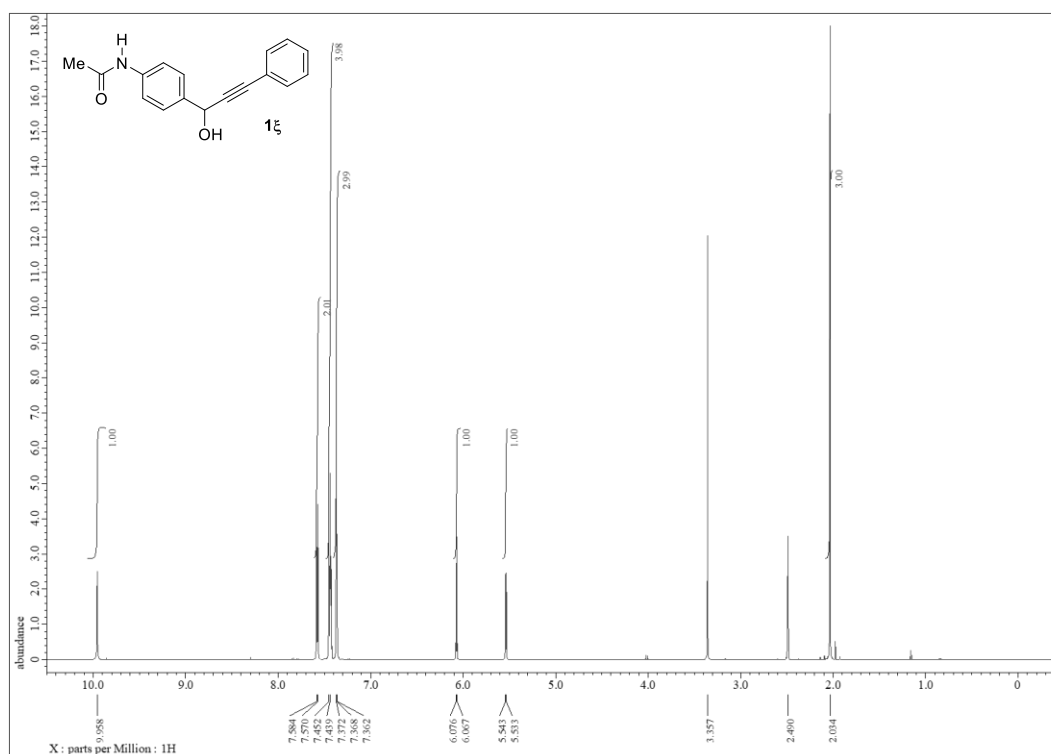

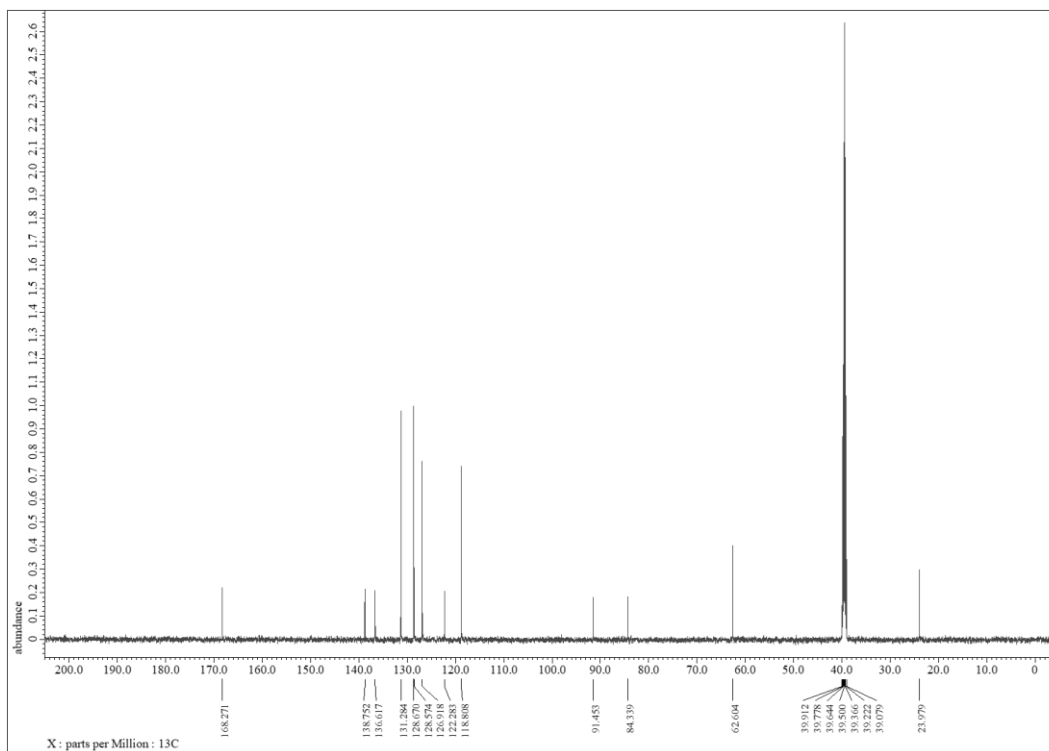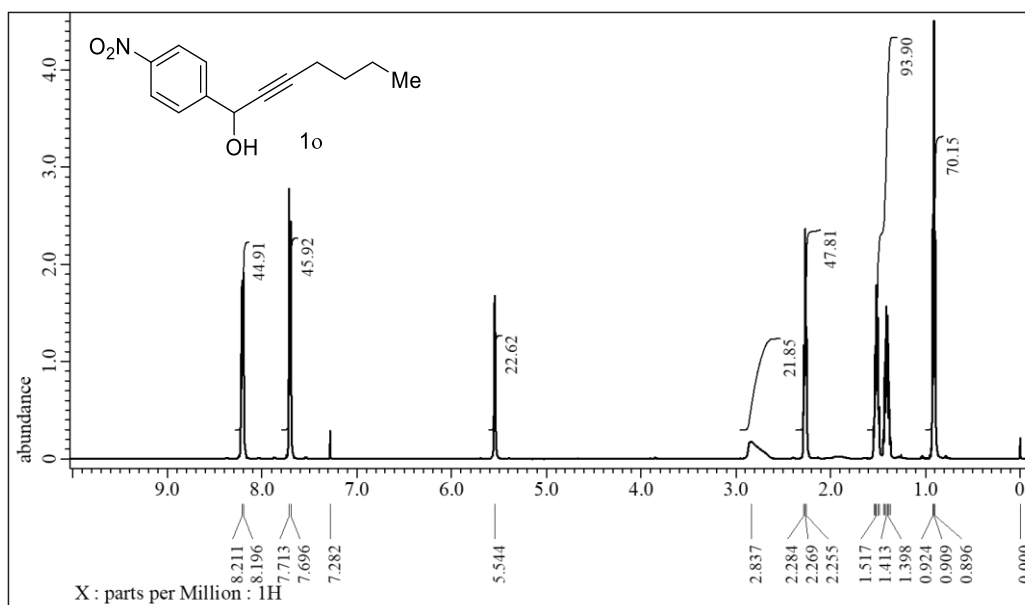

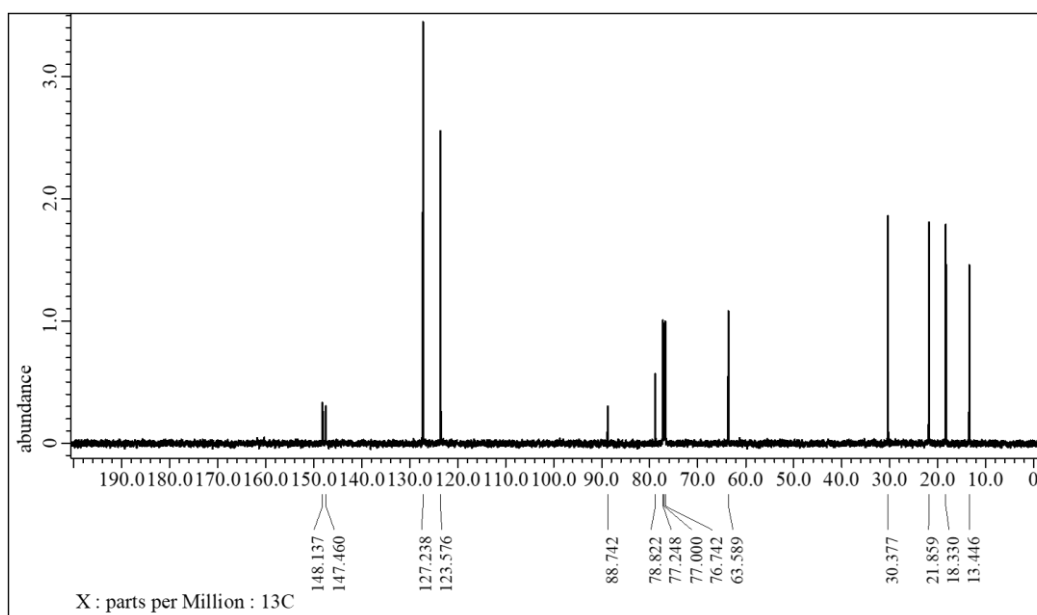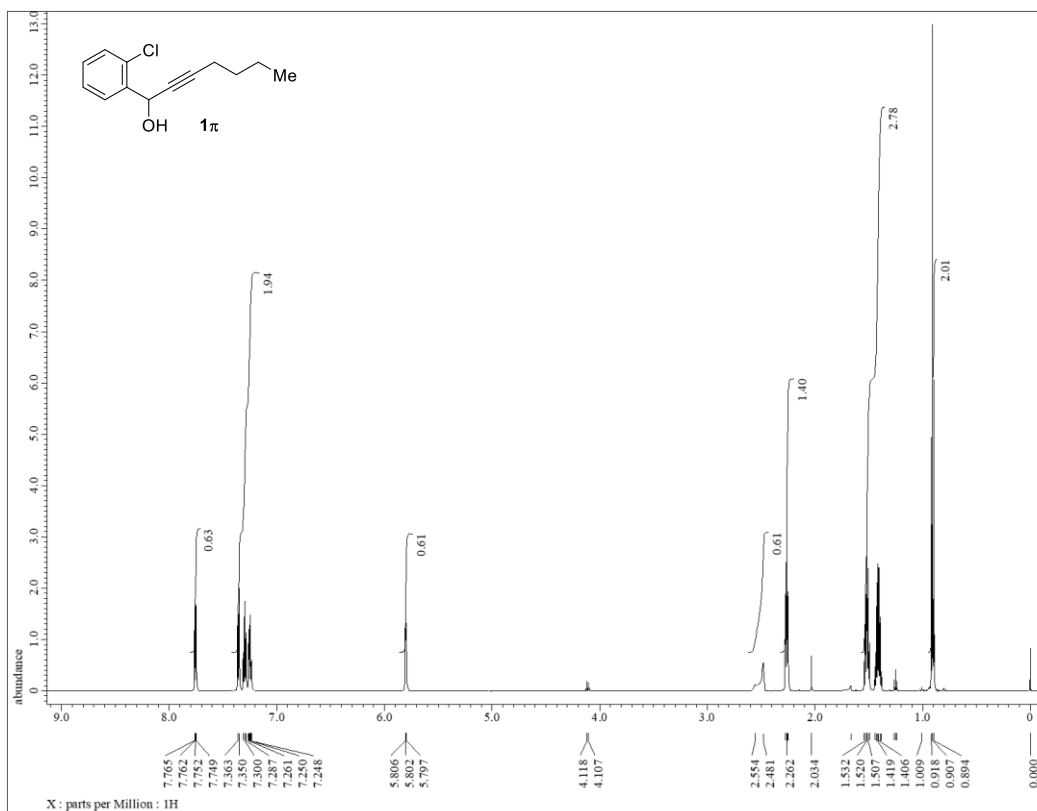

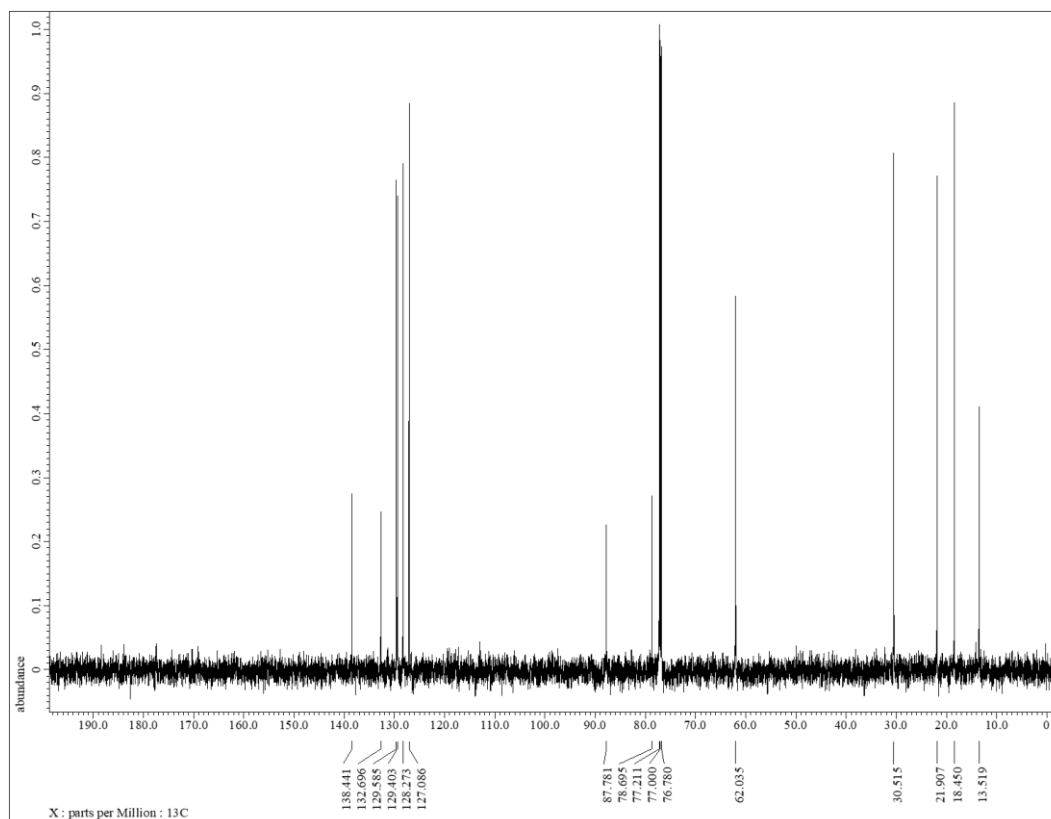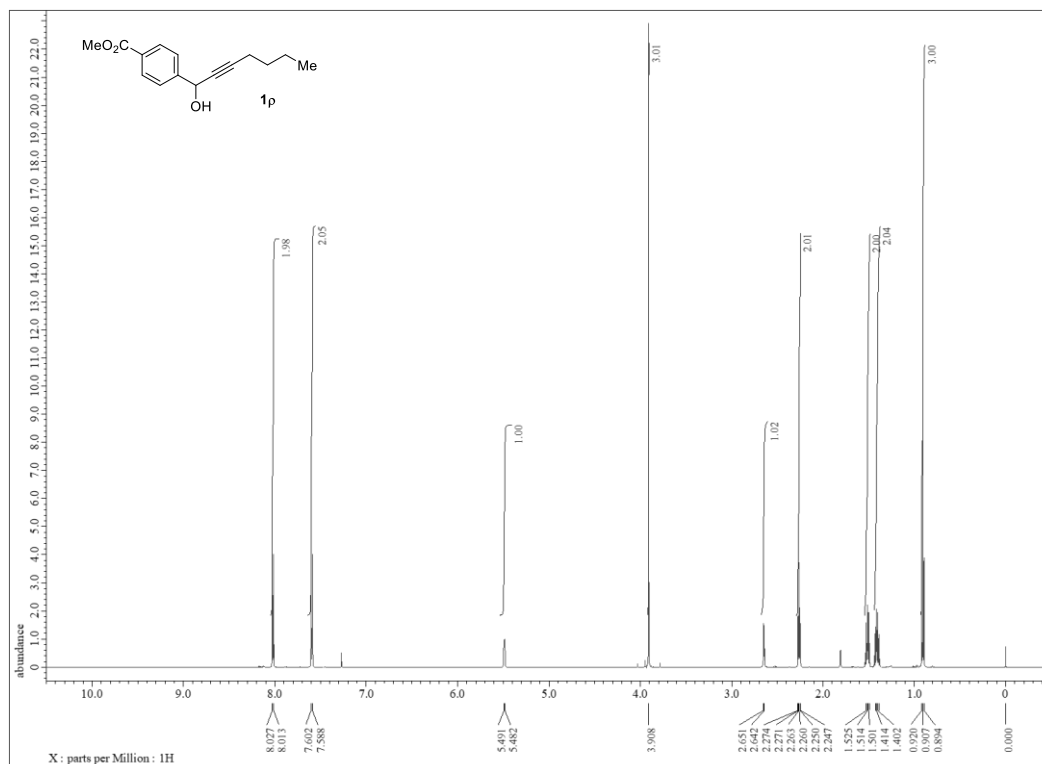

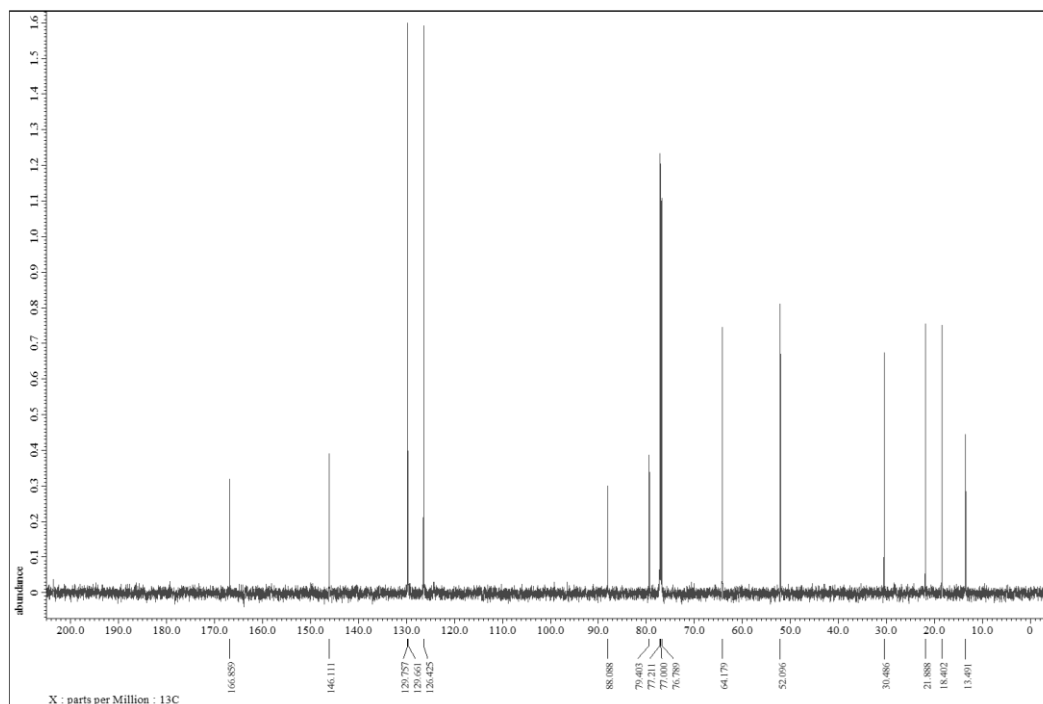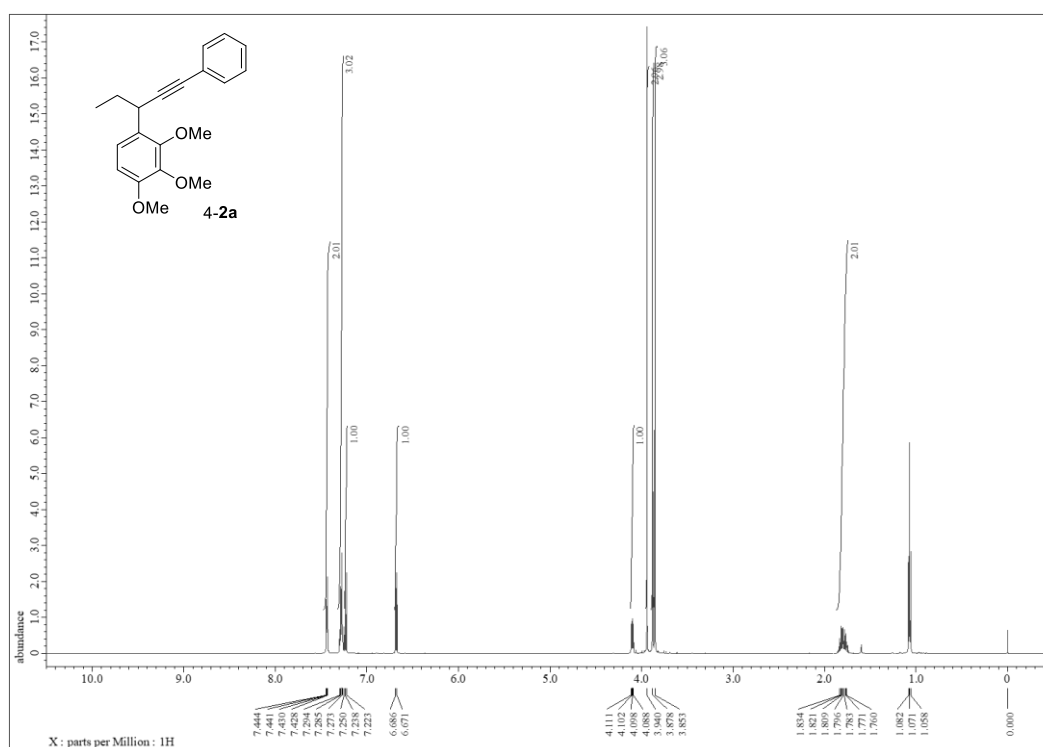

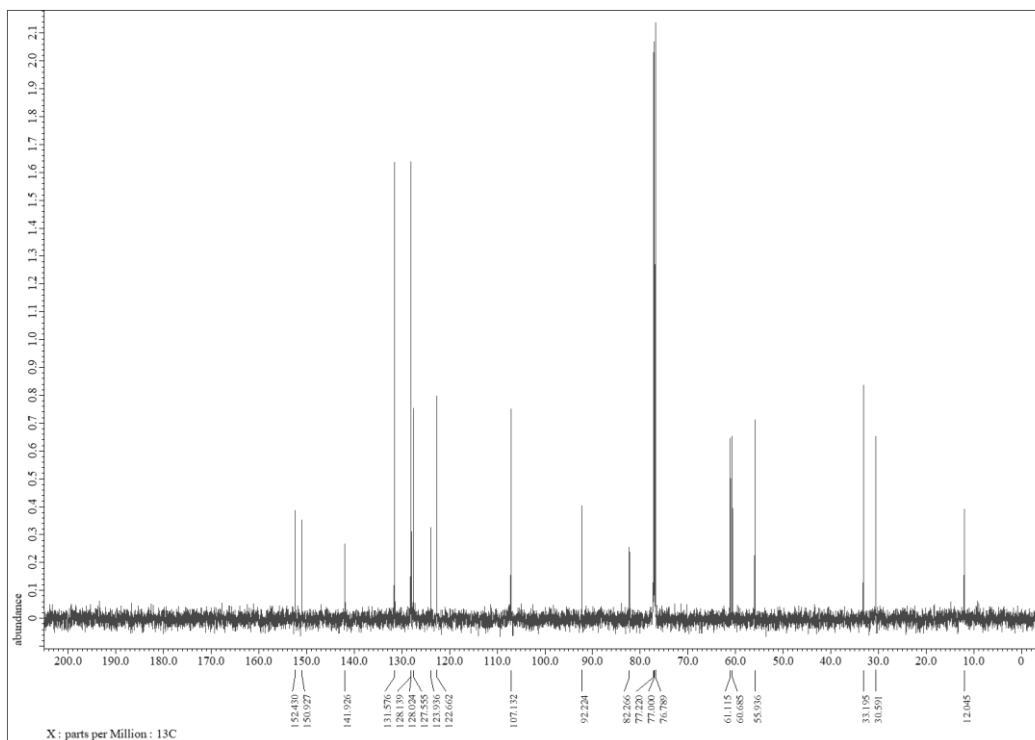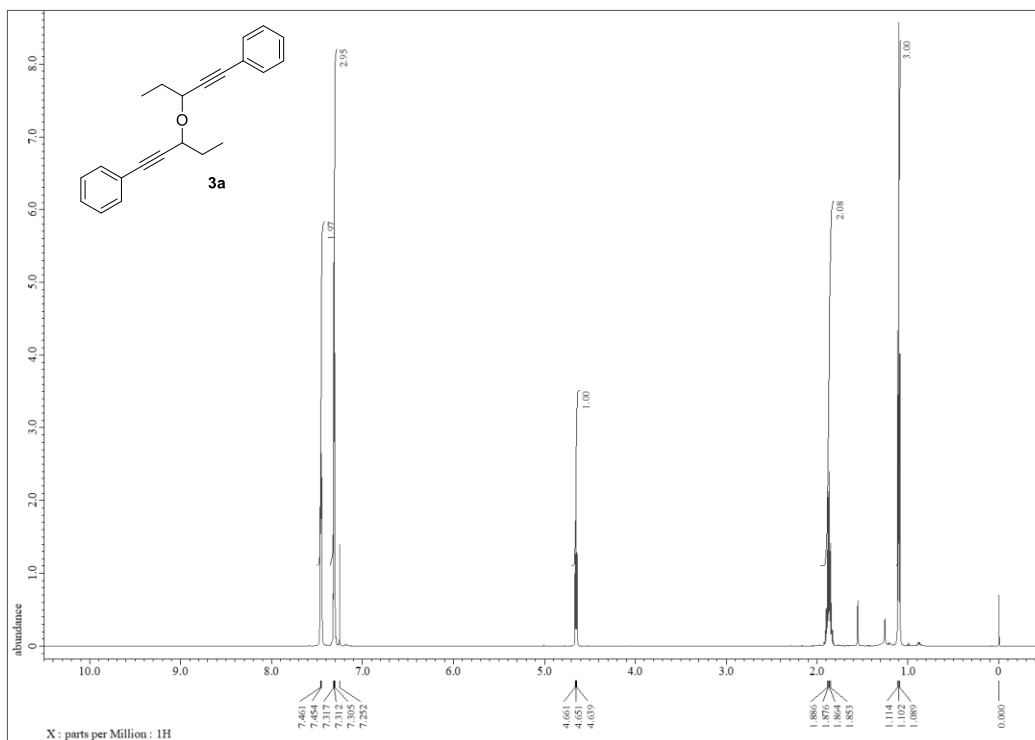

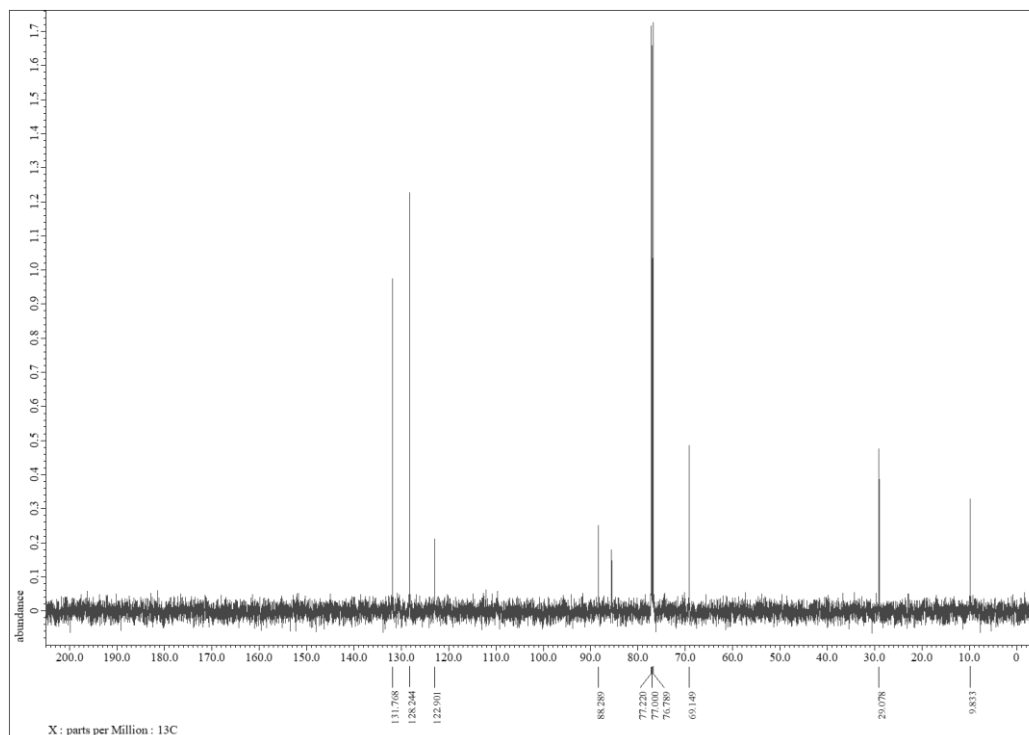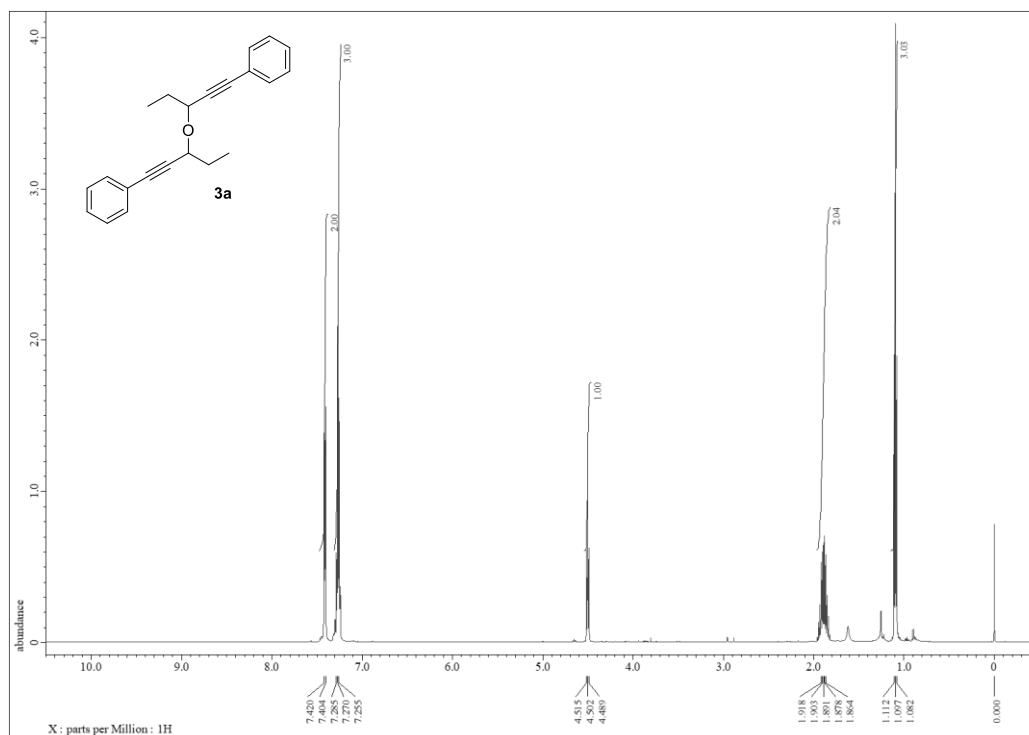

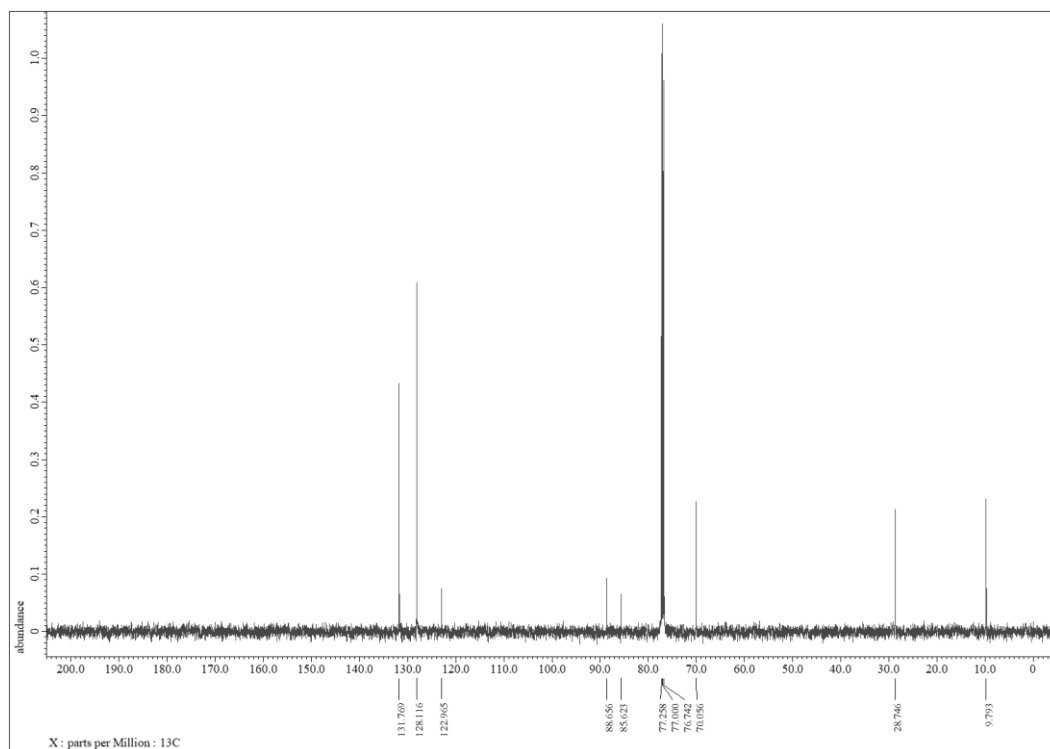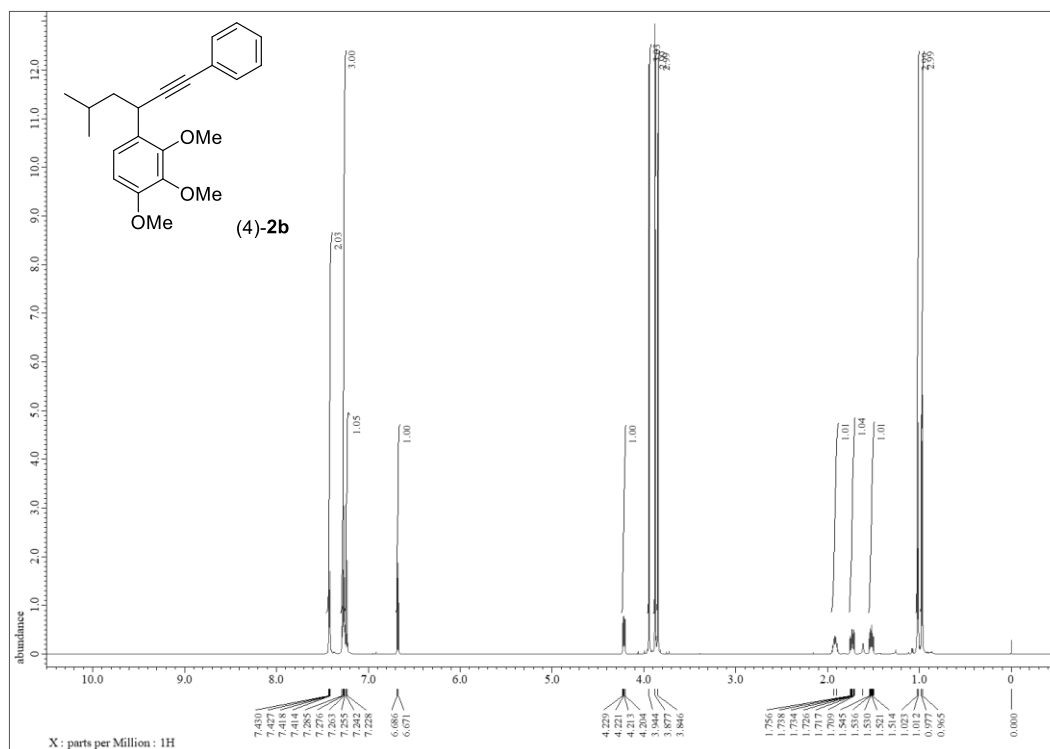



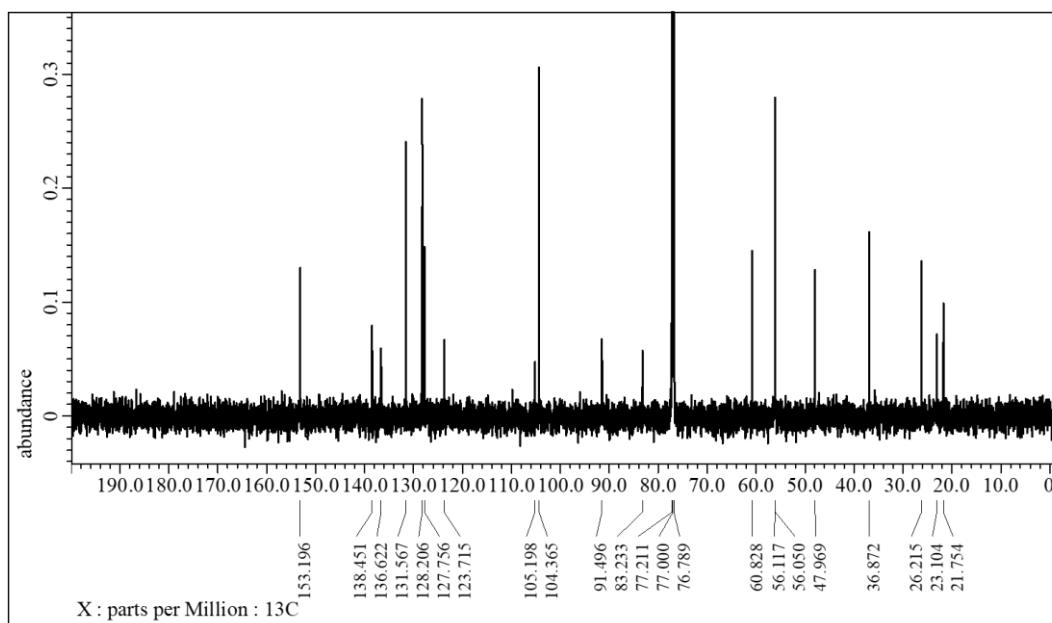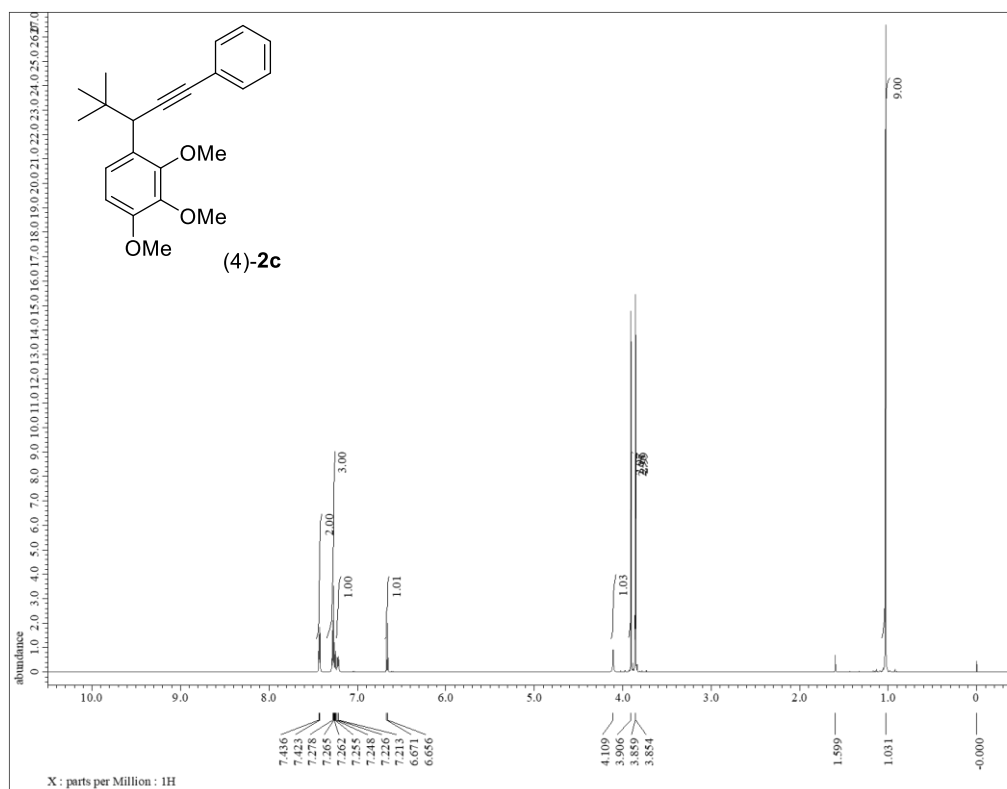

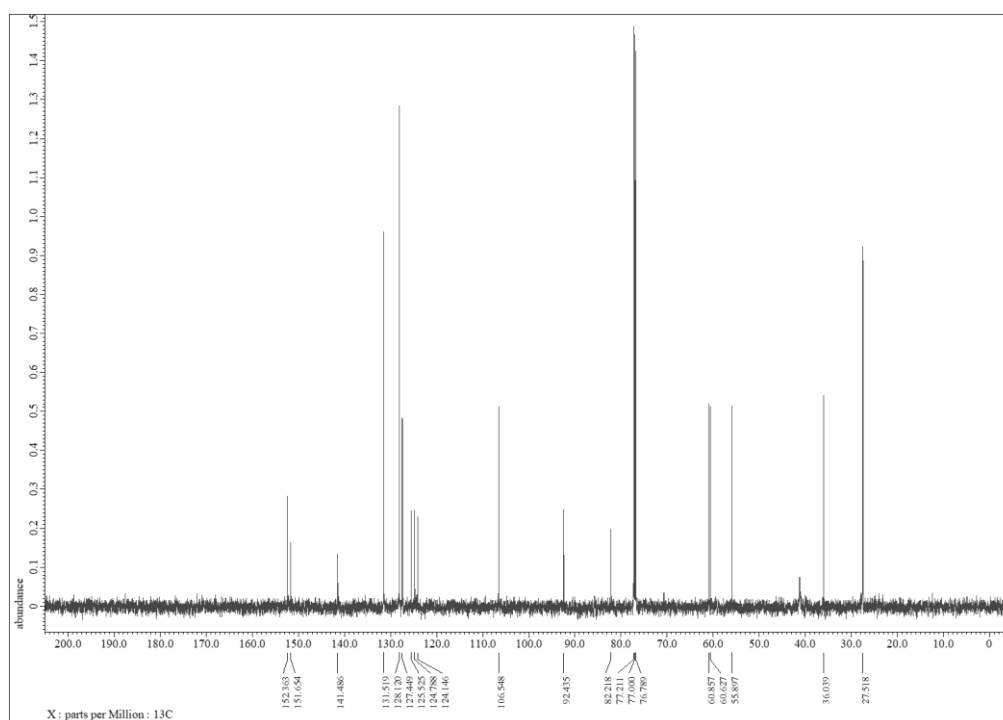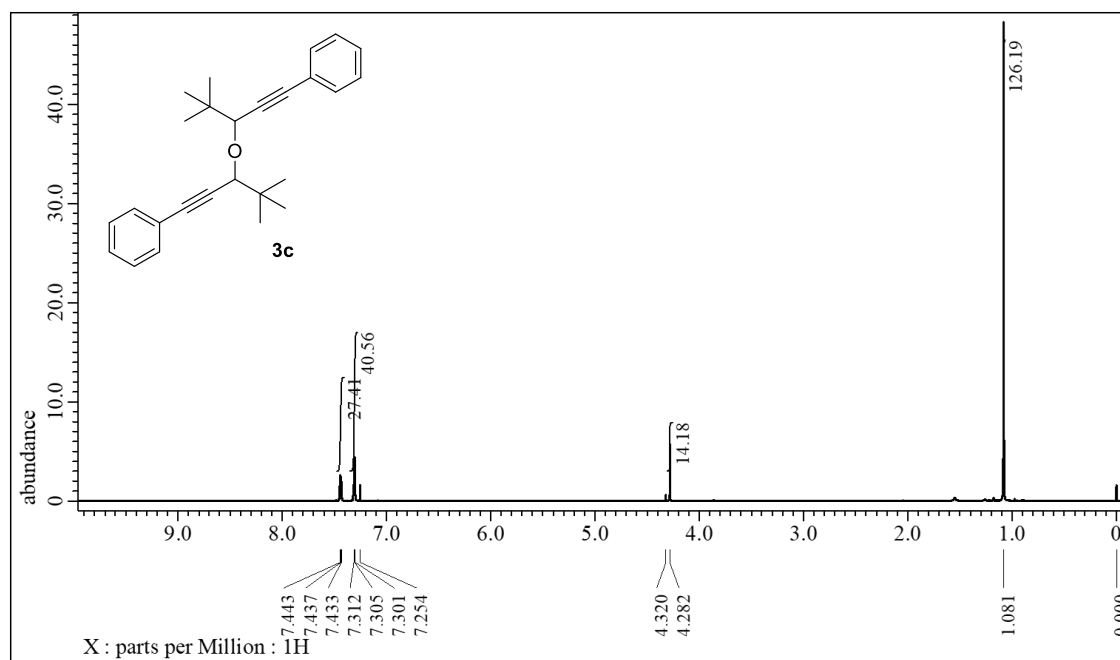

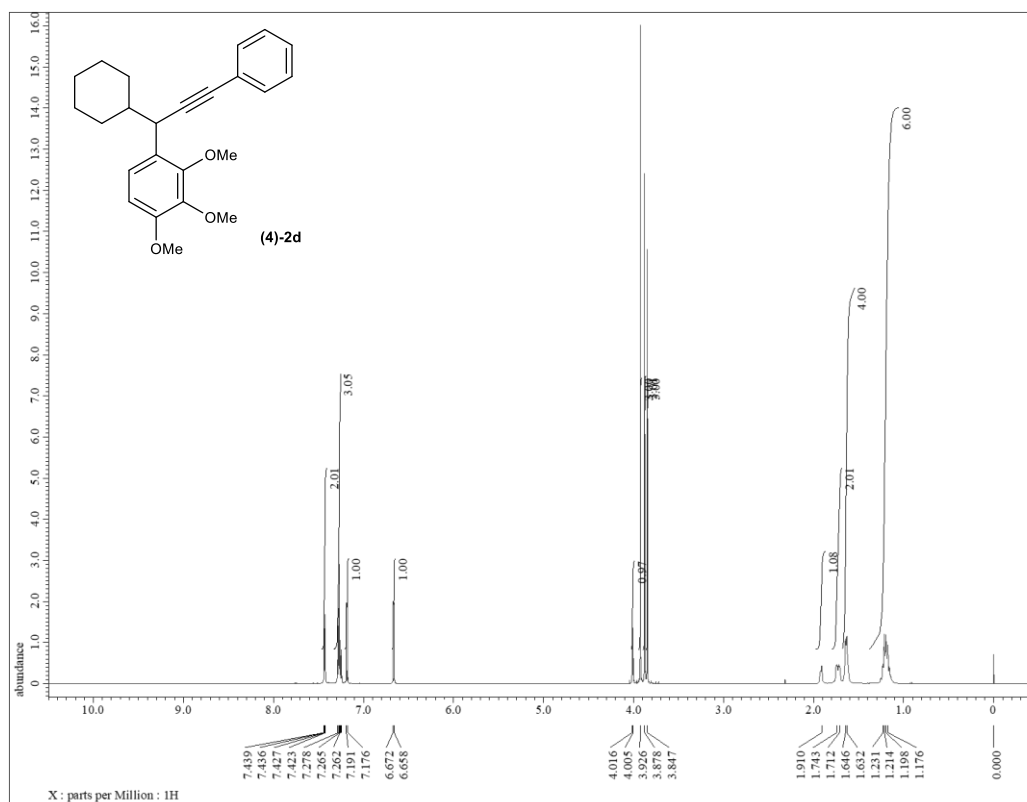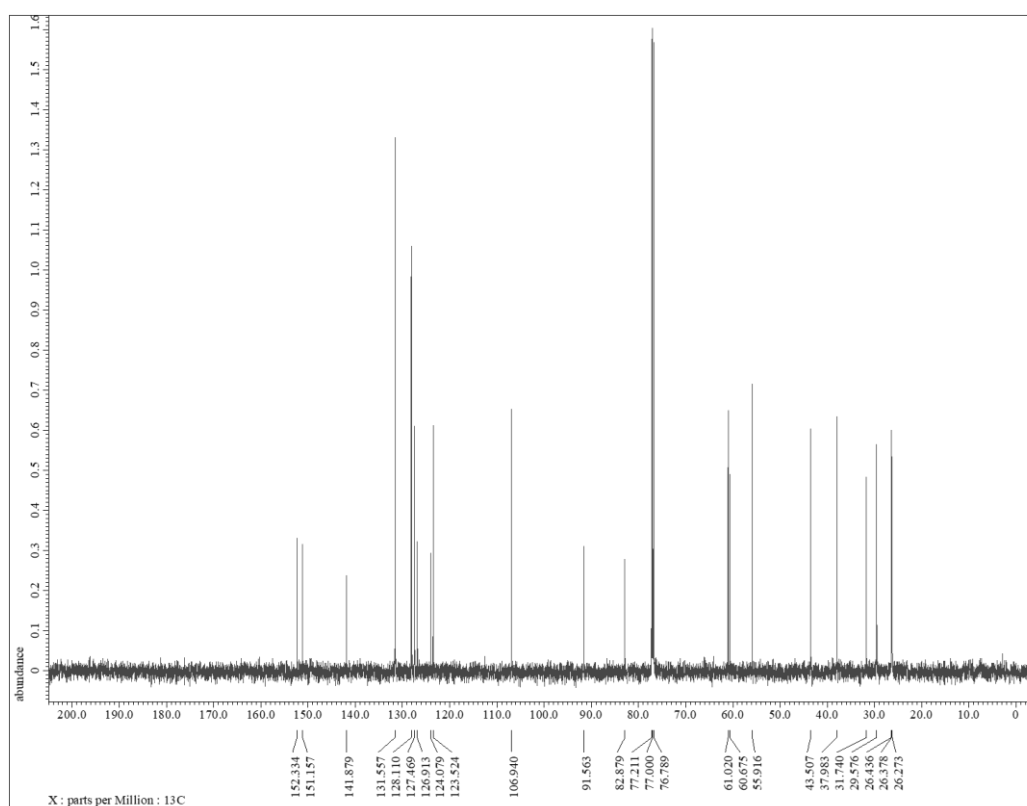

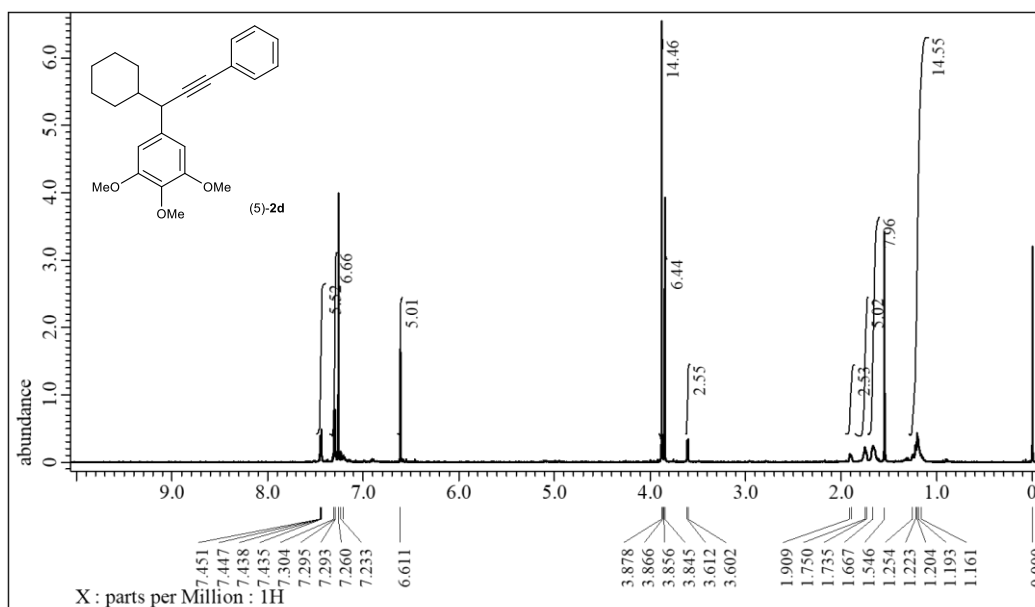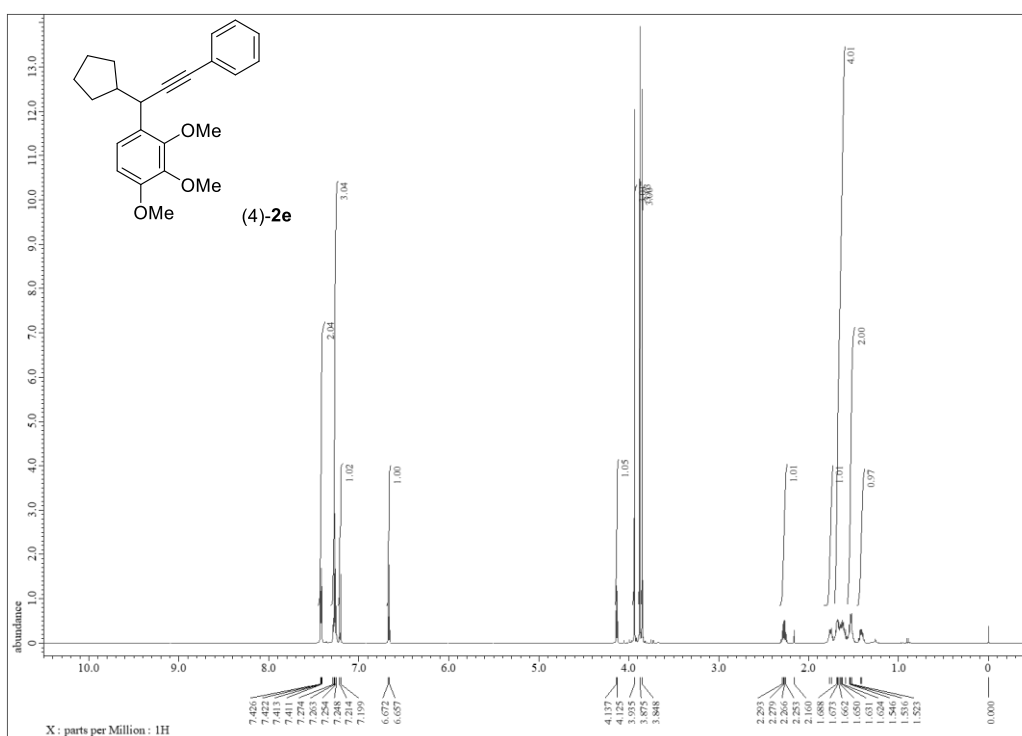

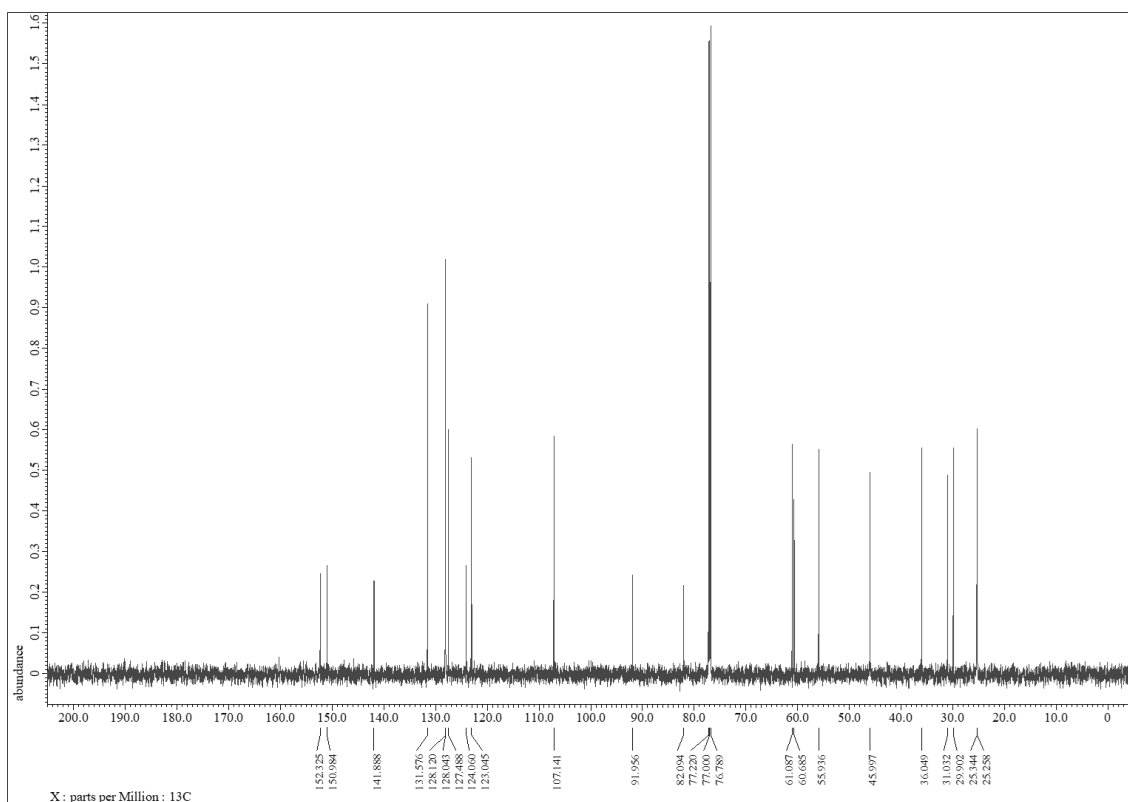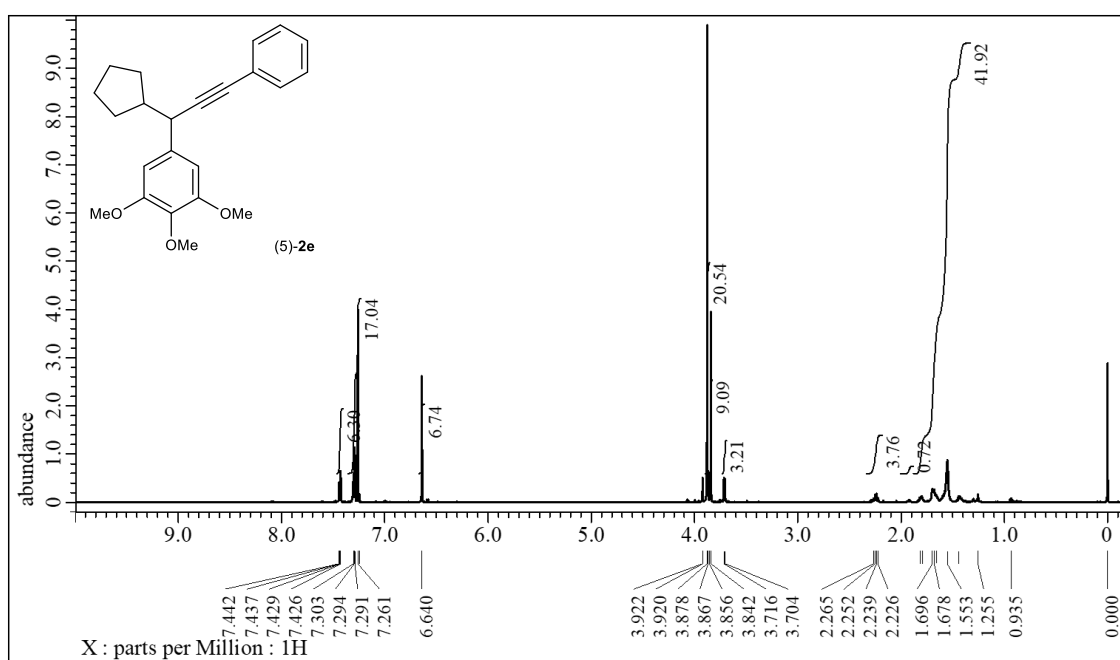



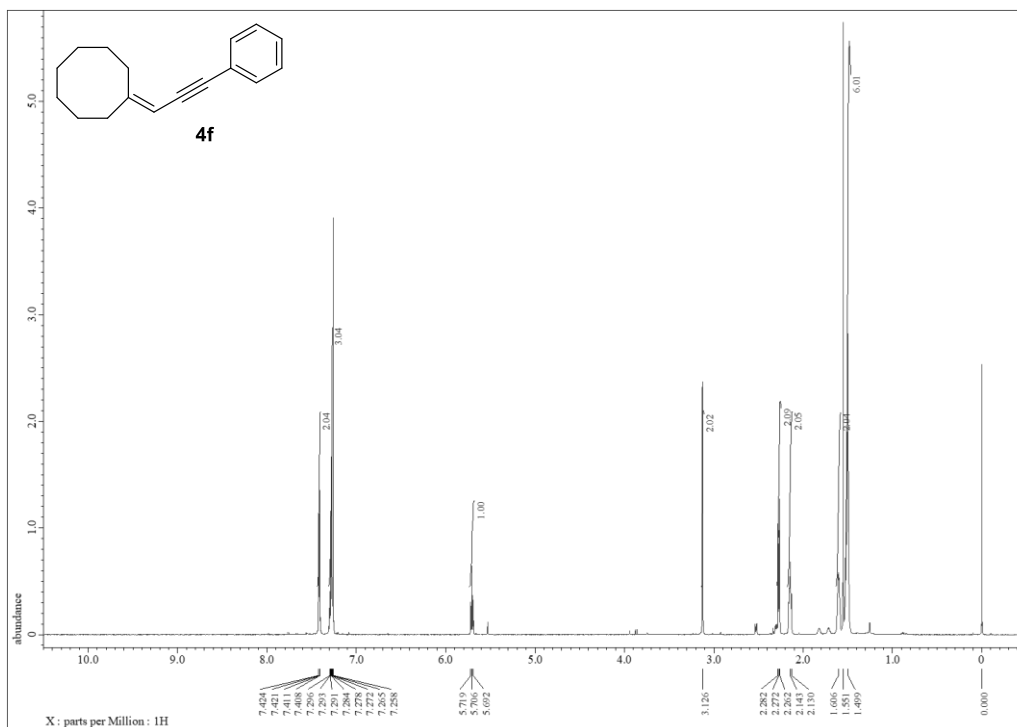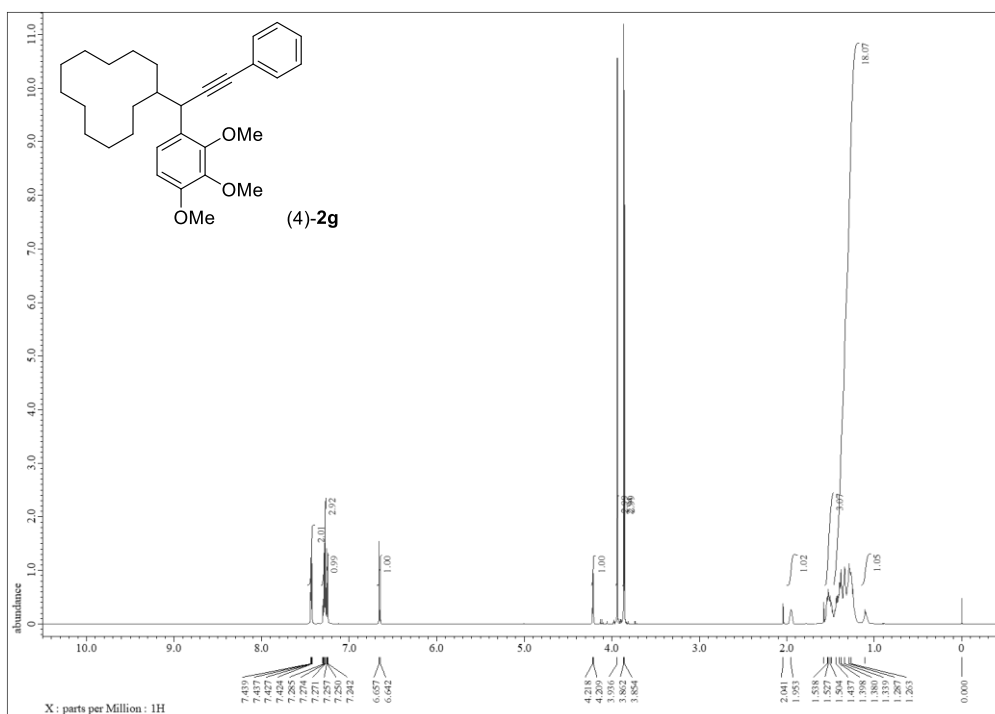

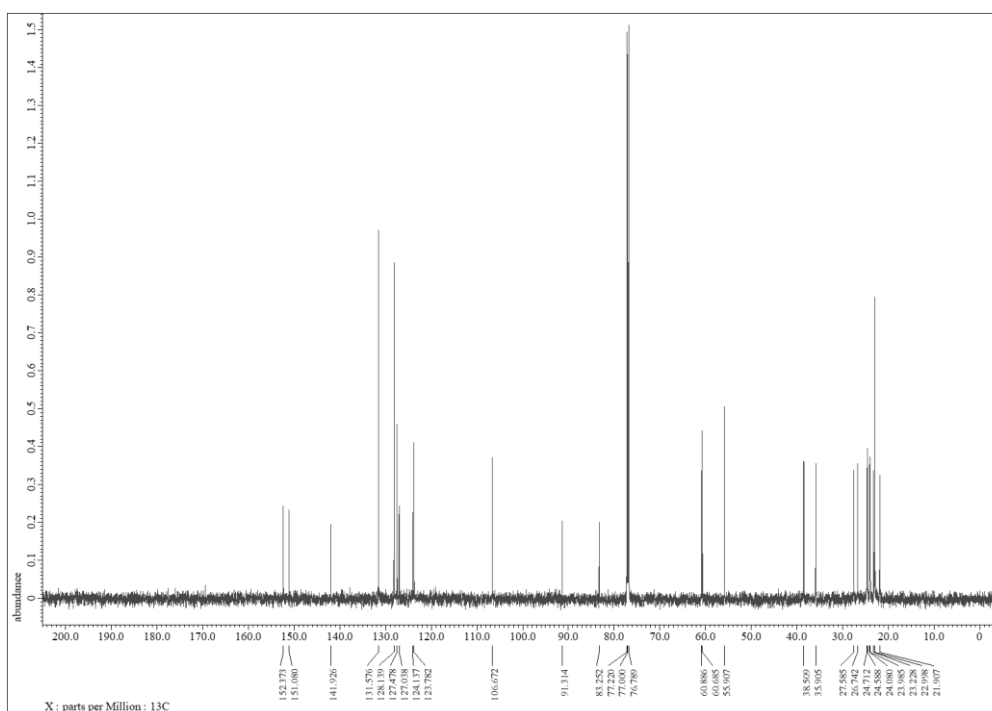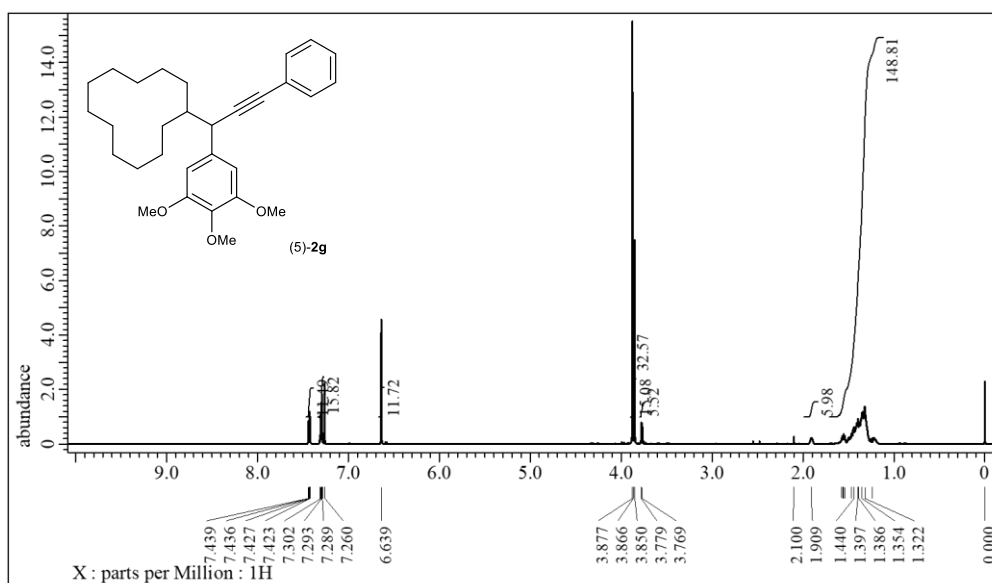

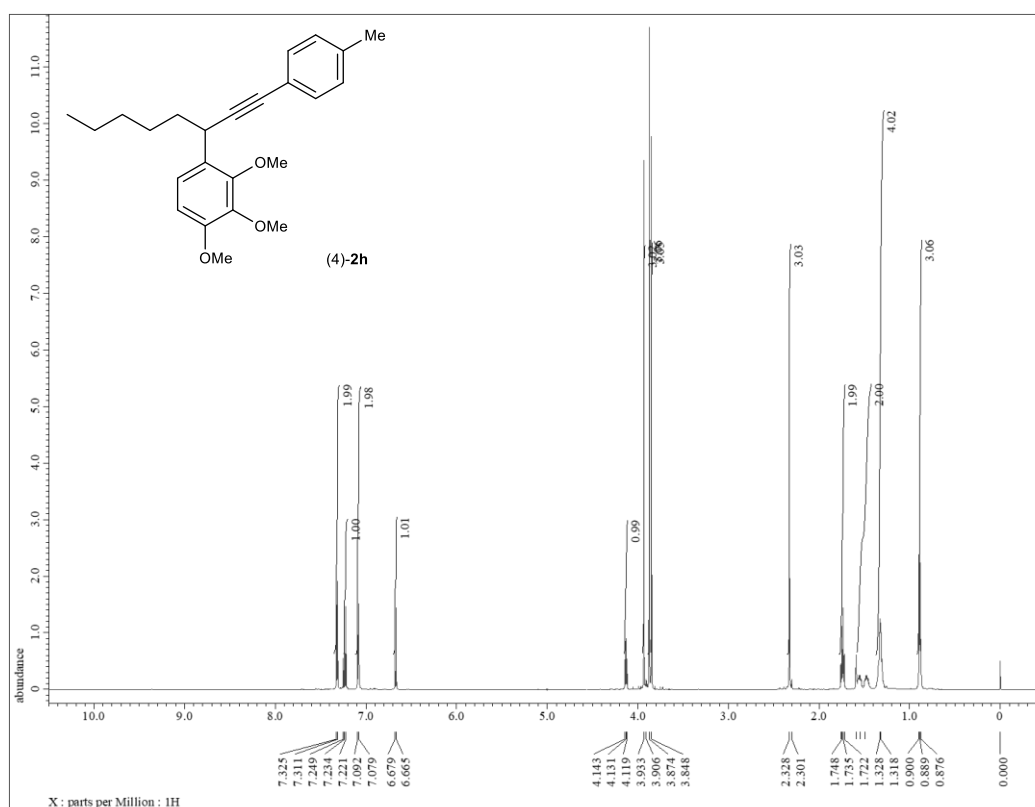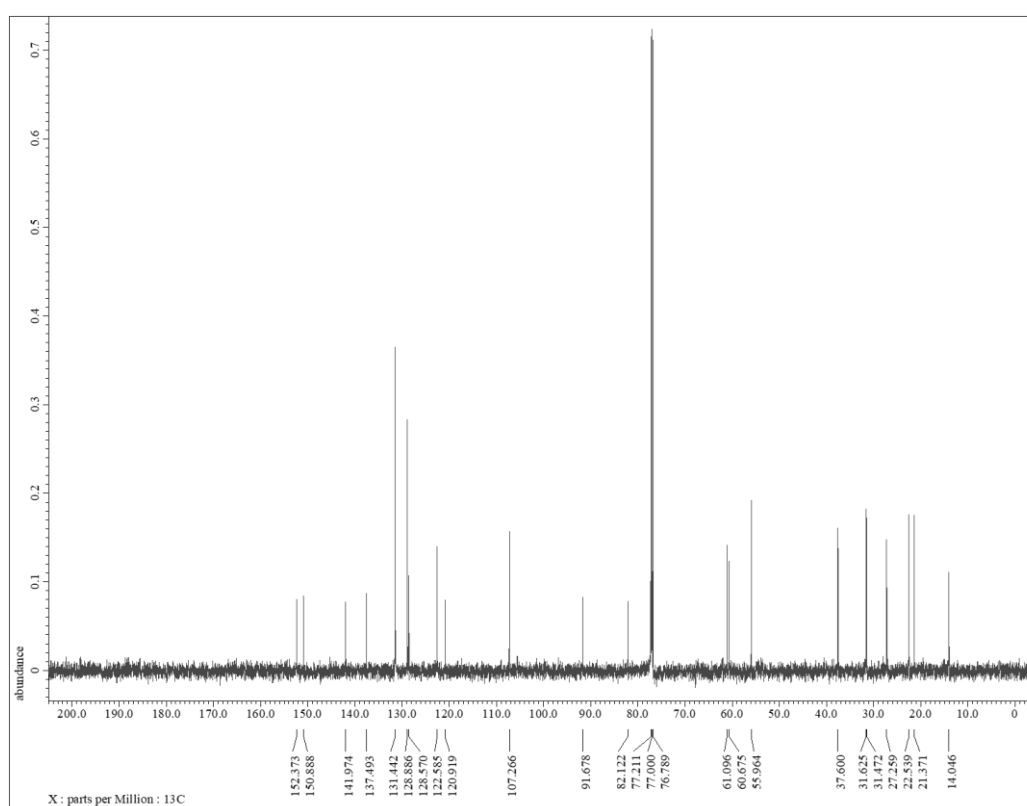

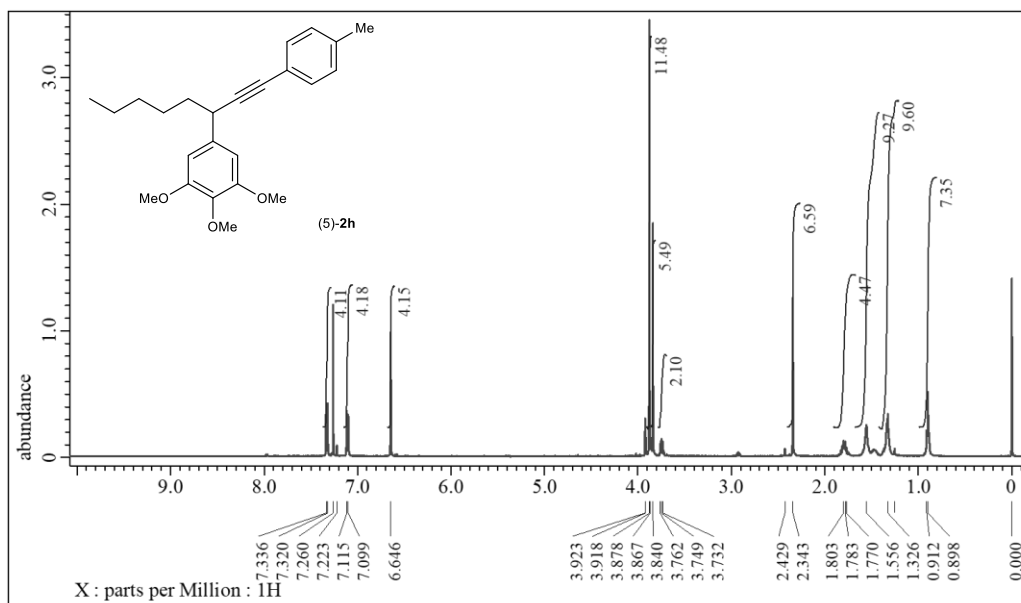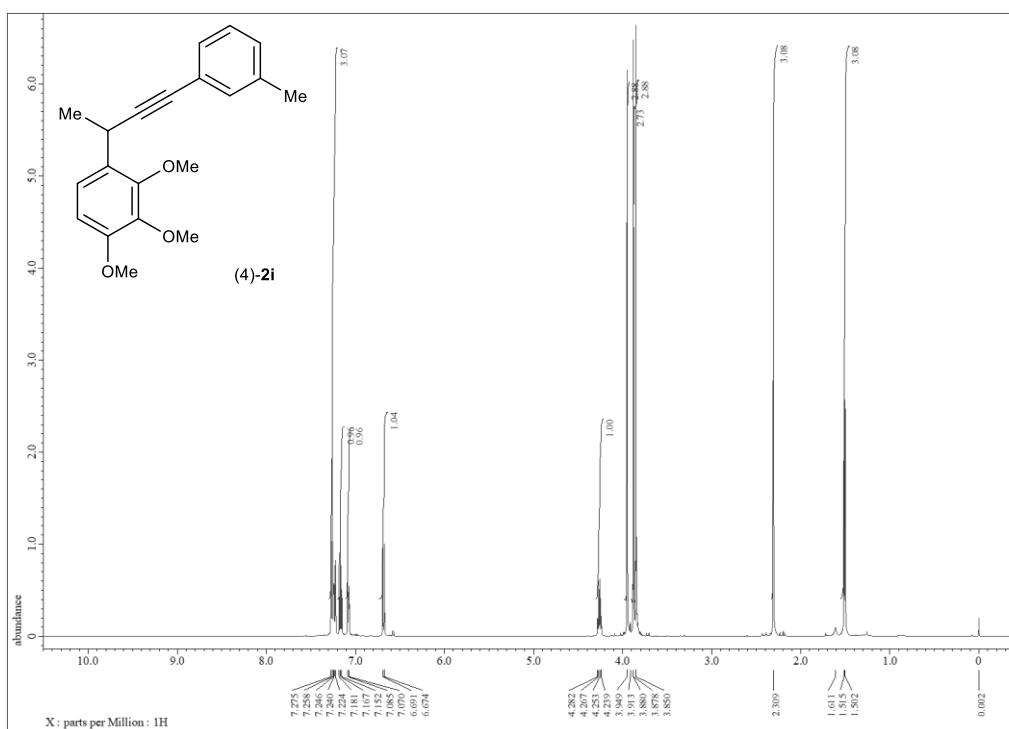

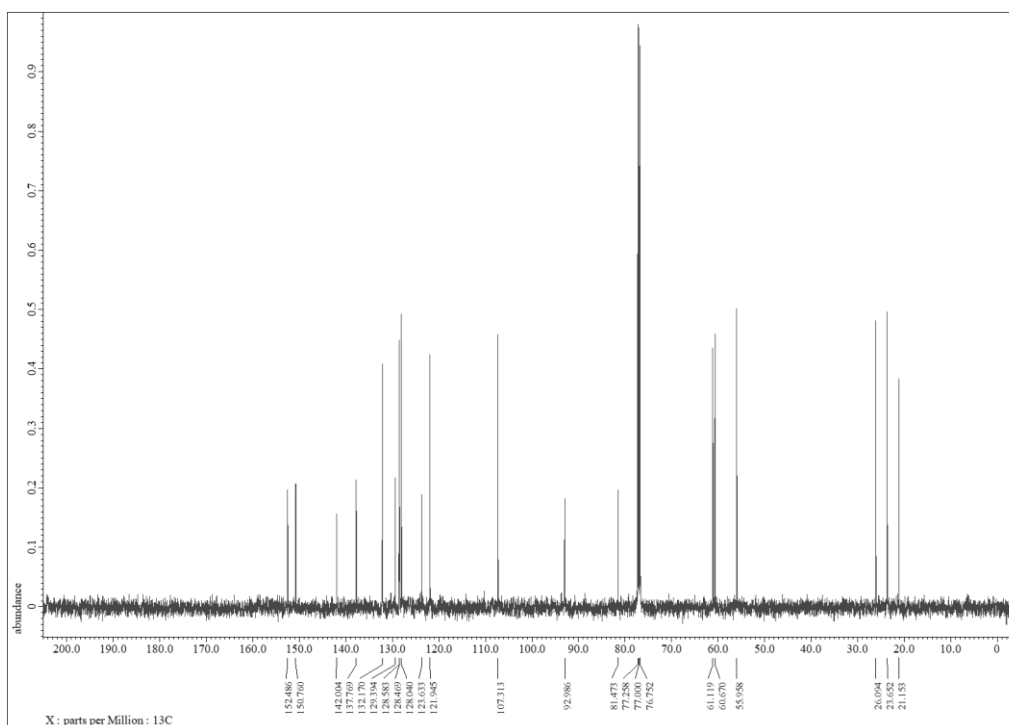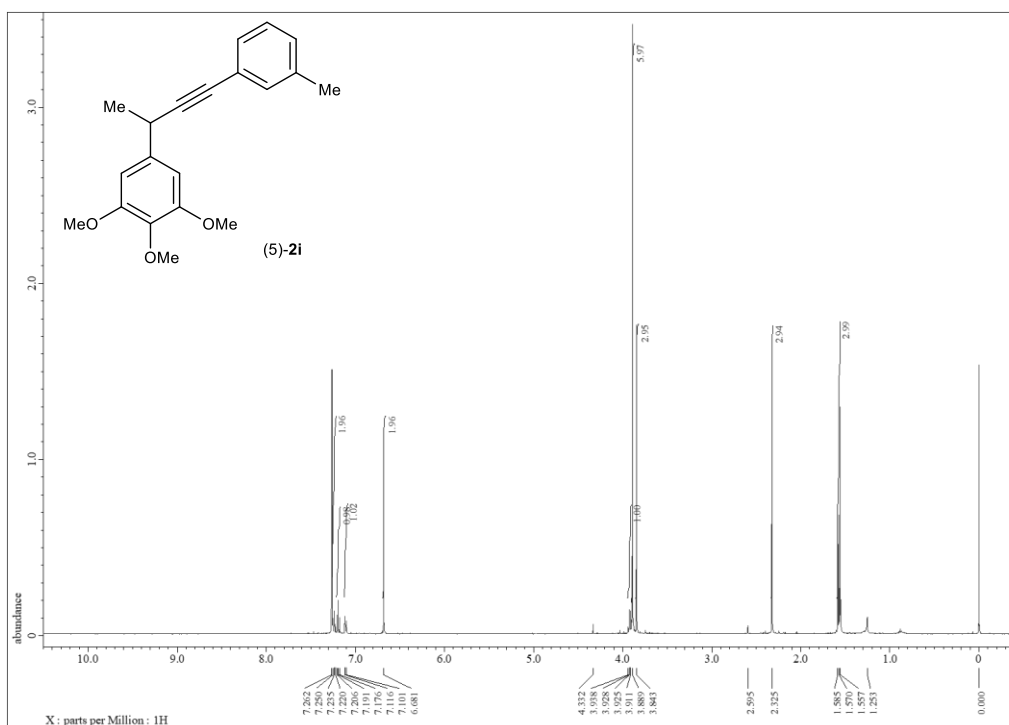

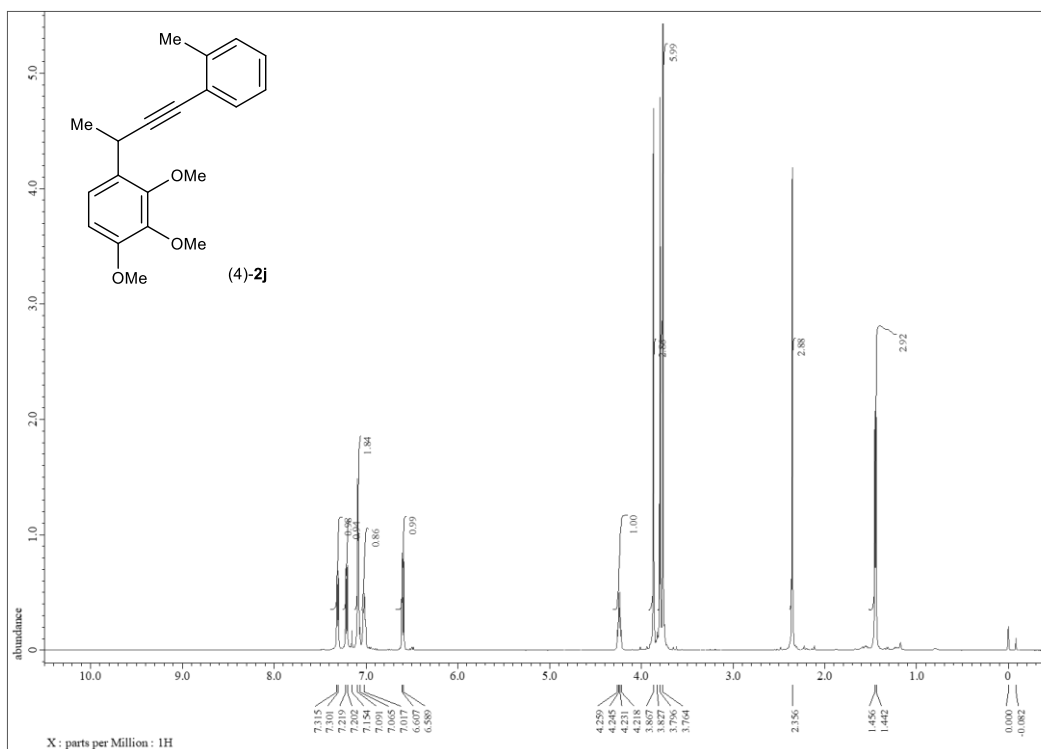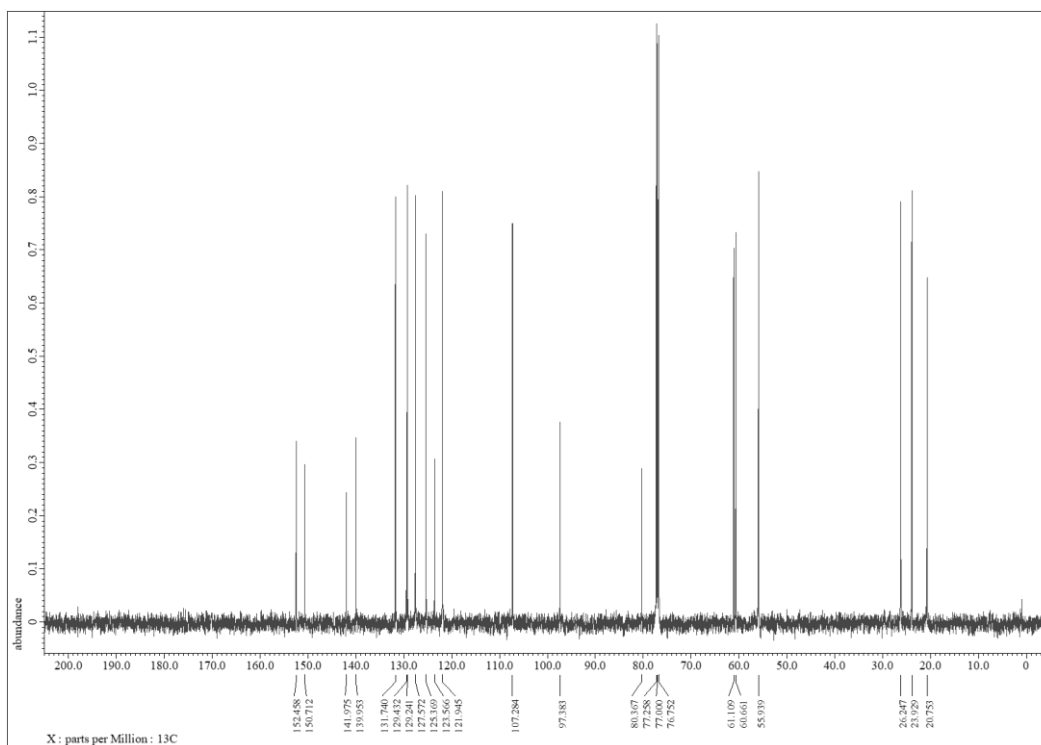

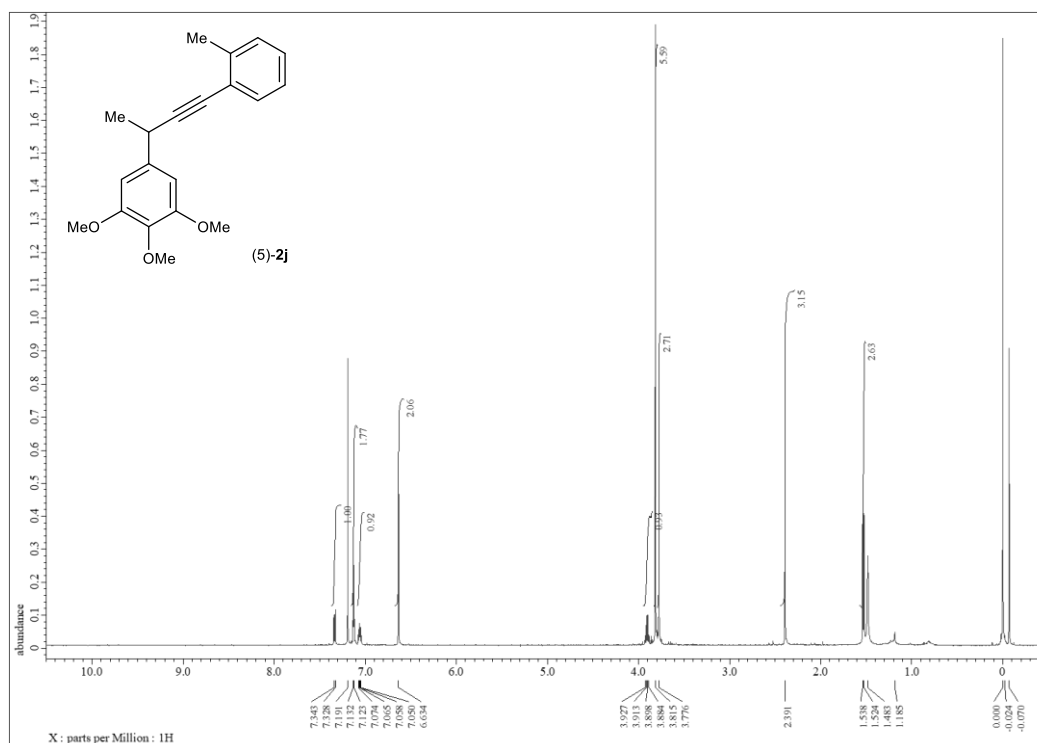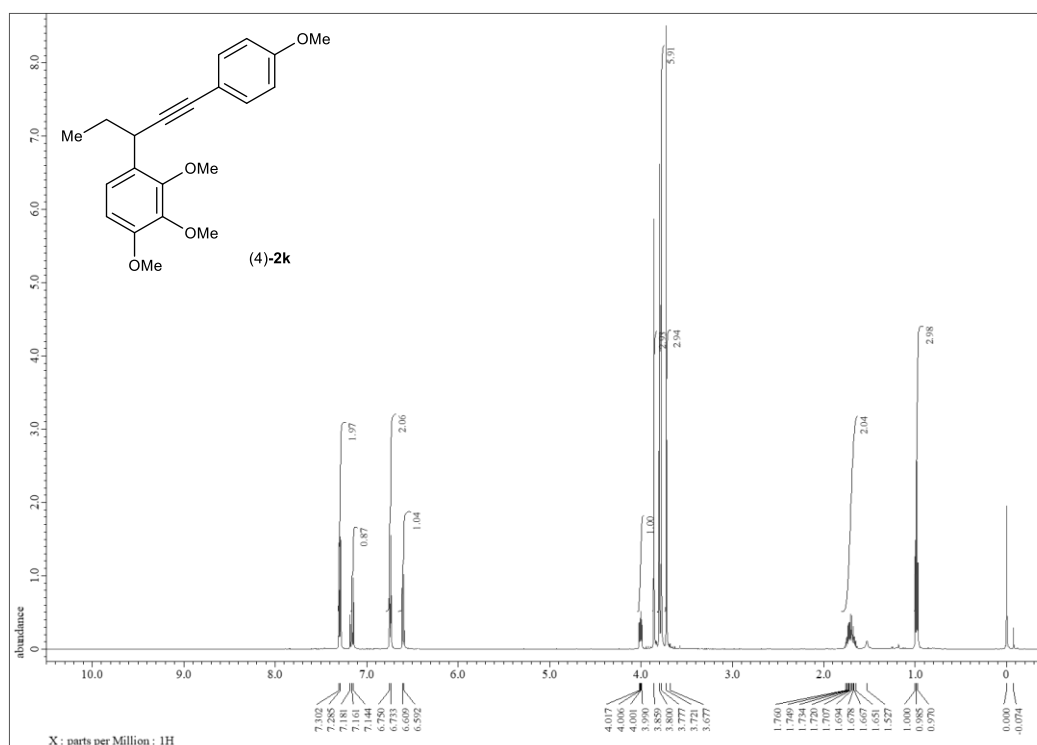

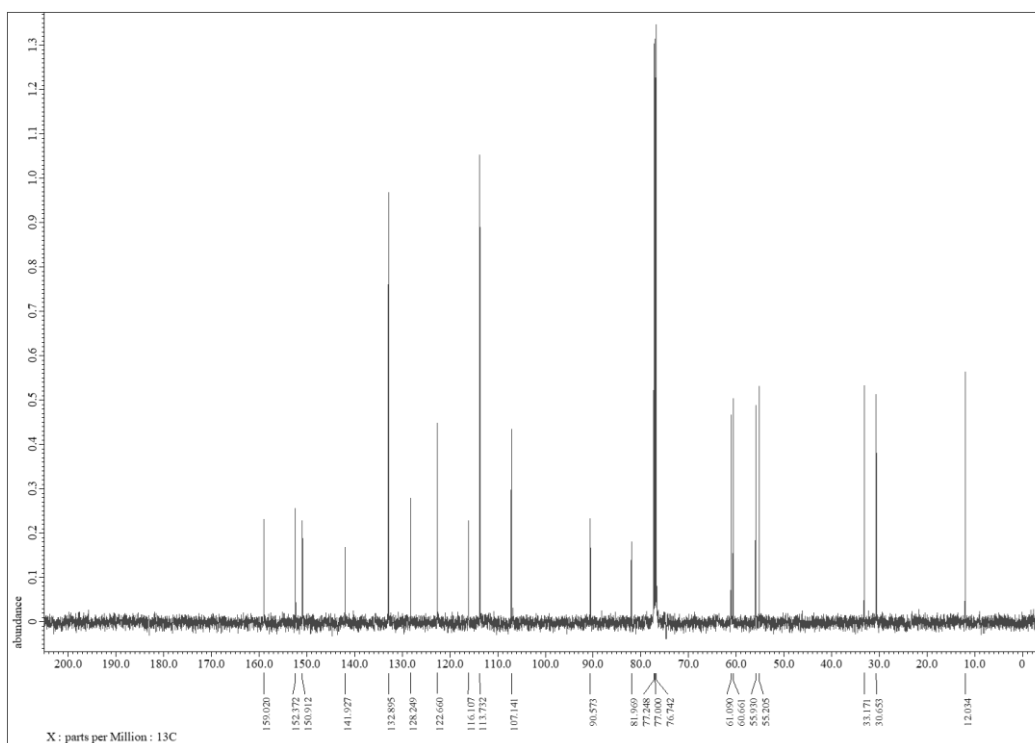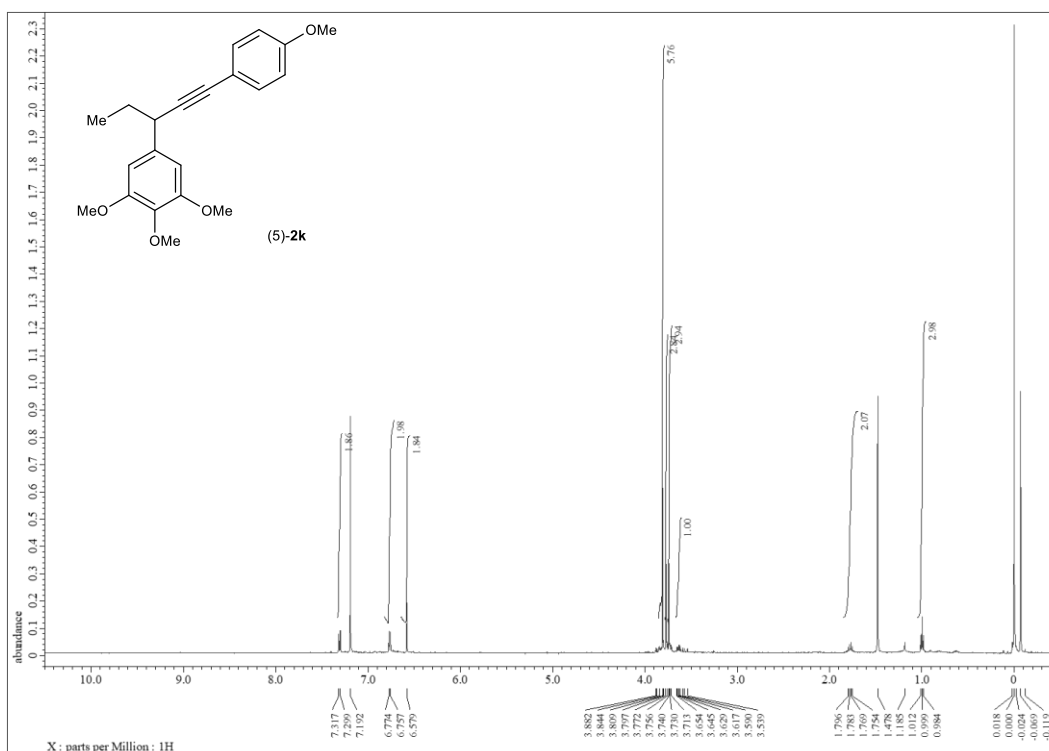



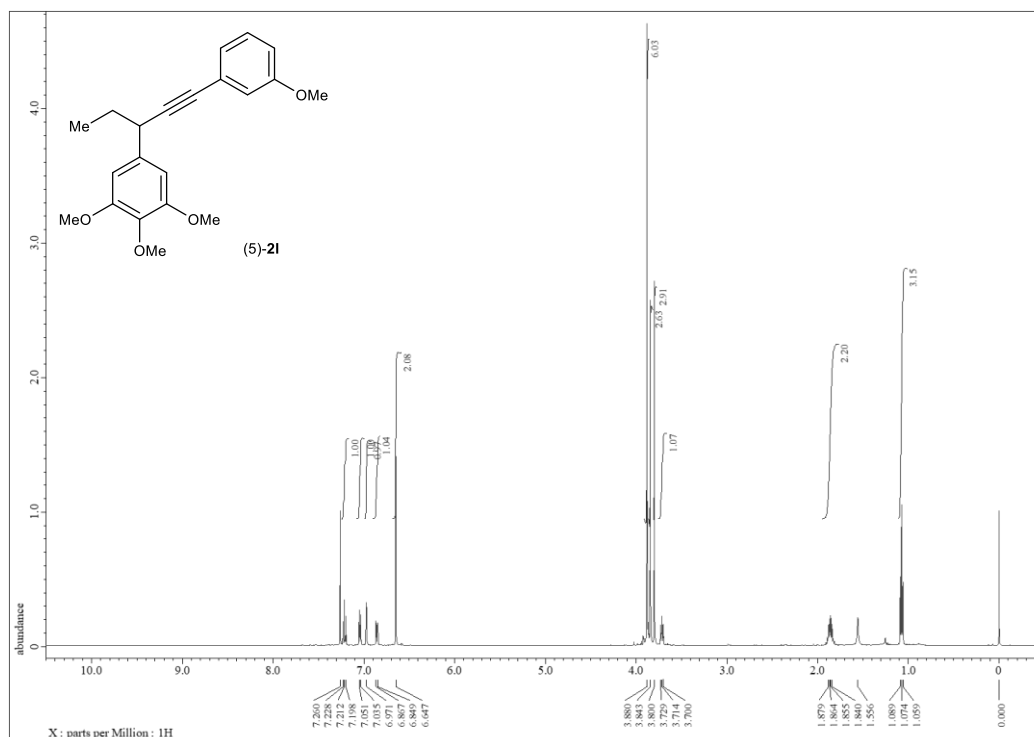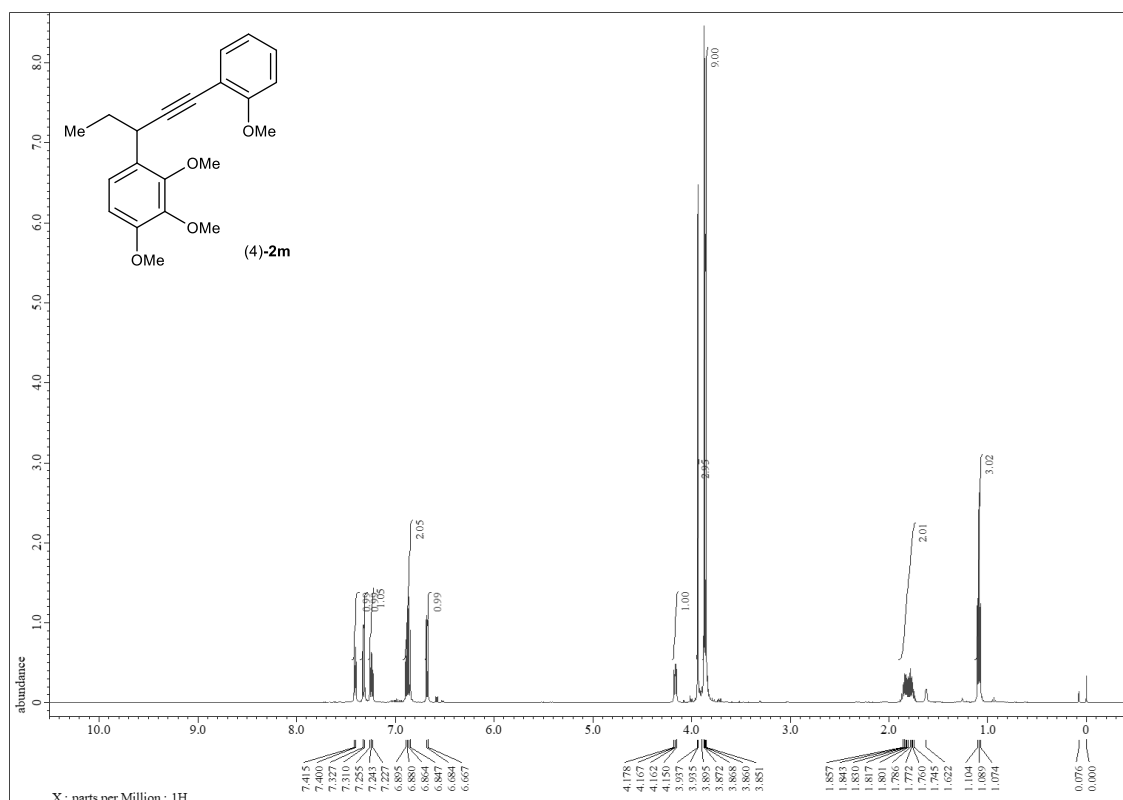

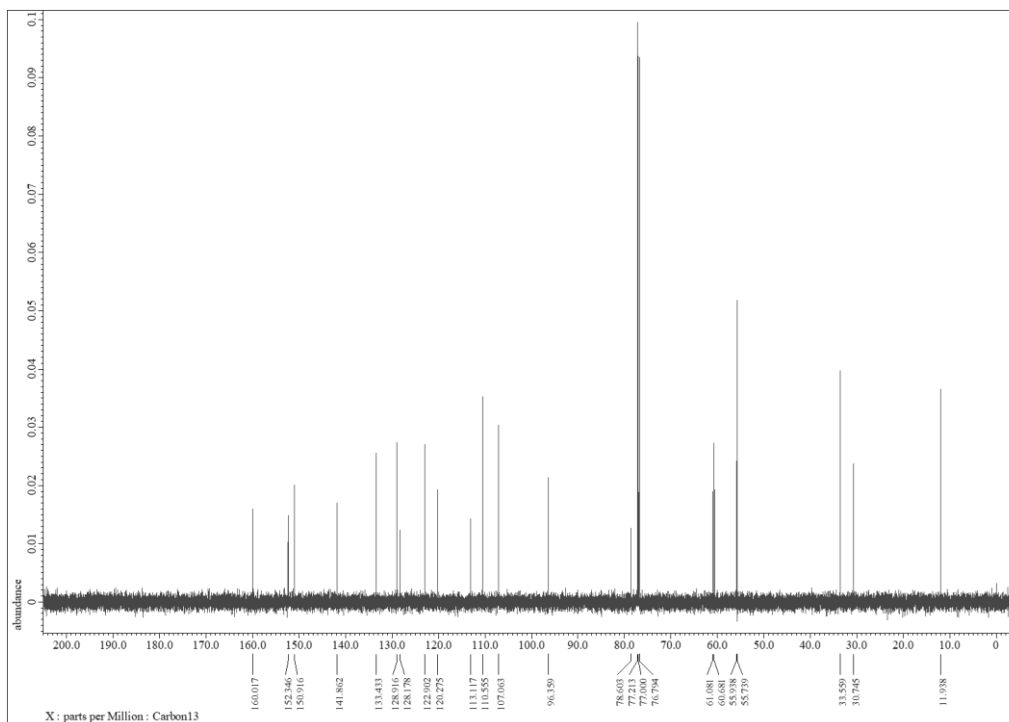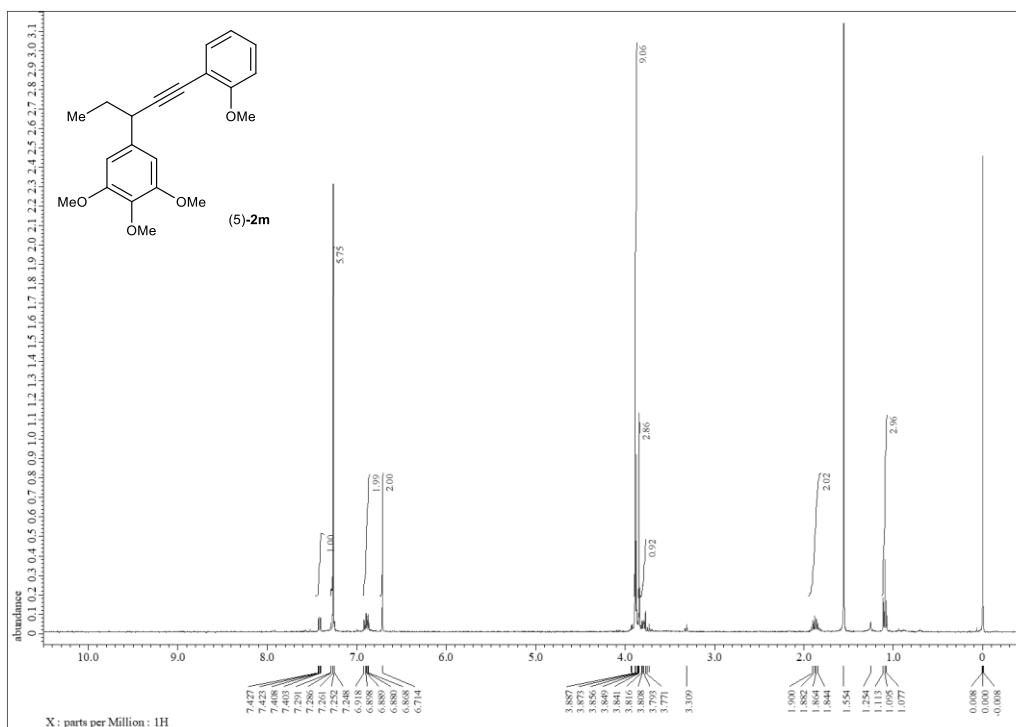

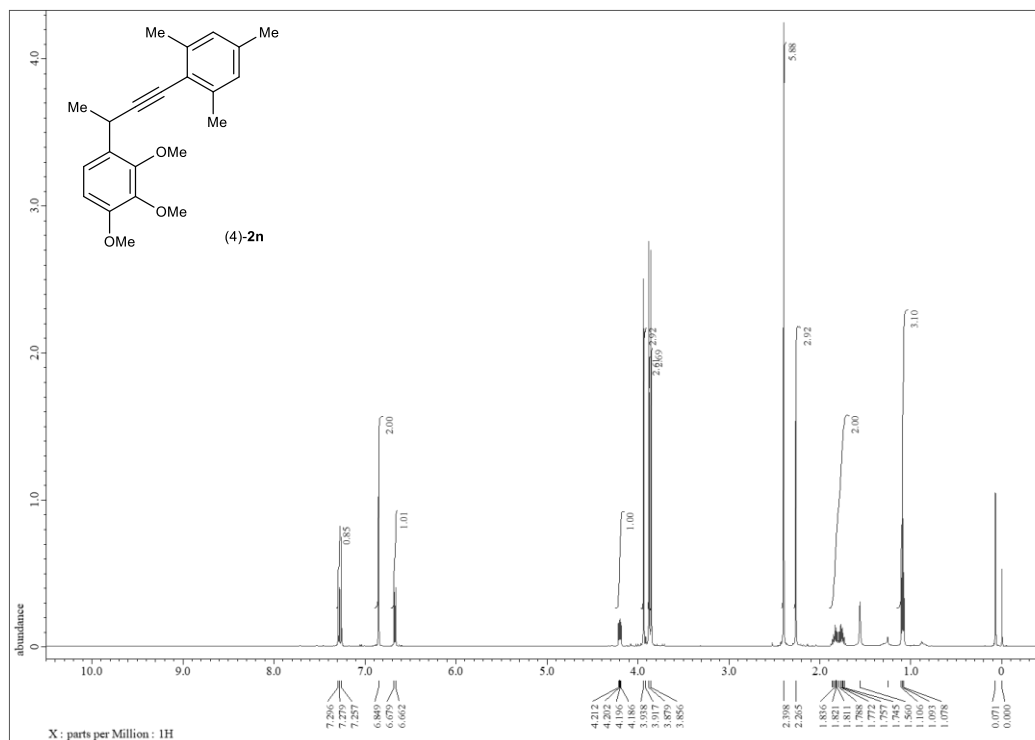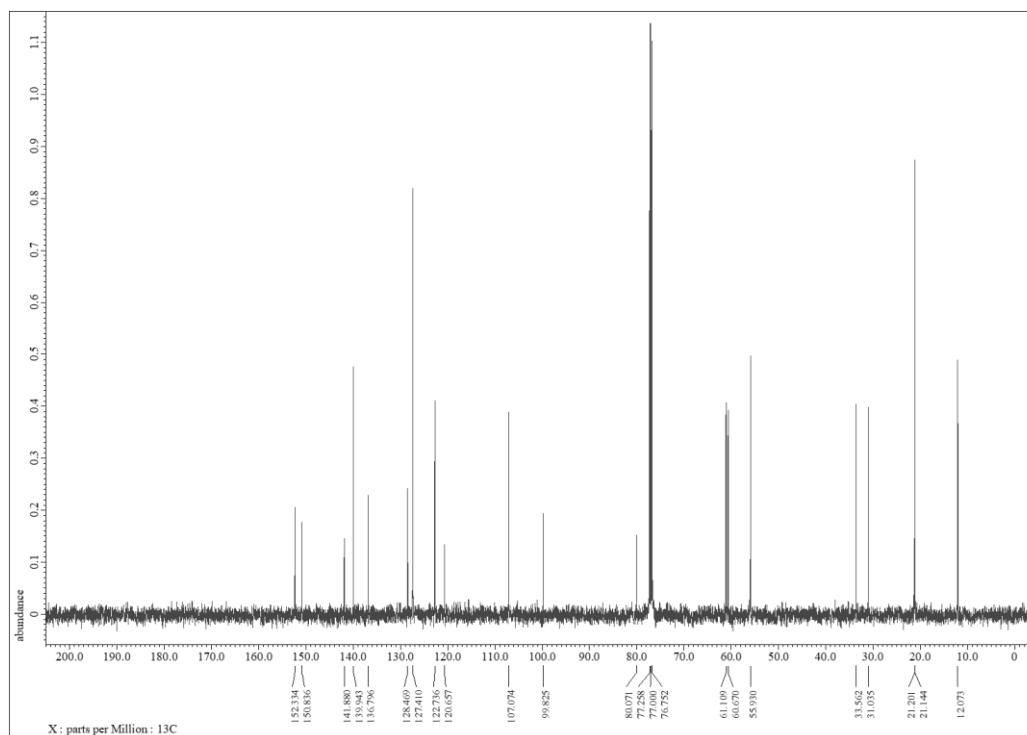

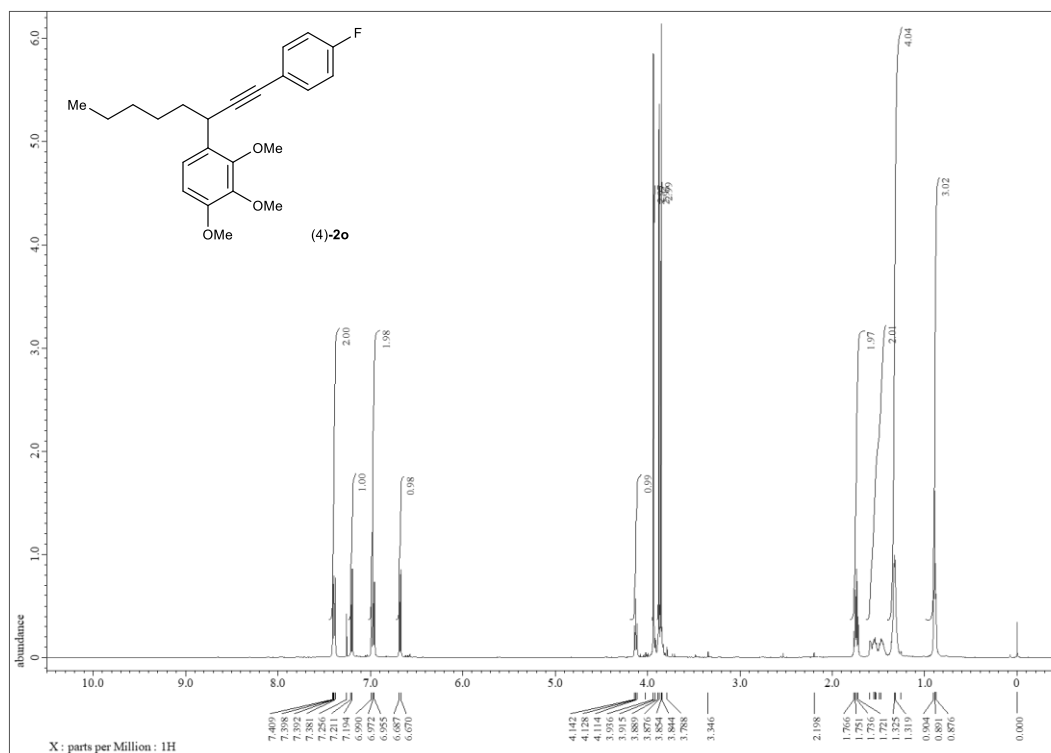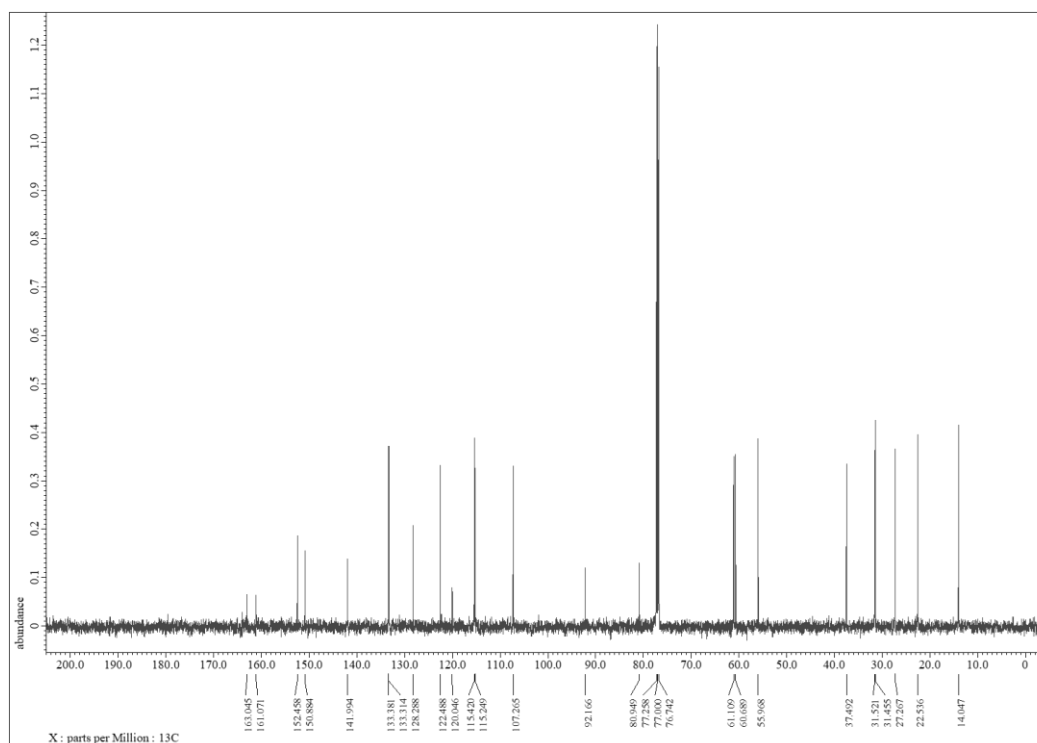

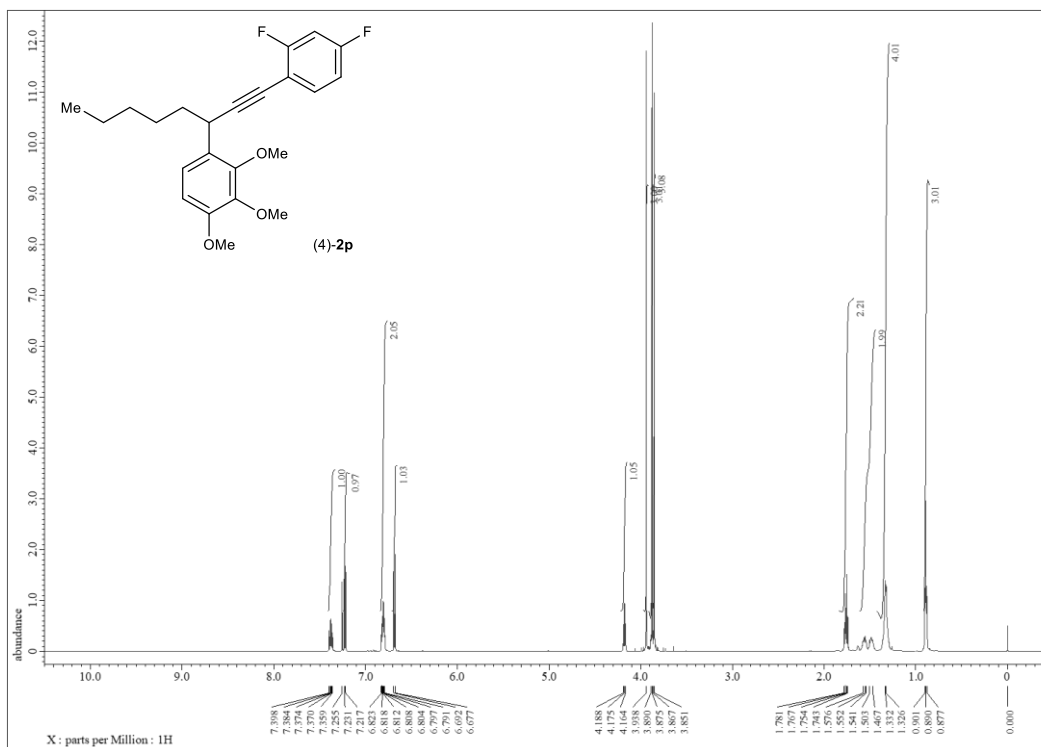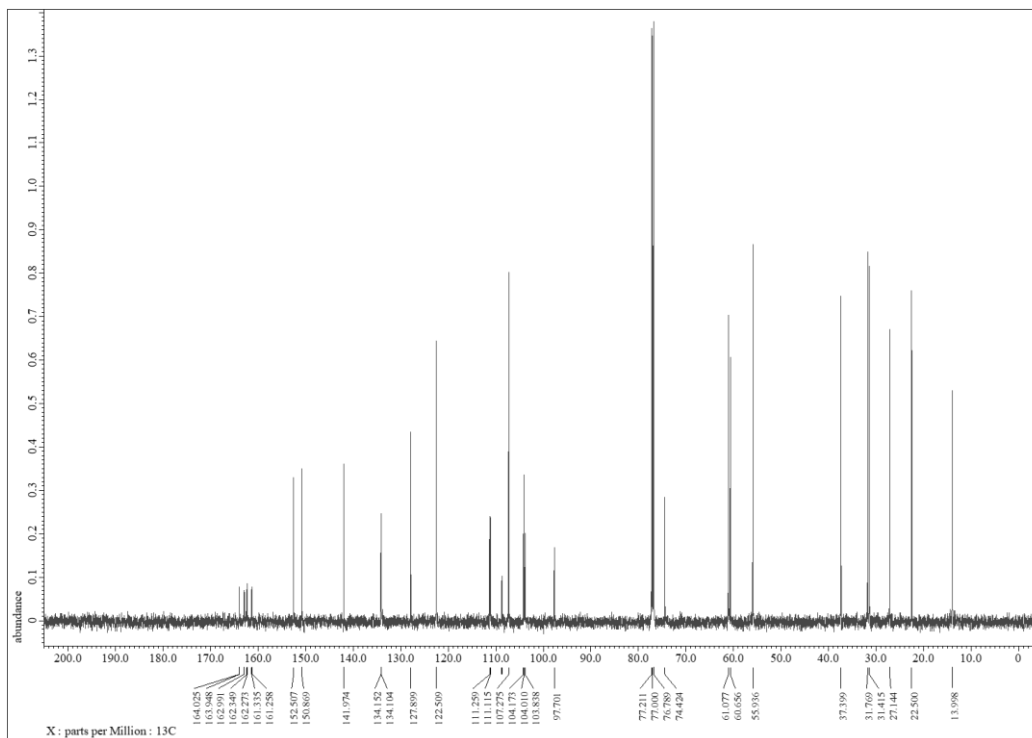

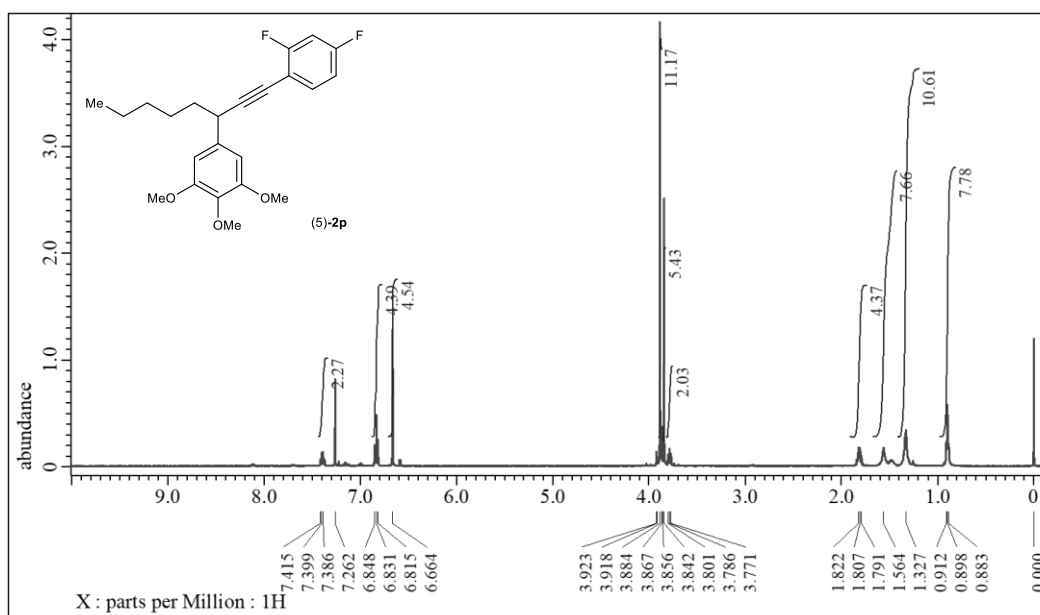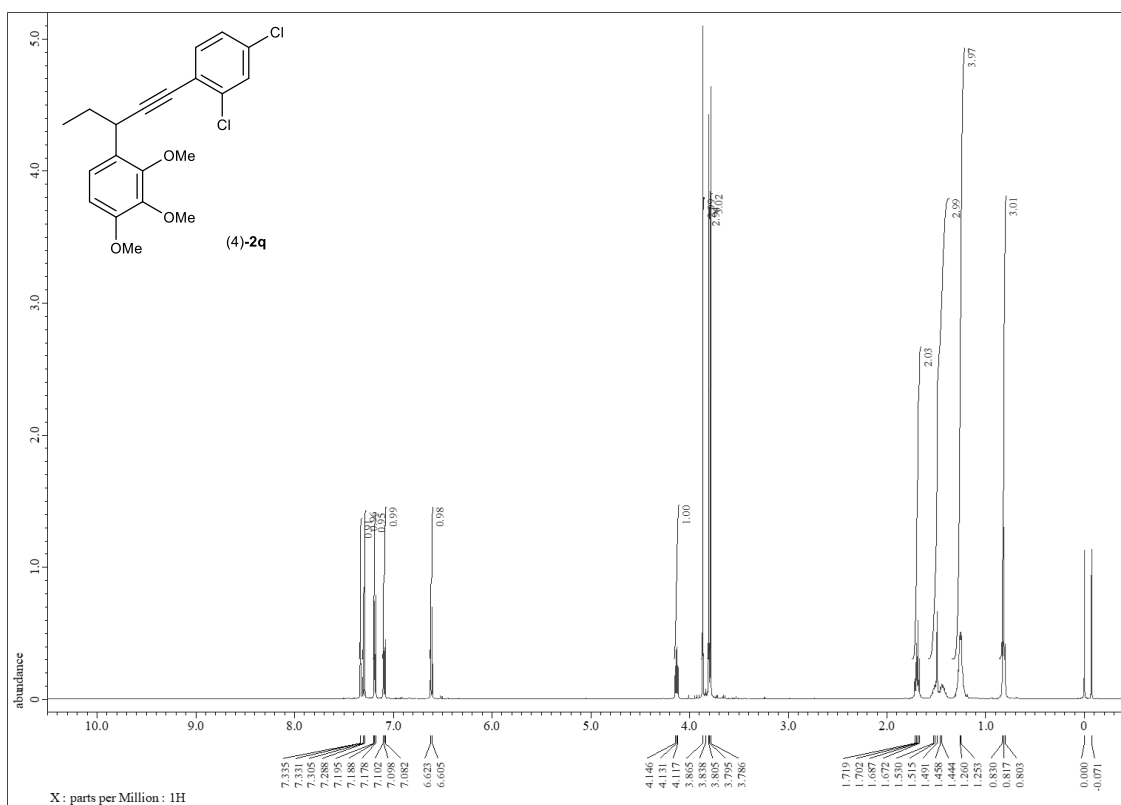

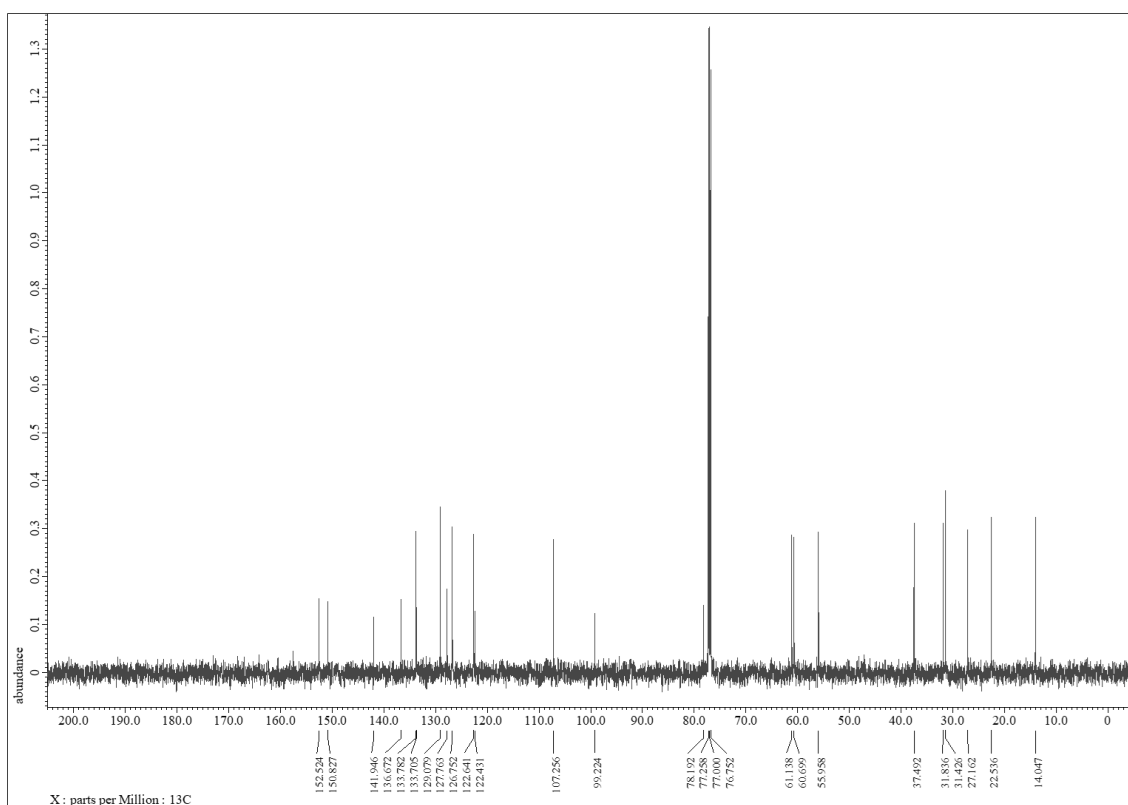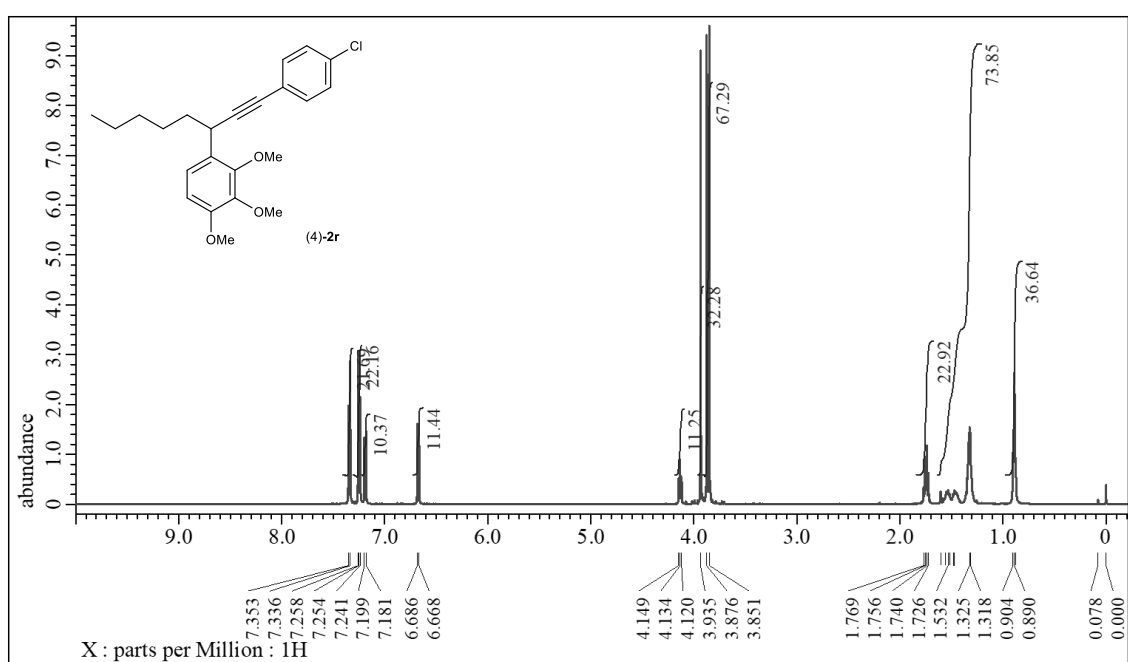

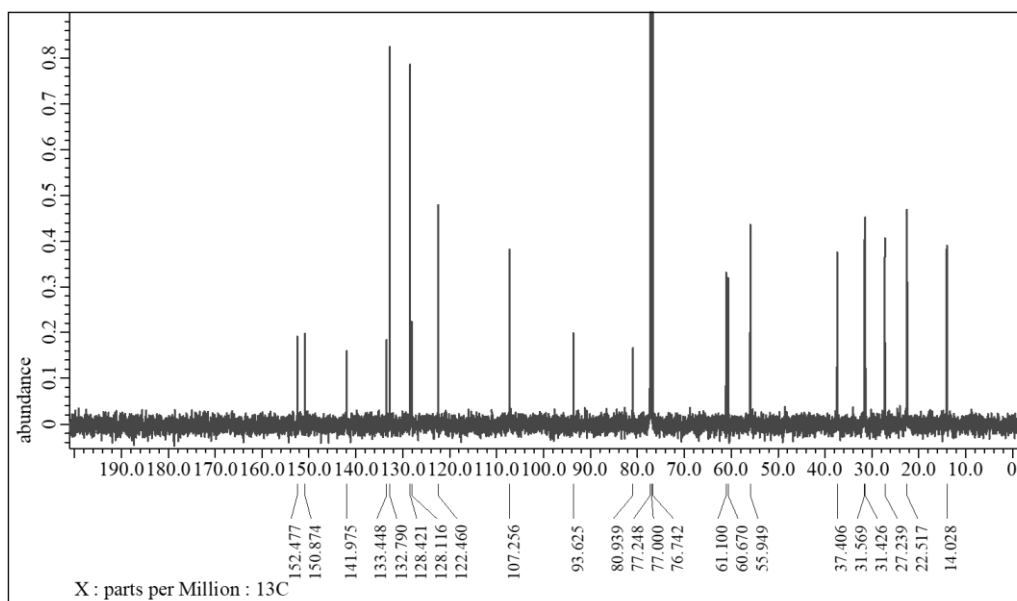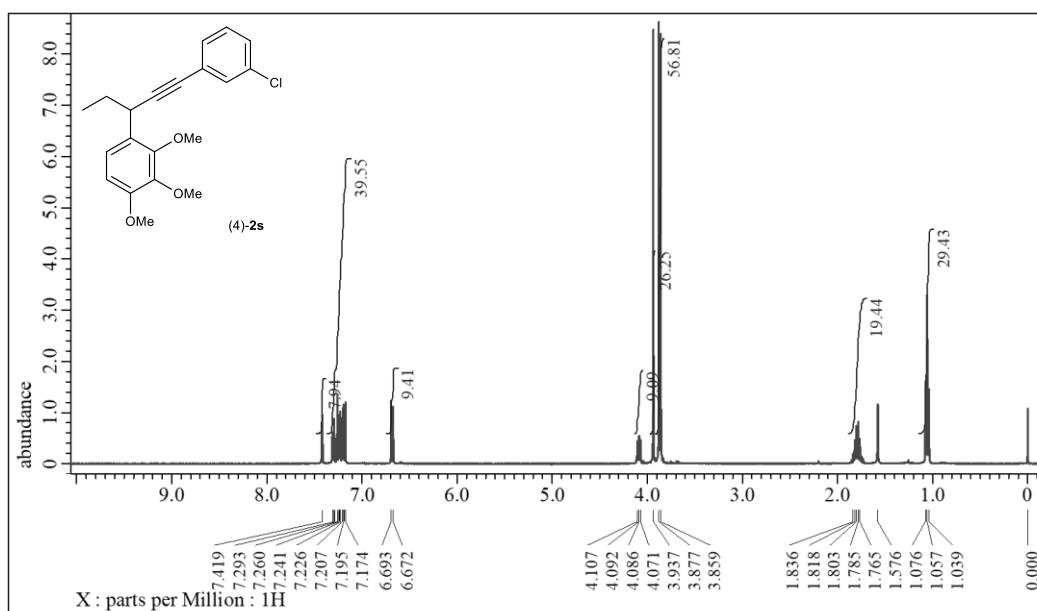

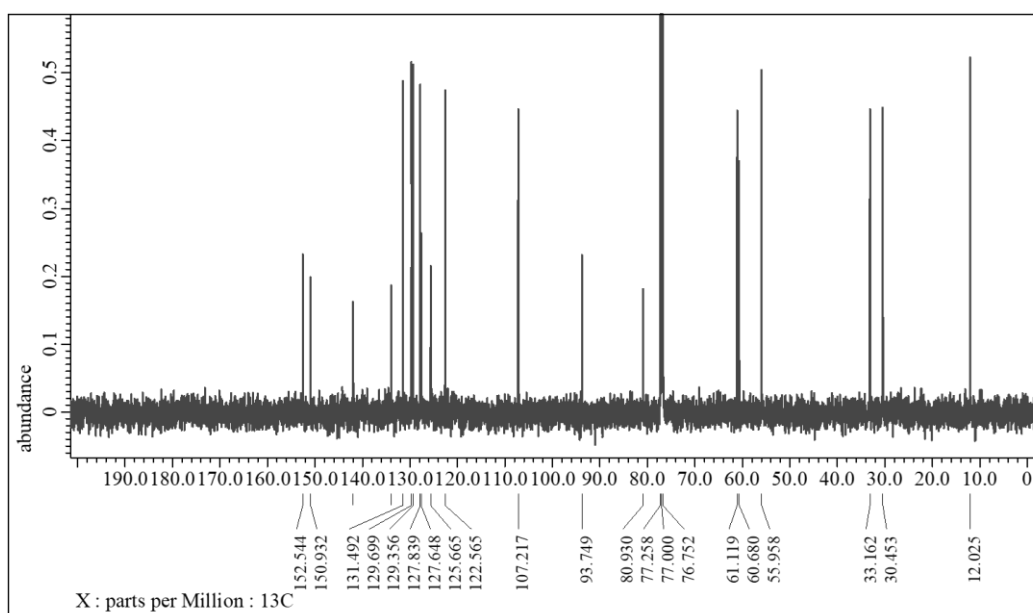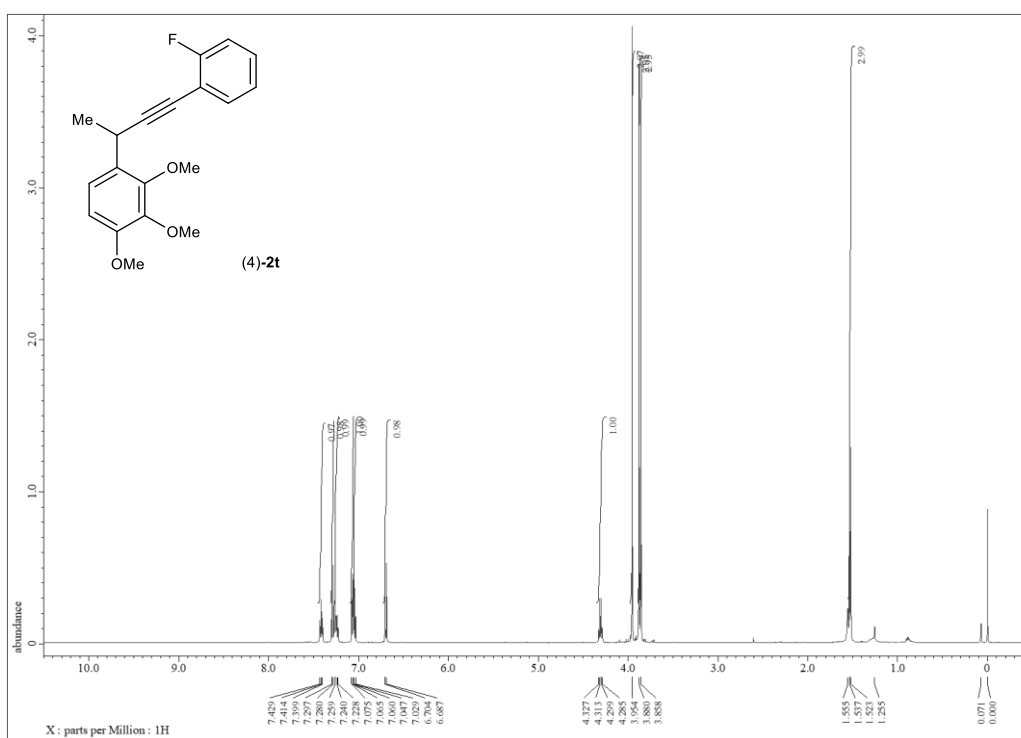

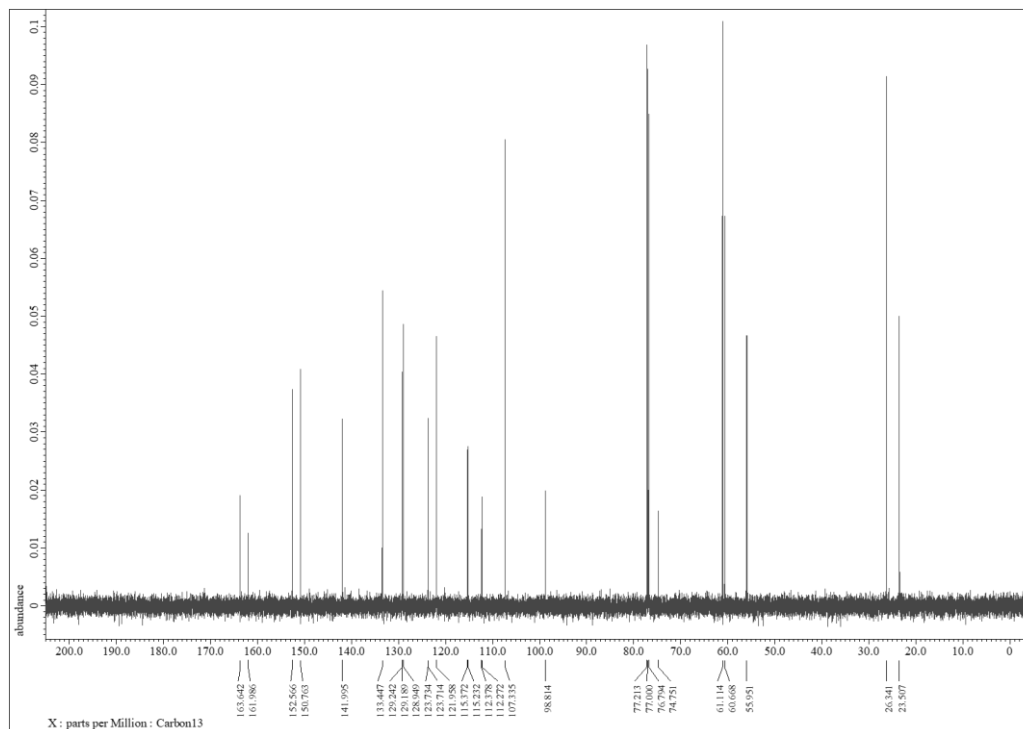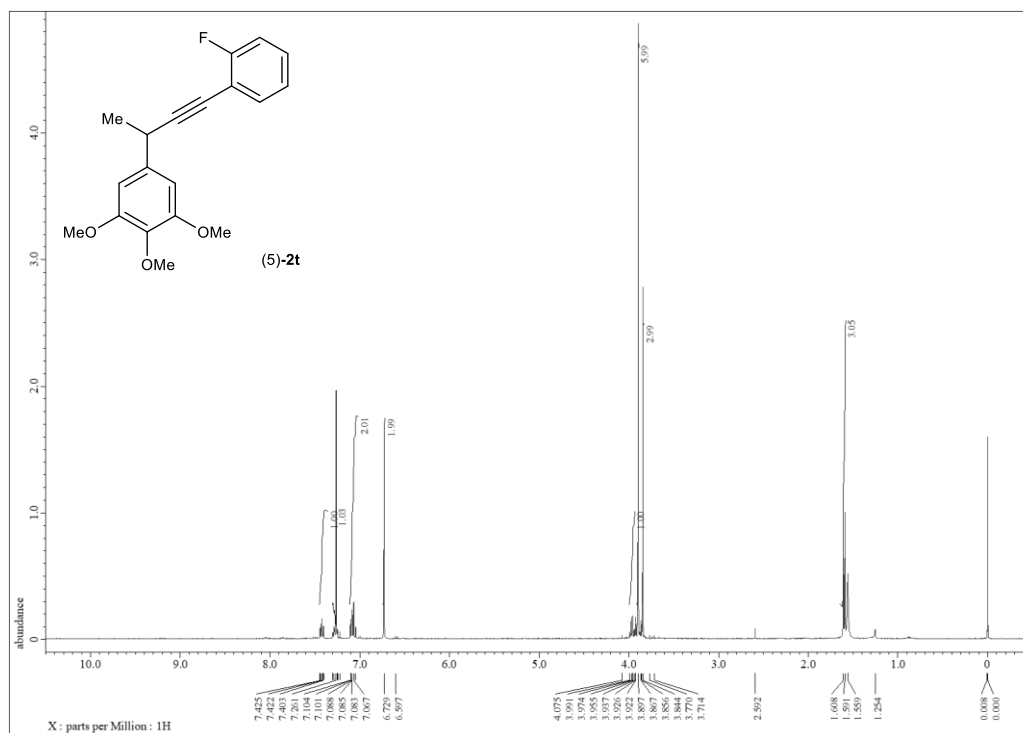

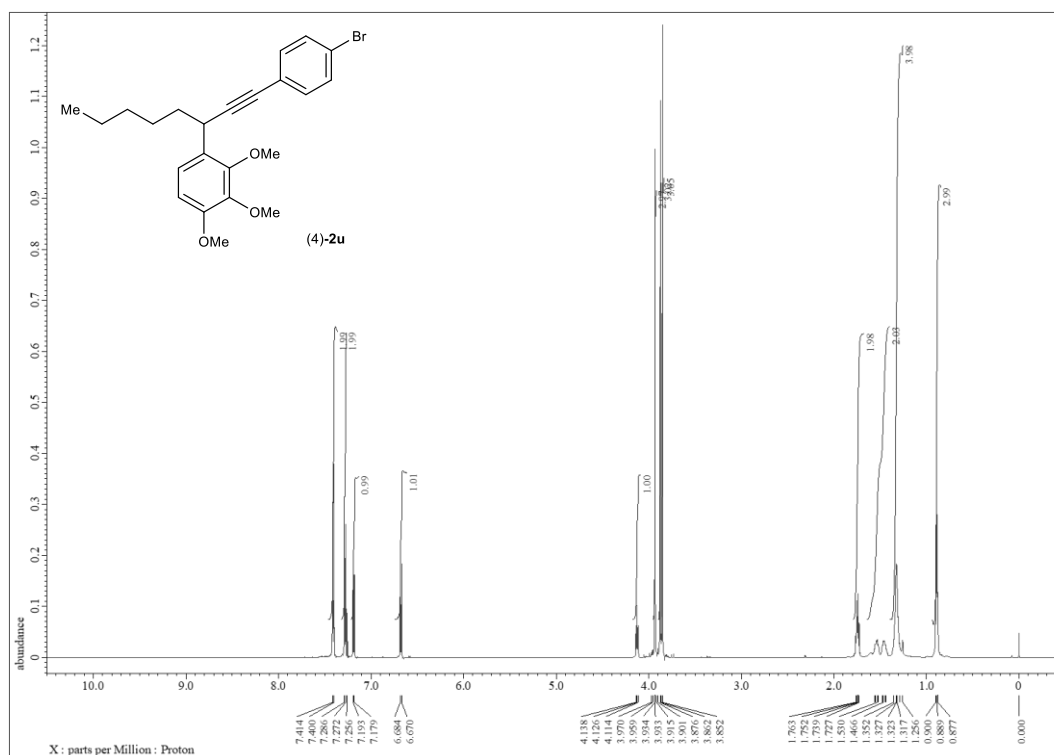

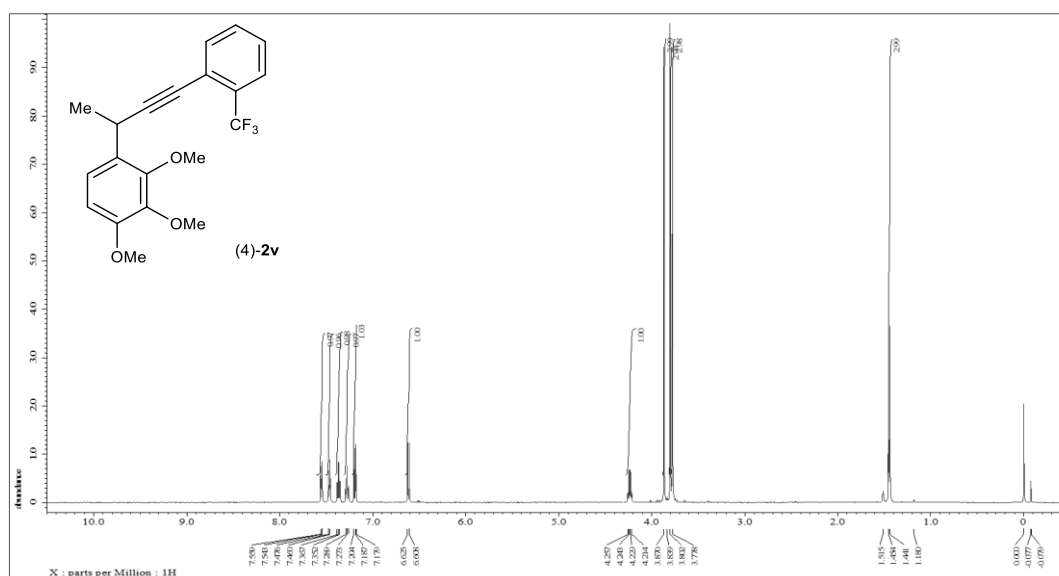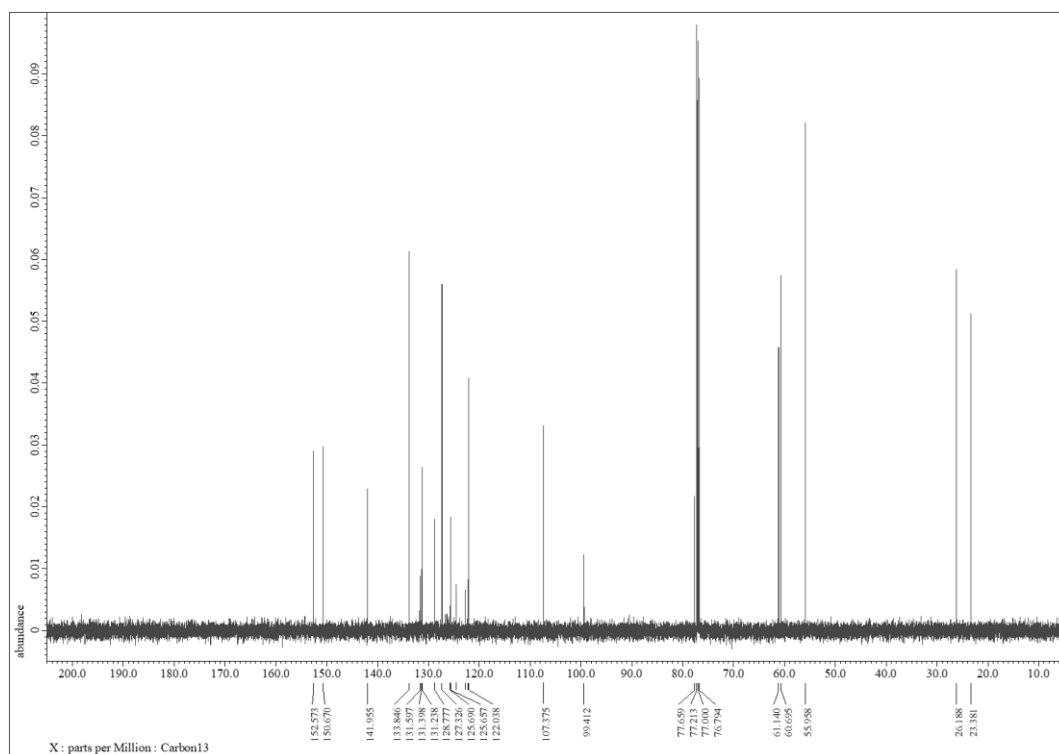



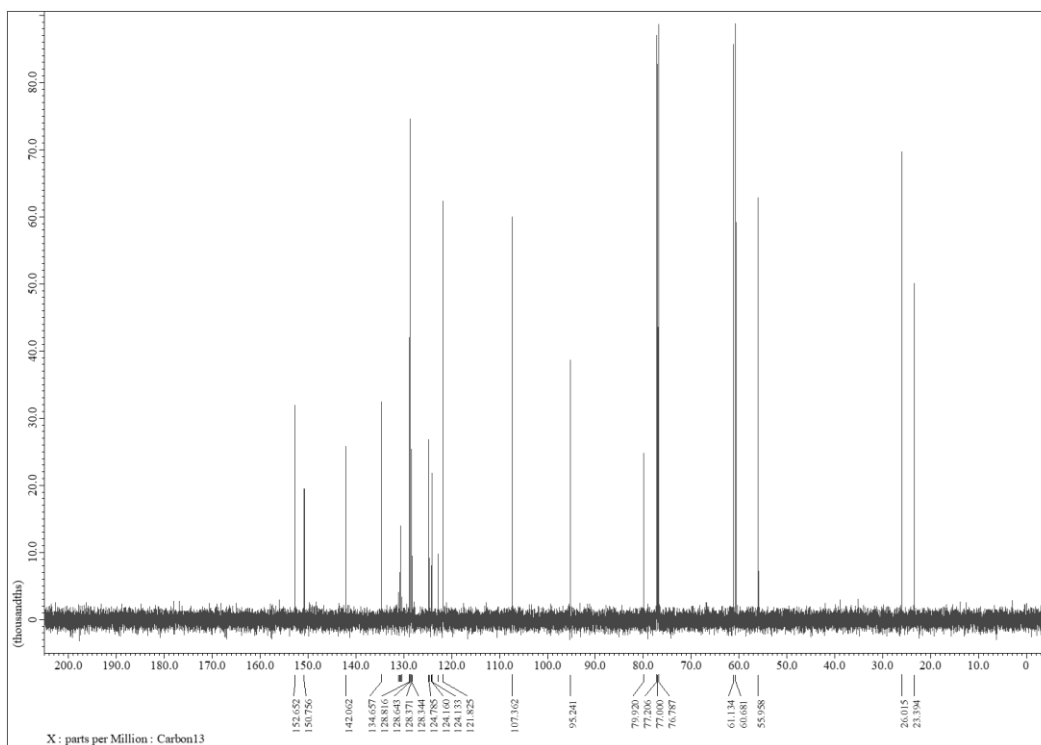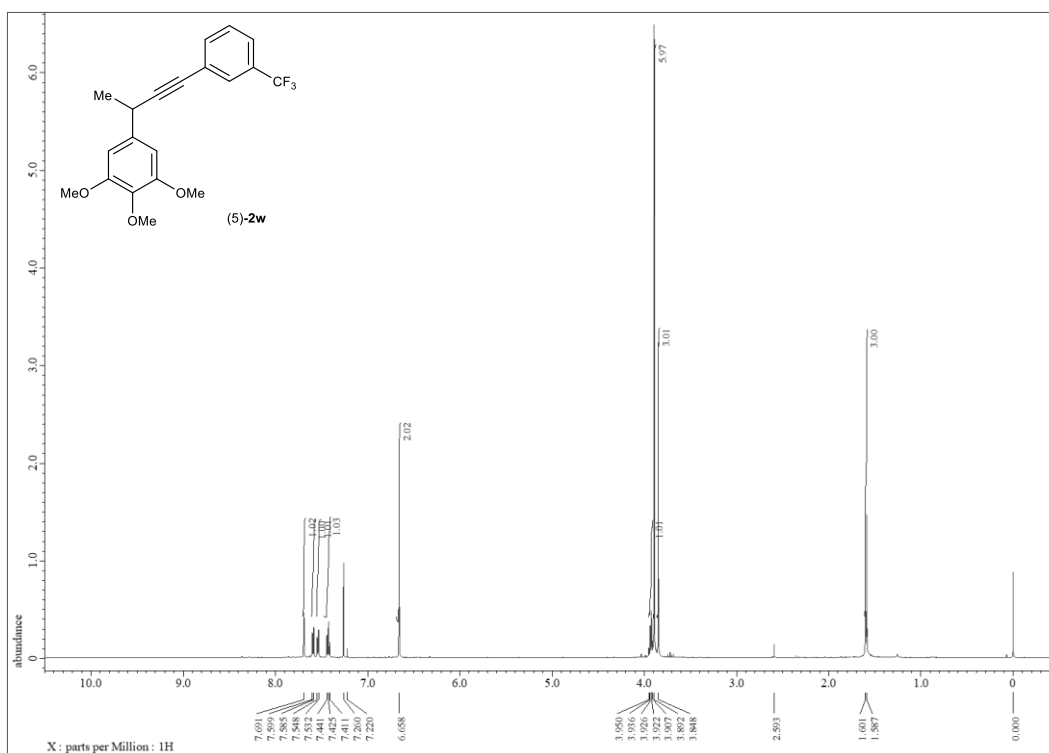

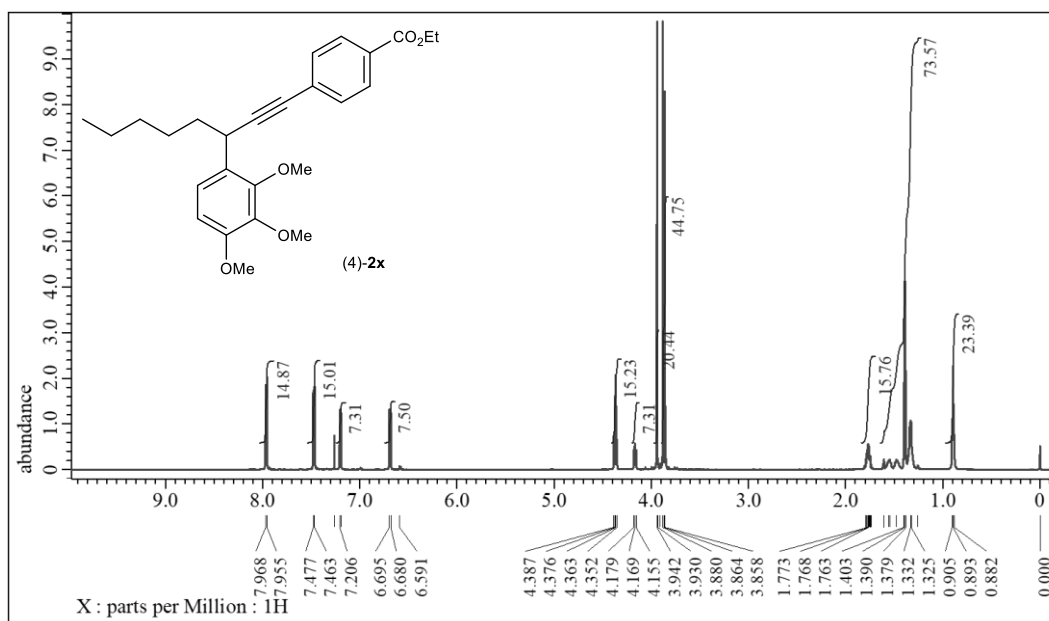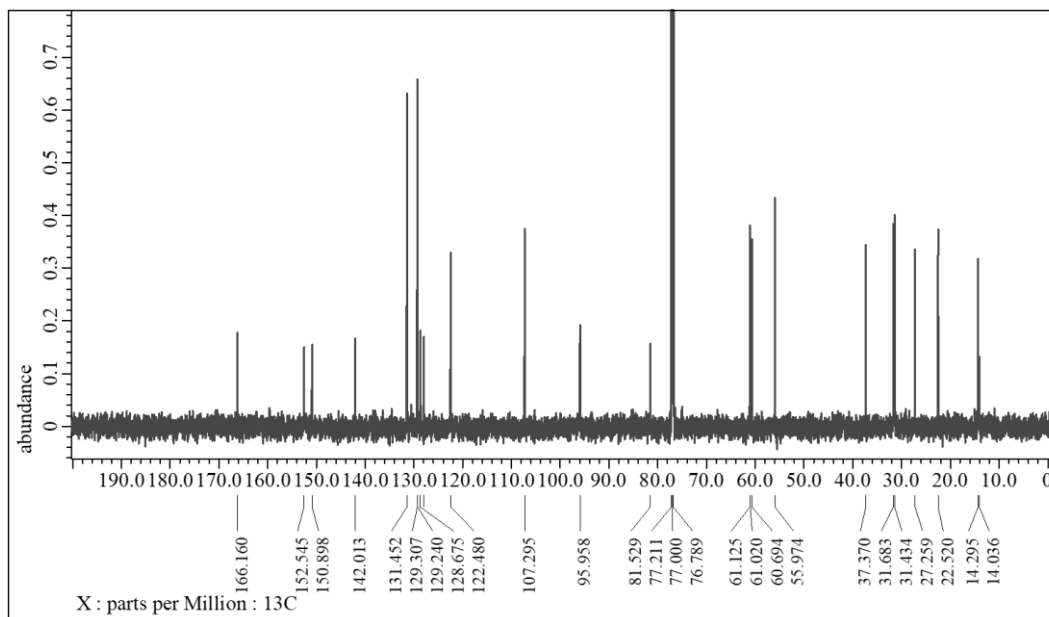

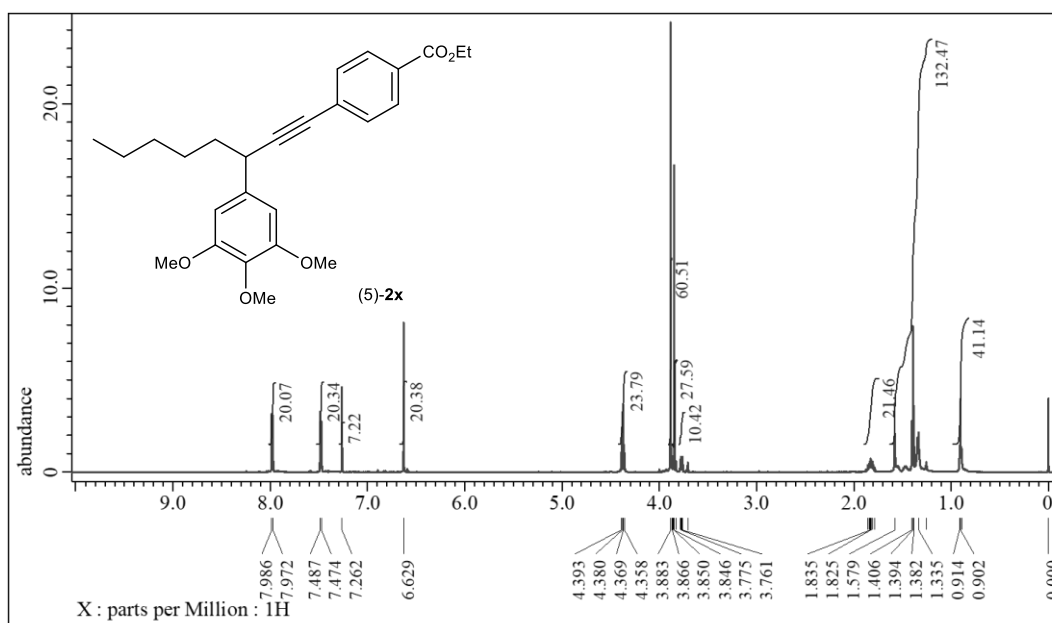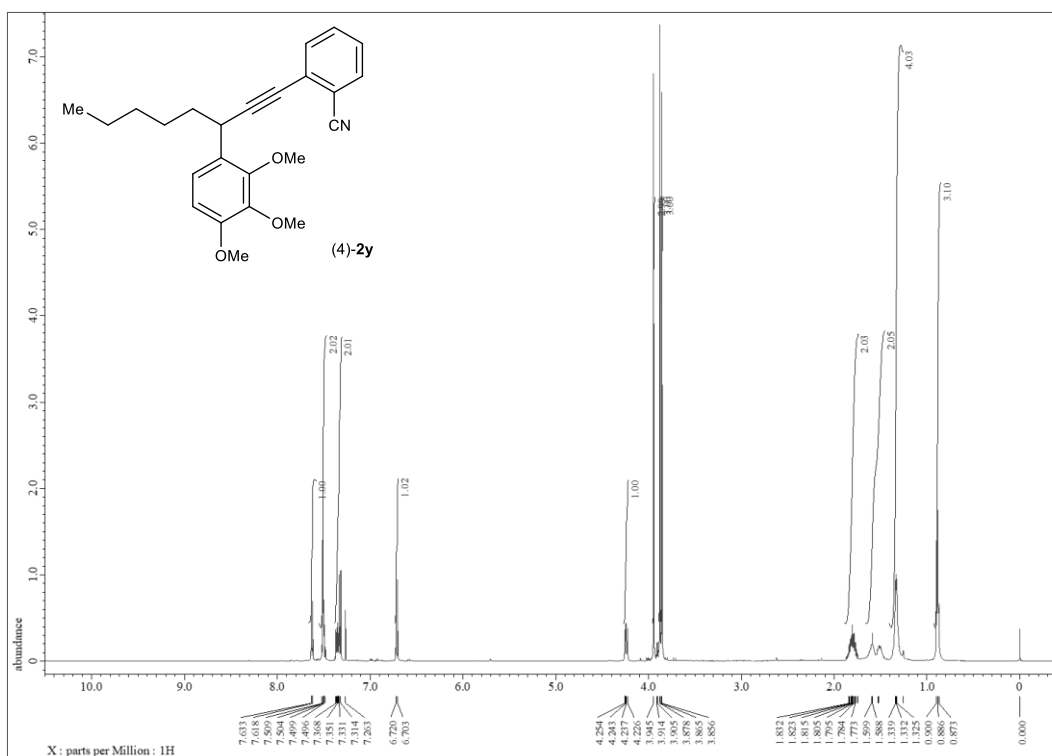

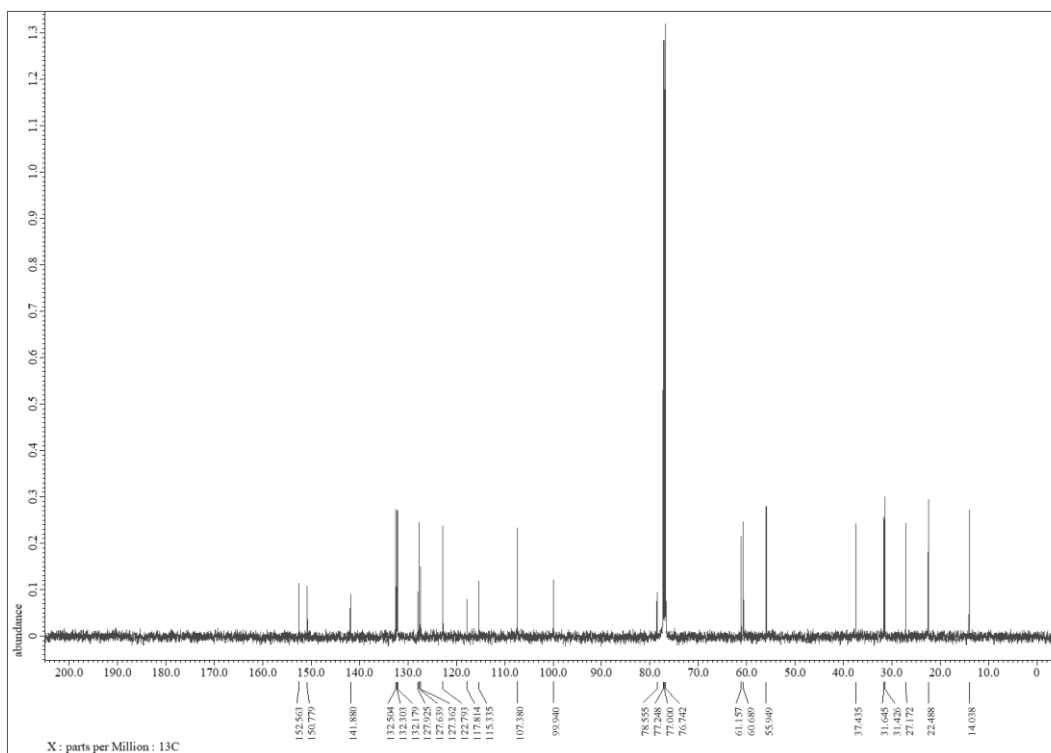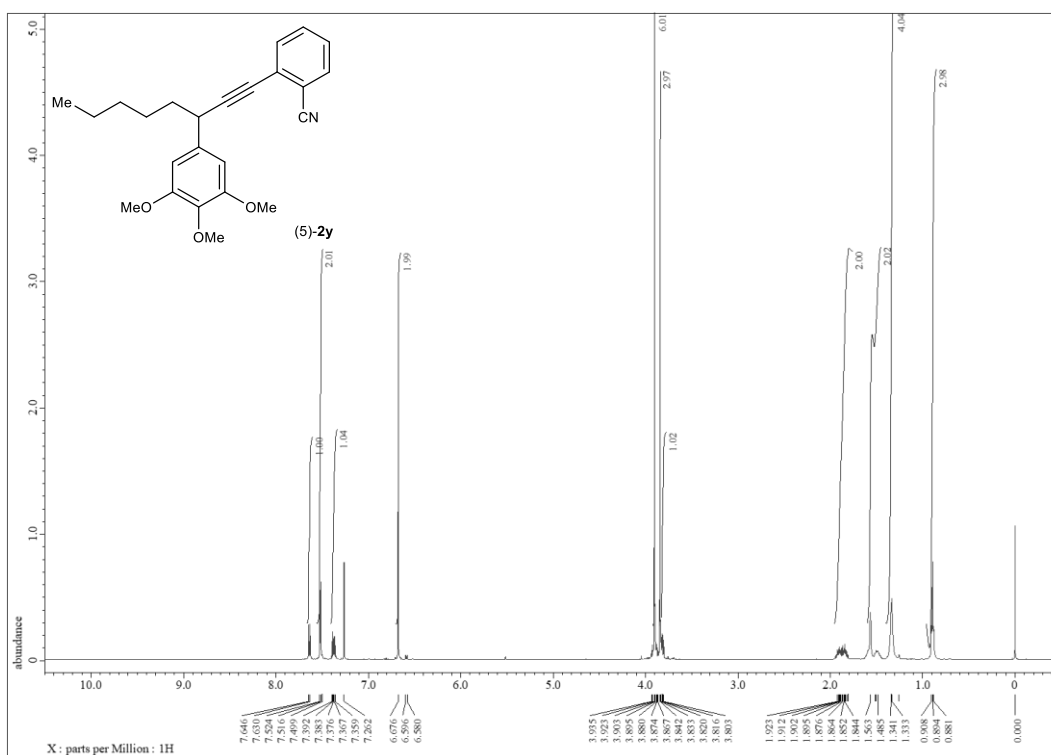

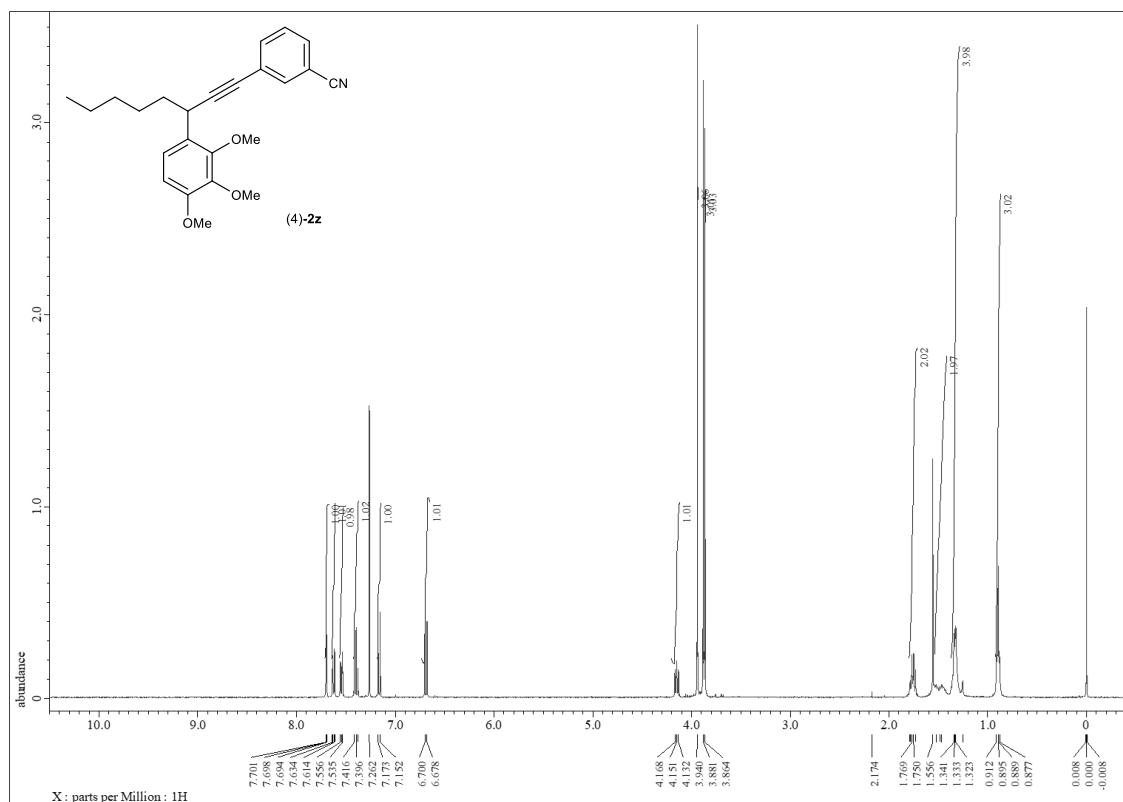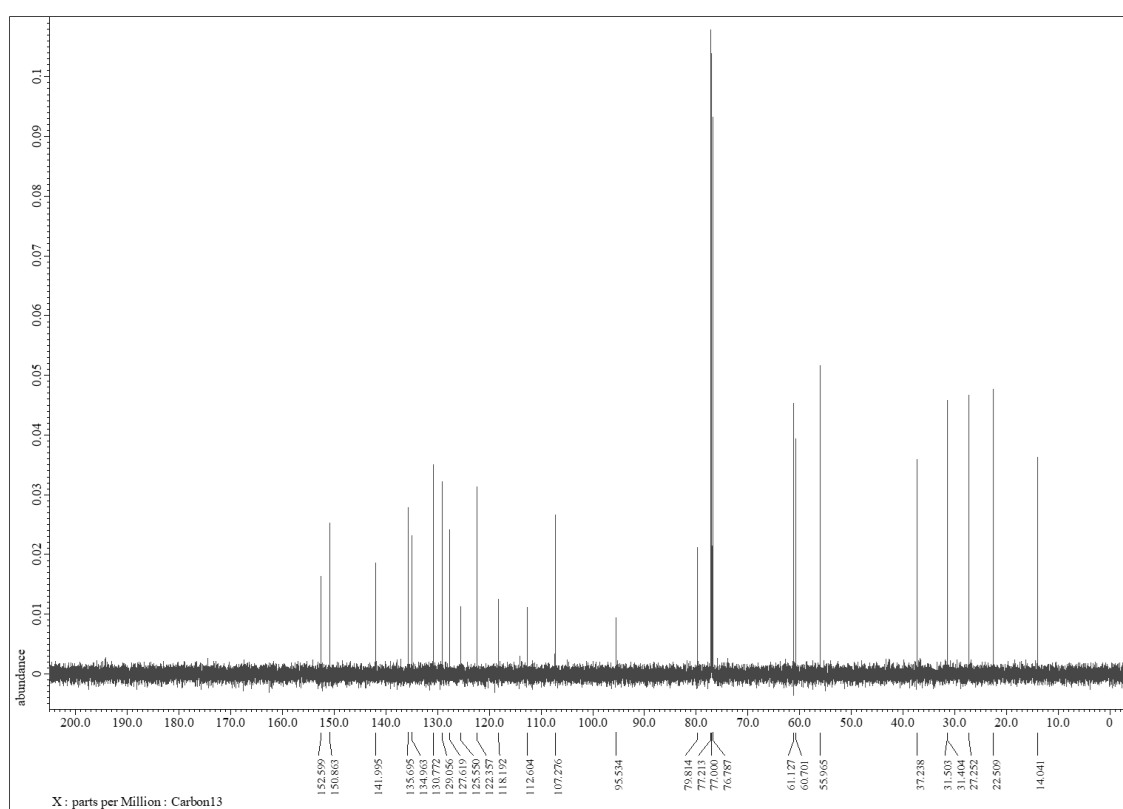



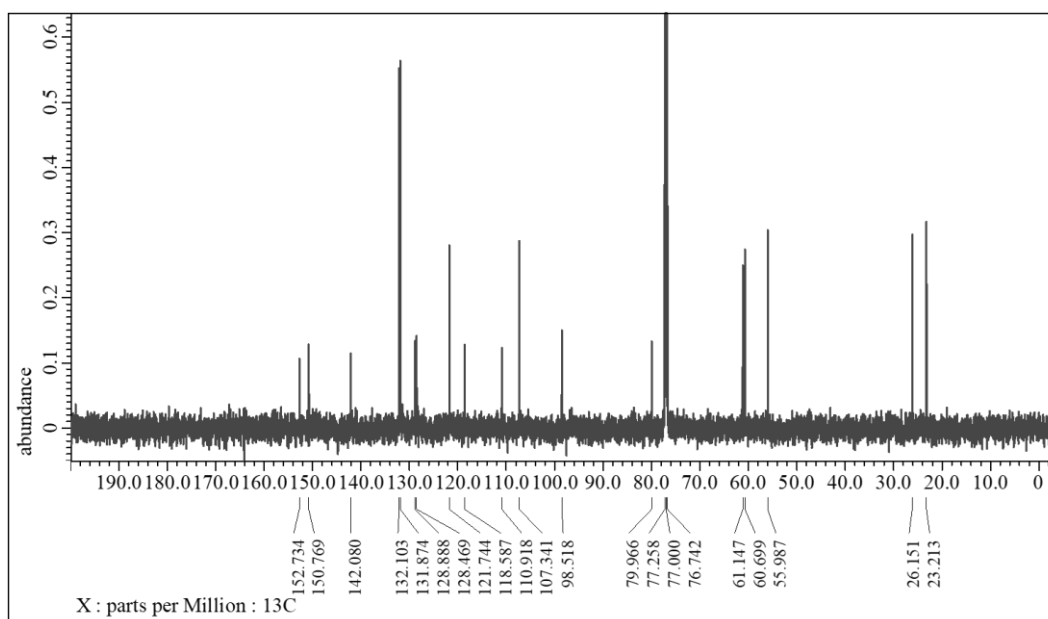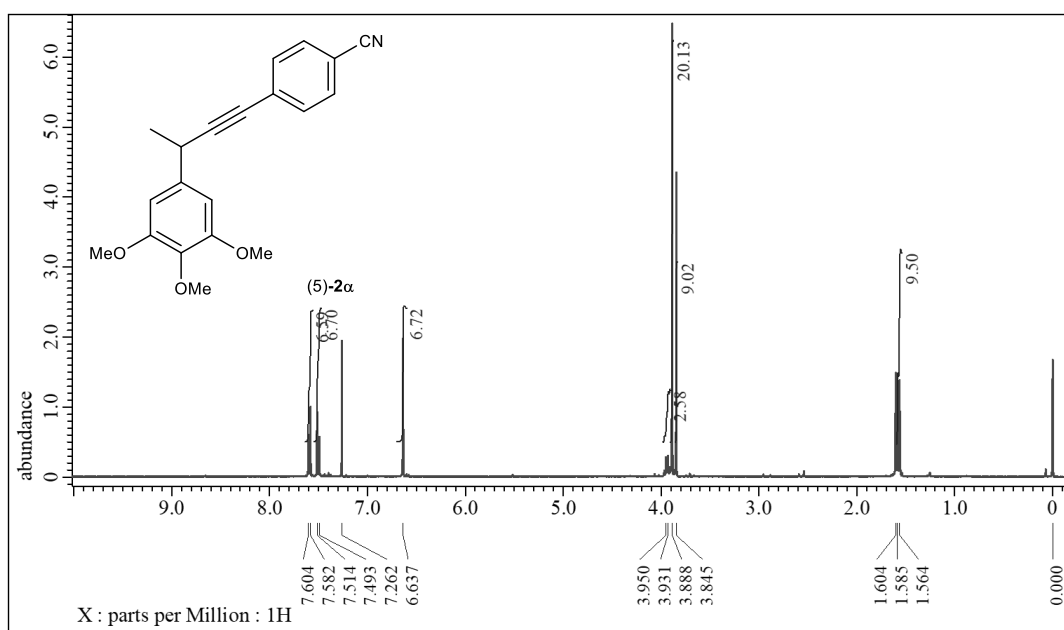

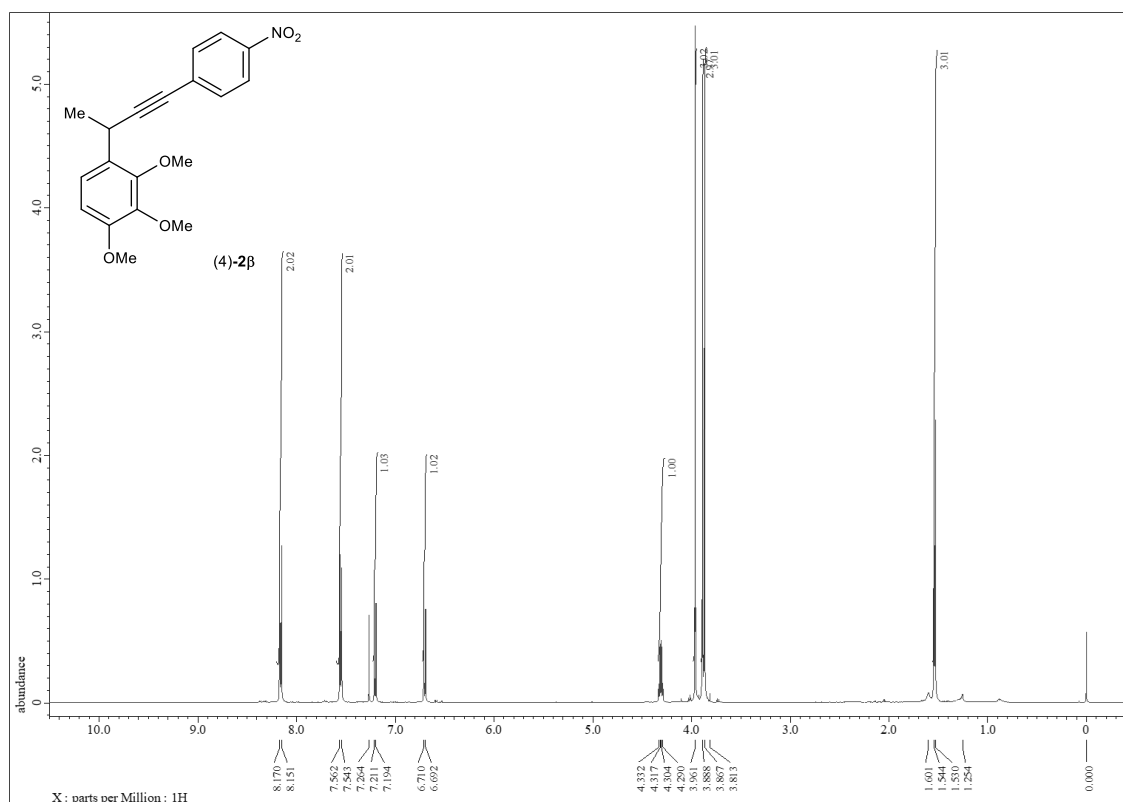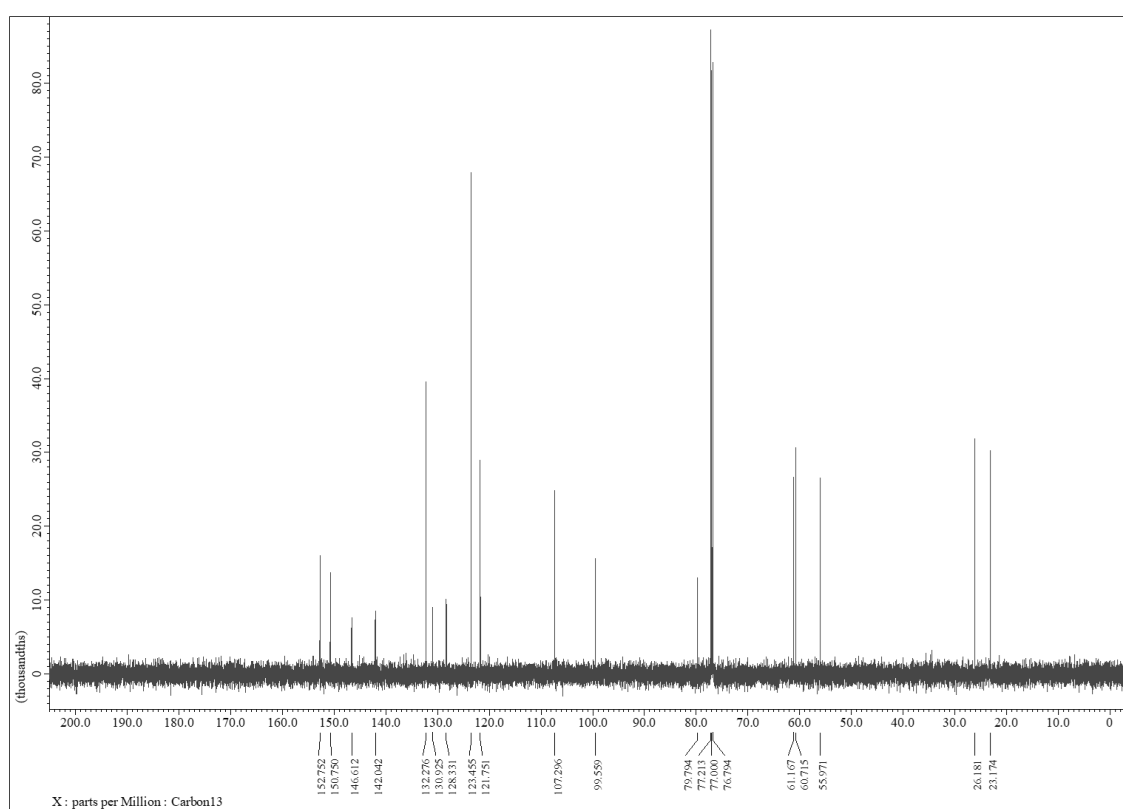

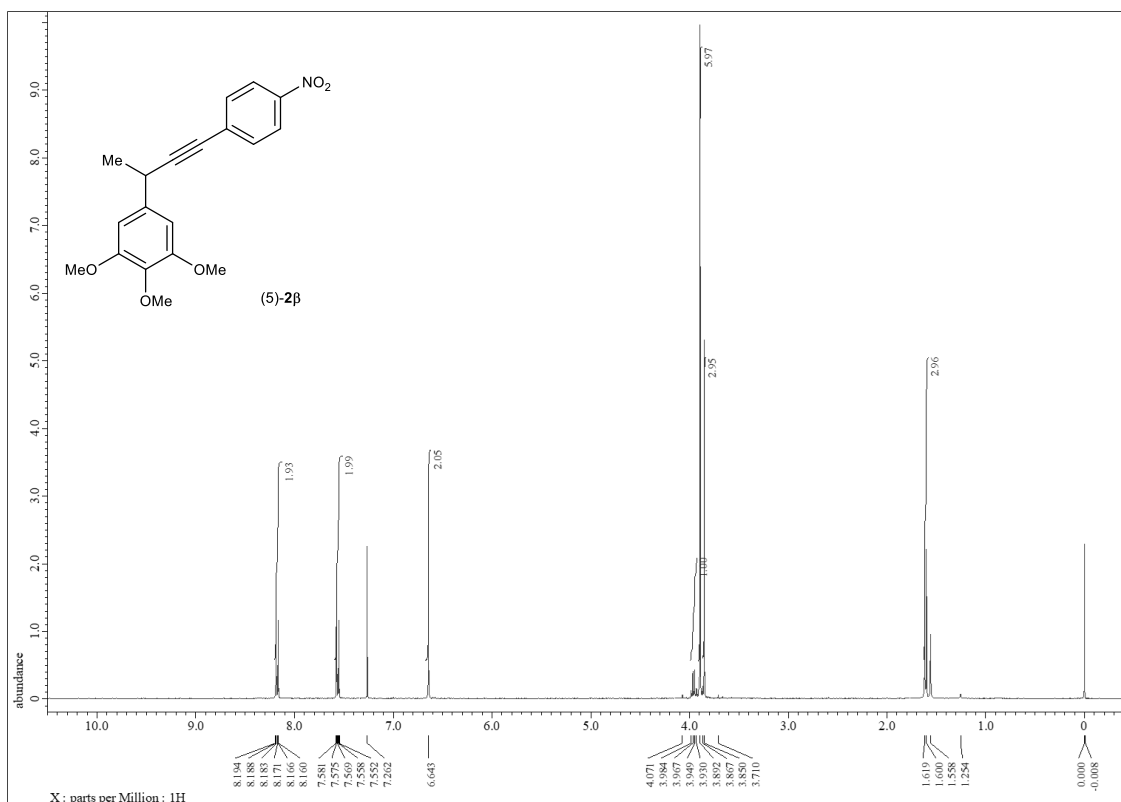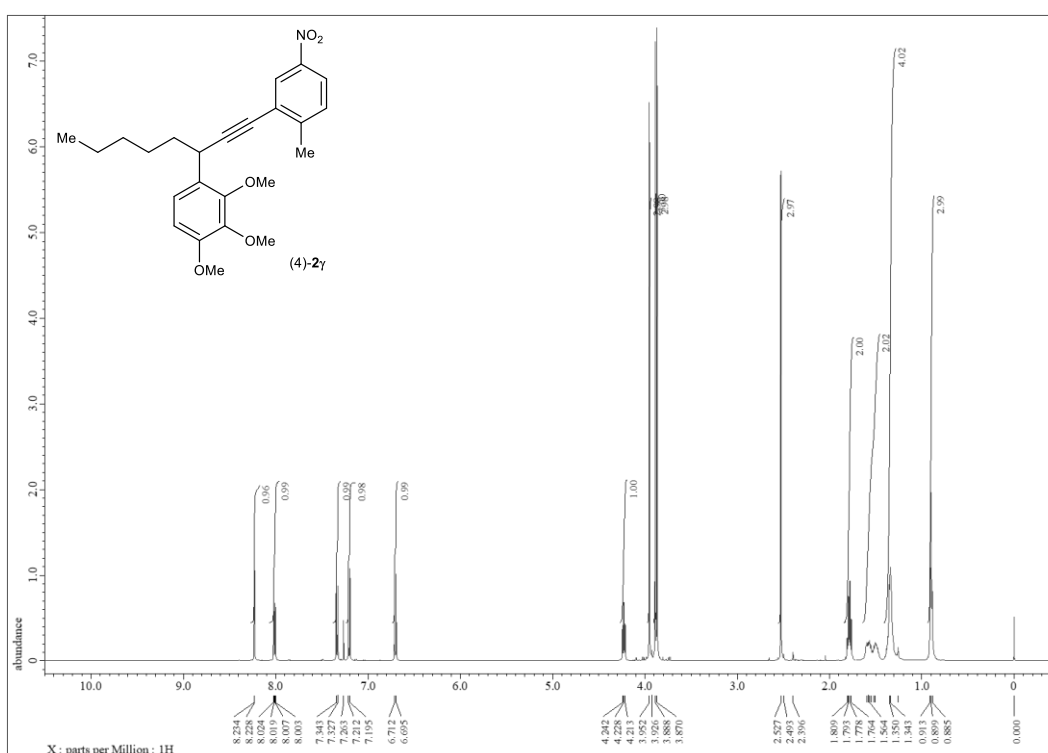

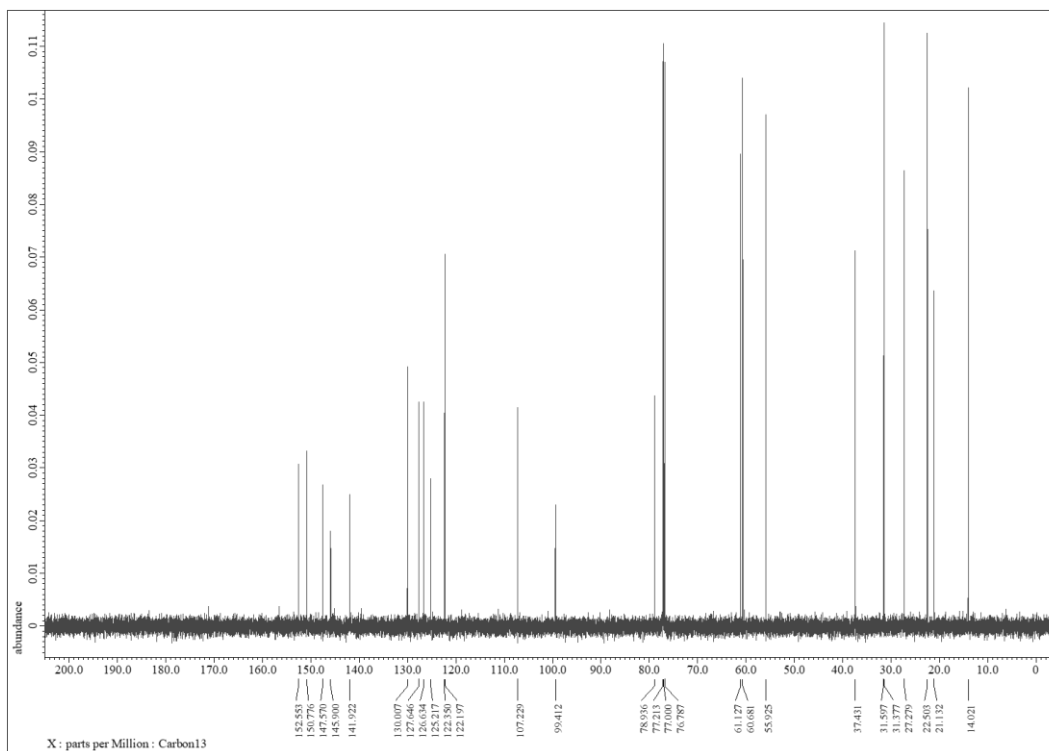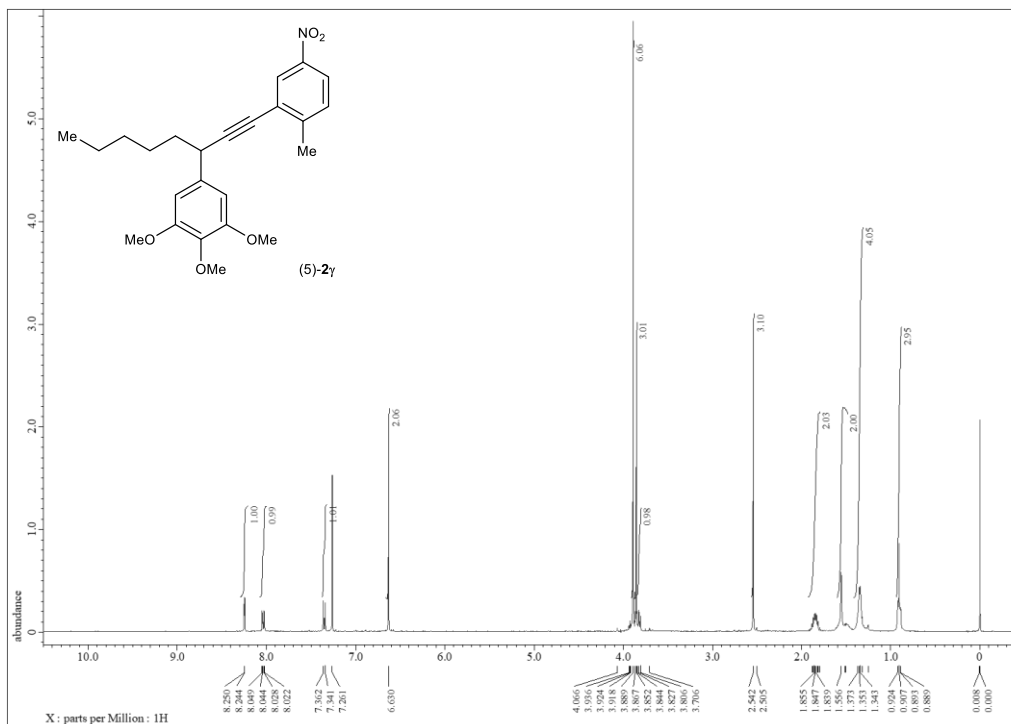

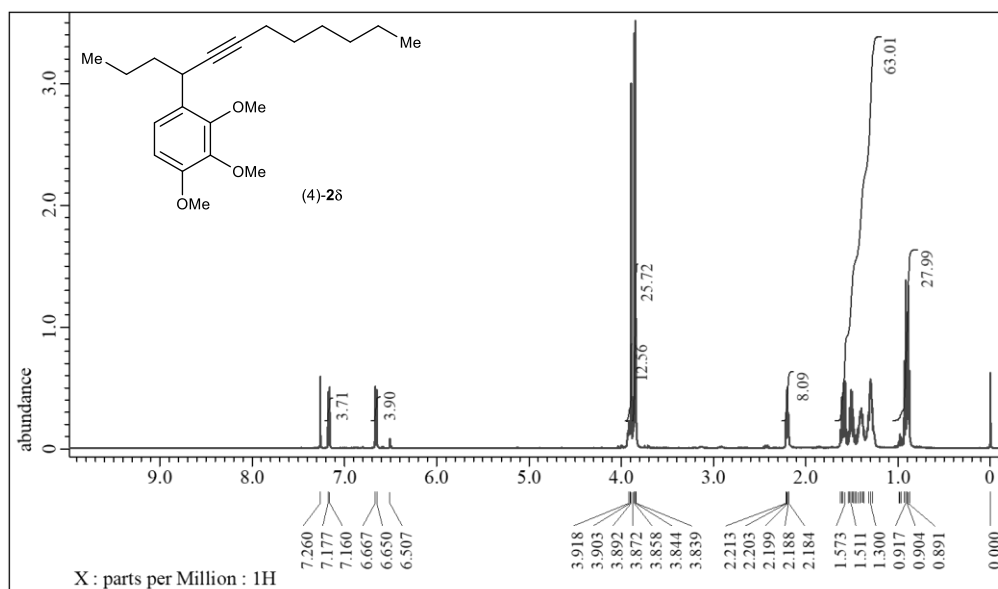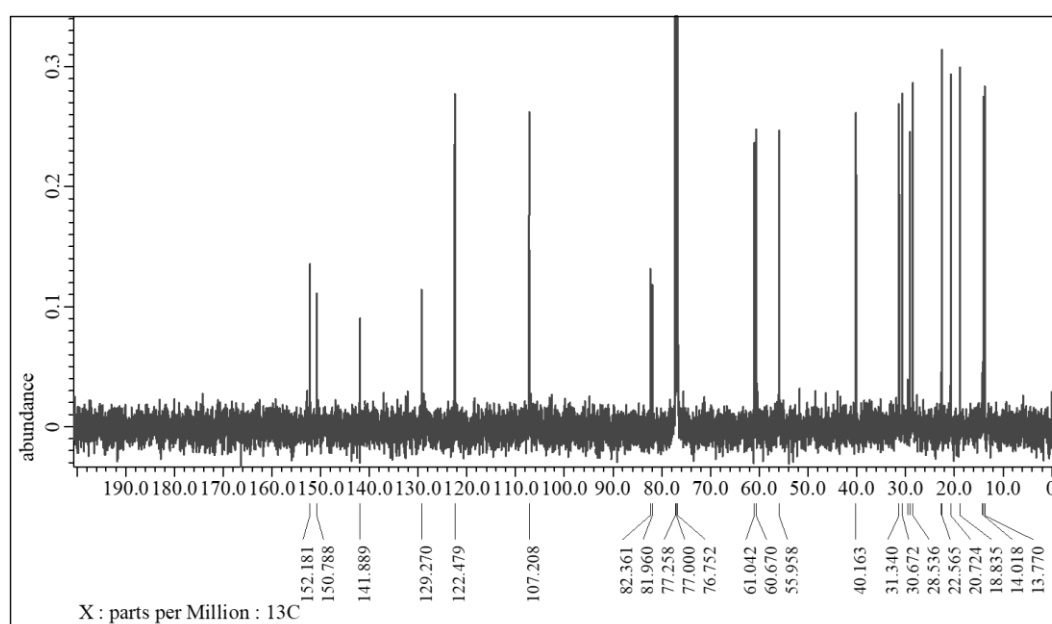

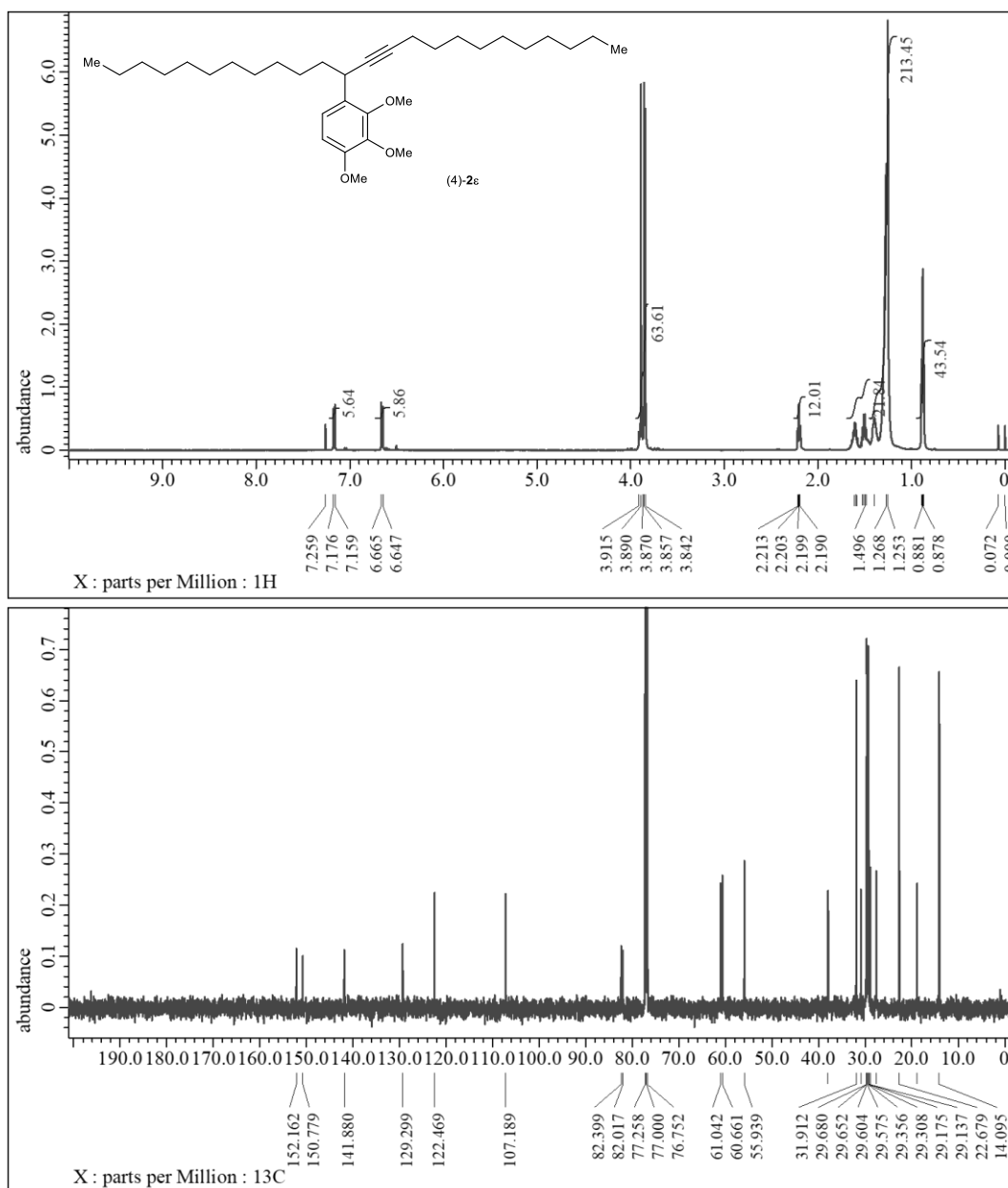

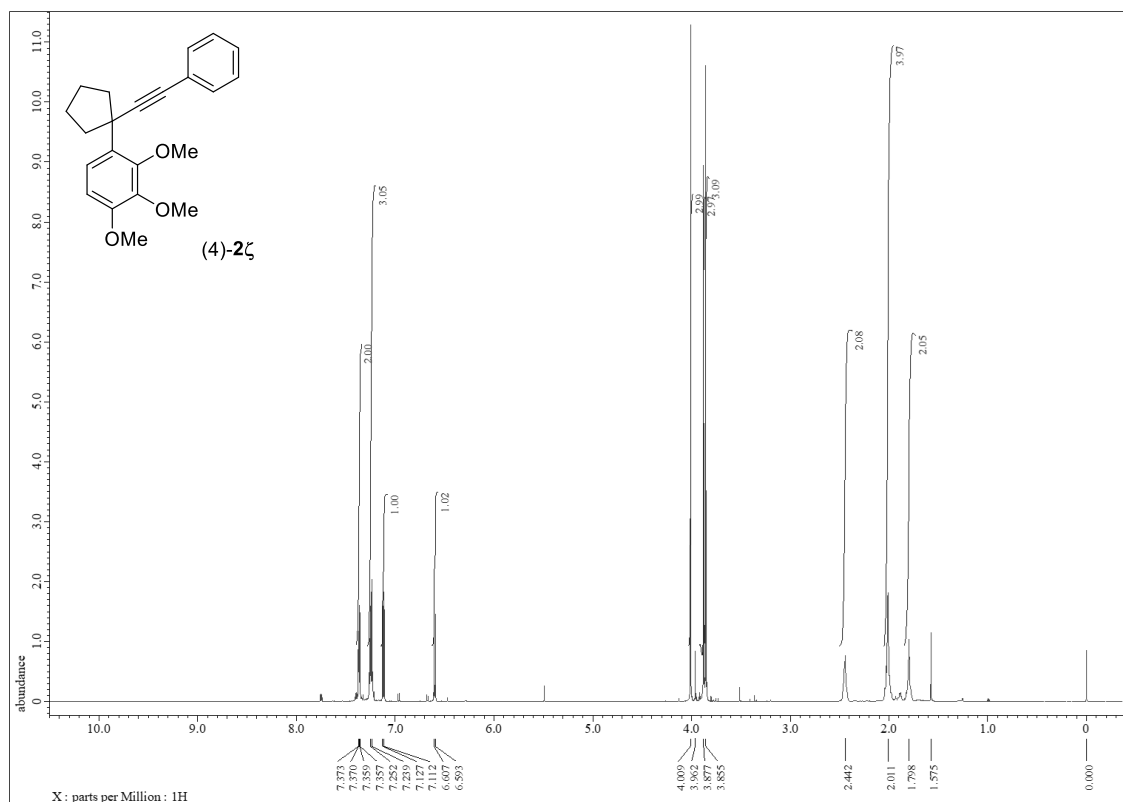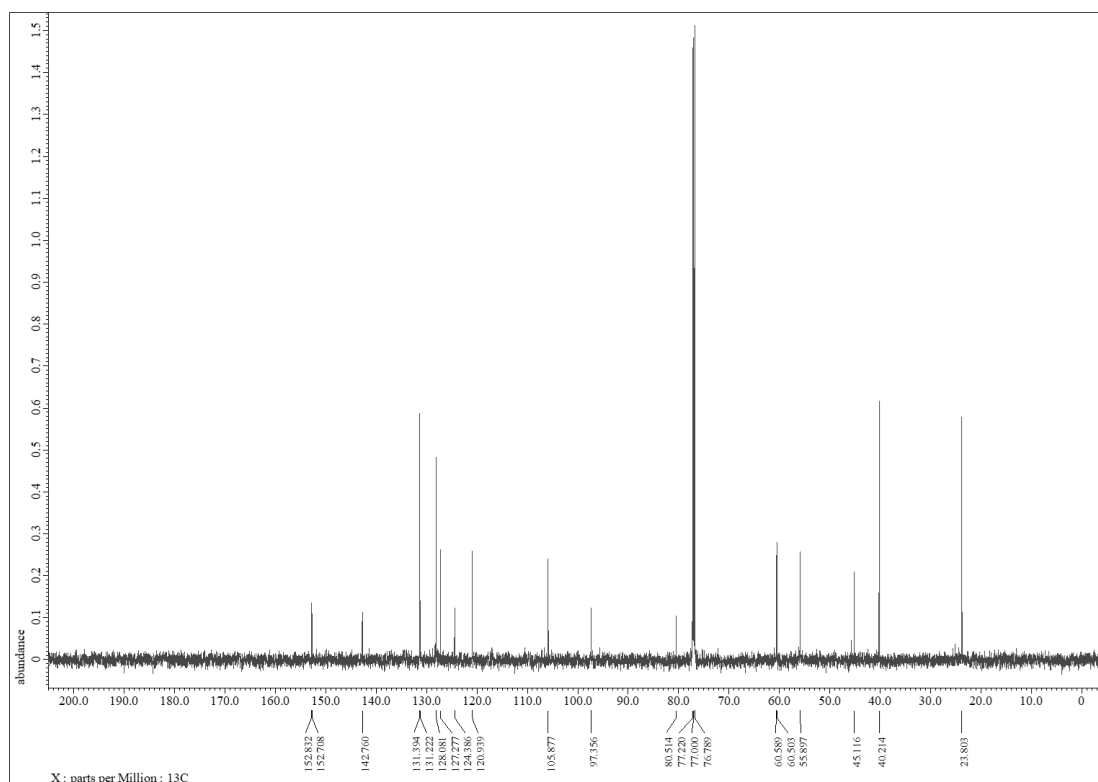

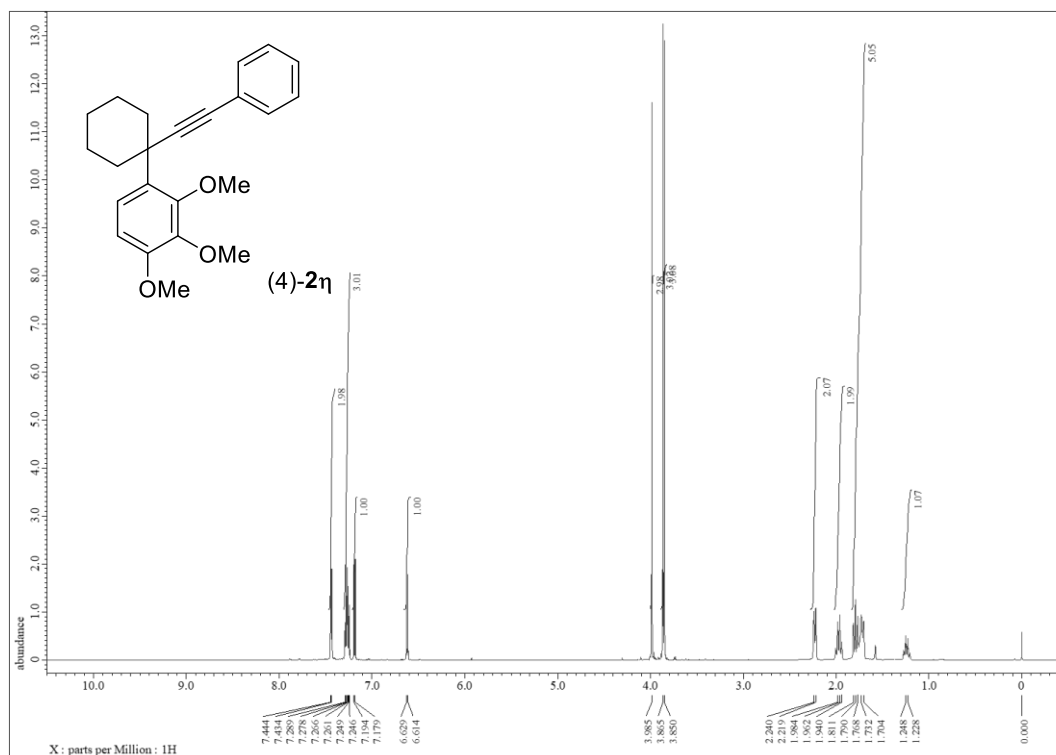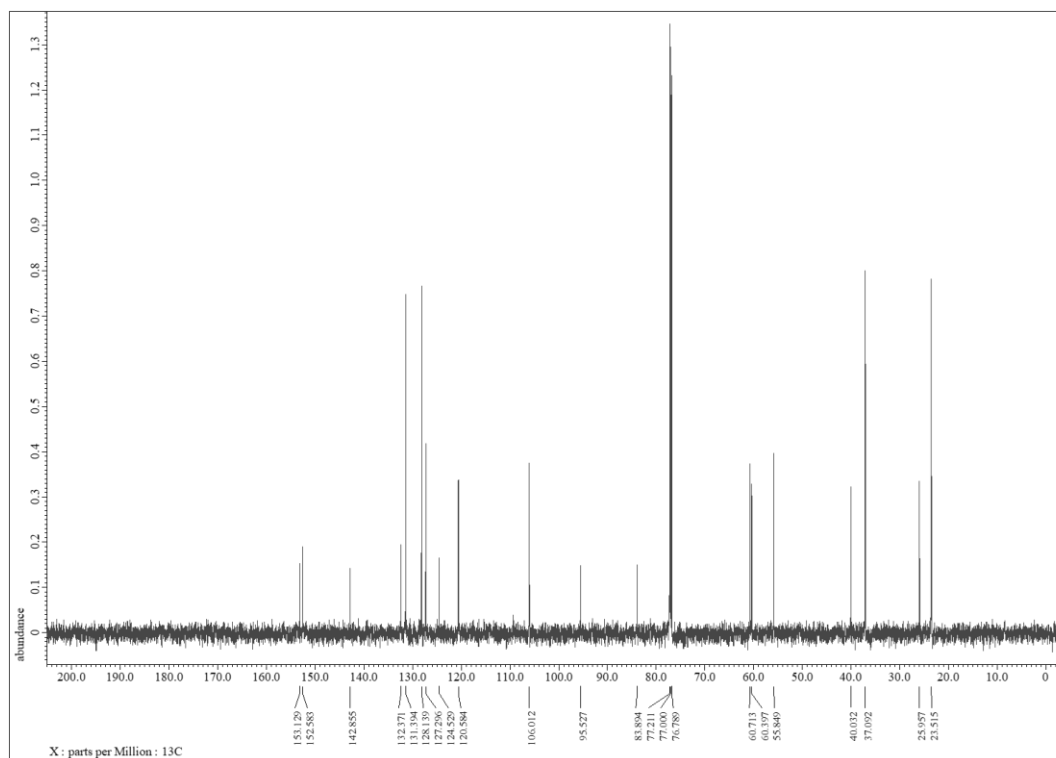

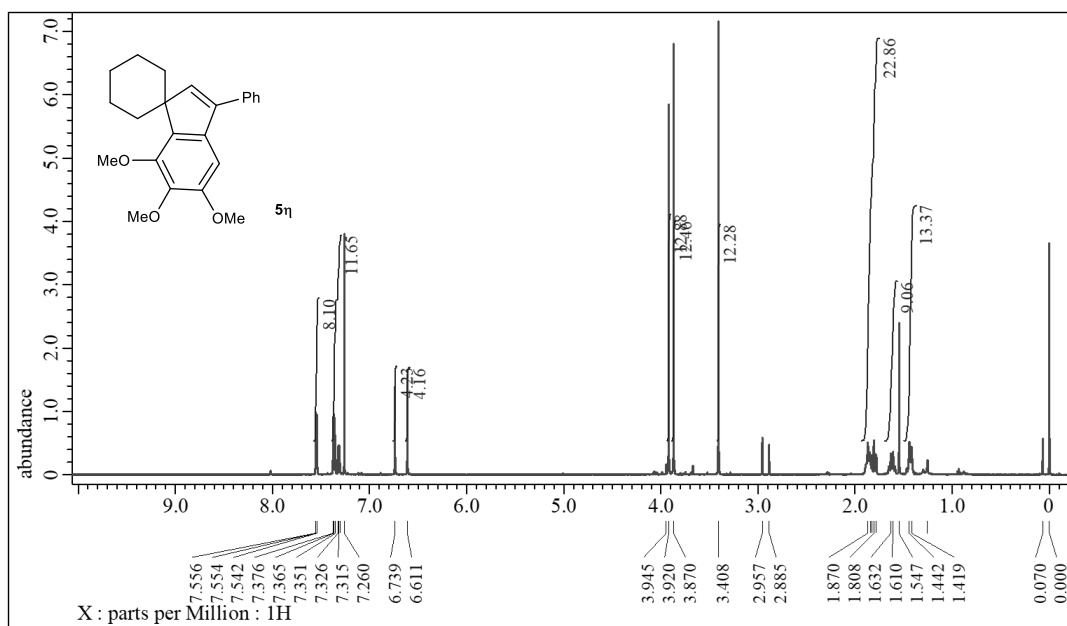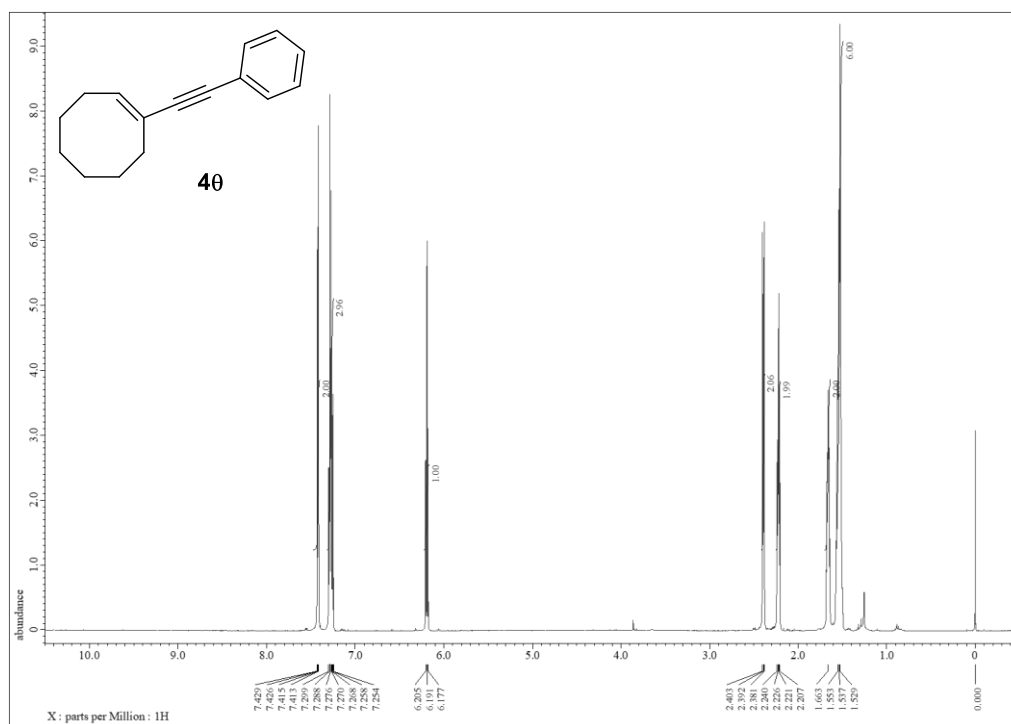

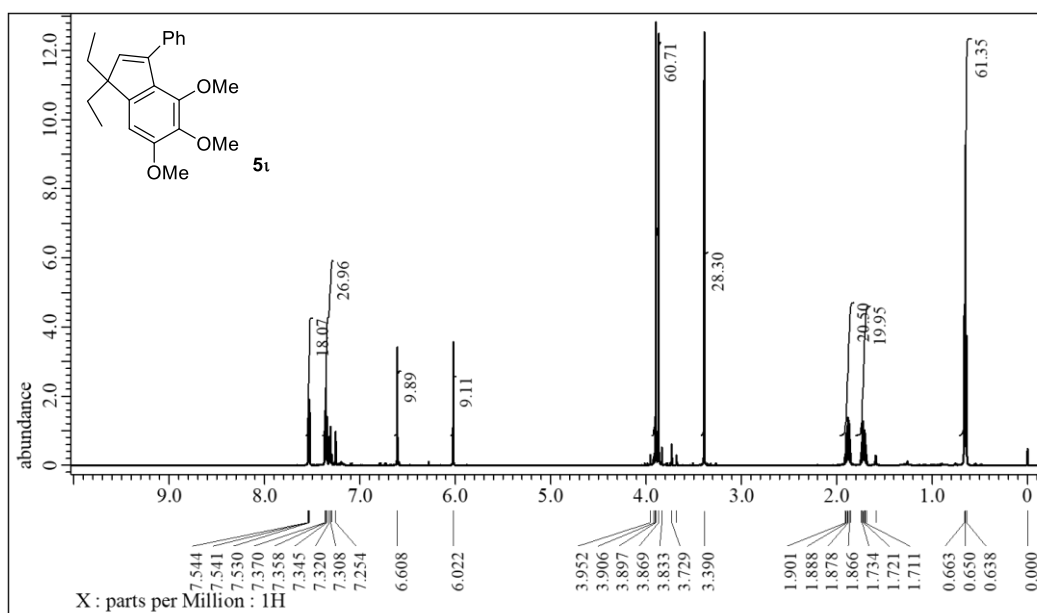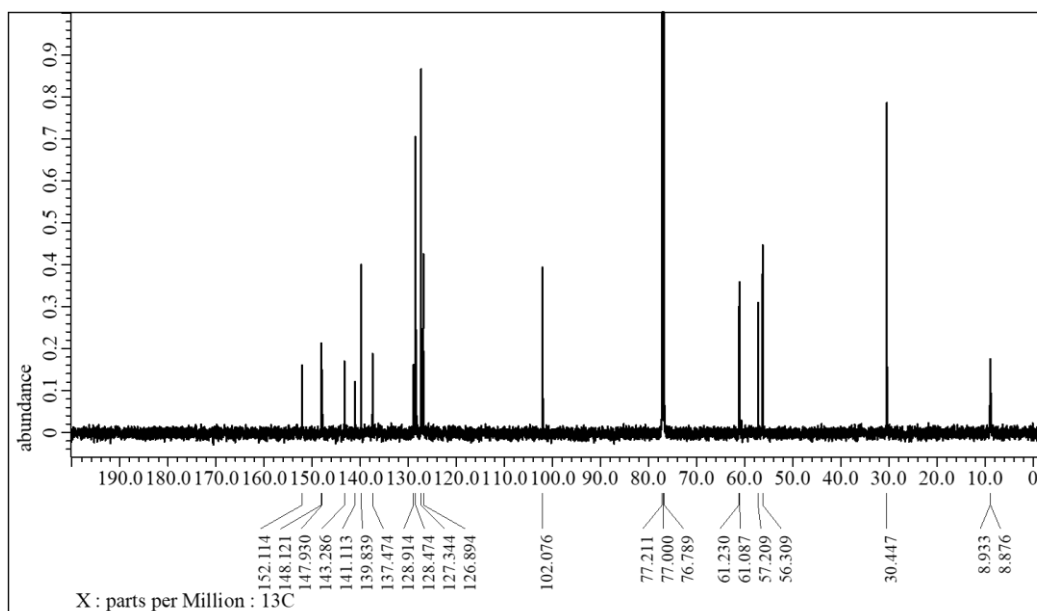

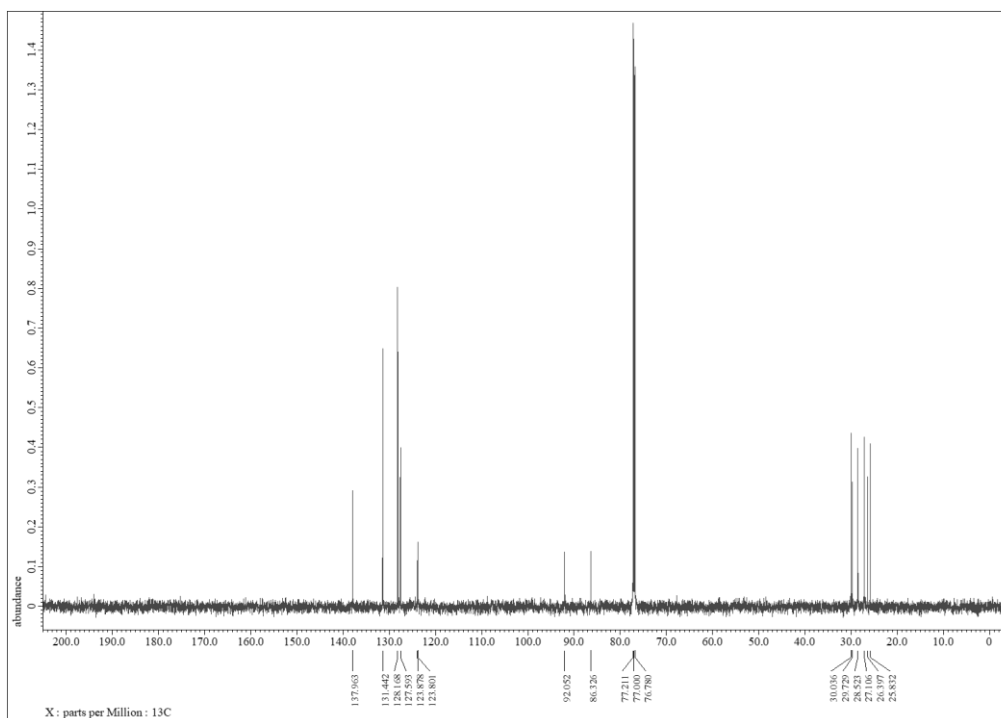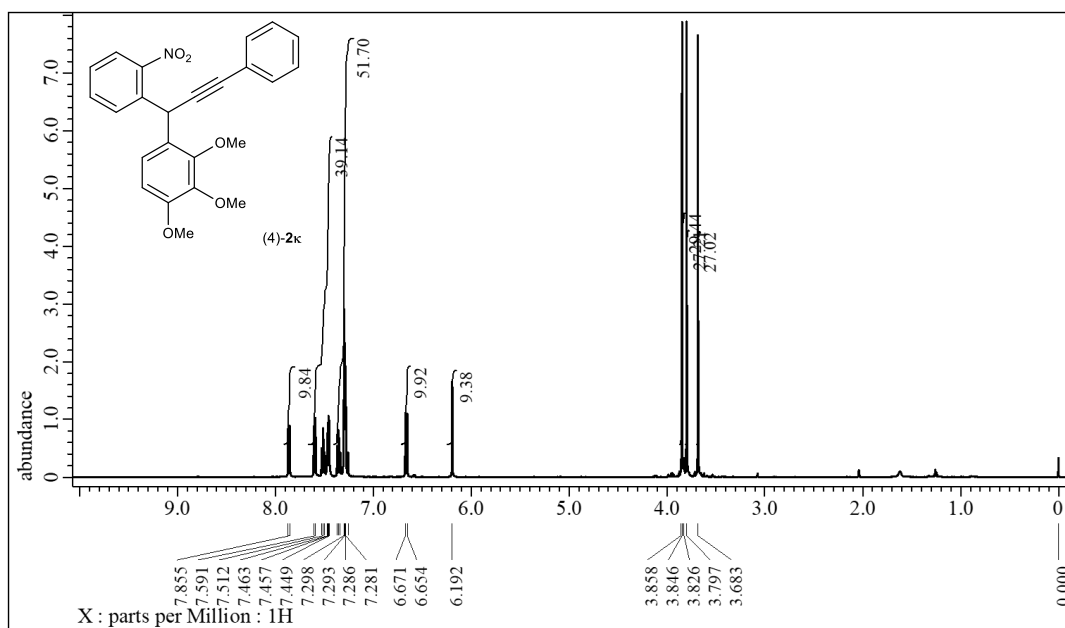

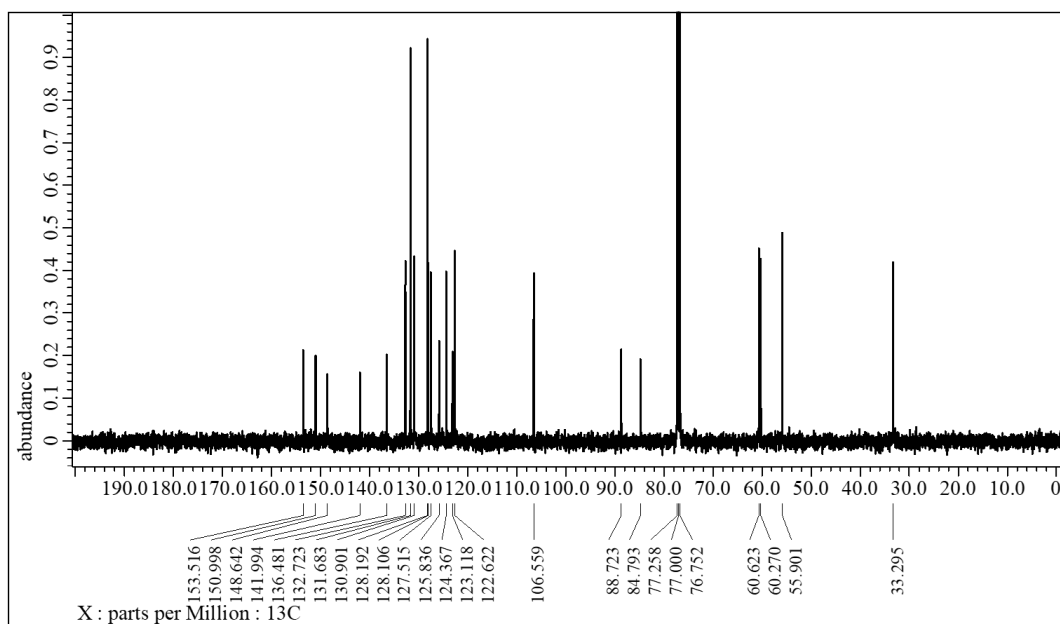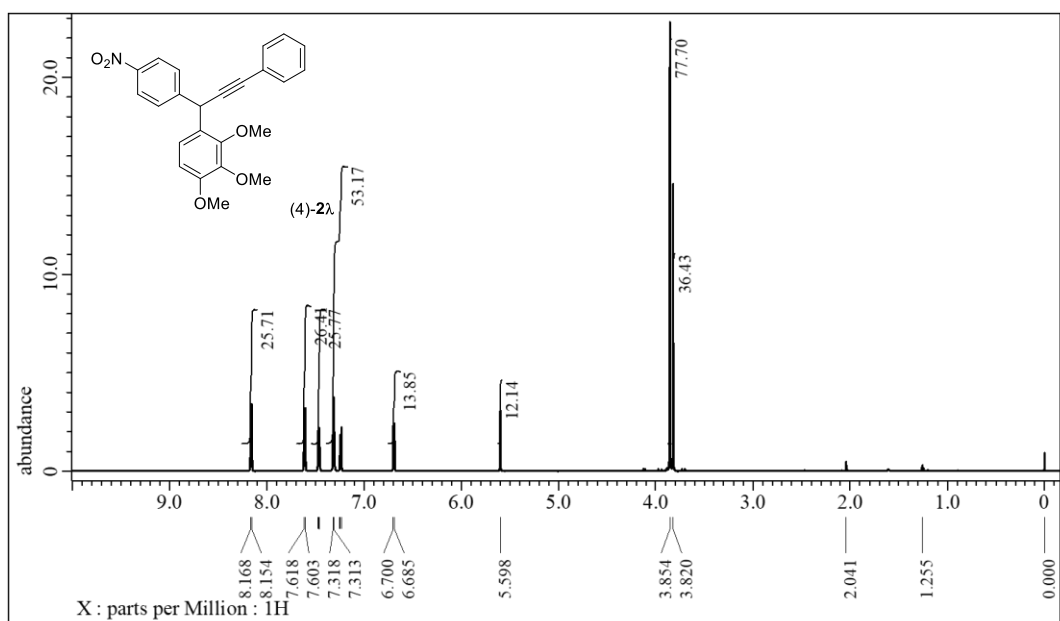

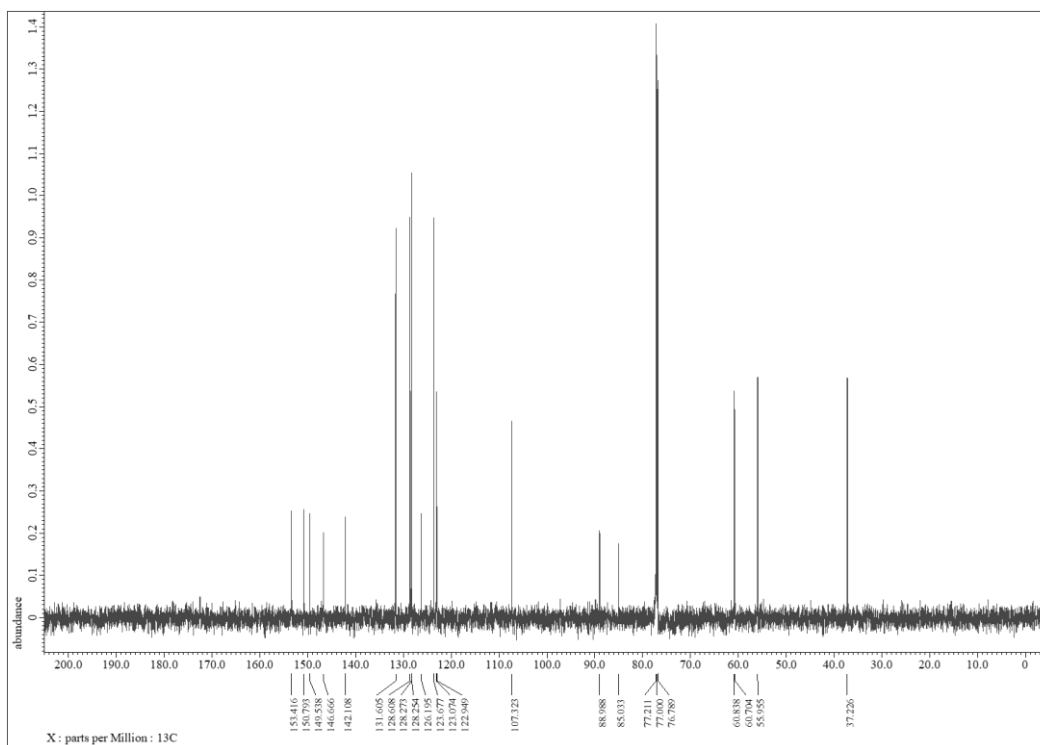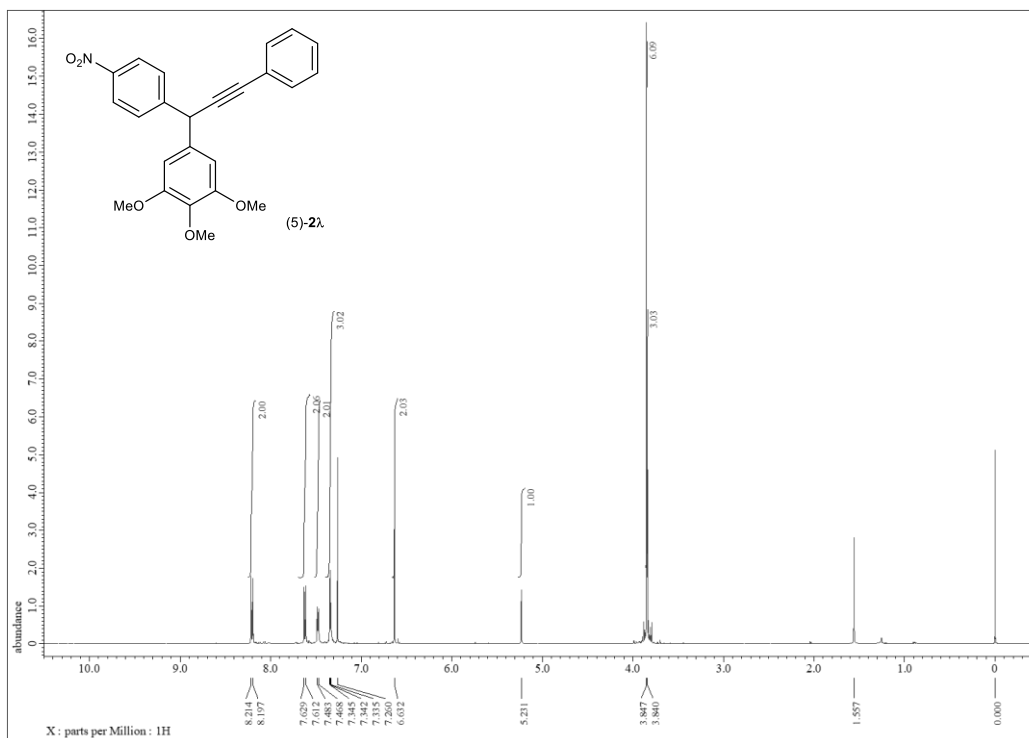

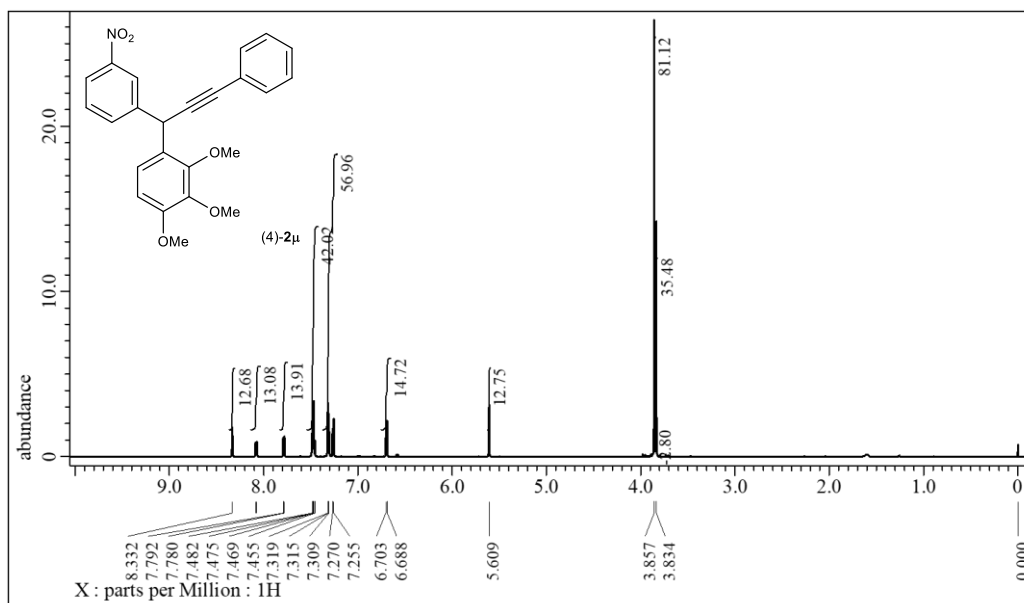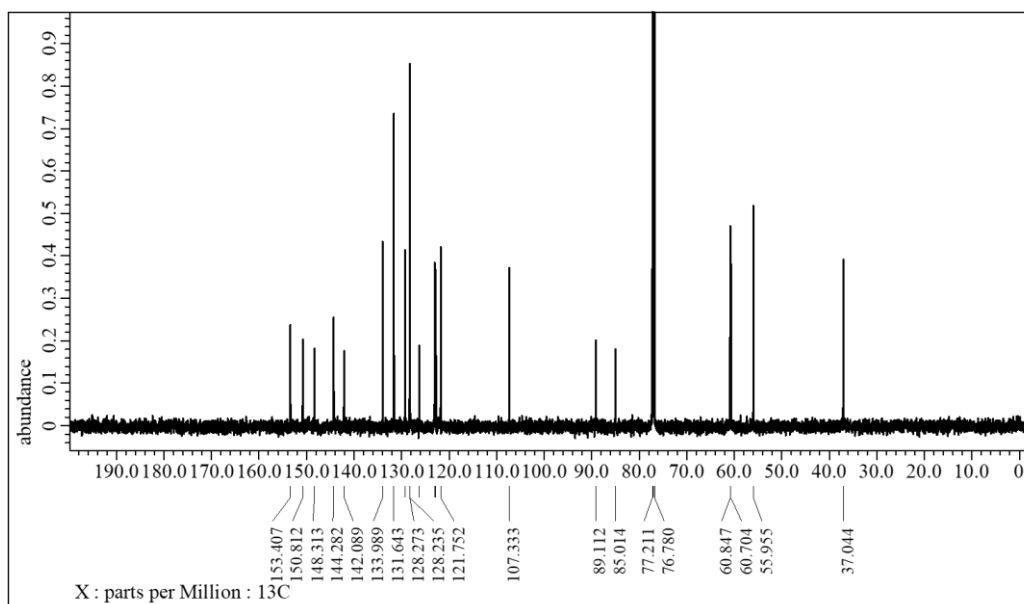

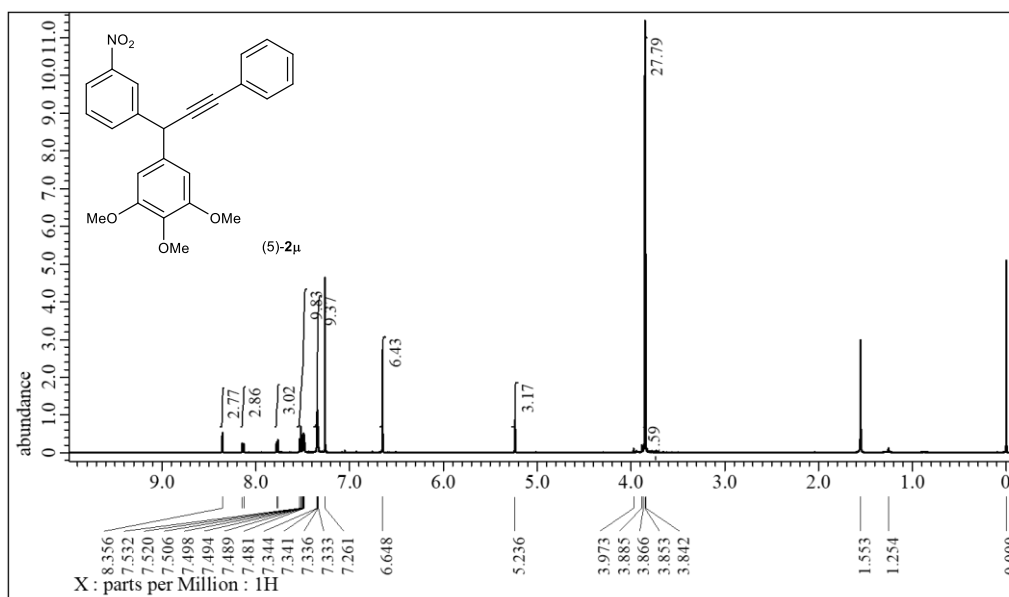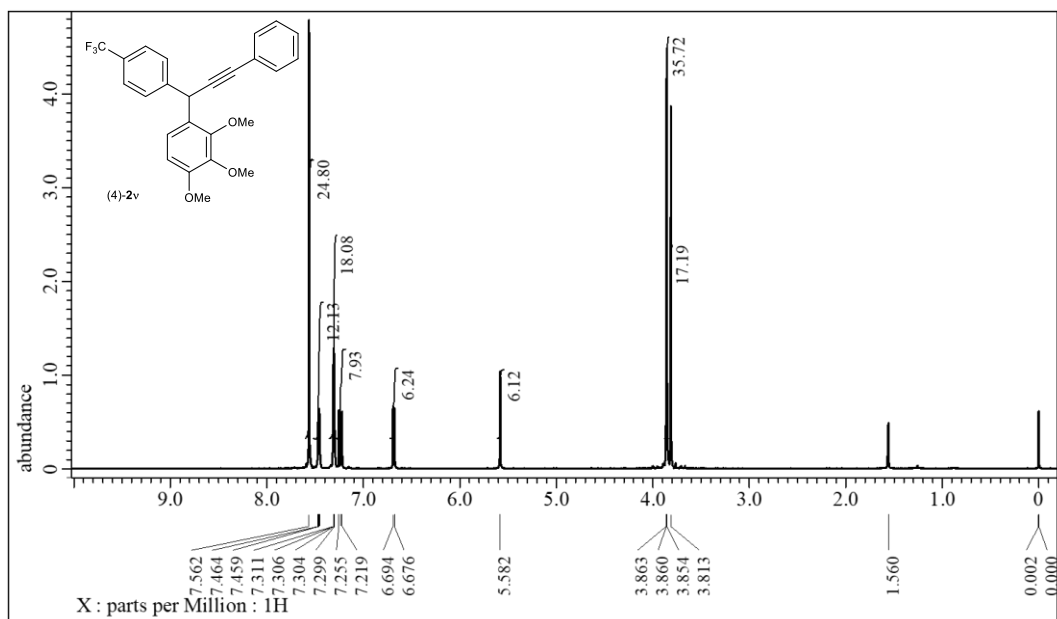

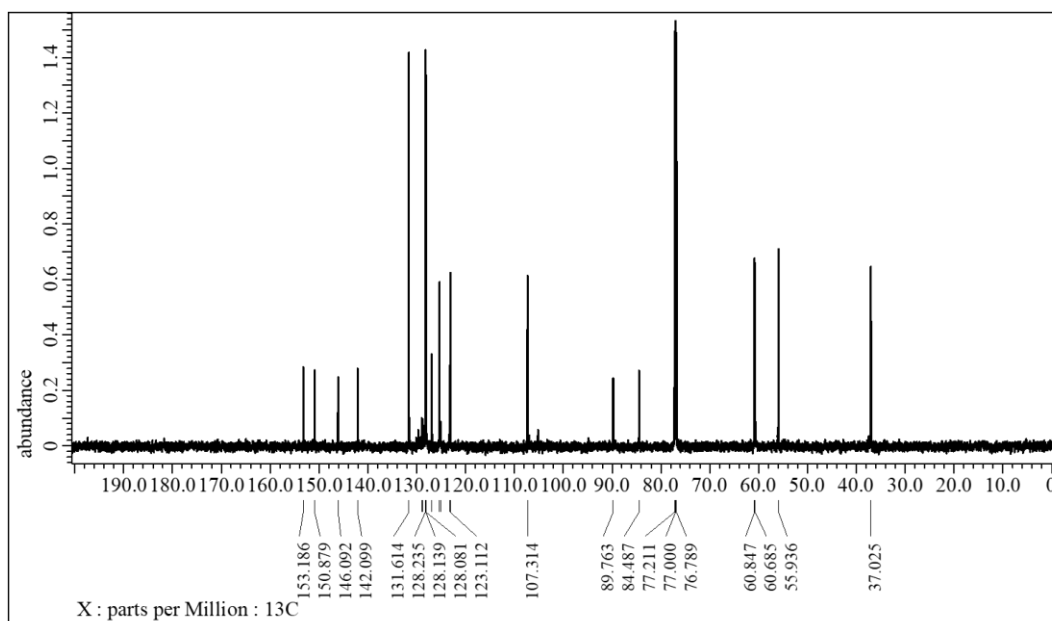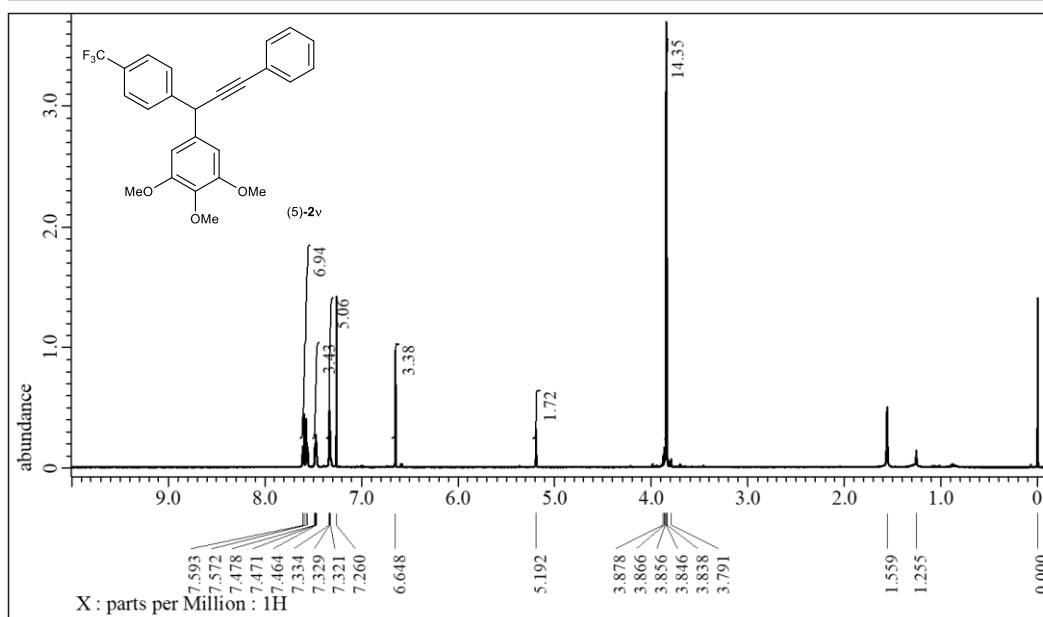

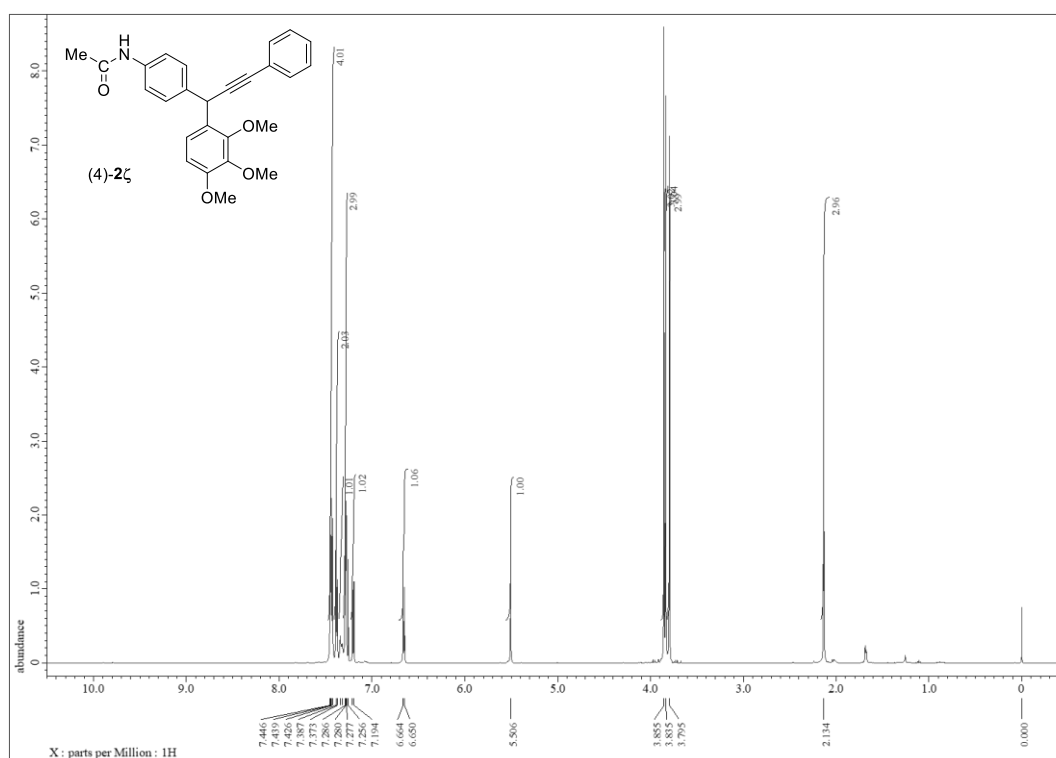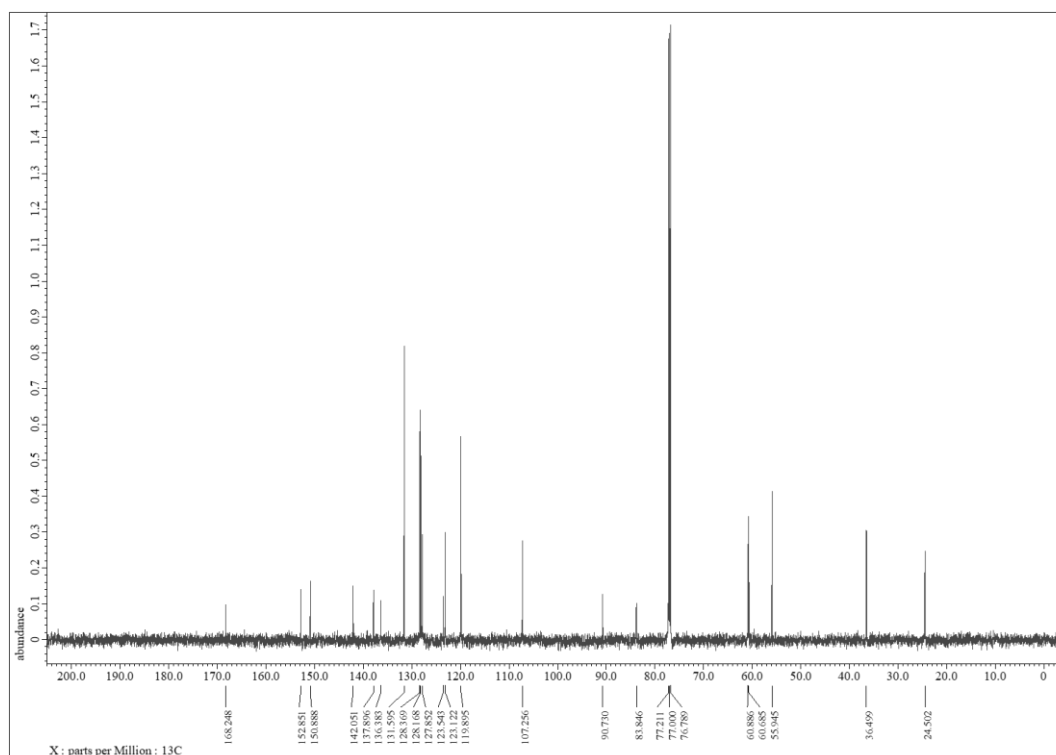

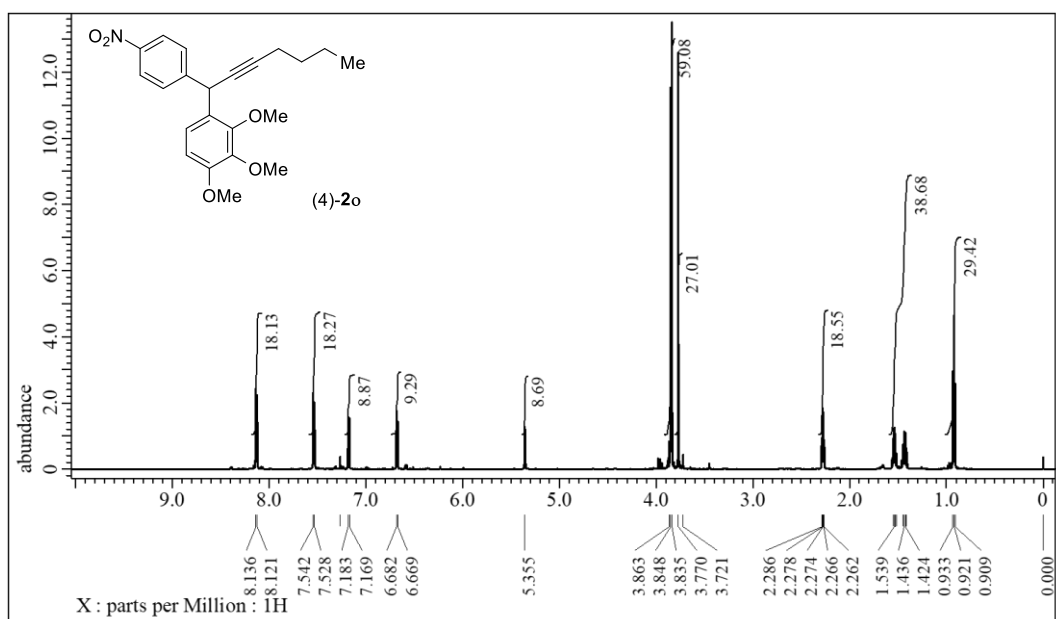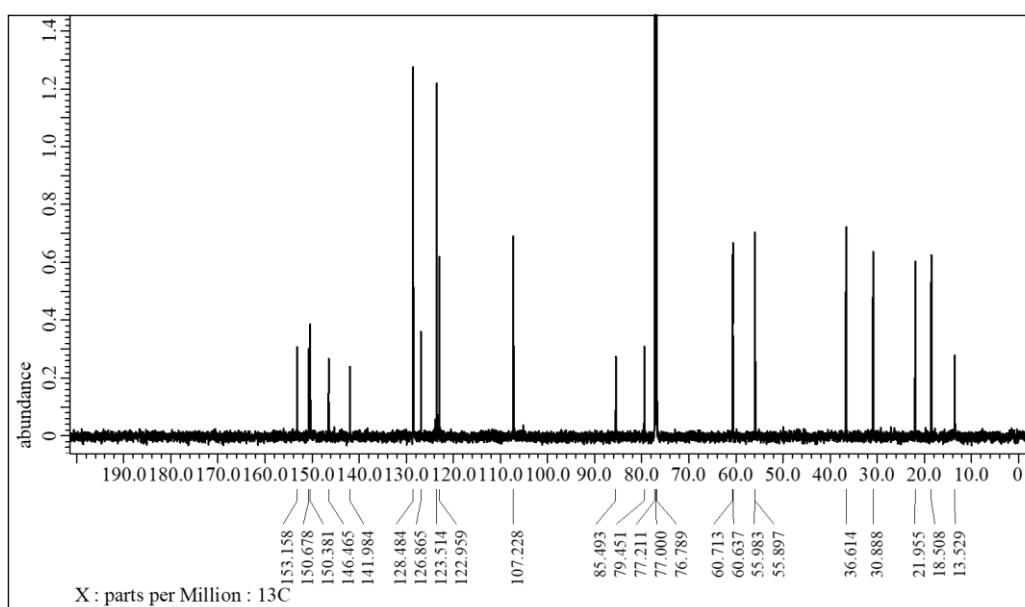



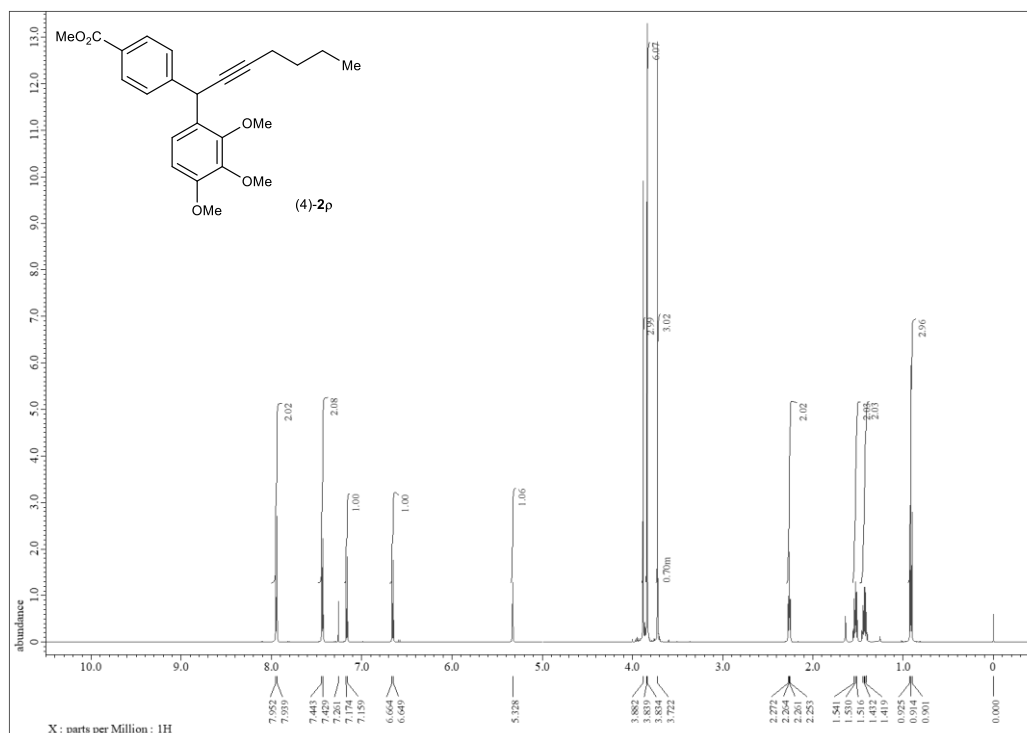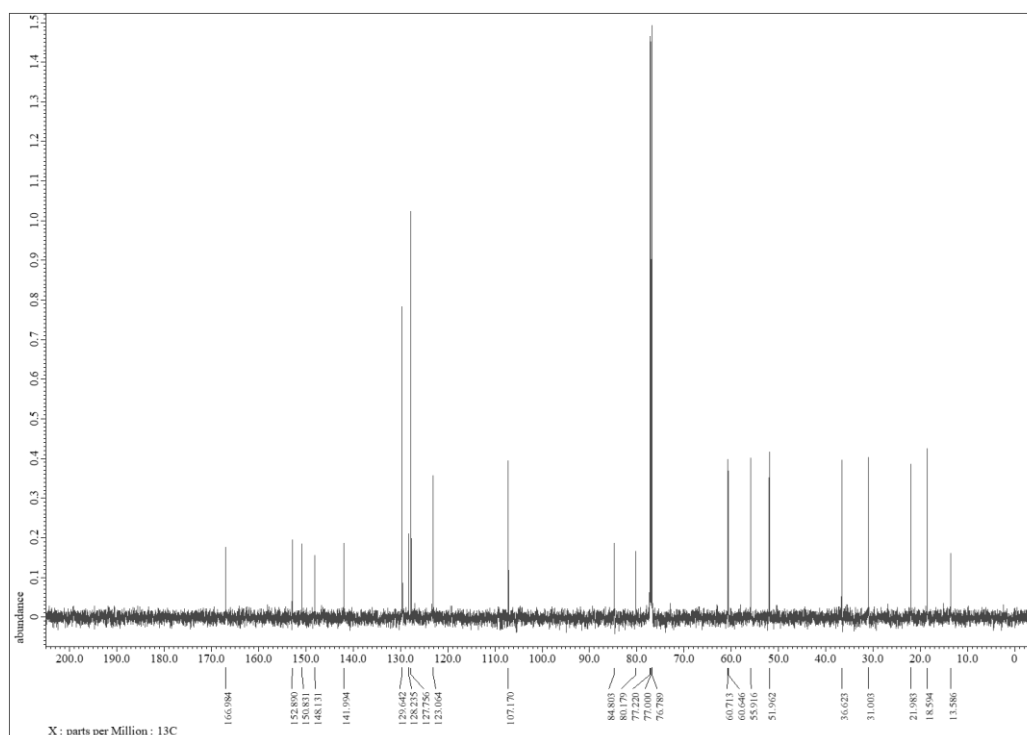

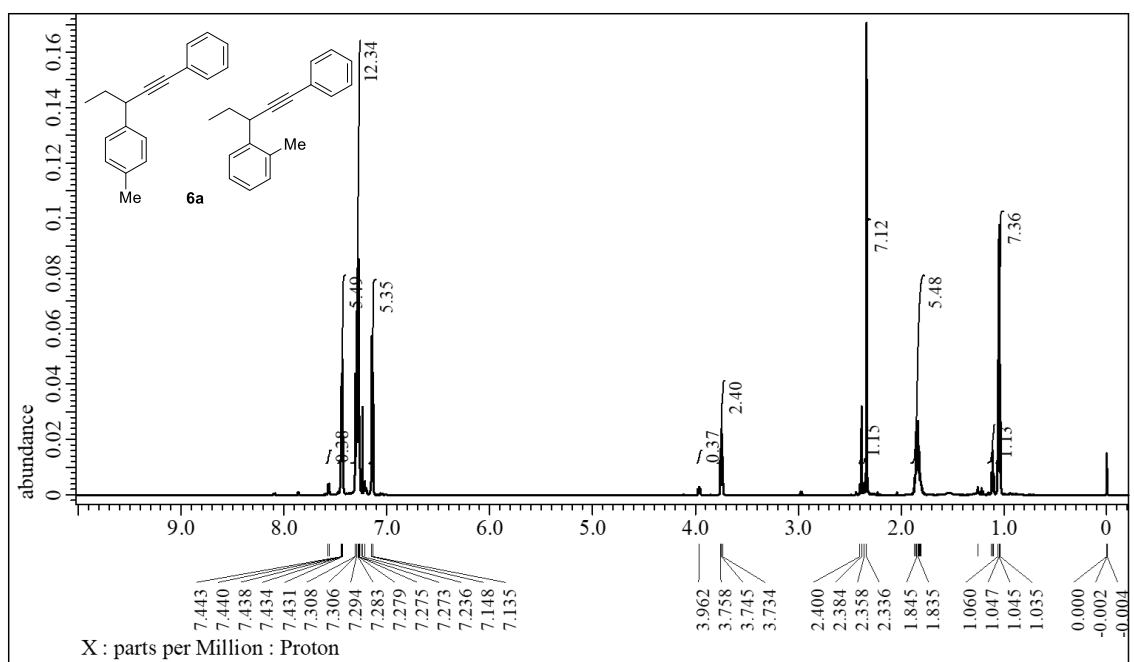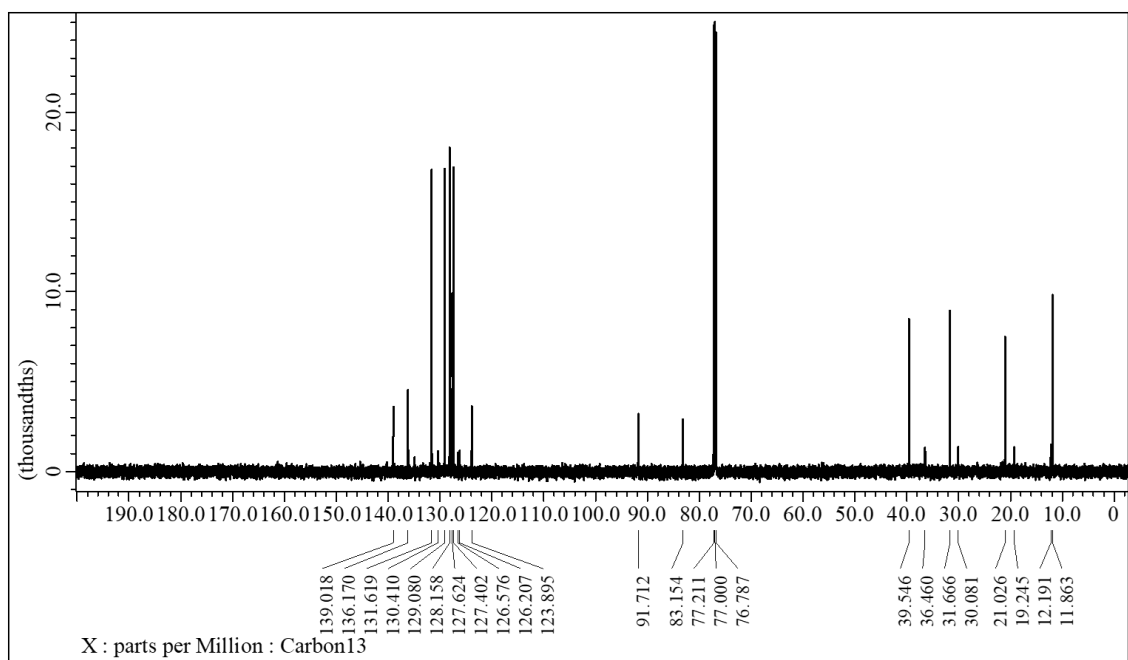

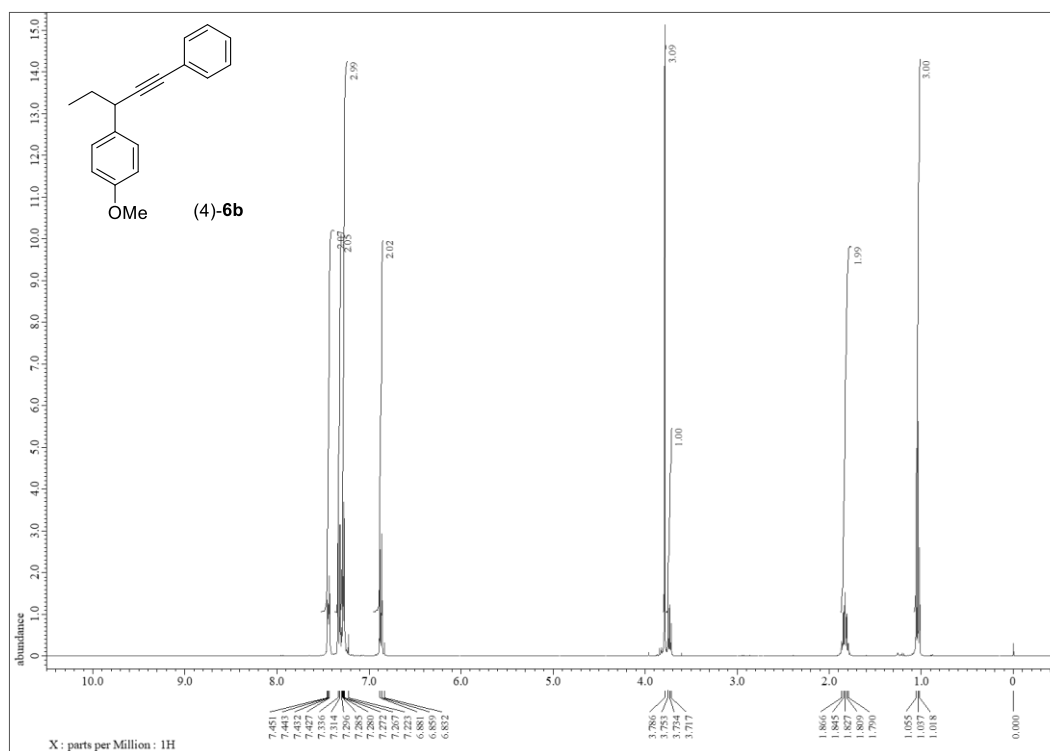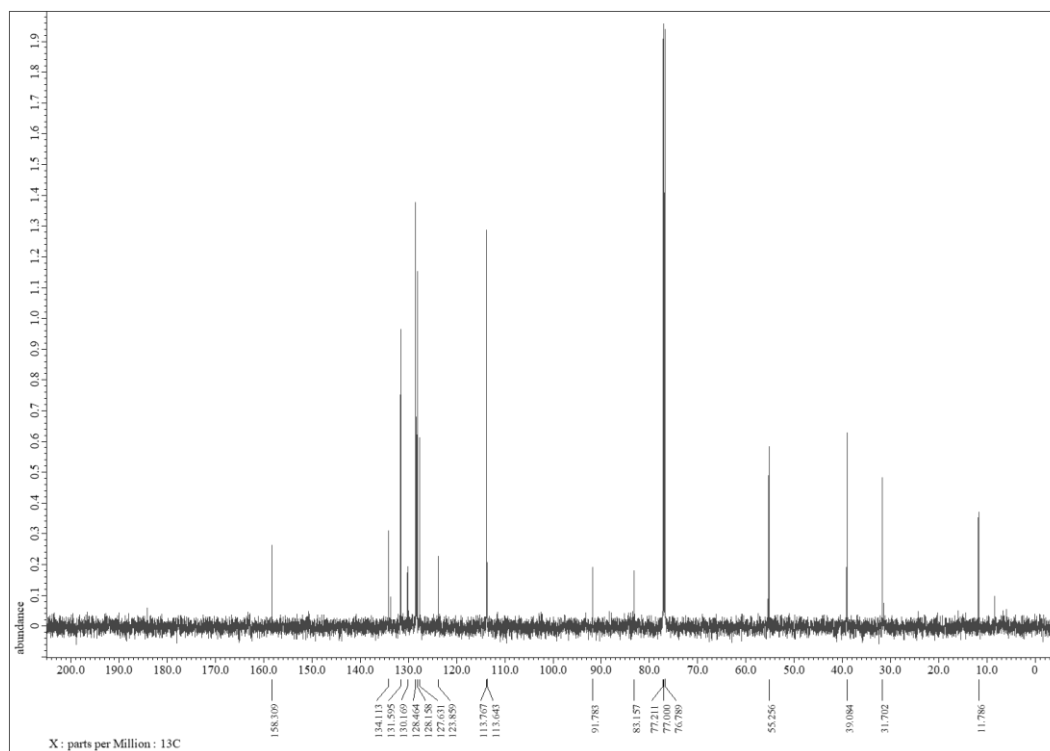

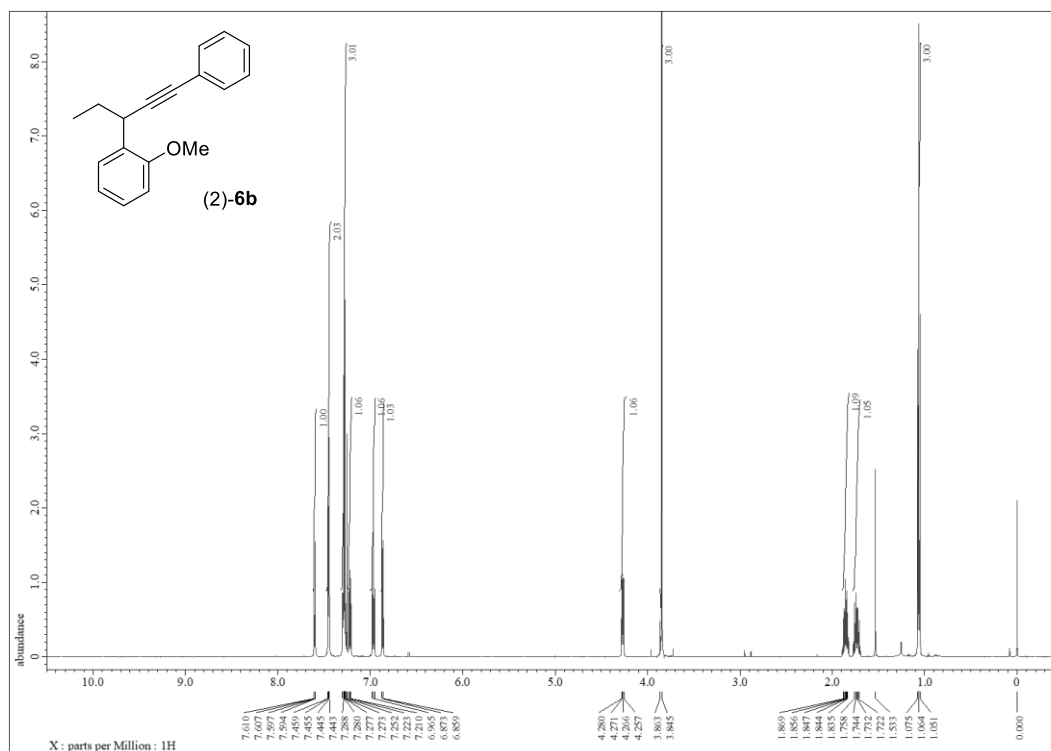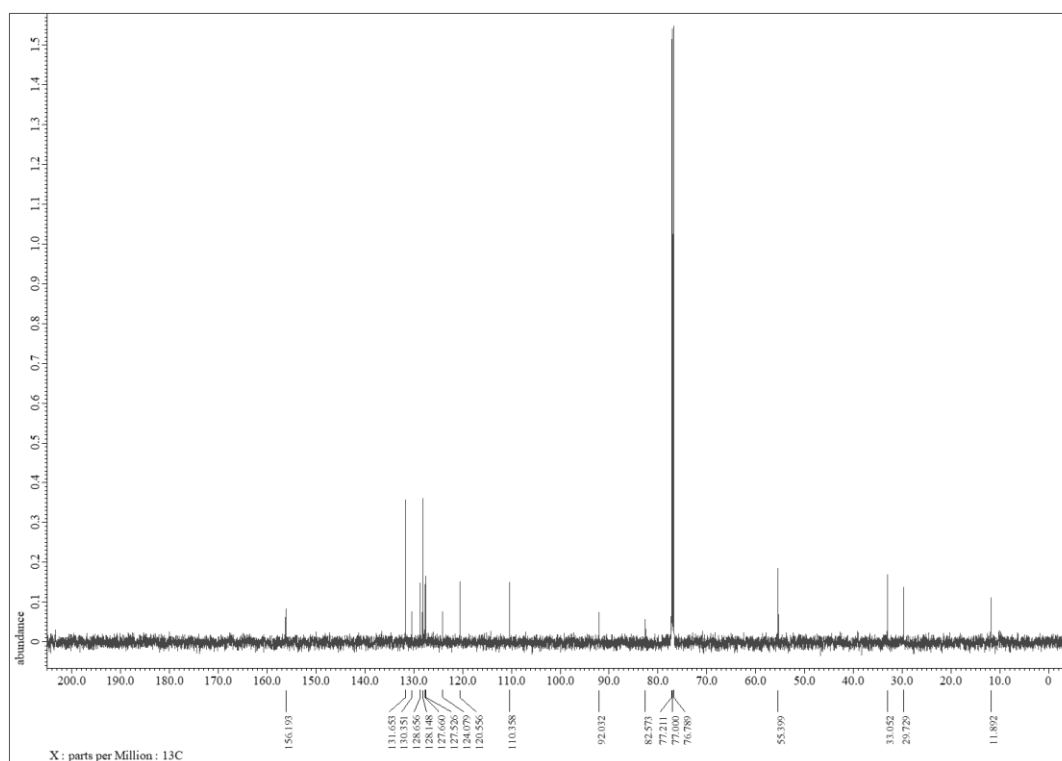

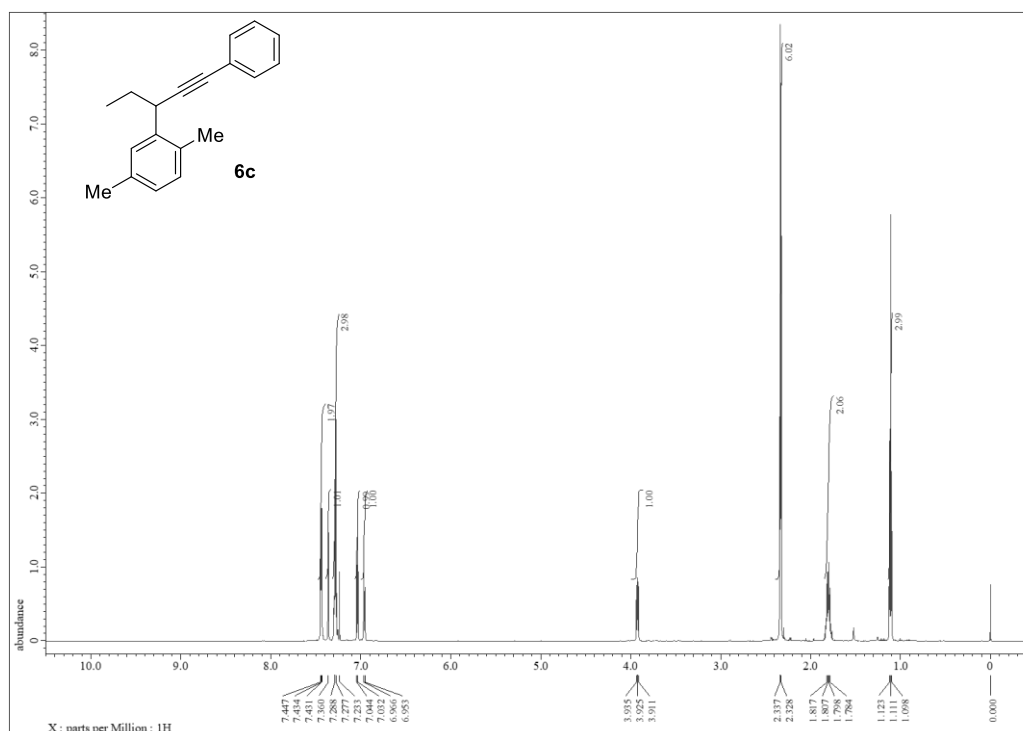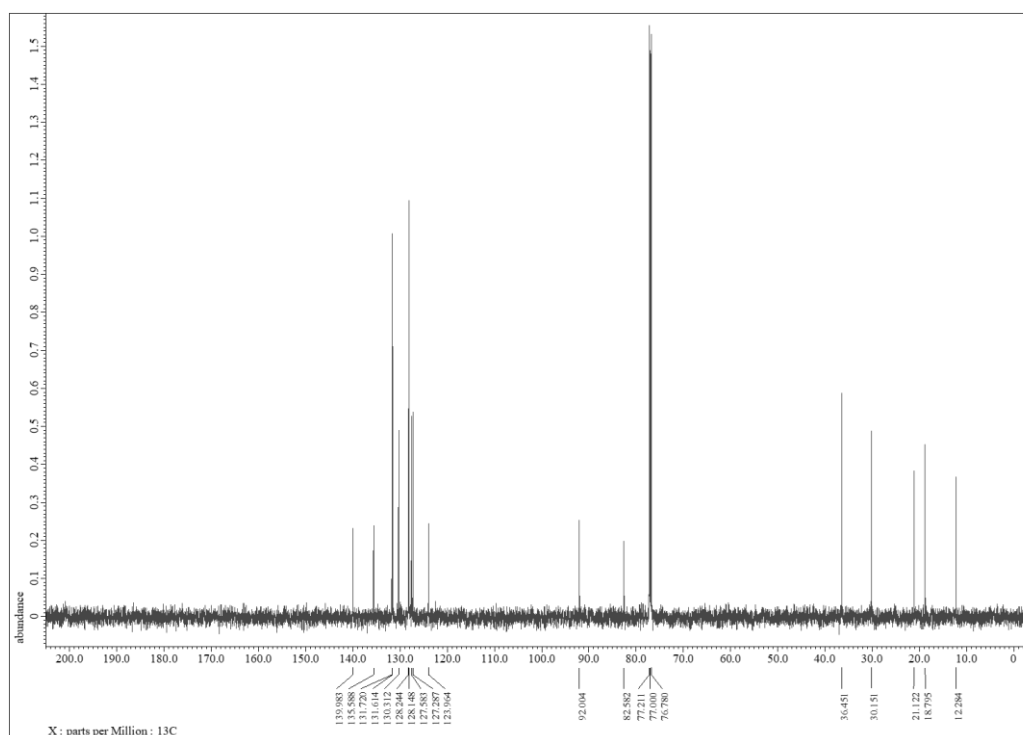

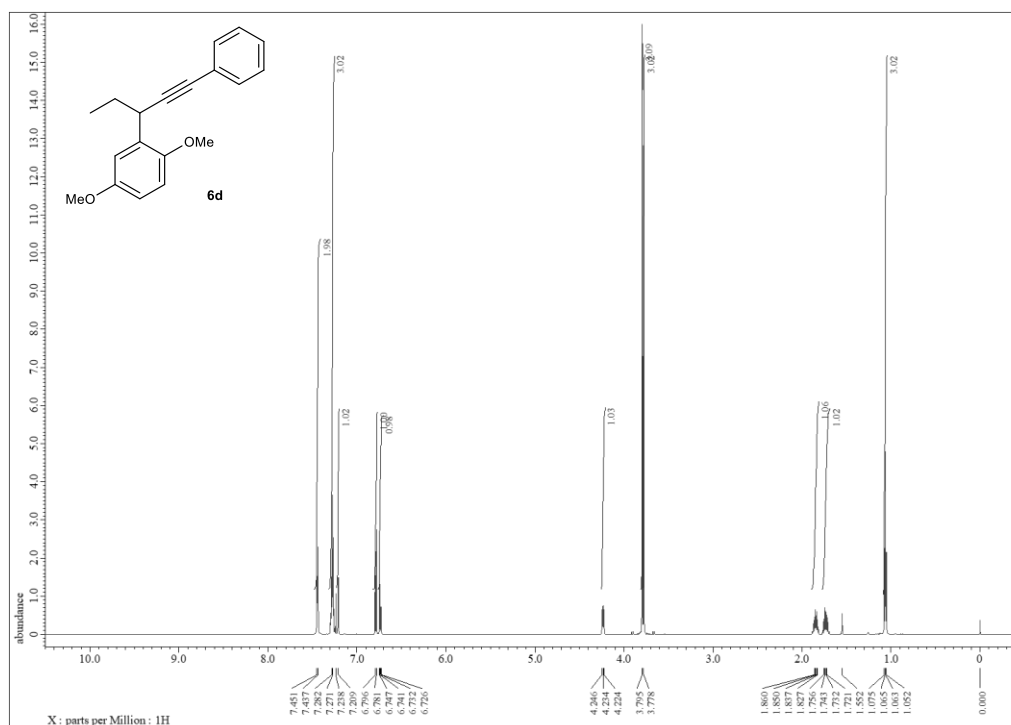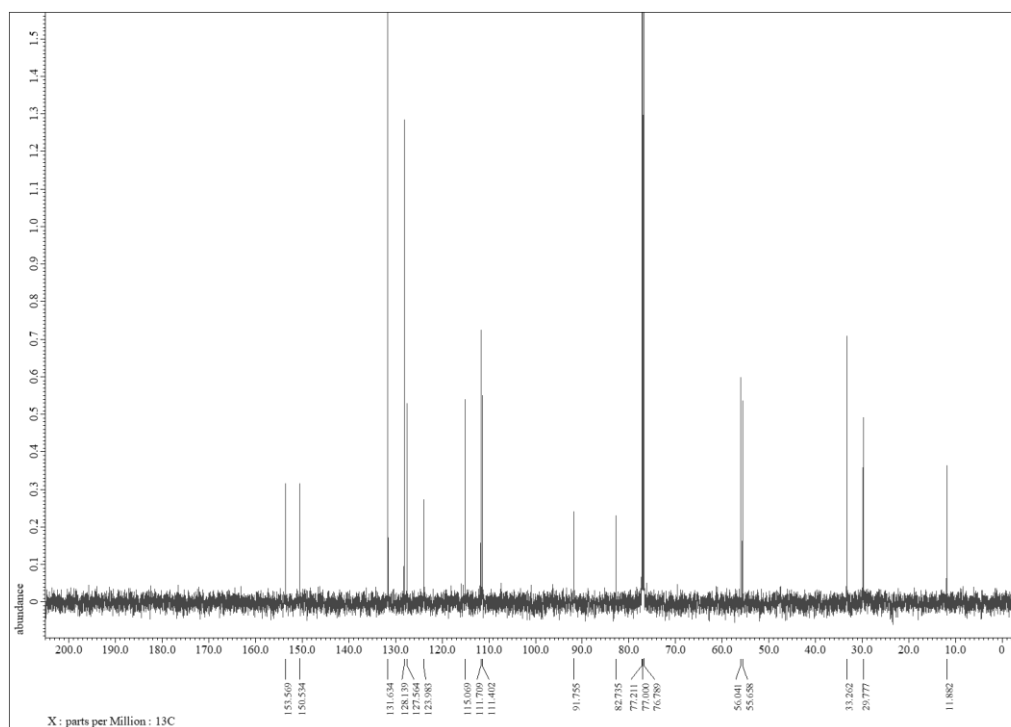

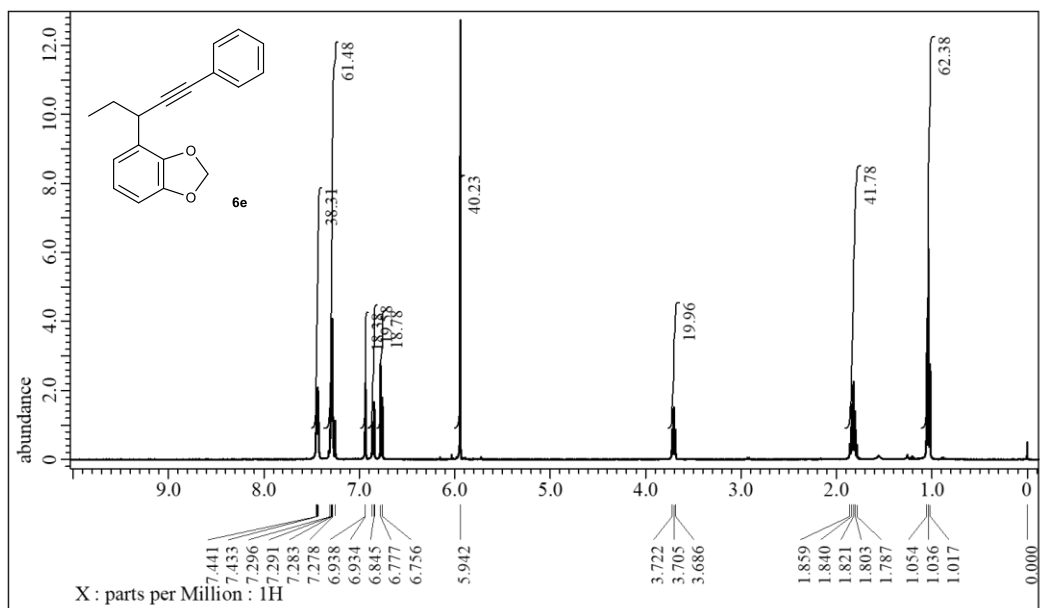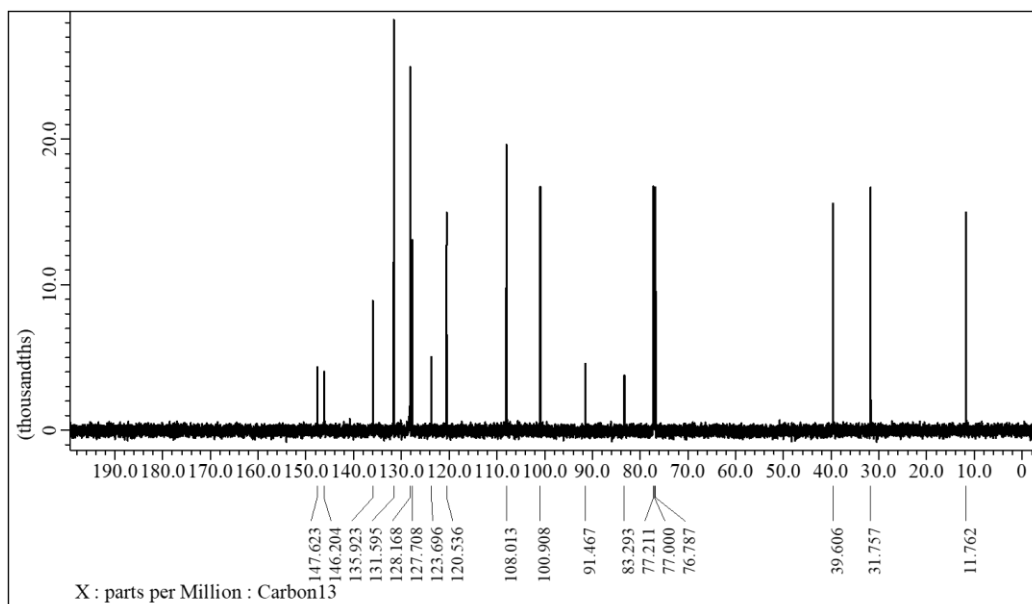

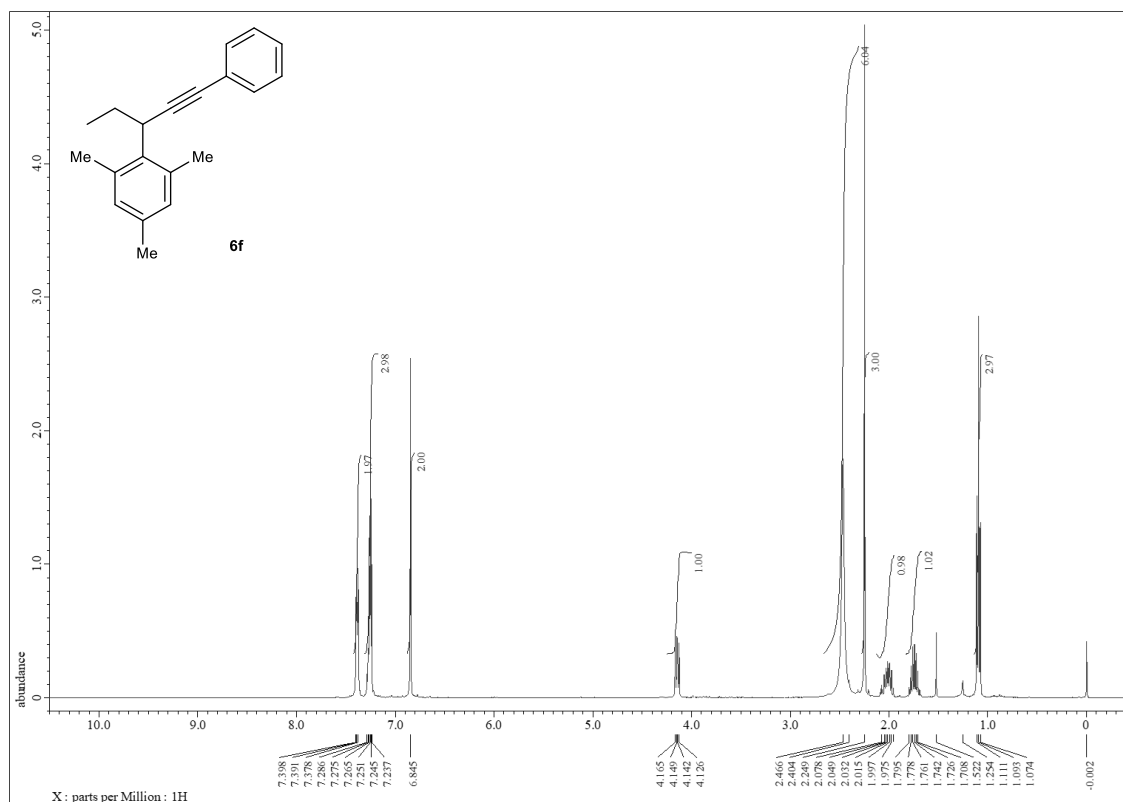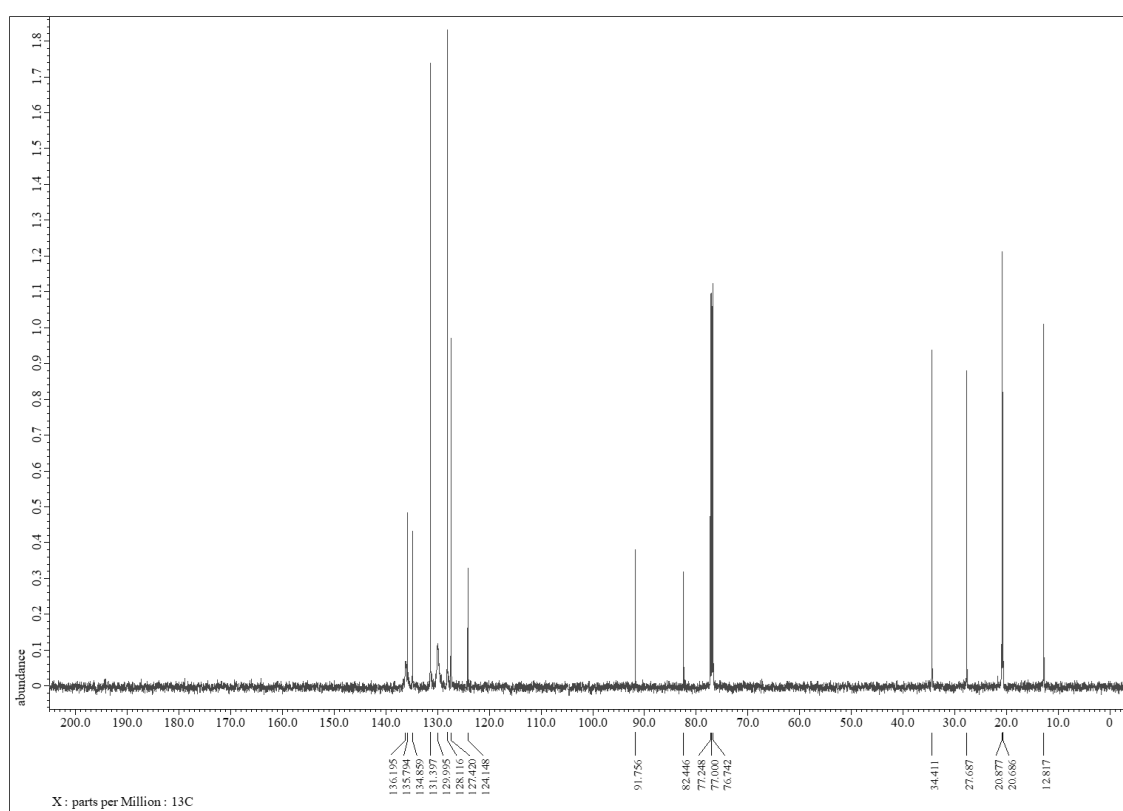

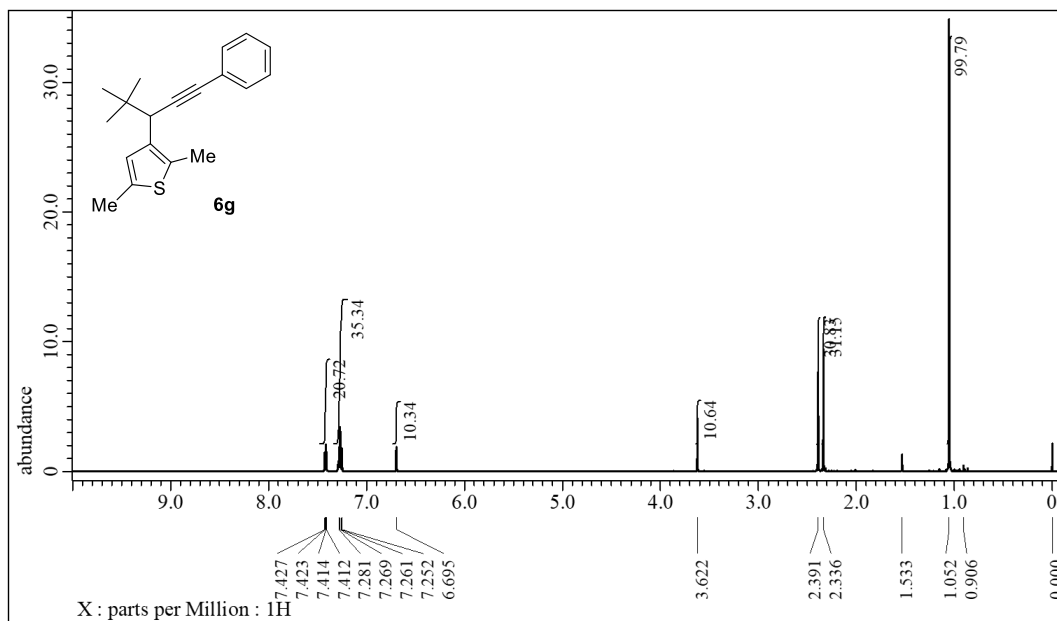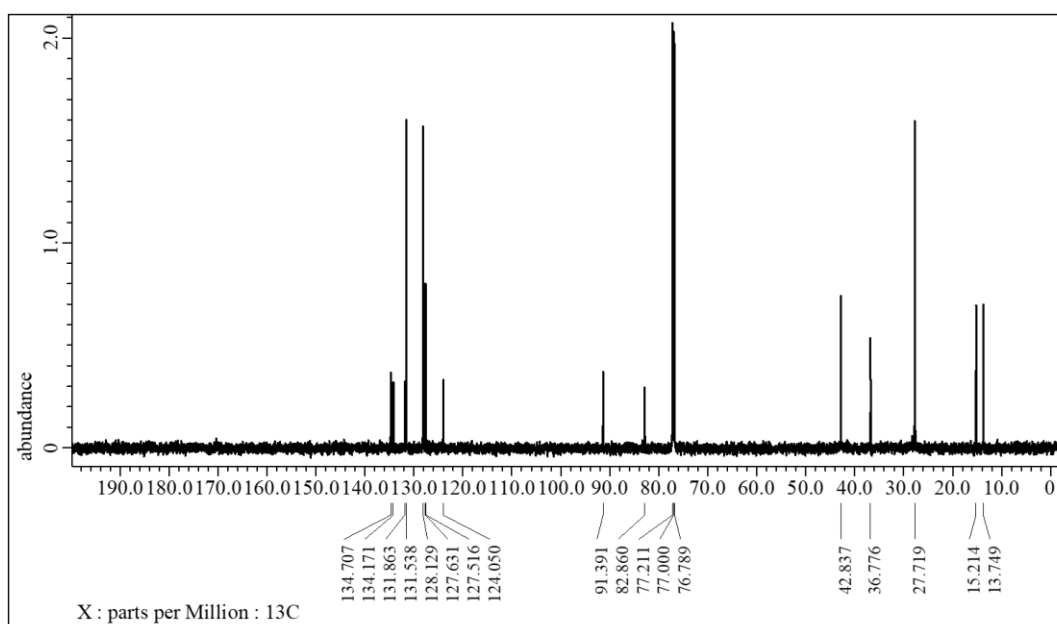

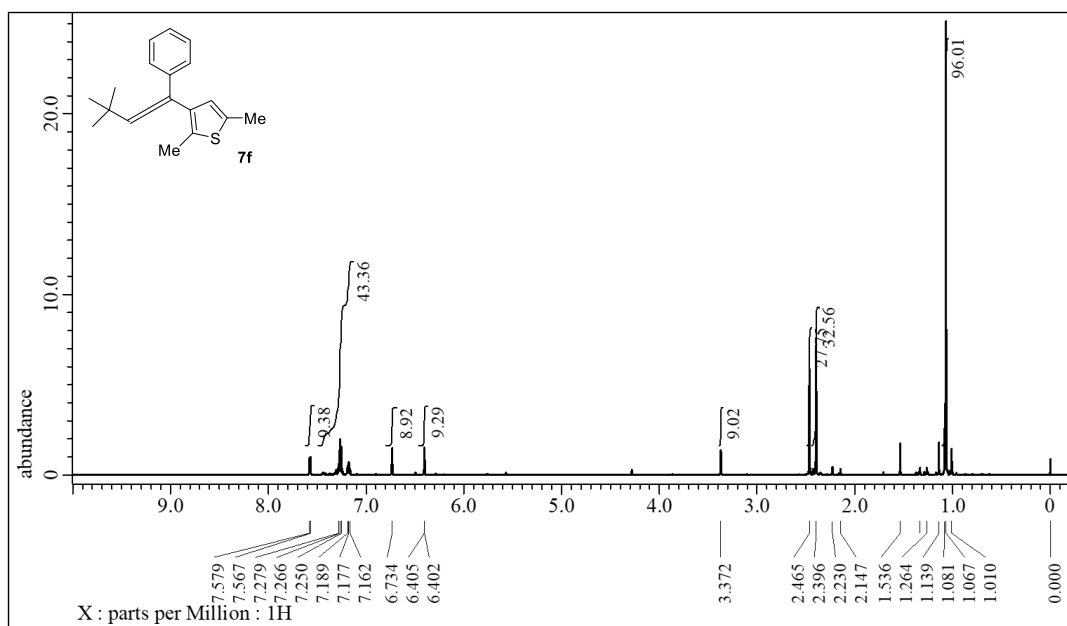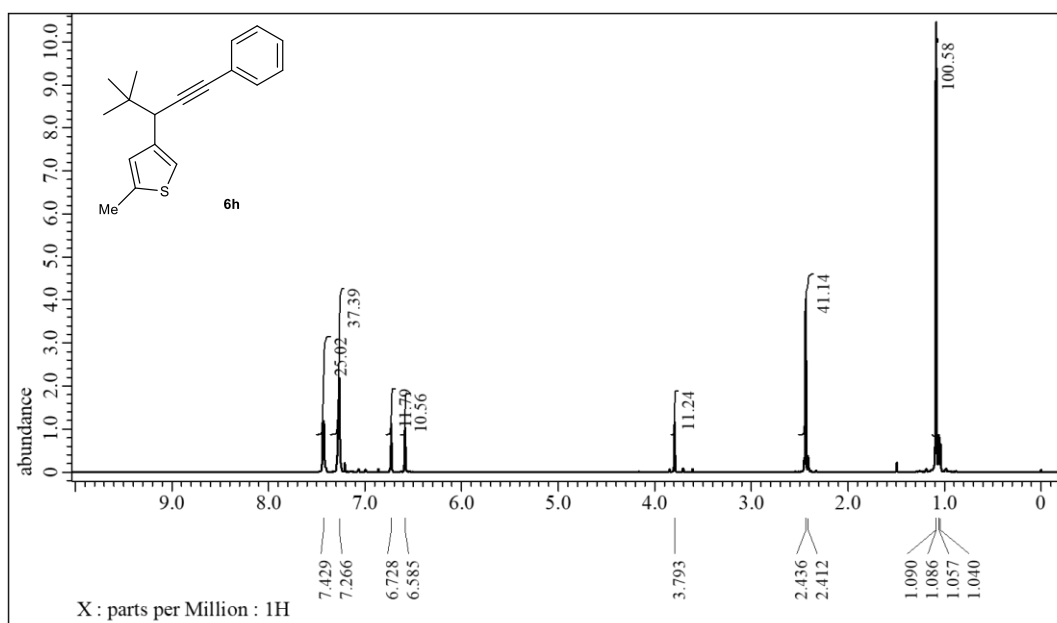

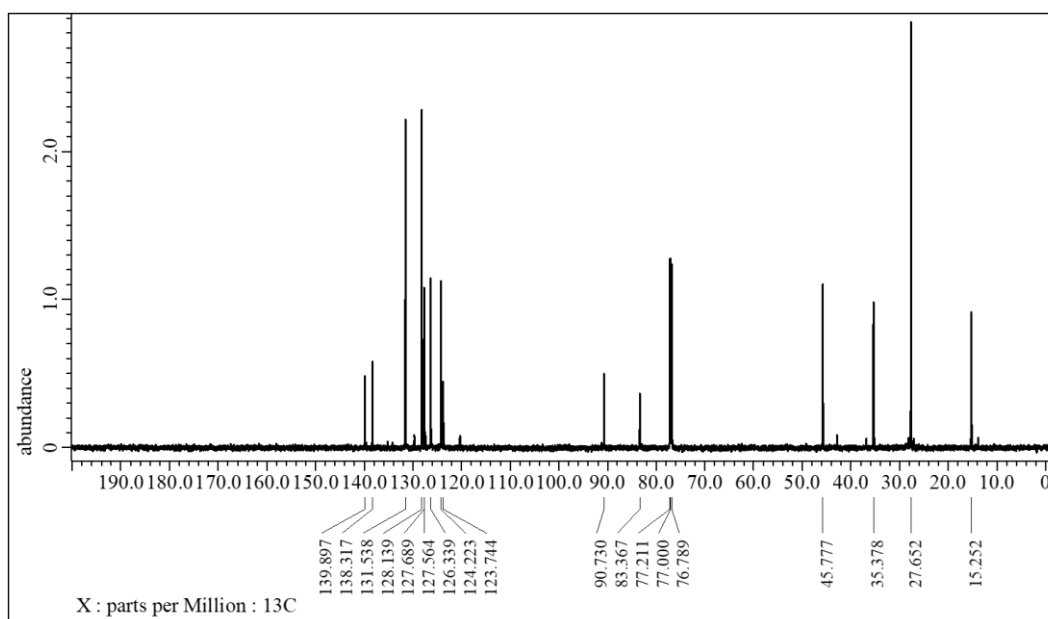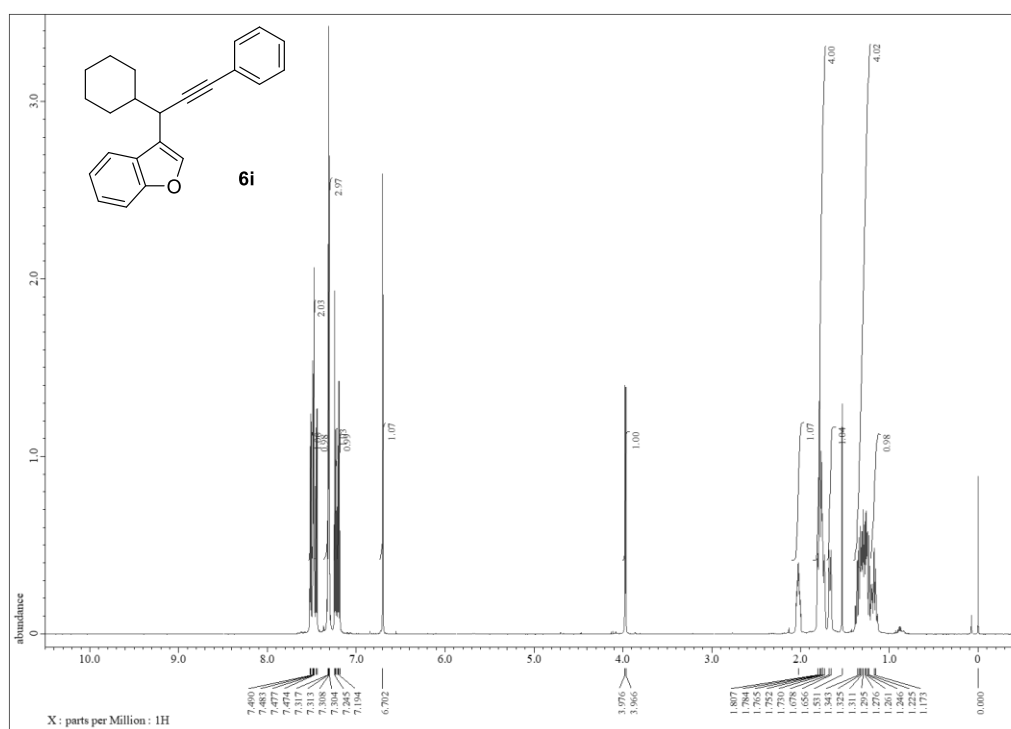

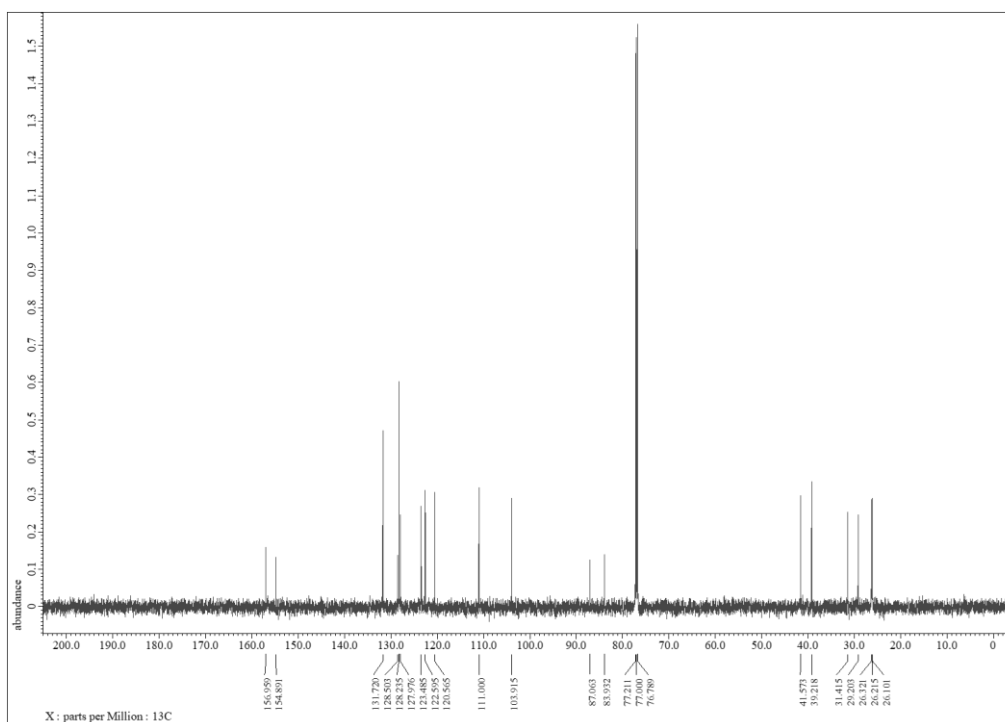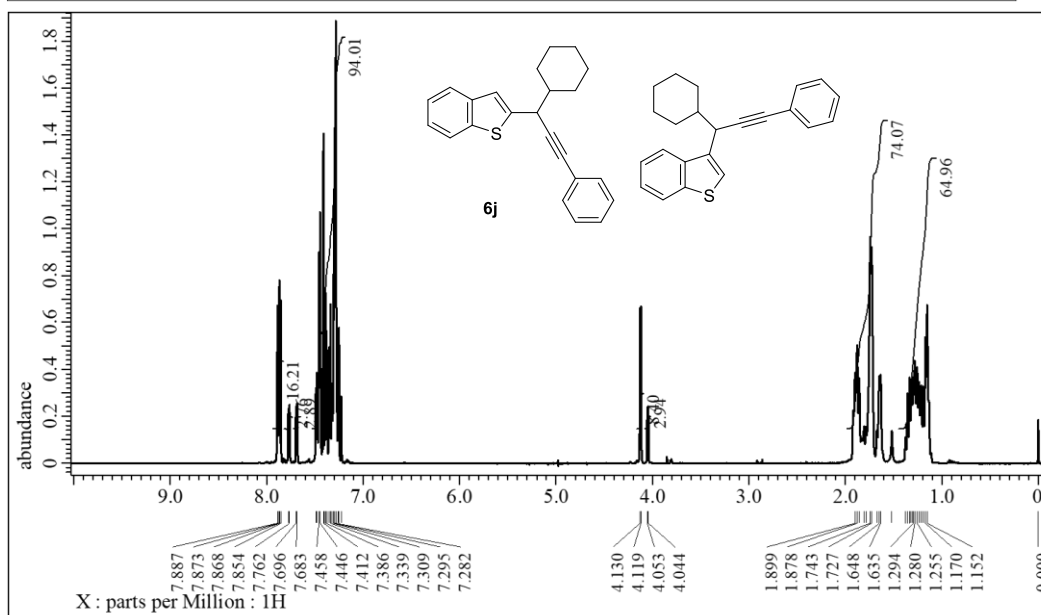

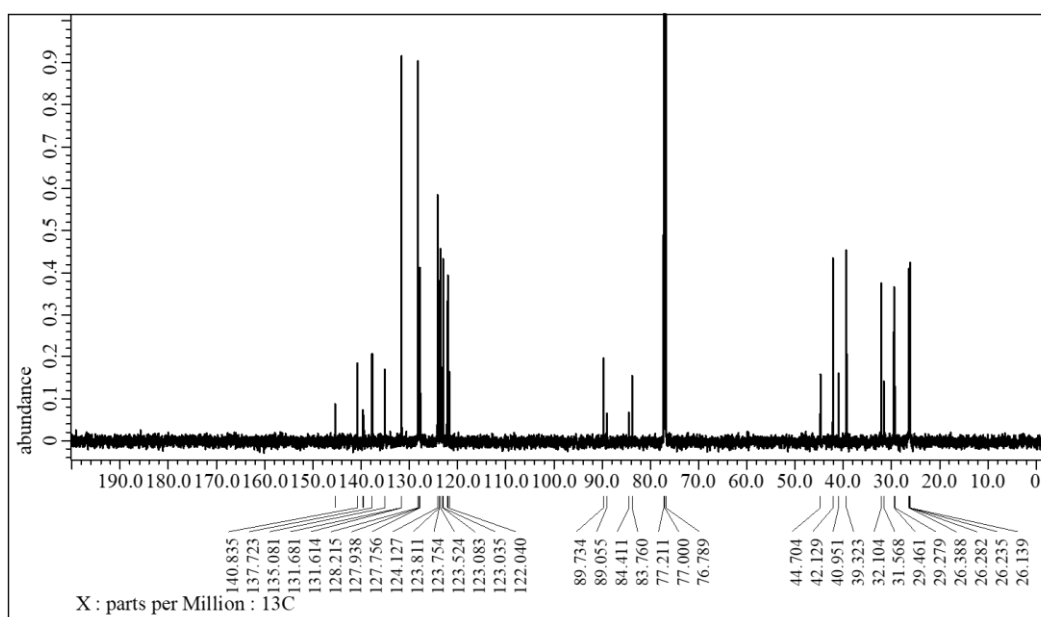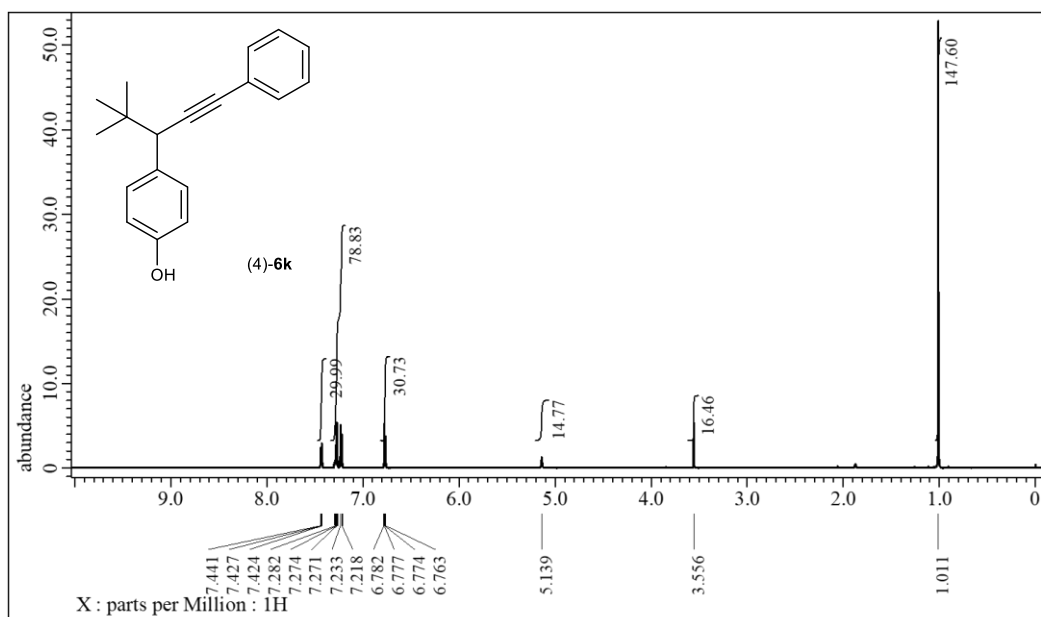

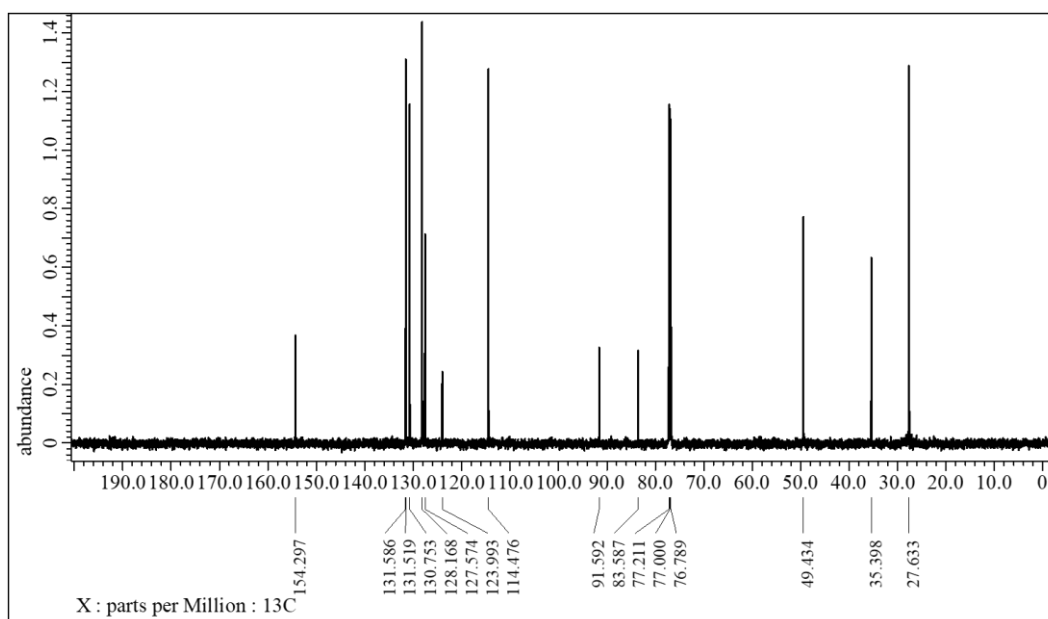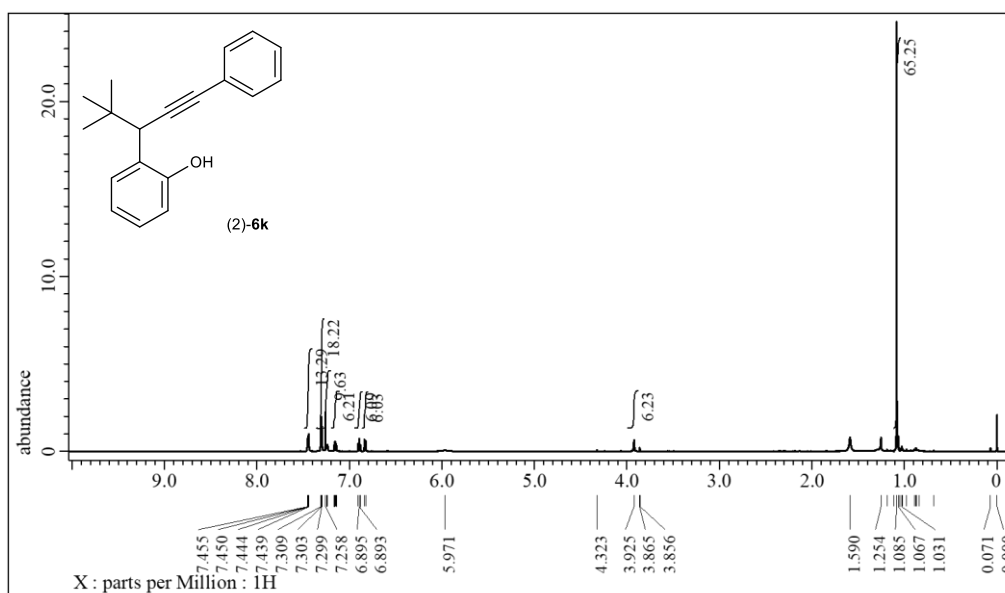

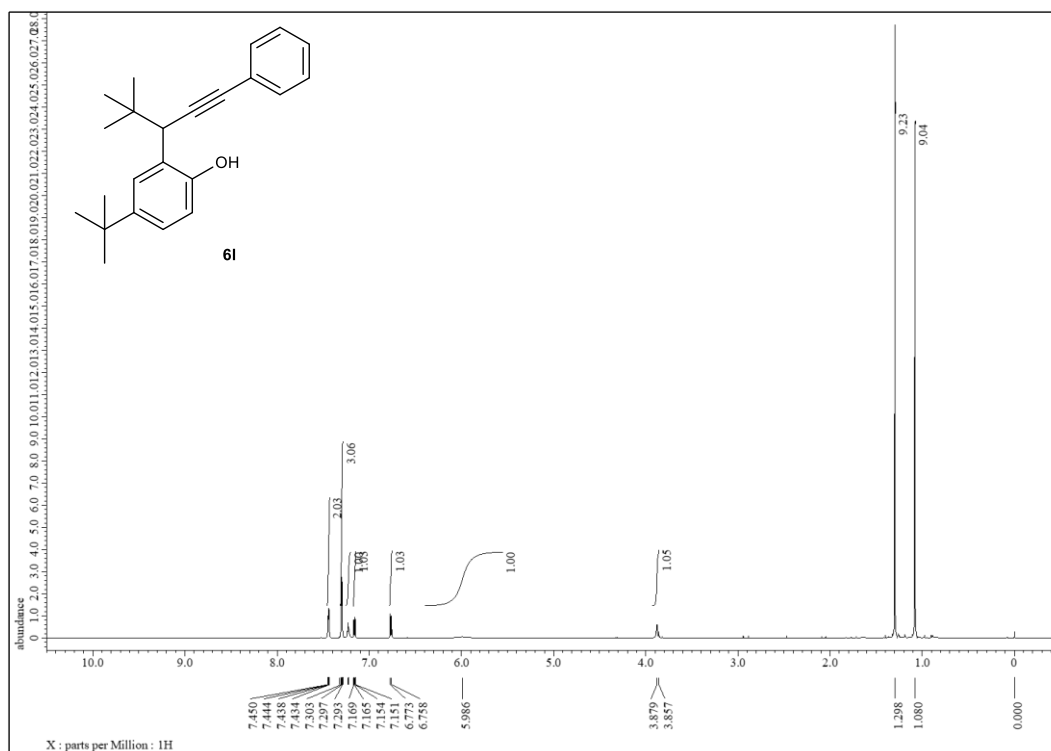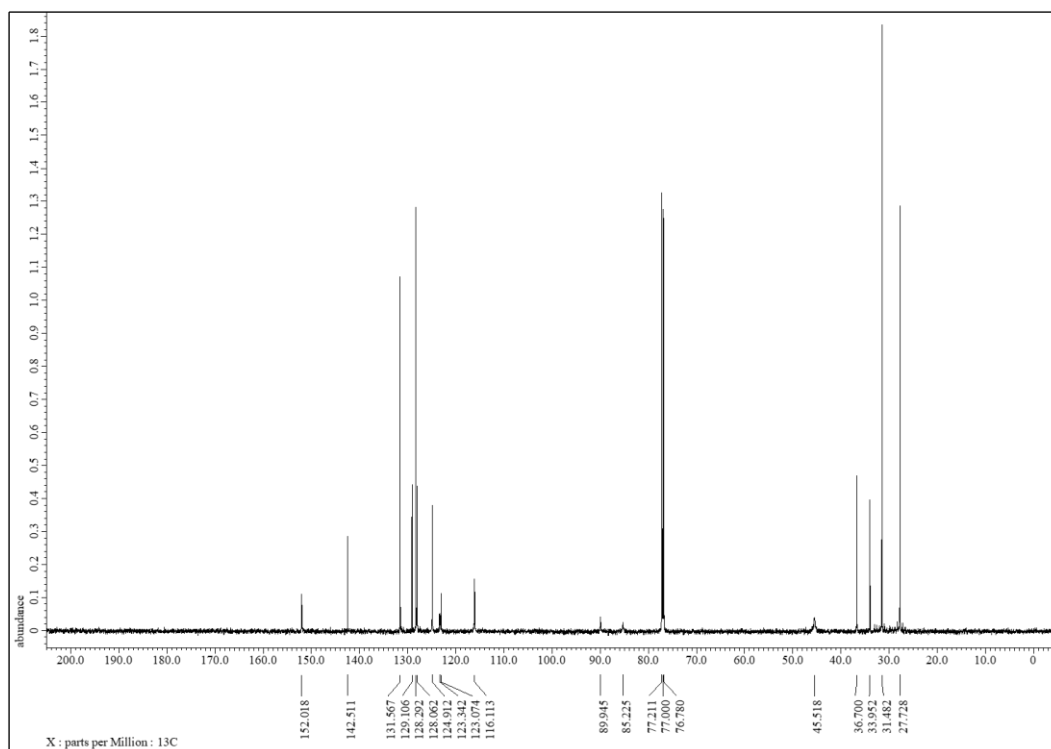

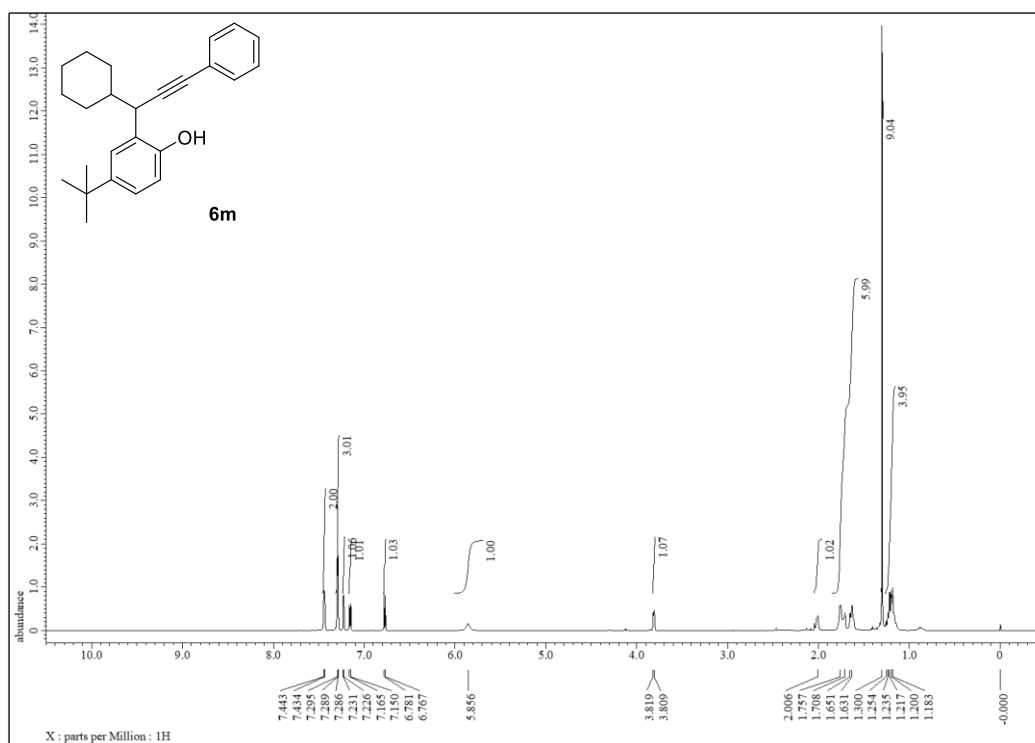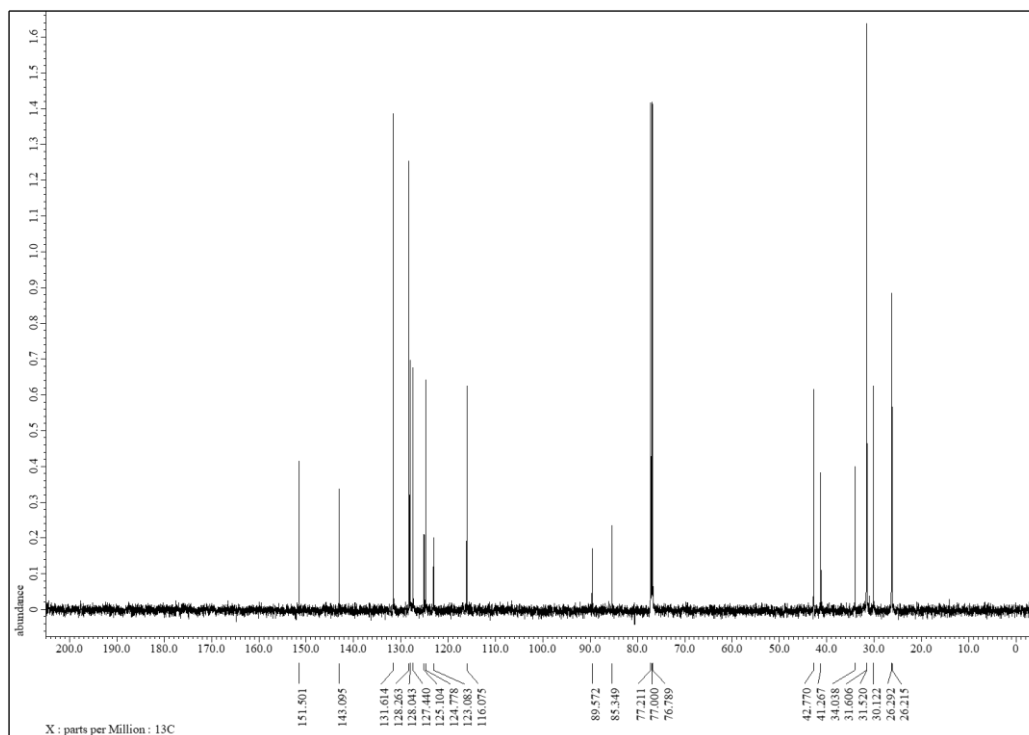

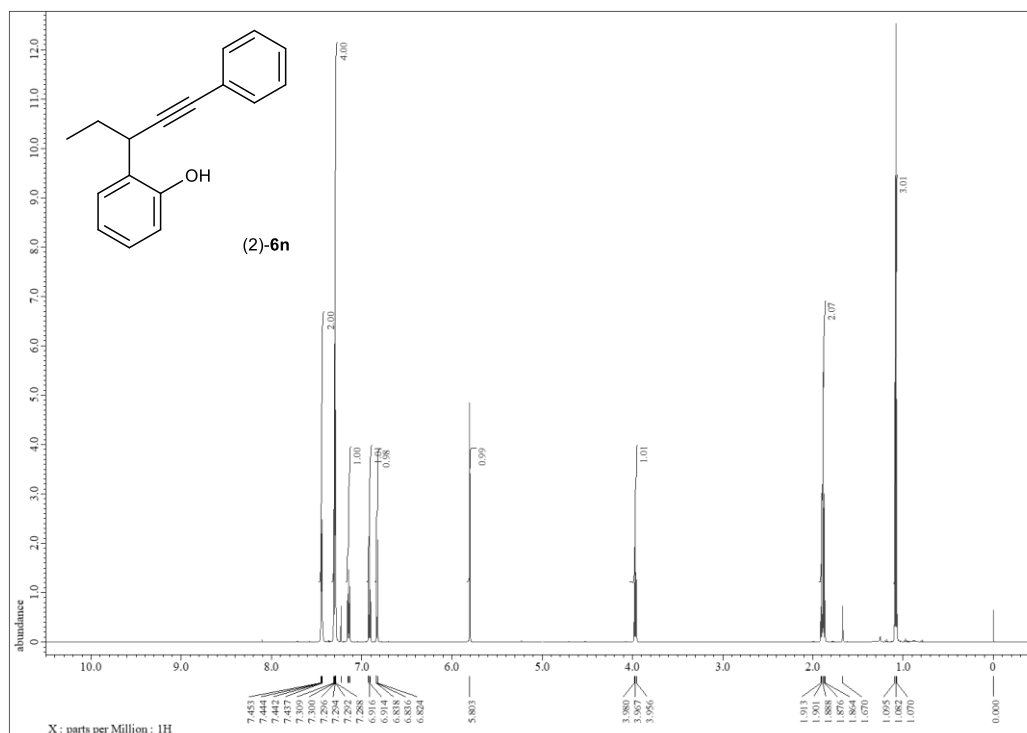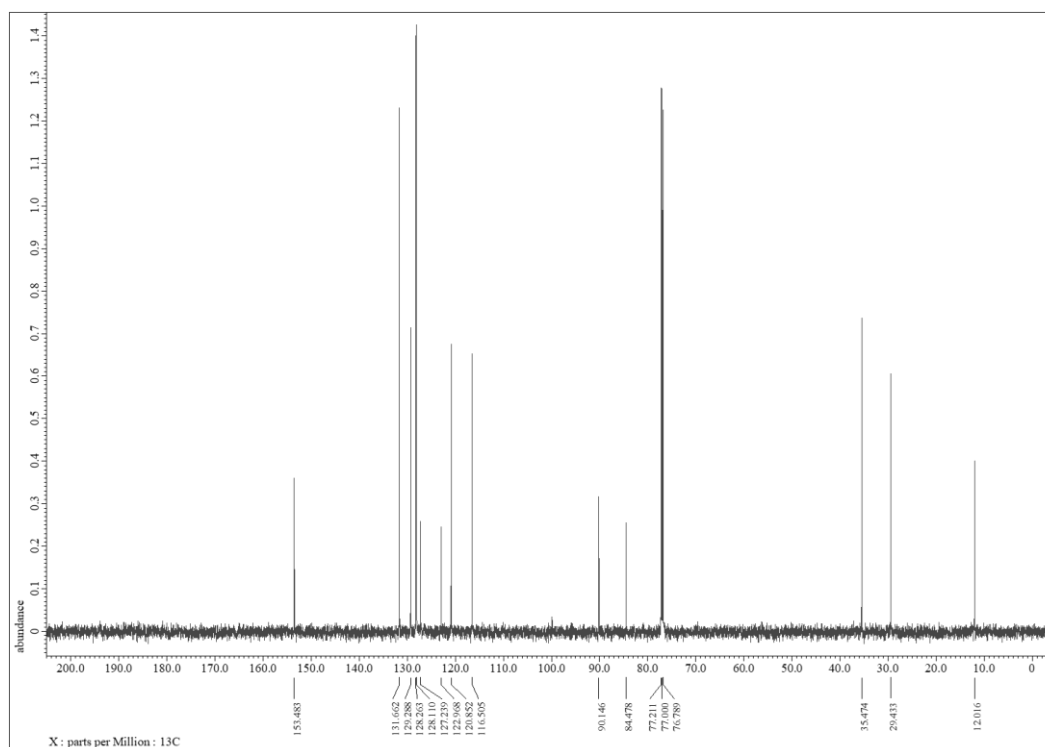

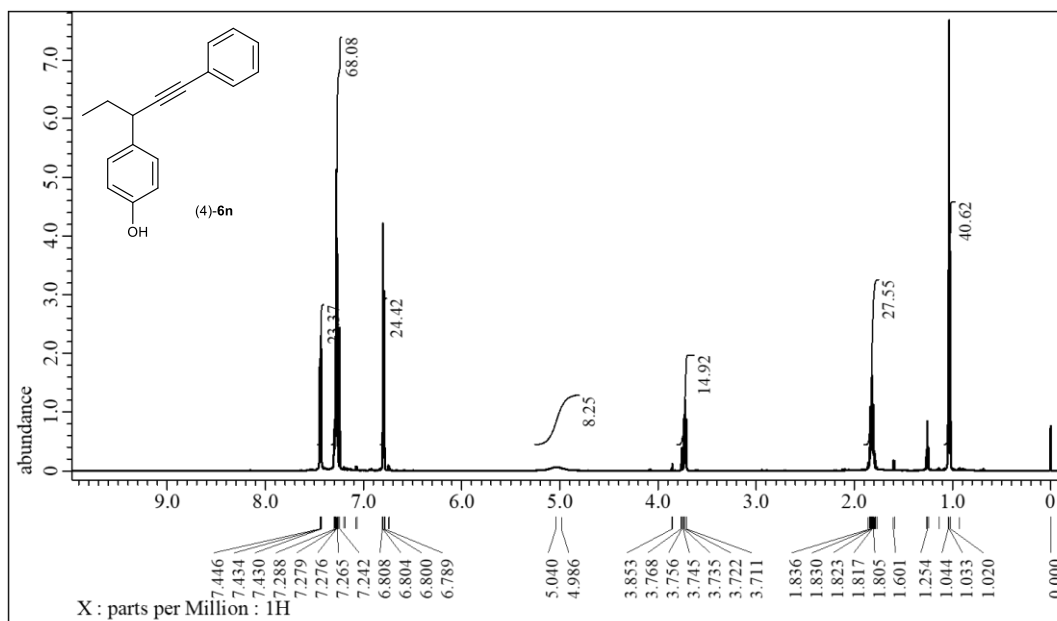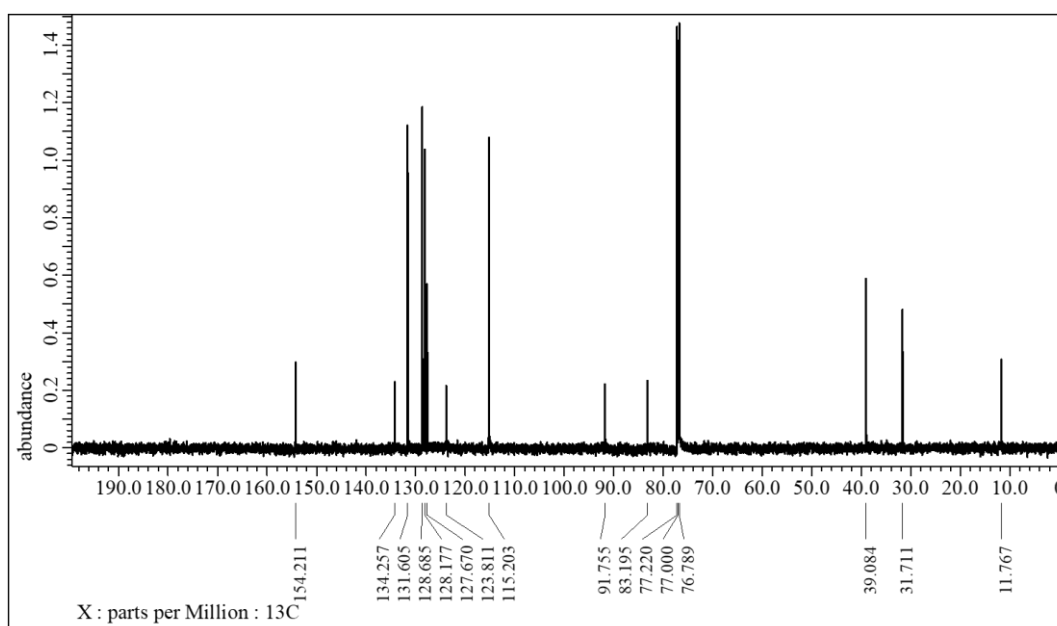

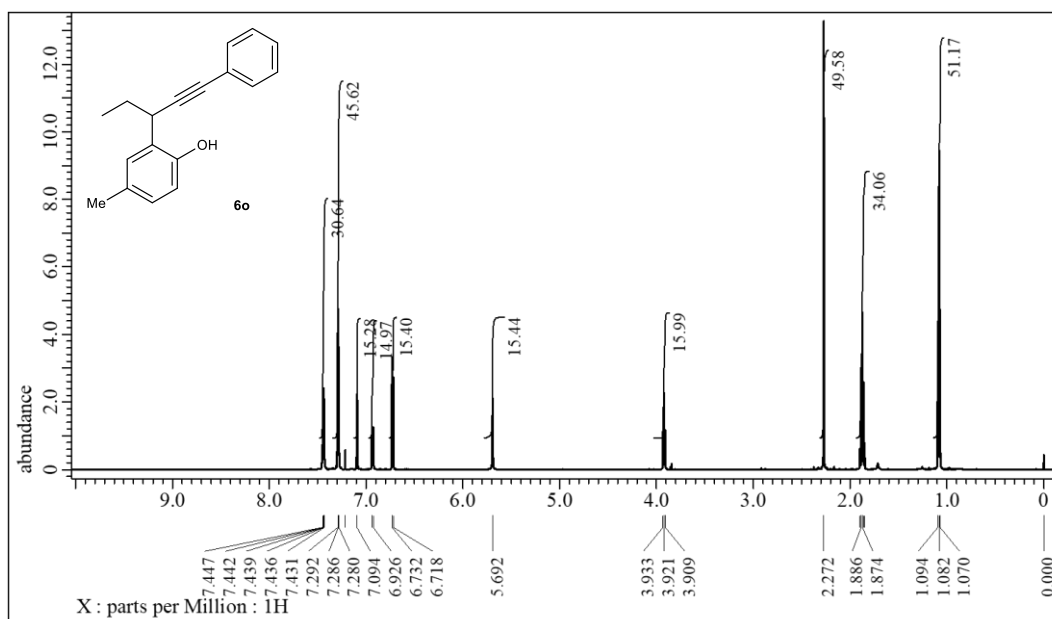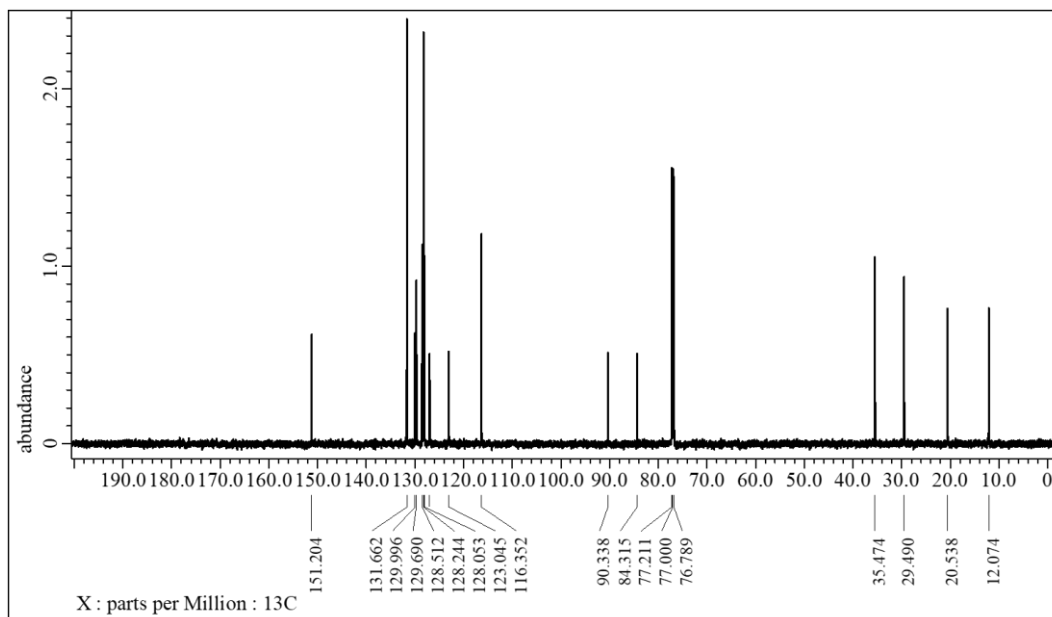

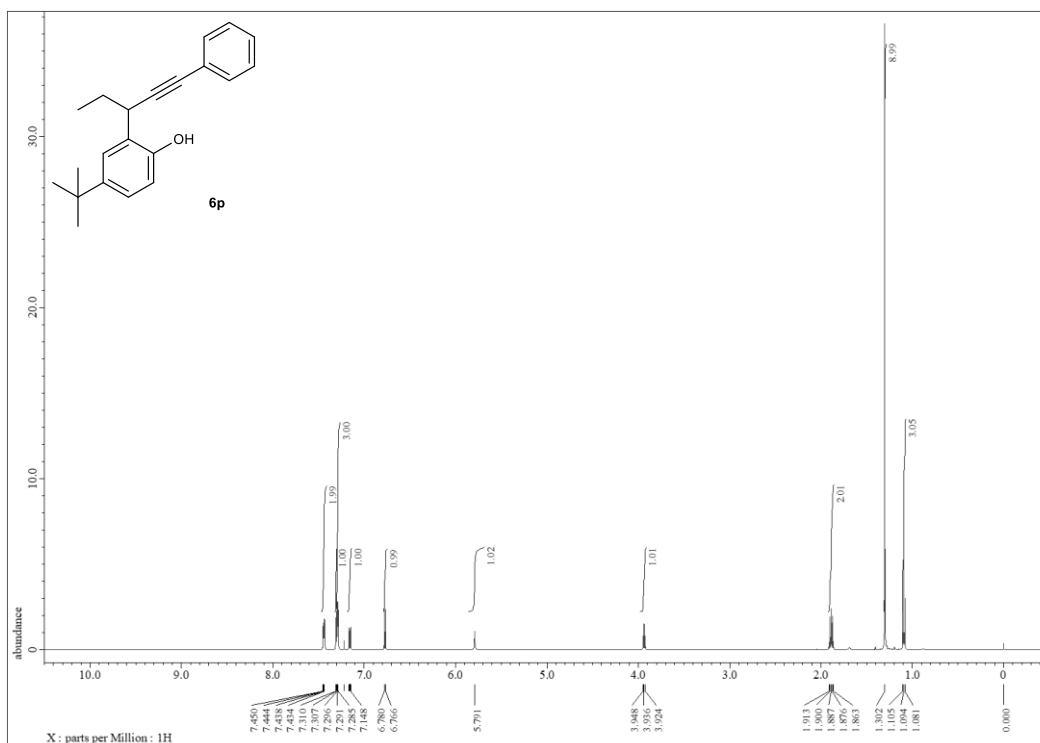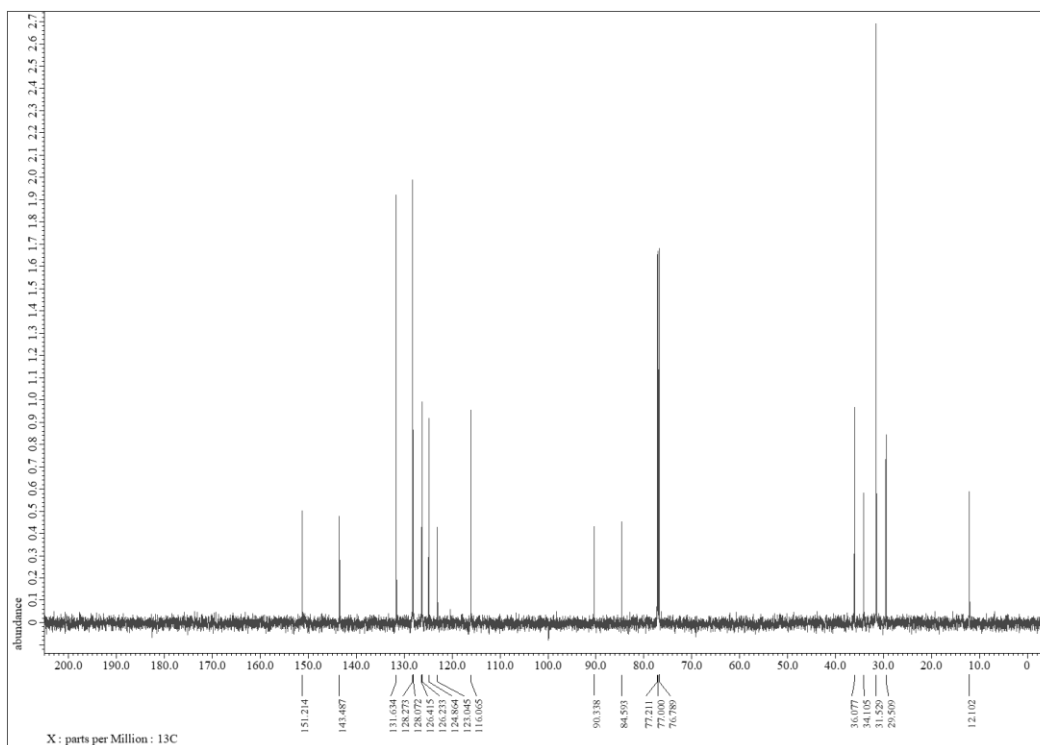

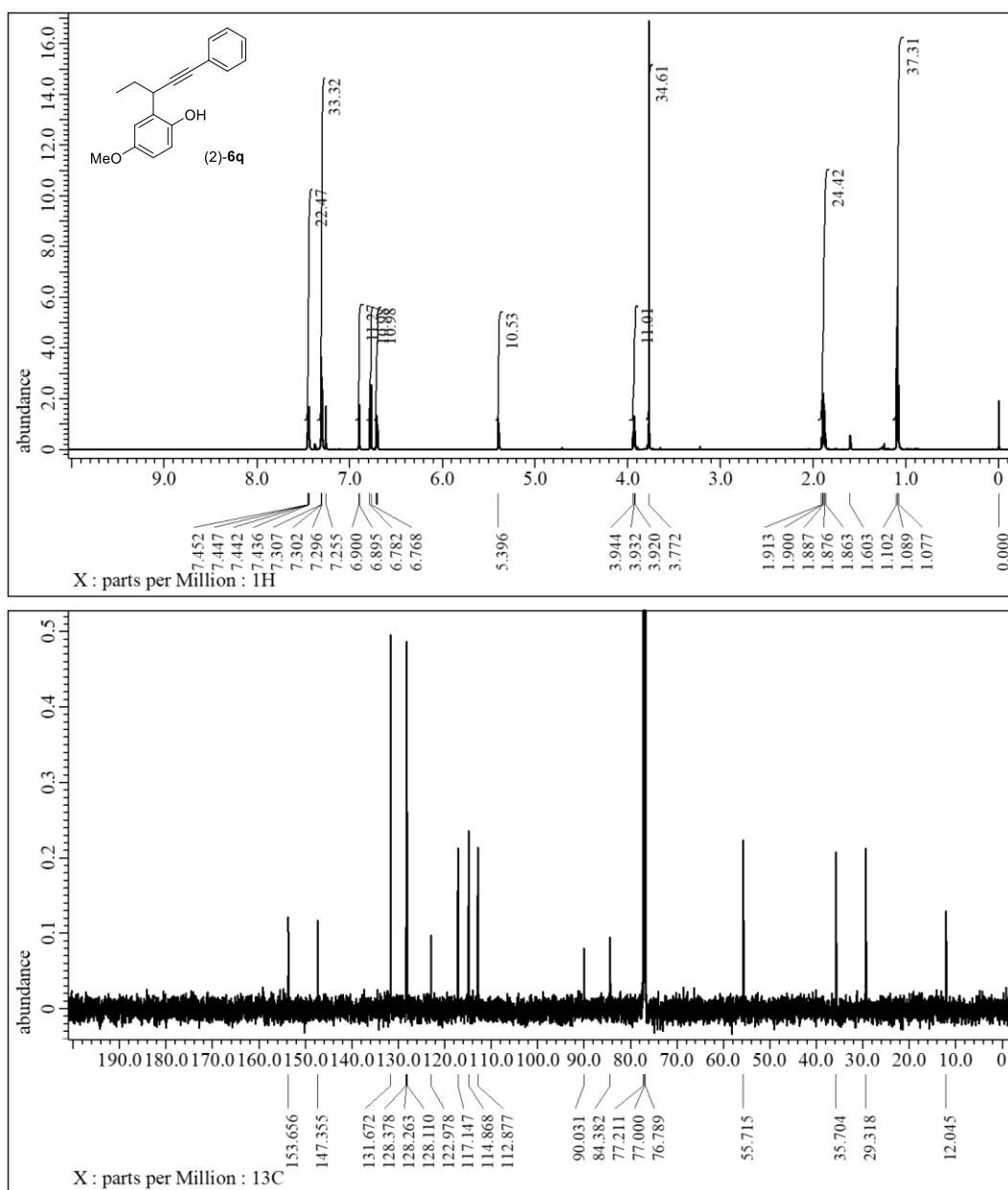

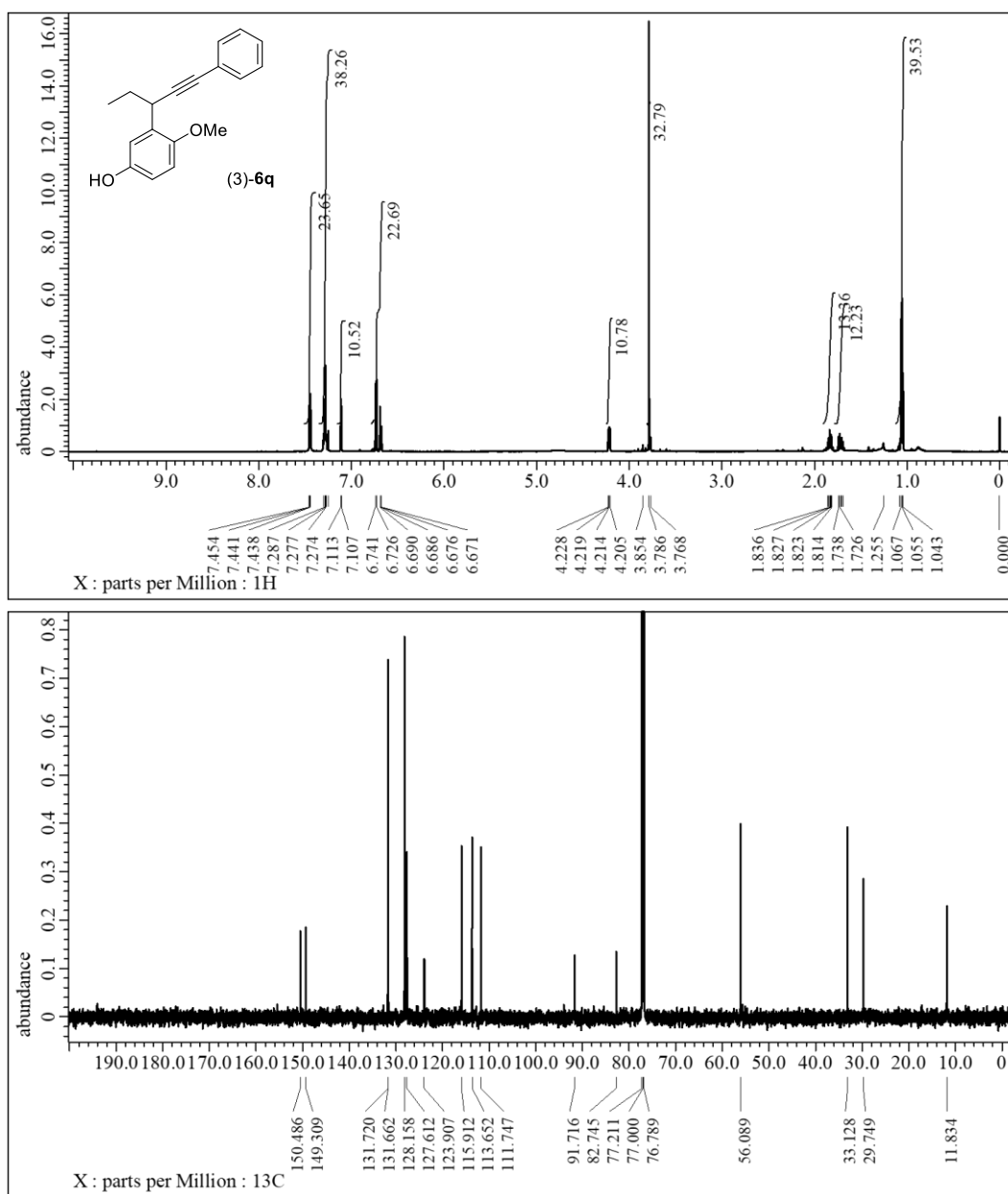

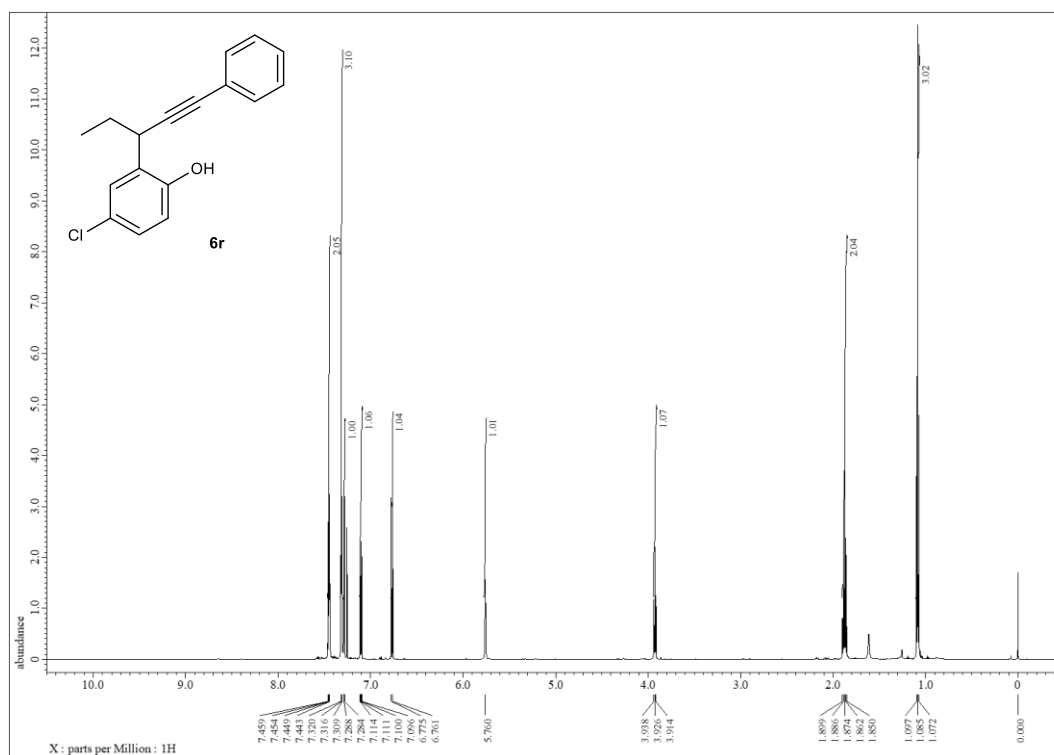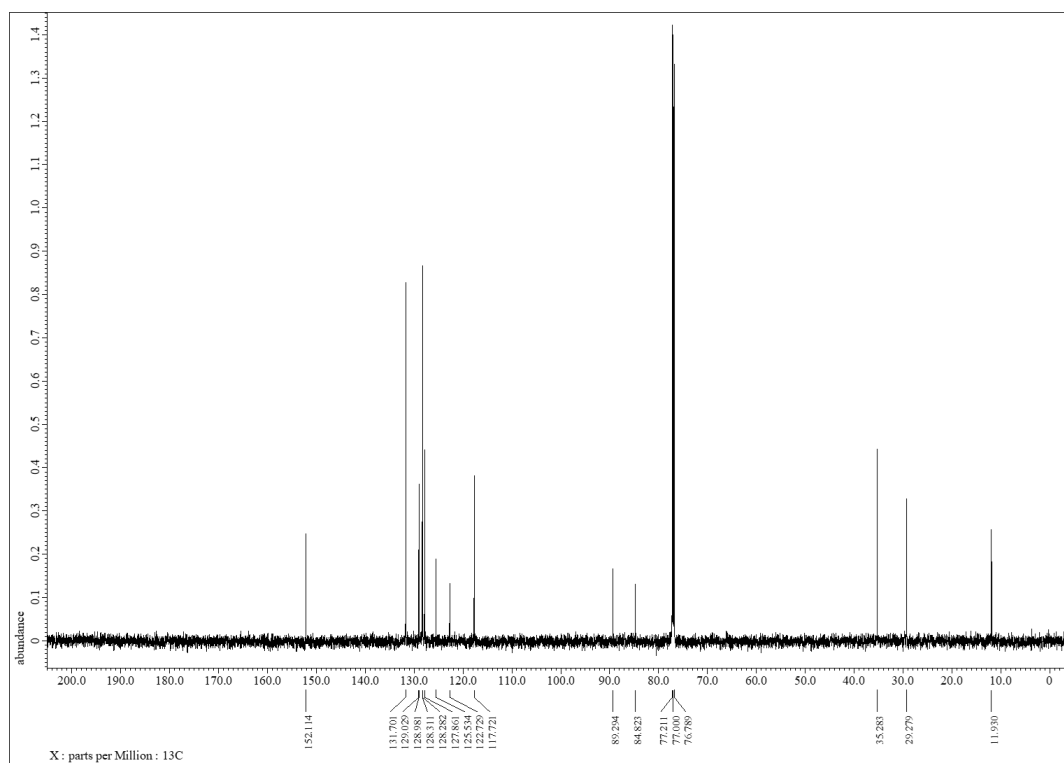



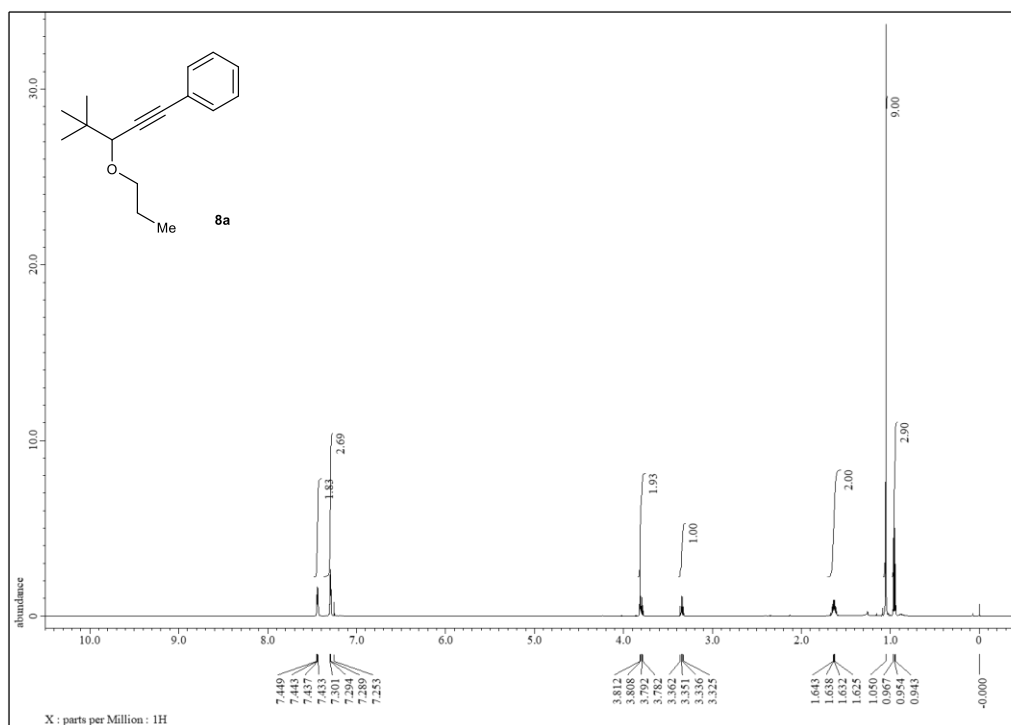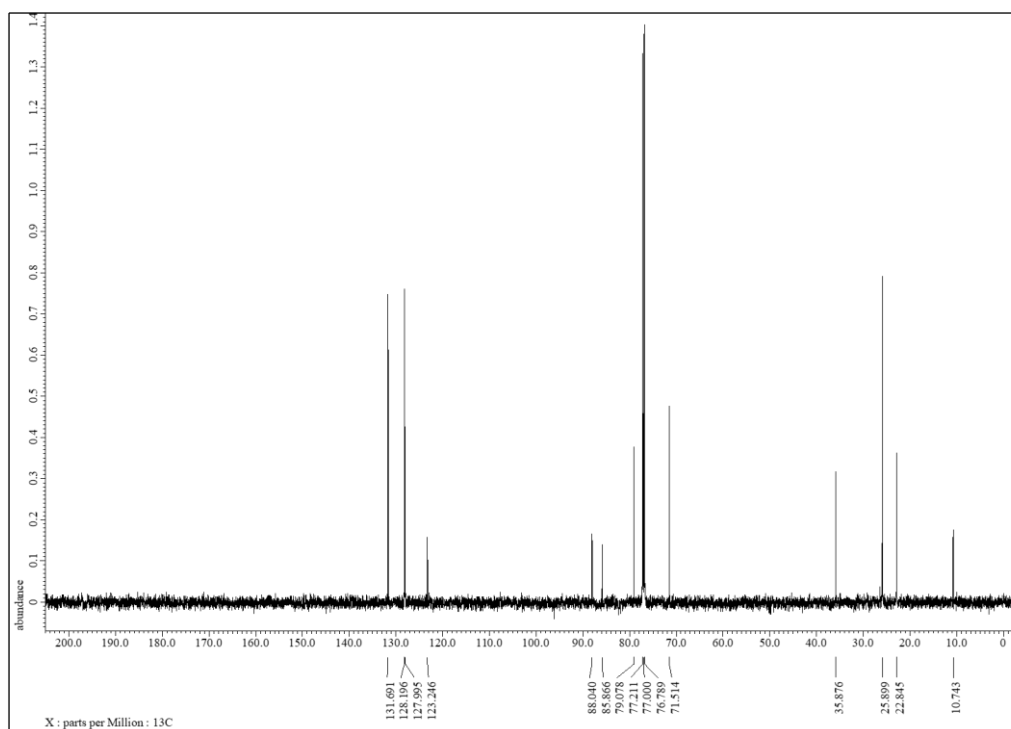

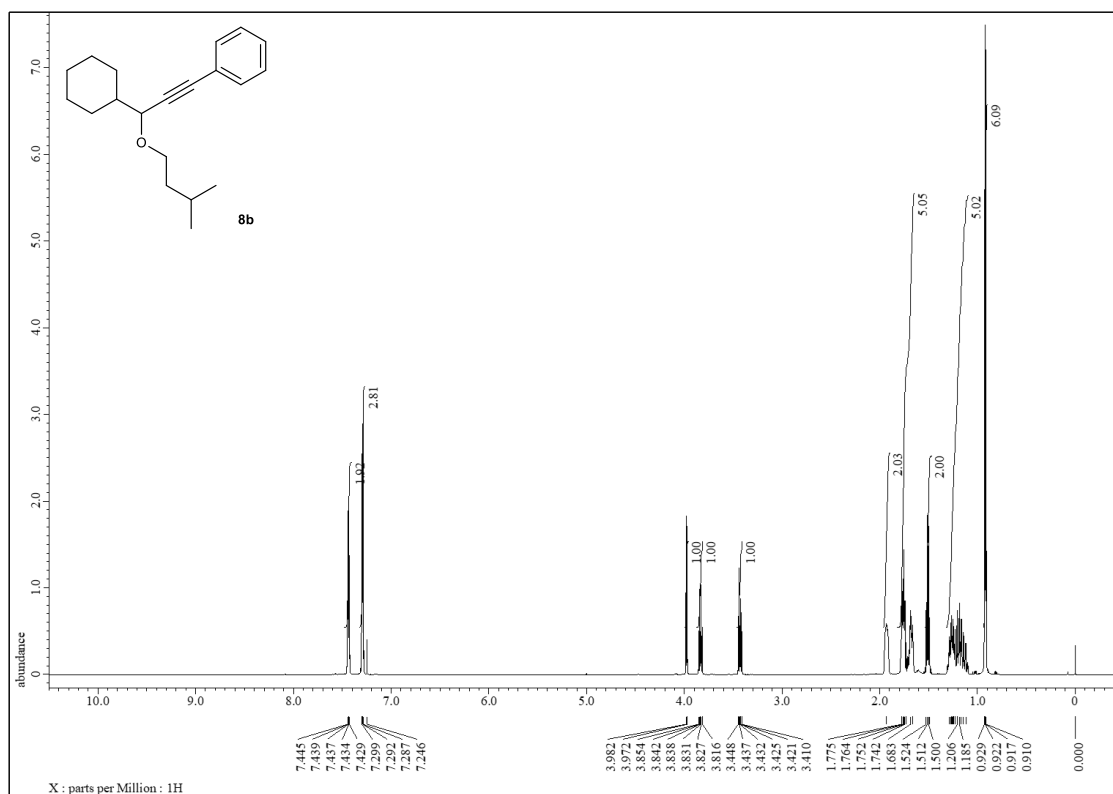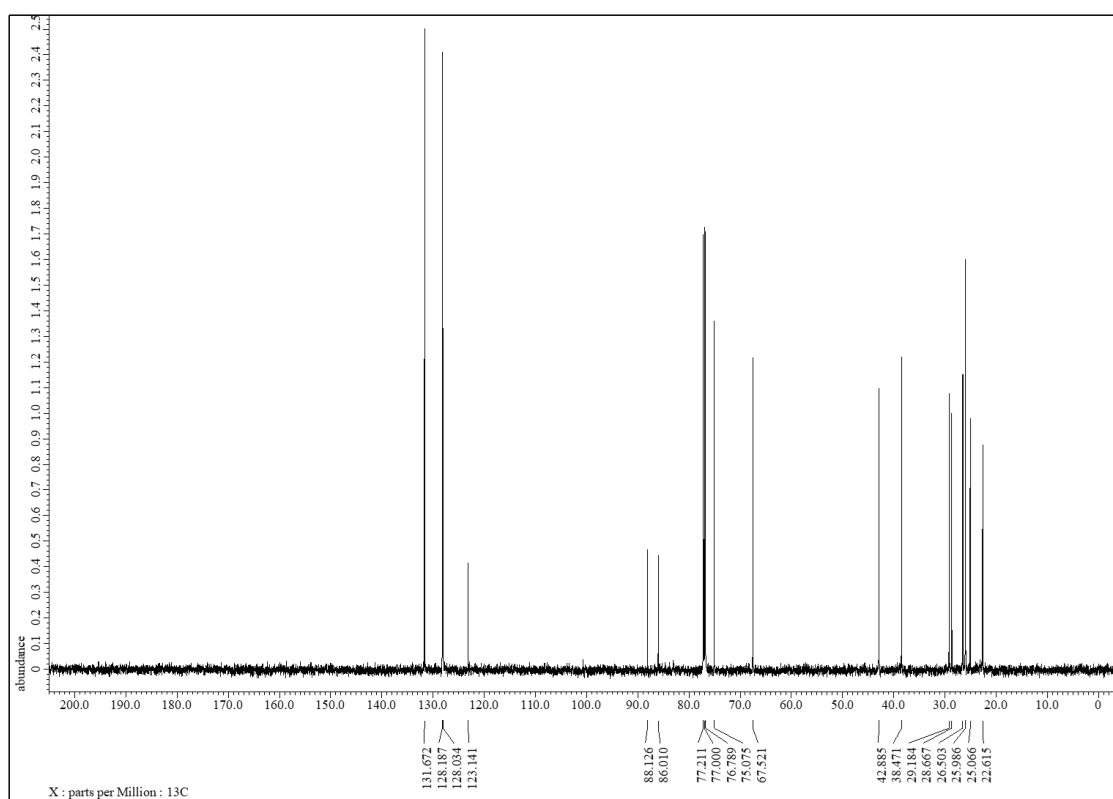

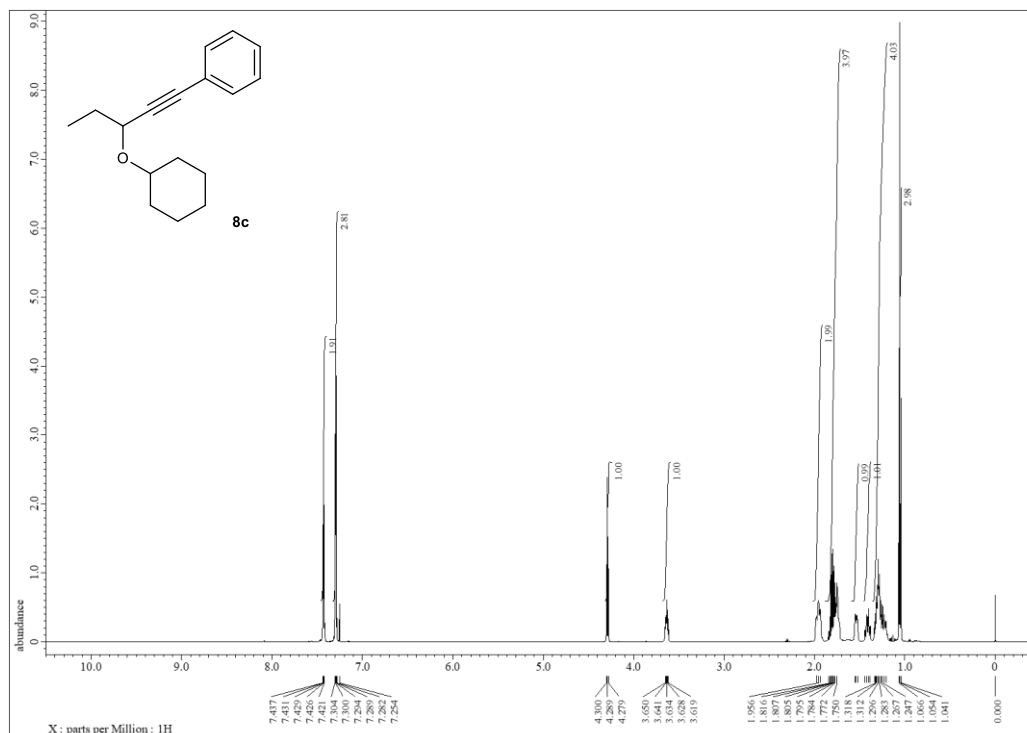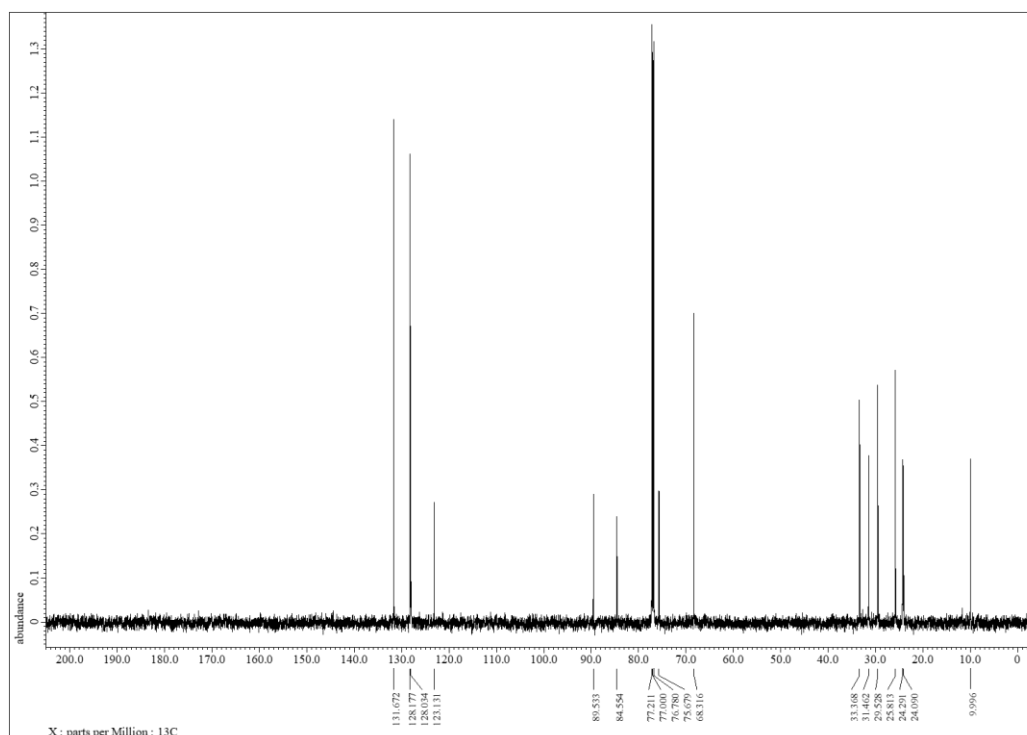

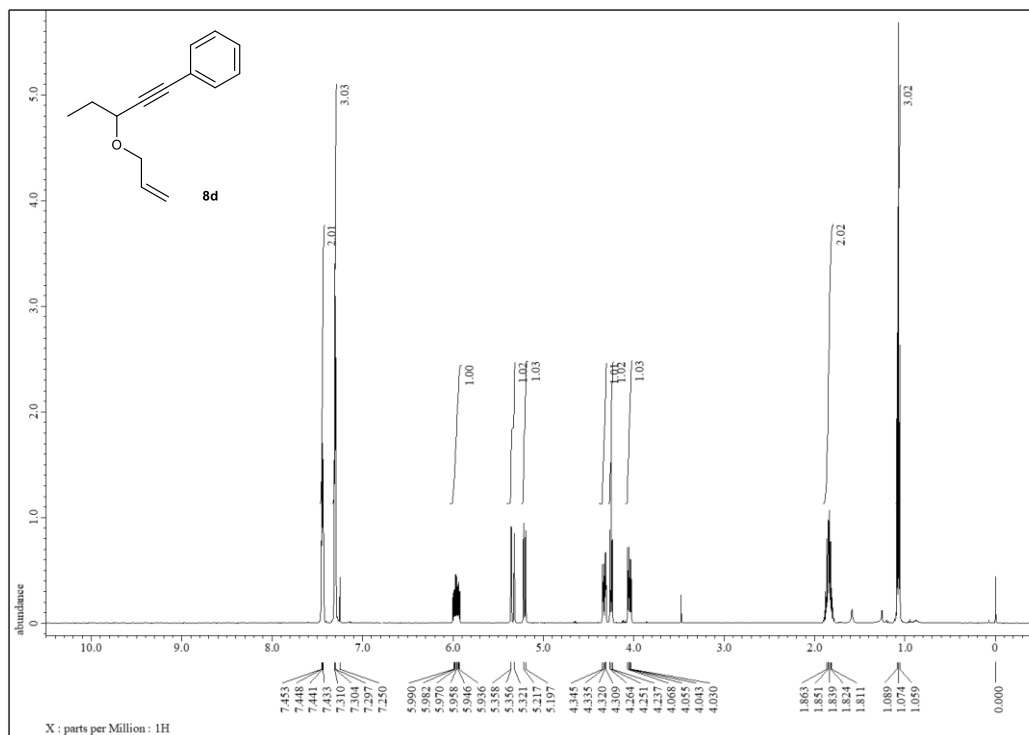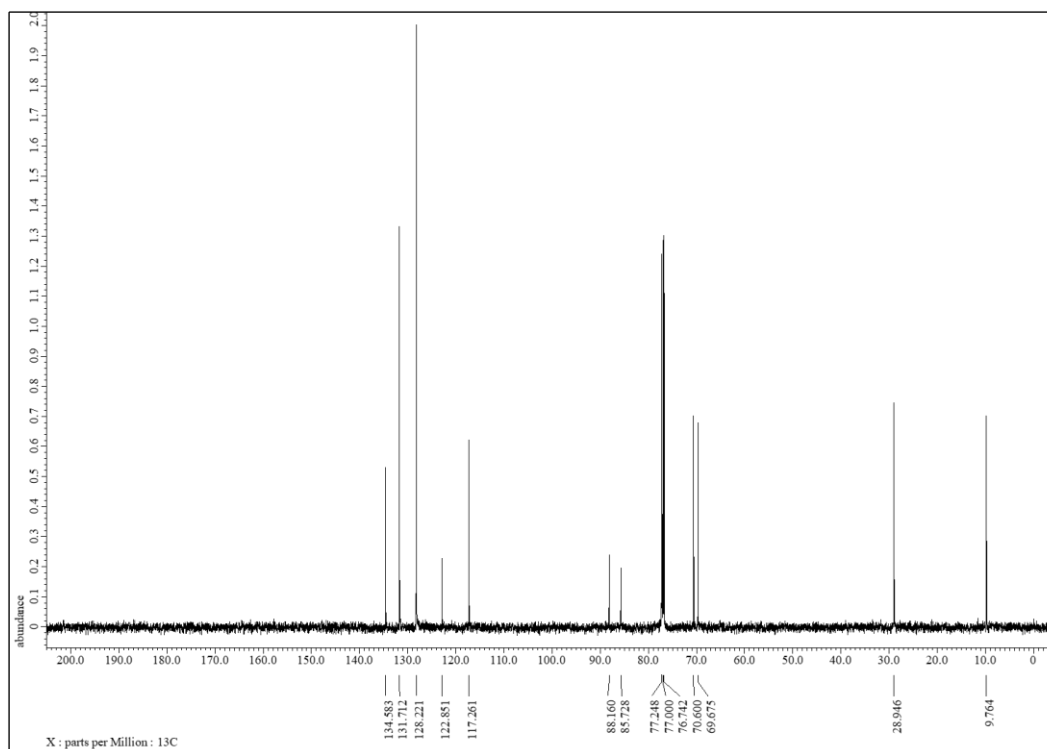

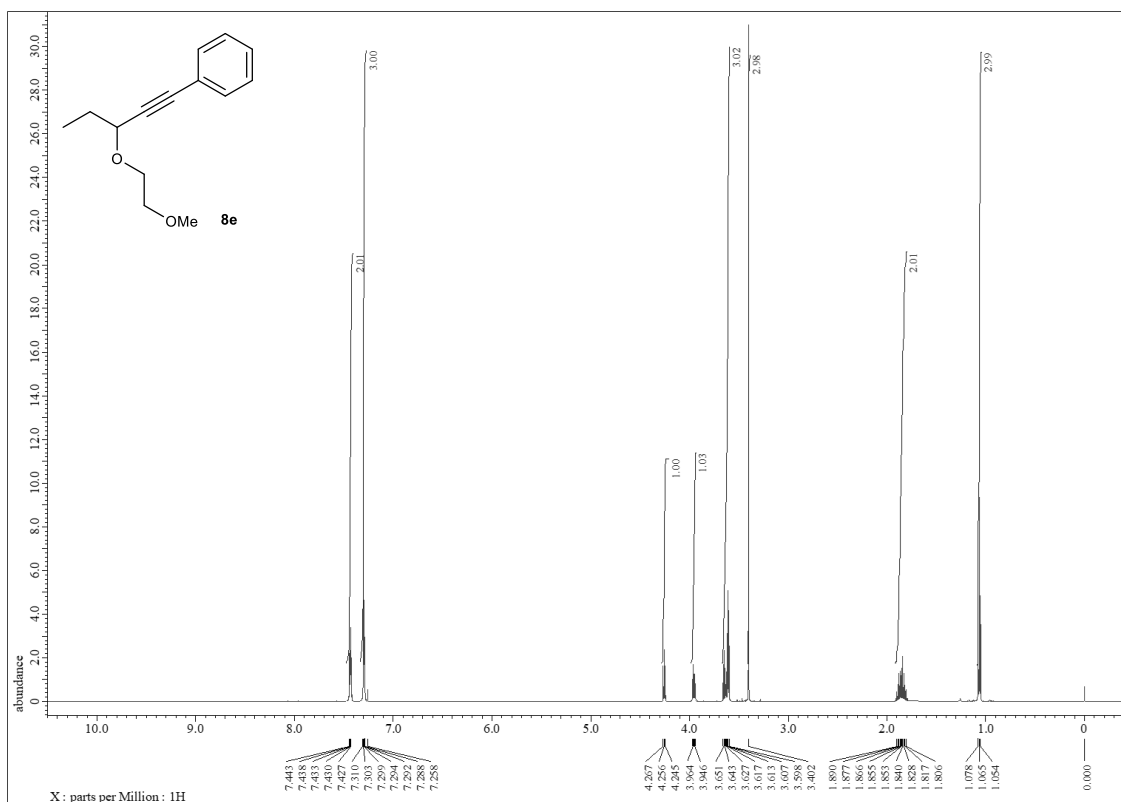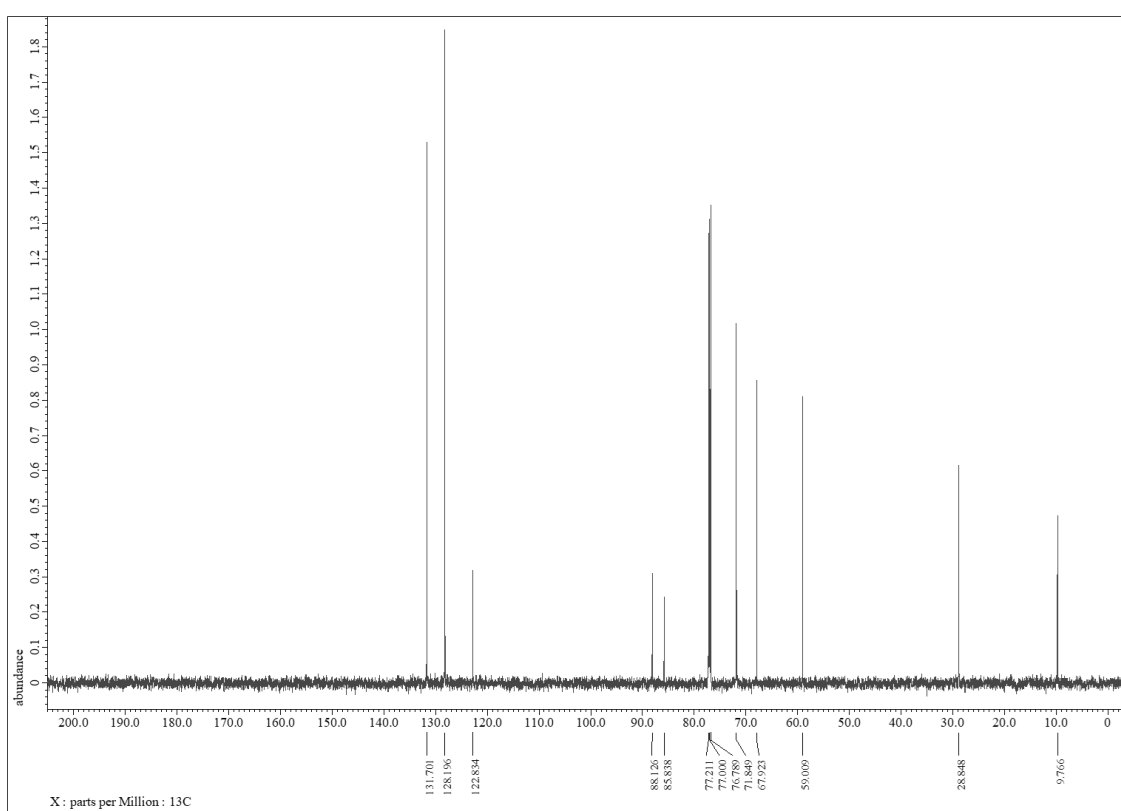

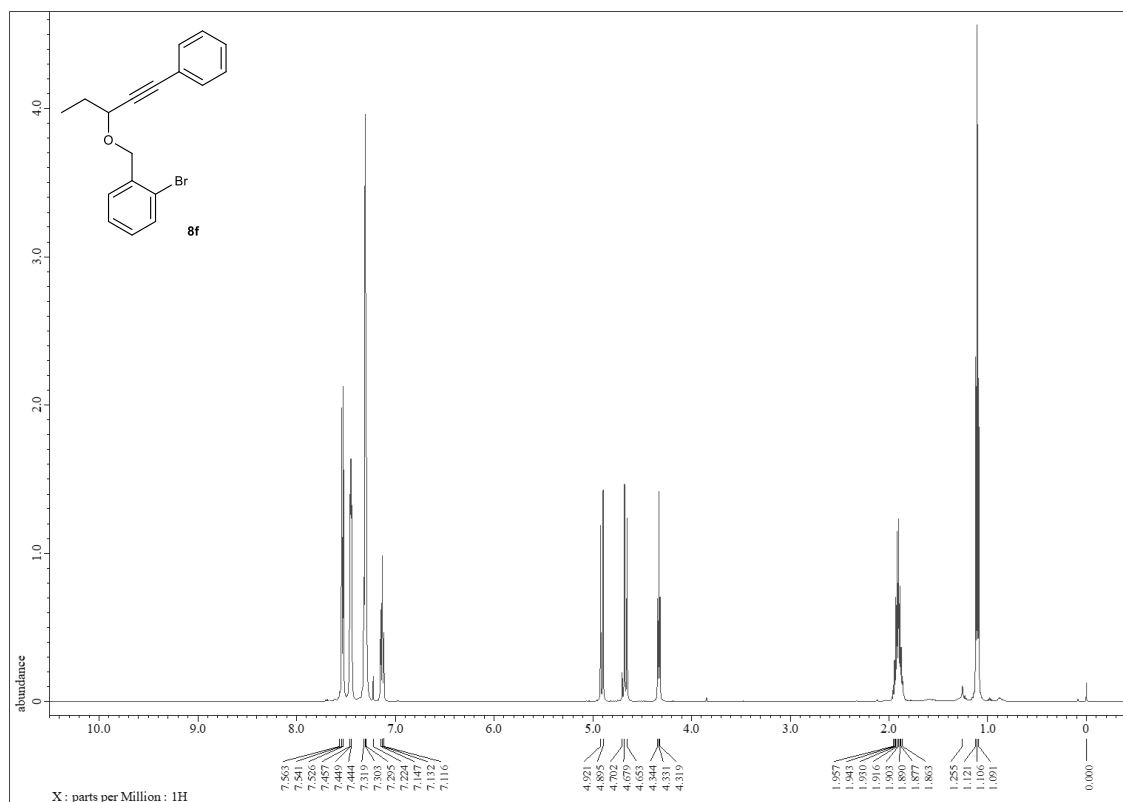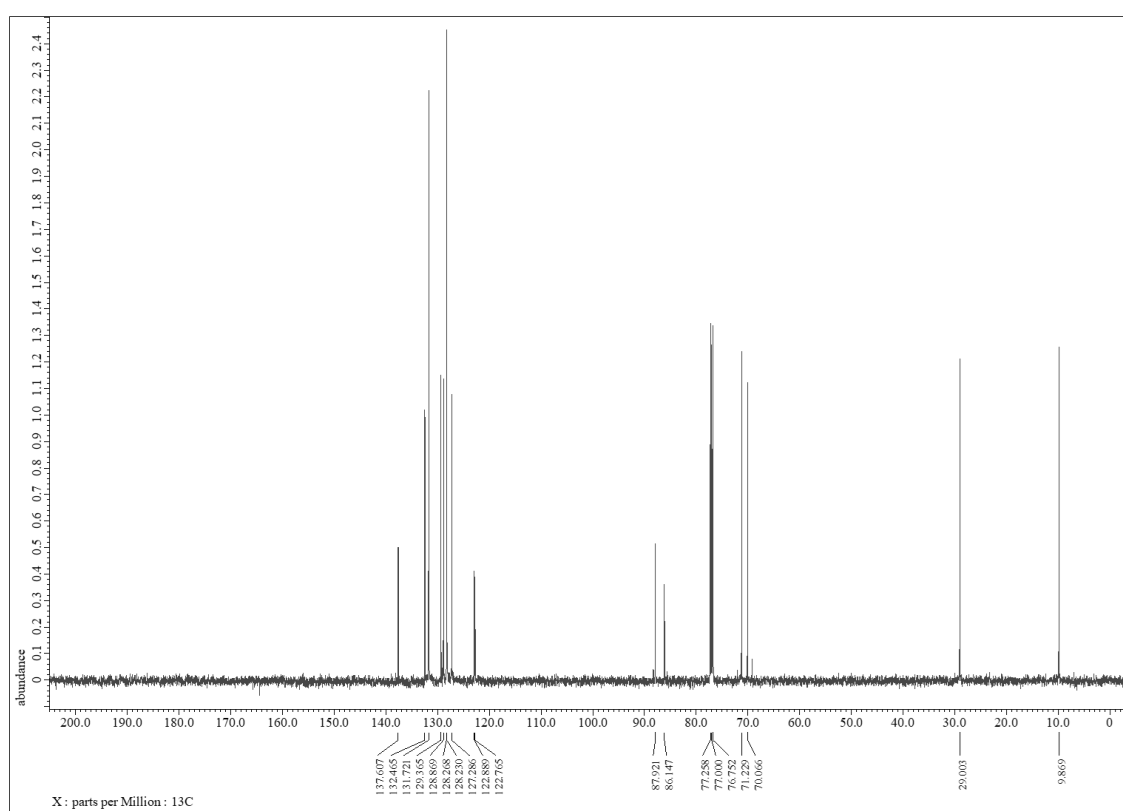

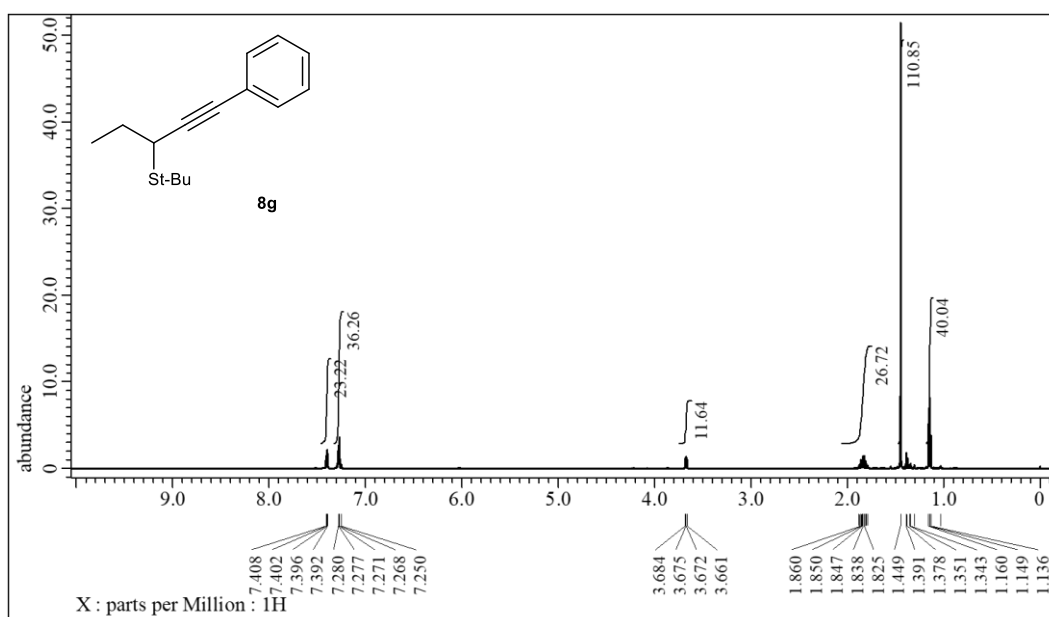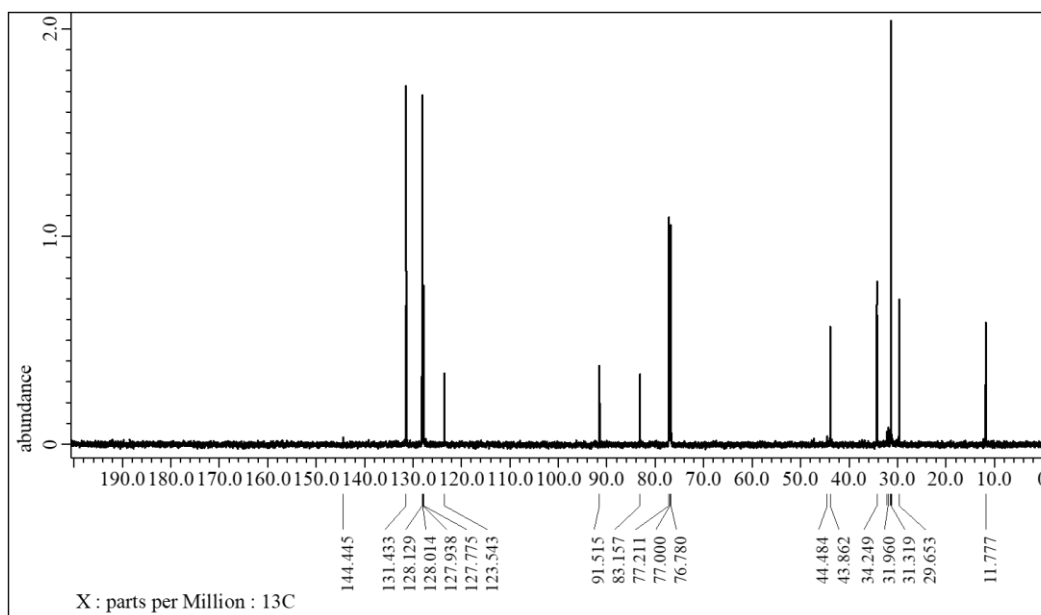

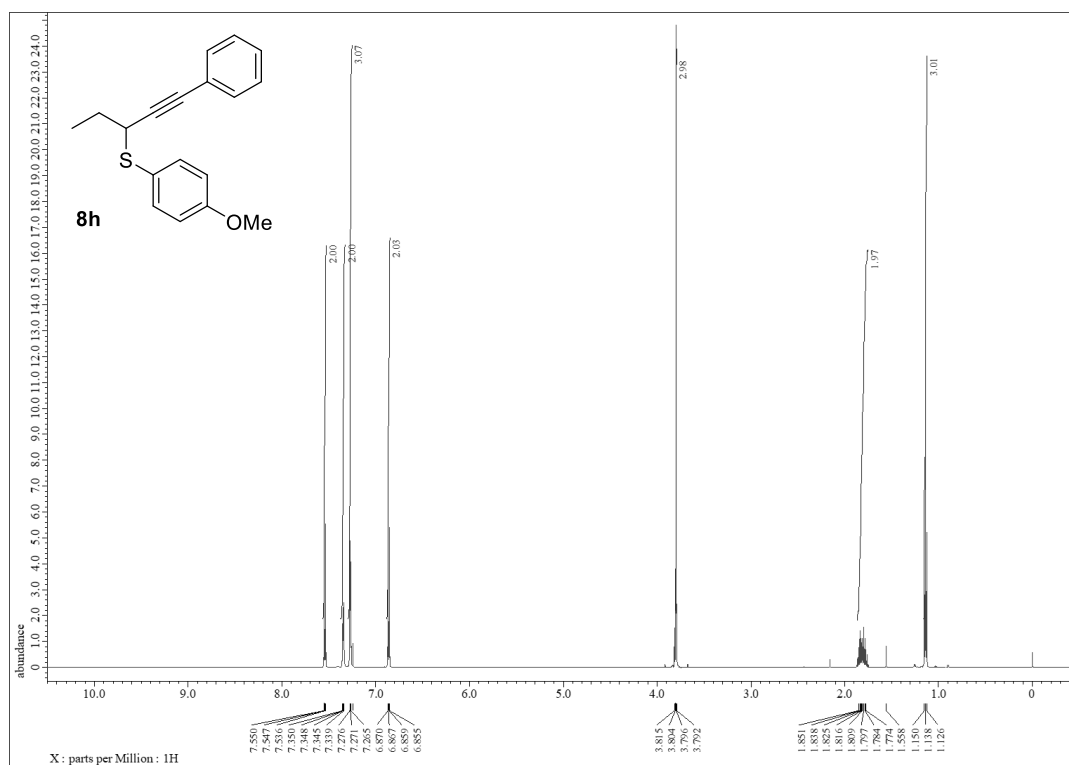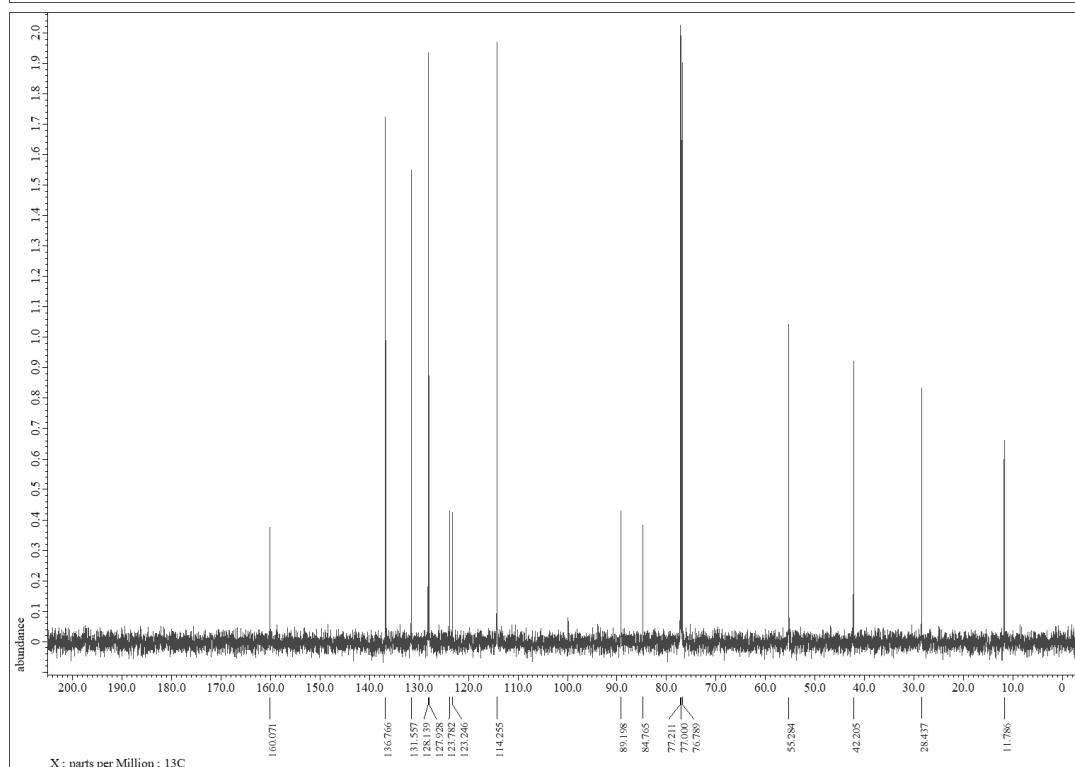

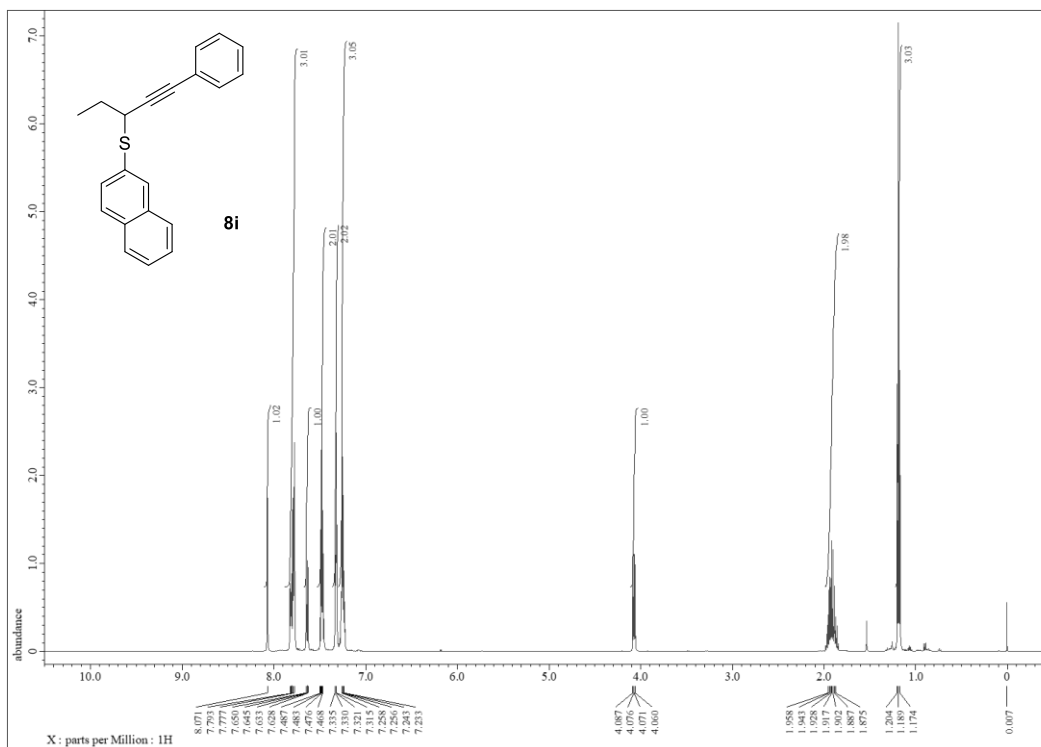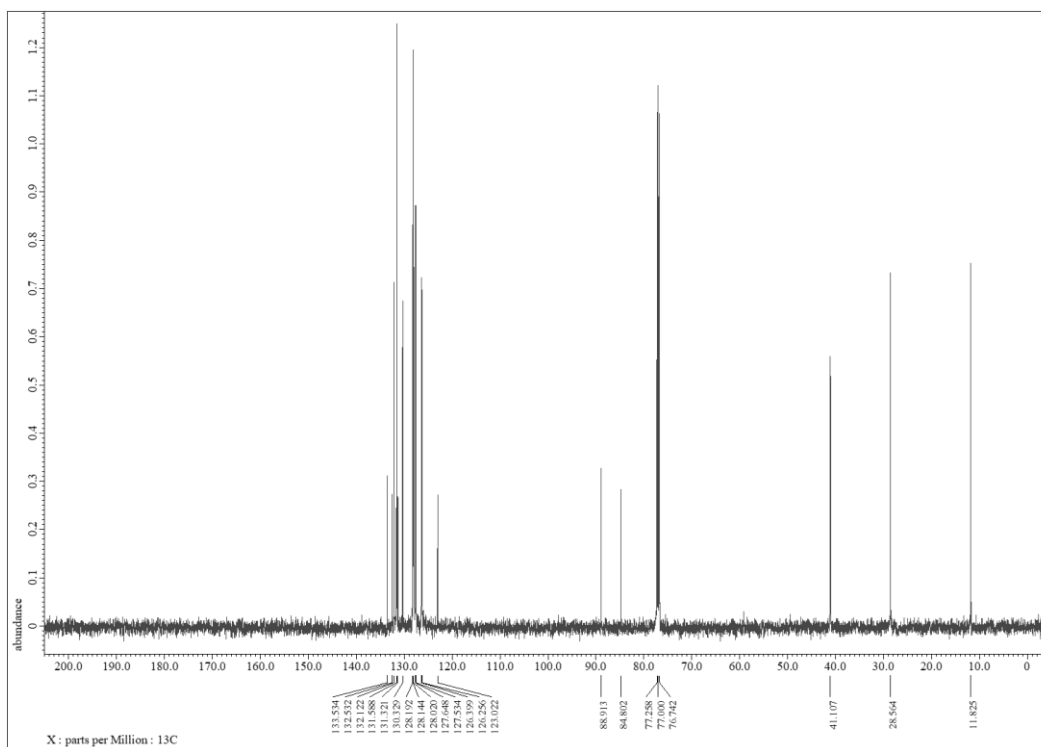

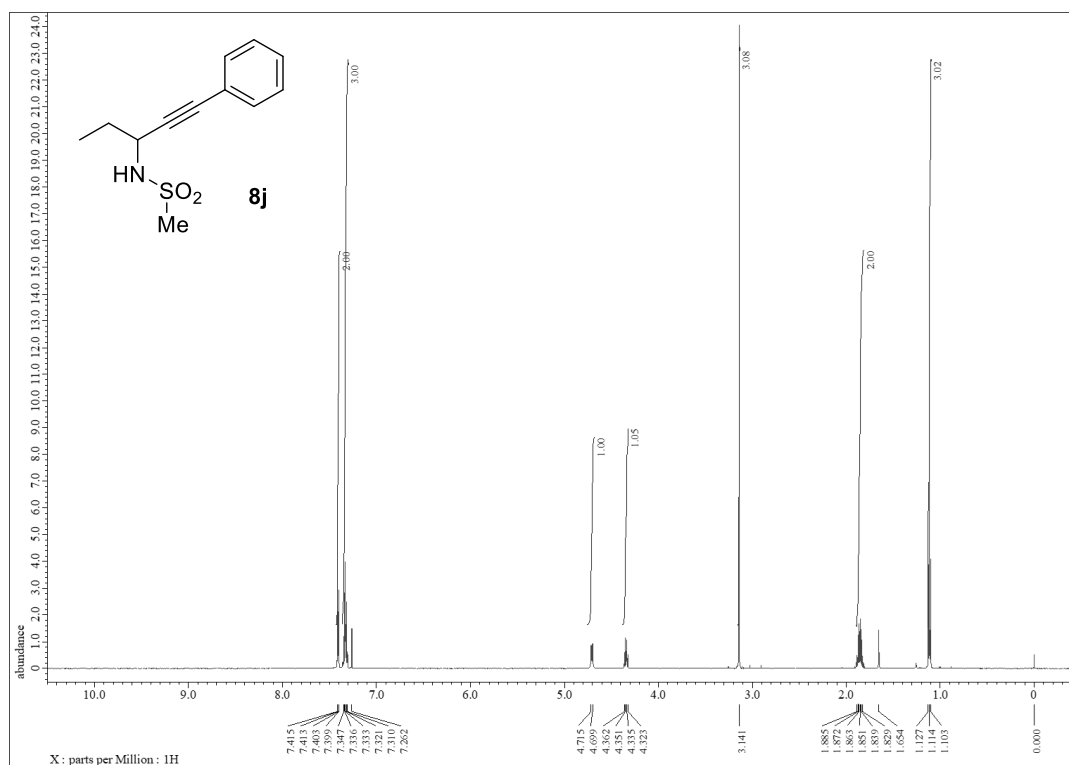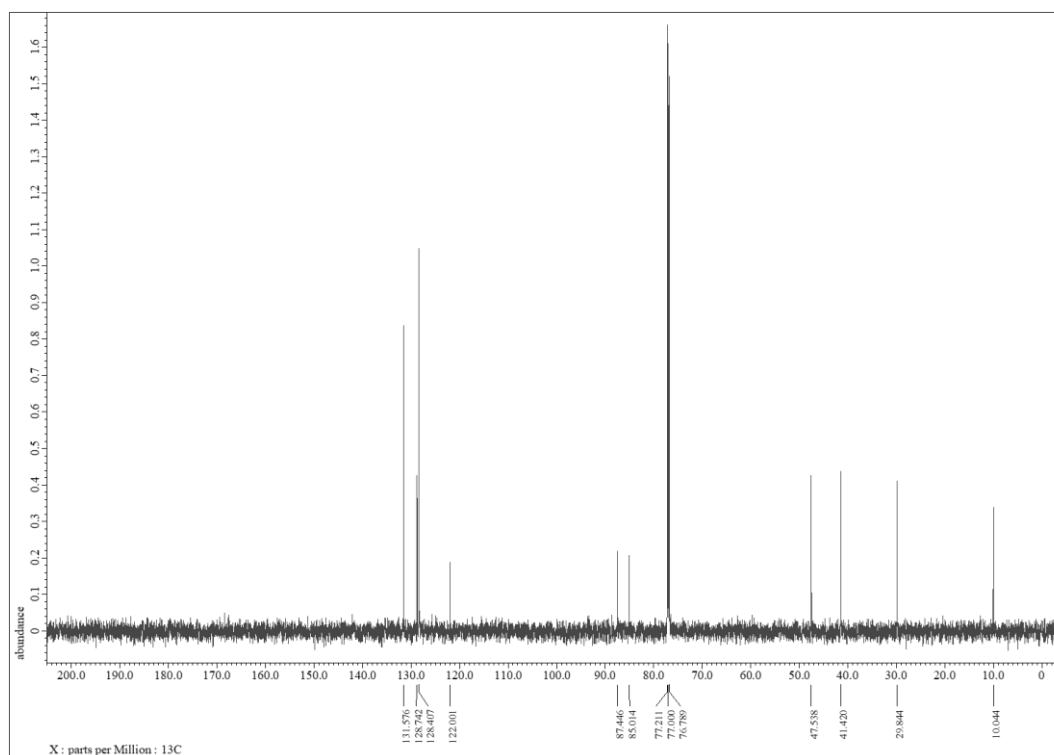

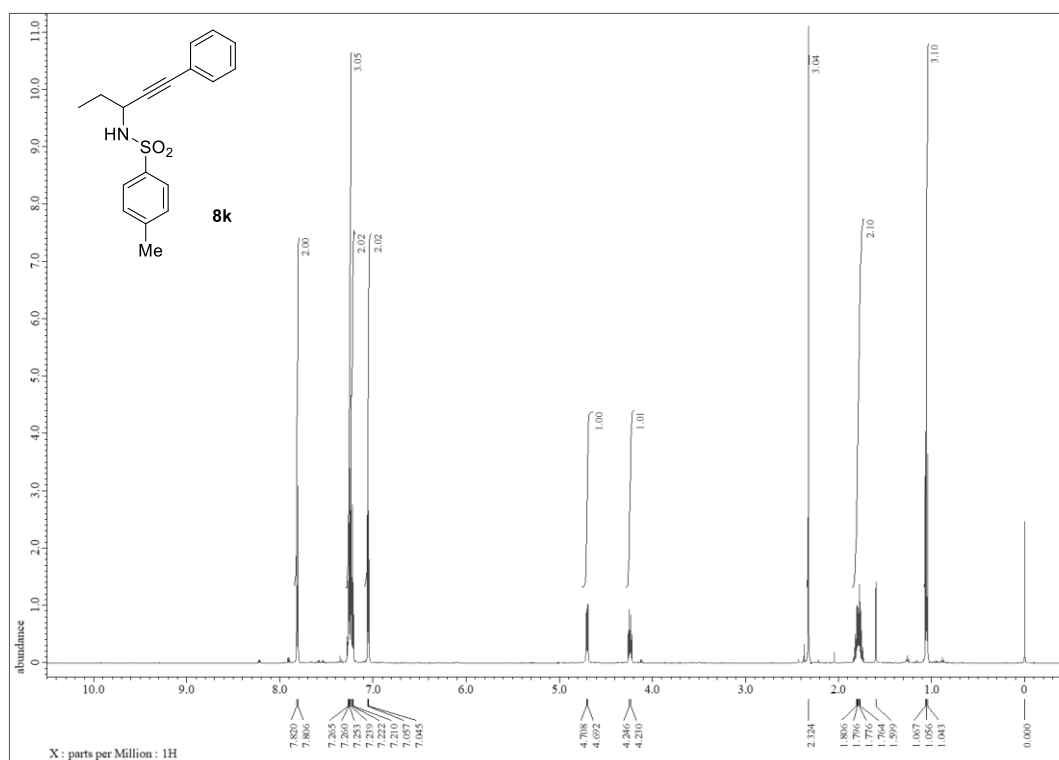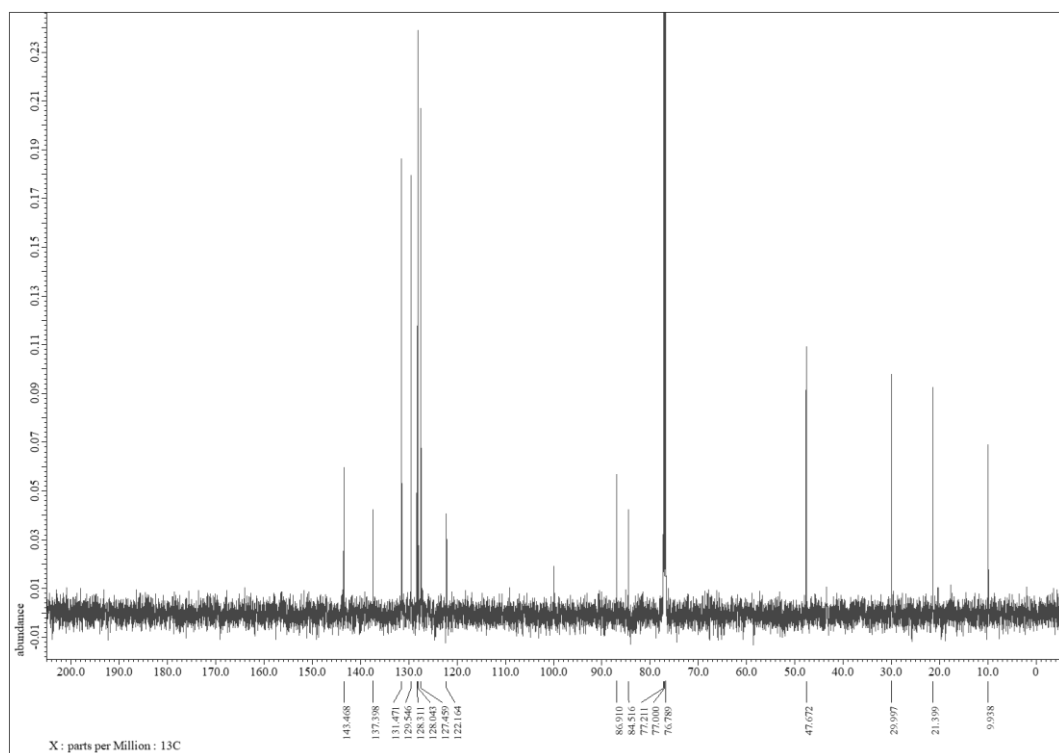

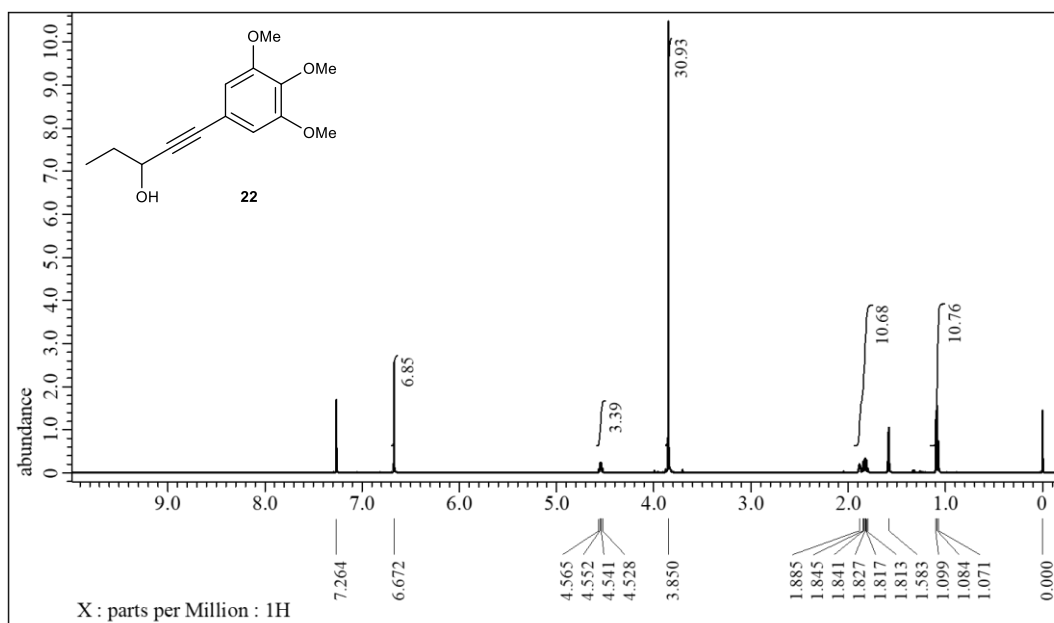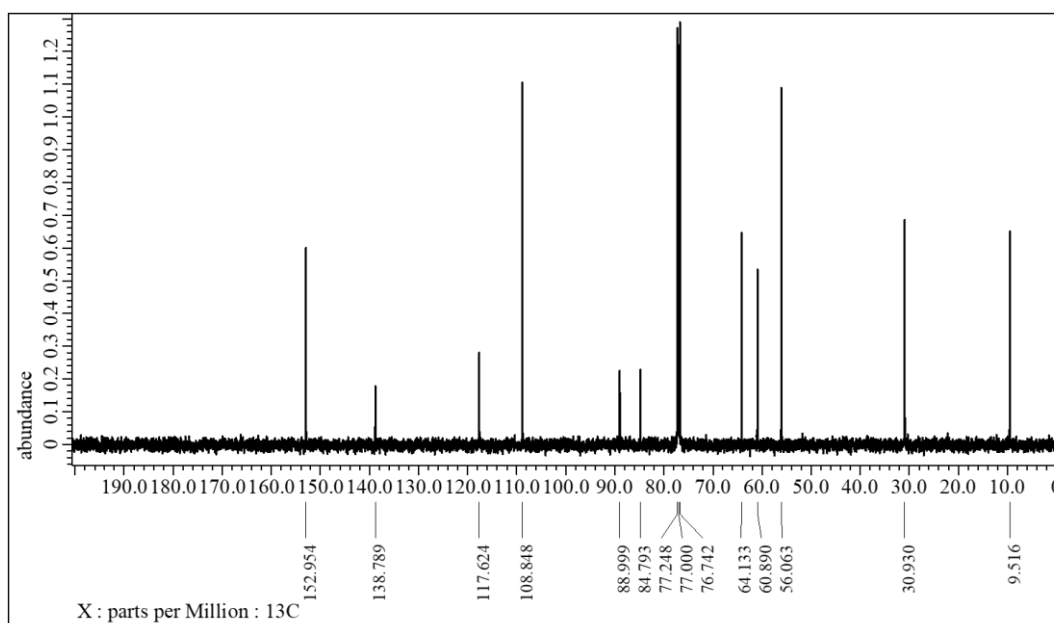

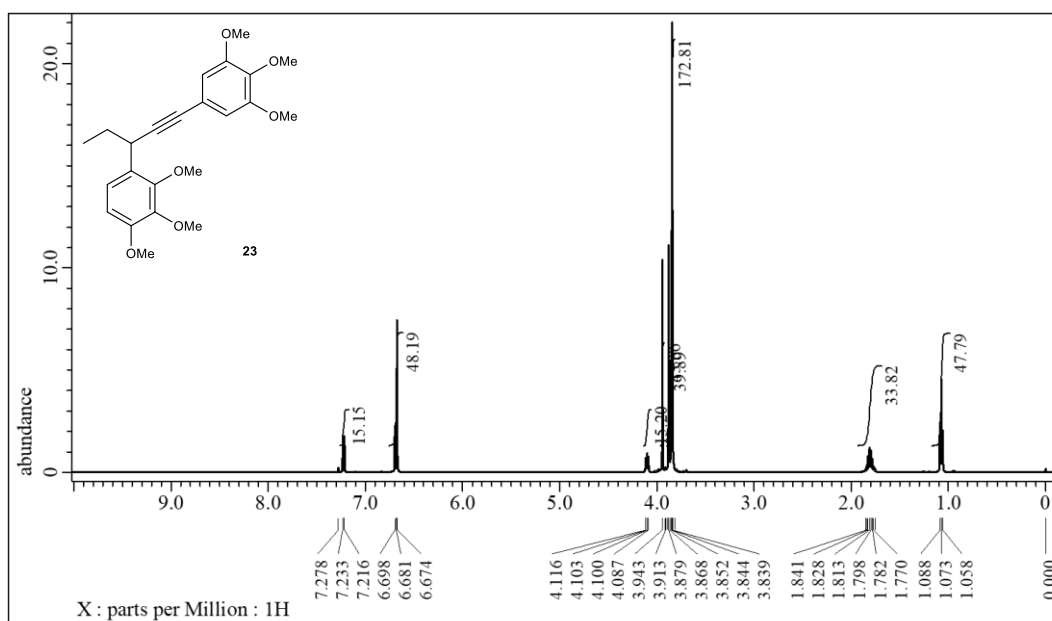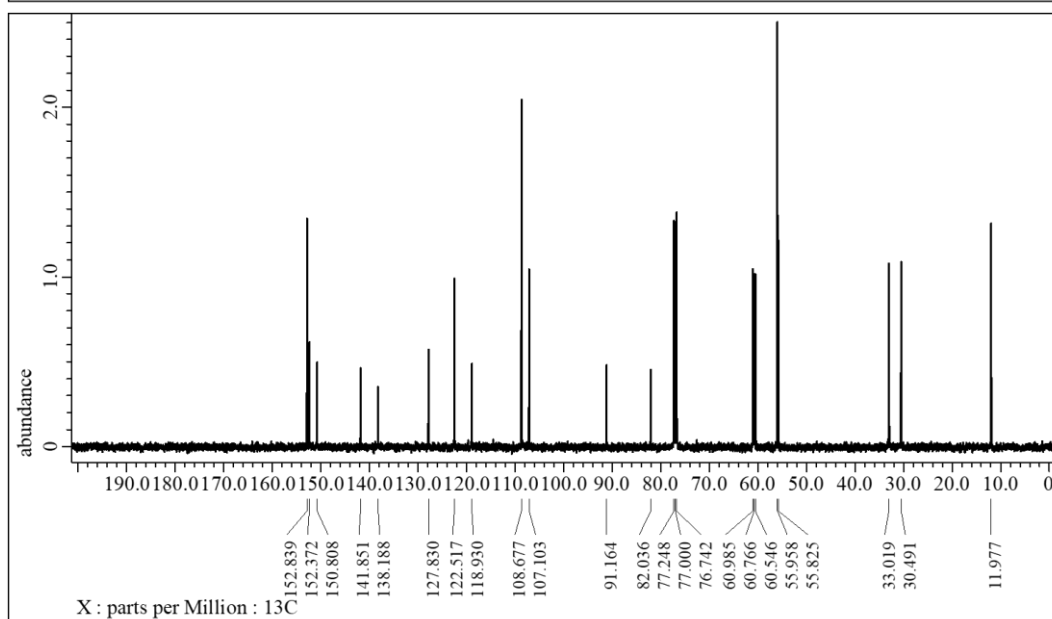

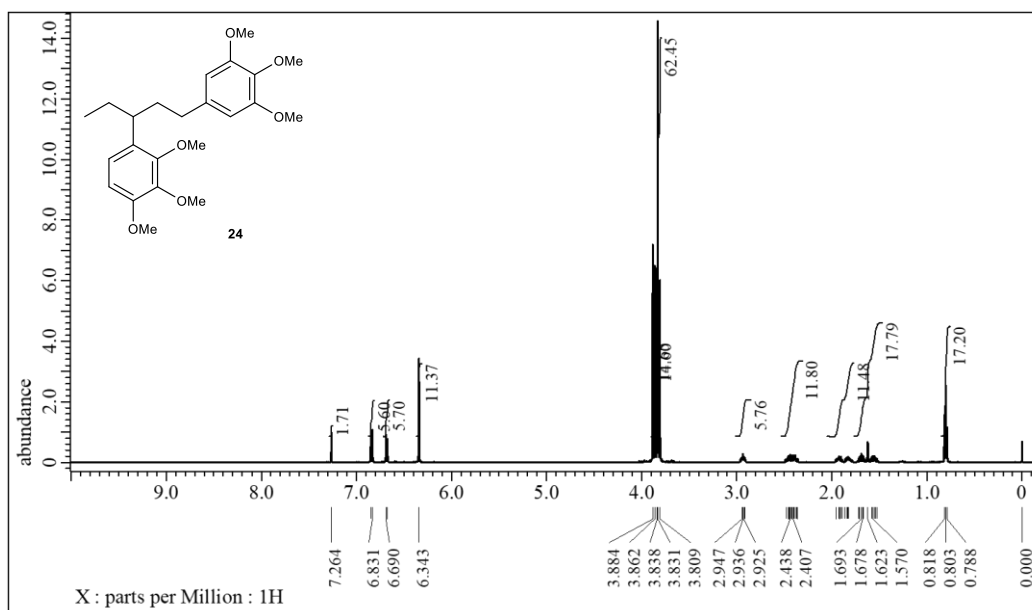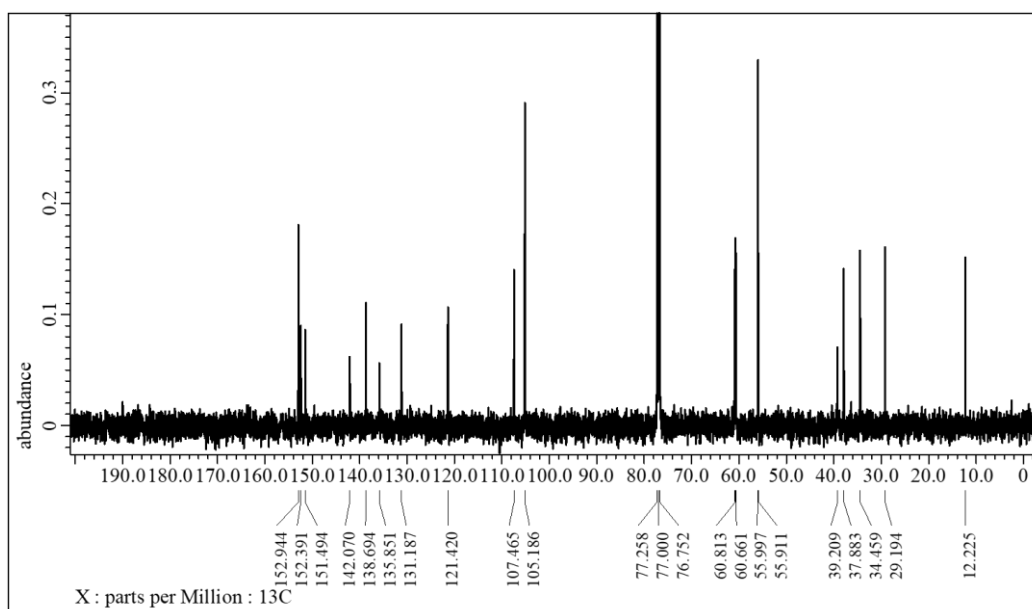

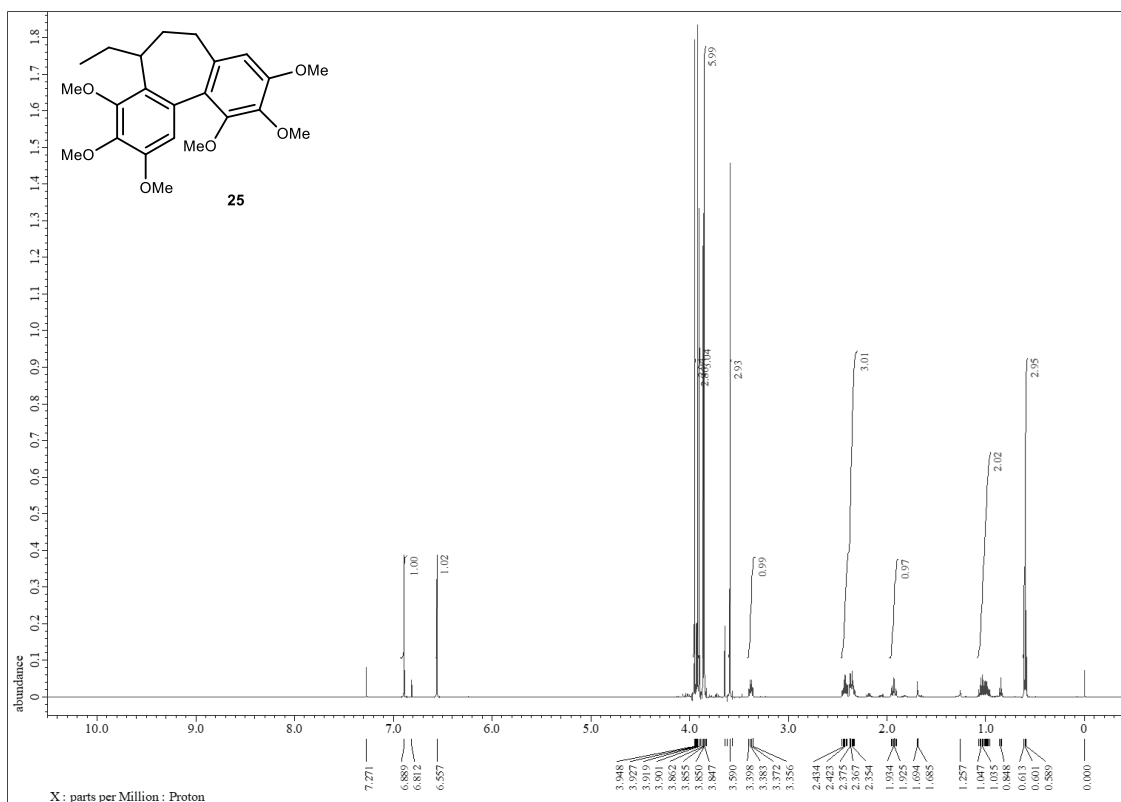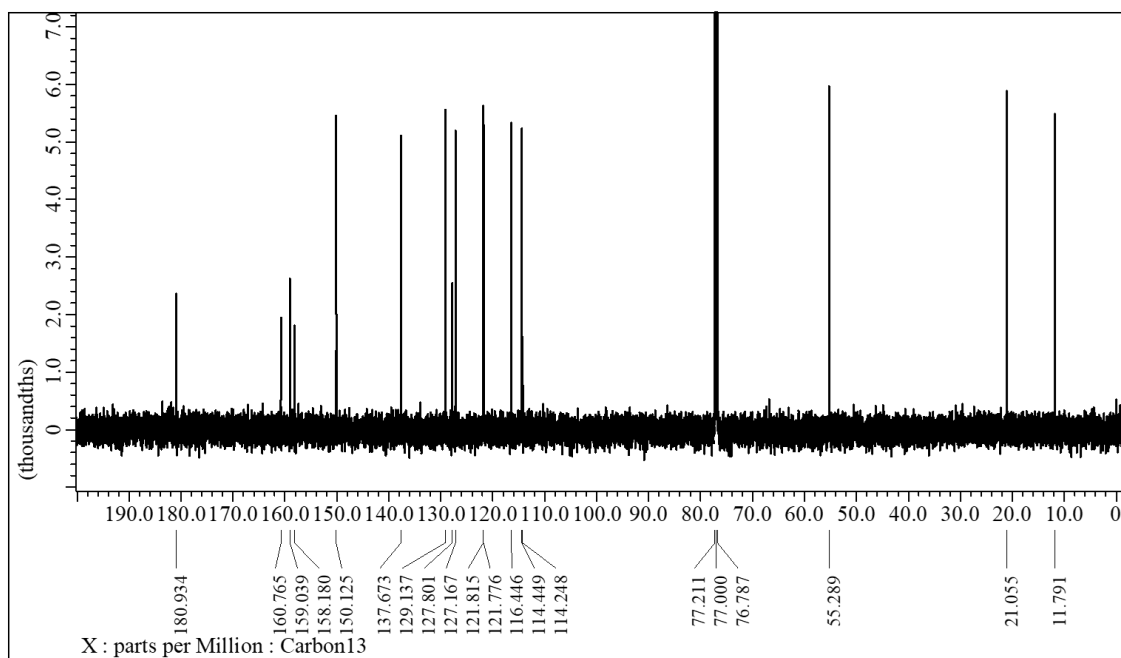

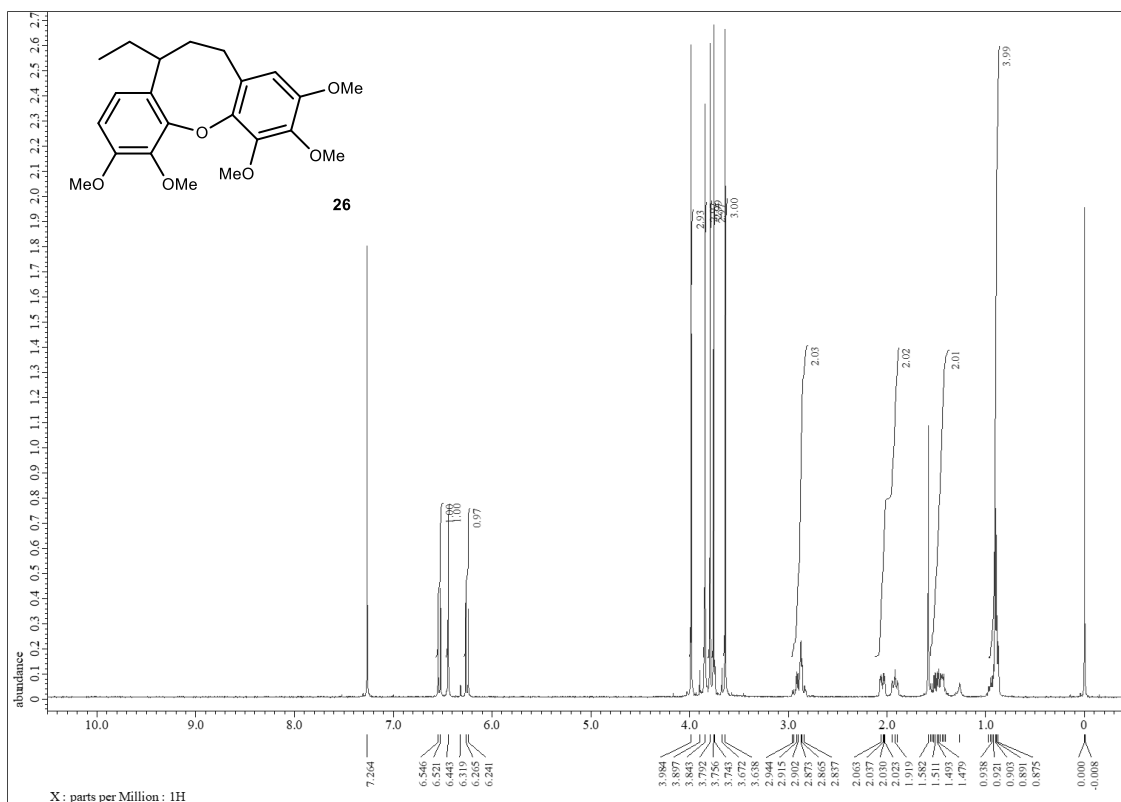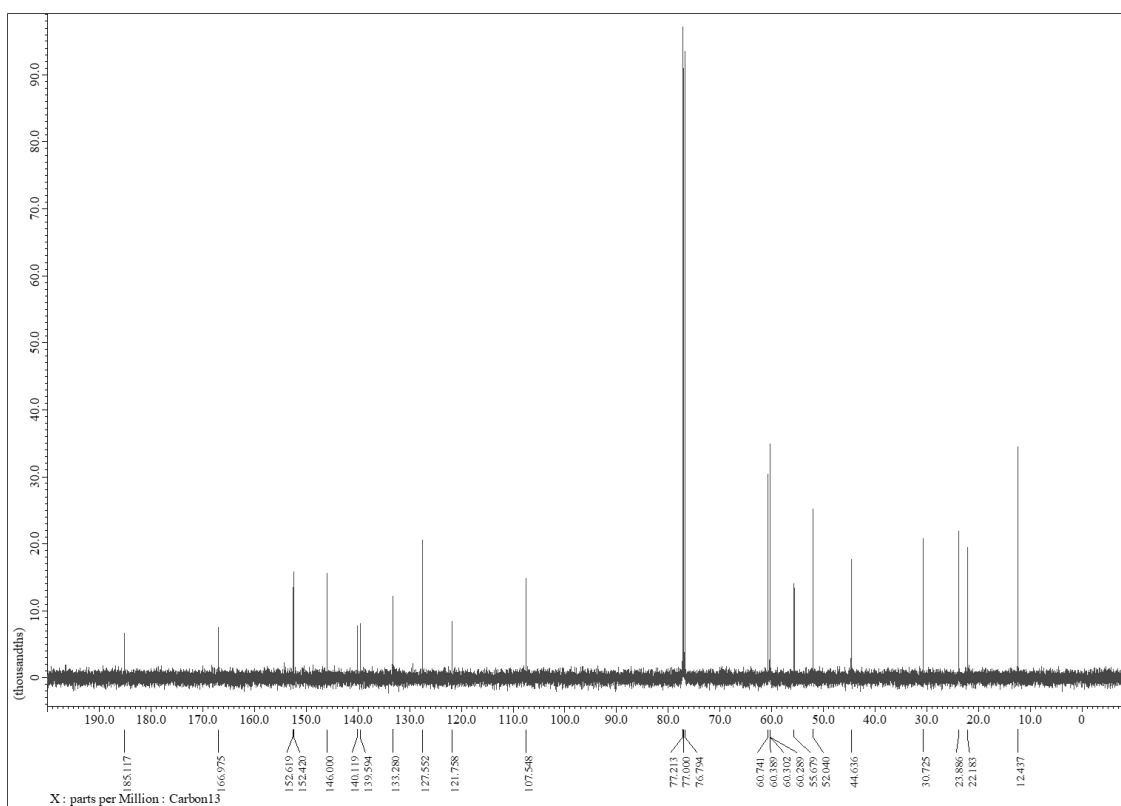

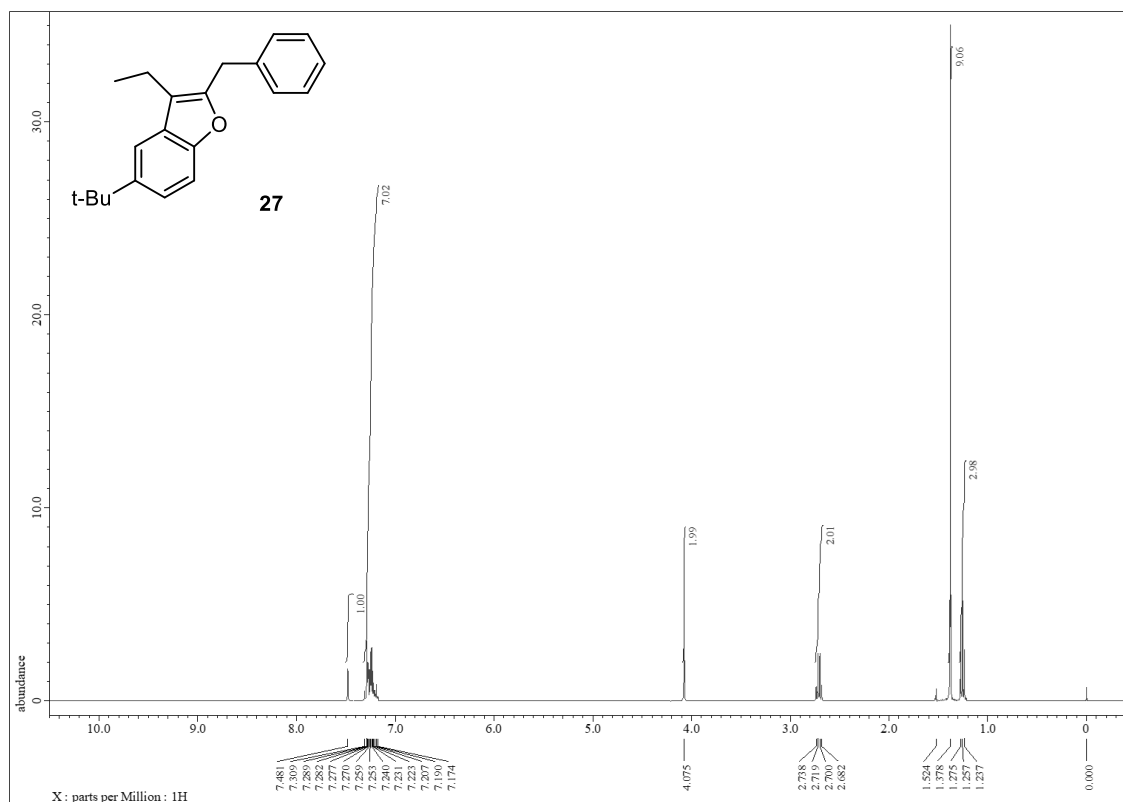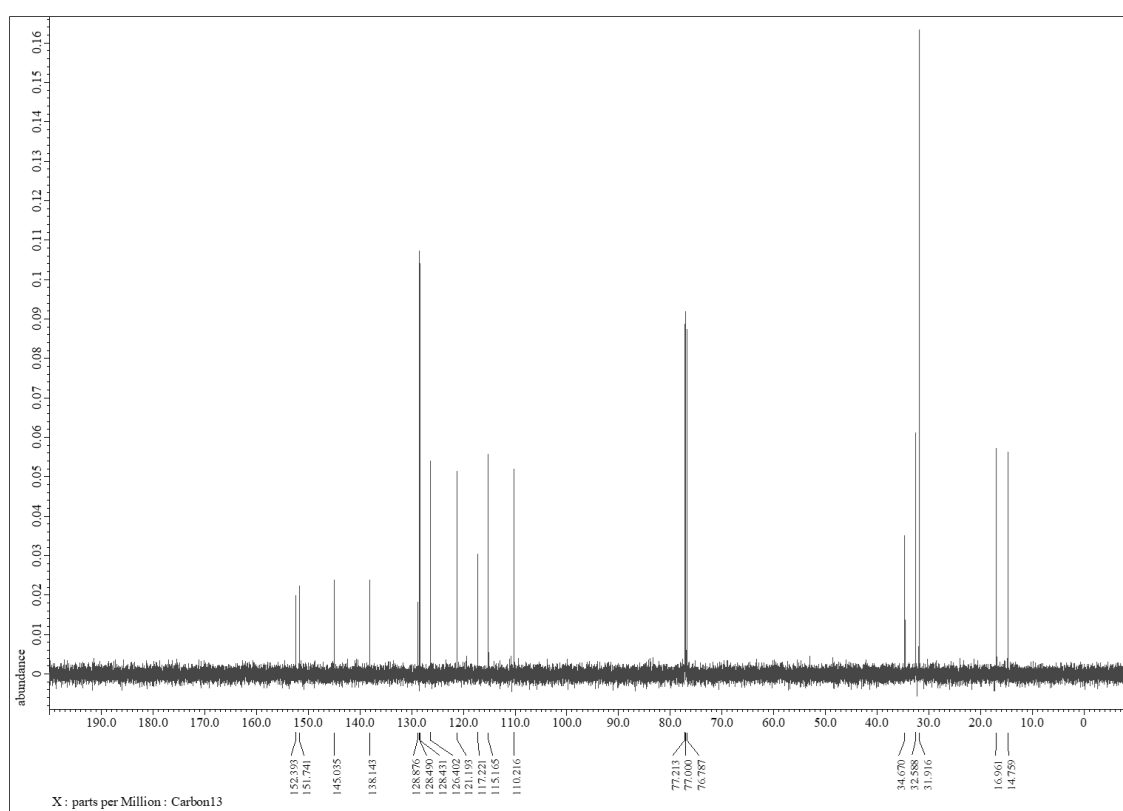

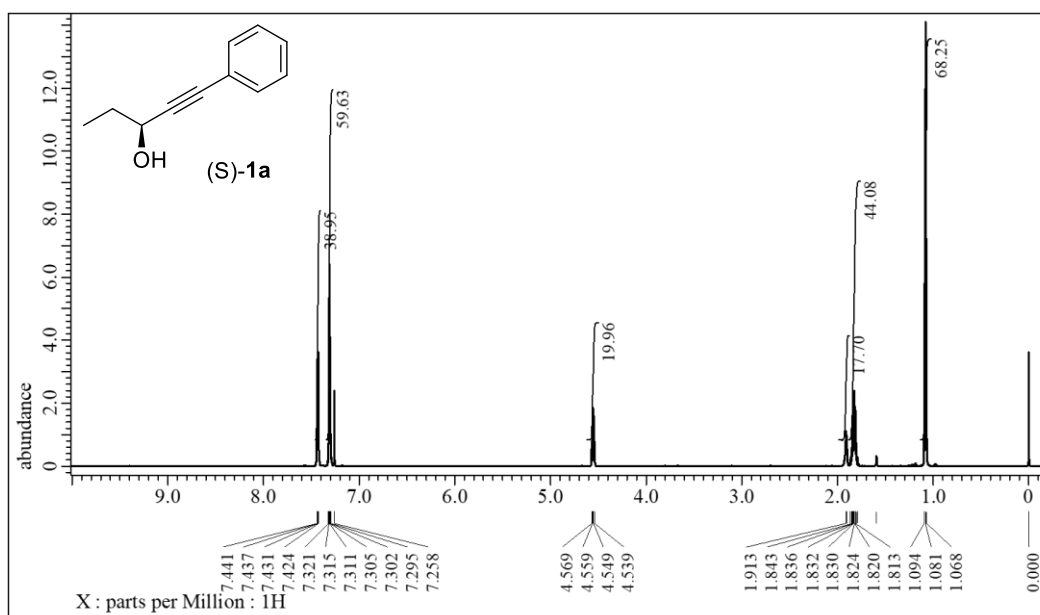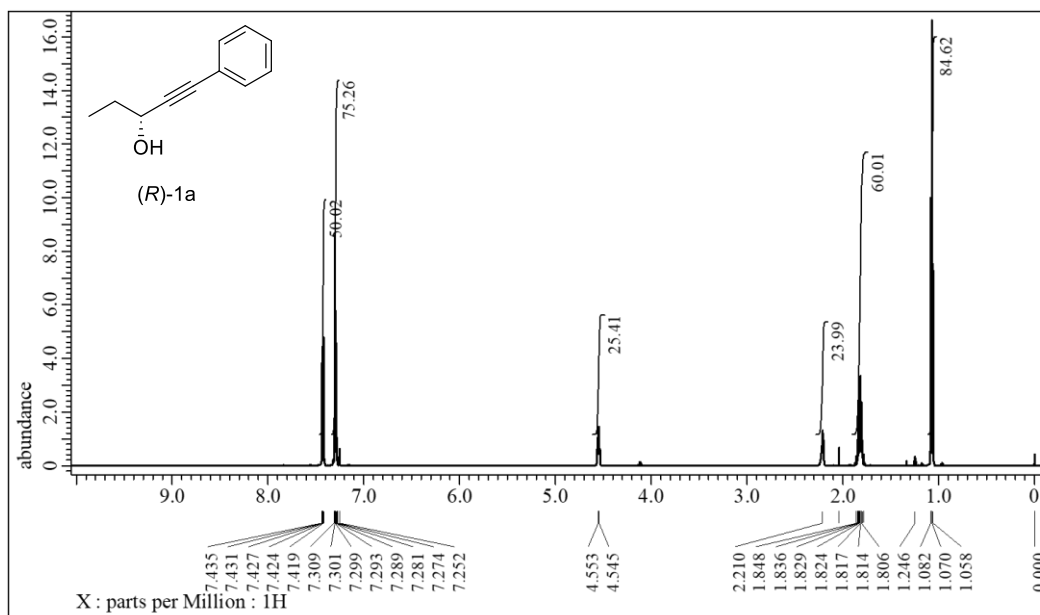

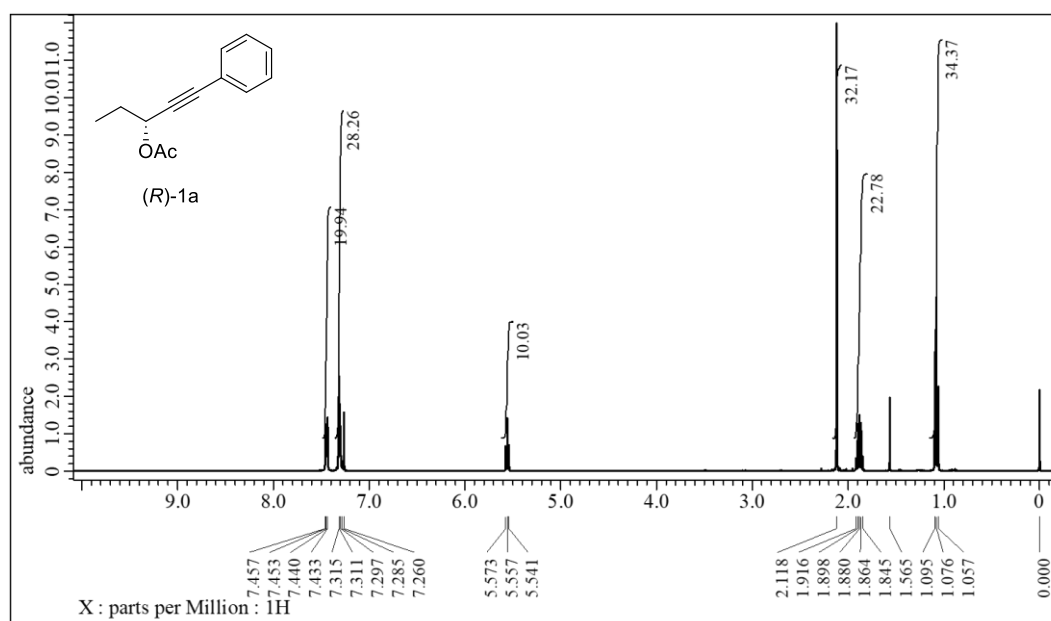

Supplement: Supplementary file 4 — Supplementary Data 1 [file 42004_2023_1048_MOESM4_ESM.pdf]
